# Importance of Binding Site Hydration and Flexibility Revealed When Optimizing a Macrocyclic Inhibitor of the Keap1–Nrf2 Protein–Protein Interaction

**DOI:** 10.1021/acs.jmedchem.1c01975

**Published:** 2022-02-02

**Authors:** Fabio Begnini, Stefan Geschwindner, Patrik Johansson, Lisa Wissler, Richard J. Lewis, Emma Danelius, Andreas Luttens, Pierre Matricon, Jens Carlsson, Stijn Lenders, Beate König, Anna Friedel, Peter Sjö, Stefan Schiesser, Jan Kihlberg

**Affiliations:** † Department of Chemistry—BMC, Uppsala University, Box 576, 75123 Uppsala, Sweden; ‡ Mechanistic and Structural Biology, Discovery Sciences, R&D, AstraZeneca, 43183 Mölndal, Sweden; § Department of Medicinal Chemistry, Research and Early Development, Respiratory and Immunology (R&I), BioPharmaceuticals R&D, AstraZeneca, 43183 Mölndal, Sweden; ∥ Science for Life Laboratory, Department of Cell and Molecular Biology, Uppsala University, Box 596, 75124 Uppsala, Sweden; ⊥ Drugs for Neglected Diseases Initiative (DNDi), 15 Chemin Camille-Vidart, 1202 Geneva, Switzerland

## Abstract

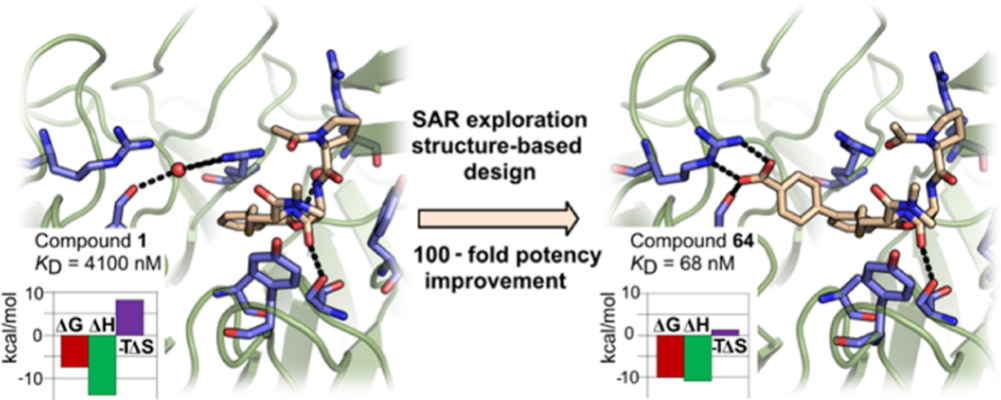

Upregulation of the transcription factor Nrf2 by inhibition of the interaction with its negative regulator Keap1 constitutes an opportunity for the treatment of disease caused by oxidative stress. We report a structurally unique series of nanomolar Keap1 inhibitors obtained from a natural product-derived macrocyclic lead. Initial exploration of the structure-activity relationship of the lead, followed by structure-guided optimization, resulted in a 100-fold improvement in inhibitory potency. The macrocyclic core of the nanomolar inhibitors positions three pharmacophore units for productive interactions with key residues of Keap1, including R415, R483, and Y572. Ligand optimization resulted in the displacement of a coordinated water molecule from the Keap1 binding site and a significantly altered thermodynamic profile. In addition, minor reorganizations of R415 and R483 were accompanied by major differences in affinity between ligands. This study therefore indicates the importance of accounting both for the hydration and flexibility of the Keap1 binding site when designing high-affinity ligands.

## Introduction

The human proteome has been estimated to consist of 100,000–1,000,000 binary protein-protein interactions (PPIs). Compounds modulating PPIs are therefore considered as an exciting but often challenging opportunity for drug discovery.^1^ The surface area buried in PPIs may reach up to 6000 Å^2^ with binding affinities between the two proteins ranging from high micromolar to picomolar ranges. The druggability of interactions between pairs of proteins has been classified based on the structure of the interacting epitopes.^2^ PPIs in which a short linear sequence from one protein binds in a small and well-defined pocket in the other protein are often suitable for inhibition by small molecules. Binding of a secondary structural motif, such as an α-helix from one protein, into a large groove on the other protein are more challenging. Finally, PPI interfaces formed by several peptide sequences from both proteins are often flat and featureless and therefore very challenging for small-molecule inhibitors. Macrocycles have been highlighted as being of particular interest for the modulation of challenging targets, such as PPIs.^3^ Analyses of macrocycle–target crystal structures have concluded that this originates from the size and ability of macrocycles to adopt disc- and sphere-like shapes that allow them to bind to targets that have large, pocket- or grove-shaped, or flat binding sites.^4,5^

**Figure 1:**
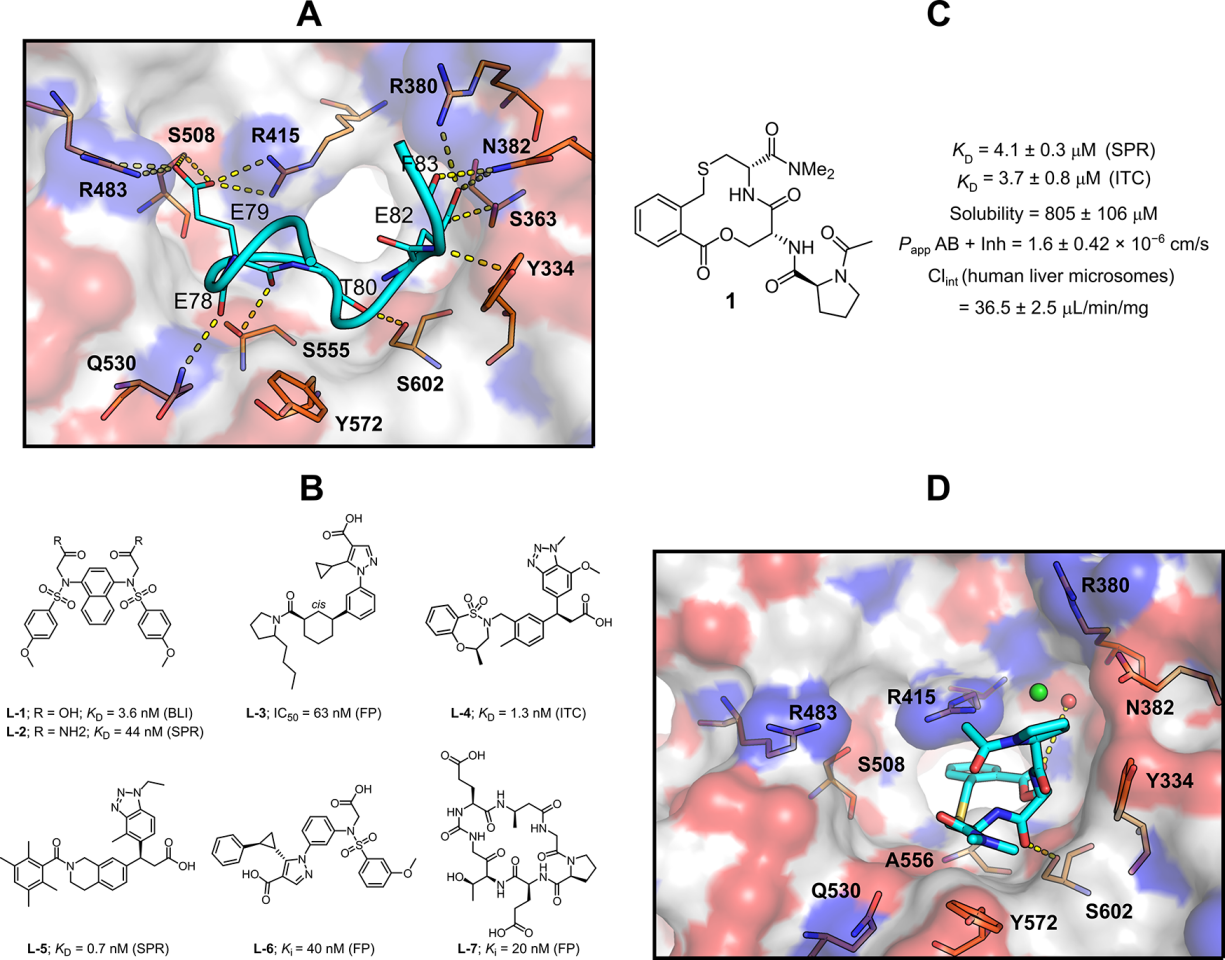
(A) Binding site of Keap1 bound to a truncated peptide from Nrf2 containing the ETGE motif (residues 76–84: LDEETGEFL) determined by X-ray crystallography (PDB ID: 1X2R).^8^ Keap1 is shown as a white surface with oxygen atoms in red and nitrogen atoms in blue, selected Keap1 residues are shown as orange sticks, Nrf2 is shown as a teal ribbon with selected side chains or carbonyl groups shown as sticks, while polar contacts are shown as yellow dashed lines. (B) Structures of members of the five series of inhibitors of the Keap1–Nrf2 PPI that have *in vitro* potencies below 100 nM (**L-4** and **L-5** belong to the third series). Potencies were determined by biolayer interferometry (BLI), surface plasmon resonance (SPR), fluorescence polarization (FP), and isothermal titration calorimetry (ITC). (C) Structure, binding activity, and *in vitro* ADME properties for compound **1**. Dissociation constants determined by SPR and ITC, permeability across a Caco-2 cell monolayer in the presence of inhibitors of the three major efflux transporters (*P*_app_ AB + inh), and intrinsic clearance (Cl_int_) in human liver microsomes. (D) Binding site of the complex of Keap1 and compound **1** (PDB ID: 6Z6A).^9^ Keap1 is shown as a white surface with oxygen atoms in red and nitrogen atoms in blue, selected Keap1 residues are shown as orange sticks, compound **1** is shown as teal sticks, polar contacts are shown as yellow dashed lines, a chloride ion is shown as a green sphere, and a bound water molecule is shown as a red sphere.

The nuclear factor erythroid 2-related factor 2 (Nrf2) upregulates the cellular defense system against oxidative stress by mediating the transcription of enzymes acting as antioxidants or being involved in detoxification.^6^ The Kelch-like ECH-associated protein 1 (Keap1) is the substrate adaptor unit of an E3 ligase that regulates the cellular concentration of Nrf2 by promoting its polyubiquitination and subsequent proteasomal degradation. Compounds that inhibit the Nrf2–Keap1 PPI are therefore of interest for the treatment of a large number of diseases caused by oxidative stress and inflammation, including metabolic and autoimmune disorders, as well as diseases affecting the lungs, liver, kidneys, gastrointestinal tract, and cardiovascular system.^6^ The C-terminal Kelch domain of Keap1 binds a high-affinity ETGE-motif and a low-affinity DLG-motif in the Nrf2–ECH homology 2 (Neh2) domain of Nrf2. The Neh2-binding pocket of Keap1 is large (550–780 Å^2^ buried surface area) and contains three arginine residues (R380, R415, and R483) which interact with glutamic and aspartic acid residues in the two Nrf2 motifs that are essential for high-affinity binding.^7^ These three arginine moieties form an intricate network of salt bridges and hydrogen bonds with side chains of E79 and E82 in the ETGE motif of Nrf2 (Figure 1A).^8^ Because of the size of the binding interface and its high polarity, the Nrf2–Keap1 PPI represents a challenging target for drug discovery.

Different lead generation strategies, including high-throughput screening, virtual screening, fragment-based approaches, and macrocyclization of peptides derived from Nrf2, have been used to find inhibitors of the Keap1–Nrf2 PPI. For example, the PubChem Bioassay database reports 528 compounds obtained from medium- and high-throughput screens of >337,000 compounds.^9^ However, the majority of these and other hits reported have low potency, with very few confirmed as inhibitors of the Keap1–Nrf2 PPI in orthogonal assays and/or by X-ray crystallography.^9,10^ In spite of the significant efforts invested to discover inhibitors of Keap1, only five structural series have members that show *in vitro* potencies below 100 nM, as determined by biophysical techniques, that is, **L-1** and **L-2**,^11,12^**L-3**,^13^**L-4** and **L-5**,^14,15^**L-6**,^16^ and **L-7**(17) (Figure 1B).

We recently reported a novel series of inhibitors of the Keap1–Nrf2 PPI discovered by docking of a set of macrocyclic cores obtained by mining of the natural product chemical space.^9^ The synthesis of only 13 members of the series provided compound **1**, which binds to Keap1 with *K*_D_ = 4 μM (Figure 1C). In addition, compound **1** has high aqueous solubility, low-to-moderate permeability across Caco-2 cell monolayers, and a medium *in vitro* clearance in human liver microsomes. Compounds from this series occupy a unique chemical space as compared to previously reported inhibitors of Keap1, and the crystal structure of the complex between **1** and Keap1 provides a platform for further optimization (Figure 1D). Herein, we report how compound **1** was optimized to a double-digit nanomolar inhibitor of the Keap1–Nrf2 PPI. We also present the analysis of crystal structures of five novel inhibitors with Keap1, as well as the *in vitro* characterization of three of the most potent inhibitors.

## Results and Discussion

### Overview of Potency Optimization

**Figure 2:**
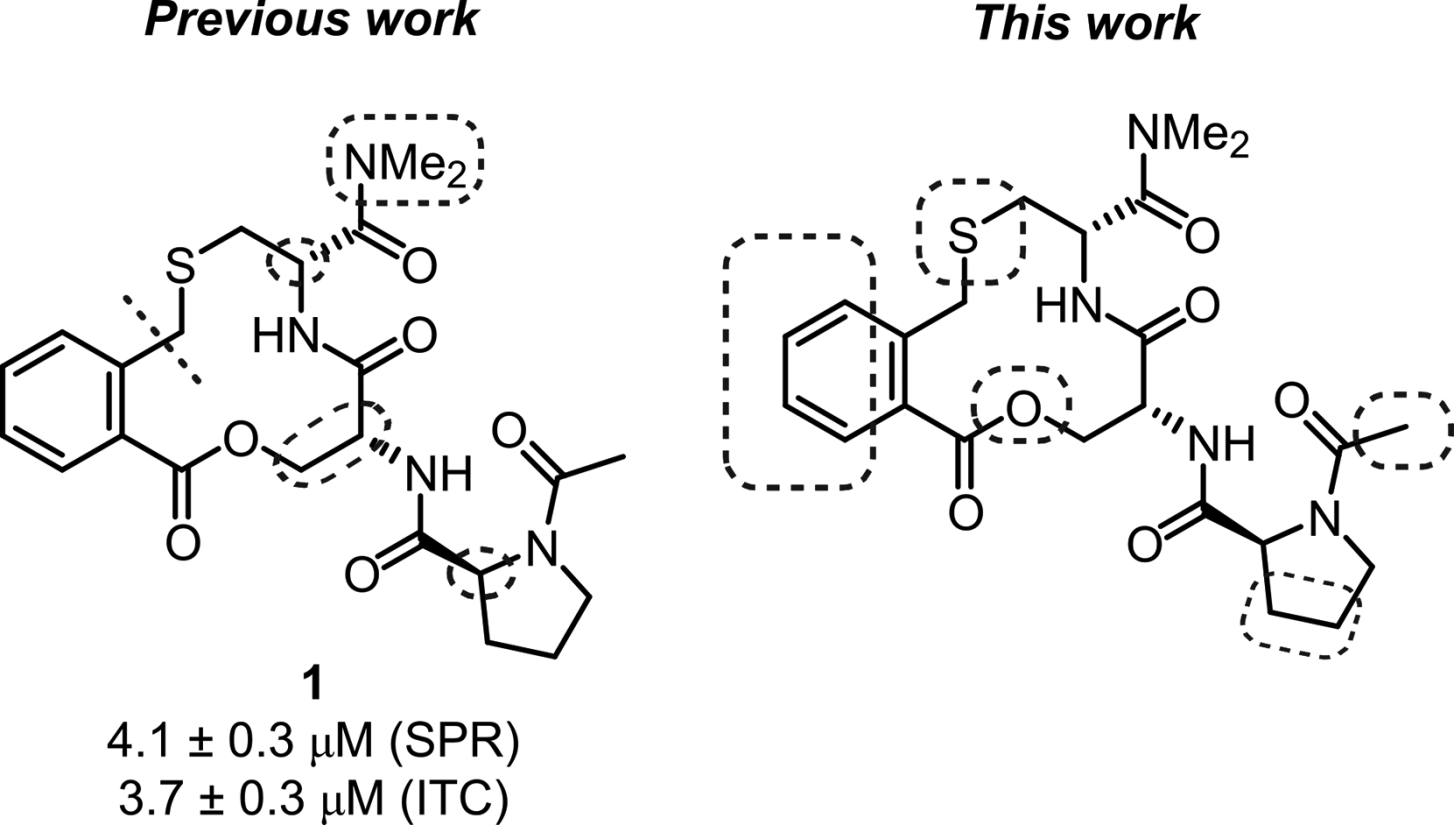
Overview of the SAR investigations that provided compound **1**(9) and the studies reported in this work.

Our previous structure–activity relationship (SAR) studies of the 13 compounds that provided compound **1** revealed the importance of the macrocycle, its ring size, the stereochemistry of the amino acid constituents, and the dimethyl amide for inhibition of the Keap1–Nrf2 PPI (Figure 2).^9^ Herein, we describe our optimization of the potency of this macrocycle series in two steps. First, opportunities for optimization were explored by variations within the macrocyclic ring, of the N-terminal proline and its attached acyl group, as well as by substitution on the phenylene ring. This approach identified the proline acyl group and the position of the phenylene ring *ortho* to the thiomethylene group as the most promising for more extensive optimization. For these two positions, molecular docking of diverse libraries was used to select smaller sets of compounds for synthesis.

All compounds synthesized were first evaluated as inhibitors of the Keap1–Nrf2 PPI by SPR using an inhibition in solution assay (ISA)^18^ or a direct binding assay (DBA). In the ISA, the potency of each compound as an inhibitor of the binding of Keap1 to a surface-immobilized peptide derived from Nrf2 was determined. As the ISA format is limited in its ability to differentiate inhibitors that display *K*_D_ values below the assay protein concentration (50 nM), the DBA format using an immobilized Keap1 domain was used for the most potent inhibitors. Further validation of the *K*_D_ values and collection of additional thermodynamic parameters were subsequently conducted for the most potent and informative compounds using ITC.

### Exploring the Macrocyclic Ring

**Figure 3:**
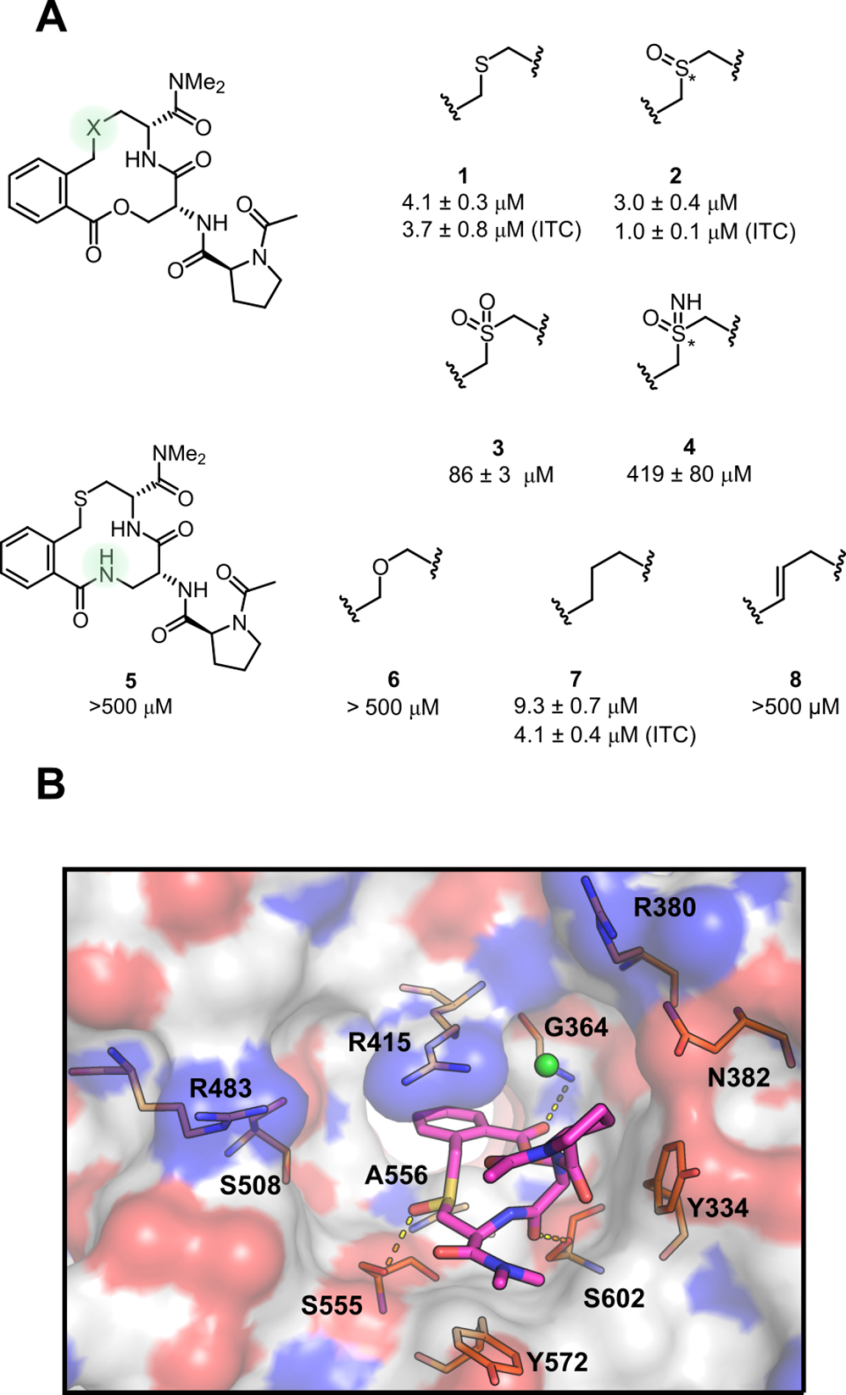
(A) Investigation of the role of the sulfur atom and lactone of **1** for inhibition of the Keap1–Nrf2 PPI. Dissociation constants were obtained by SPR using an ISA for **1–8** and by ITC for compounds **1**, **2**, and **7**. Mean values ± standard deviation derived from a minimum of three experiments are reported. (B) Crystal structure of the complex between Keap1 and compound **2** determined at a 2.3 Å resolution (magenta, PDB ID: 7Q5H). Keap1 is shown as a white surface with oxygen atoms in red and nitrogen atoms in blue. Selected Keap1 residues are shown as orange sticks. Hydrogen bonds between the two compounds and Keap1 are indicated by yellow dashed lines. A chloride ion is shown as a green sphere.

We began the exploration of the SAR of compound **1** by probing the role of the sulfur atom and the lactone in the macrocyclic ring (Figure 3). Oxidation of the sulfur atom to a sulfoxide (compound **2**) resulted in a 3-fold increase in potency according to ITC, whereas sulfone **3** and sulfoximine **4** were 20- and 100-fold less potent, respectively. Replacement of the lactone with a lactam provided the inactive compound **5**, revealing the importance of the ester group for the potency of this series of Keap1 inhibitors. Interestingly, substituting the sulfur atom of **1** with an oxygen atom (compound **6**) led to a dramatic reduction in potency, while the binding affinity was retained for the CH_2_-isostere **7**. Introduction of a trans-double bond resulted in the inactive compound **8**.

#### Rationalization of the SAR for Compounds **2–5**

Determination of the crystal structure of compound **2** bound to Keap1 at 2.3 Å resolution revealed that the sulfoxide oxygen atom of **2** forms a hydrogen bond with the hydroxyl group of S555 which may contribute to the slightly increased affinity compared to **1** (Figure 3B). Additionally, in the complex with **2**, the lactone carbonyl group forms a hydrogen bond with the backbone NH of G364 in Keap1, whereas this carbonyl group interacts with a crystalized water molecule in the complex with **1** (Figure 1D). The crystal structure showed an *S* configuration for the sulfoxide stereocenter of **2**. Apart from the additional hydrogen bond to S555, the change in the hydrogen bond network of the lactone, and some minor difference at the C- and N-termini of **2**, the complex between Keap1 and **2** closely resembles the one determined recently for compound **1** (Figure 3B).^9^ In brief, the phenylene moiety of **2** is wedged between the side chains of A556 and R415 facing toward the KELCH channel, while the C-terminal dimethyl amide is stacked against the side chain of Y572. Just as for **1**, a chloride ion bridges Keap1 and the serine NH of **2**, while the amide carbonyl group in the macrocyclic ring forms a hydrogen bond with the hydroxyl of S602.

Inspection of the crystal structures of **1** and sulfoxide **2** bound to Keap1 also allowed us to rationalize the loss of potency observed for compounds **3–5** (Figure 3B). While the sulfoxide oxygen atom of **2** is engaged in a hydrogen bond with S555, an additional oxygen or nitrogen atom as in **3** and **4** would be oriented toward a nonpolar region of Keap1. An additional atom would also force the macrocyclic ring of **3** and **4** to adjust its conformation to avoid a steric clash with the carbonyl group of the lactam linking Ser and Cys, thereby contributing to the reduced potency. For **5**, the NH of the lactam replacing the lactone of **1** would point toward a nonpolar surface of Keap1. In this orientation, the amide bond would not be able to compensate for desolvation by hydrogen bonding to Keap1 without major rearrangements of the binding site.

#### Rationalization of the SAR for Compounds **1**, **6**, and **7**

**Figure 4:**
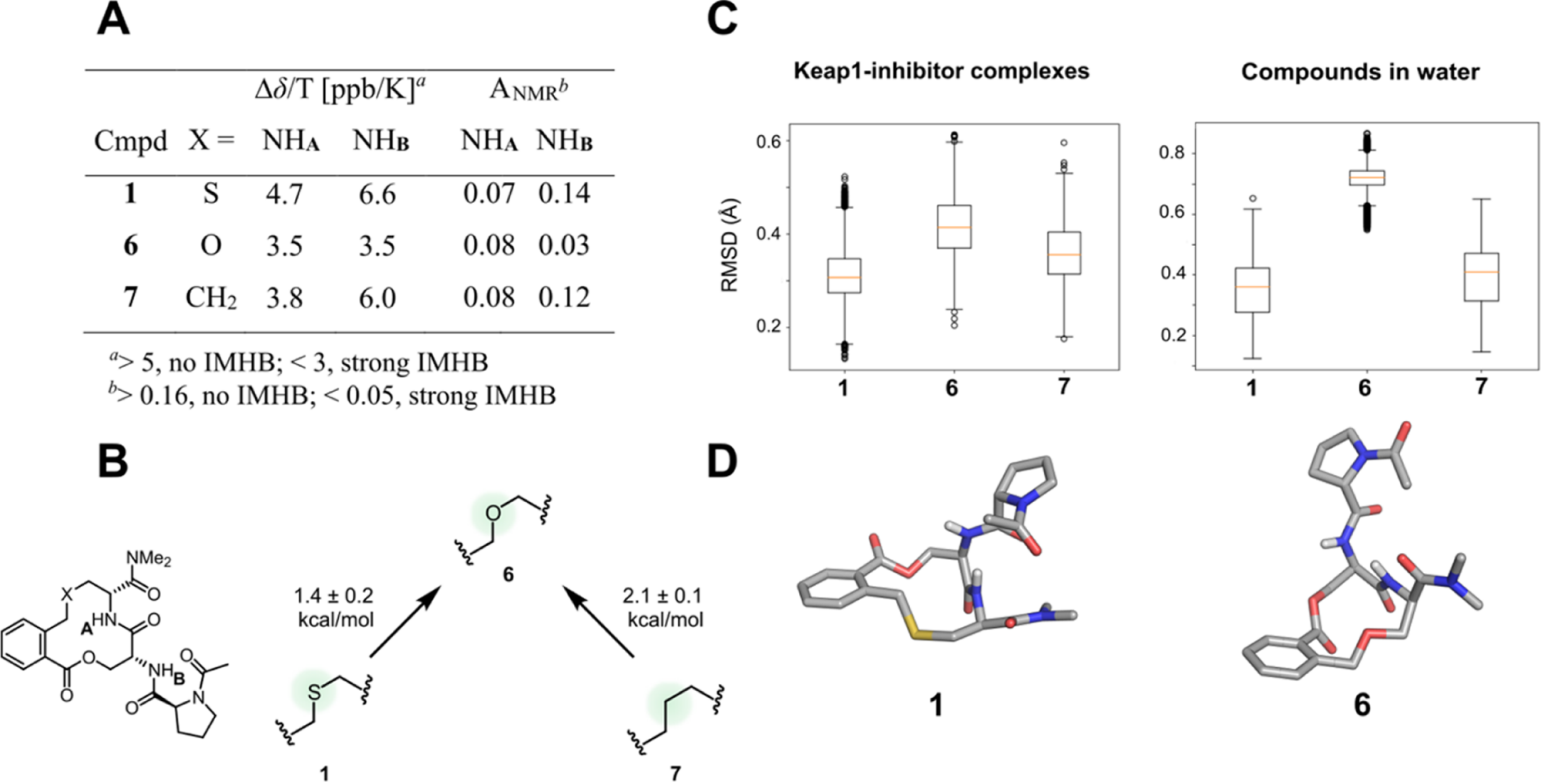
(A) Temperature and *A*_NMR_ coefficients for the NH protons of compounds **1**, **6**, and **7**. VT NMR experiments were performed in DMSO-*d*_6_. *A*_NMR_ coefficients were determined from the chemical shift difference between DMSO-*d*_6_ and CDCl_3_.^19^ (B) Calculated relative binding free energies (kcal/mol) rationalizing the differences in the activity of compounds **1**, **6**, and **7**. Binding free energies relative to compound **6** were calculated from molecular dynamics (MD) simulations using the free energy perturbation method.^20^ (C) Box plots of rmsd values (Å) for the macrocyclic ring atoms of compounds **1**, **6**, and **7** in MD simulation snapshots with respect to those of compound **1** in the crystalline complex with Keap1. (D) Bound conformation of compound **1** in the complex with Keap1 and a representative conformation of compound **6** from the MD simulations in an aqueous solution.

We performed NMR studies of compounds **1**, **6**, and **7** to get initial insight into the origin of the difference in potency between the oxygen analogue **6**, the parent thioether **1**, and the methylene analogue **7**. DMSO-*d*_6_ was used as the solvent to mimic the polar extra- and intracellular environment while still allowing monitoring of the exchangeable amide protons in the compounds. Variable-temperature (VT) NMR spectroscopy showed that the cysteine NH (NH_A_) is shielded from the surrounding solution, indicating it to be involved in a medium-strong intramolecular hydrogen bond (IMHB) in all three compounds (Figure 4A and Table S1). In addition, the serine NH (NH_B_) of compound **6** is involved in a strong IMHB, or is highly shielded, in contrast to NH_B_ of **1** and **7** (Δδ/*T* = 3.5 *vs* ∼6 ppb/K). This IMHB and/or shielding was further supported by the determination of the *A*_NMR_ coefficient,^19^ which was significantly lower for NH_B_ of compound **6**. Compound **6** therefore appears to adopt a different conformational ensemble than **1** and **7**, which could explain the reduced potency displayed by **6**.

The origin of the differences in inhibitory potencies displayed by compounds **1**, **6**, and **7** was further investigated using MD simulations of their complexes with Keap1 and when free in an aqueous solution. The crystal structure of Keap1 bound to **1** was used as a starting point for the simulations, and the other two compounds were modeled based on the binding mode of **1**. Calculation of relative binding free energies^20^ between compounds **1**, **6**, and **7** from the MD simulations correctly captured a loss of affinity for compound **6** relative to compounds **1** and **7** (Figure 4B). Analysis of extended MD simulations confirmed that compound **6** explores different conformations than **1** and **7** both in the complex with Keap1 and in water (Figure 4C). Root-mean-square deviations (rmsds) for the macrocyclic ring with respect to the Keap1-bound conformation of **1** deviated more from this reference conformation for **6** than for **1** and **7**, and the difference was most pronounced in water. Additional analysis of MD snapshots in water showed that the conformational change of the macrocyclic ring involved a reorientation of the oxygen atom in **6** as compared to the sulfur atom of **1**, a flip of the lactone, and adoption of a different orientation of the proline moiety versus the macrocyclic ring (Figure 4D). As a result of these conformational changes, an intramolecular hydrogen bond may be formed between the ether oxygen atom and NH_A_ in the ring of **6**, whereas NH_B_ becomes more shielded from the surrounding solution, both of which match the temperature coefficients observed by VT NMR for NH_A_ and NH_B_ of **6**.

### Conformational Ensemble of **1** in Water–DMSO

The above SAR analysis of compounds **1**, **6**, and **7** revealed a strong dependence of the potency of the inhibitors on the conformation adopted by the macrocyclic ring. We therefore determined the conformational ensemble populated by macrocycle **1** to understand if the Keap1-bound conformation was populated in solution or if conformationally restricted analogues should be prepared to increase the potency. The conformational ensemble was determined by deconvolution of the time-averaged NMR data using the NMR analysis of molecular flexibility in solution (NAMFIS) algorithm,^21^ which has been validated on numerous macrocycles and cyclic peptides.^22−28^ Due to limited aqueous solubility, **1** was dissolved in a mixture of water and DMSO-*d*_6_ (1:4 by volume) as a mimic of the aqueous environment inside a cell (Tables S2–S5).

**Figure 5:**
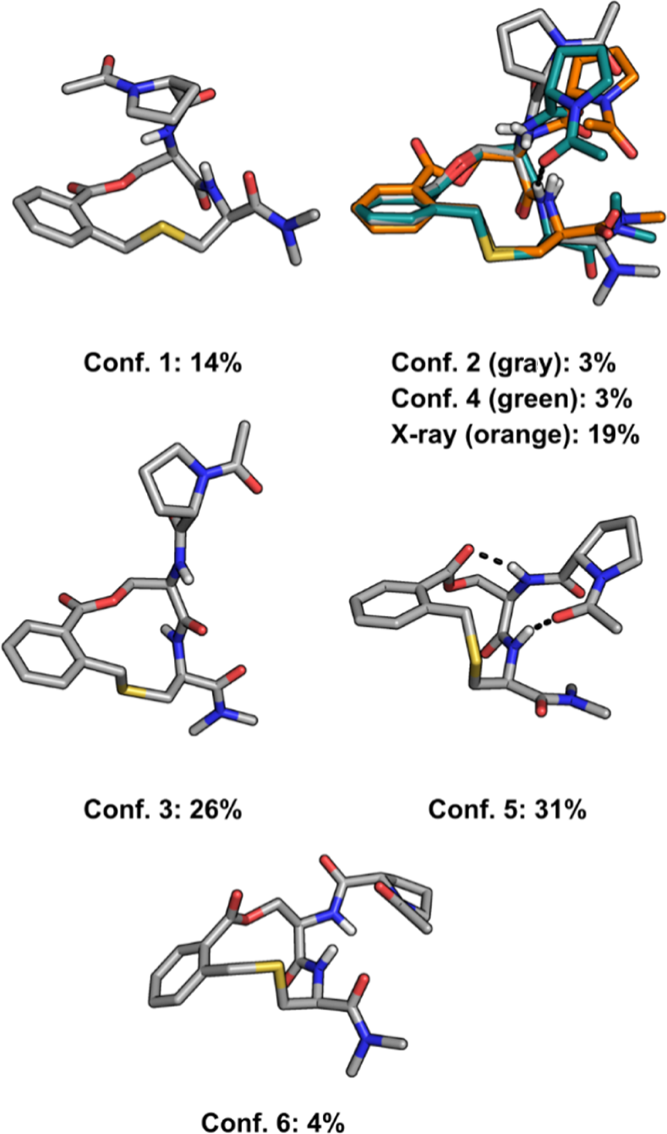
Conformational ensemble of compound **1** in H_2_O/DMSO-*d*_6_ (1:4), as selected by NAMFIS analysis. Intramolecular hydrogen bonds are shown as black dashed lines. Five conformational families were identified based on the comparison of the heavy atoms in the macrocyclic ring using an rmsd cutoff of 0.5 Å.

According to the NAMFIS analysis, the solution ensemble of compound **1** is populated by seven conformations, three of which are minor and represent only 10% of the ensemble (Figure 5). It is important to note that the Keap1-bound X-ray structure of **1** constitutes one of the major conformers of the ensemble (19%). The seven conformations showed some differences in the macrocycle core (rmsd 0.22–1.02 Å, Table S4), while larger differences for the orientations of the side chains resulted in overall conformations ranging from being quite similar to significantly different from each other (all heavy atom rmsd 1.09–3.47 Å, Table S5). Five conformational families were identified for the macrocycle core using an rmsd cutoff of 0.5 Å for the heavy atoms in the macrocyclic ring (Figure 2 and Table S4). The cores of conformations 1 (14%), 2, 4, and the X-ray structure (25% in total), 3 (26%), and 5 (31%) make up the four major families. Conformation 6, which constitutes the fifth core family, is only populated to 4%.

In summary, the target-bound conformation of **1** constitutes a significant proportion (19%) of the overall ensemble. This indicates that approaches to increase the proportion of the binding conformation in solution will not lead to a major increase in potency. Instead, we focused on increasing the binding interactions of our series of macrocycles with Keap1. First, the phenylene ring was explored, and then the N-terminal proline moiety was investigated.

### Exploring Substitution of the Phenylene Ring

**Figure 6:**
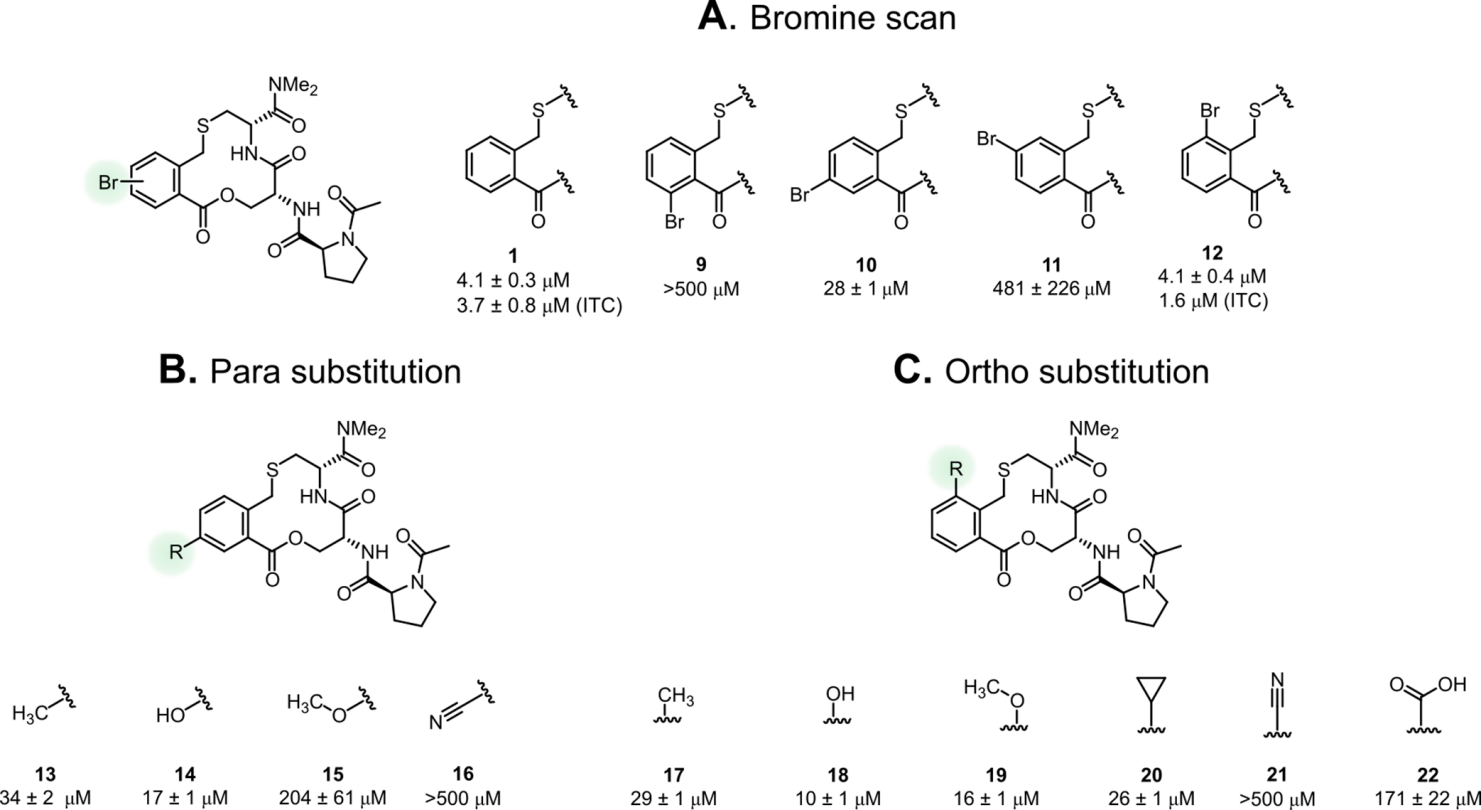
Investigation of the opportunities for substitution of the phenylene ring in optimization of **1** as an inhibitor of the Keap1–Nrf2 PPI. Structures and inhibitory activities of (A) compounds **9–12** that constitute a bromine scan of the phenylene ring, (B) compounds **13–16** that probe the position *para* to the thioether substituent of the phenylene ring and (C) compounds **17–22** which probe the *ortho*-position. Compound **1** is shown as reference. Dissociation constants were obtained by SPR using an ISA for **1** and **9–22** and also by ITC for compounds **1** and **12**. Mean values ± standard deviation derived from a minimum of three experiments are reported.

The effect of attaching substituents to the aromatic moiety of **1** on inhibition of the interaction between Keap1 and Nrf2 was investigated by the preparation of three series of derivatives, compounds **9–22** (Figure 6). In the first series, a bromine atom was used to scan the positions amenable for substitution, revealing steep differences in SAR (Figure 6A). Introduction of a bromine atom at either of the two *meta*-positions of the thioether (compounds **9** and **11**) led to a dramatic loss in activity. The *para*-position (compound **10**) appeared to allow substitution as only a 7-fold loss of potency was observed upon bromination. Interestingly, bromination at the position *ortho* to the thioether appeared even more promising as compound **12** had a potency almost identical to that of macrocycle **1**.

The two positions amenable for substitution were probed further using substituents having different steric and electronic properties. Substitution at the position *para* to the thioether revealed that small substituents, such as methyl or hydroxyl groups (compounds **13** and **14**), were tolerated but led to a 4–8-fold loss in activity (Figure 6B). Introduction of a methoxy group (compound **15**) led to a 50-fold reduction of potency, while a nitrile was detrimental for activity as compound **16** was more than 100-fold less potent than compound **1**. In general, substitution *ortho* to the thioether moiety proved to be better tolerated (Figure 6C). Introduction of either a methyl, hydroxyl, methoxy, or the somewhat bulkier cyclopropyl group (compounds **17–20**) only led to 2–7-fold reductions in potency. However, the nitrile-containing compound **21** was drastically less active (*K*_D_ >500 μM), while carboxylic acid **22** displayed approximately a 40-fold loss in potency.

The origin of the major reductions in potency displayed by nitriles **16** and **21** was investigated by free-energy perturbation (FEP)^20^ calculations based on MD simulations as for compounds **1**, **6**, and **7** (*cf.* above). The calculations captured the loss of affinity for **16** (+1.7 kcal/mol) and **21** (+0.8 kcal/mol) relative to compound **1**, although the calculated loss in free energy for compound **21** was substantially smaller than the corresponding experimental value (>2.9 kcal/mol). Extended MD simulations of the two Keap1 complexes, and of **16** and **21** in water, did not indicate a significant conformational deviation as compared to the bound conformation of compound **1** (Figure S1). However, the electron-withdrawing properties of the nitrile of compounds **16** and **21** could influence the π-cation interaction between the phenylene ring of **1** and the guanidine moiety of R415, which is essential for the stabilization of the complex with Keap1.^9^ This possibility was investigated by the analysis of force field interaction energies between compounds **16** and **21** and residues in the binding site of Keap1 from the extended MD simulations of the complexes. The highest per-residue difference as compared to compound **1** indeed involved R415 and was +3.6 ± 0.6 and +2.8 ± 1.3 kcal/mol for compounds **16** and **21**, respectively, pointing to weaker π-cation interactions with R415 as a potential reason for the reduced potency of **16** and **21**. In summary, the potencies of compounds **9–22** identified the position *ortho* to the thiomethylene group in the phenylene ring of **1** as the most promising for further optimization. In addition, the computational analysis of **16** and **21** revealed that electron-withdrawing groups should not be introduced at this position.

### Exploring the Role of Proline

**Figure 7:**
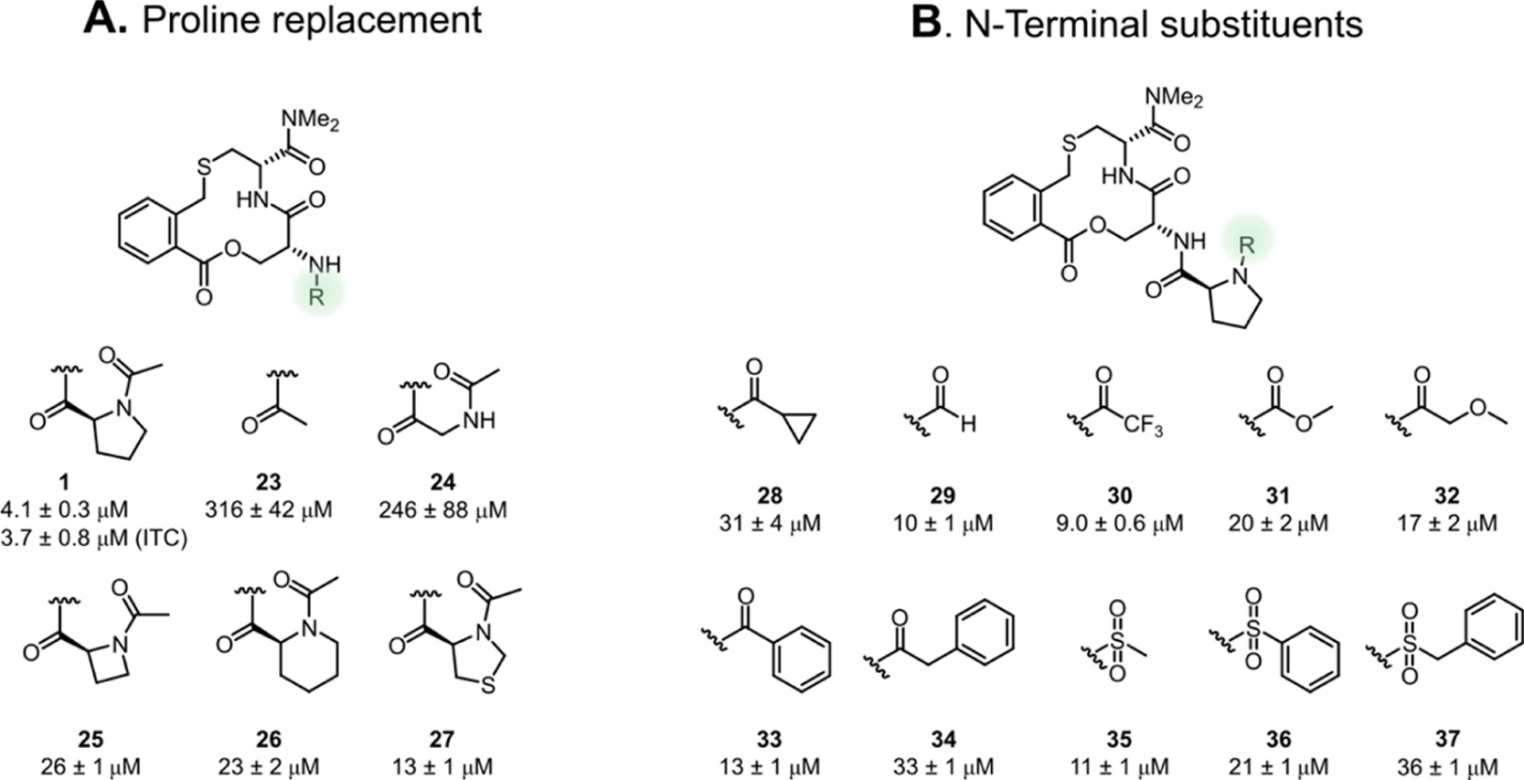
Investigation of the (A) role of the proline ring and the (B) substituent attached to the amino group of proline in **1** for the inhibition of the Keap1–Nrf2 PPI. Dissociation constants were obtained by SPR using an ISA for **1** and **23–37** and also by ITC for compound **1**. Mean values ± standard deviation derived from a minimum of three experiments are reported.

We investigated modifications of the N-terminus by replacements of the proline ring and by attachment of different substituents to the amino group of proline (Figure 7). Replacement of the proline moiety by an acetyl group or a *N*-acetyl glycine residue proved to be detrimental, with compounds **23** and **24** showing a 60–80-fold loss in potency in the ISA. This confirmed the importance of the proline ring for the inhibitory activity, as indicated previously by inversion of the stereochemistry at proline.^9^ Ring-contracted and ring-expanded analogues **25** and **26** resulted in an approximately 6-fold loss in potency, while incorporation of a sulfur atom led to a 3-fold loss in potency (compound **27**).

Changing the electronics and sterics of the proline *N*-acyl group was tolerated, as revealed by compounds **28–34** which displayed 2–8-fold losses in potency as compared to **1**. Sulfonamides (*cf.***35–37**) were also tolerated at this position with similar decreases in potency as for **28–34**. Based on the results obtained with compounds **23–37**, we concluded to keep the proline residue and that the incorporation of structurally diverse groups at its N-terminus constitutes an opportunity for further optimization of **1**.

### Initial Design of Inhibitors by Growing from Proline

**Figure 8:**
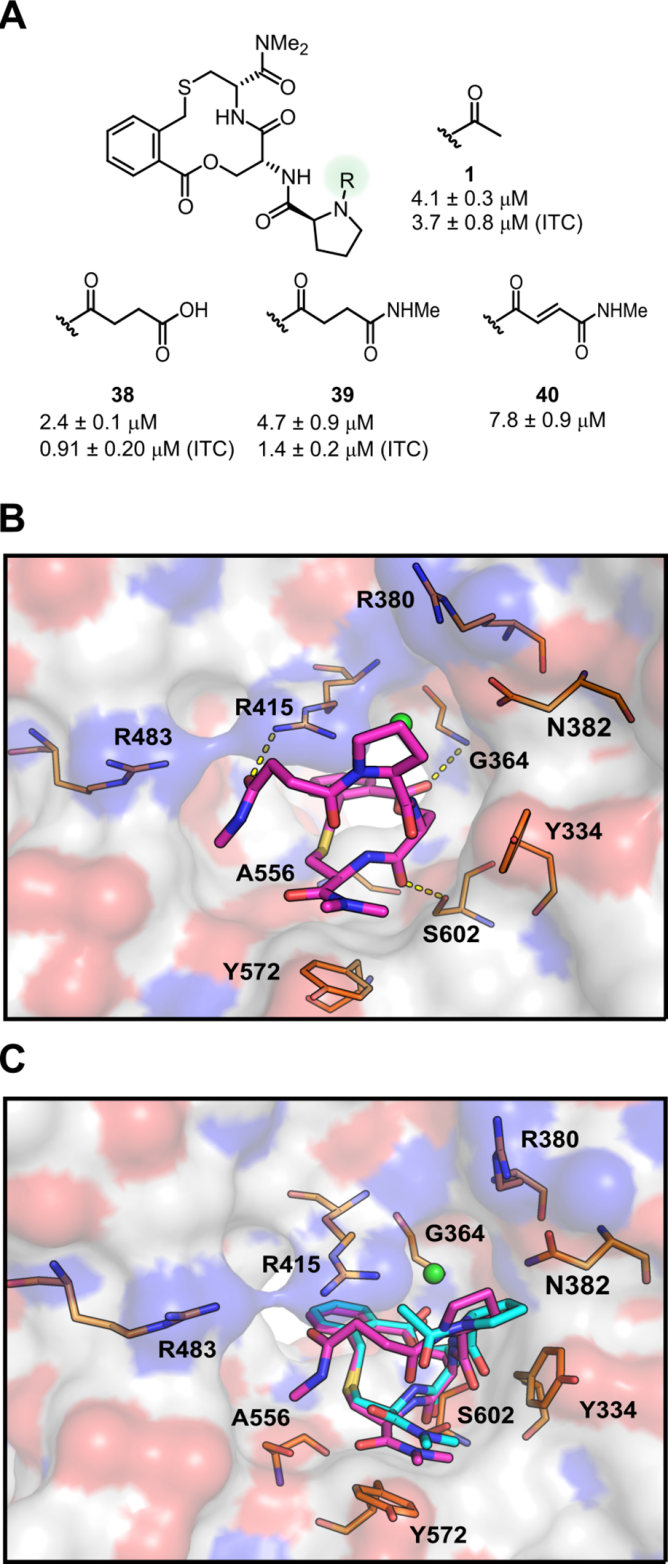
Initial attempts of the structure-based design of Keap1 inhibitors forming interactions with R415. (A) Structures and inhibitory activities of compounds **38–40**. Compound **1** is shown for reference. Dissociation constants were obtained by SPR using an ISA for **1** and **38–40** and also by ITC for compounds **1**, **38**, and **39**. Mean values ± standard deviation derived from a minimum of three experiments are reported. (B) Crystal structure of the complex between Keap1 and compound **39** determined at a 2.6 Å resolution (PDB ID: 7Q6Q). (C) Superimposition of the complexes of compound **39** (magenta) and compound **1** (teal, PDB ID: 6Z6A) with Keap1. Keap1 is shown as a white surface with oxygen atoms in red and nitrogen atoms in blue in (B,C). Selected Keap1 residues are shown as orange sticks. Hydrogen bonds to Keap1 are indicated by yellow dashed lines. A chloride ion is shown as a green sphere. For clarity, only Keap1 from the complex with compound **39** is shown.

Inspection of the Keap1-bound crystal structure of **1**(9) (PDB ID: 6Z6A, Figure 1C) shows that the proline *N*-acetyl group is directed toward R415, making it a suitable vector for introducing additional interactions with Keap1. In addition, our exploration of the role of the proline residue found that the *N*-acetyl group could be replaced with only minor losses in potency (*cf.*Figure 7B). We therefore synthesized eight compounds containing different types of hydrogen bond acceptor and donor groups linked to proline *via* an aliphatic chain with the aim of forming additional interactions with R415. Only three compounds (**38–40**) possessed *K*_D_ values below 10 μM (2–8 μM) in the ISA (Figure 8A). Succinic acid **38** showed a 4-fold increase in potency relative to compound **1**, as determined by ITC, suggesting this to be a substituent having an appropriate length to reach R415. We then investigated if a neutral analogue of succinate **38** could retain potency. Monomethyl amide **39** showed a potency in between that of **38** and **1** according to ITC. Rigidification of the carbon chain with a double bond led to the less potent compound **40**. The structures and inhibitory potencies for the five compounds with even higher *K*_D_ values (14–31 μM) are presented in the Supporting Information (Scheme S1 and Compounds **S1–S5**).

Determination of the crystal structure of **39** bound to Keap1 at a 2.6 Å resolution revealed that the carbonyl group of the methyl amide in **39** formed a hydrogen bond with the side chain of R415, demonstrating the feasibility of targeting this residue (Figure 8B). As observed for sulfoxide **2**, compound **39** was bound by Keap1 essentially as compound **1**, that is, with the phenylene moiety pointing toward the KELCH channel, the C-terminal dimethyl amide stacked against the side chain of Y572, while polar contacts were mediated by a chloride ion and hydrogen bonds to G364 and S602. The hydrogen bonding of the methyl amide of **39** to R415 of Keap1, together with the conserved orientation and contacts of inhibitors **1**, **2**, and **39** with Keap1, convinced us to attempt to optimize the potency of **1** by molecular docking of virtual libraries designed to establish interactions with the side chain of R415. The libraries were also designed to contain compounds that would allow interactions with R380 and R483, the two other arginine residues in the binding site of Keap1.

### Optimization Using Libraries Grown from Proline

**Figure 9:**
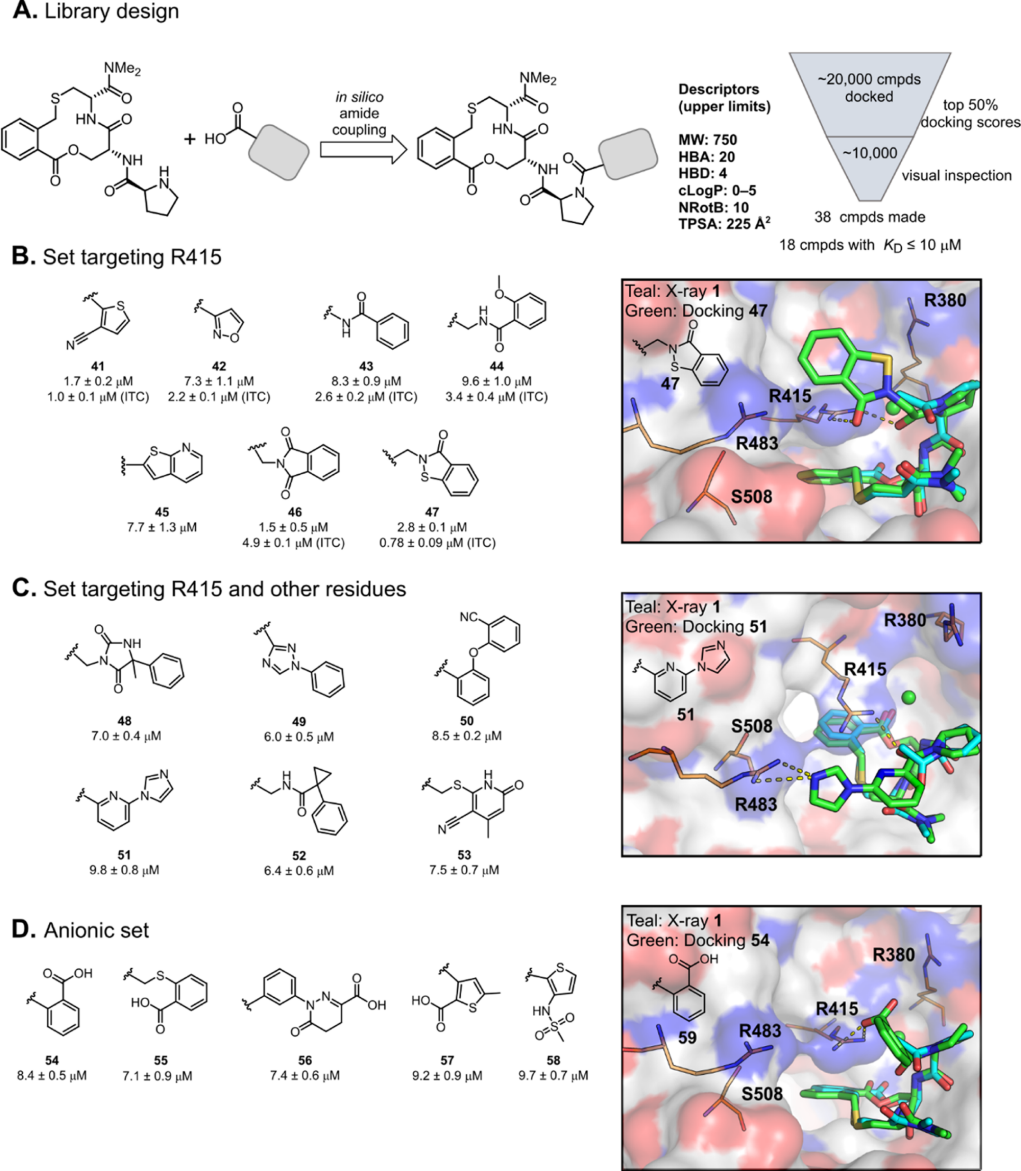
Design and evaluation of three sets of compounds targeting one or two of R380, R415, and R483 in the binding site of Keap1. (A) Schematic description of the design of the library of amides, including the cutoff values used to remove less drug-like compounds and a summary of the number of compounds docked, visually inspected, and synthesized. Structures and dissociation constants of (B) compounds **41–47**, designed to interact mainly with R415, (C) compounds **48–53**, designed to interact also with arginines other than R415, and (D) compounds **54–58**, designed to interact with one or two of the arginine residues *via* a salt bridge. Dissociation constants were obtained by SPR using an ISA and also by ITC for selected compounds. Mean values ± standard deviation derived from a minimum of three experiments are reported. The inserted figures in (panels B–D) show the predicted binding modes of compounds **47**, **51**, and **54** in the Keap1 binding site superimposed on the structure of **1** in the complex with Keap1 (PDB ID: 6Z6A). Compound **1** is shown in teal, and compounds **47**, **51**, and **54** are in green. Keap1 is shown as a white surface with oxygen atoms in red and nitrogen atoms in blue in the inserted figures. Selected residues in Keap1 are shown as orange sticks.

Compounds targeting R415 and/or one of R380 and R483 in the binding site of Keap1 were selected from docking screens of diverse libraries of amides formed at the N-terminus of the proline residue of **1** (Figure 9A). In this workflow, suitable and readily available building blocks were first identified from chemical vendors, and then a virtual library of amides was generated. The virtual library was further filtered by confining molecular descriptors of its products (*i.e.*, MW, HBA, HBD, cLogP, NRotB, and TPSA) into a more drug-like chemical subspace (Figure 9A). A PAINS filter was applied to reduce the risk of obtaining promiscuous inhibitors and false positives, for example, compounds that would inhibit the formation of the Keap1–Nrf2 PPI by reacting with the cysteine sulfhydryl groups of Keap1.^29^ Approximately 20,000 compounds met the selection criteria and were docked into the crystal structure of the complex of **1** and Keap1 (PDB ID: 6Z6A).^9^ The top-scoring compounds were visually inspected, and the compounds that maintained the binding mode of the macrocyclic core and engaged in an interaction with at least one of the three arginine residues were selected. Of these, 38 compounds that can be separated into three distinct subsets targeting a specific residue or type of interaction were subsequently synthesized (*cf.*Figure 9B–D for compounds with *K*_D_ <10 μM in the ISA). A first set of uncharged compounds was designed to target mainly R415 through a hydrogen bond or a π-cation interaction (Figure 9B). The second set of compounds formed the same type of interactions also with one of the other two arginine residues found in the Keap1 binding site (Figure 9C). Finally, the third set of compounds contained an acidic group to allow the formation of a salt bridge to one or two of the arginines in the binding site (Figure 9D). The structures and inhibitory potencies for compounds with *K*_D_ ≥10 μM (Supporting Information Compounds **S6–S24**), as well as the predicted binding modes of compounds **41–58**, are presented in the Supporting Information (Scheme S1 and Figures S2 and S3).

Evaluation in the ISA revealed that 7 of the 17 compounds in the set designed to target R415 had *K*_D_ values ≤10 μM (Figure 9B). As determined by ITC, the nitrile substituted thiophene **41** and the heterocycle **47** both provided a 3–4-fold improvement in potency in comparison to inhibitor **1**. Isoxazole **42**, *N*-acylurea **43**, and glycine derivative **44** all displayed potencies that ranged from 2-fold lower to comparable to that of compound **1** in the ITC measurement, while phthalimide **46** was slightly less potent. Six of the 11 compounds in the set targeting R415 and one of the other two arginines possessed a *K*_D_ ≤10 μM in the ISA (Figure 9C). Unfortunately, no compound from this set displayed any improvement in potency relative to **1** and they were therefore not studied further. The anionic set consisted of 10 compounds which contained either a carboxylic acid or an arylmethanesulfonamide (Figure 9D). However, neither the carboxylic acids nor the sulfonamides showed any increased potency as compared to **1**. The most potent carboxylic acids, **54–57**, and sulfonamide **58** displayed approximately a 2–3-fold loss of potency in the ISA. As no major gain in potency was obtained for any of the inhibitors acylated at proline, further efforts to optimize **1** were directed to the phenylene moiety.

### Optimization Using Libraries Grown from the Phenylene Moiety

**Figure 10:**
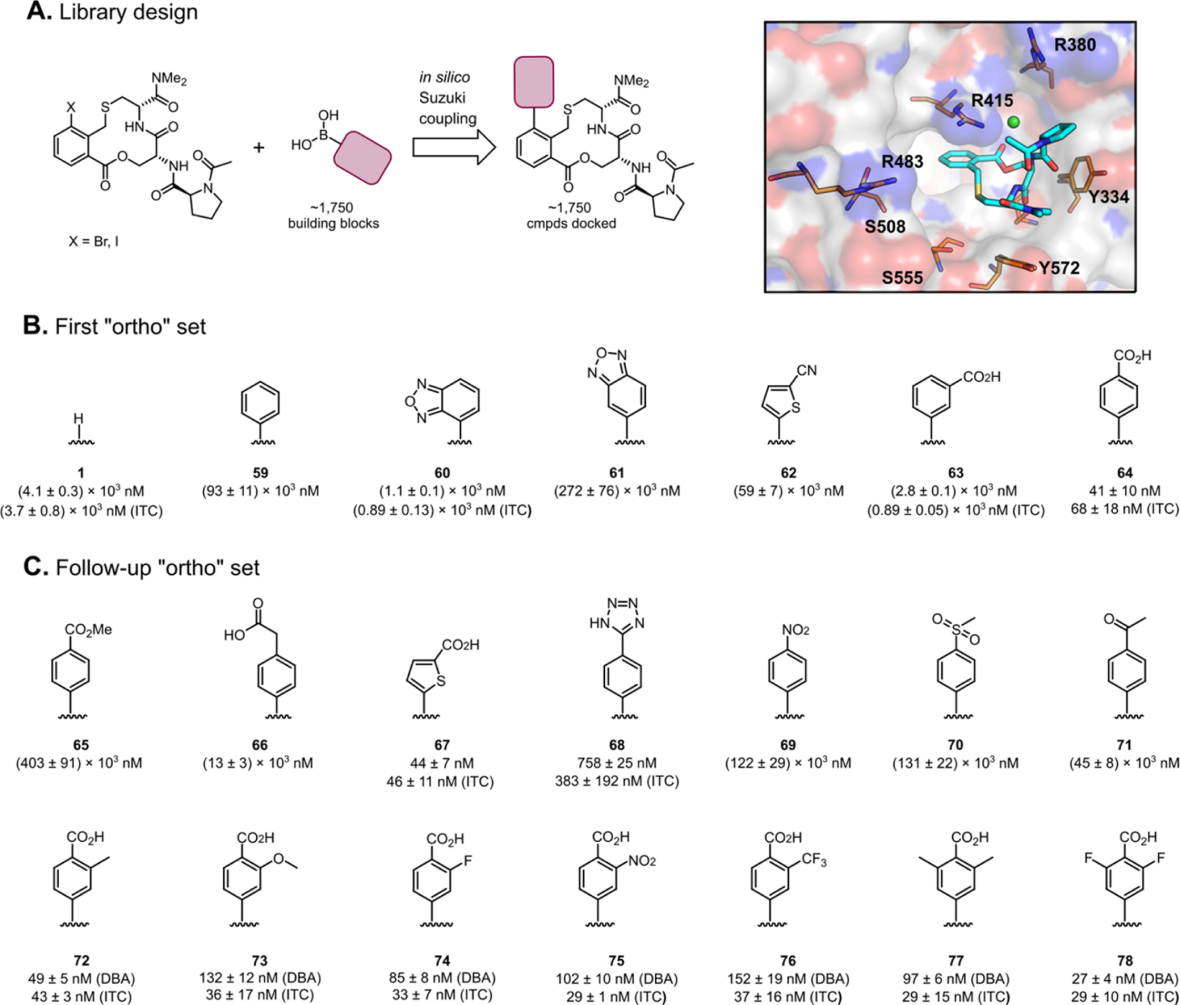
Design and evaluation of two sets of compounds grown from the phenylene ring of compound **1**. (A) Schematic description of the design of a virtual library based on the Suzuki coupling. The Keap1-bound crystal structure of compound **1** (PDB ID: 6Z6A) illustrates the opportunity of growing toward R415, R483, and S508 by substituting the position *ortho* to the thiomethylene group of **1**. (B) Structures and dissociation constants of compounds **59–64**; compound **1** is shown for comparison. (C) Structures and dissociation constants of compounds **65–78**. Dissociation constants were obtained by SPR using an ISA, unless otherwise stated. A DBA and ITC was used for selected compounds. Mean values ± standard deviation derived from a minimum of three experiments are reported.

The initial SAR exploration at the position *ortho* to the thioether of the phenylene moiety inspired us to construct a virtual library at this position (Figure 10). Substituents at the *ortho*-position are located deeper in the Keap1 binding pocket than those attached at the N-terminus of proline, while still having the possibility of targeting R415 and R483. Analogous to the virtual amide library, suitable and readily available building blocks bearing functionalities compatible with a Suzuki coupling were extracted from chemical vendor catalogs and enumerated *in silico* to afford biarylic compounds. The library was then filtered to retain compounds with molecular descriptors within the drug-like range used for the amide library and to remove PAINS chemotypes. The remaining compounds in the library (∼1750) were docked into Keap1 and those maintaining the binding mode of the macrocyclic core and that interacting with at least one of the polar residues lining the part of the Keap1 pocket facing the *ortho* position of the ligand (R483, S508, and R415, *cf.* inset at top right of Figure 10A) were further processed for visual inspection. A set of six compounds were first selected and synthesized (**59–64**, Figure 10B); then, the most potent compound of this set (**64**) was followed up by a set of 14 compounds (**65–78**, Figure 10C).

The first six compounds of the “*ortho* set” contained four neutral and two ionic compounds that displayed a pronounced SAR (Figure 10). Thus, phenyl derivative **59** showed a 50-fold loss of potency as compared to **1**. Oxadiazole **60** instead provided a 5-fold improvement in the binding activity, as determined by ITC, while the regioisomeric oxadiazole **61** was close to 300-fold less potent than **60**. The nitrile-substituted thiophene **62** provided a 15-fold loss in potency as compared to **1**. The *m*- and *p*-benzoic acids **63** and **64** were included in the set to target R483 and R415 *via* salt bridges, and *m*-benzoic acid **63** had a potency comparable to compound **1**. Interestingly, the *p*-benzoic acid regioisomer **64** provided almost a 100-fold improvement in the ISA. As determined by ITC, compound **64** had *K*_D_ = 68 nM, making it the first compound of the series to show a potency in the double-digit nanomolar range.

The second of the two “*ortho* sets” was prepared to probe the SAR of *p*-benzoic acid **64**, first using a few analogues and isosteres (**65–71**) and then by incorporation of small substituents in one or both of the *ortho* positions of the benzoic acid moiety (**72–78**). The importance of the benzoic acid moiety of **64** was revealed by methyl ester **65**, which showed a 10,000-fold decrease in potency as compared to **64**. Homologation of **64** to compound **66** reduced the potency approximately 200-fold, illustrating the importance of a correct positioning of the carboxylic acid in the Keap1 binding site. However, incorporation of a thiophene carboxylic acid (compound **67**) instead of the *p*-benzoic acid of **64** was tolerated, most likely as it positioned the carboxylic acid in the correct location. Replacement of the carboxylic acid with the bioisosteric tetrazole to give compound **68** led approximately to a 5-fold loss of potency (ITC). Neutral compounds **69–71**, which contain one or two negatively polarized oxygen atoms that could act as replacements for the carboxylic acid, proved to be at least 1000 times less potent than **64**. Substitution at the position *ortho* to the carboxylic acid of **64** was investigated by the preparation of compounds **72**–**76**, bearing a methyl, methoxy, fluoro, nitro, or trifluoromethyl group, respectively. All these compounds showed a slight increase of potency relative to **64**, with the potencies ranging from 29 to 43 nM (ITC). The bis-methyl and bis-fluoro derivatives **77** and **78** were the most potent and both had 29 nM *K*_D_ values (ITC).

### Characterization of Compounds **64**, **77**, and **78**

Compounds **64**, **77**, and **78** were 50–100 times more potent than compound **1**(9) (Table 1). These three compounds all have a low lipophilicity (LogD_7.4_ <−0.8) and high aqueous solubility (>800 μM), reflecting that they contain a carboxylic acid. Their permeability across a Caco-2 cell monolayer is low (0.16–0.19 × 10^–6^ cm/s) just as their efflux ratio (approximately 1), the latter of which constitutes an improvement over compound **1** (ER approximately 50). As expected from their low lipophilicity compounds, **64**, **77**, and **78** display low *in vitro* metabolism when incubated with human liver microsomes. The compounds were also stable in rat plasma (data not shown). In contrast, the more lipophilic and uncharged **1** has a higher *in vitro* metabolism. Macrocycles **64** and **77** displayed cellular efficacies similar to that of **1**(9) as determined by their ability to induce Nrf2 translocation into the nucleus by inhibiting the formation of the Keap1–Nrf2 complex. The low cellular efficacies of **64** and **77** most likely result from their low cell permeabilities. We speculate that the overlapping cellular efficacies of **1** and **77**, which differ 100-fold in affinity for Keap1, at least in part originate from the higher passive permeability of **1**(9) (1.6 × 10^–6^ cm/s) as compared to that of **77** (0.19 × 10^–6^ cm/s).

**Table 1:** *In Vitro* Characterization of Compounds **64**, **77**, and **78**, in Comparison to **1**(9)^a^

	**64**	**77**	**78**	**1**
*K*_D_, ITC (nM)	68 ± 18	29 ± 15	29 ± 10	3700 ± 800
LogD_7.4_	<−1.5	<−1.4	<−0.8	0.43 ± 0.05
solubility^b^ (μM)	>850	965 ± 38	>1000	805 ± 106
*P*_app_ AB^c^ (×10^–6^ cm/s)	0.16 ± 0.01	0.19 ± 0.12	0.18 ± 0.10	0.19 ± 0.07
ER^d^	1.19 ± 0,15	0.84 ± 0.23	0.84 ± 0.26	52.2 ± 28.0
Cl_int_, human mics. (μL/min/mg)	<3.0	<3.0	<3.0	36.5 ± 2.5
Nrf2 translocation^e^ (% induction)	16 ± 2	38 ± 0.4		37 ± 4

a The values for *K*_D_, LogD_7.4_, solubility, cell permeability, efflux ratio, and the clearance in human liver microsomes and rat hepatocytes are mean values ±standard deviation from ≥three replicates. The induction of Nrf2 translocation into the nucleus is the mean from two measurements on two distinct samples.

b Solubility in phosphate-buffered saline at 25 °C and pH 7.4.

c Permeability across a Caco-2 cell monolayer in the apical-to-basolateral direction.

d ER = efflux ratio (BA/AB) for permeability across a Caco-2 cell monolayer.

e Induction of Nrf2 translocation into the nucleus at 256 μM.

Compounds **64**, **77**, and **78** were also screened in a secondary pharmacology panel (CEREP) consisting of 88 distinct GPCRs, ion channels, enzymes, nuclear hormone receptors, and transporters, most of which are of human origin (Table S6). The three compounds were inactive at the vast majority of the targets, that is, they did not elicit a response at the upper detection limit of the assays (IC_50_ or EC_50_ 100 μM for most targets). However, compounds **77** and **78** showed weak activity (IC_50_ 20–70 μM) at five and one of the targets, respectively. In conclusion, compounds **64**, **77**, and **78** showed high selectivity for Keap1 over a panel of 88 potential off-targets.

### Keap1-Bound Crystal Structures of Compounds **60**, **63**, and **64**

**Figure 11:**
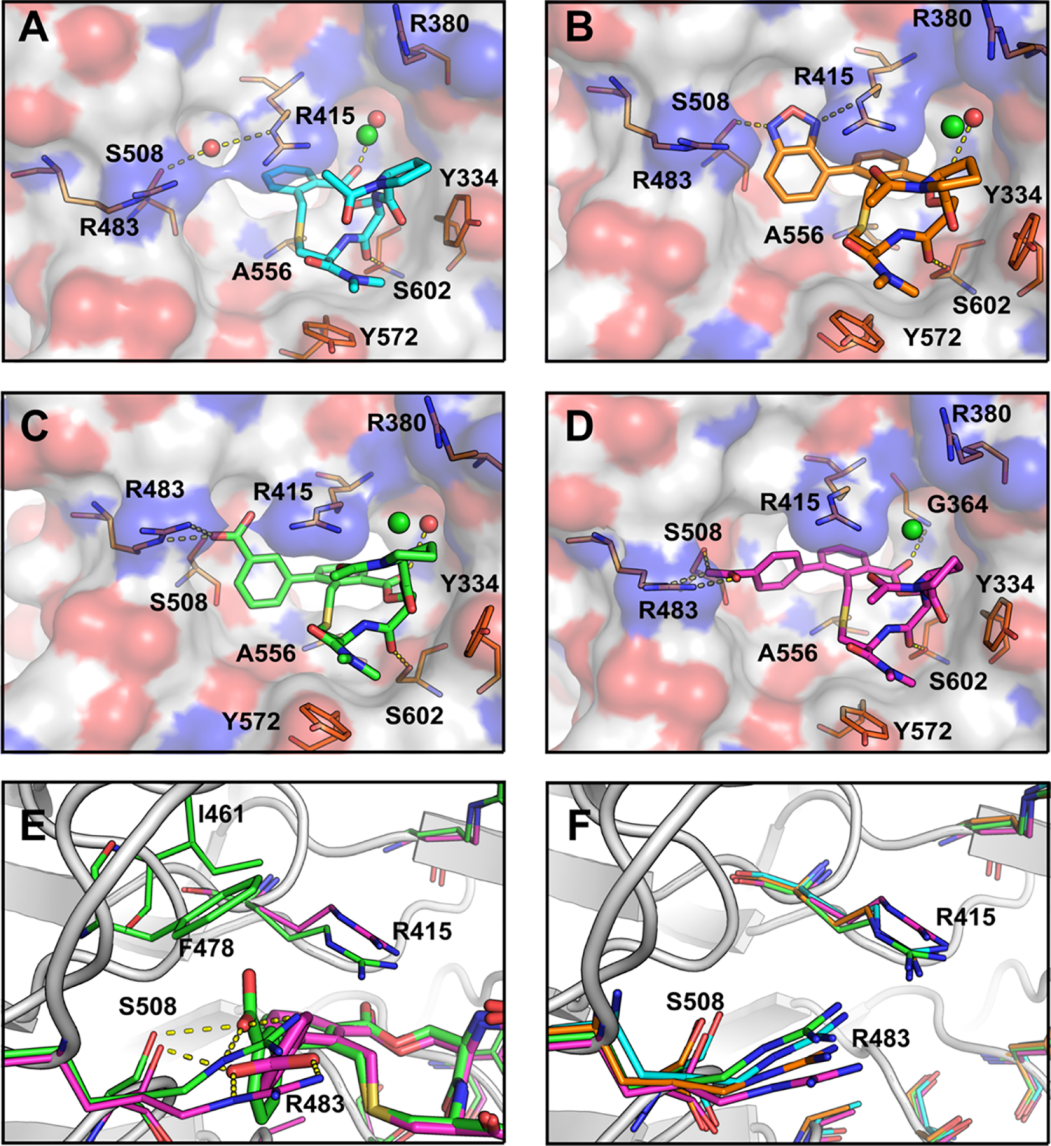
Crystal structures of the complexes of Keap1 with compounds (A) **1** (PDB ID: 6Z6A),^9^ (B) **60** (PDB ID: 7Q6S), (C) **63** (PDB ID: 7Q8R), and (D) **64** (PDB ID: 7Q96). Compound **1** is shown as teal sticks, while compounds **60**, **63**, and **64** are shown as orange, green, and magenta sticks, respectively. Keap1 is shown as a white surface with the oxygen atoms in red and nitrogen atoms in blue, selected Keap1 residues are shown as orange sticks, polar contacts are shown as yellow dashed lines, a chloride ion is shown as a green sphere, and bound water molecules are shown as red spheres. (E) Overlap of compounds **63** (green) and **64** (magenta) providing a close-up view of their polar contacts (yellow dashed lines) with R483 and S508. Keap1 (from PDB ID: 7Q96) is shown as a gray cartoon, with selected residues in the appropriate complex shown as sticks matching the color of the corresponding ligand. (F) Close-up view showing the orientations of residues R415, R483, and S508 of Keap1 in the complexes with compounds **1**, **60**, **63**, and **64**. The ligands have been removed for clarity, but the side chains of the three residues are colored as for each ligand in (panels A–D), *i.e.*, teal (**1**), orange (**60**), green (**63**), and magenta (**64**). Keap1 (from PDB ID: 6Z6A) is shown as a gray cartoon.

The Keap1-bound structures of compounds **60**, **63**, and **64** were determined by X-ray crystallography at 2.2, 2.3, and 2.4 Å resolution, respectively (Figure 11). Inspection of these structures, and that of **1** bound by Keap1, emphasized the common features of how Keap1 binds this series of inhibitors and allowed the rationalization of the large differences in the binding affinity displayed by them. In all four structures, the macrocyclic ring of the four ligands adopts an almost identical conformation (Figure 11A–D). This positions the phenylene moiety between R415 and A556, stacks the C-terminal dimethyl amide against Y572, and orients the carbonyl group of the serine moiety so that it forms a hydrogen bond with the side-chain hydroxyl group of S602 in Keap1. A chloride ion bridging the serine NH proton with several residues on Keap1 is also common to all the crystal structures. The lactone carbonyl oxygen of **60** and **63** is involved in a hydrogen bond with a water molecule just as for **1**, while the same carbonyl group in compound **64** is hydrogen-bonded to G364.

The substituents at the *ortho* position of the phenylene ring of **60**, **63**, and **64** form different interactions with Keap1, with *para*-acid **64** forming the largest number of additional polar interactions as compared to the complex of Keap1 with **1**. The nitrogen atoms of the oxadiazole of **60** are involved in hydrogen bonds with the side chains of S508 and R415 but form no interaction with R483 (Figure 11B). The *meta* carboxylic acid group of compound **63** forms a salt bridge with the side chain of R483 (Figure 11C), just as the *para* carboxylic acid of **64** which also forms a hydrogen bond with S508 (Figure 11D). Both oxygen atoms of the carboxylate group of compound **64** interact with the guanidine side chain of R483 *via* a bidentate and coplanar interaction (Figure 11E). Such a “side-on” geometry has been found to be the preferred orientation in salt bridges between arginine and the side chains of aspartic and glutamic acids in proteins.^30^ In contrast, only one oxygen atom of the carboxyl group of **63** interacts with R483 in a less preferred “back-side” geometry (Figure 11E).^30^ Moreover, the other oxygen atom points toward a hydrophobic pocket formed by the side chains of I461, F478, and the aliphatic chain of R415. These structural differences, together with the additional hydrogen bond to S508 formed by **64**, are most likely important for the >50-fold higher affinity for Keap1 displayed by **64** over **63**. Even though the oxadiazole of **60** forms hydrogen bonds with S508 and R415, its inability to make a salt bridge with arginine most likely explains the lower potency as compared to **64**.

Overlap of the Keap1-bound structures of compounds **1**, **60**, **63**, and **64** reveals how seemingly small reorientations of R415 and R483 appear to have a major impact on the affinity by which Keap1 can recognize different ligands (Figures 11F and S4). The orientation of R483 is almost identical in the complexes with compounds **1** and **63**, whereas it has undergone a significant adjustment in the complex with **64**. In the complex with **60**, R483 occupies a position intermediate between that in the complexes with compounds **1** and **64**. The orientation of R415 is nearly identical in the complexes with compounds **1**, **60**, and **63**, while **64** again shows a pronounced difference. Accounting for the flexibility of the side chains of these two arginine residues, for example, by MD simulations of ligand–Keap1 complexes, thus appears to be essential for the precise design of high-affinity ligands for Keap1.

### Thermodynamics of Ligand Binding to Keap1

**Figure 12:**
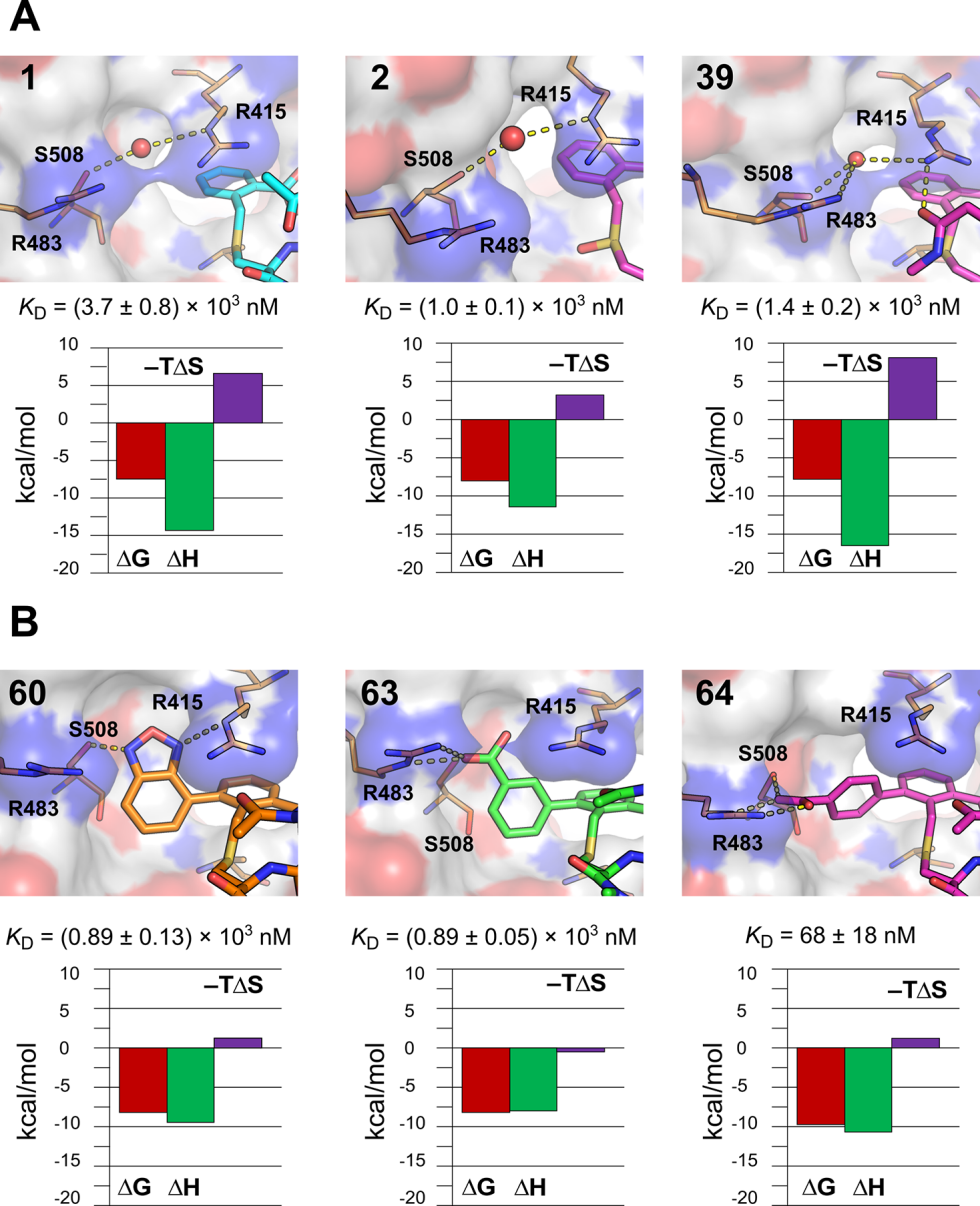
Close-up views of the crystal structures of Keap1 with (A) compounds **1**, **2**, and **39** and (B) compounds **60**, **63**, and **64**. Dissociation constants and thermodynamic profiles determined by ITC for the formation of each complex are shown below the structures. A bound water molecule that bridges S508 and R415 in the complexes with **1**, **2**, and **39** is shown as a red sphere. Keap1 is shown as a white surface with oxygen atoms in red and nitrogen atoms in blue, selected Keap1 residues are shown as orange sticks, and polar contacts are shown as yellow dashed lines.

A water molecule bridges S508 and R415 in each of the complexes of compounds **1**, **2**, and **39** with Keap1 (Figure 12A). Interestingly, this water molecule has been displaced by the substituents at the *ortho*-position of the macrocyclic phenylene moiety of compounds **60**, **63**, and **64** (Figure 12B). Displacement of this bound water by a carboxyl group has previously been reported to provide major increases in potency for other lead series.^11,15^ It is therefore reasonable to assume that this displacement also contributes to the 50–100-fold potency improvement of **64** over **1**, in addition to the ability of R415 and R483 to reorient so that R483 forms an optimal bidentate interaction with **64**.

The binding of compounds **1**, **2**, and **39** to Keap1 is enthalpy-driven under the experimental conditions (*T* = 25 °C) and displays unfavorable entropy components, in particular for **1** and **39** (Figure 12A and Table S7). In contrast, the observed entropy contribution is considerably less unfavorable for compounds **60** and **64**, and even somewhat favorable for **63**, while the observed enthalpic component is reduced as compared to **1**, **2**, and **39** (Figure 12B and Table S7). Thermodynamic data is influenced by different factors such as solute effects, structural flexibility, and cooperativity, making its interpretation in terms of intermolecular interactions and solvation difficult.^31^ However, it appears reasonable to propose that the more favorable entropy term displayed by compounds **60**, **63**, and **64**, as compared to **1**, **2**, and **39**, to a large extent originates from the displacement of the water molecule bridging residues S508 and R415 of Keap1. Compounds **67**, **68**, and **72**–**78**, all of which contain a carboxyl or tetrazole group on the aromatic moiety attached at the *ortho*-position of the phenylene ring, provide additional support for this hypothesis. These all show a thermodynamic profile with a low observed entropy contribution, just as **60**, **63**, and **64** (Table S7). In contrast, compounds **7**, **38**, **41**, **42**, **44**, **46**, and **47**, which lack substituents on the phenylene ring just as **1**, **2**, and **39**, all display potencies and thermodynamic profiles similar to those of **1**, **2**, and **39** (Table S7).

### Synthesis

#### Modification of the Macrocyclic Ring

**Scheme 1:**
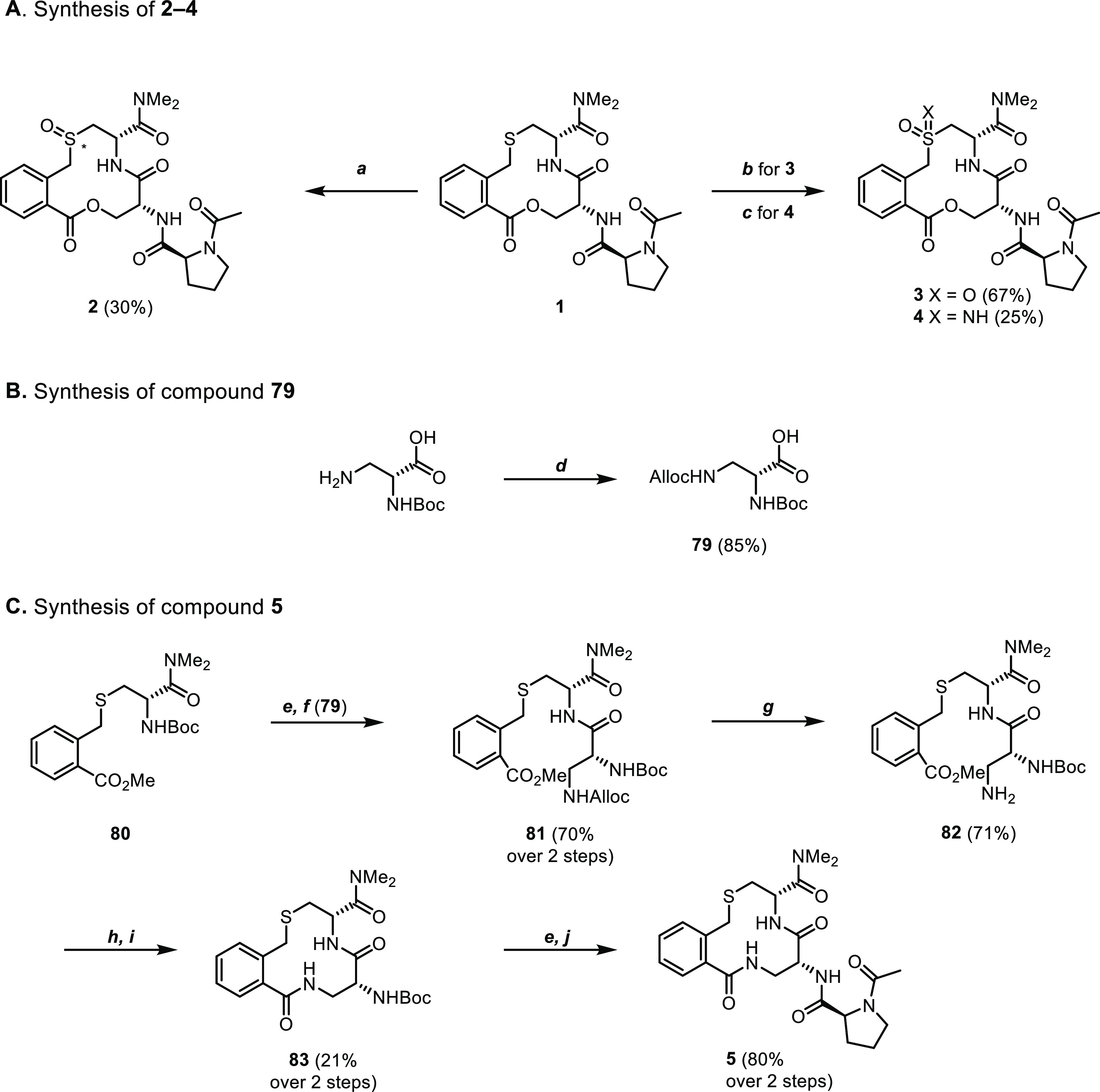
Synthesis of Compounds **2–5** Reagents and conditions: (a) *m*-CPBA (1.0 equiv), DCM, rt, 1 h. (b) *m*-CPBA (3.0 equiv), DCM, rt, 2 h. (c) H_2_NCO_2_NH_4_, PhI(OAc)_2_, MeOH, rt, 2 h. (d) Allyl chloroformate, K_2_CO_3_, 1,4-dioxane/H_2_O 1:1, 16 h, rt. (e) 4 M HCl in 1,4-dioxane, rt, 1 h. (f) **79**, 1-ethyl-3-(3-dimethylaminopropyl)-carbodiimide hydrochloride (EDC)·HCl, MeCN, rt, 1 h. (g) Pd(PPh_3_)_4_, K_2_CO_3_, MeOH, rt, 2 h. (h) LiOH, MeOH/H_2_O 1:2, 40 °C, 16 h. (i) HATU, DIPEA, DMF, rt, 2 h. (j) Ac-l-Pro-OH, EDC·HCl, DIPEA, DMSO, rt, 2 h.

Oxidation of sulfide **1**, either with stoichiometric or excess *m*-CPBA, gave compounds **2** and **3**, respectively (Scheme 1A). Sulfoximine **4** was readily accessible by the application of a chemoselective, one-pot procedure using bisacetoxyiodobenzene.^32^ In order to synthesize macrocyclic lactam **5** (Scheme 1B,C), the Alloc-protected amino acid **79** was first prepared from the commercially available Boc-d-Dap-OH (Scheme 1B). Subsequently, the Boc group of compound **80**(9) was cleaved, followed by coupling with **79**. The Alloc group of amide **81** was cleaved to afford amine **82**, followed by saponification of the methyl ester and lactamization to give macrocycle **83** (21% over two steps). Finally, Boc removal and coupling with Ac-l-Pro-OH gave compound **5**.

**Scheme 2:**
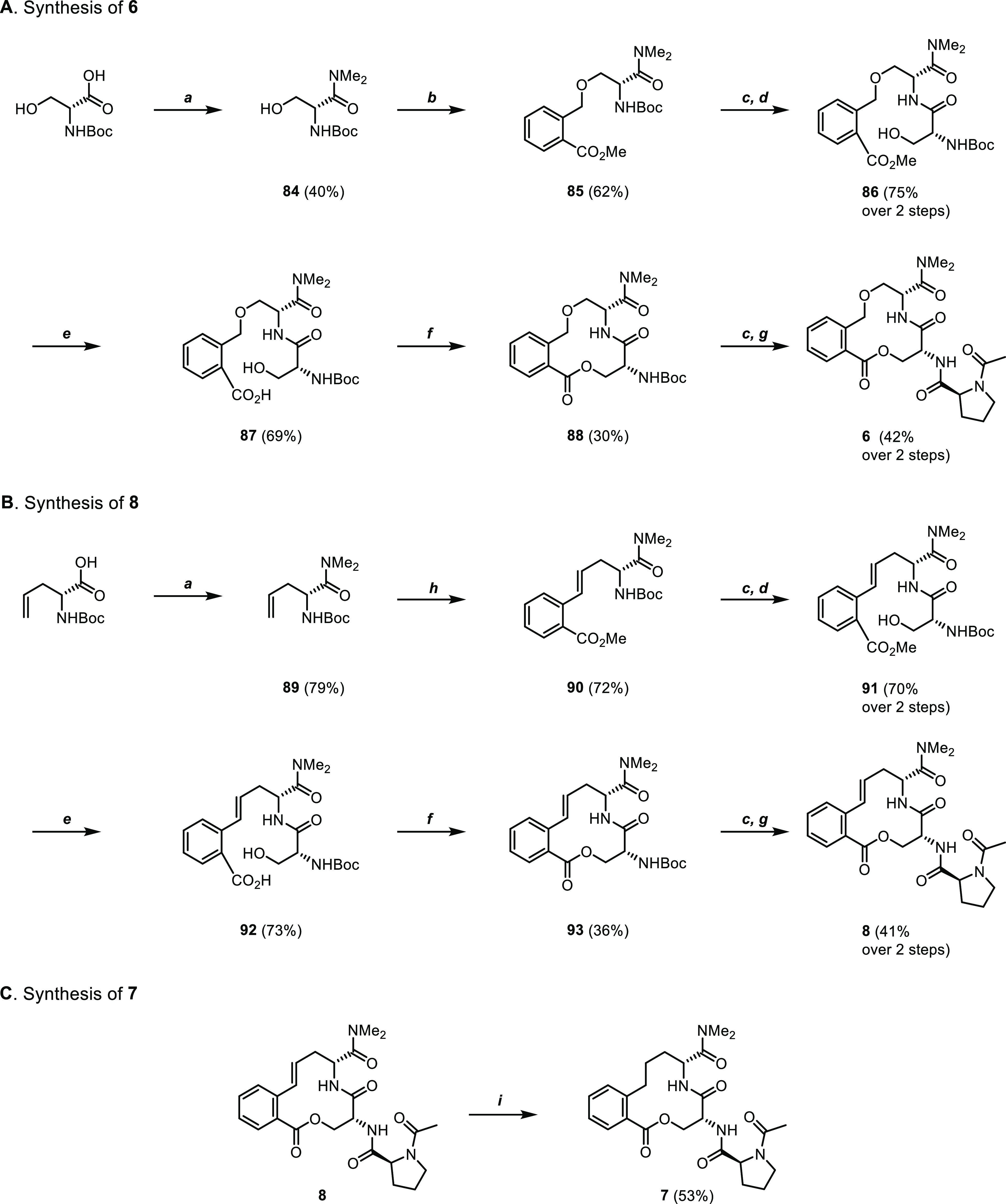
Synthesis of Compounds **6–8** Reagents and conditions: (a) Me_2_NH·HCl, EDC·HCl, HOBt·*x*H_2_O, DMF, rt, 2 h. (b) Methyl 2-(bromomethyl)benzoate, NaH, DMF, 0 °C, 45 min. (c) 4 M HCl in 1,4-dioxane, rt, 1 h. (d) Boc-d-Ser-OH, EDC·HCl, MeCN, rt, 2 h. (e) LiOH, MeOH/H_2_O 1:1, 40 °C, 16 h. (f) PPh_3_, DBAD, THF, rt, 2 h. (g) Ac-l-Pro-OH, EDC·HCl, DIPEA, DMSO, rt, 2 h. (h) Methyl 2-iodobenzoate, Pd(OAc)_2_, DIPEA, MeCN, 85 °C, 16 h. (i) H_2_ (5 bar), Pd/C, MeOH, rt, 16 h.

The synthesis of the oxygen and carbon analogues of **1** and **6–8** required multistep routes (Scheme 2A–C). For the oxygen analogue **6**, Boc-d-Ser-OH was first transformed into the corresponding dimethyl amide **84**, which was then alkylated with methyl 2-(bromomethyl)benzoate in the presence of sodium hydride to obtain ether **85**. The Boc-protecting group was cleaved, and the resulting free amine coupled with Boc-d-Ser-OH. Saponification of the methyl ester of dipeptide **86** afforded acid **87**, which was transformed into macrocycle **88** using Mitsunobu conditions (30%). Removal of the Boc group and coupling with Ac-l-Pro-OH gave compound **6**. The synthesis of carbon analogues **7** and **8** started with transforming Boc-d-allyl-Gly-OH into dimethylamide **89**, which then underwent a Heck reaction with methyl 2-iodobenzoate to afford alkene **90** (Scheme 2B). After the removal of the Boc group, the liberated amine was coupled with Boc-d-Ser-OH to afford dipeptide **91**. Saponification of the methyl ester provided acid **92**, which underwent macrolactonization to give **93** (36%). Finally, Boc cleavage and coupling with Ac-l-Pro-OH gave compound **8**. Compound **7** was obtained by catalytic hydrogenation of the alkene moiety of compound **8** (Scheme 2C).

#### Substitution of the Phenylene Ring

**Scheme 3:**
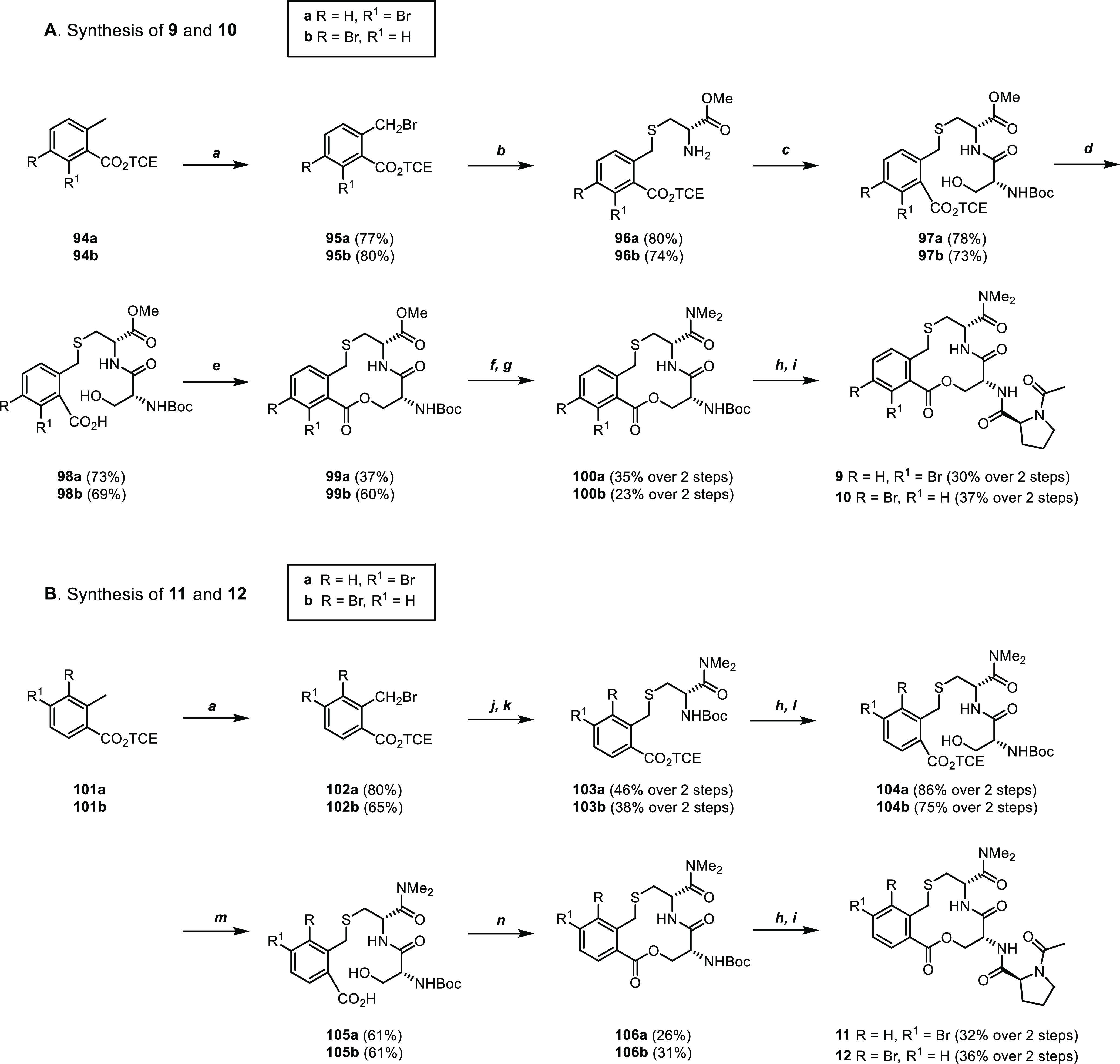
Synthesis of Compounds **9–12** Reagents and conditions: (a) *N*-bromo succinimide, AIBN, chlorobenzene, 70 °C, 16 h. (b) H-d-Cys-OMe, triethylamine, DMSO, rt, 2 h. (c) Boc-d-Ser-OH, EDC·HCl, MeCN, rt, 1 h. (d) Zn, NH_4_OAc, THF/H_2_O 5:1, rt, 2 h. (e) PPh_3_, DBAD, toluene, rt, 4 h. (f) Me_3_SnOH, 1,2-DCE, 83 °C, 45 min. (g) 2 M Me_2_NH in THF, EDC·HCl, HOBt·*x*H_2_O, DMF, rt, 2 h. (h) 4 M HCl in 1,4-dioxane, rt, 1 h. (i) Ac-l-Pro-OH, EDC·HCl, DIPEA, DMSO, rt, 2 h. (j) Boc-d-Cys-OH, triethylamine, DMSO, rt, 2 h. (k) Me_2_NH·HCl, HATU, DIPEA, DMF, rt, 2 h. (l) Boc-d-Ser-OH, EDC·HCl, DIPEA, rt, 1 h. (m) Zn, NH_4_OAc, THF/H_2_O 10:1, rt, 2 h. (n) PPh_3_, DBAD, THF, rt, 4 h.

The four compounds of the bromine scan (**9–12**) were synthesized starting from 2,2,2-trichloroethyl (TCE) benzoic acid esters **94a,b** and **101a,b** (Scheme 3). Radical bromination of compounds **94a,b** afforded benzyl bromides **95a,b** (Scheme 3A). Nucleophilic substitution with the thiol of H-d-Cys-OMe and subsequent amide coupling of intermediates **96a,b** with Boc-d-Ser-OH gave dipeptides **97a,b**. Cleavage of the TCE group followed by macrocyclization of acids **98a,b** using Mitsunobu conditions led to lactones **99a,b** (37 and 60%, respectively, in the macrocyclization step), whereas the use of aqueous alkaline hydroxide solutions that resulted in the partial opening of the macrocycle trimethyltin hydroxide allowed for the selective saponification of the methyl ester, leaving the lactone intact. Coupling of the resulting free carboxylic acids with dimethylamine gave amides **100a,b**. Finally, Boc cleavage and coupling with Ac-l-Pro-OH gave compounds **9** and **10**. To circumvent the ring opening encountered for **99a,b** and the use of toxic Me_3_SnOH and 1,2-DCE for the saponification of the methyl ester, the dimethyl amide moiety was introduced at an earlier stage in the synthesis of compounds **11** and **12** (Scheme 3B). Thus, radical bromination of compounds **101a,b** afforded benzyl bromides **102a,b**, which underwent nucleophilic substitution with the thiol of Boc-d-Cys-OH, followed by coupling with dimethylamine to afford amides **103a,b**. Cleavage of the Boc group and subsequent coupling with Boc-d-Ser-OH gave dipeptides **104a,b**, the TCE ester of which were removed to afford benzoic acids **105a,b**. Macrolactonization using Mitsunobu conditions gave macrocycles **106a,b** (26 and 31% yields, respectively), which were subjected to Boc removal and coupling with Ac-l-Pro-OH to give compounds **11** and **12**.

**Scheme 4:**
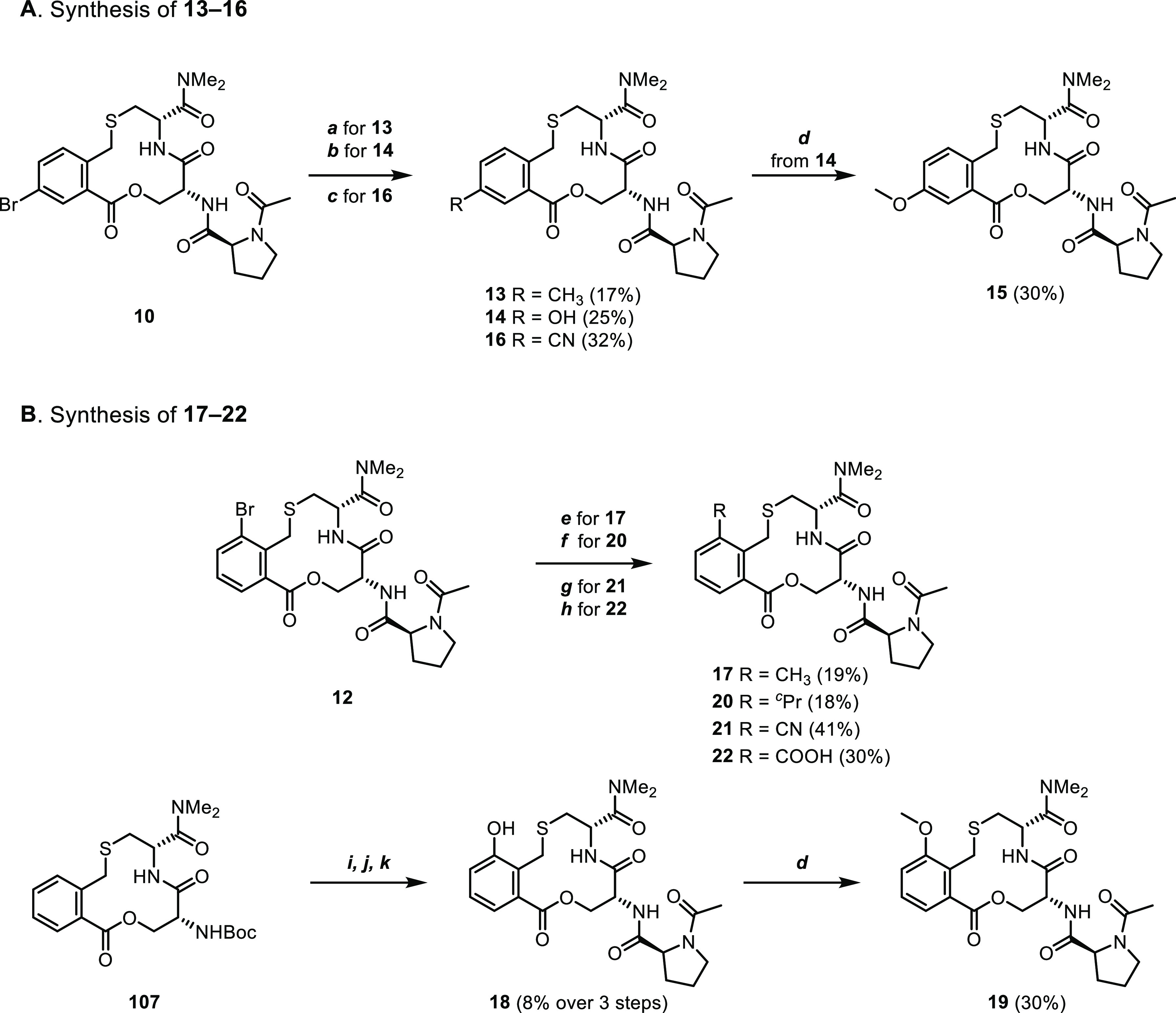
Synthesis of Compounds **13–22** Reagents and conditions: (a) MeBF_3_K, XPhos Pd G3, K_3_PO_4_, toluene/H_2_O 3:1, 135 °C, μW, 2 h. (b) B_2_(pin)_2_, PdCl_2_(dppf), KOAc, 1,4-dioxane, 90 °C, 16 h, then NaBO_3_·4H_2_O, THF/H_2_O 1:1, rt, 3 h. (c) Zn(CN)_2_, ^*t*^BuBrettPhos Pd G3, THF/H_2_O 1:3, 40 °C, 16 h. (d) CH_3_I, K_2_CO_3_, DMSO, 90 °C, 1 h. (e) MeBF_3_K, XPhos Pd G3, K_2_CO_3_, toluene/H_2_O 3:1, 90 °C, 16 h. (f) ^*c*^PrBF_3_K, XPhos Pd G3, K_3_PO_4_, 1,4-dioxane/H_2_O 3:1, 135 °C, μW, 2 h. (g) CuCN, NMP, 180 °C, μW, 20 min. (h) Mo(CO)_6_, Pd(OAc)_2_, DMAP, DIPEA, THF/H_2_O 1:1, 100 °C, μW, 15 min. (i) [Ir(OMe)(1,5-cod)]_2_, B_2_pin_2_, 1,4-dioxane, 95 °C, 16 h. (j) NaBO_3_·4H_2_O, THF/H_2_O 1:1, rt, 1 h. (k) 4 M HCl in 1,4-dioxane, rt, 1 h, then Ac-l-Pro-OH, EDC·HCl, DIPEA, DMSO, rt, 2 h.

The *para*-substituted compounds **13–16** were readily accessible from bromide **10** (Scheme 4A). However, to access gram amounts of building block **10**, the synthetic sequence described above was first optimized (Scheme S3 and Procedure S4). Methylated compound **13** was accessed from **10***via* a Suzuki cross-coupling using methyl trifluoroborate, while the hydroxyl group of **14** was introduced using a palladium-catalyzed borylation, followed by oxidation of the boron species. Phenol **14** was then treated with methyl iodide to give methyl ether **15**. Nitrile **16** was synthesized from **10** by a palladium-catalyzed cyanation reaction.

**Scheme 5:**
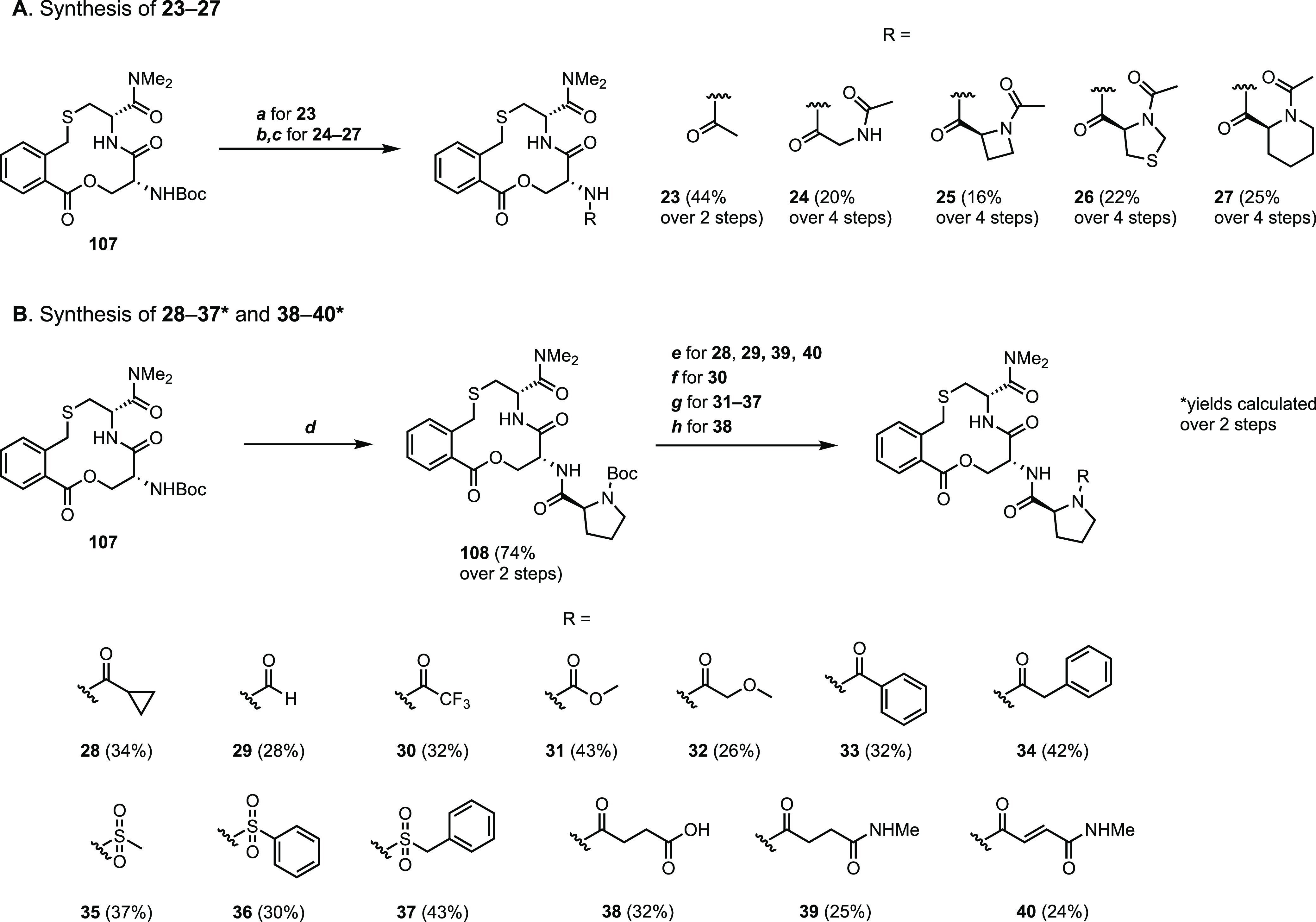
Synthesis of Compounds **23–40** Reagents and conditions: (a) 4 M HCl in 1,4-dioxane, rt, 1 h, then Ac-Cl, triethylamine, DCM, rt, 2 h. (b) 4 M HCl in 1,4-dioxane, rt, 1 h, then corresponding Boc-protected amino acid, EDC·HCl, DIPEA, MeCN, rt, 1 h. (c) 4 M HCl in 1,4-dioxane, rt, 1 h, then Ac-Cl, pyridine or triethylamine, DCM, rt, 2 h. (d) 4 M HCl in 1,4-dioxane, rt, 1 h, then Boc-l-Pro-OH, HATU, DIPEA, MeCN, rt, 2 h. (e) 4 M HCl in 1,4-dioxane, rt, 1 h, then R-OH, HATU, DIPEA, DMSO, rt, 1 h. (f) 4 M HCl in 1,4-dioxane, rt, 1 h, then (CF_3_CO)_2_O, triethylamine, DCM, 2 h, rt. (g) 4 M HCl in 1,4-dioxane, rt, 1 h, then R-Cl, triethylamine, DCM, 2 h, rt. (h) 4 M HCl in 1,4-dioxane, rt, 1 h, then succinic anhydride, DMSO, 2 h, 50 °C.

The *ortho*-substituted compounds **17–22** were readily accessible from bromide **12** (Scheme 5B). Just as for **10**, the synthetic sequence to **12** described above was first optimized to provide gram amounts of **12** (Scheme S4 and Procedure S5). The methyl group and the cyclopropyl group of compounds **17** and **20** were introduced *via* a Suzuki reaction using the corresponding alkyl trifluoroborates. Whereas the conditions used for the synthesis of the *para*-nitrile **16** only gave poor conversion to nitrile **21**, the desired compound could be obtained in 41% yield using copper catalysis. Benzoic acid **22** was prepared by a palladium-catalyzed carbonylation reaction using Mo(CO)_6_ as the CO source. Using a similar strategy for the synthesis of phenol **18** as for phenol **14** was unsuccessful. However, Boc-protected intermediate **107**(9) was successfully subjected to an iridium-catalyzed C–H borylation, followed by oxidation of the obtained boron species, which after Boc cleavage and coupling with Ac-l-Pro-OH gave phenol **18**. Treatment of the hydroxyl group of **18** with methyl iodide gave compound **19**.

#### Replacement of Proline or Its *N*-Acetyl Group

Compounds **23–40** were obtained from the common precursor **107** (Schemes 5A,B), the milligram-scale synthesis of which has been reported previously.^9^ In order to allow the synthesis of several analogues of compound **1** using **107** as the building block, this synthetic route was modified to provide gram quantities of **107** over six steps (16% overall yield) from the reported compound **80**(9) (Scheme S2 and Procedure S3). Then, cleavage of the Boc group of macrocycle **107** followed by acetylation of the free amine provided acetamide **23** (Scheme 3A). Compounds **24–27** were prepared from **107** by a sequence of Boc deprotection, amide coupling with the corresponding Boc-protected amino acid, cleavage of the thereby introduced Boc group, and acetylation of the resulting free amine.

In order to access compounds **28–40** bearing different substituents at the amino group of the proline moiety, the Boc group of compound **107** was cleaved, followed by coupling with Boc-l-Pro-OH to give **108** (Scheme 5B). Boc deprotection of **108** followed by coupling of the free amine with cyclopropanecarboxylic acid or formic acid afforded compounds **28** and **29**, respectively. Treatment of the liberated amine of **108** with trifluoroacetic anhydride provided compound **30**, while reaction with a series of acyl and sulfonyl chlorides provided the corresponding amides **31–34** and sulfonamides **35–37**. Reaction between the deprotected amine of **108** and succinic anhydride provided compound **38**, while compounds **39** and **40** were prepared by amide coupling of the free amine of **108** with the respective carboxylic acid (Scheme 5B).

#### Libraries Grown from Proline

**Scheme 6:**
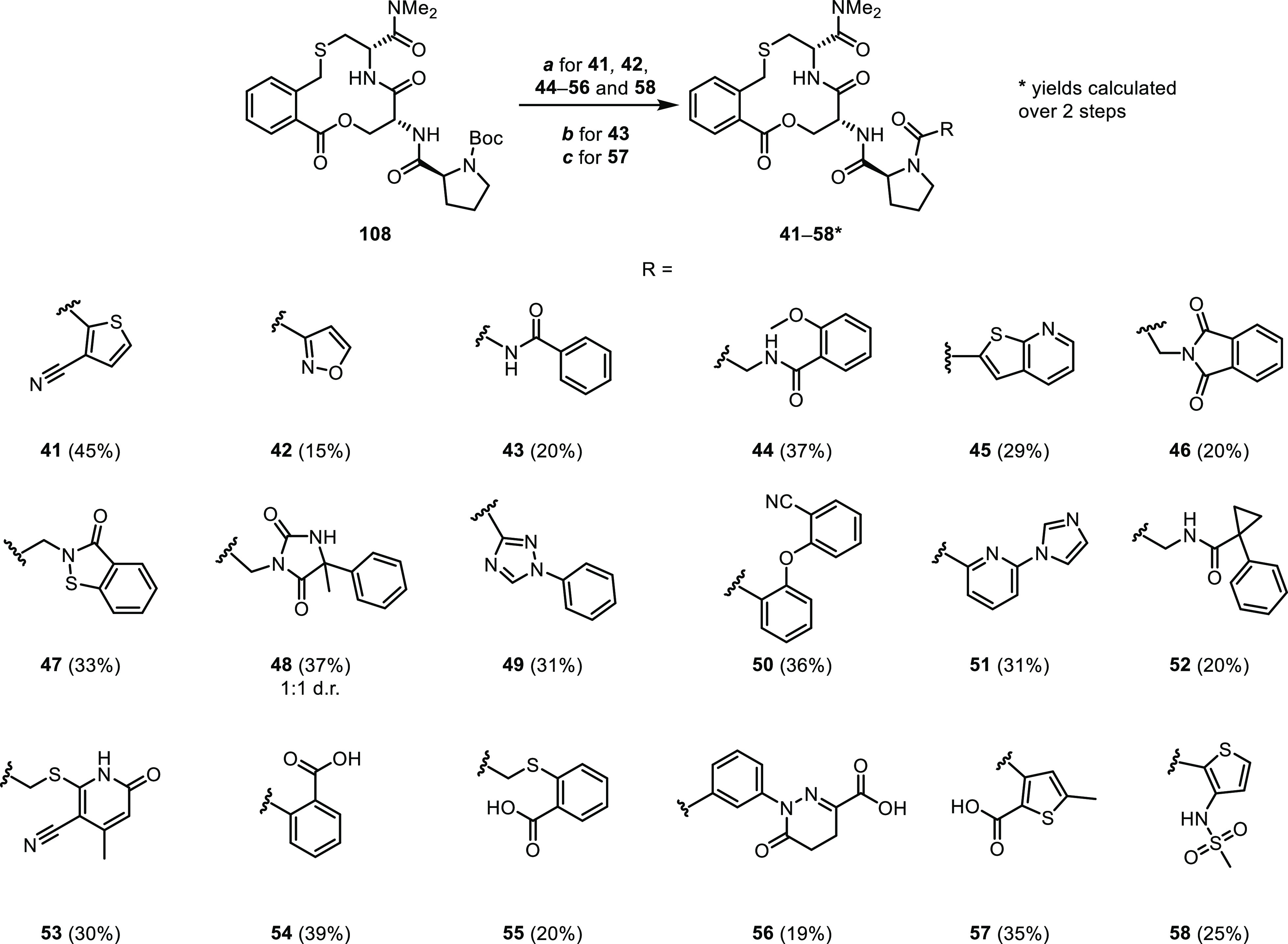
Synthesis of Compounds **41–58** Reagents and conditions: (a) 4 M HCl in 1,4-dioxane, rt, 1 h, then R-COOH, HATU or EDC·HCl, DIPEA, DMSO, rt, 2 h. (b) 4 M HCl in 1,4-dioxane, rt, 1 h, then benzoyl isocyanate, DIPEA, MeCN, rt, 4 h. (c) 4 M HCl in 1,4-dioxane, rt, 1 h, then 5-methylthiophene-2,3-dicarbonyl dichloride, triethylamine, DCM, rt, 16 h.

Compounds **41–58** were synthesized from the common precursor **108** in 15–45% yields (Scheme 6). After cleavage of the Boc group, the liberated amine was coupled with the corresponding carboxylic acids using either EDC·HCl or HATU to give compounds **41**, **42**, **44–56**, and **58**. In order to synthesize compound **57**, the corresponding acid had to be activated using SOCl_2_. For the synthesis of the urea derivative **43**, the liberated amine obtained after Boc deprotection of compound **108** was reacted with benzoyl isocyanate to give the desired compound.

#### Libraries Grown from the Phenylene Moiety

**Scheme 7:**
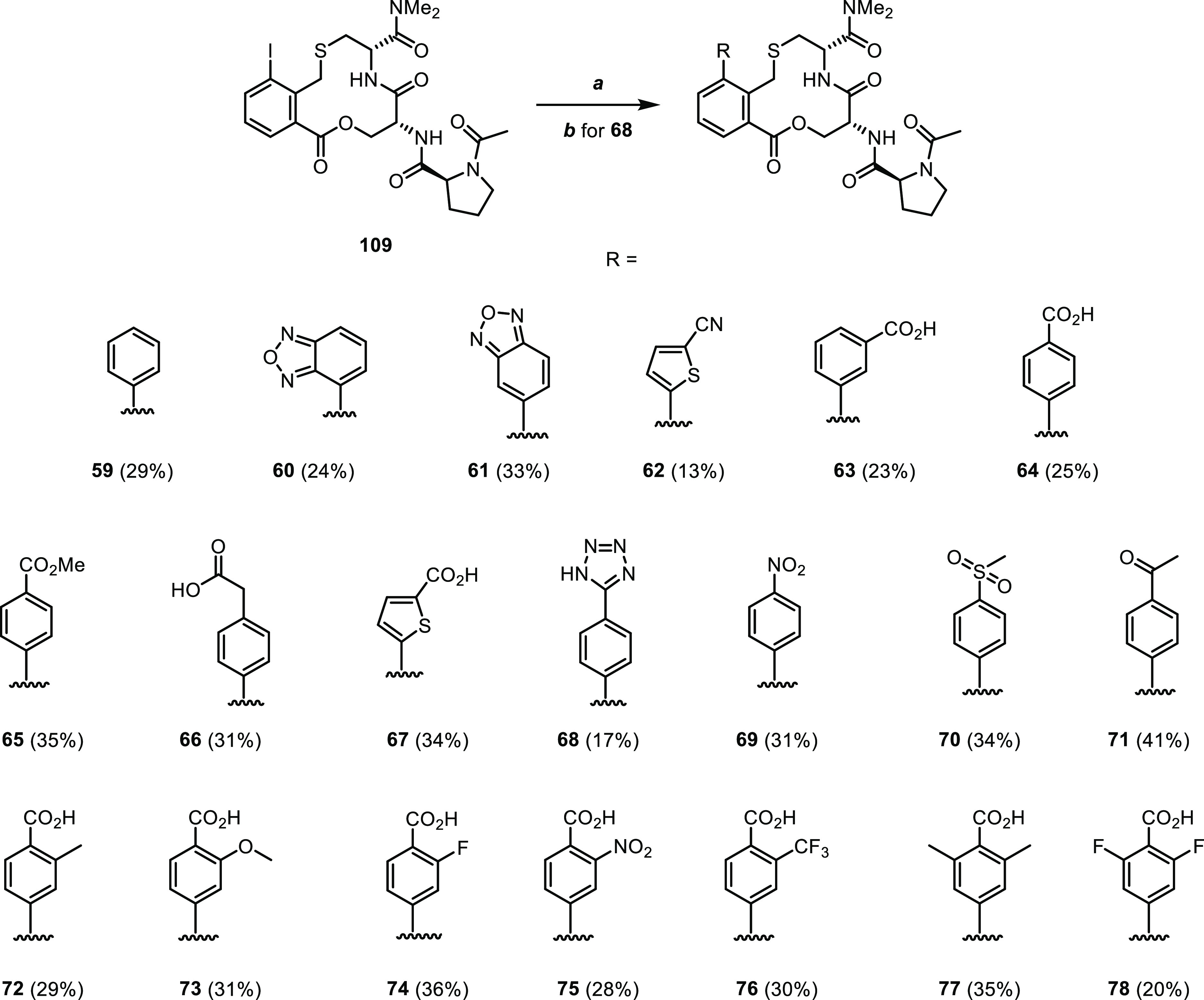
Synthesis of Compounds **59–78** Reagents and conditions: (a) R-B(OH)_2_ or R-Bpin, XPhos Pd G3, K_3_PO_4_, THF/H_2_O 3:1, 65 °C, 4 h. (b) R-B(OH)_2_, XPhos Pd G3, K_3_PO_4_, 1,4-dioxane/H_2_O 3:1, 120 °C, μW, 90 min.

Biaryls **59–78** were synthesized from the common precursor **109** (Scheme 7). A synthetic route, similar to that providing **107**, was developed that provided gram quantities of **109** (eight steps, 12% overall yield from commercially available starting materials; Scheme S5 and Procedure S6). Compounds **59–78** were then synthesized using a Suzuki cross-coupling with a third-generation palladium precatalyst^33^ and the corresponding arylboronic acids or arylboronate esters. Whereas the Suzuki reaction to give compounds **59–67** and **69–78** proceeded at 65 °C, the synthesis of tetrazole **68** required microwave irradiation and a higher temperature to give a satisfactory conversion.

## Summary and Conclusions

In this study, we have improved the potency of a 4 μM macrocyclic natural product-derived inhibitor of Keap1 (**1**)^9^ by over 100-fold, resulting in compounds displaying 30–70 nM nanomolar potencies. The increase in potency was achieved by first exploring the SAR of the series at multiple positions using a set of 36 compounds. This identified the N-terminus of the proline moiety, and the position *ortho* to the thiomethyl group of the phenylene ring of **1**, as suitable for further optimization. Structure-based design, including the generation of virtual libraries at either position, which were subsequently docked into Keap1, allowed the selection of two sets of macrocycles consisting of 46 and 20 compounds for synthesis. Substituents at proline targeted more exposed parts of the binding site on Keap1 and provided only minor increases in potency. In contrast, substituents located deeper in the binding site that formed a salt bridge with R483 provided a 100-fold increase in potency over **1**. A few series that also contain nanomolar inhibitors of Keap1 have previously been reported (*cf.*Figure 1B). These ligands also form polar interactions with R483, highlighting this interaction to be essential for high-affinity. The SAR exploration was greatly facilitated by establishing efficient routes that allowed the synthesis of macrocycle **1**, and analogues, *via* routes of 7–8 steps on a gram scale. The use of robust chemical transformations, that is, amide bond formation and the Suzuki reaction, for optimization at the two selected positions was also essential for the success of the project.

The macrocyclic moiety is an essential element of the lead series as a linear analogue of **1** displayed a 100-fold loss in potency.^9^ Moreover, the precise conformation of the macrocyclic ring needs to be maintained, as illustrated by the loss of activity of the oxygen analogue **6**. This is further supported by the observation that the conformation of the macrocyclic ring is maintained in the five crystal structures of novel inhibitors bound by Keap1 reported herein. Inspection of the crystal structures of the complexes with Keap1 also reveals that the macrocyclic ring positions the pharmacophore units of the inhibitors in orientations that allow productive interactions with Keap1. Thus, the phenylene ring is wedged between A556 and R415 forming a π-cation interaction, the C-terminal dimethylamide stacks against Y572, while the *para*-benzoic acid moiety of the nM inhibitors forms a “side-on” bidentate salt bridge^30^ with R483. However, the macrocyclic ring is not only a scaffold for the three pharmacophores since it is also involved in key interactions with Keap1. This includes a hydrogen bond between the carbonyl group of the lactam moiety and S602 in Keap1, which is also present in complexes of high-affinity ligands from other series.

The binding pocket of Keap1 is highly polar as it is lined by the side chains of three arginine residues, R380, R415, and R483. These three residues form salt bridges with the glutamic acid side chains in the ETGE binding motif of Nrf2 and must be considered in the design of inhibitors of the Keap1–Nrf2 PPI. The nanomolar inhibitors developed herein interact with two of the three arginine residues, that is, R415 and R483. The electronics of the π-cation interaction with R415 appears to be essential for the affinity of the series as attachment of an electron withdrawing nitrile to the phenylene ring of the inhibitors results in a 100-fold reduction in affinity for Keap1 (*cf.* compounds **16** and **21**). Formation of a “side-on” bidentate salt bridge^30^ with R483 as in the complex of *para*-benzoic acid **64** with Keap1, but not a “backside” salt-bridge as formed by *meta*-benzoic acid **63**, provides an optimal interaction with R483.

Importantly, our studies also suggest that it is essential to account for the flexibility and hydration of the binding-site arginine residues^9,34^ in the precise design of high-affinity ligands for Keap1. Comparison of the crystal structures of inhibitors **1**, **60**, **63**, and **64** reveals that a limited reorientation of R415 and R483 can have a profound effect on the affinity of Keap1 for a ligand. In addition, displacement of a water molecule that bridges S508 and R415 in the complex of Keap1 and compound **1** by substituents such as the *para*-benzoic acid of **64** alters the thermodynamic profile of the series from having a highly unfavorable entropy component to the one that is close to negligible. We assume that this displacement contributes to the 50–100-fold potency improvement of *para*-benzoic acid **64** over **1** in addition to the reorientation of R415 and R483.

To the best of our knowledge, no noncovalent inhibitor of the Keap1–Nrf2 PPI has yet entered clinical studies. It therefore remains to be established whether or not the release of Nrf2 *via* this mode of action will show clinical efficacy. The development and evaluation of novel Keap1 inhibitors should provide additional knowledge, helping to advance compounds into clinical studies in the future. However, so far, only a handful of series of Keap1 inhibitors display potencies in the drug-like range (*K*_D_ ≤100 nM). The series reported herein originates from a natural-product starting point and is therefore located in a region of the chemical space different from that of previously reported ligands for Keap1. This could provide an advantage in efforts toward its further optimization.

## Experimental Section

### VT NMR Experiments for Compounds **1**, **6**, and **7**

Spectra were recorded on an Agilent Technologies 400 MR spectrometer at 400 MHz (^1^H) and referenced to the magnet frequency (400 MHz) to circumvent reference signal drift. Amide-proton temperature shift coefficients (Δδ/*T*) were calculated from VT NMR experiments acquired for a DMSO-*d*_6_ solution in the temperature range 25–85 °C with 10 °C increments. Chemical shifts are reported in Table S1.

### NMR Analysis of Compound **1**

Compound **1** (6 mg) was dissolved in 120 μL of 20% H_2_O in DMSO-*d*_6_. H_2_O was used instead of D_2_O to prevent hydrogen–deuterium chemical exchange. All spectra were acquired on a Bruker AVANCE III spectrometer with a proton frequency of 600 MHz fitted with a QNP cryoprobe. All spectra were acquired at a nominal probe temperature of 298 K. Proton spectra were acquired with suppression to reduce the intense water resonance. The ^1^H NMR spectra were referenced to residual DMSO-*d*_5_ at 2.50 ppm. The proton spectrum was assigned using standard 1D and 2D methods. Individual assignments of the diastereotopic protons 19, 29, and 31 and methyl protons 33 and 34 were made based on the distances to adjacent protons determined from the rotating-frame nuclear Overhauser effect correlation spectroscopy (ROESY) spectrum. Assignments are shown in Table S2.

Interproton distances were estimated from a jump-symmetrized ROESY spectrum:^35^ Bruker pulse sequence: roesyadjphpr, spinlock time: 250 ms, tilt angle: 45°, recycle time: 5.3 s, total experiment time: 51 h. Cross-peak intensities were normalized to the diagonal by applying the PANIC correction.^36^ Where possible, cross-peaks were taken from both quadrants and averaged. A reference distance of 1.75 Å for the geminal H29 proton pair was used to convert the intensities of cross-peaks into an average distance.

### Monte Carlo Molecular Mechanics Conformational Search

The theoretical ensemble of compound **1** was obtained from Monte Carlo molecular mechanics (MCMM) calculations using the software Macromodel (v.9.1), as implemented in the Schrödinger package. OPLS and AMBER force fields, an energy window of 42 kJ mol^–1^, and 50 000 Monte Carlo steps were used. The energy minimization was performed using the Polak–Ribiere-type conjugate gradient (PRCG) with maximum iteration steps set to 5000. All obtained conformations (148 for OPLS and 156 for AMBER) were combined and subjected to redundant conformer eliminations (RCE) by comparison of the coordinates of heavy atoms with an rmsd cutoff = 1 Å. Additionally, the X-ray structure of compound **1** was added, providing the final ensemble employed in the NAMFIS analysis.

### NAMFIS Analysis

The solution ensemble of compound **1** was determined by fitting the experimentally measured distances to those back-calculated for the computationally predicted conformations following the NAMFIS protocol.^21^ The results of the NAMFIS analysis were validated by evaluating the variation of the conformational restraints upon addition of 10% random noise to the experimental distances, by the random removal of 10% of individual restrains, and by comparison of the experimentally observed and back-calculated distances.

### Cheminformatics and Preparation of Virtual Chemical Libraries

Chemical SMARTS patterns were used to retrieve molecules that had suitable functional groups for the intended organic synthesis from Enamine’s “in-stock” catalogs.^37,38^ Building blocks were coupled *in silico* to the parent scaffold using reaction SMIRKS patterns. Chemical pattern matching and reagent coupling were performed with in-house scripts based on OpenEye’s OEToolkits (version 2020.2). The product molecules were filtered using a PAINS-filter^29^ to reduce the risk of having false positives and additionally constrained to have the following physicochemical properties: MW ≤ 750 Da, HBA ≤ 20, HBD ≤ 4, 0 ≤ cLogP ≤ 5, NRotB ≤ 10, 110 ≤ TPSA ≤ 225. The products were prepared for docking using DOCK3.7 protocols (db2 format).^39^ ChemAxon’s CXCalc (from ChemAxon’s Marvin package Marvin 18.10.0) was used for calculating relevant protomers. Only sulfonamides that were estimated by ChemAxon CXCalc to be approximately 98% deprotonated at physiological pH levels were included in the docking screen. Conformational ensembles were generated with OpenEye’s OMEGA (version 2020.2) and capped at 200 conformations per rigid segment with an interconformer rmsd diversity threshold of 0.05 Å. Atoms in the macrocyclic core were constrained to the same relative coordinates in the cocrystallized macrocyclic inhibitor during conformer generation. As a result, conformational ensembles that emphasized sampling molecular geometries for newly introduced substituents were obtained.

### Molecular Docking

The crystal structure of Keap1 bound to compound **1** (PDB ID: 6Z6A(9)) was used in the docking screen of the virtual library. Crystallographic waters and ions were removed from the structure with the exception of the chloride anion 1 due to its direct interaction with the ligand. The atoms of the cocrystallized inhibitor were used to generate matching spheres in the active site. DOCK3.7^39^ uses a flexible ligand algorithm that superimposes rigid segments of a molecule’s precalculated conformational ensemble on top of the matching spheres. The N-terminus of the protease was acetylated, the C-terminus methylated, and the histidine protonation states assigned manually. To describe the hydrogen bonding network, histidines 424, 436, 437, 451, 516, 552, 562, and 575 were protonated at the N_ε_, whereas histidine 432 was protonated at the N_δ_. The remainder of the enzyme structure was protonated by REDUCE^40^ and assigned AMBER^41^ united atom charges. The atoms of the cocrystallized inhibitor were used to create two sets of so-called thin sphere layers on the protein surface. One set of spheres with radius 1.2 Å described the low protein dielectric and defined the boundary between the solute and solvent. A second set of spheres with a radius of 0.2 Å was used to calibrate ligand desolvation penalties. Scoring grids were precalculated using QNIFFT^42^ for Poisson–Boltzmann electrostatic potentials, SOLVMAP^43^ for ligand desolvation potentials, and CHEMGRID^44^ for AMBER van der Waals potentials. Property-matched and property-perturbed decoys of macrocyclic inhibitors were generated using in-house scripts.^45^ The obtained control sets were used to evaluate the performance of the docking grids by calculating the enrichment of ligands over decoys. The enrichment of ligands and the predicted binding poses of macrocycles were used to select the optimal grid parameters.^46^ For each ligand, the best scoring pose was optimized using a simplex rigid-body minimizer. In the screens of custom macrocyclic libraries, the 50 lowest-energy poses of each molecule were retained from the docking. The lowest-energy pose that had a macrocycle rmsd value below 2 Å with the cocrystallized inhibitor was considered as the most relevant pose. The rmsd value was calculated between the atoms that composed the macrocyclic core using an in-house script.

### FEP and MD

Free-energy calculations were carried out with the program Q,^47^ and the MD simulations were initiated based on the crystal structure of Keap1 bound to compound **1** (PDB ID: 6Z6A(9)). The OPLS all-atom force field^48^ was used together with the TIP3P water model.^49^ MD simulations were carried out using spherical boundary conditions with a sphere radius of 21 Å centered on the ligands. Relative binding free energies were calculated based on the alchemical transformation of one ligand into another in both the binding site and in the aqueous solution using 52–84 intermediate states. Free energies were calculated with the Zwanzig equation and a thermodynamic cycle, as described previously.^50^ Extended MD simulations were analyzed with the Python library MDTraj,^51^ and box plots were generated with Matplotlib.^52^ Detailed simulation protocols are described in the Supporting Information.

### SPR Inhibition in Solution Assay

The SPR ISA was performed in analogy to the protocol developed by Chen *et al.*(18) with the following differences. Instead of a biotinylated peptide, a lysine-tagged version of the Nrf2-peptide (KKKKAFFAQLQLDEETGEFL) was utilized for tethering to the sensor surface. For the covalent tethering, a CM5 biosensor (GE Healthcare—research grade) was employed using HBS-P [10 mM (4-(2-hydroxyethyl)-1-piperazineethanesulfonic acid) (HEPES), 150 mM NaCl, 0.05% (v/v) Tween 20, 1.0 mM tris(2-carboxyethyl)phosphine (TCEP), pH 7.40] as the running buffer at a rate of 10 μL/min at 20 °C on a BIAcore 3000 optical biosensor unit (GE Healthcare). The surface was activated by injecting a mixture of EDC and *N*-hydroxysuccinimide (NHS) for 7 min, followed by an injection of 50 μM Nrf2-peptide in 10 mM Na-acetate, pH 4.0, for 2 min. Any reactive groups still present on the surface were deactivated by a 7 min injection of 0.1 M ethanolamine hydrochloride–NaOH, pH 8.5. The peptide immobilization levels were typically between 400 and 600 RU to ensure mass transport limitation and thus a protein concentration-dependent response.

For the detection of active compounds, a solution of 25 nM Keap1 (Kelch domain = aa321–aa609) was prepared in the running buffer and preincubated with the compounds at either constant or varying concentrations. These mixtures were subsequently injected over the peptide-modified biosensor, and control samples devoid of the compounds were used to determine the remaining free protein concentration of Keap1 in response to compound binding. For this, the initial association slopes of the binding sensorgrams have been measured (interval 5–15 s after sample injection) for each sample, followed by a regeneration of the sensor for the next cycle through a 45 s injection of 50 mM Tris/Cl, 0.25% SDS, 5 mM TCEP, pH 7.5. The results have been reported as *K*_D_-values for concentration–response experiments. This was achieved by a nonlinear regression analysis of the concentration response data, which is normalized by the control samples representing 0% inhibition.

### SPR Direct Binding Assay

The SPR DBA was performed as described previously,^53^ with the difference that HBS-P [10 mM HEPES, 150 mM NaCl, 0.05% (v/v) Tween 20, 1.0 mM TCEP, pH 7.40] was used as a continuous-flow buffer and that the contact and dissociation times were 45 and 60 s, respectively.

### Isothermal Titration Calorimetry

ITC experiments were carried out on a MicroCal ITC-200 system (GE Healthcare) using the Keap1 protein (Kelch domain = aa321–aa609) that had been passed through a PD-10 column (GE-Healthcare) equilibrated with 10 mM HEPES, 150 mM NaCl, 0.05% (v/v) Tween 20, 1 mM TCEP, pH 7.40, and 1% DMSO. Complete titration of 60 μM Keap1 was typically achieved by injecting 19 × 2 μL aliquots of 0.6 mM compound at a temperature of 25 °C and a spacing interval of 90 s between injections. The thermodynamic binding parameters were extracted by the nonlinear regression analysis of the binding isotherms (Microcal Origin version 7.0 software package). A single-site binding model was applied, yielding binding enthalpy (Δ*H*), stoichiometry (*n*), entropy (Δ*S*), and association constant (*K*_a_).

### Aqueous Solubility

The aqueous solubility of compounds **64**, **77**, and **78** was determined at AstraZeneca as reported previously.^54^

### Cell Permeability

The efflux inhibited permeability of compounds **64**, **77**, and **78** across a Caco-2 cell monolayer was determined in the presence of a cocktail of inhibitors of efflux transporters (50 μM quinidine, 30 μM benzbromarone, and 20 μM sulfasalazine) by the DMPK department at Pharmaron as reported previously.^55^

### Clearance by Human Liver Microsomes and Rat Hepatocytes

The clearance of compounds **64**, **77**, and **78** by human liver microsomes and rat hepatocytes (CL_int_) was determined at AstraZeneca as reported previously.^54^

### Cellular Assay

The cellular activity of macrocycles **64** and **77** was determined by DiscoveRx using their PathHunter U2OS Keap1–Nrf2 functional assay,^56^ which quantifies the translocation of Nrf2 into the nucleus, resulting from the inhibition of the formation of the Keap1–Nrf2 complex.

### Keap1 Expression, Purification, and Crystallization

The DNA coding domain for human Keap1 (6× His-TCS-Keap1(A321–T609) [E540A, E542S]) was cloned into pET24 and expressed in *Escherichia coli* BL21(DE3)-Star in LB media at 291 K. After harvest, the cells were resuspended in 20 mM Tris/HCl, pH 7.5, 200 mM NaCl, 5% glycerol, 1 mM DTT, disrupted with a high-pressure homogenizer, and clarified by centrifugation. Purification was carried out by affinity chromatography using Ni Sepharose FF (GE Healthcare), eluted in one step with 300 mM imidazole, followed by size exclusion chromatography (SEC, Superdex200, GE Healthcare). The purified protein was detagged at room temperature (rt) with a thrombin cleaving kit (Sigma) and finalized by a second SEC step (Superdex200, GE Healthcare) in a buffer of 20 mM Tris/HCl, pH 7.5, 5 mM DTT before concentration (Amicon cells, 10 kDa cut off). The crystals were grown at 20 °C in sitting drops using 200 nL of protein (11–13 mg/mL) and 200 nL of the well solution (3.7–4.1 M ammonium acetate, 0.09 M sodium acetate pH 4.6, and 10 mM cadmium chloride). Crystals appeared after 1 day but continued to grow for approximately 1 week. Selected compounds were soaked by incubating the crystals for 45–60 min with 1–2 mM of the compound in 5 M ammonium acetate and 0.1 m sodium acetate at pH 4.6. The crystals were subsequently frozen in liquid nitrogen using a soaking solution supplemented with 20% glycerol as the cryo-protectant prior to data collection.

### Data Collection, Structure Solution, and Refinement

X-ray diffraction data were collected at the Max Lab IV BioMax beamline and at the European Synchrotron Facility, beamlines ID30B and ID 231 to resolutions between 2.1 and 2.6 Å. All data were indexed, integrated, and scaled with AutoPROC^57^ in the space group *P*2_1_2_1_2_1_ with the cell dimensions of 75, 75, and 202 Å. Two Keap1 molecules were identified by PHASER^58^ using a published Keap1 structure (PDB ID: 1ZGK(59)) in each data set. The Keap1 macrocycle complex was further refined by alternative cycles of model rebuilding in Coot^60^ and refinement in AutoBuster 2.11.6 (Global Phasing Ltd, Cambridge UK).

### Chemistry

#### General Methods

All reagents were purchased from Sigma-Aldrich, Fluorochem, and VWR International. The reagents used for the preparation of compounds **41**–**58** were purchased from Enamine Ltd. DCM, DMSO, hexane, DMF, and acetonitrile were purchased from VWR International, while 1,2-DCE, toluene, and THF were purchased from Sigma-Aldrich. All nonaqueous reactions were performed in oven-dried glassware under an argon atmosphere. The Büchi rotary evaporator R-114 was used to remove solvents *in vacuo*. The reactions were generally monitored using liquid chromatography–mass spectrometry (LC–MS) with an Agilent 1100 series high-performance liquid chromatography (HPLC) with a C18 Atlantis T3 column (3.0 mm × 50 mm, 5 μm) using acetonitrile–water (flow rate 0.75 mL/min over 6 min) as the mobile phase and a Waters micromass ZQ (model code: MM1) mass spectrometer with electrospray ionization (ESI) mode as the detector. Alternatively, TLC silica gel 60 F_254_ plates from VWR International were used and visualization was done using UV light (254 nm). Silica gel (43–63 μm, VWR international) was used for the purification of compounds with flash column chromatography. Preparative reversed-phase HPLC was performed on a Kromasil C8 column (250 mm × 21.2 mm, 5 μm) on a Gilson HPLC equipped with a Gilson 322 pump, a UV–visible-156 detector, and a 202 collector using acetonitrile–water gradients as the eluents with a flow rate of 15 mL/min and detection at 214 or 254 nm. ^1^H and ^13^C NMR spectra for the synthesized compounds were recorded at 298 K on an Agilent Technologies 400 MR spectrometer at 400 MHz (^1^H) or 100 MHz (^13^C) or on a Bruker 500 AVANCE Neo spectrometer at 500 MHz (^1^H) or 126 MHz (^13^C) or on a Bruker 600 AVANCE Neo spectrometer at 600 MHz (^1^H) or at 151 MHz (^13^C). Chemical shifts are reported in parts per million (δ) and referenced from the residual protonium for ^1^H NMR [CDCl_3_: δ 7.26 (CHCl_3_); DMSO-*d*_6_: δ 2.50 (DMSO-*d*_5_)]. ^13^C NMR spectra are referenced from the carbon reference of the solvent [CDCl_3_: δ 77.2; DMSO-*d*_6_: δ 39.5]. The HRMS spectra for all new compounds were recorded in ESI mode on an S3 LCT Premier connected to a Waters Acquity UPLC I-class with acetonitrile–water used as the mobile phase (1:1, with a flow rate of 0.25 mL/min). The purity of compounds **2–78** is ≥95%, with the exception of compounds **45**, **56**, and **68** which are 93, 94, and 93% pure, respectively. For purity analysis, a GenTech Scientific Waters ACQ equipped with an Acquity UPLC system, an HSS C18 column (1.8 μm, 2.1 mm × 50 mm), and an SQ2 detector with a wavelength range of 220–350 nm was used. Acetonitrile and water (modified either with 47 mM ammonia and 6.5 mM ammonium carbonate, pH 10, or with 1 mM ammonium formate and 10 mM formic acid, pH 3) were used as the mobile phases.

#### (4*S*,7*R*)-7-((*S*)-1-Acetylpyrrolidine-2-carboxamido)-*N*,*N*-dimethyl-6,10-dioxo-1,3,4,5,6,7,8,10-octahydrobenzo[*j*][1]oxa[8]thia[5]azacyclododecine-4-carboxamide 2-Oxide (**2**)

Compound **1** (23 mg, 47 μmol, 1.0 equiv) was dissolved in DCM (2 mL) and cooled down to 0 °C. *m*-Chloroperbenzoic acid (70%, 13 mg, 53 μmol, 1.1 equiv) was added, and the mixture was allowed to warm up to rt and stirred for 1 h. After the evaporation of the volatiles under reduced pressure, the crude product was purified by reverse-phase HPLC with a gradient 15–75% MeCN/water to give **2** (7 mg, 14 μmol, 30% yield) as a colorless powder. The compound was isolated as a single diastereoisomer. The formation of a minor diastereoisomer was detected by LC–MS, but the isolation of a significant amount was not possible on this reaction scale. HRMS (ESI) *m*/*z*: calcd for C_23_H_31_N_4_O_7_S [M + H]^+^, 507.1908; found, 507.1915. ^1^H NMR (500 MHz, CDCl_3_): δ 8.22 (d, *J* = 7.8 Hz, 1H), 7.94–7.83 (m, 2H), 7.57 (t, *J* = 7.8 Hz, 1H), 7.53–7.42 (m, 2H), 5.71–5.65 (m, 1H), 5.05–4.97 (m, 2H), 4.84–4.76 (m, 1H), 4.54 (dd, *J* = 11.2, 2.0 Hz, 1H), 4.42 (dd, *J* = 7.6, 3.8 Hz, 1H), 4.09 (d, *J* = 12.6 Hz, 1H), 3.88–3.81 (m, 1H), 3.76–3.61 (m, 1H), 3.54–3.49 (m, 1H), 3.13 (s, 3H), 3.09 (dd, *J* = 13.9, 3.7 Hz, 1H), 2.97 (s, 3H), 2.37–2.32 (m, 1H), 2.29–2.22 (m, 1H), 2.20 (s, 3H), 2.06–1.96 (m, 2H). ^13^C NMR (126 MHz, CDCl_3_): δ 172.4, 171.4, 168.1, 168.0, 166.8, 134.5, 133.3, 133.2, 131.9, 128.9, 128.8, 67.2, 62.3, 60.2, 58.3, 52.9, 48.5, 45.2, 37.2, 36.1, 27.7, 25.3, 22.8.

#### (4*S*,7*R*)-7-((*S*)-1-Acetylpyrrolidine-2-carboxamido)-*N*,*N*-dimethyl-6,10-dioxo-1,3,4,5,6,7,8,10-octahydrobenzo[*j*][1]oxa[8]thia[5]azacyclododecine-4-carboxamide 2,2-Dioxide (**3**)

Compound **1** (10 mg, 20 μmol, 1.0 equiv) was dissolved in DCM (1 mL). *m*-Chloroperbenzoic acid (70%, 15 mg, 61 μmol, 3.0 equiv) was added, and the mixture was stirred at rt for 2 h. After the evaporation of the volatiles under reduced pressure, the crude product was purified by reverse-phase HPLC with a gradient 15–75% MeCN/water to give **3** (7 mg, 13 μmol, 67% yield) as a colorless powder. HRMS (ESI) *m*/*z*: calcd for C_23_H_31_N_4_O_8_S [M + H]^+^, 523.1857; found, 523.1842. ^1^H NMR (500 MHz, CDCl_3_): δ 8.41 (d, *J* = 9.6 Hz, 1H), 8.14 (d, *J* = 7.8 Hz, 1H), 7.63–7.56 (m, 2H), 7.56–7.49 (m, 1H), 7.37 (d, *J* = 8.9 Hz, 1H), 5.64 (ddd, *J* = 11.4, 9.6, 3.5 Hz, 1H), 5.26–5.21 (m, 2H), 4.85 (dt, *J* = 8.9, 2.1 Hz, 1H), 4.63 (d, *J* = 12.8 Hz, 1H), 4.38 (dd, *J* = 11.0, 2.1 Hz, 1H), 4.32 (dd, *J* = 8.0, 4.5 Hz, 1H), 4.13 (dd, *J* = 15.6, 11.4 Hz, 1H), 3.72–3.66 (m, 1H), 3.59–3.52 (m, 1H), 3.37 (dd, *J* = 15.6, 3.5 Hz, 1H), 3.19 (s, 3H), 2.95 (s, 3H), 2.34–2.22 (m, 2H), 2.18 (s, 3H), 2.11–2.06 (m, 1H), 2.04–1.96 (m, 1H). ^13^C NMR (126 MHz, CDCl_3_): δ 172.0, 171.8, 168.5, 167.6, 166.5, 134.5, 133.0, 132.7, 131.1, 129.3, 125.7, 66.9, 60.6, 58.6, 55.8, 53.3, 48.6, 45.5, 37.4, 36.3, 28.5, 25.4, 22.7.

#### (4*S*,7*R*)-7-((*S*)-1-Acetylpyrrolidine-2-carboxamido)-*N*,*N*-dimethyl-6,10-dioxo-1,3,4,5,6,7,8,10-octahydrobenzo[*j*][1]oxa[8]thia[5]azacyclododecine-4-carboxamide 2,2-Dioxide (**4**)

Compound **1** (30 mg, 61 μmol, 1.0 equiv), ammonium carbamate (10 mg, 0.12 mmol, 2.0 equiv), and (diacetoxyiodo)benzene (49 mg, 0.15 mmol, 2.5 equiv) were dissolved in MeOH (2 mL), and the mixture was stirred at rt for 2 h. The solvent was evaporated under reduced pressure, and the crude product was purified by reverse-phase HPLC with a gradient 15–70% MeCN/water to give **4** (8 mg, 15 μmol, 25% yield) as a colorless powder. The compound was isolated as a single diastereoisomer. The formation of a minor diastereoisomer was detected by LC–MS, but the isolation of a significant amount was not possible on this reaction scale. HRMS (ESI) *m*/*z*: calcd for C_23_H_32_N_5_O_7_S [M + H]^+^, 522.2017; found, 522.2011. ^1^H NMR (500 MHz, CDCl_3_): δ 8.18–8.11 (m, 2H), 7.78 (d, *J* = 7.6 Hz, 1H), 7.63 (td, *J* = 7.5, 1.5 Hz, 1H), 7.58–7.53 (m, 2H), 5.70 (td, *J* = 9.1, 3.3 Hz, 1H), 5.59 (d, *J* = 12.2 Hz, 1H), 5.30 (dd, *J* = 11.2, 2.1 Hz, 1H), 5.09 (d, *J* = 12.2 Hz, 1H), 4.76 (ddd, *J* = 8.6, 7.6, 2.1 Hz, 1H), 4.40–4.34 (m, 2H), 4.13 (dd, *J* = 15.0, 9.1 Hz, 1H), 3.80–3.74 (m, 1H), 3.66–3.59 (m, 1H), 3.53–3.47 (m, 1H), 3.13 (s, 3H), 2.95 (s, 3H), 2.33–2.26 (m, 1H), 2.22–2.18 (m, 1H), 2.07 (s, 3H), 2.06–1.94 (m, 2H). *The SONHproton was not detectable in this spectrum.*^13^C NMR (126 MHz, CDCl_3_): δ 172.1, 171.9, 168.6, 167.9, 167.0, 134.8, 133.2, 132.7, 130.7, 129.6, 126.5, 66.3, 61.0, 60.4, 57.5, 53.6, 48.4, 47.6, 37.5, 36.3, 28.1, 25.3, 22.6.

#### (*R*)-3-(((Allyloxy)carbonyl)amino)-2-((*tert*-butoxycarbonyl)amino)propanoic Acid (**79**)

(*R*)-3-Amino-2-((*tert*-butoxycarbonyl)amino)propanoic acid (600 mg, 2.94 mmol, 1.0 equiv) was dissolved in 1,4-dioxane (5 mL) and water (5 mL). K_2_CO_3_ (1.22 g, 8.82 mmol, 3.0 equiv) and allyl chloroformate (627 μL, 5.88 mmol, 2.0 equiv) were added, and the mixture was stirred for 16 h at rt. Water (25 mL) was added, and the mixture was extracted with Et_2_O (25 mL). The aqueous phase was acidified with concentrated aqueous HCl and extracted with DCM (3 × 50 mL). The combined organic phase was dried over MgSO_4_, filtered, and concentrated under reduced pressure, providing **79** as a yellow oil (719 mg, 2.49 mmol, 85% yield). HRMS (ESI) *m*/*z*: calcd for C_23_H_32_N_5_O_5_S [M + H]^+^, 490.2119; found, 490.2108. ^1^H NMR (400 MHz, DMSO-*d*_6_): δ 12.62 (br s, 1H), 7.19 (t, *J* = 6.1 Hz, 1H), 6.91 (d, *J* = 8.2 Hz, 1H), 5.86 (ddt, *J* = 17.2, 10.5, 5.3 Hz, 1H), 5.24 (dd, *J* = 17.2, 1.8 Hz, 1H), 5.13 (dd, *J* = 10.5, 1.8 Hz, 1H), 4.44–4.40 (m, 2H), 4.03–3.95 (m, 1H), 3.30–3.25 (m, 2H), 1.35 (s, 9H). ^13^C NMR (101 MHz, DMSO-*d*_6_): δ 172.6, 156.5, 155.8, 134.0, 117.3, 78.7, 64.8, 54.1, 42.0, 28.6.

#### Methyl 2-((4*S*,7*R*)-7-((*tert*-Butoxycarbonyl)amino)-4-(dimethylcarbamoyl)-6,10-dioxo-11-oxa-2-thia-5,9-diazatetradec-13-en-1-yl)benzoate (**81**)

Compound 80^9^ (802 mg, 2.03 mmol, 1.0 equiv) was dissolved in 4 M HCl in 1,4-dioxane (10 mL) and stirred for 1 h at rt. After evaporation under reduced pressure, water (25 mL) and K_2_CO_3_ (700 mg, 5.08 mmol, 2.5 equiv) were added, and the aqueous phase was extracted with DCM (3 × 75 mL). The combined organic phase was dried over MgSO_4_, filtered, and concentrated under reduced pressure. The resulting oil was dissolved in MeCN (10 mL), and compound **79** (642 mg, 2.23 mmol, 1.1 equiv) and EDC·HCl (426 mg, 2.23 mmol, 1.1 equiv) were added, and the reaction mixture was stirred for 1 h at rt. EtOAc (150 mL) was added, and the mixture was washed with a 1 M aqueous HCl solution (100 mL), saturated aqueous NaHCO_3_ solution (100 mL), and brine (100 mL). The organic phase was dried over MgSO_4_, filtered, and concentrated under reduced pressure. The crude product was purified by flash chromatography on a silica gel column using 1–10% MeOH/DCM as the eluent to give **81** (799 mg, 1.41 mmol, 70% yield) as a colorless powder. HRMS (ESI) *m*/*z*: calcd for C_26_H_39_N_4_O_8_S [M + H]^+^, 567.2483; found, 567.2471. ^1^H NMR (600 MHz, CDCl_3_): δ 7.90 (d, *J* = 7.7, 1H), 7.45 (t, *J* = 7.6, 1H), 7.39 (d, *J* = 7.6 Hz, 1H), 7.32 (t, *J* = 7.7, 1H), 7.20–7.11 (m, 1H), 5.88 (ddt, *J* = 17.2, 10.4, 5.5 Hz, 1H), 5.79–5.70 (m, 1H), 5.59–5.52 (, 1H), 5.28 (d, *J* = 17.2 Hz, 1H), 5.19 (d, *J* = 10.4 Hz, 1H), 5.04–4.98 (m, 1H), 4.57 (dd, *J* = 13.4, 5.5 Hz, 1H), 4.52 (dd, *J* = 13.4, 5.5 Hz, 1H), 4.28–4.22 (m, 1H), 4.18 (d, *J* = 13.1 Hz, 1H), 4.12 (d, *J* = 13.1 Hz, 1H), 3.91 (s, 3H), 3.64–3.53 (m, 2H), 3.02 (s, 3H), 2.93 (s, 3H), 2.83 (dd, *J* = 13.9, 6.1 Hz, 1H), 2.66 (dd, *J* = 13.9, 7.2 Hz, 1H), 1.44 (s, 9H). ^13^C NMR (151 MHz, CDCl_3_): δ 170.0, 169.9, 167.8, 157.4, 155.8, 140.0, 132.6, 132.0, 131.2, 131.2, 129.5, 127.3, 117.8, 80.5, 65.9, 55.7, 52.3, 48.5, 42.9, 37.3, 35.9, 34.9, 34.3, 28.3.

#### Methyl 2-((4*S*,7*R*)-7-(Aminomethyl)-4-(dimethylcarbamoyl)-11,11-dimethyl-6,9-dioxo-10-oxa-2-thia-5,8-diazadodecyl)benzoate (**82**)

A 100 mL round-bottom flask was charged with compound **81** (700 mg, 1.24 mmol, 1.0 equiv), K_2_CO_3_ (342 mg, 2.48 mmol, 2.0 equiv), and tetrakis(triphenylphosphine)palladium (36 mg, 31 μmol, 2.5 mol %). After the flask was evacuated and refilled with argon three times, MeOH (12 mL) was added, and the reaction mixture was stirred for 2 h at rt. The solvent was concentrated under reduced pressure, and the crude product was purified by flash chromatography on a silica gel column using 1–20% MeOH/DCM as the eluent to give **82** (425 mg, 0.88 mmol, 71% yield) as a yellow oil. HRMS (ESI) *m*/*z*: calcd for C_22_H_35_N_4_O_6_S [M + H]^+^, 483.2272; found, 483.2257. ^1^H NMR (600 MHz, CDCl_3_): δ 7.90 (d, *J* = 7.7 Hz, 1H), 7.84–7.75 (m, 1H), 7.44 (t, *J* = 7.5 Hz, 1H), 7.39 (d, *J* = 7.5 Hz, 1H), 7.32 (t, *J* = 7.7 Hz, 1H), 5.67 (d, *J* = 7.0 Hz, 1H), 5.05–5.01 (m, 1H), 4.17 (d, *J* = 13.2 Hz, 1H), 4.13 (d, *J* = 13.2 Hz, 1H), 4.13–4.09 (m, 1H), 3.90 (s, 3H), 3.29–3.21 (m, 1H), 3.02 (s, 3H), 2.94 (s, 3H), 2.85–2.79 (m, 2H), 2.67 (dd, *J* = 13.8, 7.0 Hz, 1H), 1.44 (s, 9H). *The NH*_*2*_*protons were not detectable in this spectrum.*^13^C NMR (151 MHz, CDCl_3_): δ 170.8, 170.4, 167.8, 155.7, 140.1, 132.0, 131.2, 131.2, 129.5, 127.3, 80.1, 55.1, 52.2, 48.6, 43.7, 37.4, 36.0, 35.0, 34.2, 28.3.

#### *tert*-Butyl-((4*S*,7*R*)-4-(dimethylcarbamoyl)-6,10-dioxo-3,4,5,6,7,8,9,10-octahydro-1*H*-benzo[*j*][1]thia[4,8]diazacyclododecin-7-yl)carbamate (**83**)

Compound **82** (75 mg, 0.16 mmol, 1.0 equiv) was dissolved in MeOH (3 mL). Subsequently, a 0.25 M aqueous LiOH (10 mL) was added, and the mixture was warmed to 40 °C and stirred for 16 h. MeOH was evaporated under reduced pressure and the water removed by freeze-drying. After the obtained solid was dissolved in DMF (4 mL), HATU (122 mg, 0.32 mmol, 2.0 equiv) and DIPEA (84 μL, 0.48 mmol, 3.0 equiv) were added, and the reaction mixture was stirred for 2 h at rt. The crude product was purified by reverse-phase HPLC with a gradient 20–75% MeCN/water to give **83** (15 mg, 33 μmol, 21% over two steps) as a colorless powder. HRMS (ESI) *m*/*z*: calcd for C_21_H_31_N_4_O_5_S [M + H]^+^, 451.2010; found, 451.2002. ^1^H NMR (500 MHz, CDCl_3_): δ 7.45 (d, *J* = 9.4 Hz, 1H), 7.41–7.34 (m, 3H), 7.35–7.27 (m, 1H), 6.74 (t, *J* = 6.4 Hz, 1H), 6.65–6.58 (m, 1H), 5.23 (ddd, *J* = 10.6, 9.4, 3.9 Hz, 1H), 4.60–4.48 (m, 1H), 4.15–4.05 (m, 1H), 4.01 (d, *J* = 10.6 Hz, 1H), 3.88 (d, *J* = 10.6 Hz, 1H), 3.87–3.80 (m, 1H), 3.09 (s, 3H), 3.03–2.99 (m, 1H), 2.98 (s, 3H), 2.80 (dd, *J* = 15.2, 10.6 Hz, 1H), 1.51 (s, 9H). ^13^C NMR (126 MHz, CDCl_3_): δ 171.9, 170.4, 169.5, 156.0, 136.8, 133.5, 131.0, 130.3, 127.9, 127.0, 80.9, 57.6, 49.8, 43.2, 37.7, 37.2, 35.9, 35.3, 28.3.

#### (4*S*,7*R*)-7-((*S*)-1-Acetylpyrrolidine-2-carboxamido)-*N*,*N*-dimethyl-6,10-dioxo-3,4,5,6,7,8,9,10-octahydro-1*H*-benzo[*j*][*j*][1]thia[4,8]diazacyclododecine-4-carboxamide (**5**)

Compound **83** (9 mg, 20 μmol, 1.0 equiv) was dissolved in 4 M HCl in 1,4-dioxane (2 mL) and stirred for 1 h at rt. After evaporation of the volatiles under reduced pressure, the resulting salt was dissolved in DMSO (1 mL). Ac-l-Pro-OH (16 mg, 0.10 mmol, 5.0 equiv), EDC·HCl (20 mg, 0.10 mmol, 5.0 equiv), and DIPEA (20 μL, 0.12 mmol, 6.0 equiv) were added, and the reaction mixture was stirred for 2 h at rt. The crude product was purified by reverse-phase HPLC with a gradient 15–75% MeCN/water to give **5** (8 mg, 16 μmol, 80% yield over two steps) as a colorless powder. HRMS (ESI) *m*/*z*: calcd for C_23_H_32_N_5_O_5_S [M + H]^+^, 490.2119; found, 490.2108. ^1^H NMR (500 MHz, CDCl_3_): δ 8.36 (d, *J* = 9.8 Hz, 1H), 8.24 (d, *J* = 7.0 Hz, 1H), 7.43–7.35 (m, 3H), 7.34–7.30 (m, 1H), 6.99–6.89 (m, 1H), 5.05 (ddd, *J* = 11.3, 9.8, 4.5 Hz, 1H), 4.68–4.58 (m, 1H), 4.29–4.24 (m, 1H), 4.19 (dd, *J* = 14.3, 8.0 Hz, 1H), 4.08 (d, *J* = 10.5 Hz, 1H), 3.95 (d, *J* = 10.5 Hz, 1H), 3.86 (dt, *J* = 14.3, 4.9 Hz, 1H), 3.67 (dt, *J* = 10.0, 7.1 Hz, 1H), 3.54 (ddd, *J* = 10.0, 7.1, 5.1 Hz, 1H), 3.17 (dd, *J* = 15.0, 11.3 Hz, 1H), 3.11 (dd, *J* = 15.0, 4.5 Hz, 1H), 3.02 (s, 3H), 2.92 (s, 3H), 2.28–2.21 (m, 1H), 2.20–2.15 (m, 2H), 2.09 (s, 3H), 2.01–1.92 (m, 1H). ^13^C NMR (126 MHz, CDCl_3_): δ 172.8, 172.7, 170.9, 169.3, 169.2, 136.9, 133.0, 131.1, 130.2, 127.8, 126.9, 77.0, 76.8, 61.0, 56.9, 50.6, 48.4, 42.0, 38.2, 37.1, 36.0, 35.3, 29.0, 25.4, 22.4.

#### *tert*-Butyl (*R*)-(1-(Dimethylamino)-3-hydroxy-1-oxopropan-2-yl)carbamate (**84**)

Boc-d-Ser-OH (6.00 g, 29.3 mmol, 1 equiv) was dissolved in DMF (60 mL). Dimethylamine hydrochloride (3.57 g, 43.9 mmol, 1.5 equiv), EDC·HCl (8.38 g, 43.9 mmol, 1.5 equiv), HOBt·*x*H_2_O (5.92 g, 43.9 mmol, 1.5 equiv), and DIPEA (7.65 mL, 43.9 mmol, 1.5 equiv) were added, and the mixture was stirred for 2 h at rt. The mixture was diluted with EtOAc (300 mL) and washed with a 1 M aqueous HCl solution (150 mL), saturated aqueous NaHCO_3_ solution (150 mL), and brine (150 mL). The organic phase was dried over MgSO_4_, filtered, and concentrated under reduced pressure, providing **84** as a yellow oil (2.72 g, 11.7 mmol, 40% yield). HRMS (ESI) *m*/*z*: calcd for C_10_H_21_N_2_O_4_ [M + H]^+^, 233.1496; found, 233.1490. ^1^H NMR (400 MHz, CDCl_3_): δ 4.69–4.64 (m, 1H), 3.82 (dd, *J* = 11.4, 4.3 Hz, 1H), 3.74 (dd, *J* = 11.4, 4.2 Hz, 1H), 3.13 (s, 3H), 2.99 (s, 3H), 1.45 (s, 9H). *The CH*_*2*_*OHand NHprotons were not detectable in this spectrum.*^13^C NMR (101 MHz, CDCl_3_): δ 170.3, 155.9, 80.2, 64.3, 51.7, 37.2, 35.8, 28.3.

#### Methyl-(*R*)-2-((2-((*tert*-butoxycarbonyl)amino)-3-(dimethylamino)-3-oxopropoxy)methyl)benzoate (**85**)

Compound **84** (900 mg, 3.85 mmol, 1.0 equiv) was dissolved in DMF (20 mL) and cooled down to 0 °C. Sodium hydride (60% dispersion in mineral oil, 162 mg, 4.04 mmol, 1.05 equiv) was added in one portion, and the mixture was stirred for 30 min at 0 °C. Methyl 2-(bromomethyl)benzoate (881 mg, 3.85 mmol, 1.0 equiv) was added, and the mixture was stirred for an additional 15 min. The reaction was quenched with water (25 mL) and subsequently diluted with EtOAc (200 mL). The organic phase was washed with a saturated aqueous NaHCO_3_ solution (2 × 50 mL) and brine (100 mL), and the organic phase was dried over MgSO_4_, filtered, and concentrated under reduced pressure. The crude reaction mixture was purified by flash chromatography on a silica gel column using 10–100% EtOAc/hexane as the eluent to give **85** (904 mg, 2.38 mmol, 62% yield) as a yellow oil. HRMS (ESI) *m*/*z*: calcd for C_14_H_21_N_2_O_4_ [M–Boc + H^+^]^+^, 281.1496; found, 281.1499. ^1^H NMR (400 MHz, CDCl_3_): δ 7.92 (d, *J* = 7.7 Hz, 1H), 7.58 (d, *J* = 7.5 Hz, 1H), 7.51 (t, *J* = 7.7 Hz, 1H), 7.33 (t, *J* = 7.5 Hz, 1H), 5.50 (d, *J* = 8.0 Hz, 1H), 4.96–4.84 (m, 3H), 3.88 (s, 3H), 3.75–3.63 (m, 2H), 3.11 (s, 3H), 2.99 (s, 3H), 1.43 (s, 9H). ^13^C NMR (101 MHz, CDCl_3_): δ 170.5, 167.4, 155.2, 140.3, 132.3, 130.4, 128.0, 127.4, 127.0, 79.7, 71.7, 71.3, 52.0, 50.0, 37.4, 35.9, 28.3.

#### Methyl 2-((4*R*,7*R*)-4-(Dimethylcarbamoyl)-7-(hydroxymethyl)-11,11-dimethyl-6,9-dioxo-2,10-dioxa-5,8-diazadodecyl)benzoate (**86**)

Compound **85** (880 mg, 2.32 mmol, 1.0 equiv) was dissolved in 4 M HCl in 1,4-dioxane (7 mL) and stirred for 1 h at rt. After evaporation of the volatiles under reduced pressure, water (20 mL) and K_2_CO_3_ (800 mg, 5.80 mmol, 2.5 equiv) were added, and the aqueous phase was extracted with DCM (3 × 100 mL). The organic phase was dried over MgSO_4_, filtered, and concentrated under reduced pressure. After the resulting oil was dissolved in MeCN (15 mL), Boc-d-Ser-OH (713 mg, 3.48 mmol, 1.5 equiv) and EDC·HCl (665 mg, 3.48 mmol, 1.5 equiv) were added, and the reaction mixture was stirred for 2 h at rt. EtOAc (100 mL) was added, and the mixture was washed with a 1 M aqueous HCl solution (50 mL), saturated aqueous NaHCO_3_ solution (50 mL), and brine (100 mL). The organic phase was dried over MgSO_4_, filtered, and concentrated under reduced pressure. The crude product was purified by flash chromatography on a silica gel column using 1–10% MeOH/DCM as the eluent to give **86** (816 mg, 1.74 mmol, 75% yield) as a colorless powder. HRMS (ESI) *m*/*z*: calcd for C_22_H_34_N_3_O_8_ [M + H]^+^, 468.2340; found, 468.2329. ^1^H NMR (400 MHz, CDCl_3_): δ 7.93 (d, *J* = 7.7 Hz, 1H), 7.56–7.49 (m, 2H), 7.39–7.31 (m, 1H), 7.23 (d, *J* = 7.0 Hz, 1H), 5.50 (d, *J* = 7.6 Hz, 1H), 5.18–5.08 (m, 1H), 4.92 (d, *J* = 13.7 Hz, 1H), 4.87 (d, *J* = 13.8 Hz, 1H), 4.30–4.20 (m, 1H), 4.05–3.96 (m, 1H), 3.90 (s, 3H), 3.81–3.69 (m, 2H), 3.63 (dd, *J* = 11.2, 6.2 Hz, 1H), 3.10 (s, 3H), 2.99 (s, 3H), 1.44 (s, 9H). *The CH*_*2*_*OHproton was not detectable in this spectrum.*^13^C NMR (101 MHz, CDCl_3_): δ 171.1, 169.7, 167.6, 155.6, 140.2, 132.4, 130.6, 128.3, 127.8, 127.4, 80.2, 71.4, 70.2, 63.4, 54.5, 52.2, 49.4, 37.8, 35.5, 28.3.

#### 2-((4*R*,7*R*)-4-(Dimethylcarbamoyl)-7-(hydroxymethyl)-11,11-dimethyl-6,9-dioxo-2,10-dioxa-5,8-diazadodecyl)benzoic Acid (**87**)

Compound **86** (800 mg, 1.71 mmol, 1.0 equiv) was dissolved in MeOH (10 mL). Subsequently, a 0.25 M aqueous solution of LiOH (10 mL) was added, and the reaction mixture was stirred at 40 °C for 16 h in a preheated oil bath. The reaction was allowed to cool down to rt and acidified with a 1 M aqueous HCl solution (15 mL) and extracted with DCM (3 × 50 mL). The organic phase was dried over MgSO_4_, filtered, and concentrated under reduced pressure. The crude product was purified by flash chromatography on a silica gel column using 5–25% MeOH/DCM as the eluent to give **87** (534 mg, 1.17 mmol, 69% yield) as a colorless powder. HRMS (ESI) *m*/*z*: calcd for C_21_H_32_N_3_O_8_ [M + H]^+^, 454.2184; found, 454.2167. ^1^H NMR (400 MHz, DMSO-*d*_6_): δ 12.91 (br s, 1H), 7.99 (d, *J* = 8.0 Hz, 1H), 7.85 (d, *J* = 7.7 Hz, 1H), 7.60–7.53 (m, 2H), 7.41–7.33 (m, 1H), 6.72 (d, *J* = 8.2 Hz, 1H), 5.04–4.96 (m, 1H), 4.85–4.79 (m, 2H), 4.02–3.96 (m, 1H), 3.69–3.62 (m, 1H), 3.60–3.54 (m, 1H), 3.54–3.45 (m, 2H), 3.03 (s, 3H), 2.84 (s, 2H), 1.38 (s, 9H). *The CH*_*2*_*OHproton was not detectable in this spectrum.*^13^C NMR (101 MHz, DMSO-*d*_6_): δ 170.4, 169.7, 168.6, 154.9, 140.4, 133.1, 130.6, 129.2, 127.6, 127.5, 79.6, 72.6, 70.7, 63.2, 58.1, 48.9, 36.6, 35.7, 28.6.

#### *tert*-Butyl-((4*R*,7*R*)-4-(dimethylcarbamoyl)-6,10-dioxo-1,3,4,5,6,7,8,10-octahydrobenzo[*j*][1,8]dioxa[4]azacyclododecin-7-yl)carbamate (**88**)

Compound **87** (104 mg, 0.229 mmol, 1.0 equiv) was dissolved in THF (5.8 mL). Triphenylphosphine (121 mg, 0.458 mmol, 2.0 equiv) and di-*tert*-butyl azodicarboxylate (106 mg, 0.458 mmol, 2.0 equiv) were added, and the mixture was stirred for 2 h at rt. After evaporation of the volatiles, the crude product was purified by reverse-phase HPLC with a gradient 20–70% MeCN/water to give **88** (40 mg, 92 μmol, 40% yield) as a colorless powder. HRMS (ESI) *m*/*z*: calcd for C_21_H_30_N_3_O_7_ [M + H]^+^, 436.2078; found, 436.2068. ^1^H NMR (500 MHz, CDCl_3_): δ 7.64 (dd, *J* = 7.5, 1.5 Hz, 1H), 7.41 (td, *J* = 7.5, 1.5 Hz, 1H), 7.37 (td, *J* = 7.5, 1.3 Hz, 1H), 7.25–7.19 (m, 2H), 5.57 (d, *J* = 7.2 Hz, 1H), 5.52 (dd, *J* = 11.5, 2.1 Hz, 1H), 5.10–5.03 (m, 1H), 5.00 (d, *J* = 10.5 Hz, 1H), 4.43–4.36 (m, 1H), 4.33 (d, *J* = 10.5 Hz, 1H), 4.15–4.10 (m, 1H), 3.85–3.79 (m, 1H), 3.64–3.58 (m, 1H), 3.05 (s, 3H), 2.92 (s, 3H), 1.42 (s, 9H). ^13^C NMR (126 MHz, CDCl_3_): δ 169.0, 168.7, 167.7, 156.3, 136.9, 131.3, 131.3, 130.3, 129.4, 128.3, 80.9, 71.7, 68.9, 64.5, 56.2, 49.6, 37.2, 35.9, 28.2.

#### (4*R*,7*R*)-7-((*S*)-1-Acetylpyrrolidine-2-carboxamido)-*N*,*N*-dimethyl-6,10-dioxo-1,3,4,5,6,7,8,10-octahydrobenzo[*j*][1,8]dioxa[4]azacyclododecine-4-carboxamide (**6**)

Compound **88** (20 mg, 45 μmol, 1.0 equiv) was dissolved in 4 M HCl in 1,4-dioxane (3 mL) and stirred for 1 h at rt. After evaporation of the volatiles under reduced pressure, the resulting salt was dissolved in DMSO (1 mL). Ac-l-Pro-OH (15 mg, 90 μmol, 2.0 equiv), EDC·HCl (17 mg, 90 μmol, 2.0 equiv), and DIPEA (30 μL, 0.18 mmol, 4.0 equiv) were added, and the reaction mixture was stirred for 2 h at rt. The crude product was purified by reverse-phase HPLC with a gradient 15–75% MeCN/water to give **6** (9 mg, 19 μmol, 42% yield) as a colorless powder. HRMS (ESI) *m*/*z*: calcd for C_23_H_31_N_4_O_7_ [M + H]^+^, 475.2187; found, 475.2177. ^1^H NMR (500 MHz, CDCl_3_): δ 8.27 (d, *J* = 7.2 Hz, 1H), 7.76 (d, *J* = 7.1 Hz, 1H), 7.44–7.34 (m, 2H), 7.25–7.23 (m, 1H), 7.21 (d, *J* = 8.3 Hz, 1H), 5.41 (dd, *J* = 11.4, 2.5 Hz, 1H), 5.06–5.01 (m, 1H), 4.99 (d, *J* = 10.2 Hz, 1H), 4.62–4.57 (m, 1H), 4.56–4.51 (m, 1H), 4.38 (d, *J* = 10.2 Hz, 1H), 4.22 (dd, *J* = 11.4, 2.4 Hz, 1H), 3.84 (dd, *J* = 9.8, 3.4 Hz, 1H), 3.76–3.71 (m, 1H), 3.60 (dd, *J* = 9.8, 6.0 Hz, 1H), 3.39–3.32 (m, 1H), 3.01 (s, 3H), 2.88 (s, 3H), 2.43–2.35 (m, 1H), 2.12–2.01 (m, 2H), 1.95 (s, 3H), 1.92–1.81 (m, 2H). ^13^C NMR (126 MHz, CDCl_3_): δ 171.8, 171.7, 168.4, 168.2, 168.1, 137.5, 131.3, 131.2, 130.6, 130.0, 128.3, 71.5, 69.4, 64.8, 59.9, 54.9, 49.5, 48.2, 37.0, 35.7, 27.4, 25.0, 22.2.

#### *tert*-Butyl (*R*)-(1-(Dimethylamino)-1-oxopent-4-en-2-yl)carbamate (**89**)

Boc-d-allylglycine (5.00 g, 23.2 mmol, 1.0 equiv) was dissolved in DMF (45 mL). Dimethylamine hydrochloride (2.81 g, 34.8 mmol, 1.5 equiv), EDC·HCl (6.65 g, 34.8 mmol, 1.5 equiv), HOBt·*x*H_2_O (4.70 g, 34.8 mmol, 1.5 equiv), and DIPEA (6.06 mL, 34.8 mmol, 1.5 equiv) were added, and the mixture was stirred for 2 h at rt. The mixture was diluted with EtOAc (350 mL) and washed with a 1 M aqueous HCl solution (150 mL), saturated aqueous NaHCO_3_ solution (150 mL), and brine (150 mL). The organic phase was dried over MgSO_4_, filtered, and concentrated under reduced pressure, providing **89** as a yellow oil (4.44 g, 18.3 mmol, 79% yield). HRMS (ESI) *m*/*z*: calcd for C_12_H_23_N_2_O_3_ [M + H]^+^, 243.1703; found, 243.1705. ^1^H NMR (400 MHz, CDCl_3_): δ 5.76 (ddt, *J* = 17.2, 10.1, 7.2 Hz, 1H), 5.38 (d, *J* = 7.3, 1H), 5.20–5.00 (m, 2H), 4.78–4.59 (m, 1H), 3.09 (s, 3H), 2.97 (s, 3H), 2.46 (dt, *J* = 13.3, 6.4 Hz, 1H), 2.35 (t, *J* = 7.1 Hz, 1H), 1.43 (s, 9H). ^13^C NMR (101 MHz, CDCl_3_): δ 171.6, 155.3, 132.8, 118.5, 79.5, 49.8, 37.7, 37.2, 35.7, 28.3.

#### Methyl-(*R*,*E*)-2-(4-((*tert*-butoxycarbonyl)amino)-5-(dimethylamino)-5-oxopent-1-en-1-yl)benzoate (**90**)

A 20 mL vial was charged with compound **89** (277 mg, 1.14 mmol, 1.0 equiv), methyl 2-iodobenzoate (360 mg, 1.37 mmol, 1.2 equiv), and Pd(OAc)_2_ (38 mg, 0.17 mmol, 15 mol %). After the vial was evacuated and refilled with argon three times, MeCN (8 mL) and DIPEA (1.00 mL, 5.70 mmol, 5.0 equiv) were added. The vial was sealed, and the reaction mixture was stirred at 85 °C for 16 h in a preheated oil bath. The mixture was allowed to cool to rt and concentrated under reduced pressure. The crude reaction mixture was purified by flash chromatography on a silica gel column using 10–100% EtOAc/hexane as the eluent to give **90** (310 mg, 0.824 mmol, 72% yield) as a yellow oil. HRMS (ESI) *m*/*z*: calcd for C_20_H_29_N_2_O_5_ [M + H]^+^, 377.2071; found, 377.2062. ^1^H NMR (500 MHz, CDCl_3_): δ 7.85 (d, *J* = 9.2 Hz, 1H), 7.52 (d, *J* = 8.0 Hz, 1H), 7.44 (t, *J* = 8.3 Hz, 1H), 7.29–7.25 (m, 1H), 7.21 (d, *J* = 15.2 Hz, 1H), 6.03 (dt, *J* = 15.2, 7.3 Hz, 1H), 5.50 (d, *J* = 8.5 Hz, 1H), 4.79–4.72 (m, 1H), 3.90 (s, 3H), 3.10 (s, 3H), 2.98 (s, 3H), 2.69–2.58 (m, 1H), 2.55–2.47 (m, 1H), 1.41 (s, 9H). ^13^C NMR (126 MHz, CDCl_3_): δ 171.4, 167.8, 155.4, 139.1, 132.2, 132.1, 130.3, 128.2, 127.8, 127.4, 127.0, 79.6, 52.0, 50.1, 37.2, 37.1, 35.8, 28.3.

#### Methyl-2-((*R*,*E*)-4-((*R*)-2-((*tert*-butoxycarbonyl)amino)-3-hydroxypropanamido)-5-(dimethylamino)-5-oxopent-1-en-1-yl)benzoate (**91**)

Compound **90** (300 mg, 0.798 mmol, 1.0 equiv) was dissolved in 4 M HCl in 1,4-dioxane (5 mL) and stirred for 1 h at rt. After evaporation of the volatiles under reduced pressure, the resulting salt was dissolved in MeCN (8 mL). Boc-d-Ser-OH (246 mg, 1.20 mmol, 1.5 equiv), EDC·HCl (229 mg, 1.20 mmol, 1.5 equiv), and DIPEA (210 μL, 1.20 mmol, 1.5 equiv) were added, and the reaction mixture was stirred for 2 h at rt. EtOAc (100 mL) was added, and the mixture was washed with a 1 M aqueous HCl solution (50 mL), saturated aqueous NaHCO_3_ solution (50 mL), and brine (50 mL). The organic phase was dried over MgSO_4_, filtered, and concentrated under reduced pressure. The crude reaction mixture was purified by flash chromatography on a silica gel column using 0–10% MeOH/DCM as the eluent to give **91** (255 mg, 0.558 mmol, 70% yield) as a yellow oil. HRMS (ESI) *m*/*z*: calcd for C_23_H_34_N_3_O_7_ [M + H]^+^, 464.2391; found, 464.2378. ^1^H NMR (600 MHz, CDCl_3_): δ 7.89 (d, *J* = 7.9 Hz, 1H), 7.48–7.42 (m, 2H), 7.31–7.27 (m, 1H), 7.24–7.19 (m, 1H), 7.16 (d, *J* = 15.6 Hz, 1H), 5.91 (dt, *J* = 15.6, 7.6 Hz, 1H), 5.71 (d, *J* = 8.7 Hz, 1H), 5.03 (td, *J* = 7.6, 4.6 Hz, 1H), 4.37–4.28 (m, 1H), 3.99–3.93 (m, 1H), 3.91 (s, 3H), 3.65–3.56 (m, 1H), 3.37 (br s, 1H), 3.13 (s, 3H), 2.98 (s, 3H), 2.73–2.65 (m, 1H), 2.59–2.52 (m, 1H), 1.42 (s, 9H). ^13^C NMR (151 MHz, CDCl_3_): δ 171.1, 170.8, 167.8, 155.8, 139.4, 133.8, 132.4, 130.4, 128.0, 127.8, 127.3, 126.2, 80.0, 63.5, 55.3, 52.3, 49.3, 37.2, 36.0, 35.6, 27.8.

#### 2-((*R*,*E*)-4-((*R*)-2-((*tert*-Butoxycarbonyl)amino)-3-hydroxypropanamido)-5-(dimethylamino)-5-oxopent-1-en-1-yl)benzoic Acid (**92**)

Compound **91** (255 mg, 0.549 mmol, 1.0 equiv) was dissolved in MeOH (5 mL). Subsequently, a 0.25 M aqueous LiOH (15 mL) was added, and the mixture was warmed to 40 °C and stirred for 16 h. The reaction was acidified with a 1 M aqueous HCl solution (5 mL) and extracted with DCM (3 × 50 mL). The combined organic phase was dried over MgSO_4_, filtered, and concentrated under reduced pressure, providing **92** as a colorless solid (180 mg, 0.401 mmol, 73% yield). HRMS (ESI) *m*/*z*: calcd for C_22_H_32_N_3_O_7_ [M + H^+^, ] 450.2235; found, 450.2228. ^1^H NMR (500 MHz, DMSO-*d*_6_): δ 12.95 (br s, 1H), 7.96 (d, *J* = 8.1 Hz, 1H), 7.73 (d, *J* = 7.7 Hz, 1H), 7.52 (d, *J* = 7.5 Hz, 1H), 7.46 (t, *J* = 7.5 Hz, 1H), 7.31 (t, *J* = 7.7 Hz, 1H), 7.12 (d, *J* = 15.2 Hz, 1H), 6.81 (d, *J* = 8.5 Hz, 1H), 6.07 (dt, *J* = 15.2, 7.3 Hz, 1H), 4.85 (ddd, *J* = 8.1, 7.3, 5.5 Hz, 1H), 4.01–3.94 (m, 1H), 3.56–3.45 (m, 2H), 3.01 (s, 3H), 2.83 (s, 3H), 2.54–2.50 (m, 1H), 2.41 (dd, *J* = 14.2, 7.3 Hz, 1H), 1.37 (s, 9H). *The CH*_*2*_*OHproton was not detectable in this spectrum.*^13^C NMR (126 MHz, DMSO-*d*_6_): δ 170.6, 170.3, 169.4, 155.7, 138.1, 132.0, 131.8, 131.1, 130.2, 129.1, 127.3, 127.2, 79.6, 62.3, 57.7, 49.0, 37.0, 36.2, 35.7, 28.6.

#### *tert*-Butyl-((4*R*,7*R*,*E*)-7-(dimethylcarbamoyl)-1,5-dioxo-3,4,5,6,7,8-hexahydro-1*H*-benzo[*j*][1]oxa[5]azacyclododecin-4-yl)carbamate (**93**)

Compound **92** (80 mg, 0.16 mmol, 1.0 equiv) was dissolved in THF (4.1 mL). Triphenylphosphine (84 mg, 0.32 mmol, 2.0 equiv) and di-*tert*-butyl azodicarboxylate (74 mg, 0.32 mmol, 2.0 equiv) were added, and the mixture was stirred for 2 h at rt. After evaporation of the volatiles, the crude product was purified by reverse-phase HPLC with a gradient 15–75% MeCN/water to give **93** (24 mg, 58 μmol, 36% yield) as a colorless powder. HRMS (ESI) *m*/*z*: calcd for C_22_H_30_N_3_O_6_ [M + H]^+^, 432.2129; found, 432.2118. ^1^H NMR (500 MHz, CDCl_3_): δ 7.80 (dd, *J* = 7.7, 1.4 Hz, 1H), 7.48 (td, *J* = 7.7, 1.4 Hz, 1H), 7.39 (d, *J* = 6.5 Hz, 1H), 7.37–7.30 (m, 2H), 6.61 (d, *J* = 15.5 Hz, 1H), 5.64 (ddd, *J* = 15.5, 10.4, 4.9 Hz, 1H), 5.22 (d, *J* = 7.8 Hz, 1H), 5.03–4.93 (m, 1H), 4.80 (dd, *J* = 11.1, 2.0 Hz, 1H), 4.55–4.50 (m, 1H), 4.48 (dd, *J* = 11.1, 2.3 Hz, 1H), 3.20–3.15 (m, 1H), 3.14 (s, 3H), 3.02 (s, 3H), 2.47–2.39 (m, 1H), 1.45 (s, 9H). ^13^C NMR (126 MHz, CDCl_3_): δ 170.2, 168.6, 168.1, 155.0, 139.2, 136.7, 132.4, 129.9, 129.1, 128.2, 127.2, 124.3, 81.0, 66.6, 54.4, 50.0, 37.0, 35.9, 33.1, 28.2.

#### (4*R*,7*R*,*E*)-4-((*S*)-1-Acetylpyrrolidine-2-carboxamido)-*N*,*N*-dimethyl-1,5-dioxo-3,4,5,6,7,8-hexahydro-1*H*-benzo[*j*][1]oxa[5]azacyclododecine-7-carboxamide (**8**)

Compound **93** (24 mg, 58 μmol, 1.0 equiv) was dissolved in 4 M HCl in 1,4-dioxane (3 mL) and stirred for 1 h at rt. After evaporation of the volatiles under reduced pressure, the resulting salt was dissolved in DMSO (1 mL). Ac-l-Pro-OH (18 mg, 0.12 mmol, 2.0 equiv), EDC·HCl (23 mg, 0.12 mmol, 2.0 equiv), and DIPEA (43 μL, 0.24 mmol, 4.0 equiv) were added, and the reaction mixture was stirred for 2 h at rt. The crude product was purified by reverse-phase HPLC with a gradient 25–75% MeCN/water to give **8** (11 mg, 24 μmol, 41% yield) as a colorless powder. HRMS (ESI) *m*/*z*: calcd for C_24_H_31_N_4_O_6_ [M + H]^+^, 471.2238; found, 471.2228. ^1^H NMR (500 MHz, CDCl_3_): δ 8.06 (d, *J* = 8.8 Hz, 1H), 7.94 (dd, *J* = 7.7, 1.4 Hz, 1H), 7.48 (td, *J* = 7.5, 1.4 Hz, 1H), 7.41–7.25 (m, 3H), 6.79 (d, *J* = 15.7 Hz, 1H), 5.68 (ddd, *J* = 15.1, 8.0, 6.2 Hz, 1H), 5.03 (td, *J* = 7.0, 4.6 Hz, 1H), 4.86 (dd, *J* = 10.9, 2.6 Hz, 1H), 4.80 (dt, *J* = 8.8, 2.3 Hz, 1H), 4.54 (dd, *J* = 8.0, 2.3 Hz, 1H), 4.48 (dd, *J* = 10.9, 1.9 Hz, 1H), 3.80–3.72 (m, 1H), 3.46–3.38 (m, 1H), 3.15 (s, 3H), 2.98 (s, 3H), 2.84–2.77 (m, 1H), 2.59–2.52 (m, 1H), 2.45–2.38 (m, 1H), 2.22–2.13 (m, 1H), 2.09 (s, 3H), 2.05–1.95 (m, 1H), 1.91–1.83 (m, 1H). ^13^C NMR (126 MHz, CDCl_3_): δ 172.3, 171.1, 170.4, 168.1, 167.8, 139.7, 136.2, 132.4, 130.8, 128.7, 128.6, 127.2, 124.9, 66.2, 59.7, 53.0, 49.1, 48.3, 37.1, 35.8, 33.7, 27.1, 25.1, 22.3.

#### (4*R*,7*R*)-4-((*S*)-1-Acetylpyrrolidine-2-carboxamido)-*N*,*N*-dimethyl-1,5-dioxo-3,4,5,6,7,8,9,10-octahydro-1*H*-benzo[*j*][1]oxa[5]azacyclododecine-7-carboxamide (**7**)

A round-bottom flask was charged with compound **8** (9 mg, 19 μmol, 1.0 equiv) and Pd/C (10 wt %, 20 mg, 19 μmol Pd, 1.0 equiv). The flask was evacuated and refilled with argon three times, followed by the addition of MeOH (3 mL). A hydrogen atmosphere was introduced *via* purging of the system three times with hydrogen gas in a Parr hydrogenator and maintained at 5 bar. After stirring at rt for 18 h, the reaction mixture was diluted with MeOH (10 mL) and filtered through a 0.45 μm syringe filter. After evaporation of the volatiles under reduced pressure, the crude product was purified by reverse-phase HPLC with a gradient 20–75% MeCN/water to give **7** (5 mg, 10 μmol, 53% yield) as a colorless powder. HRMS (ESI) *m*/*z*: calcd for C_24_H_33_N_4_O_6_ [M + H]^+^, 473.2395; found, 473.2388. ^1^H NMR (500 MHz, CDCl_3_): δ 8.01 (d, *J* = 9.3 Hz, 1H), 7.87 (dd, *J* = 7.8, 1.5 Hz, 1H), 7.44 (td, *J* = 7.8, 1.5 Hz, 1H), 7.33 (d, *J* = 9.2 Hz, 1H), 7.29–7.25 (m, 1H), 7.24 (dd, *J* = 7.8, 1.3 Hz, 1H), 5.15–5.09 (m, 1H), 5.08 (dd, *J* = 11.0, 3.2 Hz, 1H), 4.90 (ddd, *J* = 9.3, 3.1, 1.6 Hz, 1H), 4.55 (dd, *J* = 7.9, 2.6 Hz, 1H), 4.39 (dd, *J* = 11.0, 1.7 Hz, 1H), 3.79–3.73 (m, 1H), 3.52–3.44 (m, 1H), 3.15 (s, 3H), 2.95 (s, 3H), 2.86–2.72 (m, 2H), 2.46–2.40 (m, 1H), 2.32–2.23 (m, 1H), 2.20 (s, 3H), 2.08–1.99 (m, 1H), 1.98–1.89 (m, 3H), 1.89–1.80 (m, 1H), 1.71–1.65 (m, 1H). ^13^C NMR (126 MHz, CDCl_3_): δ 172.4, 171.3, 170.9, 168.9, 168.4, 142.8, 132.2, 131.6, 131.4, 129.5, 126.1, 67.5, 59.7, 53.0, 48.4, 47.9, 37.2, 35.8, 35.4, 30.3, 28.3, 27.1, 25.3, 22.6.

#### 2,2,2-Trichloroethyl 2-Bromo-6-methylbenzoate (**94a**)

2-Bromo-6-methylbenzoic acid (5.00 g, 23.3 mmol, 1.0 equiv) was dissolved in 2,2,2-trichloroethanol (15 mL). Concentrated sulfuric acid (0.1 mL) was added, and the reaction mixture was stirred for 16 h at 120 °C in a preheated oil bath. The reaction mixture was allowed to cool down to rt, diluted with EtOAc (250 mL), and washed with a saturated aqueous NaHCO_3_ solution (50 mL). The organic phase was dried over MgSO_4_, filtered, and concentrated under reduced pressure, providing **94a** (4.88 g, 14.2 mmol, 61% yield) as a pale yellow oil. HRMS (ESI) *m*/*z*: calcd for C_10_H_7_BrCl_3_O_2_ [M – H]^−^, 342.8690; found, 342.8692. ^1^H NMR (400 MHz, CDCl_3_): δ 7.46–7.41 (m, 1H), 7.23–7.17 (m, 2H), 5.00 (s, 2H), 2.41 (s, 3H). ^13^C NMR (101 MHz, CDCl_3_): δ 166.3, 137.4, 134.4, 131.0, 130.1, 129.1, 119.3, 94.4, 75.0, 19.9.

#### 2,2,2-Trichloroethyl 2-Bromo-6-(bromomethyl)benzoate (**95a**)

Compound **94a** (4.52 g, 13.1 mmol, 1.0 equiv) was dissolved in chlorobenzene (20 mL). *N*-Bromosuccinimide (2.33 g, 13.1 mmol, 1.0 equiv) and 2,2́-azobis(2-methylpropionitrile) (AIBN) (107 mg, 0.65 mmol, 0.05 equiv) were added, and the mixture was stirred for 16 h at 70 °C. The reaction mixture was allowed to cool down to rt, the solvent was removed under reduced pressure, and the crude product was purified using flash chromatography on a silica gel column with 1–10% EtOAc/hexane as the eluent to give **95a** (4.25 g, 10.1 mmol, 77% yield) as a brown oil. HRMS (ESI) *m*/*z*: calcd for C_10_H_7_Br_2_Cl_3_O_2_ 421.7878, molecular ion peak not found at the HRMS analysis, possibly due to the instability of the compound. ^1^H NMR (400 MHz, CDCl_3_): δ 7.58 (d, *J* = 7.9 Hz, 1H), 7.41 (d, *J* = 7.9 Hz, 1H), 7.30 (t, *J* = 7.9 Hz, 1H), 5.03 (s, 2H), 4.55 (s, 2H). ^13^C NMR (101 MHz, CDCl_3_): δ 165.2, 137.5, 133.9, 133.2, 131.6, 129.3, 120.4, 94.1, 75.5, 29.3.

#### 2,2,2-Trichloroethyl-(*S*)-2-bromo-6-(((2-((*tert*-butoxycarbonyl)amino)-3-methoxy-3-oxopropyl)thio)methyl)benzoate (**96a**)

Compound **95a** (1.21 g, 2.84 mmol, 1.0 equiv) was dissolved in DMSO (20 mL). H-Cys-d-OMe·HCl (584 mg, 3.41 mmol, 1.2 equiv) and triethylamine (885 μL, 6.25 mmol, 2.2 equiv) were added, and the mixture was stirred for 2 h at rt. The mixture was diluted with EtOAc (200 mL) and washed with a saturated aqueous NaHCO_3_ solution (2 × 100 mL) and brine (100 mL). The organic phase was dried over MgSO_4_, filtered, and concentrated under reduced pressure. The crude product was purified using flash chromatography on a silica gel column with 1–5% MeOH/DCM as the eluent to give **96a** (1.08 g, 2.27 mmol, 80% yield) as a yellow oil. HRMS (ESI) *m*/*z*: calcd for C_14_H_16_BrCl_3_NO_4_S [M + H]^+^, 477.9049; found, 477.9060. ^1^H NMR (400 MHz, CDCl_3_): δ 7.54 (d, *J* = 7.9 Hz, 1H), 7.38 (d, *J* = 7.9 Hz, 1H), 7.28 (t, *J* = 7.9 Hz, 1H), 5.03–4.95 (m, 2H), 3.90–3.82 (m, 2H), 3.72 (s, 3H), 3.62 (dd, *J* = 7.2, 4.8 Hz, 1H), 2.83 (dd, *J* = 13.6, 4.8 Hz, 1H), 2.68 (dd, *J* = 13.6, 7.2 Hz, 1H), 1.75 (s, 2H). ^13^C NMR (101 MHz, CDCl_3_): δ 173.9, 165.8, 138.3, 133.9, 132.1, 131.2, 129.1, 120.4, 94.2, 54.1, 52.4, 50.9, 36.1, 34.5.

#### 2,2,2-Trichloroethyl-2-bromo-6-((4*S*,7*R*)-7-(hydroxymethyl)-4-(methoxycarbonyl)-11,11-dimethyl-6,9-dioxo-10-oxa-2-thia-5,8-diazadodecyl)benzoate (**97a**)

Compound **96a** (1.08 g, 2.27 mmol, 1.0 equiv) was dissolved in MeCN (20 mL). Boc-d-Ser-OH (698 mg, 3.41 mmol, 1.5 equiv) and EDC·HCl (651 mg, 3.41 mmol, 1.5 equiv) were added, and the reaction mixture was stirred for 1 h at rt. EtOAc (150 mL) was added, and the mixture was washed with a 1 M aqueous HCl solution (50 mL), saturated aqueous NaHCO_3_ solution (50 mL), and brine (100 mL). The organic phase was dried over MgSO_4_, filtered, and concentrated under reduced pressure. The crude product was purified using flash chromatography on a silica gel column with 1–10% MeOH/DCM as the eluent to give **97a** (1.17 g, 1.77 mmol, 78% yield) as a yellow oil. HRMS (ESI) *m*/*z*: calcd for C_22_H_29_BrCl_3_N_2_O_8_S [M + H]^+^, 664.9894; found, 664.9915. ^1^H NMR (500 MHz, CDCl_3_): δ 7.48 (d, *J* = 7.9 Hz, 1H), 7.27 (d, *J* = 7.9 Hz, 1H), 7.23–7.17 (m, 2H), 5.54 (d, *J* = 8.0 Hz, 1H), 4.98–4.89 (m, 2H), 4.71–4.65 (m, 1H), 4.20–4.12 (m, 1H), 4.04–3.98 (m, 1H), 3.81 (d, *J* = 13.8 Hz, 1H), 3.75 (d, *J* = 13.8 Hz, 1H), 3.67 (s, 3H), 3.64–3.59 (m, 1H), 2.86 (dd, *J* = 14.0, 4.7 Hz, 1H), 2.72 (dd, *J* = 14.0, 6.7 Hz, 1H), 1.39 (s, 9H). *The CH*_*2*_*OHproton was not detectable in this spectrum.*^13^C NMR (126 MHz, CDCl_3_): δ 171.3, 170.8, 165.8, 155.8, 137.8, 133.8, 132.2, 131.3, 129.2, 120.6, 94.2, 80.5, 75.6, 63.0, 55.2, 52.9, 51.7, 34.4, 33.3, 28.3.

#### 2-Bromo-6-((4*S*,7*R*)-7-(hydroxymethyl)-4-(methoxycarbonyl)-11,11-dimethyl-6,9-dioxo-10-oxa-2-thia-5,8-diazadodecyl)benzoic Acid (**98a**)

Compound **97a** (1.05 g, 1.57 mmol, 1.0 equiv) was dissolved in THF (20 mL). Aqueous 1 M NH_4_OAc (4 mL) and Zn dust (2.06 g, 31.4 mmol, 20 equiv) were added, and the reaction was vigorously stirred for 2 h at rt. The mixture was diluted with MeOH (300 mL), filtered through a pad of Celite, and concentrated under reduced pressure. The crude product was purified using flash chromatography on a silica gel column with 70–90% EtOAc/hexane as the eluent to give **98a** (610 mg, 1.14 mmol, 73% yield) as a colorless powder. HRMS (ESI) *m*/*z*: calcd for C_20_H_28_BrN_2_O_8_S [M + H]^+^, 535.0750; found, 535.0753. ^1^H NMR (500 MHz, DMSO-*d*_6_): δ 8.40 (d, *J* = 6.6 Hz, 1H), 7.43 (d, *J* = 7.9 Hz, 1H), 7.33 (d, *J* = 7.9 Hz, 1H), 7.19–7.10 (m, 2H), 4.58–4.51 (m, 1H), 4.13–4.08 (m, 1H), 3.84 (d, *J* = 13.5 Hz, 1H), 3.74 (d, *J* = 13.5 Hz, 1H), 3.64–3.55 (m, 2H), 3.61 (s, 3H), 2.84–2.76 (m, 2H), 1.40 (s, 9H). *The COOHand CH*_*2*_*OHprotons were not detectable in this spectrum.*^13^C NMR (126 MHz, DMSO-*d*_6_): δ 171.3, 171.3, 169.9, 155.7, 136.5, 131.0, 129.1, 129.1, 128.8, 118.1, 78.6, 62.5, 57.6, 53.1, 52.6, 34.1, 32.5, 28.7.

#### Methyl-(4*S*,7*R*)-11-bromo-7-((*tert*-butoxycarbonyl)amino)-6,10-dioxo-1,3,4,5,6,7,8,10-octahydrobenzo[*j*][1]oxa[8]thia[5]azacyclododecine-4-carboxylate (**99a**)

Compound **98a** (590 mg, 1.10 mmol, 1.0 equiv) was dissolved in toluene (28 mL). Triphenylphosphine (432 mg, 1.65 mmol, 1.5 equiv) and di-*tert*-butyl azodicarboxylate (380 mg, 1.65 mmol, 1.5 equiv) were added, and the mixture was stirred for 4 h at rt. After evaporation of the volatiles under reduced pressure, the crude product was purified using flash chromatography on a silica gel column with 45% EtOAc/hexane as the eluent to give **99a** (210 mg, 0.406 mmol, 37% yield) as a colorless powder. HRMS (ESI) *m*/*z*: calcd for C_20_H_26_BrN_2_O_7_S [M + H]^+^, 517.0644; found, 517.0642. ^1^H NMR (500 MHz, CDCl_3_): δ 7.52 (d, *J* = 7.9 Hz, 1H), 7.35 (d, *J* = 7.9 Hz, 1H), 7.34–7.30 (m, 1H), 7.28–7.24 (m, 1H), 5.76–5.68 (m, 1H), 5.34–5.28 (m, 1H), 4.91–4.82 (m, 1H), 4.66–4.58 (m, 1H), 4.42–4.36 (m, 1H), 3.99 (d, *J* = 12.0 Hz, 1H), 3.79 (s, 3H), 3.49 (d, *J* = 12.0 Hz, 1H), 3.33–3.23 (m, 1H), 3.17–3.06 (m, 1H), 1.52 (s, 9H). ^13^C NMR (126 MHz, CDCl_3_): δ 170.3, 169.0, 167.0, 155.0, 136.6, 134.6, 131.8, 131.2, 128.7, 119.6, 81.3, 66.7, 55.3, 53.0, 52.5, 35.7, 34.1, 28.2.

#### *tert*-Butyl-((4*S*,7*R*)-11-bromo-4-(dimethylcarbamoyl)-6,10-dioxo-1,3,4,5,6,7,8,10-octahydrobenzo[*j*][1]oxa[8]thia[5]azacyclododecin-7-yl)carbamate (**100a**)

Compound **99a** (130 mg, 0.252 mmol, 1.0 equiv) was dissolved in 1,2-dichloroethane (10 mL). Me_3_SnOH (180 mg, 1.00 mmol, 4.0 equiv) was added, and the mixture was stirred for 45 min at 85 °C. The reaction was allowed to cool down to rt, acidified with 1 M aqueous HCl solution (10 mL), and extracted with DCM (3 × 50 mL). The combined organic phases were dried over MgSO_4_, filtered, and concentrated under reduced pressure, and the resulting crude acid was dissolved in DMF (5 mL). HOBt·*x*H_2_O (68 mg, 0.50 mmol, 2.0 equiv), EDC·HCl (96 mg, 0.50 mmol, 2.0 equiv), and dimethylamine (2 M in THF, 190 μL, 0.38 mmol, 1.5 equiv) were added, and the mixture was stirred for 2 h at rt. The mixture was diluted with EtOAc (75 mL) and washed with a 1 M aqueous HCl solution (50 mL), saturated aqueous NaHCO_3_ solution (50 mL), and brine (50 mL). The organic phase was dried over MgSO_4_, filtered, and concentrated under reduced pressure. The crude product was purified using reverse-phase HPLC with a gradient 25% to 75% MeCN in water to give **100a** (46 mg, 87 μmol, 35% yield over two steps) as a colorless powder. HRMS (ESI) *m*/*z*: calcd for C_21_H_29_BrN_3_O_6_S [M + H]^+^, 530.0960; found, 530.0957. ^1^H NMR (500 MHz, CDCl_3_): δ 7.43 (d, *J* = 7.9 Hz, 1H), 7.24 (d, *J* = 7.9 Hz, 1H), 7.18–7.12 (m, 2H), 5.61–5.53 (m, 1H), 5.38–5.33 (d, *J* = 10.5 Hz, 1H), 5.10–5.05 (m, 1H), 4.59–4.51 (m, 1H), 4.22–4.13 (m, 1H), 3.74 (d, *J* = 11.7 Hz, 1H), 3.53 (d, *J* = 11.7 Hz, 1H), 3.00 (s, 3H), 2.99–2.94 (m, 1H), 2.88 (s, 3H), 2.83–2.74 (m, 1H), 1.40 (s, 9H). ^13^C NMR (126 MHz, CDCl_3_): δ 169.3, 169.0, 166.9, 155.1, 136.3, 134.9, 131.8, 131.0, 129.1, 119.7, 81.3, 66.4, 55.4, 48.8, 37.1, 36.0, 35.9, 34.8, 28.2.

#### (4*S*,7*R*)-7-((*S*)-1-Acetylpyrrolidine-2-carboxamido)-11-bromo-*N*,*N*-dimethyl-6,10-dioxo-1,3,4,5,6,7,8,10-octahydrobenzo[*j*][1]oxa[8]thia[5]azacyclododecine-4-carboxamide (**9**)

Compound **100a** (46 mg, 87 μmol, 1.0 equiv) was dissolved in 4 M HCl in 1,4-dioxane (4 mL) and stirred for 1 h at rt. After evaporation of the volatiles under reduced pressure, the resulting salt was dissolved in DMSO (3 mL). Ac-l-Pro-OH (26 mg, 0.17 mmol, 2.0 equiv), EDC·HCl (32 mg, 0.17 mmol, 2.0 equiv), and DIPEA (45 μL, 0.26 mmol, 3.0 equiv) were added, and the reaction mixture was stirred for 2 h at rt. The crude product was purified using reverse-phase HPLC with a gradient 20–75% MeCN/water in water to give **9** (18 mg, 32 μmol, 37% yield over two steps) as a colorless powder. HRMS (ESI) *m*/*z*: calcd for C_23_H_29_BrN_4_NaO_6_S [M + Na]^+^, 591.0889; found, 591.0878. ^1^H NMR (500 MHz, CDCl_3_): δ 7.92 (d, *J* = 9.3 Hz, 1H), 7.86 (d, *J* = 8.5 Hz, 1H), 7.50 (d, *J* = 7.9 Hz, 1H), 7.37 (d, *J* = 7.9 Hz, 1H), 7.25 (t, *J* = 7.9 Hz, 1H), 5.45 (dd, *J* = 11.4, 3.3 Hz, 1H), 5.15–5.06 (m, 1H), 4.94–4.88 (m, 1H), 4.48 (dd, *J* = 11.3, 2.0 Hz, 1H), 4.36 (dd, *J* = 7.6, 4.1 Hz, 1H), 3.84–3.78 (m, 2H), 3.73–3.65 (m, 1H), 3.58–3.48 (m, 1H), 3.10–3.07 (m, 1H), 3.06 (s, 3H), 3.06–3.03 (m, 1H), 2.93 (s, 3H), 2.33–2.22 (m, 2H), 2.11 (s, 3H), 2.10–2.06 (m, 1H), 2.03–1.95 (m, 1H). ^13^C NMR (126 MHz, CDCl_3_): δ 172.0, 171.3, 169.2, 168.2, 168.0, 136.1, 135.3, 131.6, 131.0, 129.5, 119.4, 66.3, 60.6, 54.6, 49.8, 48.3, 37.1, 36.3, 35.9, 34.9, 28.4, 25.4, 22.5.

#### 2,2,2-Trichloroethyl 5-Bromo-2-methylbenzoate (**94b**)

5-Bromo-6-methylbenzoic acid (5.00 g, 23.3 mmol, 1.0 equiv) was dissolved in DCM (50 mL). EDC·HCl (6.65 g, 34.9 mmol, 1.5 equiv), 4-dimethylaminopyridine (DMAP) (284 mg, 2.33 mmol, 0.1 equiv), and 2,2,2-trichloroethanol (2.90 mL, 30.2 mmol, 1.3 equiv) were added, and the mixture was stirred for 16 h at rt. The mixture was diluted with DCM (150 mL) and washed with a 1 M aqueous HCl solution (2 × 100 mL) and saturated aqueous NaHCO_3_ solution (2 × 100 mL). The organic phase was dried over MgSO_4_, filtered, and concentrated under reduced pressure, providing **94b** as a brown oil (6.72 g, 19.5 mmol, 84% yield). HRMS (ESI) *m*/*z*: calcd for C_10_H_7_BrCl_3_O_2_ [M – H]^−^, 342.8690; found, 342.8691. ^1^H NMR (400 MHz, CDCl_3_): δ 8.15 (d, *J* = 2.2 Hz, 1H), 7.57 (dd, *J* = 8.2, 2.2 Hz, 1H), 7.17 (d, *J* = 8.2 Hz, 1H), 4.96 (s, 2H), 2.60 (s, 3H). ^13^C NMR (101 MHz, CDCl_3_): δ 164.4, 140.0, 135.8, 133.8, 133.5, 129.6, 119.4, 94.8, 74.5, 21.5.

#### 2,2,2-Trichloroethyl 5-Bromo-2-(bromomethyl)benzoate (**95b**)

Compound **94b** (4.07 g, 11.8 mmol, 1.0 equiv) was dissolved in chlorobenzene (20 mL). *N*-Bromosuccinimide (2.09 g, 11.8 mmol, 1.0 equiv) and AIBN (96 mg, 0.59 mmol, 0.05 equiv) were added, and the mixture was stirred for 16 h at 70 °C. After evaporation of the volatiles under reduced pressure, the crude product was purified using flash chromatography on a silica gel column with 1–10% EtOAc/hexane as the eluent to give **95b** (3.96 g, 9.41 mmol, 80% yield) as a brown oil. HRMS (ESI) *m*/*z*: calcd for C_10_H_6_Br_2_Cl_3_O_2_ [M – H]^−^, 420.7800; found, 420.7822. ^1^H NMR (400 MHz, CDCl_3_): δ 8.20 (d, *J* = 2.2 Hz, 1H), 7.68 (dd, *J* = 8.3, 2.2 Hz, 1H), 7.38 (d, *J* = 8.3 Hz, 1H), 5.00 (s, 2H), 4.91 (s, 2H). ^13^C NMR (101 MHz, CDCl_3_): δ 163.4, 139.0, 136.4, 134.6, 133.4, 129.0, 122.6, 94.6, 74.8, 30.1.

#### 2,2,2-Trichloroethyl-(*S*)-2-(((2-amino-3-methoxy-3-oxopropyl)thio)methyl)-5-bromobenzoate (**96b**)

Compound **95b** (3.52 g, 8.28 mmol, 1.0 equiv) was dissolved in DMSO (35 mL). H-Cys-d-OMe·HCl (1.70 g, 9.94 mmol, 1.2 equiv) and triethylamine (2.58 mL, 18.2 mmol, 2.2 equiv) were added, and the mixture was stirred for 2 h at rt. The mixture was diluted with EtOAc (250 mL) and washed with a saturated aqueous NaHCO_3_ solution (2 × 100 mL) and brine (100 mL). The organic phase was dried over MgSO_4_, filtered, and concentrated under reduced pressure. The crude reaction mixture was purified using flash chromatography on a silica gel column with 0–5% MeOH/DCM as the eluent to give **96b** (2.92 g, 6.13 mmol, 74% yield) as a yellow oil. HRMS (ESI) *m*/*z*: calcd for C_14_H_16_BrCl_3_NO_4_S [M + H]^+^, 477.9049; found, 477.9032. ^1^H NMR (500 MHz, CDCl_3_): δ 8.18 (d, *J* = 2.0 Hz, 1H), 7.66 (dd, *J* = 8.2, 2.0 Hz, 1H), 7.32 (d, *J* = 8.2 Hz, 1H), 5.00–4.95 (m, 2H), 4.19 (d, *J* = 13.4 Hz, 1H), 4.13 (d, *J* = 13.4 Hz, 1H), 3.74 (s, 3H), 3.68 (dd, *J* = 7.5, 4.7 Hz, 1H), 2.87 (dd, *J* = 13.6, 4.7 Hz, 1H), 2.71 (dd, *J* = 13.6, 7.5 Hz, 1H), 2.08 (s, 2H). ^13^C NMR (126 MHz, CDCl_3_): δ 174.1, 164.1, 140.0, 135.8, 134.5, 132.9, 129.5, 121.2, 94.7, 74.7, 54.2, 52.4, 36.7, 34.3.

#### 2,2,2-Trichloroethyl-2-((4*S*,7*R*)-7-(hydroxymethyl)-4-(methoxycarbonyl)-11,11-dimethyl-6,9-dioxo-10-oxa-2-thia-5,8-diazadodecyl)benzoate (**97b**)

Compound **96b** (5.02 g, 10.5 mmol, 1.0 equiv) was dissolved in MeCN (50 mL). Boc-d-Ser-OH (3.22 g, 15.8 mmol, 1.5 equiv) and EDC·HCl (3.01 g, 15.8 mmol, 1.5 equiv) were added, and the reaction mixture was stirred for 1 h at rt. EtOAc (300 mL) was added, and the mixture was washed with a 1 M aqueous HCl solution (150 mL), saturated aqueous NaHCO_3_ solution (150 mL), and brine (100 mL). The organic phase was dried over MgSO_4_, filtered, and concentrated under reduced pressure. The crude reaction mixture was purified using flash chromatography on a silica gel column with 5% MeOH/DCM as the eluent to give **97b** (5.08 g, 7.65 mmol, 73% yield) as a yellow oil. HRMS (ESI) *m*/*z*: calcd for C_22_H_28_BrCl_3_N_2_NaO_8_S [M + Na]^+^, 686.9713; found, 686.9710. ^1^H NMR (400 MHz, DMSO-*d*_6_): δ 8.26 (d, *J* = 7.9 Hz, 1H), 8.00 (d, *J* = 2.0 Hz, 1H), 7.82 (dd, *J* = 8.3, 2.0 Hz, 1H), 7.44 (d, *J* = 8.3 Hz, 1H), 6.65 (d, *J* = 7.9 Hz, 1H), 5.13–5.08 (m, 2H), 4.81–4.75 (m, 1H), 4.54–4.43 (m, 1H), 4.11–4.05 (m, 2H), 4.04–3.98 (m, 1H), 3.59 (s, 3H), 3.57–3.42 (m, 2H), 2.75 (dd, *J* = 13.9, 5.6 Hz, 1H), 2.66 (dd, *J* = 13.9, 7.2 Hz, 1H), 1.36 (s, 9H). ^13^C NMR (126 MHz, CDCl_3_): δ 171.3, 170.8, 164.1, 155.9, 139.5, 135.9, 134.5, 133.0, 129.4, 121.4, 94.7, 80.6, 74.7, 63.1, 55.2, 52.9, 52.0, 34.3, 33.6, 28.3.

#### 5-Bromo-2-((4*S*,7*R*)-7-(hydroxymethyl)-4-(methoxycarbonyl)-11,11-dimethyl-6,9-dioxo-10-oxa-2-thia-5,8-diazadodecyl)benzoic Acid (**98b**)

Compound **97b** (5.00 g, 7.51 mmol, 1.0 equiv) was dissolved in THF (50 mL). Aqueous 1 M NH_4_OAc (5 mL) and Zn dust (4.88 g, 75.1 mmol, 10 equiv) were added, and the reaction was vigorously stirred for 2 h at rt. The mixture was diluted with MeOH (500 mL), filtered through a pad of Celite, and concentrated under reduced pressure. The crude reaction mixture was purified using flash chromatography on a silica gel column with 1–10% MeOH/DCM as the eluent to give **98b** (2.80 g, 5.24 mmol, 69% yield) as a colorless powder. HRMS (ESI) *m*/*z*: calcd for C_20_H_28_BrN_2_O_8_S [M + H]^+^, 535.0750; found, 535.0745. ^1^H NMR (500 MHz, DMSO-*d*_6_): δ 13.36 (br s, 1H), 8.31–8.24 (m, 1H), 7.95 (d, *J* = 2.2 Hz, 1H), 7.72–7.68 (m, 1H), 7.36 (dd, *J* = 8.3, 2.2 Hz, 1H), 6.71–6.62 (m, 1H), 4.85–4.74 (m, 1H), 4.54–4.42 (m, 1H), 4.10–4.06 (m, 2H), 4.06–4.01 (m, 1H), 3.62 (s, 3H), 3.60–3.43 (m, 2H), 2.77 (dd, *J* = 13.8, 5.8 Hz, 1H), 2.68 (dd, *J* = 13.8, 7.3 Hz, 1H), 1.38 (s, 9H). ^13^C NMR (126 MHz, DMSO-*d*_6_): δ 171.3, 171.0, 167.5, 155.7, 139.8, 134.7, 133.7, 133.6, 132.7, 120.3, 79.7, 78.7, 62.2, 57.2, 52.6, 33.5, 32.7, 28.6.

#### Methyl-(4*S*,7*R*)-12-bromo-7-((*tert*-butoxycarbonyl)amino)-6,10-dioxo-1,3,4,5,6,7,8,10-octahydrobenzo[*j*][1]oxa[8]thia[5]azacyclododecine-4-carboxylate (**99b**)

Compound **98b** (2.68 g, 5.01 mmol, 1.0 equiv) was dissolved in toluene (125 mL). Triphenylphosphine (1.97 g, 7.52 mmol, 1.5 equiv) and di-*tert*-butyl azodicarboxylate (1.73 g, 7.52 mmol, 1.5 equiv) were added, and the mixture was stirred for 4 h at rt. After evaporation of the volatiles under reduced pressure, the crude product was purified using flash chromatography on a silica gel column with 50% EtOAc/hexane as the eluent to give **99b** (1.55 g, 3.01 mmol, 60% yield) as a colorless powder. HRMS (ESI) *m*/*z*: calcd for C_20_H_25_BrN_2_NaO_7_S [M + Na]^+^, 539.0464; found, 539.0470. ^1^H NMR (400 MHz, acetone-*d*_6_): δ 8.04–7.96 (m, 1H), 7.84–7.73 (m, 1H), 7.70 (dd, *J* = 8.2, 2.0 Hz, 1H), 7.40 (d, *J* = 8.2 Hz, 1H), 6.95–6.84 (m, 1H), 5.02–4.91 (m, 1H), 4.91–4.82 (m, 1H), 4.57–4.51 (m, 3H), 3.86 (d, *J* = 10.6 Hz, 1H), 3.71 (s, 3H), 3.20 (dd, *J* = 14.6, 4.2 Hz, 1H), 3.04 (dd, *J* = 14.6, 8.8 Hz, 1H), 1.44 (s, 9H). ^13^C NMR (126 MHz, CDCl_3_): δ 170.4, 169.1, 167.1, 155.0, 136.5, 135.4, 134.1, 133.0, 131.6, 121.4, 81.3, 67.0, 55.3, 52.9, 52.2, 35.7, 35.0, 28.2.

#### *tert*-Butyl-((4*S*,7*R*)-12-bromo-4-(dimethylcarbamoyl)-6,10-dioxo-1,3,4,5,6,7,8,10-octahydrobenzo[*j*][1]oxa[8]thia[5]azacyclododecin-7-yl)carbamate (**100b**)

Compound **99b** (191 mg, 0.37 mmol, 1.0 equiv) was dissolved in 1,2-dichloroethane (8 mL). Me_3_SnOH (278 mg, 1.48 mmol, 4.0 equiv) was added, and the mixture was stirred for 45 min at 85 °C. The reaction mixture was allowed to cool to rt, acidified with 1 M aqueous HCl solution (10 mL), and extracted with DCM (3 × 50 mL). The combined organic phases were dried over MgSO_4_, filtered, and concentrated under reduced pressure, and the resulting crude acid was dissolved in DMF (7 mL). HOBt·*x*H_2_O (75 mg, 0.55 mmol, 1.5 equiv), EDC·HCl (105 mg, 0.55 mmol, 1.5 equiv), and dimethylamine (2 M in THF, 280 μL, 0.56 mmol, 1.5 equiv) were added, and the mixture was stirred for 2 h at rt. The mixture was diluted with EtOAc (100 mL) and washed with a 1 M aqueous HCl solution (50 mL), saturated aqueous NaHCO_3_ (50 mL), and brine (50 mL). The organic phase was dried over MgSO_4_, filtered, and concentrated under reduced pressure. The crude product was purified using reverse-phase HPLC with a gradient of 30–85% MeCN/water to give **100b** (45 mg, 85 μmol, 23% yield over two steps) as a colorless powder. HRMS (ESI) *m*/*z*: calcd for C_21_H_29_BrN_3_O_6_S [M + H]^+^, 530.0960; found, 530.0958. ^1^H NMR (500 MHz, CDCl_3_): δ 7.95–7.88 (m, 1H), 7.49 (dd, *J* = 8.2, 2.1 Hz, 1H), 7.36 (d, *J* = 8.5 Hz, 1H), 7.15 (d, *J* = 8.2 Hz, 1H), 5.57–5.49 (m, 1H), 5.16–5.08 (m, 1H), 5.00 (dd, *J* = 11.2, 2.4 Hz, 1H), 4.56–4.50 (m, 1H), 4.41 (dd, *J* = 11.2, 2.1 Hz, 1H), 4.08–3.97 (m, 1H), 3.89–3.81 (m, 1H), 3.02 (s, 3H), 3.00–2.89 (m, 2H), 2.87 (s, 3H), 1.38 (s, 9H). ^13^C NMR (126 MHz, CDCl_3_): δ 169.4, 169.1, 166.7, 155.1, 136.2, 135.4, 134.3, 133.4, 131.6, 121.4, 81.3, 67.3, 55.0, 48.9, 37.1, 37.1, 35.9, 35.6, 28.2.

#### (4*S*,7*R*)-7-((*S*)-1-Acetylpyrrolidine-2-carboxamido)-12-bromo-*N*,*N*-dimethyl-6,10-dioxo-1,3,4,5,6,7,8,10-octahydrobenzo[*j*][1]oxa[8]thia[5]azacyclododecine-4-carboxamide (**10**)

Compound **100b** (41 mg, 77 μmol, 1.0 equiv) was dissolved in 4 M HCl in 1,4-dioxane (4 mL) and stirred for 1 h at rt. After evaporation of the volatiles under reduced pressure, the resulting salt was dissolved in DMSO (3 mL). Ac-l-Pro-OH (24 mg, 0.15 mmol, 2.0 equiv), EDC·HCl (29 mg, 0.15 mmol, 2.0 equiv), and DIPEA (40 μL, 0.23 mmol, 3.0 equiv) were added, and the reaction mixture was stirred for 2 h at rt. The crude product was purified using reverse-phase HPLC with a gradient 25–75% MeCN/water to give **10** (13 mg, 23 μmol, 30% yield over two steps) as a colorless powder. HRMS (ESI) *m*/*z*: calcd for C_23_H_29_BrN_4_NaO_6_S [M + Na]^+^, 591.0889; found, 591.0902. ^1^H NMR (400 MHz, CDCl_3_): δ 8.09 (d, *J* = 2.1 Hz, 1H), 7.94 (d, *J* = 9.2 Hz, 1H), 7.60–7.54 (m, 2H), 7.26 (d, *J* = 8.1 Hz, 1H), 5.15 (dd, *J* = 11.1, 2.6 Hz, 1H), 5.14–5.09 (m, 1H), 4.86 (dt, *J* = 9.2, 2.6 Hz, 1H), 4.51–4.45 (m, 1H), 4.45–4.39 (m, 2H), 3.84 (d, *J* = 10.0 Hz, 1H), 3.77–3.69 (m, 1H), 3.51–3.42 (m, 1H), 3.05 (s, 3H), 3.03–2.94 (m, 2H), 2.91 (s, 3H), 2.42–2.33 (m, 1H), 2.29–2.17 (m, 1H), 2.15 (s, 3H), 2.04–1.89 (m, 2H). ^13^C NMR (126 MHz, CDCl_3_): δ 172.1, 171.4, 169.3, 168.6, 166.3, 136.2, 135.6, 134.8, 134.0, 131.4, 121.4, 67.2, 60.0, 53.1, 49.9, 48.4, 37.8, 37.1, 35.8, 35.8, 27.5, 25.3, 22.6.

#### 2,2,2-Trichloroethyl 4-Bromo-2-methylbenzoate (**101a**)

4-Bromo-2-methylbenzoic acid (4.42 g, 20.6 mmol, 1.0 equiv) was dissolved in DCM (45 mL). EDC·HCl (5.86 g, 30.8 mmol, 1.5 equiv), DMAP (250 mg, 2.06 mmol, 0.1 equiv), and 2,2,2-trichloroethanol (2.56 mL, 26.7, 1.3 equiv) were added, and the mixture was stirred at rt for 16 h. The mixture was diluted with DCM (150 mL) and washed with a 1 M aqueous HCl solution (2 × 100 mL) and saturated aqueous NaHCO_3_ solution (2 × 100 mL). The organic phase was dried over MgSO_4_, filtered, and concentrated under reduced pressure, providing **101a** as a brown oil (5.79 g, 16.9 mmol, 82% yield). HRMS (ESI) *m*/*z*: calcd for C_10_H_8_BrCl_3_O_2_ 343.8773, molecular ion peak not found in the HRMS analysis possibly due to the instability of the compound. ^1^H NMR (400 MHz, CDCl_3_): δ 7.93 (d, *J* = 8.4 Hz, 1H), 7.49–7.46 (m, 1H), 7.44 (dd, *J* = 8.4, 1.7 Hz, 1H), 4.95 (s, 2H), 2.64 (s, 3H). ^13^C NMR (101 MHz, CDCl_3_): δ 164.9, 143.2, 134.8, 132.7, 129.3, 127.9, 126.7, 94.9, 74.4, 21.9.

#### 2,2,2-Trichloroethyl 4-Bromo-2-(bromomethyl)benzoate (**102a**)

Compound **101a** (4.98 g, 14.4 mmol, 1.0 equiv) was dissolved in chlorobenzene (20 mL). *N*-Bromosuccinimide (2.54 g, 14.4 mmol, 1.0 equiv) and AIBN (118 mg, 0.719 mmol, 0.05 equiv) were added, and the mixture was stirred for 16 h at 70 °C. After evaporation of the volatiles under reduced pressure, the crude product was purified using flash chromatography on a silica gel column with 1–5% EtOAc/hexane as the eluent to give **102a** (4.85 g, 11.5 mmol, 80% yield) as a yellow oil. HRMS (ESI) *m*/*z*: calcd for C_10_H_6_Br_2_Cl_3_O_2_ [M – H]^−^, 420.7800; found, 420.7817. ^1^H NMR (400 MHz, CDCl_3_): δ 7.98 (d, *J* = 8.4 Hz, 1H), 7.68 (d, *J* = 2.0 Hz, 1H), 7.57 (dd, *J* = 8.4, 2.0 Hz, 1H), 4.99 (s, 2H), 4.92 (s, 2H). ^13^C NMR (126 MHz, CDCl_3_): δ 164.0, 142.0, 134.9, 133.2, 132.0, 128.3, 126.1, 94.7, 74.7, 29.9.

#### 2,2,2-Trichloroethyl-(*S*)-4-bromo-2-(((2-((*tert*-butoxycarbonyl)amino)-3-(dimethylamino)-3-oxopropyl)thio)methyl)benzoate (**103a**)

Boc-d-Cys-OH (3.32 g, 15.0 mmol, 1.5 equiv) was dissolved in DMF (10 mL) and THF (40 mL). Triethylamine (4.20 mL, 30.0 mmol, 3.0 equiv) and compound **102a** (4.25 g, 10.0 mmol, 1.0 equiv) were added, and the mixture was stirred for 16 h at rt. After evaporation of THF under reduced pressure, the mixture was diluted with EtOAc (200 mL) and washed with a 1 M aqueous HCl solution (2 × 100 mL) and brine (100 mL). The organic phase was dried over MgSO_4_, filtered, and concentrated under reduced pressure. The obtained oil was dissolved in DMF (10 mL) and THF (40 mL). Dimethylamine hydrochloride (1.23 g, 15.0 mmol, 1.5 equiv), HATU (5.70 g, 15.0 mmol, 1.5 equiv), and DIPEA (5.23 mL, 30.0 mmol, 3.0 equiv) were added, and the mixture was stirred for 2 h at rt. After evaporation of THF under reduced pressure, the mixture was diluted with EtOAc (150 mL) and washed with a 1 M aqueous HCl solution (100 mL), saturated aqueous NaHCO_3_ solution (100 mL), and brine (100 mL). The organic phase was dried over MgSO_4_, filtered, and concentrated under reduced pressure. The crude reaction mixture was purified using flash chromatography on a silica gel column with 20–100% EtOAc in hexane as the eluent to give **103a** (4.08 g, 6.89 mmol, 46% over two steps) as a yellow oil. HRMS (ESI) *m*/*z*: calcd for C_20_H_26_BrCl_3_N_2_NaO_5_S [M + Na]^+^, 612.9709; found, 612.9720. ^1^H NMR (400 MHz, CDCl_3_): δ 7.93 (d, *J* = 8.4 Hz, 1H), 7.62 (d, *J* = 2.0 Hz, 1H), 7.51 (dd, *J* = 8.4, 2.0 Hz, 1H), 5.40 (d, *J* = 8.3 Hz, 1H), 4.96–4.90 (m, 2H), 4.86–4.74 (m, 1H), 4.17 (d, *J* = 13.7 Hz, 1H), 4.11 (d, *J* = 13.7 Hz, 1H), 3.11 (s, 3H), 2.98 (s, 3H), 2.78 (dd, *J* = 13.8, 7.4 Hz, 1H), 2.64 (dd, *J* = 13.8, 5.8 Hz, 1H), 1.45 (s, 9H). ^13^C NMR (101 MHz, CDCl_3_): δ 170.9, 164.5, 155.1, 143.4, 134.3, 133.2, 130.7, 127.8, 126.5, 94.8, 80.0, 74.6, 49.4, 37.5, 35.9, 34.5, 34.2, 28.4.

#### 2,2,2-Trichloroethyl 4-Bromo-2-((4*S*,7*R*)-4-(dimethylcarbamoyl)-7-(hydroxymethyl)-11,11-dimethyl-6,9-dioxo-10-oxa-2-thia-5,8-diazadodecyl)benzoate (**104a)**

Compound **103a** (1.62 g, 2.74 mmol, 1.0 equiv) was dissolved in 4 M HCl in 1,4-dioxane (10 mL) and stirred for 1 h at rt. After evaporation of the volatiles under reduced pressure, the resulting salt was dissolved in MeCN (25 mL). Boc-d-Ser-OH (842 mg, 4.11 mmol, 1.5 equiv), EDC·HCl (785 mg, 4.11 mmol, 1.5 equiv), and DIPEA (981 μL, 5.48 mmol, 2.0 equiv) were added, and the mixture was stirred for 2 h at rt. EtOAc (200 mL) was added, and the mixture was washed with a 1 M aqueous HCl solution (100 mL), saturated aqueous NaHCO_3_ solution (100 mL), and brine (100 mL). The organic phase was dried over MgSO_4_, filtered, and concentrated under reduced pressure. The crude product was purified using flash chromatography on a silica gel column with 0–5% MeOH/DCM as the eluent to give **104a** (1.60 g, 2.36 mmol, 86% yield over two steps) as a yellow oil. HRMS (ESI) *m*/*z*: calcd for C_23_H_31_BrCl_3_N_3_NaO_7_S [M + Na]^+^, 700.0029; found, 700.0040. ^1^H NMR (500 MHz, CDCl_3_): δ 7.92 (d, *J* = 8.4 Hz, 1H), 7.61 (d, *J* = 2.0 Hz, 1H), 7.53–7.47 (m, 1H), 7.34–7.29 (m, 1H), 5.60–5.52 (m, 1H), 5.08–5.02 (m, 1H), 4.96–4.91 (m, 2H), 4.26–4.20 (m, 1H), 4.16 (d, *J* = 13.7 Hz, 1H), 4.12 (d, *J* = 13.7 Hz, 1H), 4.07–3.96 (m, 1H), 3.68–3.59 (m, 1H), 3.07 (s, 3H), 2.96 (s, 3H), 2.83 (dd, *J* = 14.0, 6.5 Hz, 1H), 2.65 (dd, *J* = 14.0, 6.9 Hz, 1H), 1.43 (s, 9H). *The CH*_*2*_*OHproton was not detectable in this spectrum.*^13^C NMR (126 MHz, CDCl_3_): δ 170.9, 170.2, 164.6, 155.7, 143.0, 134.3, 133.2, 130.8, 127.8, 126.6, 94.8, 80.4, 74.7, 63.2, 55.5, 48.4, 37.4, 36.1, 34.2, 33.9, 28.3.

#### 4-Bromo-2-((4*S*,7*R*)-4-(dimethylcarbamoyl)-7-(hydroxymethyl)-11,11-dimethyl-6,9-dioxo-10-oxa-2-thia-5,8-diazadodecyl)benzoic Acid (**105a**)

Compound **104a** (1.52 g, 2.24 mmol, 1.0 equiv) was dissolved in THF (25 mL). Aqueous 1 M NH_4_OAc (3 mL) and Zn dust (2.91 g, 44.8 mmol, 20 equiv) were added, and the mixture was vigorously stirred for 2 h at rt. The mixture was diluted with MeOH (200 mL), filtered through a pad of Celite, and concentrated under reduced pressure. The crude reaction mixture was purified using flash chromatography on a silica gel column with 70–100% EtOAc/hexane as the eluent to give **105a** (743 mg, 1.36 mmol, 61% yield) as a colorless powder. HRMS (ESI) *m*/*z*: calcd for C_21_H_30_BrN_3_NaO_7_S [M + Na]^+^, 570.0886; found, 570.0890. ^1^H NMR (500 MHz, DMSO-*d*_6_): δ 8.15 (d, *J* = 8.2 Hz, 1H), 7.76 (d, *J* = 8.2 Hz, 1H), 7.57 (d, *J* = 2.0 Hz, 1H), 7.46 (dd, *J* = 8.2, 2.0 Hz, 1H), 6.67 (d, *J* = 7.1 Hz, 1H), 4.88–4.80 (m, 2H), 4.23 (d, *J* = 12.8 Hz, 1H), 4.14 (d, *J* = 12.8 Hz, 1H), 4.03–3.97 (m, 1H), 3.58–3.51 (m, 2H), 2.95 (s, 3H), 2.81 (s, 3H), 2.81–2.75 (m, 2H), 1.38 (s, 9H). *The COOHproton was not detectable in this spectrum*. ^13^C NMR (126 MHz, DMSO-*d*_6_): δ 170.4, 170.0, 170.0, 155.6, 142.4, 133.4, 133.2, 133.1, 129.9, 123.3, 78.7, 62.4, 57.5, 49.0, 37.1, 35.8, 34.0, 33.5, 28.6.

#### *tert*-Butyl-((4*S*,7*R*)-13-bromo-4-(dimethylcarbamoyl)-6,10-dioxo-1,3,4,5,6,7,8,10-octahydrobenzo[*j*][1]oxa[8]thia[5]azacyclododecin-7-yl)carbamate (**106a**)

Compound **105a** (130 mg, 0.238 mmol, 1.0 equiv) was dissolved in THF (6 mL). Triphenylphosphine (126 mg, 0.476 mmol, 2.0 equiv) and di-*tert*-butyl azodicarboxylate (110 mg, 0.476 mmol, 2.0 equiv) were added, and the mixture was stirred for 4 h at rt. After evaporation of the volatiles under reduced pressure, the crude product was purified using reverse-phase HPLC with a gradient 25–75% MeCN/water to give **106a** (33 mg, 62 μmol, 26% yield) as a colorless powder. HRMS (ESI) *m*/*z*: calcd for C_21_H_28_BrN_3_NaO_6_S [M + Na]^+^, 552.0780; found, 552.0790. ^1^H NMR (400 MHz, CDCl_3_): δ 7.81 (d, *J* = 8.4 Hz, 1H), 7.62 (d, *J* = 2.1 Hz, 1H), 7.57 (dd, *J* = 8.4, 2.1 Hz, 1H), 7.44 (d, *J* = 9.1 Hz, 1H), 6.28–6.19 (m, 1H), 5.14 (td, *J* = 9.1, 4.3 Hz, 1H), 4.95 (dd, *J* = 11.3, 2.7 Hz, 1H), 4.53 (d, *J* = 10.1 Hz, 1H), 4.41–4.36 (m, 2H), 3.82 (d, *J* = 10.1 Hz, 1H), 3.09–3.04 (m, 1H), 3.03 (s, 3H), 2.87 (s, 3H), 2.90–2.82 (m, 1H), 1.44 (s, 9 H). ^13^C NMR (101 MHz, CDCl_3_): δ 169.3, 169.1, 167.3, 155.0, 139.1, 134.7, 133.1, 130.9, 128.7, 127.2, 81.3, 67.1, 55.2, 48.9, 37.2, 37.1, 35.9, 35.6, 28.2.

#### (4*S*,7*R*)-7-((*S*)-1-Acetylpyrrolidine-2-carboxamido)-13-bromo-*N*,*N*-dimethyl-6,10-dioxo-1,3,4,5,6,7,8,10-octahydrobenzo[*j*][1]oxa[8]thia[5]azacyclododecine-4-carboxamide (**11**)

Compound **106a** (30 mg, 57 μmol, 1.0 equiv) was dissolved in 4 M HCl in 1,4-dioxane (3 mL) and stirred for 1 h at rt. After evaporation of the volatiles under reduced pressure, the resulting salt was dissolved in DMSO (2 mL). Ac-l-Pro-OH (17 mg, 0.11 mmol, 2.0 equiv), EDC·HCl (21 mg, 0.11 mmol, 2.0 equiv), and DIPEA (30 μL, 0.17 mmol, 3.0 equiv) were added, and the reaction mixture was stirred for 2 h at rt. The crude product was purified using reverse-phase HPLC with a gradient 25–75% MeCN/water to give **11** (10 mg, 18 μmol, 32% yield) as a colorless powder. HRMS (ESI) *m*/*z*: calcd for C_23_H_29_BrN_4_NaO_6_S [M + Na]^+^, 591.0889; found, 591.0894. ^1^H NMR (500 MHz, CDCl_3_): δ 7.92 (d, *J* = 8.5 Hz, 1H), 7.78 (d, *J* = 8.4 Hz, 1H), 7.52 (d, *J* = 8.3 Hz, 1H), 7.49 (d, *J* = 1.6 Hz, 1H), 7.40 (dd, *J* = 8.4, 1.6 Hz, 1H), 5.11–5.02 (m, 2H), 4.80–4.74 (m, 1H), 4.46–4.40 (m, 2H), 4.40–4.33 (m, 1H), 3.77 (d, *J* = 10.0 Hz, 1H), 3.72–3.65 (m, 1H), 3.45–3.37 (m, 1H), 2.98 (s, 3H), 2.97–2.93 (m, 1H), 2.92–2.87 (m, 1H), 2.85 (s, 3H), 2.34–2.27 (m, 1H), 2.21–2.10 (m, 1H), 2.08 (s, 3H), 1.97–1.79 (m, 2H). ^13^C NMR (126 MHz, CDCl_3_): δ 172.2, 171.4, 169.4, 168.6, 166.9, 139.3, 135.2, 133.7, 130.9, 128.4, 127.4, 67.0, 60.0, 53.1, 49.9, 48.4, 38.0, 37.1, 35.9, 35.9, 27.5, 25.3, 22.6.

#### 2,2,2-Trichloroethyl 3-Bromo-2-methylbenzoate (**101b**)

3-Bromo-6-methylbenzoic acid (5.00 g, 23.3 mmol, 1.0 equiv) was dissolved in DCM (50 mL). EDC·HCl (6.65 g, 34.9 mmol, 1.5 equiv), DMAP (284 mg, 2.33 mmol, 0.1 equiv), and 2,2,2-trichloroethanol (22.9 mL, 30.2 mmol, 1.3 equiv) were added, and the mixture was stirred at rt for 16 h. The mixture was diluted with DCM (250 mL) and washed with a 1 M aqueous HCl solution (2 × 100 mL) and saturated aqueous NaHCO_3_ solution (2 × 100 mL). The organic phase was dried over MgSO_4_, filtered, and concentrated under reduced pressure, providing **101b** as a brown oil (4.72 g, 13.6 mmol, 58% yield). HRMS (ESI) *m*/*z*: calcd for C_10_H_7_BrCl_3_O_2_ [M – H]^−^, 342.8690; found, 342.8691. ^1^H NMR (500 MHz, CDCl_3_): δ 7.83 (dd, *J* = 8.0, 1.0 Hz, 1H), 7.68 (dd, *J* = 8.0, 1.0 Hz, 1H), 7.07 (td, *J* = 8.0, 1.0 Hz, 1H), 4.88 (s, 2H), 2.62 (s, 3H). ^13^C NMR (126 MHz, CDCl_3_): δ 165.3, 139.7, 136.9, 130.8, 129.8, 127.3, 127.0, 94.9, 74.6, 20.8.

#### 2,2,2-Trichloroethyl 3-Bromo-2-(bromomethyl)benzoate (**102b**)

Compound **101b** (4.51 g, 13.0 mmol, 1.0 equiv) was dissolved in chlorobenzene (25 mL). *N*-Bromosuccinimide (2.31 g, 13.0 mmol, 1.0 equiv) and AIBN (106 mg, 0.646 mmol, 0.05 equiv) were added, and the mixture was stirred for 16 h at 70 °C. After evaporation of the volatiles under reduced pressure, the crude product was purified using flash chromatography on a silica gel column with 2–5% EtOAc/hexane as the eluent to give **102b** (3.59 g, 8.45 mmol, 65% yield) as a yellow oil. HRMS (ESI) *m*/*z*: calcd for C_10_H_6_BrCl_3_O_2_ [M – H]^−^, 420.7800; found, 420.7828. ^1^H NMR (400 MHz, CDCl_3_): δ 8.05 (dd, *J* = 7.9, 1.3 Hz, 1H), 7.83 (dd, *J* = 7.9, 1.3 Hz, 1H), 7.29 (t, *J* = 7.9 Hz, 1H), 5.17 (s, 2H), 5.00 (s, 2H). ^13^C NMR (126 MHz, CDCl_3_): δ 164.1, 138.8, 138.1, 130.9, 129.7, 127.4, 127.0, 94.7, 74.8, 29.7.

#### 2,2,2-Trichloroethyl-(*S*)-3-bromo-2-(((2-((*tert*-butoxycarbonyl)amino)-3-(dimethylamino)-3-oxopropyl)thio)methyl)benzoate (**103b**)

Boc-d-Cys-OH (1.94 g, 8.75 mmol, 1.1 equiv) was dissolved in DMF (10 mL) and THF (40 mL). Triethylamine (2.47 mL, 17.5 mmol, 2.2 equiv) and compound **102b** (3.38 g, 7.95 mmol, 1.0 equiv) were added, and the mixture was stirred for 16 h at rt. After evaporation of THF under reduced pressure, the mixture was diluted with EtOAc (150 mL) and washed with a 1 M aqueous HCl solution (2 × 150 mL) and brine (100 mL). The organic phase was dried over MgSO_4_, filtered, and concentrated under reduced pressure. The obtained oil was dissolved in DMF (10 mL) and THF (40 mL). Dimethylamine hydrochloride (965 mg, 11.9 mmol, 1.5 equiv), HATU (4.53 g, 11.9 mmol, 1.5 equiv), and DIPEA (4.10 mL, 23.9 mmol, 3.0 equiv) were added, and the mixture was stirred for 2 h at rt. After evaporation of THF under reduced pressure, the mixture was diluted with EtOAc (150 mL) and washed with a 1 M aqueous HCl solution (100 mL), saturated aqueous NaHCO_3_ solution (100 mL), and brine (100 mL). The organic phase was dried over MgSO_4_, filtered, and concentrated under reduced pressure. The crude reaction mixture was purified using flash chromatography on a silica gel column with 20–80% EtOAc in hexane as the eluent to give **103b** (1.76 g, 3.02 mmol, 38% over two steps) as a yellow oil. HRMS (ESI) *m*/*z*: calcd for C_20_H_26_BrCl_3_N_2_NaO_5_S [M + Na]^+^, 612.9709; found, 612.9720. ^1^H NMR (500 MHz, CDCl_3_): δ 7.96 (d, *J* = 7.9 Hz, 1H), 7.77 (d, *J* = 7.9 Hz, 1H), 7.21 (t, *J* = 7.9 Hz, 1H), 5.41–5.30 (m, 1H), 4.99–4.93 (m, 2H), 4.86–4.77 (m, 1H), 4.46–4.38 (s, 2H), 3.11 (s, 3H), 2.96 (s, 3H), 2.91–2.76 (m, 2H), 1.42 (s, 9H). ^13^C NMR (126 MHz, CDCl_3_): δ 170.9, 164.8, 155.1, 140.5, 137.5, 130.6, 130.4, 128.4, 126.8, 94.8, 79.8, 74.8, 49.8, 37.5, 35.9, 35.7, 33.9, 28.4.

#### 2,2,2-Trichloroethyl 3-Bromo-2-((4*S*,7*R*)-4-(dimethylcarbamoyl)-7-(hydroxymethyl)-11,11-dimethyl-6,9-dioxo-10-oxa-2-thia-5,8-diazadodecyl)benzoate (**104b**)

Compound **103b** (1.12 g, 1.89 mmol, 1.0 equiv) was dissolved in 4 M HCl in 1,4-dioxane (10 mL) and stirred for 1 h at rt. After evaporation of the volatiles under reduced pressure, the resulting salt was dissolved in MeCN (20 mL). Boc-d-Ser-OH (581 mg, 2.84 mmol, 1.5 equiv), EDC·HCl (542 mg, 2.84 mmol, 1.5 equiv), and DIPEA (658 μL, 3.78 mmol, 2.0 equiv) were added, and the reaction mixture was stirred for 2 h at rt. EtOAc (100 mL) was added, and the mixture was washed with a 1 M aqueous HCl solution (50 mL), saturated aqueous NaHCO_3_ (50 mL), and brine (50 mL). The organic phase was dried over MgSO_4_, filtered, and concentrated under reduced pressure. The crude product was purified using flash chromatography on a silica gel column with 0–10% MeOH/DCM as the eluent to give **104b** (961 mg, 1.42 mmol, 75% yield over two steps) as a yellow oil. HRMS (ESI) *m*/*z*: calcd for C_23_H_31_BrCl_3_N_3_NaO_7_S [M + Na]^+^, 700.0029; found, 700.0040. ^1^H NMR (400 MHz, CDCl_3_): δ 7.98 (dd, *J* = 7.8, 1.2 Hz, 1H), 7.79 (dd, *J* = 7.8, 1.2 Hz, 1H), 7.24 (t, *J* = 7.8, 1H), 7.06 (d, *J* = 9.0 Hz, 1H), 5.47 (d, *J* = 7.0 Hz, 1H), 5.11–5.03 (m, 1H), 4.99–4.95 (m, 2H), 4.45 (d, *J* = 12.9 Hz, 1H), 4.38 (d, *J* = 12.9 Hz, 1H), 4.26–4.19 (m, 1H), 4.11–4.04 (m, 1H), 3.62 (dd, *J* = 11.7, 5.6 Hz, 1H), 3.10 (s, 3H), 3.01 (dd, *J* = 13.8, 5.6 Hz, 1H), 2.95 (s, 3H), 2.82 (dd, *J* = 13.8, 8.0 Hz, 1H), 1.45 (s, 9H). *The CH*_*2*_*OHproton was not detectable in this spectrum.*^13^C NMR (126 MHz, CDCl_3_): δ 170.9, 170.1, 164.9, 155.7, 140.1, 137.6, 130.7, 130.3, 128.6, 126.9, 94.7, 80.4, 74.9, 63.4, 55.6, 48.9, 37.3, 36.1, 34.9, 34.0, 28.3.

#### 3-Bromo-2-((4*S*,7*R*)-4-(dimethylcarbamoyl)-7-(hydroxymethyl)-11,11-dimethyl-6,9-dioxo-10-oxa-2-thia-5,8-diazadodecyl)benzoic Acid (**105b**)

Compound **104b** (900 mg, 1.33 mmol, 1.0 equiv) was dissolved in THF (15 mL). Aqueous 1 M NH_4_OAc (3 mL) and Zn dust (1.73 g, 26.6 mmol, 20 equiv) were added, and the reaction mixture was vigorously stirred for 2 h at rt. The mixture was diluted with MeOH (150 mL), filtered through a pad of Celite, and concentrated under reduced pressure. The crude product was purified using flash chromatography on a silica gel column with 70–90% EtOAc/hexane as the eluent to give **105b** (443 mg, 0.809 mmol, 61% yield) as a colorless powder. HRMS (ESI) *m*/*z*: calcd for C_21_H_31_BrN_3_O_7_S [M + H]^+^, 548.1061; found, 548.1043. ^1^H NMR (500 MHz, DMSO-*d*_6_, mixture of two rotamers in 0.4:0.6 ratio): δ 8.21 (d, *J* = 8.4 Hz, 0.4H), 8.12 (d, *J* = 8.4 Hz, 0.6H), 7.82–7.74 (m, 2H), 7.29 (t, *J* = 7.9 Hz, 1H), 6.67 (d, *J* = 8.3 Hz, 0.4H), 6.64 (d, *J* = 8.2 Hz, 0.6H), 4.92–4.87 (m, 1H), 4.35–4.31 (m, 1H), 4.25 (d, *J* = 12.7 Hz, 1H), 4.03–3.98 (m, 1H), 3.60–3.48 (m, 2H), 3.01 (s, 1.2H), 2.99 (s, 1.8H), 2.91 (dd, *J* = 13.1, 7.8 Hz, 1H), 2.84 (s, 1.2H), 2.83 (s, 1.8H), 2.68 (dd, *J* = 13.6, 6.1 Hz, 1H), 1.39 (s, 5.4H), 1.38 (s, 3.6H). *The COOHand CH*_*2*_*OH-protons were not detectable in this spectrum.*^13^C NMR (126 MHz, DMSO-*d*_6_, *mixture of 2 rotamers*): δ 170.4 and 170.3 (1C), 170.0 (1C), 168.6 and 168.6 (1C), 155.6 and 155.6 (1C), 138.9 (1C), 136.3 (1C), 134.2 (1C), 130.2 (1C), 129.2 (1C), 126.2 (1C), 78.7 and 78.6 (1C), 62.4 (1C), 57.5 and 57.2 (1C), 49.0 and 48.9 (1C), 37.1 (1C), 35.8 (1C), 34.8 (1C), 33.8 and 33.6 (1C), 28.6 (3C).

#### *tert*-Butyl-((4*S*,7*R*)-14-bromo-4-(dimethylcarbamoyl)-6,10-dioxo-1,3,4,5,6,7,8,10-octahydrobenzo[*j*][1]oxa[8]thia[5]azacyclododecin-7-yl)carbamate (**106b**)

Compound **105b** (145 mg, 0.265 mmol, 1.0 equiv) was dissolved in THF (9 mL). Triphenylphosphine (136 mg, 0.530 mmol, 2.0 equiv) and di-*tert*-butyl azodicarboxylate (120 mg, 0.530 mmol, 2.0 equiv) were added, and the mixture was stirred for 4 h at rt. After evaporation of the volatiles under reduced pressure, the crude product was purified using reverse-phase HPLC with a gradient 30% to 75% MeCN in water to give **106b** (42 mg, 80 μmol, 31% yield) as a colorless powder. HRMS (ESI) *m*/*z*: calcd for C_21_H_28_BrN_3_NaO_6_S [M + Na]^+^, 552.0780; found, 552.0774. ^1^H NMR (500 MHz, CDCl_3_): δ 7.74–7.69 (m, 2H), 7.39 (d, *J* = 8.2 Hz, 1H), 7.19 (t, *J* = 7.9 Hz, 1H), 5.61–5.54 (s, 1H), 5.20 (dd, *J* = 11.3, 2.6 Hz, 1H), 5.19–5.13 (m, 1H), 4.67–4.61 (m, 1H), 4.40 (dd, *J* = 11.3, 2.0 Hz, 1H), 4.30 (d, *J* = 10.1 Hz, 1H), 4.13 (d, *J* = 10.1 Hz, 1H), 3.15–3.09 (m 2H), 3.09 (s, 3H), 2.95 (s, 3H), 1.46 (s, 9H). ^13^C NMR (126 MHz, CDCl_3_): δ 169.2, 169.1, 167.6, 155.1, 137.0, 136.1, 132.9, 130.5, 128.7, 126.9, 81.3, 67.1, 55.1, 48.8, 37.1, 37.0, 35.9, 35.7, 28.2.

#### (4*S*,7*R*)-7-((*S*)-1-Acetylpyrrolidine-2-carboxamido)-14-bromo-*N*,*N*-dimethyl-6,10-dioxo-1,3,4,5,6,7,8,10-octahydrobenzo[*j*][1]oxa[8]thia[5]azacyclododecine-4-carboxamide (**12**)

Compound **106b** (40 mg, 75 μmol, 1.0 equiv) was dissolved in 4 M HCl in 1,4-dioxane (3 mL) and stirred for 1 h at rt. After evaporation of the volatiles under reduced pressure, the resulting salt was dissolved in DMSO (2 mL). Ac-l-Pro-OH (24 mg, 0.15 mmol, 2.0 equiv), EDC·HCl (29 mg, 0.15 mmol, 2.0 equiv), and DIPEA (41 μL, 0.23 mmol, 3.0 equiv) were added, and the reaction mixture was stirred for 2 h at rt. The crude product was purified using reverse-phase HPLC with a gradient 25% to 75% MeCN in water to give **12** (15 mg, 27 μmol, 36% yield) as a colorless powder. HRMS (ESI) *m*/*z*: calcd for C_23_H_30_BrN_4_O_6_S [M + H]^+^, 569.1069; found, 569.1074. ^1^H NMR (400 MHz, CDCl_3_): δ 8.08 (d, *J* = 8.6 Hz, 1H), 7.83 (dd, *J* = 7.8, 1.2 Hz, 1H), 7.72 (dd, *J* = 7.8, 1.2 Hz, 1H), 7.59 (d, *J* = 8.9 Hz, 1H), 7.18 (t, *J* = 7.8 Hz, 1H), 5.26 (dd, *J* = 11.1, 2.6 Hz, 1H), 5.17–5.07 (m, 1H), 4.86 (ddd, *J* = 8.6, 2.6, 2.0 Hz, 1H), 4.52 (d, *J* = 10.1 Hz, 1H), 4.50–4.46 (m, 1H), 4.39 (dd, *J* = 11.1, 2.0 Hz, 1H), 4.14 (d, *J* = 10.1 Hz, 1H), 3.79–3.70 (m, 1H), 3.50–3.41 (m, 1H), 3.12–2.97 (m, 2H), 3.05 (s, 3H), 2.91 (s, 3H), 2.42–2.31 (m, 1H), 2.25–2.16 (m, 1H), 2.13 (s, 3H), 2.07–1.86 (m, 2H). ^13^C NMR (101 MHz, CDCl_3_): δ 172.0, 171.5, 169.3, 168.6, 167.2, 137.2, 136.1, 132.6, 131.1, 128.6, 127.1, 66.9, 60.0, 53.3, 49.7, 48.3, 37.5, 37.0, 35.9, 35.8, 27.5, 25.2, 22.5.

#### (4*S*,7*R*)-7-((*S*)-1-Acetylpyrrolidine-2-carboxamido)-*N*,*N*,12-trimethyl-6,10-dioxo-1,3,4,5,6,7,8,10-octahydrobenzo[*j*][1]oxa[8]thia[5]azacyclododecine-4-carboxamide (**13**)

A 5 mL vial was charged with compound **10** (30 mg, 52 μmol, 1.0 equiv), XPhos Pd G3 (7 mg, 8 μmol, 15 mol %), K_3_PO_4_ (33 mg, 0.16 mmol, 3.0 equiv), and potassium methyltrifluoroborate (20 mg, 0.16 mmol, 3.0 equiv). After the tube was evacuated and refilled with argon three times, a mixture of degassed toluene/water 3:1 (0.70 mL) was added. The vial was sealed, and the reaction mixture was heated in a microwave reactor at 135 °C for 2 h. The reaction mixture was diluted with MeOH (10 mL) and filtered through a 0.45 μm syringe filter. After evaporation of the volatiles under reduced pressure, the crude product was purified using reverse-phase HPLC with a gradient 25–75% MeCN/water to give **13** (5 mg, 9 μmol, 17%) as a colorless solid. HRMS (ESI) *m*/*z*: calcd for C_24_H_33_N_4_O_6_S [M + H]^+^, 505.2115; found, 505.2100. ^1^H NMR (500 MHz, CDCl_3_): δ 7.93 (d, *J* = 8.9 Hz, 1H), 7.81–7.78 (m, 1H), 7.62 (d, *J* = 9.3 Hz, 1H), 7.33–7.26 (m, 2H), 5.20–5.12 (m, 2H), 4.88 (dt, *J* = 8.9, 2.3 Hz, 1H), 4.52–4.48 (m, 1H), 4.47 (d, *J* = 10.1 Hz, 1H), 4.45 (dd, *J* = 11.3, 2.3 Hz, 1H), 3.89 (d, *J* = 10.1 Hz, 1H), 3.78–3.73 (m, 1H), 3.52–3.47 (m, 1H), 3.08 (s, 3H), 3.08–3.05 (m, 1H), 3.02–2.97 (m, 1H), 2.94 (s, 3H), 2.41–2.37 (m, 1H), 2.38 (s, 3H), 2.30–2.21 (m, 1H), 2.17 (s, 3H), 2.07–1.95 (m, 2H). ^13^C NMR (126 MHz, CDCl_3_): δ 172.0, 171.5, 169.5, 168.7, 167.9, 137.6, 134.2, 133.5, 132.6, 132.4, 129.3, 66.7, 60.0, 53.2, 49.9, 48.3, 38.2, 37.1, 35.8, 35.8, 27.6, 25.3, 22.6, 20.9.

#### (4*S*,7*R*)-7-((*S*)-1-Acetylpyrrolidine-2-carboxamido)-12-hydroxy-*N*,*N*-dimethyl-6,10-dioxo-1,3,4,5,6,7,8,10-octahydrobenzo[*j*][1]oxa[8]thia[5]azacyclododecine-4-carboxamide (**14**)

A 5 mL vial was charged with compound **10** (30 mg, 52 μmol, 1.0 equiv), Pd(dppf)Cl_2_ (8 mg, 10 μmol, 20 mol %), B_2_(pin)_2_ (16 mg, 63 μmol, 1.2 equiv), and KOAc (16 mg, 0.16 mmol, 3.0 equiv). After the tube was evacuated and refilled with argon three times, 1,4-dioxane (0.52 mL) was added. The vial was sealed, and the reaction mixture was stirred for 16 h at 90 °C in a preheated oil bath. The reaction mixture was allowed to cool down to rt, diluted with MeOH (25 mL), and filtered through a plug of Celite. After evaporation of the volatiles under reduced pressure, the obtained solid was dissolved in a mixture of THF/water 1:1 (2 mL). NaBO_3_·4H_2_O (12 mg, 78 μmol, 1.5 equiv) was added, and the reaction mixture was stirred for 2 h at rt. The mixture was diluted with EtOAc (20 mL) and washed with brine (2 × 5 mL). The organic phase was dried over MgSO_4_, filtered, and concentrated under reduced pressure. The crude product was purified using reverse-phase HPLC with a gradient 15–65% MeCN/water to give **14** (7 mg, 13 μmol, 25% over two steps) as a colorless solid. HRMS (ESI) *m*/*z*: calcd for C_23_H_31_N_4_O_6_S [M + H]^+^, 507.1908; found, 507.1897. ^1^H NMR (500 MHz, CDCl_3_): δ 8.94 (br s, 1H), 8.31 (d, *J* = 6.4 Hz, 1H), 7.79 (d, *J* = 2.6 Hz, 1H), 7.47 (d, *J* = 9.8 Hz, 1H), 6.85–6.76 (m, 2H), 5.21 (ddd, *J* = 11.4, 9.8, 4.5 Hz, 1H), 4.82 (dd, *J* = 11.1, 3.0 Hz, 1H), 4.76 (dd, *J* = 11.1, 1.7 Hz, 1H), 4.75–4.65 (m, 2H), 4.25 (d, *J* = 9.6 Hz, 1H), 3.80–3.71 (m, 1H), 3.62–3.55 (m, 1H), 3.26 (d, *J* = 9.6 Hz, 1H), 3.08 (s, 3H), 3.00 (dd, *J* = 15.0, 4.5 Hz, 1H), 2.94 (s, 3H), 2.82 (dd, *J* = 15.0, 11.4 Hz, 1H), 2.47–2.32 (m, 2H), 2.29 (s, 3H), 2.18–2.10 (m, 1H), 2.07–1.99 (m, 1H). ^13^C NMR (126 MHz, CDCl_3_): δ 173.5, 172.1, 169.5, 168.3, 168.2, 156.3, 135.1, 128.8, 127.3, 121.2, 118.3, 67.0, 60.3, 53.9, 50.5, 48.7, 39.9, 37.1, 36.0, 35.8, 28.6, 25.3, 22.7.

#### (4*S*,7*R*)-7-((*S*)-1-Acetylpyrrolidine-2-carboxamido)-12-methoxy-*N*,*N*-dimethyl-6,10-dioxo-1,3,4,5,6,7,8,10-octahydrobenzo[*j*][1]oxa[8]thia[5]azacyclododecine-4-carboxamide (**15**)

Compound **14** (11 mg, 20 μmol, 1.0 equiv) was dissolved in DMSO (0.75 mL). K_2_CO_3_ (11 mg, 80 μmol, 4.0 equiv) and MeI (10 μL, 0.16 mmol, 8.0 equiv) were added, and the reaction mixture was stirred for 1 h at 90 °C in a preheated oil bath. The reaction mixture was allowed to cool down to rt, diluted with MeOH (10 mL), and filtered through a 0.45 μm syringe filter. After evaporation of the volatiles under reduced pressure, the crude product was purified using reverse-phase HPLC with a gradient 25–75% MeCN/water to give **15** (3 mg, 6 μmol, 30%) as a colorless solid. HRMS (ESI) *m*/*z*: calcd for C_24_H_33_N_4_O_7_S [M + H]^+^, 521.2070; found, 521.2077. ^1^H NMR (500 MHz, CDCl_3_): δ 7.93 (d, *J* = 9.0 Hz, 1H), 7.58 (d, *J* = 9.1 Hz, 1H), 7.48 (d, *J* = 2.9 Hz, 1H), 7.29 (d, *J* = 8.5 Hz, 1H), 7.00 (dd, *J* = 8.5, 2.9 Hz, 1H), 5.19–5.08 (m, 1H), 5.16 (dd, *J* = 11.2, 2.6 Hz, 1H), 4.86 (ddd, *J* = 9.1, 2.6, 2.0 Hz, 1H), 4.44–4.40 (m, 1H), 4.39 (d, *J* = 10.0 Hz, 1H), 4.36 (dd, *J* = 11.2, 2.0 Hz, 1H), 3.84 (d, *J* = 10.0 Hz, 1H), 3.82 (s, 3H), 3.76–3.70 (m, 1H), 3.49–3.42 (m, 1H), 3.05 (s, 3H), 2.96 (dd, *J* = 15.5, 4.5 Hz, 1H), 2.88 (dd, *J* = 15.5, 8.9 Hz, 1H), 2.91 (s, 3H), 2.39–2.32 (m, 1H), 2.25–2.18 (m, 1H), 2.15 (s, 3H), 2.04–1.94 (m, 2H). ^13^C NMR (126 MHz, CDCl_3_): δ 172.1, 171.4, 169.5, 168.7, 167.5, 158.7, 133.7, 130.5, 129.3, 119.3, 116.2, 66.9, 60.0, 55.5, 53.1, 50.0, 48.3, 38.0, 37.1, 35.8, 35.7, 27.5, 25.3, 22.6.

#### (4*S*,7*R*)-7-((*S*)-1-Acetylpyrrolidine-2-carboxamido)-12-cyano-*N*,*N*-dimethyl-6,10-dioxo-1,3,4,5,6,7,8,10-octahydrobenzo[*j*][1]oxa[8]thia[5]azacyclododecine-4-carboxamide (**16**)

A 5 mL vial was charged with compound **10** (30 mg, 52 μmol, 1.0 equiv), ^*t*^BuBrettPhos Pd G3 (7 mg, 8 μmol, 15 mol %), and Zn(CN)_2_ (15 mg, 0.13 mmol, 2.5 equiv). After the tube was evacuated and refilled with argon three times, a mixture of degassed THF/water 1:3 (0.70 mL) was added. The vial was sealed, and the reaction mixture was stirred for 16 h at 40 °C in a preheated oil bath. The reaction mixture was allowed to cool down to rt, diluted with MeOH (10 mL), and filtered through a 0.45 μm syringe filter. After evaporation of the volatiles under reduced pressure, the crude product was purified using reverse-phase HPLC with a gradient 20–70% MeCN/water to give **16** (9 mg, 17 μmol, 32%) as a colorless solid. HRMS (ESI) *m*/*z*: calcd for C_24_H_30_N_5_O_6_S [M + H]^+^, 516.1911; found, 516.1900. ^1^H NMR (500 MHz, CDCl_3_): δ 8.28 (d, *J* = 1.8 Hz, 1H), 8.02 (d, *J* = 9.0 Hz, 1H), 7.74 (dd, *J* = 7.9, 1.8 Hz, 1H), 7.62 (d, *J* = 9.2 Hz, 1H), 7.54 (d, *J* = 7.9 Hz, 1H), 5.22 (dd, *J* = 11.0, 2.6 Hz, 1H), 5.16 (td, *J* = 9.0, 4.5 Hz, 1H), 4.92–4.88 (m, 1H), 4.59–4.45 (m, 3H), 3.95 (d, *J* = 10.0 Hz, 1H), 3.79–3.73 (m, 1H), 3.53–3.45 (m, 1H), 3.11–3.03 (m, 1H), 3.08 (s, 3H), 3.03–2.97 (m, 1H), 2.94 (s, 3H), 2.44–2.37 (m, 1H), 2.27–2.18 (m, 1H), 2.17 (s, 3H), 2.09–1.92 (m, 2H). ^13^C NMR (126 MHz, CDCl_3_): δ 172.3, 171.4, 169.2, 168.5, 165.8, 142.5, 135.9, 135.3, 133.4, 131.0, 117.6, 112.0, 67.5, 60.0, 53.1, 49.8, 48.4, 37.1, 37.1, 35.9, 35.9, 27.5, 25.3, 22.7.

#### (4*S*,7*R*)-7-((*R*)-1-Acetylpyrrolidine-2-carboxamido)-*N*,*N*,14-trimethyl-6,10-dioxo-1,3,4,5,6,7,8,10-octahydrobenzo[*j*][1]oxa[8]thia[5]azacyclododecine-4-carboxamide (**17**)

A 5 mL vial was charged with compound **12** (81 mg, 0.14 mmol, 1.0 equiv), XPhos Pd G3 (18 mg, 21 μmol, 15 mol %), K_2_CO_3_ (58 mg, 0.42 mmol, 3.0 equiv), and potassium methyltrifluoroborate (44 mg, 0.35 mmol, 2.5 equiv). After the tube was evacuated and refilled with argon three times, a mixture of degassed toluene/water 4:1 (2.0 mL) was added. The vial was sealed, and the reaction mixture was stirred was stirred for 16 h at 90 °C in a preheated oil bath. The reaction mixture was allowed to cool down to rt, diluted with MeOH (10 mL), and filtered through a 0.45 μm syringe filter. After evaporation of the volatiles under reduced pressure, the crude product was purified using reverse-phase HPLC with a gradient 25–75% MeCN/water to give **17** (14 mg, 27 μmol, 19%) as a colorless solid. HRMS (ESI) *m*/*z*: calcd for C_24_H_33_N_4_O_6_S [M + H]^+^, = 505.2115; found, 505.2110. ^1^H NMR (500 MHz, CDCl_3_): δ 7.86 (d, *J* = 8.8 Hz, 1H), 7.77–7.68 (m, 2H), 7.34 (d, *J* = 7.7 Hz, 1H), 7.25 (t, *J* = 7.7 Hz, 1H), 5.22 (dd, *J* = 11.1, 2.7 Hz, 1H), 5.19–5.14 (m, 1H), 4.90 (dt, *J* = 8.8, 2.7 Hz, 1H), 4.49–4.43 (m, 2H), 4.34 (d, *J* = 10.0 Hz, 1H), 3.95 (d, *J* = 10.0 Hz, 1H), 3.76–3.71 (m, 1H), 3.53–3.45 (m, 1H), 3.15–3.04 (m, 5H), 2.94 (s, 3H), 2.54 (s, 3H), 2.39–2.33 (m, 1H), 2.28–2.21 (m, 1H), 2.14 (s, 3H), 2.07–1.95 (m, 2H). ^13^C NMR (126 MHz, CDCl_3_): δ 171.7, 171.7, 169.4, 168.8, 168.8, 138.9, 134.6, 134.5, 131.1, 129.5, 127.3, 66.6, 60.2, 53.5, 49.8, 48.3, 37.1, 35.9, 35.8, 34.5, 27.9, 25.3, 22.5, 19.6.

#### (4*S*,7*R*)-7-((*S*)-1-Acetylpyrrolidine-2-carboxamido)-14-hydroxy-*N*,*N*-dimethyl-6,10-dioxo-1,3,4,5,6,7,8,10-octahydrobenzo[*j*][1]oxa[8]thia[5]azacyclododecine-4-carboxamide (**18**)

A 5 mL vial was charged with **107**(9) (82 mg, 0.18 mmol, 1.0 equiv), [Ir(OMe)(cod)]_2_ (18 mg, 27 μmol, 15 mol %), and B_2_pin_2_ (96 mg, 0.36 mmol, 2.0 equiv). After the tube was evacuated and refilled with argon three times, 1,4-dioxane (2 mL) was added. The vial was sealed, and the reaction mixture was stirred for 16 h at 95 °C in a preheated oil bath. The reaction mixture was allowed to cool down to rt and filtered through a plug of Celite using EtOAc as the eluent. After evaporation of the volatiles under reduced pressure, the obtained oil was dissolved in THF (1 mL) and H_2_O (1 mL). NaBO_3_·H_2_O (28 mg, 0.18 mmol, 1.0 equiv) was added, and the reaction mixture was stirred for 1 h at rt. The mixture was diluted with EtOAc (20 mL) and washed with brine (2 × 5 mL). The organic phase was dried over MgSO_4_, filtered, and concentrated under reduced pressure. The resulting solid was dissolved in 4 M HCl in 1,4-dioxane (2 mL), and the reaction mixture was stirred for 1 h at rt. After evaporation of the volatiles under reduced pressure, the obtained salt was dissolved in DMSO (1 mL). Ac-l-Pro-OH (42 mg, 0.27 mmol, 1.5 equiv), EDC·HCl (52 mg, 0.27 mmol, 1.5 equiv), and DIPEA (90 μL, 0.54 mmol, 3.0 equiv) were added, and the mixture was stirred for 2 h at rt. The crude product was purified using reverse-phase HPLC with a gradient 25–75% MeCN/water to give **18** (7 mg, 14 μmol, 8% over 4 steps) as a colorless solid. HRMS (ESI) *m*/*z*: calcd for C_23_H_31_N_4_O_6_S [M + H]^+^, 507.1908; found, 507.1907. ^1^H NMR (500 MHz, CDCl_3_): δ 7.99 (d, *J* = 9.0 Hz, 1H), 7.66 (d, *J* = 9.2 Hz, 1H), 7.43 (d, *J* = 7.9 Hz, 1H), 7.14 (t, *J* = 7.9 Hz, 1H), 7.04 (d, *J* = 7.9 Hz, 1H), 5.22–5.16 (m, 1H), 5.13 (dd, *J* = 11.1, 2.8 Hz, 1H), 4.92 (ddd, *J* = 9.0, 2.8, 1.9 Hz, 1H), 4.56–4.52 (m, 1H), 4.50 (dd, *J* = 11.2, 1.9 Hz, 1H), 4.40 (d, *J* = 10.5 Hz, 1H), 4.05 (d, *J* = 10.5 Hz, 1H), 3.82–3.75 (m, 1H), 3.54–3.47 (m, 1H), 3.08 (s, 3H), 3.06–2.97 (m, 2H), 2.95 (s, 3H), 2.43–2.36 (m, 1H), 2.30–2.23 (m, 1H), 2.21 (s, 3H), 2.09–1.93 (m, 2H). *The CH*_*2*_*OHproton was not detectable in this spectrum.*^13^C NMR (126 MHz, CDCl_3_): δ 172.5, 171.4, 169.4, 168.7, 167.8, 155.8, 131.1, 128.4, 124.0, 122.8, 120.5, 66.7, 60.1, 53.2, 49.9, 48.4, 37.1, 35.9, 35.1, 31.5, 27.6, 25.2, 22.5.

#### (4*S*,7*R*)-7-((*S*)-1-Acetylpyrrolidine-2-carboxamido)-14-methoxy-*N*,*N*-dimethyl-6,10-dioxo-1,3,4,5,6,7,8,10-octahydrobenzo[*j*][1]oxa[8]thia[5]azacyclododecine-4-carboxamide (**19**)

Compound **18** (7.0 mg, 14 μmol, 1.0 equiv) was dissolved in DMSO (0.75 mL). K_2_CO_3_ (11 mg, 80 μmol, 5.0 equiv) and MeI (10 μL, 0.14 mmol, 10 equiv) were added, and the reaction mixture was stirred for 1 h at 90 °C in a preheated oil bath. The crude product was purified using reverse-phase HPLC with a gradient 20–70% MeCN/water to give **19** (3 mg, 6 μmol, 42%) as a colorless solid. HRMS (ESI) *m*/*z*: calcd for C_24_H_33_N_4_O_7_S [M + H]^+^, 521.2064; found, 521.2050. ^1^H NMR (400 MHz, CDCl_3_): δ 8.00 (d, *J* = 8.8 Hz, 1H), 7.57 (d, *J* = 8.9 Hz, 1H), 7.50 (d, *J* = 7.9 Hz, 1H), 7.29 (t, *J* = 7.9 Hz, 1H), 7.04 (d, *J* = 7.9 Hz, 1H), 5.19 (dd, *J* = 11.1, 2.7 Hz, 1H), 5.12 (ddd, *J* = 8.9, 8.8, 4.5 Hz, 1H), 4.89–4.80 (m, 1H), 4.51–4.46 (m, 1H), 4.44–4.36 (m, 2H), 4.09 (d, *J* = 9.8 Hz, 1H), 3.88 (s, 3H), 3.78–3.70 (m, 1H), 3.48–3.41 (m, 1H), 3.10–3.06 (m, 1H), 3.05 (s, 3H), 2.98 (dd, *J* = 14.6, 8.8 Hz, 1H), 2.91 (s, 3H), 2.40–2.33 (m, 1H), 2.25–2.17 (m, 1H), 2.14 (s, 3H), 2.01–1.89 (m, 2H). ^13^C NMR (126 MHz, CDCl_3_): δ 172.0, 171.5, 169.5, 168.7, 168.1, 158.1, 131.6, 128.3, 125.7, 123.8, 114.7, 66.7, 60.0, 56.4, 53.4, 49.9, 48.3, 37.1, 35.9, 30.6, 30.6, 27.6, 25.2, 22.6.

#### (4*S*,7*R*)-7-((*S*)-1-Acetylpyrrolidine-2-carboxamido)-14-cyclopropyl-*N*,*N*-dimethyl-6,10-dioxo-1,3,4,5,6,7,8,10-octahydrobenzo[*j*][1]oxa[8]thia[5]azacyclododecine-4-carboxamide (**20**)

A 5 mL vial was charged with compound **12** (30 mg, 52 μmol, 1.0 equiv), XPhos Pd G3 (7 mg, 8 μmol, 15 mol %), K_3_PO_4_ (28 mg, 0.13 mmol, 2.5 equiv), and potassium cyclopropyltrifluoroborate (15 mg, 0.10 mmol, 2.0 equiv). After the tube was evacuated and refilled with argon three times, a mixture of degassed 1,4-dioxane:H_2_O 3:1 (0.7 mL) was added. The vial was sealed, and the reaction mixture was heated in a microwave reactor at 135 °C for 2 h. The reaction mixture was cooled down to rt, diluted with MeOH (10 mL), and filtered through a 0.45 μm syringe filter. After evaporation of the volatiles under reduced pressure, the crude product was purified using reverse-phase HPLC with a gradient 20–70% MeCN/water to give **20** (5 mg, 9 μmol, 18%) as a colorless solid. HRMS (ESI) *m*/*z*: calcd for C_26_H_35_N_4_O_6_S [M + H]^+^, 531.2272; found, 531.2260. ^1^H NMR (500 MHz, CDCl_3_): δ 7.91 (d, *J* = 8.7 Hz, 1H), 7.74–7.66 (m, 2H), 7.25 (t, *J* = 7.7 Hz, 1H), 7.21 (d, *J* = 7.7 Hz, 1H), 5.25 (dd, *J* = 11.2, 2.6 Hz, 1H), 5.19–5.14 (m, 1H), 4.90 (ddd, *J* = 8.7, 2.6, 2.0 Hz, 1H), 4.52 (d, *J* = 10.2 Hz, 1H), 4.49–4.42 (m, 2H), 4.22 (d, *J* = 10.2 Hz, 1H), 3.77–3.71 (m, 1H), 3.52–3.45 (m, 1H), 3.13–3.07 (m, 2H), 3.09 (s, 3H), 2.95 (s, 3H), 2.39–2.29 (m, 2H), 2.26–2.21 (m, 1H), 2.13 (s, 3H), 2.04–1.95 (m, 2H), 1.07–0.98 (m, 2H), 0.75–0.69 (m, 2H). ^13^C NMR (126 MHz, CDCl_3_): δ 171.8, 171.6, 169.4, 168.9, 168.8, 143.5, 136.1, 131.2, 130.3, 129.2, 127.4, 66.5, 60.2, 53.6, 49.7, 48.3, 37.1, 36.0, 35.9, 34.0, 27.9, 25.3, 22.5, 13.2, 7.4, 7.3.

#### (4*S*,7*R*)-7-((*S*)-1-Acetylpyrrolidine-2-carboxamido)-14-cyano-*N*,*N*-dimethyl-6,10-dioxo-1,3,4,5,6,7,8,10-octahydrobenzo[*j*][1]oxa[8]thia[5]azacyclododecine-4-carboxamide (**21**)

A 5 mL vial was charged with compound **12** (30 mg, 52 μmol, 1.0 equiv) and copper (I) cyanide (14 mg, 0.16 mmol, 2.0 equiv). After the tube was evacuated and refilled with argon three times, NMP (0.30 mL) was added. The vial was sealed, and the reaction mixture was heated in a microwave reactor at 180 °C for 20 min. The reaction mixture was cooled down to rt, diluted with MeOH (10 mL), and filtered through a 0.45 μm syringe filter. After evaporation of the volatiles under reduced pressure, the crude product was purified using reverse-phase HPLC with a gradient 15–65% MeCN/water to give **21** (11 mg, 21 μmol, 41%) as a colorless solid. HRMS (ESI) *m*/*z*: calcd for C_24_H_30_N_5_O_6_S [M + H]^+^, 516.1911; found, 516.1900. ^1^H NMR (400 MHz, CDCl_3_): δ 8.23 (d, *J* = 9.1 Hz, 1H), 8.18 (dd, *J* = 8.0, 1.5 Hz, 1H), 7.80 (dd, *J* = 7.7, 1.5 Hz, 1H), 7.52–7.41 (m, 2H), 5.25 (dd, *J* = 11.1, 2.6 Hz, 1H), 5.15 (ddd, *J* = 9.5, 9.1, 4.5 Hz, 1H), 4.92–4.82 (m, 1H), 4.67 (d, *J* = 10.5 Hz, 1H), 4.53 (dd, *J* = 8.1, 2.7 Hz, 1H), 4.42 (dd, *J* = 11.1, 2.2 Hz, 1H), 4.17 (d, *J* = 10.5 Hz, 1H), 3.83–3.73 (m, 1H), 3.50–3.40 (m, 1H), 3.15 (dd, *J* = 14.8, 4.5 Hz, 1H), 3.06 (s, 3H), 2.97 (dd, *J* = 14.8, 9.5 Hz, 1H), 2.92 (s, 3H), 2.45–2.39 (m, 1H), 2.24–2.18 (m, 1H), 2.16 (s, 3H), 2.03–1.91 (m, 2H). ^13^C NMR (126 MHz, CDCl_3_): δ 172.5, 171.2, 169.1, 168.5, 166.1, 140.7, 136.9, 136.3, 131.5, 128.1, 117.0, 115.8, 67.5, 59.9, 53.0, 49.7, 48.4, 37.1, 36.4, 35.9, 35.8, 27.1, 25.2, 22.7.

#### (4*S*,7*R*)-7-((*S*)-1-Acetylpyrrolidine-2-carboxamido)-4-(dimethylcarbamoyl)-6,10-dioxo-1,3,4,5,6,7,8,10-octahydrobenzo[*j*][1]oxa[8]thia[5]azacyclododecine-14-carboxylic Acid (**22**)

A 5 mL vial was charged with compound **12** (30 mg, 52 μmol, 1.0 equiv), Pd(OAc)_2_ (5 mg, 22 μmol, 40 mol %), DMAP (14 mg, 0.11 mmol, 2.0 equiv), and Mo(CO)_6_ (29 mg, 0.11 mmol, 2.0 equiv). After the tube was evacuated and refilled with argon three times, a mixture of degassed THF/H_2_O 1:1 (0.5 mL) was added, followed by DIPEA (20 μL, 0.11 mmol, 2.0 equiv). The vial was sealed, and the reaction mixture was heated in a microwave reactor at 100 °C for 15 min. The reaction mixture was cooled down to rt, diluted with MeOH (10 mL), and filtered through a 0.45 μm syringe filter. After evaporation of the volatiles under reduced pressure, the crude product was purified using reverse-phase HPLC with a gradient 20–70% MeCN/water (containing 0.1% TFA) to give **22** (9 mg, 16 μmol, 30%) as a colorless solid. HRMS (ESI) *m*/*z*: calcd for C_24_H_31_N_4_O_8_S [M + H]^+^, 535.1863; found, 535.1847. ^1^H NMR (500 MHz, CDCl_3_): δ 8.39 (d, *J* = 8.6 Hz, 1H), 7.88 (dd, *J* = 7.9, 1.6 Hz, 1H), 7.79 (dd, *J* = 7.9, 1.6 Hz, 1H), 7.63 (d, *J* = 9.1 Hz, 1H), 7.21 (t, *J* = 7.9 Hz, 1H), 5.45 (dd, *J* = 11.2, 2.7 Hz, 1H), 5.20 (td, *J* = 9.1, 4.4 Hz, 1H), 4.90 (ddd, *J* = 8.6, 2.7, 2.1 Hz, 1H), 4.74 (d, *J* = 10.3 Hz, 1H), 4.69 (dd, *J* = 7.9, 3.4 Hz, 1H), 4.43 (d, *J* = 10.3 Hz, 1H), 4.37 (dd, *J* = 11.2, 2.1 Hz, 1H), 3.83–3.78 (m, 1H), 3.54–3.49 (m, 1H), 3.09 (s, 3H), 3.07–2.98 (m, 2H), 2.96 (s, 3H), 2.42–2.34 (m, 1H), 2.34–2.25 (m, 1H), 2.24 (s, 3H), 2.07–1.96 (m, 2H). *The COOHproton was not detectable in this spectrum.*^13^C NMR (126 MHz, CDCl_3_): δ 173.0, 171.7, 169.6, 169.1, 168.7, 167.8, 137.4, 134.8, 134.7, 132.5, 131.7, 127.1, 66.4, 60.0, 49.8, 48.5, 53.7, 37.2, 36.1, 36.0, 33.2, 27.9, 25.2, 22.4.

#### (4*S*,7*R*)-7-Acetamido-*N*,*N*-dimethyl-6,10-dioxo-1,3,4,5,6,7,8,10-octahydrobenzo[*j*][1]oxa[8]thia[5]azacyclododecine-4-carboxamide (**23**)

Compound **107**(9) (23 mg, 52 μmol, 1.0 equiv) was dissolved in 4 M HCl in 1,4-dioxane (2 mL) and stirred for 1 h at rt. After evaporation of the volatiles under reduced pressure, the resulting salt was dissolved in DCM (2 mL). Acetyl chloride (11 μL, 0.16 mmol, 3.0 equiv) and triethylamine (28 μL, 0.21 mmol, 4.0 equiv) were added, and the mixture was stirred for 2 h at rt. After evaporation of the volatiles under reduced pressure, the crude product was purified by reverse-phase HPLC with a gradient 25–75% MeCN/water to give **23** (9 mg, 23 μmol, 44% yield over two steps) as a colorless powder. HRMS (ESI) *m*/*z*: calcd for C_18_H_24_N_3_O_5_S [M + H]^+^, 394.1437; found, 394.1442. ^1^H NMR (500 MHz, CDCl_3_): δ 7.87–7.83 (m, 1H), 7.51–7.45 (m, 1H), 7.41 (d, *J* = 7.7 Hz, 1H), 7.38–7.34 (m, 2H), 6.82 (d, *J* = 7.7 Hz, 1H), 5.14 (ddd, *J* = 7.7, 5.3, 5.1 Hz, 1H), 5.04 (dd, *J* = 11.4, 3.1 Hz, 1H), 4.93 (ddd, *J* = 7.7, 3.1, 2.1 Hz, 1H), 4.59 (dd, *J* = 11.4, 2.1 Hz, 1H), 4.11 (d, *J* = 10.4 Hz, 1H), 4.04 (d, *J* = 10.4 Hz, 1H), 3.18 (dd, *J* = 14.4, 5.1 Hz, 1H), 3.09 (s, 3H), 3.05 (dd, *J* = 14.3, 5.3 Hz, 1H), 2.96 (s, 3H), 2.14 (s, 3H). ^13^C NMR (126 MHz, CDCl_3_): δ 170.4, 169.2, 168.9, 168.4, 137.2, 132.5, 131.6, 131.4, 130.1, 127.7, 66.7, 54.2, 48.7, 37.4, 37.0, 36.0, 35.1, 23.4.

#### (4*S*,7*R*)-7-(2-Acetamidoacetamido)-*N*,*N*-dimethyl-6,10-dioxo-1,3,4,5,6,7,8,10-octahydrobenzo[*j*][1]oxa[8]thia[5]azacyclododecine-4-carboxamide (**24**)

Compound **107**(9) (35 mg, 69 μmol, 1.0 equiv) was dissolved in 4 M HCl in 1,4-dioxane (3 mL) and stirred at rt for 1 h. After evaporation of the volatiles under reduced pressure, the resulting salt was dissolved in MeCN (4 mL). Boc-Gly-OH (25 mg, 0.14 mmol, 2.0 equiv), EDC·HCl (27 mg, 0.14 mmol, 2.0 equiv), and DIPEA (48 μL, 0.28 mmol, 4.0 equiv) were added, and the reaction mixture was stirred for 1 h at rt. EtOAc (50 mL) was added, and the mixture was washed with a 1 M aqueous HCl solution (25 mL), saturated aqueous NaHCO_3_ solution (25 mL), and brine (25 mL). The organic phase was dried over MgSO_4_, filtered, and concentrated under reduced pressure. The resulting oil was dissolved in 4 M HCl in 1,4-dioxane (3 mL) and stirred for 1 h at rt. After evaporation of the volatiles under reduced pressure, the resulting salt was dissolved in DCM (3 mL). Acetyl chloride (25 μL, 0.35 mmol, 5.0 equiv) and triethylamine (95 μL, 0.69 mmol, 10 equiv) were added, and the reaction mixture was stirred for 2 h at rt. EtOAc (75 mL) was added, and the organic phase was washed with a 1 M aqueous HCl solution (25 mL) and brine (25 mL). The organic phase was dried over MgSO_4_, filtered, and concentrated under reduced pressure. The crude product was purified by reverse-phase HPLC with a gradient 15–75% MeCN/water to give **24** (6 mg, 14 μmol, 20% yield over four steps) as a colorless powder. HRMS (ESI) *m*/*z*: calcd for C_20_H_26_N_4_NaO_6_S [M + Na]^+^, 473.1471; found, 473.1465. ^1^H NMR (400 MHz, DMSO-*d*_6_): δ 8.50 (d, *J* = 7.4 Hz, 1H), 8.18–8.11 (m, 1H), 7.88 (d, *J* = 8.5 Hz, 1H), 7.54 (t, *J* = 7.4 Hz, 1H), 7.47 (d, *J* = 7.5 Hz, 1H), 7.39 (t, *J* = 7.5 Hz, 1H), 4.96 (td, *J* = 9.9, 5.0 Hz, 1H), 4.82 (dd, *J* = 11.1, 2.5 Hz, 1H), 4.65 (d, *J* = 9.5 Hz, 1H), 4.55 (d, *J* = 7.1 Hz, 1H), 4.27 (dd, *J* = 11.2, 2.0 Hz, 1H), 3.89 (dd, *J* = 16.4, 5.5 Hz, 1H), 3.79–3.68 (m, 2H), 3.05–2.94 (m, 1H), 2.93 (s, 3H), 2.91–2.84 (m, 1H), 2.79 (s, 3H), 1.83 (s, 3H). ^13^C NMR (101 MHz, DMSO-*d*_6_): δ 170.4, 170.3, 169.7, 169.2, 167.3, 138.2, 133.2, 133.0, 132.0, 129.9, 128.1, 66.9, 53.2, 50.7, 42.7, 38.8, 36.9, 35.8, 35.4, 22.8.

#### (4*S*,7*R*)-7-((*S*)-1-Acetylazetidine-2-carboxamido)-*N*,*N*-dimethyl-6,10-dioxo-1,3,4,5,6,7,8,10-octahydrobenzo[*j*][1]oxa[8]thia[5]azacyclododecine-4-carboxamide (**25**)

Compound **107**(9) (37 mg, 84 μmol, 1.0 equiv) was dissolved in 4 M HCl in 1,4-dioxane (3 mL) and stirred for 1 h at rt. After evaporation of the volatiles under reduced pressure, the resulting salt was dissolved in MeCN (3 mL). (*S*)-1-Boc-azetidine-2-carboxylic acid (34 mg, 0.17 mmol, 2.0 equiv), EDC·HCl (32 mg, 0.17 mmol, 2.0 equiv), and DIPEA (60 μL, 0.34 mmol, 4.0 equiv) were added, and the reaction mixture was stirred for 1 h at rt. EtOAc (75 mL) was added, and the mixture was washed with a 1 M aqueous HCl solution (25 mL), saturated aqueous NaHCO_3_ solution (25 mL), and brine (25 mL). The organic phase was dried over MgSO_4_, filtered, and concentrated under reduced pressure. The resulting oil was dissolved in 4 M HCl in 1,4-dioxane (3 mL) and stirred for 1 h at rt. After evaporation of the volatiles under reduced pressure, the resulting salt was dissolved in DCM (3 mL). Acetyl chloride (30 μL, 0.41 mmol, 5.0 equiv) and pyridine (67 μL, 0.82 mmol, 10 equiv) were added, and the reaction mixture was stirred for 2 h at rt. EtOAc (75 mL) was added, and the mixture was washed with a 1 M aqueous HCl solution (50 mL) and brine (50 mL). The organic phase was dried over MgSO_4_, filtered, and concentrated under reduced pressure. The crude product was purified by reverse-phase HPLC with a gradient 15–75% MeCN/water to give **25** (6 mg, 13 μmol, 16% yield over 4 steps) as a colorless powder. HRMS (ESI) *m*/*z*: calcd for C_22_H_29_N_4_O_6_S_2_ [M + H]^+^, 477.1808; found, 477.1799. ^1^H NMR (500 MHz, CDCl_3_): δ 8.60 (d, *J* = 9.1 Hz, 1H), 7.99 (dd, *J* = 7.9, 1.5 Hz, 1H), 7.45 (td, *J* = 7.7, 1.5 Hz, 1H), 7.41 (d, *J* = 8.9 Hz, 1H), 7.37 (dd, *J* = 7.6, 1.4 Hz, 1H), 7.33 (td, *J* = 7.6, 1.4 Hz, 1H), 5.18 (td, *J* = 9.2, 4.2 Hz, 1H), 5.14 (dd, *J* = 11.1, 2.6 Hz, 1H), 4.96 (dt, *J* = 9.1, 2.4 Hz, 1H), 4.90 (dd, *J* = 9.1, 5.8 Hz, 1H), 4.60 (d, *J* = 10.1 Hz, 1H), 4.44 (dd, *J* = 11.1, 2.2 Hz, 1H), 4.29–4.24 (m, 1H), 4.12–4.05 (m, 1H), 3.88 (d, *J* = 10.1 Hz, 1H), 3.08 (s, 3H), 3.03 (dd, *J* = 14.7, 4.2 Hz, 1H), 2.93 (s, 3H), 2.87 (dd, *J* = 14.7, 9.4 Hz, 1H), 2.81–2.73 (m, 1H), 2.47–2.37 (m, 1H), 1.94 (s, 3H). ^13^C NMR (126 MHz, CDCl_3_): δ 173.4, 170.0, 169.6, 168.5, 167.6, 137.3, 132.6, 132.3, 132.3, 129.6, 127.6, 66.9, 62.7, 53.2, 49.9, 49.2, 38.6, 37.1, 35.9, 35.8, 18.8, 17.4.

#### (4*S*,7*R*)-7-((*S*)-1-Acetylpiperidine-2-carboxamido)-*N*,*N*-dimethyl-6,10-dioxo-1,3,4,5,6,7,8,10-octahydrobenzo[*j*][1]oxa[8]thia[5]azacyclododecine-4-carboxamide (**26**)

Compound **107**(9) (29 mg, 64 μmol, 1.0 equiv) was dissolved in 4 M HCl in 1,4-dioxane (3 mL) and stirred for 1 h at rt. After evaporation of the volatiles under reduced pressure, the resulting salt was dissolved in MeCN (3 mL). (*S*)-1-*N*-Boc-Pipecolinic acid (30 mg, 0.13 mmol, 2.0 equiv), EDC·HCl (25 mg, 0.13 mmol, 2.0 equiv), and DIPEA (45 μL, 0.26 mmol, 4.0 equiv) were added, and the reaction mixture was stirred for 1 h at rt. EtOAc (50 mL) was added, and the organic phase was washed with a 1 M aqueous HCl solution (25 mL), saturated aqueous NaHCO_3_ solution (25 mL), and brine (25 mL). The organic phase was dried over MgSO_4_, filtered, and concentrated under reduced pressure. The resulting oil was dissolved in 4 M HCl in 1,4-dioxane (3 mL) and stirred for 1 h at rt. After evaporation of the volatiles under reduced pressure, the resulting salt was dissolved in DCM (2 mL). Acetyl chloride (23 μL, 0.32 mmol, 5.0 equiv) and pyridine (52 μL, 0.64 mmol, 10 equiv) were added, and the reaction mixture was stirred for 2 h at rt. EtOAc (50 mL) was added, and the mixture was washed with a 1 M aqueous HCl solution (50 mL) and brine (50 mL). The organic phase was dried over MgSO_4_, filtered, and concentrated under reduced pressure. The crude product was purified by reverse-phase HPLC with a gradient 20–75% MeCN/water to give **26** (8 mg, 16 μmol, 25% yield over four steps) as a colorless powder. HRMS (ESI) *m*/*z*: calcd for C_24_H_33_N_4_O_6_S [M + H]^+^, 505.2121; found, 505.2112. ^1^H NMR (500 MHz, CDCl_3_): δ 7.96–7.89 (m, 1H), 7.72 (d, *J* = 7.6 Hz, 1H), 7.49–746 (m, 1H), 7.38–7.31 (m, 3H), 5.18–5.11 (m, 2H), 5.05 (dd, *J* = 11.4, 3.0 Hz, 1H), 4.82 (ddd, *J* = 7.6, 2.5, 2.0 Hz, 1H), 4.58 (dd, *J* = 11.4, 2.0 Hz, 1H), 4.26 (d, *J* = 10.3 Hz, 1H), 4.06 (d, *J* = 10.3 Hz, 1H), 3.88–3.81 (m, 1H), 3.37–3.29 (m, 1H), 3.11 (dd, *J* = 14.5, 4.7 Hz, 1H), 3.08 (s, 3H), 2.97–2.91 (m, 1H), 2.93 (s, 3H), 2.31–2.25 (m, 1H), 2.25 (s, 3H), 1.84–1.75 (m, 2H), 1.71–1.48 (m, 3H). ^13^C NMR (126 MHz, CDCl_3_): δ 172.3, 171.6, 169.1, 168.6, 168.3, 137.4, 132.6, 131.9, 131.9, 129.8, 127.7, 66.8, 53.9, 52.5, 49.1, 44.7, 37.9, 37.0, 35.8, 35.4, 25.1, 24.8, 21.8, 20.0.

#### (4*S*,7*R*)-7-((*R*)-3-Acetylthiazolidine-4-carboxamido)-*N*,*N*-dimethyl-6,10-dioxo-1,3,4,5,6,7,8,10-octahydrobenzo[*j*][1]oxa[8]thia[5]azacyclododecine-4-carboxamide (**27**)

Compound **107**(9) (29 mg, 64 μmol, 1.0 equiv) was dissolved in 4 M HCl in 1,4-dioxane (3 mL) and stirred for 1 h at rt. After evaporation of the volatiles under reduced pressure, the resulting salt was dissolved in MeCN. (4*R*)-3-[(*tert*-Butoxy)carbonyl]-1,3-thiazolidine-4-carboxylic acid (31 mg, 0.13 mmol, 2.0 equiv), EDC·HCl (25 mg, 0.13 mmol, 2.0 equiv), and DIPEA (45 μL, 0.26 mmol, 4.0 equiv) were added, and the reaction mixture was stirred for 1 h at rt. EtOAc (50 mL) was added, and the mixture was washed with a 1 M aqueous HCl solution (25 mL), saturated aqueous NaHCO_3_ solution (25 mL), and brine (25 mL). The organic phase was dried over MgSO_4_, filtered, and concentrated under reduced pressure. The resulting oil was dissolved in 4 M HCl in 1,4-dioxane (3 mL) and stirred for 1 h at rt. After evaporation of the volatiles under reduced pressure, the resulting salt was dissolved in DCM (2 mL). Acetyl chloride (23 μL, 0.32 mmol, 5.0 equiv) and pyridine (52 μL, 0.64 mmol, 10 equiv) were added, and the reaction mixture was stirred for 2 h at rt. EtOAc (50 mL) was added, and the mixture was washed with a 1 M aqueous HCl solution (50 mL) and brine (50 mL). The organic phase was dried over MgSO_4_, filtered, and concentrated under reduced pressure. The crude product was purified by reverse-phase HPLC with a gradient 15–75% MeCN/water to give **27** (7 mg, 14 μmol, 22% yield over 4 steps) as a colorless powder. HRMS (ESI) *m*/*z*: calcd for C_22_H_29_N_4_O_6_S_2_ [M + H]^+^, 509.1529; found, 509.1521. ^1^H NMR (500 MHz, CDCl_3_): δ 7.99 (d, *J* = 8.1 Hz, 1H), 7.88 (dd, *J* = 7.8, 1.5 Hz, 1H), 7.50–7.42 (m, 2H), 7.39–7.31 (m, 2H), 5.12 (ddd, *J* = 8.8, 7.3, 4.9 Hz, 1H), 5.03 (dd, *J* = 11.3, 2.9 Hz, 1H), 4.95 (dd, *J* = 6.8, 3.7 Hz, 1H), 4.82 (ddd, *J* = 8.1, 2.9, 2.0 Hz, 1H), 4.67 (d, *J* = 8.7 Hz, 1H), 4.64–4.56 (m, 2H), 4.18 (d, *J* = 10.2 Hz, 1H), 4.03 (d, *J* = 10.2 Hz, 1H), 3.51 (dd, *J* = 11.9, 3.7 Hz, 1H), 3.15 (dd, *J* = 11.8, 6.8 Hz, 1H), 3.06 (s, 3H), 3.02 (dd, *J* = 10.5, 5.0 Hz, 1H), 2.91 (s, 3H), 2.22 (s, 3H). ^13^C NMR (126 MHz, CDCl_3_): δ 170.8, 169.9, 169.2, 168.9, 168.2, 137.1, 132.6, 131.9, 131.6, 130.0, 127.7, 66.6, 62.5, 54.1, 50.1, 49.2, 37.6, 37.1, 35.9, 35.4, 32.5, 22.8.

#### *tert*-Butyl-(*S*)-2-(((4*S*,7*R*)-4-(dimethylcarbamoyl)-6,10-dioxo-1,3,4,5,6,7,8,10-octahydrobenzo[*j*][1]oxa[8]thia[5]azacyclododecin-7-yl)carbamoyl)pyrrolidine-1-carboxylate (**108**)

Compound **107**(9) (800 mg, 1.77 mmol, 1.0 equiv) was dissolved in 4 M HCl in 1,4-dioxane (10 mL) and stirred for 1 h at rt. After evaporation of the volatiles under reduced pressure, the resulting salt was dissolved in MeCN (20 mL). Boc-l-Pro-OH (762 mg, 3.54 mmol, 2.0 equiv), HATU (1.35 g, 3.54 mmol, 2.0 equiv), and DIPEA (1.22 mL, 7.01 mmol, 4.0 equiv) were added, and the reaction mixture was stirred for 2 h at rt. EtOAc (100 mL) was added, and the mixture was washed with a 1 M aqueous HCl solution (50 mL), saturated aqueous NaHCO_3_ solution (50 mL), and brine (50 mL). The organic phase was dried over MgSO_4_, filtered, and concentrated under reduced pressure. The crude reaction mixture was purified by flash chromatography on a silica gel column using 1–5% MeOH/DCM as the eluent to give **108** (720 mg, 1.31 mmol, 74% yield over two steps) as a colorless powder. HRMS (ESI) *m*/*z*: calcd for C_26_H_37_N_4_O_7_S [M + H]^+^, 549.2383; found, 549.2391. ^1^H NMR (500 MHz, CDCl_3_): δ 7.76 (d, *J* = 7.8 Hz, 1H), 7.64–7.59 (m, 1H), 7.59–7.51 (m, 1H), 7.38 (td, *J* = 7.6, 1.8 Hz, 1H), 7.30 (d, *J* = 7.3 Hz, 1H), 7.26 (t, *J* = 7.6 Hz, 1H), 5.09 (d, *J* = 10.4 Hz, 1H), 5.06–4.97 (m, 1H), 4.89 (d, *J* = 8.1 Hz, 1H), 4.34 (d, *J* = 10.2 Hz, 1H), 4.13–4.05 (m, 1H), 3.95 (d, *J* = 4.6 Hz, 1H), 3.82 (d, *J* = 8.9 Hz, 1H), 3.50–3.42 (m, 1H), 3.34–3.27 (m, 1H), 3.11–3.03 (m, 2H), 3.01 (s, 3H), 2.84 (s, 3H), 2.25–2.15 (m, 1H), 1.98–1.91 (m, 2H), 1. 85–1.76 (m, 1H), 1.30 (s, 9H). ^13^C NMR (126 MHz, CDCl_3_): δ 172.8, 169.5, 169.0, 168.1, 155.7, 136.8, 132.3, 132.1, 131.3, 130.5, 127.6, 80.8, 66.7, 60.8, 53.2, 49.4, 47.3, 37.0, 36.9, 36.0, 35.8, 28.9, 28.3, 24.7.

#### (4*S*,7*R*)-7-((*S*)-1-(Cyclopropanecarbonyl)pyrrolidine-2-carboxamido)-*N*,*N*-dimethyl-6,10-dioxo-1,3,4,5,6,7,8,10-octahydrobenzo[*j*][1]oxa[8]thia[5]azacyclododecine-4-carboxamide (**28**)

Compound **108** (25 mg, 46 μmol, 1.0 equiv) was dissolved in 4 M HCl in 1,4-dioxane (3 mL) and stirred for 1 h at rt. After evaporation of the volatiles under reduced pressure, the resulting salt was dissolved in DMSO (1 mL). Cyclopropanecarboxylic acid (15 μL, 0.18 mmol, 4.0 equiv), HATU (35 mg, 92 μmol, 2.0 equiv), and DIPEA (40 μL, 0.23 mmol, 5.0 equiv) were added, and the reaction mixture was stirred for 1 h at rt. EtOAc (75 mL) was added and the mixture was washed with a 1 M aqueous HCl solution (25 mL), saturated aqueous NaHCO_3_ solution (25 mL), and brine (25 mL). The organic phase was dried over MgSO_4_, filtered, and concentrated under reduced pressure. The crude product was purified using reverse-phase HPLC with a gradient 15% to 80% MeCN in water to give **28** (8 mg, 16 μmol, 34% yield over two steps) as a colorless powder. HRMS (ESI) *m*/*z*: calcd for C_25_H_33_N_4_O_6_S [M + H]^+^, 517.2121; found, 517.2114. ^1^H NMR (500 MHz, CDCl_3_): δ 7.92 (dd, *J* = 7.6, 1.4 Hz, 1H), 7.75 (d, *J* = 8.8 Hz, 1H), 7.73 (d, *J* = 9.0 Hz, 1H), 7.48 (td, *J* = 7.5, 1.5 Hz, 1H), 7.40 (dd, *J* = 7.5, 1.5 Hz, 1H), 7.36 (td, *J* = 7.6, 1.4 Hz, 1H), 5.20 (dd, *J* = 10.9, 2.5 Hz, 1H), 5.13 (td, *J* = 9.0, 5.5 Hz, 1H), 4.89 (dt, *J* = 8.8, 2.5 Hz, 1H), 4.44–4.37 (m, 2H), 4.14 (d, *J* = 9.8 Hz, 1H), 3.95 (d, *J* = 9.8 Hz, 1H), 3.95–3.88 (m, 1H), 3.72–3.66 (m, 1H), 3.16–3.04 (m, 2H), 3.09 (s, 3H), 2.95 (s, 3H), 2.40–2.33 (m, 1H), 2.30–2.17 (m, 1H), 2.08–1.94 (m, 2H), 1.73–1.65 (m, 1H), 1.02–0.89 (m, 2H), 0.84–0.69 (m, 2H). ^13^C NMR (126 MHz, CDCl_3_): δ 174.5, 172.1, 169.2, 169.2, 167.7, 137.0, 132.5, 132.3, 131.7, 130.1, 127.6, 66.6, 60.9, 53.1, 49.6, 47.6, 37.7, 37.0, 36.0, 36.0, 27.8, 25.2, 12.7, 8.3, 8.1.

#### (4*S*,7*R*)-7-((*S*)-1-Formylpyrrolidine-2-carboxamido)-*N*,*N*-dimethyl-6,10-dioxo-1,3,4,5,6,7,8,10-octahydrobenzo[*j*][1]oxa[8]thia[5]azacyclododecine-4-carboxamide (**29**)

Compound **108** (25 mg, 46 μmol, 1.0 equiv) was dissolved in 4 M HCl in 1,4-dioxane (3 mL) and stirred for 1 h at rt. After evaporation of the volatiles under reduced pressure, the resulting salt was dissolved in DMSO (1 mL). Formic acid (18 μL, 0.46 mmol, 10 equiv), HATU (35 mg, 92 μmol, 2.0 equiv), and DIPEA (95 μL, 0.55 mmol, 12 equiv) were added, and the reaction mixture was stirred for 1 h at rt. EtOAc (50 mL) was added, and the mixture was washed with a 1 M aqueous HCl solution (25 mL), saturated aqueous NaHCO_3_ solution (25 mL), and brine (25 mL). The organic phase was dried over MgSO_4_, filtered, and concentrated under reduced pressure. The crude product was purified using reverse-phase HPLC with a gradient 20–75% MeCN/water to give **29** (6 mg, 13 μmol, 28% yield over two steps) as a colorless powder. HRMS (ESI) *m*/*z*: calcd for C_22_H_29_N_4_O_6_S [M + H]^+^, 477.1808; found, 477.1812. ^1^H NMR (500 MHz, CDCl_3_): δ 8.39 (s, 1H), 7.98 (dd, *J* = 7.8, 1.5 Hz, 1H), 7.88 (d, *J* = 8.9 Hz, 1H), 7.53 (d, *J* = 9.2 Hz, 1H), 7.49 (td, *J* = 7.5, 1.5 Hz, 1H), 7.41 (dd, *J* = 7.5, 1.5 Hz, 1H), 7.36 (td, *J* = 7.8, 1.5 Hz, 1H), 5.22–5.14 (m, 2H), 4.93 (dt, *J* = 8.9, 2.4 Hz, 1H), 4.50–4.42 (m, 3H), 3.95 (d, *J* = 9.9 Hz, 1H), 3.75–3.70 (m, 1H), 3.69–3.64 (m, 1H), 3.09 (s, 3H), 3.08–3.04 (m, 1H), 2.99 (dd, *J* = 14.8, 9.4 Hz, 1H), 2.95 (s, 3H), 2.51–2.43 (m, 1H), 2.22–2.15 (m, 1H), 2.11–2.04 (m, 1H), 1.98–1.92 (m, 1H). ^13^C NMR (126 MHz, CDCl_3_): δ 170.3, 169.6, 168.6, 167.8, 162.8, 137.2, 132.7, 132.4, 132.1, 129.7, 127.7, 66.8, 58.3, 53.4, 49.9, 47.0, 38.3, 37.2, 35.9, 35.7, 27.3, 24.5.

#### (4*S*,7*R*)-*N*,*N*-Dimethyl-6,10-dioxo-7-((*S*)-1-(2,2,2-trifluoroacetyl)pyrrolidine-2-carboxamido)-1,3,4,5,6,7,8,10-octahydrobenzo[*j*][1]oxa[8]thia[5]azacyclododecine-4-carboxamide (**30**)

Compound **108** (25 mg, 46 μmol, 1.0 equiv) was dissolved in 4 M HCl in 1,4-dioxane (3 mL) and stirred for 1 h at rt. After evaporation of the volatiles under reduced pressure, the resulting salt was dissolved in DCM (2 mL). Trifluoroacetic anhydride (19 μL, 0.14 mmol, 3.0 equiv) and triethylamine (67 μL, 0.46 mmol, 10 equiv) were added, and the mixture was stirred for 2 h at rt. EtOAc (50 mL) was added, and the mixture was washed with a 1 M aqueous HCl solution (25 mL). The organic phase was dried over MgSO_4_, filtered, and concentrated under reduced pressure. The crude product was purified using reverse-phase HPLC with a gradient 15–80% MeCN/water to give **30** (8 mg, 15 μmol, 32% yield over two steps) as a colorless powder. HRMS (ESI) *m*/*z*: calcd for C_23_H_28_F_3_N_4_O_6_S [M + H]^+^, 545.1682; found, 545.1663. ^1^H NMR (500 MHz, CDCl_3_): δ 7.91 (dd, *J* = 7.8, 1.5 Hz, 1H), 7.52 (d, *J* = 8.0 Hz, 1H), 7.49 (td, *J* = 7.5, 1.5 Hz, 1H), 7.44–7.34 (m, 3H), 5.17–5.08 (m, 2H), 4.86 (ddd, *J* = 8.0, 2.4, 2.0 Hz, 1H), 4.54 (dd, *J* = 11.3, 2.0 Hz, 1H), 4.50 (dd, *J* = 8.0, 4.1 Hz, 1H), 4.23 (d, *J* = 9.9 Hz, 1H), 3.99 (d, *J* = 9.9 Hz, 1H), 3.97–3.92 (m, 1H), 3.81–3.74 (m, 1H), 3.12 (dd, *J* = 14.9, 4.9 Hz, 1H), 3.04 (s, 3H), 3.02–2.96 (m, 1H), 2.92 (s, 3H), 2.37–2.29 (m, 2H), 2.16–2.12 (m, 1H), 2.09–2.02 (m, 1H). ^13^C NMR (126 MHz, CDCl_3_): δ 170.2, 168.9, 168.4, 168.1, 157.3 (q, *J* = 38 Hz), 137.1, 132.7, 132.2, 131.7, 129.8, 127.7, 116.1 (q, *J* = 287 Hz), 66.5, 62.2, 53.9, 49.8, 47.8, 37.9, 36.9, 35.8, 35.5, 27.8, 25.4.

#### Methyl-(*S*)-2-(((4*S*,7*R*)-4-(dimethylcarbamoyl)-6,10-dioxo-1,3,4,5,6,7,8,10-octahydrobenzo[*j*][1]oxa[8]thia[5]azacyclododecin-7-yl)carbamoyl)pyrrolidine-1-carboxylate (**31**)

Compound **108** (25 mg, 46 μmol, 1.0 equiv) was dissolved in 4 M HCl in 1,4-dioxane (3 mL) and stirred for 1 h at rt. After evaporation of the volatiles under reduced pressure, the resulting salt was dissolved in DCM (2 mL). Methyl chloroformate (18 μL, 0.23 mmol, 5.0 equiv) and triethylamine (65 μL, 0.46 mmol, 10 equiv) were added, and the mixture was stirred for 2 h at rt. EtOAc (50 mL) was added, and the mixture was washed with a 1 M aqueous HCl solution (25 mL). The organic phase was dried over MgSO_4_, filtered, and concentrated under reduced pressure. The crude product was purified using reverse-phase HPLC with a gradient 15–80% MeCN/water to give **31** (10 mg, 20 μmol, 43% yield over two steps) as a colorless powder. HRMS (ESI) *m*/*z*: calcd for C_23_H_31_N_4_O_7_S [M + H]^+^, 507.1913; found, 507.1911. ^1^H NMR (500 MHz, CDCl_3_): δ 7.93 (d, *J* = 7.8 Hz, 1H), 7.87 (d, *J* = 9.5 Hz, 1H), 7.51–7.40 (m, 3H), 7.36 (t, *J* = 7.8 Hz, 1H), 5.19 (dd, *J* = 11.0, 2.5 Hz, 1H), 5.15 (dt, *J* = 9.0, 4.7 Hz, 1H), 4.96–4.91 (m, 1H), 4.47 (dd, *J* = 11.0, 2.5 Hz, 1H), 4.22–4.14 (m, 2H), 3.97 (d, *J* = 9.8 Hz, 1H), 3.74 (s, 3H), 3.58–3.52 (m, 1H), 3.50–3.42 (m, 1H), 3.19 (dd, *J* = 14.9, 9.8 Hz, 1H), 3.11 (s, 3H), 3.10–3.07 (m, 1H), 2.94 (s, 3H), 2.33–2.26 (m, 1H), 2.19–2.11 (m, 1H), 2.09–2.00 (m, 1H), 1.93–1.82 (m, 1H). ^13^C NMR (126 MHz, CDCl_3_): δ 172.0, 169.2, 169.0, 167.9, 156.9, 136.9, 132.6, 132.4, 131.8, 130.0, 127.7, 66.7, 60.9, 53.4, 53.3, 49.7, 46.9, 37.7, 37.1, 35.9, 35.7, 28.7, 25.0.

#### (4*S*,7*R*)-7-((*S*)-1-(2-Methoxyacetyl)pyrrolidine-2-carboxamido)-*N*,*N*-dimethyl-6,10-dioxo-1,3,4,5,6,7,8,10-octahydrobenzo[*j*][1]oxa[8]thia[5]azacyclododecine-4-carboxamide (**32**)

Compound **108** (25 mg, 46 μmol, 1.0 equiv) was dissolved in 4 M HCl in 1,4-dioxane (3 mL) and stirred for 1 h at rt. After evaporation of the volatiles under reduced pressure, the resulting salt was dissolved in DCM (2 mL). Methoxyacetyl chloride (17 μL, 0.19 mmol, 4.0 equiv) and triethylamine (65 μL, 0.46 mmol, 10 equiv) were added, and the mixture was stirred for 2 h at rt. EtOAc (50 mL) was added, and the mixture was washed with a 1 M aqueous HCl solution (25 mL). The organic phase was dried over MgSO_4_, filtered, and concentrated under reduced pressure. The crude product was purified using reverse-phase HPLC with a gradient 20–75% MeCN/water to give **32** (6 mg, 12 μmol, 26% yield over two steps) as a colorless powder. HRMS (ESI) *m*/*z*: calcd for C_24_H_32_N_4_NaO_7_S [M + H]^+^, 543.1889; found, 543.1900. ^1^H NMR (500 MHz, CDCl_3_): δ 8.10 (d, *J* = 8.7 Hz, 1H), 7.98 (dd, *J* = 7.9, 1.5 Hz, 1H), 7.52–7.32 (m, 4H), 5.17 (dd, *J* = 11.0, 2.5 Hz, 1H), 5.15–5.10 (m, 1H), 4.86 (dt, *J* = 8.7, 2.5 Hz, 1H), 4.61 (dd, *J* = 7.9, 2.9 Hz, 1H), 4.57–4.44 (m, 2H), 4.28 (d, *J* = 14.6 Hz, 1H), 4.16 (d, *J* = 14.6 Hz, 1H), 4.02–3.93 (m, 1H), 3.78–3.71 (m, 1H), 3.55–3.46 (m, 1H), 3.37 (s, 3H), 3.12–3.03 (m, 1H), 3.07 (s, 3H), 3.03–2.94 (m, 1H), 2.93 (s, 3H), 2.46–2.39 (m, 1H), 2.27–2.16 (m, 1H), 2.06–1.97 (m, 1H), 1.95–1.87 (m, 1H). ^13^C NMR (126 MHz, CDCl_3_): δ 171.1, 170.9, 169.3, 168.5, 167.8, 137.4, 132.6, 132.2, 132.1, 129.7, 127.6, 71.6, 71.5, 66.6, 60.3, 59.3, 53.4, 46.4, 38.2, 37.0, 35.8, 35.6, 26.8, 25.3.

#### (4*S*,7*R*)-7-((*S*)-1-Benzoylpyrrolidine-2-carboxamido)-*N*,*N*-dimethyl-6,10-dioxo-1,3,4,5,6,7,8,10-octahydrobenzo[*j*][1]oxa[8]thia[5]azacyclododecine-4-carboxamide (**33**)

Compound **108** (30 mg, 54 μmol, 1.0 equiv) was dissolved in 4 M HCl in 1,4-dioxane (2 mL) and stirred for 1 h at rt. After evaporation of the volatiles under reduced pressure, the resulting salt was dissolved in DCM (2 mL). Benzoyl chloride (25 μL, 0.22 mmol, 4.0 equiv) and triethylamine (45 μL, 0.32 mmol, 6.0 equiv) were added, and the reaction mixture was stirred for 2 h at rt. EtOAc (50 mL) was added, and the mixture was washed with a 1 M aqueous HCl solution (25 mL). The organic phase was dried over MgSO_4_, filtered, and concentrated under reduced pressure. The crude product was purified using reverse-phase HPLC with a gradient 20–75% MeCN/water to give **33** (10 mg, 18 μmol, 32% yield over two steps) as a colorless powder. HRMS (ESI) *m*/*z*: calcd for C_28_H_32_N_4_NaO_6_S [M + Na]^+^, 575.1940; found, 575.1942. ^1^H NMR (400 MHz, CDCl_3_): δ 7.88 (dd, *J* = 8.2, 1.3 Hz, 1H), 7.87–7.74 (m, 2H), 7.67–7.61 (m, 2H), 7.48–7.34 (m, 4H), 7.31–7.27 (m, 2H), 5.18 (dd, *J* = 11.1, 2.5 Hz, 1H), 5.11 (td, *J* = 8.9, 4.7 Hz, 1H), 4.87 (ddd, *J* = 8.3, 2.5, 2.0 Hz, 1H), 4.66–4.60 (m, 1H), 4.50 (dd, *J* = 11.1, 2.0 Hz, 1H), 4.24 (d, *J* = 10.0 Hz, 1H), 3.80–3.75 (m, 1H), 3.76 (d, *J* = 10.0 Hz, 1H), 3.62–3.54 (m, 1H), 3.05 (dd, *J* = 14.8, 4.7 Hz, 1H), 3.00 (s, 3H), 2.96 (dd, *J* = 14.8, 8.9 Hz, 1H), 2.76 (s, 3H), 2.41–2.32 (m, 1H), 2.20–2.09 (m, 2H), 1.92–1.82 (m, 1H). ^13^C NMR (101 MHz, CDCl_3_): δ 172.0, 171.1, 169.1, 168.7, 168.0, 137.2, 135.2, 132.5, 132.1, 131.8, 130.6, 129.8, 128.1, 127.9, 127.6, 66.5, 61.1, 53.6, 50.6, 49.9, 38.0, 37.0, 35.7, 35.7, 27.9, 25.7.

#### (4*S*,7*R*)-*N*,*N*-Dimethyl-6,10-dioxo-7-((*S*)-1-(2-phenylacetyl)pyrrolidine-2-carboxamido)-1,3,4,5,6,7,8,10-octahydrobenzo[*j*][1]oxa[8]thia[5]azacyclododecine-4-carboxamide (**34**)

Compound **108** (27 mg, 50 μmol, 1.0 equiv) was dissolved in 4 M HCl in 1,4-dioxane (2 mL) and stirred for 1 h at rt. After evaporation of the volatiles under reduced pressure, the resulting salt was dissolved in DCM (2 mL). Phenylacetyl chloride (23 μL, 0.20 mmol, 4.0 equiv) and triethylamine (43 μL, 0.30 mmol, 6.0 equiv) were added, and the reaction mixture was stirred for 2 h at rt. EtOAc (50 mL) was added, and the mixture was washed with a 1 M aqueous HCl solution (25 mL). The organic phase was dried over MgSO_4_, filtered, and concentrated under reduced pressure. The crude product was purified using reverse-phase HPLC with a gradient 20–75% MeCN/water to give **34** (12 mg, 20 μmol, 42% yield over two steps) as a colorless powder. HRMS (ESI) *m*/*z*: calcd for C_29_H_35_N_4_O_6_S [M + H]^+^, 567.2277; found, 567.2283. ^1^H NMR (400 MHz, CDCl_3_): δ 8.06 (d, *J* = 9.0 Hz, 1H), 7.94 (dd, *J* = 7.8, 1.2 Hz, 1H), 7.50 (d, *J* = 9.4 Hz, 1H), 7.44 (td, *J* = 7.5, 1.5 Hz, 1H), 7.32 (dd, *J* = 7.5, 1.5 Hz, 1H), 7.29–7.24 (m, 6H), 5.14 (dd, *J* = 11.0, 2.6 Hz, 1H), 5.13–5.08 (m, 1H), 4.84 (ddd, *J* = 9.0, 2.6, 2.1 Hz, 1H), 4.58–4.45 (m, 1H), 4.40–4 .35 (m, 1H), 4.39 (dd, *J* = 11.0, 2.1 Hz, 1H), 3.87 (d, *J* = 15.7 Hz, 1H), 3.84–3.77 (m, 1H), 3.74 (d, *J* = 15.7 Hz, 1H), 3.57–3.46 (m, 2H), 3.06 (s, 3H), 2.99 (dd, *J* = 13.9, 5.2 Hz, 1H), 2.93 (s, 3H), 2.80 (dd, *J* = 13.9, 9.7 Hz, 1H), 2.44–2.32 (m, 1H), 2.30–2.11 (m, 1H), 2.02–1.87 (m, 2H). ^13^C NMR (101 MHz, CDCl_3_): δ 172.4, 171.1, 169.4, 168.6, 167.7, 137.3, 134.3, 132.5, 132.2, 132.1, 129.6, 129.6, 129.5, 128.4, 128.4, 127.6, 126.8, 66.7, 60.4, 53.2, 49.9, 47.8, 41.5, 38.3, 37.1, 35.8, 35.6, 27.3, 25.3.

#### (4*S*,7*R*)-*N*,*N*-Dimethyl-7-((*S*)-1-(methylsulfonyl)pyrrolidine-2-carboxamido)-6,10-dioxo-1,3,4,5,6,7,8,10-octahydrobenzo[*j*][1]oxa[8]thia[5]azacyclododecine-4-carboxamide (**35**)

Compound **108** (30 mg, 54 μmol, 1.0 equiv) was dissolved in 4 M HCl in 1,4-dioxane (2 mL) and stirred for 1 h at rt. After evaporation of the volatiles under reduced pressure, the resulting salt was dissolved in DCM (2 mL). Methanesulfonyl chloride (17 μL, 0.22 mmol, 4.0 equiv) and triethylamine (38 μL, 0.27 mmol, 5.0 equiv) were added, and the reaction mixture was stirred for 2 h at rt. EtOAc (50 mL) was added, and the mixture was washed with a 1 M aqueous HCl solution (25 mL). The organic phase was dried over MgSO_4_, filtered, and concentrated under reduced pressure. The crude product was purified using reverse-phase HPLC with a gradient 20–75% MeCN/water to give **35** (11 mg, 20 μmol, 37% yield over two steps) as a colorless powder. HRMS (ESI) *m*/*z*: calcd for C_22_H_31_N_4_O_7_S_2_ [M + H]^+^, 527.1634; found, 527.1637. ^1^H NMR (400 MHz, CDCl_3_): δ 7.89 (dd, *J* = 7.7, 1.3 Hz, 1H), 7.82 (d, *J* = 7.7 Hz, 1H), 7.48–7.40 (m, 2H), 7.33 (td, *J* = 8.6, 1.5 Hz, 2H), 5.14 (dt, *J* = 8.0, 2.8 Hz, 1H), 5.08 (dd, *J* = 11.4, 2.9 Hz, 1H), 4.86–4.76 (m, 1H), 4.56 (dd, *J* = 11.4, 2.0 Hz, 1H), 4.29 (dd, *J* = 7.8, 3.9 Hz, 1H), 4.21 (d, *J* = 10.3 Hz, 1H), 4.02 (d, *J* = 10.3 Hz, 1H), 3.64 (ddd, *J* = 9.5, 6.7, 4.6 Hz, 1H), 3.43–3.34 (m, 1H), 3.09 (dd, *J* = 14.6, 4.5 Hz, 1H), 3.07 (s, 3H), 2.97–2.93 (m, 1H), 2.95, (s, 3H), 2.91 (s, 3H), 2.45–2.34 (m, 1H), 2.16–2.06 (m, 2H), 2.05–1.95 (m, 1H). ^13^C NMR (101 MHz, CDCl_3_): δ 171.5, 169.2, 168.3, 168.2, 137.2, 132.5, 131.9, 131.7, 129.8, 127.6, 66.4, 61.6, 54.0, 49.4, 49.3, 37.5, 37.0, 36.6, 35.8, 35.3, 29.8, 25.4.

#### (4*S*,7*R*)-*N*,*N*-Dimethyl-6,10-dioxo-7-((*S*)-1-(phenylsulfonyl)pyrrolidine-2-carboxamido)-1,3,4,5,6,7,8,10-octahydrobenzo[*j*][1]oxa[8]thia[5]azacyclododecine-4-carboxamide (**36**)

Compound **108** (22 mg, 40 μmol, 1.0 equiv) was dissolved in 4 M HCl in 1,4-dioxane (2 mL) and stirred for 1 h at rt. After evaporation of the volatiles under reduced pressure, the resulting salt was dissolved in DCM (2 mL). Benzenesulfonyl chloride (15 μL, 0.12 mmol, 3.0 equiv) and triethylamine (28 μL, 0.20 mmol, 5.0 equiv) were added, and the reaction mixture was stirred for 2 h at rt. EtOAc (50 mL) was added, and the mixture was washed with a 1 M aqueous HCl solution (25 mL). The organic phase was dried over MgSO_4_, filtered, and concentrated under reduced pressure. The crude product was purified using reverse-phase HPLC with a gradient 20–75% MeCN/water to give **36** (7 mg, 12 μmol, 30% yield) as a colorless powder. HRMS (ESI) *m*/*z*: calcd for C_27_H_34_N_4_NaO_7_S_2_ [M + H]^+^, 611.1610; found, 611.1608. ^1^H NMR (500 MHz, CDCl_3_): δ 8.21 (d, *J* = 7.7 Hz, 1H), 7.97 (dd, *J* = 7.5, 1.4 Hz, 1H), 7.91–7.85 (m, 2H), 7.70–7.63 (m, 1H), 7.58–7.54 (m, 2H), 7.48 (td, *J* = 7.5, 1.4 Hz, 1H), 7.40–7.33 (m, 3H), 5.20–5.17 (m, 1H), 5.15 (dd, *J* = 11.5, 3.0 Hz, 1H), 4.87 (ddd, *J* = 7.7, 3.0, 2.0 Hz, 1H), 4.63 (dd, *J* = 11.5, 2.0 Hz, 1H), 4.38 (d, *J* = 10.5 Hz, 1H), 4.15 (dd, *J* = 8.7, 3.0 Hz, 1H), 4.08 (d, *J* = 10.5 Hz, 1H), 3.83–3.77 (m, 1H), 3.24–3.17 (m, 1H), 3.10 (dd, *J* = 14.6, 4.4 Hz, 1H), 3.08 (s, 3H), 2.96 (dd, *J* = 14.6, 7.0 Hz, 1H), 2.93 (s, 3H), 2.35–2.29 (m, 1H), 2.10–1.98 (m, 1H), 1.77–1.61 (m, 2H). ^13^C NMR (126 MHz, CDCl_3_): δ 172.0, 169.1, 168.5, 168.2, 137.3, 135.2, 133.7, 132.7, 132.0, 131.9, 129.8, 129.5, 129.5, 127.9, 127.9, 127.8, 66.4, 62.3, 54.1, 50.0, 49.5, 38.0, 37.1, 35.9, 35.4, 29.7, 24.8.

#### (4*S*,7*R*)-7-((*S*)-1-(Benzylsulfonyl)pyrrolidine-2-carboxamido)-*N*,*N*-dimethyl-6,10-dioxo-1,3,4,5,6,7,8,10-octahydrobenzo[*j*][1]oxa[8]thia[5]azacyclododecine-4-carboxamide (**37**)

Compound **108** (28 mg, 51 μmol, 1.0 equiv) was dissolved in 4 M HCl in 1,4-dioxane (2 mL) and stirred for 1 h at rt. After evaporation of the volatiles under reduced pressure, the resulting salt was dissolved in DCM (2 mL). Phenylmethanesulfonyl chloride (19 mg, 0.10 mmol, 2.0 equiv) and triethylamine (28 μL, 0.20 mmol, 4.0 equiv) were added, and the reaction mixture was stirred for 2 h at rt. EtOAc (50 mL) was added and the mixture was washed with a 1 M aqueous HCl solution (25 mL). The organic phase was dried over MgSO_4_, filtered, and concentrated under reduced pressure. The crude product was purified using reverse-phase HPLC with a gradient 20–75% MeCN/water to give **37** (13 mg, 22 μmol, 43% yield) as a colorless powder. HRMS (ESI) *m*/*z*: calcd for C_28_H_35_N_4_O_7_S_2_ [M + H]^+^, 603.1947; found, 603.1943. ^1^H NMR (400 MHz, CDCl_3_): δ 7.90 (dd, *J* = 7.7, 1.3 Hz, 1H), 7.61 (d, *J* = 7.8 Hz, 1H), 7.47–7.41 (m, 3H), 7.40–7.29 (m, 6H), 5.18–5.11 (m, 1H), 5.09 (dd, *J* = 11.3, 3.1 Hz, 1H), 4.81 (dt, *J* = 8.0, 2.2 Hz, 1H), 4.52 (dd, *J* = 11.3, 2.0 Hz, 1H), 4.40 (d, *J* = 14.0 Hz, 1H), 4.34 (d, *J* = 14.0 Hz, 1H), 4.22 (d, *J* = 10.2 Hz, 1H), 4.01 (d, *J* = 10.2 Hz, 1H), 3.90 (dd, *J* = 7.7, 3.7 Hz, 1H), 3.47–3.40 (m, 1H), 3.24–3.11 (m, 1H), 3.10–3.04 (m, 1H), 3.06 (s, 3H), 2.99 (dd, *J* = 14.6, 7.5 Hz, 1H), 2.90 (s, 3H), 2.27–2.19 (m, 1H), 2.08–1.96 (m, 1H), 1.96–1.78 (m, 2H). ^13^C NMR (101 MHz, CDCl_3_): δ 171.7, 169.1, 168.3, 168.3, 137.2, 132.5, 132.0, 131.7, 130.9, 130.9, 129.9, 129.0, 128.8, 128.8, 128.3, 127.6, 66.4, 62.6, 56.8, 53.8, 49.6, 49.5, 37.6, 37.0, 35.8, 35.3, 29.9, 25.1.

#### 4-((*S*)-2-(((4*S*,7*R*)-4-(Dimethylcarbamoyl)-6,10-dioxo-1,3,4,5,6,7,8,10-octahydrobenzo[*j*][1]oxa[8]thia[5]azacyclododecin-7-yl)carbamoyl)pyrrolidin-1-yl)-4-oxobutanoic Acid (**38**)

Compound **108** (25 mg, 46 μmol, 1.0 equiv) was dissolved in 4 M HCl in 1,4-dioxane (1 mL), and the mixture was stirred for 1 h at rt. After evaporation of the volatiles under reduced pressure, the resulting salt was dissolved in DMSO (1 mL). Succinic anhydride (14 mg, 0.14 mmol, 3.0 equiv) and DIPEA (31 μL, 0.18 mmol, 4.0 equiv) were added, and the reaction mixture was stirred for 2 h at 50 **°**C. EtOAc (50 mL) was added, and the organic phase was washed a 1 M aqueous HCl solution (25 mL). The organic phase was dried over MgSO_4_, filtered, and concentrated under reduced pressure. The crude product was purified using reverse-phase HPLC with a gradient 15–75% MeCN/water (containing 0.1% TFA) in water to give **38** (8 mg, 15 μmol, 32% over two steps) as a colorless powder. HRMS (ESI) *m*/*z*: calcd for C_25_H_33_N_4_O_8_S [M + H]^+^, 549.2019; found, 549.1998. ^1^H NMR (500 MHz, CDCl_3_): δ 7.95 (dd, *J* = 7.8, 1.6 Hz, 1H), 7.81 (d, *J* = 9.1 Hz, 1H), 7.70 (d, *J* = 9.7 Hz, 1H), 7.50 (td, *J* = 7.6, 1.5 Hz, 1H), 7.42 (dd, *J* = 7.6, 1.5 Hz, 1H), 7.36 (td, *J* = 7.8, 1.6 Hz, 1H), 5.29 (ddd, *J* = 10.0, 9.7, 4.8 Hz, 1H), 5.16 (dd, *J* = 10.9, 2.0 Hz, 1H), 5.00 (dt, *J* = 9.1, 2.0 Hz, 1H), 4.47–4.40 (m, 2H), 4.27 (d, *J* = 9.5 Hz, 1H), 3.91 (d, *J* = 9.5 Hz, 1H), 3.71–3.64 (m, 1H), 3.55–3.48 (m, 1H), 3.19 (s, 3H), 3.13 (dd, *J* = 14.6, 10.0 Hz, 1H), 3.08 (dd, *J* = 14.6, 4.8 Hz, 1H), 3.01 (s, 3H), 2.69–2.56 (m, 4H), 2.40–2.33 (m, 1H), 2.23–2.17 (m, 1H), 2.06–1.95 (m, 2H). *The COOHsignal was not detectable in this spectrum.*^13^C NMR (126 MHz, CDCl_3_): δ 175.4, 172.6, 172.0, 170.9, 169.7, 167.7, 136.7, 132.8, 132.6, 131.9, 129.7, 127.8, 67.1, 60.6, 53.2, 50.2, 47.7, 38.1, 37.6, 36.5, 35.5, 29.9, 29.7, 27.9, 25.1.

#### (4*S*,7*R*)-*N*,*N*-Dimethyl-7-((*S*)-1-(4-(methylamino)-4-oxobutanoyl)pyrrolidine-2-carboxamido)-6,10-dioxo-1,3,4,5,6,7,8,10-octahydrobenzo[*j*][1]oxa[8]thia[5]azacyclododecine-4-carboxamide (**39**)

Compound **108** (30 mg, 55 μmol, 1.0 equiv) was dissolved in 4 M HCl in 1,4-dioxane (3 mL) and stirred for 1 h at rt. After evaporation of the volatiles under reduced pressure, the resulting salt was dissolved in DMSO (1 mL). 4-(Methylamino)-4-oxobutanoic acid (14 mg, 0.11 mmol, 2.0 equiv), HATU (42 mg, 0.11 mmol, 2.0 equiv), and DIPEA (38 μL, 0.22 mmol, 4.0 equiv) were added, and the reaction mixture was stirred for 1 h at rt. EtOAc (50 mL) was added, and the mixture was washed with a 1 M aqueous HCl solution (25 mL), saturated aqueous NaHCO_3_ solution (25 mL), and brine (25 mL). The organic phase was dried over MgSO_4_, filtered, and concentrated under reduced pressure. The crude product was purified using reverse-phase HPLC with a gradient 25–75% MeCN/water to give **39** (8 mg, 14 μmol, 25% over two steps) as a colorless powder. HRMS (ESI) *m*/*z*: calcd for C_26_H_36_N_5_O_7_S [M + H]^+^, 562.2335; found, 562.2349. ^1^H NMR (500 MHz, CDCl_3_): δ 8.22 (d, *J* = 9.7 Hz, 1H), 7.86 (dd, *J* = 7.6, 1.4 Hz, 1H), 7.55–7.50 (m, 1H), 7.47 (td, *J* = 7.5, 1.5 Hz, 1H), 7.40 (dd, *J* = 7.5, 1.5 Hz, 1H), 7.35 (td, *J* = 7.6, 1.4 Hz, 1H), 7.15 (d, *J* = 9.3 Hz, 1H), 5.25 (dd, *J* = 10.8, 2.5 Hz, 1H), 5.12 (ddd, *J* = 10.5, 9.7, 4.8 Hz, 1H), 4.97 (ddd, *J* = 9.3, 2.5, 2.1 Hz, 1H), 4.34 (dd, *J* = 10.8, 2.1 Hz, 1H), 4.11 (dd, *J* = 7.6, 6.1 Hz, 1H), 3.94 (d, *J* = 9.5 Hz, 1H), 3.90 (d, *J* = 9.5 Hz, 1H), 3.62–3.50 (m, 2H), 3.19 (dd, *J* = 14.6, 10.5 Hz, 1H), 3.14 (s, 3H), 3.14–3.08 (m, 1H), 2.95 (s, 3H), 2.95–2.85 (m, 1H), 2.80 (d, *J* = 4.8 Hz, 3H), 2.49–2.35 (m, 2H), 2.28–2.02 (m, 4H), 1.95–1.87 (m, 1H). ^13^C NMR (126 MHz, CDCl_3_): δ 173.6, 172.4, 171.9, 169.9, 169.9, 167.7, 136.4, 132.6, 132.5, 131.5, 130.3, 127.8, 66.6, 61.2, 52.7, 50.0, 47.4, 37.2, 37.1, 36.3, 35.7, 30.2, 30.1, 28.8, 26.2, 25.4.

#### (4*S*,7*R*)-7-((*S*)-1-((*E*)-4-(Methylamino)-4-oxobut-2-enoyl)pyrrolidine-2-carboxamido)-*N*,*N*-dimethyl-6,10-dioxo-1,3,4,5,6,7,8,10-octahydrobenzo[*j*][1]oxa[8]thia[5]azacyclododecine-4-carboxamide (**40**)

Compound **40** was synthesized following the procedure described for the synthesis of compound **39** using compound **108** (30 mg, 55 μmol, 1.0 equiv), (*E*)-4-(methylamino)-4-oxobut-2-enoic acid, (15 mg, 0.11 mmol, 2.0 equiv), EDC·HCl (21 mg, 0.11 mmol, 2.0 equiv), and DIPEA (38 μL, 0.22 mmol, 4.0 equiv). The crude product was purified using reverse-phase HPLC with a gradient 25–75% MeCN/water to give **40** (7 mg, 13 μmol, 24% over two steps) as a colorless powder. HRMS (ESI) *m*/*z*: calcd for C_26_H_34_N_5_O_7_S [M + H]^+^, 560.2179; found, 560.2191. ^1^H NMR (500 MHz, CDCl_3_): δ 8.31 (d, *J* = 9.8 Hz, 1H), 7.95 (dd, *J* = 7.6, 1.4 Hz, 1H), 7.50 (td, *J* = 7.5, 1.5 Hz, 1H), 7.43 (dd, *J* = 7.5, 1.5 Hz, 1H), 7.37 (td, *J* = 7.6, 1.4 Hz, 1H), 7.30–7.27 (m, 1H), 7.27–7.24 (m, 1H), 7.12 (q, *J* = 4.9 Hz, 1H), 7.05 (d, *J* = 14.8 Hz, 1H), 5.25 (dd, *J* = 11.0, 2.5 Hz, 1H), 5.17 (ddd, *J* = 10.4, 9.8, 4.6 Hz, 1H), 4.92 (ddd, *J* = 8.9, 2.5, 2.1 Hz, 1H), 4.46 (dd, *J* = 11.0, 2.1 Hz, 1H), 4.40 (dd, *J* = 7.7, 5.7 Hz, 1H), 4.29 (d, *J* = 9.4 Hz, 1H), 3.92 (d, *J* = 9.4 Hz, 1H), 3.78–3.72 (m, 2H), 3.32 (dd, *J* = 15.1, 10.4 Hz, 1H), 3.15 (dd, *J* = 15.1, 4.6 Hz, 1H), 3.09 (s, 3H), 2.96 (d, *J* = 4.9 Hz, 3H), 2.84 (s, 3H), 2.39–2.19 (m, 2H), 2.19–2.09 (m, 1H), 2.02–1.97 (m, 1H). ^13^C NMR (126 MHz, CDCl_3_): δ 171.9, 169.8, 169.2, 167.6, 165.2, 165.1, 138.3, 136.8, 132.8, 132.6, 131.9, 129.8, 127.8, 127.3, 66.7, 61.5, 53.4, 50.6, 47.6, 38.4, 37.4, 36.3, 35.9, 28.3, 26.4, 25.5.

#### (4*S*,7*R*)-7-((*S*)-1-(3-Cyanothiophene-2-carbonyl)pyrrolidine-2-carboxamido)-*N*,*N*-dimethyl-6,10-dioxo-1,3,4,5,6,7,8,10-octahydrobenzo[*j*][1]oxa[8]thia[5]azacyclododecine-4-carboxamide (**41**)

Compound **41** was synthesized following the procedure described for the synthesis of compound **39** using compound **108** (25 mg, 46 μmol, 1.0 equiv), 3-cyanothiophene-2-carboxylic acid (14 mg, 92 μmol, 2.0 equiv), HATU (35 mg, 92 μmol, 2.0 equiv), and DIPEA (31 μL, 0.18 mmol, 4.0 equiv). The crude product was purified using reverse-phase HPLC with a gradient 15–75% MeCN/water to give **41** (12 mg, 20 μmol, 45% over two steps) as a colorless powder. HRMS (ESI) *m*/*z*: calcd for C_27_H_30_N_5_O_6_S_2_ [M + H]^+^, 584.1632; found, 584.1623. ^1^H NMR (600 MHz, CDCl_3_): δ 7.91 (dd, *J* = 7.8, 1.5 Hz, 1H), 7.76 (d, *J* = 9.3 Hz, 1H), 7.55 (d, *J* = 5.1 Hz, 1H), 7.54–7.51 (m, 1H), 7.45 (td, *J* = 7.5, 1.5 Hz, 1H), 7.38 (dd, *J* = 7.5, 1.5 Hz, 1H), 7.34 (d, *J* = 5.1 Hz, 1H), 7.33–7.30 (m, 1H) 5.18 (dd, *J* = 11.3, 2.5 Hz, 1H), 5.09 (ddd, *J* = 9.8, 9.3, 4.5 Hz, 1H), 4.84 (ddd, *J* = 8.6, 2.5, 2.0 Hz, 1H), 4.64 (dd, *J* = 7.7, 5.7 Hz, 1H), 4.50 (dd, *J* = 11.3, 2.0 Hz, 1H), 4.42 (d, *J* = 9.9 Hz, 1H), 4.00 (d, *J* = 9.9 Hz, 1H), 3.94–3.89 (m, 1H), 3.87–3.82 (m, 1H), 3.12 (dd, *J* = 14.8, 9.8 Hz, 1H), 3.07–3.01 (m, 1H), 3.01 (s, 3H), 2.73 (s, 3H), 2.42–2.35 (m, 1H), 2.32–2.26 (m, 1H), 2.22–2.16 (m, 1H), 2.04–1.98 (m, 1H). ^13^C NMR (151 MHz, CDCl_3_): δ 171.0, 169.0, 168.4, 168.0, 161.3, 143.4, 137.2, 132.6, 132.4, 131.9, 129.9, 129.7, 129.5, 127.6, 114.6, 113.7, 66.5, 62.1, 53.8, 50.1, 49.9, 38.2, 37.0, 35.7, 35.5, 28.2, 25.8.

#### (4*S*,7*R*)-7-((*S*)-1-(Isoxazole-3-carbonyl)pyrrolidine-2-carboxamido)-*N*,*N*-dimethyl-6,10-dioxo-1,3,4,5,6,7,8,10-octahydrobenzo[*j*][1]oxa[8]thia[5]azacyclododecine-4-carboxamide (**42**)

Compound **42** was synthesized following the procedure described for the synthesis of compound **39** using compound **108** (25 mg, 46 μmol, 1.0 equiv), isoxazole-3-carboxylic acid (11 mg, 92 μmol, 2.0 equiv), HATU (35 mg, 92 μmol, 2.0 equiv), and DIPEA (31 μL, 0.18 mmol, 4.0 equiv). The crude product was purified using reverse-phase HPLC with a gradient 25–75% MeCN/water to give **42** (4 mg, 7 μmol, 15% over two steps) as a colorless powder. HRMS (ESI) *m*/*z*: calcd for C_25_H_30_N_5_O_7_S [M + H]^+^, 544.1860; found, 544.1845. ^1^H NMR (500 MHz, CDCl_3_): δ 8.48 (d, *J* = 1.7 Hz, 1H), 8.25 (d, *J* = 9.7 Hz, 1H), 7.92 (dd, *J* = 7.8, 1.5 Hz, 1H), 7.51 (td, *J* = 7.7, 1.4 Hz, 1H), 7.43 (dd, *J* = 7.7, 1.4 Hz, 1H), 7.38 (td, *J* = 7.8, 1.5 Hz, 1H), 7.24 (d, *J* = 8.9 Hz, 1H), 7.20 (d, *J* = 1.7 Hz, 1H), 5.28 (dd, *J* = 11.0, 2.5 Hz, 1H), 5.16 (ddd, *J* = 10.7, 9.7, 4.7 Hz, 1H), 4.97 (ddd, *J* = 8.9, 2.5, 2.1 Hz, 1H), 4.46 (dd, *J* = 11.0, 2.1 Hz, 1H), 4.43 (dd, *J* = 7.6, 5.5 Hz, 1H), 4.26–4.19 (m, 1H), 4.13 (d, *J* = 9.5 Hz, 1H), 4.10–4.02 (m, 1H), 3.97 (d, *J* = 9.5 Hz, 1H), 3.34 (dd, *J* = 14.9, 10.7 Hz, 1H), 3.16 (dd, *J* = 14.9, 4.7 Hz, 1H), 3.09 (s, 3H), 2.85 (s, 3H), 2.39–2.31 (m, 1H), 2.25–2.20 (m, 2H), 2.08–2.01 (m, 1H). ^13^C NMR (126 MHz, CDCl_3_): δ 172.0, 169.2, 169.1, 167.9, 159.6, 158.9, 158.8, 158.4, 136.7, 132.8, 132.7, 131.8, 129.8, 127.8, 107.4, 66.7, 62.4, 53.2, 49.9, 37.6, 37.1, 36.1, 35.8, 28.5, 25.8.

#### (*S*)-*N*^1^-Benzoyl-*N*^2^-((4*S*,7*R*)-4-(dimethylcarbamoyl)-6,10-dioxo-1,3,4,5,6,7,8,10-octahydrobenzo[*j*][1]oxa[8]thia[5]azacyclododecin-7-yl)pyrrolidine-1,2-dicarboxamide (**43**)

Compound **108** (19 mg, 35 μmol, 1.0 equiv) was dissolved in 4 M HCl in 1,4-dioxane (2 mL), and the mixture was stirred for 1 h at rt. After evaporation of the volatiles under reduced pressure, the resulting salt was dissolved in MeCN (1 mL). Benzoyl isocyanate (12 μL, 86 μmol, 2.5 equiv) and DIPEA (25 μL, 0.14 mmol, 4.0 equiv) were added, and the mixture was stirred for 4 h at rt. EtOAc (50 mL) was added, and the organic phase was washed with a 1 M aqueous HCl solution (25 mL) and brine (25 mL). The organic phase was dried over MgSO_4_, filtered, and concentrated under reduced pressure. The crude product was purified using reverse-phase HPLC with a gradient 25–75% MeCN/water to give to give **43** (4 mg, 7 μmol, 20% over two steps) as a colorless powder. HRMS (ESI) *m*/*z*: calcd for C_29_H_34_N_5_O_7_S [M + H]^+^, 596.2173; found, 596.2159. ^1^H NMR (500 MHz, CDCl_3_): δ 9.24 (s, 1H), 8.07 (d, *J* = 9.5 Hz, 1H), 8.04–7.98 (m, 3H), 7.59 (td, *J* = 7.2, 1.3 Hz, 1H), 7.53 (d, *J* = 9.1 Hz, 1H), 7.50–7 .45 (m, 3H), 7.41–7.33 (m, 2H), 5.27 (ddd, *J* = 10.0, 9.5, 4.4 Hz, 1H), 5.20 (dd, *J* = 11.2, 2.5 Hz, 1H), 4.91 (ddd, *J* = 9.1, 2.5, 2.3 Hz, 1H), 4.75 (d, *J* = 9.8 Hz, 1H), 4.61 (dd, *J* = 8.1, 2.1 Hz, 1H), 4.47 (dd, *J* = 11.2, 2.3 Hz, 1H), 3.89 (d, *J* = 9.8 Hz, 1H), 3.88–3.81 (m, 1H), 3.66–3.59 (m, 1H), 3.17 (s, 3H), 3.07 (dd, *J* = 15.2, 10.0 Hz, 1H), 3.02 (s, 3H), 2.98 (dd, *J* = 15.0, 4.4 Hz, 1H), 2.51–2.39 (m, 1H), 2.37–2.31 (m, 1H), 2.18–2.00 (m, 2H). ^13^C NMR (126 MHz, CDCl_3_): δ 171.3, 170.5, 168.4, 167.0, 165.0, 155.0, 137.2, 132.9, 132.8, 132.6, 132.5, 132.5, 129.4, 128.7, 128.7, 128.0, 128.0, 127.8, 66.7, 61.1, 53.6, 50.4, 48.0, 39.2, 37.5, 36.0, 35.7, 28.0, 25.0.

#### (4*S*,7*R*)-7-((*S*)-1-((2-Methoxybenzoyl)glycyl)pyrrolidine-2-carboxamido)-*N*,*N*-dimethyl-6,10-dioxo-1,3,4,5,6,7,8,10-octahydrobenzo[*j*][1]oxa[8]thia[5]azacyclododecine-4-carboxamide (**44**)

Compound **44** was synthesized following the procedure described for the synthesis of compound **39** using compound **108** (25 mg, 46 μmol, 1.0 equiv), 2-[(2-methoxyphenyl)formamido]acetic acid (20 mg, 92 μmol, 2.0 equiv), HATU (35 mg, 92 μmol, 2.0 equiv), and DIPEA (31 μL, 0.18 mmol, 4.0 equiv). The crude product was purified using reverse-phase HPLC with a gradient 15–75% MeCN/water to give **44** (11 mg, 17 μmol, 37% over two steps) as a colorless powder. HRMS (ESI) *m*/*z*: calcd for C_31_H_38_N_5_O_8_S [M + H]^+^, 640.2426; found, 640.2419. ^1^H NMR (500 MHz, CDCl_3_): δ 8.81 (t, *J* = 4.5 Hz, 1H), 8.20 (dd, *J* = 7.8, 1.8 Hz, 1H), 8.08 (d, *J* = 8.9 Hz, 1H), 8.01–7.95 (m, 1H), 7.53–7.48 (m, 1H), 7.46 (d, *J* = 9.2 Hz, 1H), 7.34–7.29 (m, 2H), 7.13 (td, *J* = 7.5, 1.0 Hz, 1H), 7.01–6.98 (m, 1H), 6.94 (dd, *J* = 8.4, 1.0 Hz, 1H), 5.16–5.10 (m, 2H), 4.84 (dt, *J* = 8.9, 2.3 Hz, 1H), 4.65 (dd, *J* = 8.1, 2.7 Hz, 1H), 4.56 (d, *J* = 9.8 Hz, 1H), 4.53–4.48 (m, 2H), 4.33 (dd, *J* = 17.7, 4.5 Hz, 1H), 3.90–3.84 (m, 1H), 3.73 (s, 3H), 3.61–3.56 (m, 1H), 3.54 (d, *J* = 9.8 Hz, 1H), 3.06 (s, 3H), 2.94–2.89 (m, 1H), 2.90 (s, 3H), 2.74 (dd, *J* = 14.9, 10.0 Hz, 1H), 2.50–2.44 (m, 1H), 2.33–2.25 (m, 1H), 2.12–2.06 (m, 1H), 1.99–1.92 (m, 1H). ^13^C NMR (126 MHz, CDCl_3_): δ 171.0, 170.6, 169.9, 168.5, 167.7, 165.9, 157.9, 137.4, 133.0, 132.8, 132.5, 132.4, 132.2, 129.5, 127.7, 121.7, 121.2, 111.5, 66.9, 60.5, 55.8, 53.5, 50.0, 46.8, 43.1, 39.2, 37.2, 35.9, 35.9, 27.0, 25.5.

#### (4*S*,7*R*)-*N*,*N*-Dimethyl-6,10-dioxo-7-((*S*)-1-(thieno[2,3-b]pyridine-2-carbonyl)pyrrolidine-2-carboxamido)-1,3,4,5,6,7,8,10-octahydrobenzo[*j*][1]oxa[8]thia[5]azacyclododecine-4-carboxamide (**45**)

Compound **45** was synthesized following the procedure described for the synthesis of compound **39** using compound **108** (25 mg, 46 μmol, 1.0 equiv), thieno[2,3-*b*]pyridine-2-carboxylic acid (16 mg, 92 μmol, 2.0 equiv), HATU (35 mg, 92 μmol, 2.0 equiv), and DIPEA (31 μL, 0.18 mmol, 4.0 equiv). The crude product was purified using reverse-phase HPLC with a gradient 15–75% MeCN/water to give **45** (8 mg, 13 μmol, 29% over two steps) as a colorless powder. HRMS (ESI) *m*/*z*: calcd for C_29_H_32_N_5_O_6_S_2_ [M + H]^+^, 610.1789; found, 610.1773. ^1^H NMR (500 MHz, CDCl_3_): δ 8.67–8.64 (m, 1H), 8.14 (d, *J* = 8.0 Hz, 1H), 7.99 (s, 1H), 7.88 (d, *J* = 7.7 Hz, 1H), 7.79–7.76 (m, 2H), 7.42–7.34 (m, 2H), 7.31–7.26 (m, 2H), 5.19 (dd, *J* = 11.0, 2.5 Hz, 1H), 5.12 (td, *J* = 9.2, 4.9 Hz, 1H), 4.90–4.86 (m, 1H), 4.70 (dd, *J* = 7.9, 4.3 Hz, 1H), 4.47 (dd, *J* = 11.0, 2.3 Hz, 1H), 4.22 (d, *J* = 10.0 Hz, 1H), 4.19–4.15 (m, 1H), 4.02–3.94 (m, 1H), 3.83 (d, *J* = 10.0 Hz, 1H), 3.07–2.96 (m, 2H), 3.02 (s, 3H), 2.79 (s, 3H), 2.43–2.29 (m, 2H), 2.16–2.01 (m, 2H). ^13^C NMR (126 MHz, CDCl_3_): δ 171.5, 169.1, 168.7, 167.8, 163.6, 162.5, 148.8, 137.1, 136.4, 132.9, 132.5, 132.2, 132.1, 131.8, 129.8, 127.7, 127.5, 120.2, 66.4, 62.2, 53.5, 49.8, 49.7, 38.1, 37.0, 36.0, 35.8, 27.4, 26.0.

#### (4*S*,7*R*)-*N*,*N*-Dimethyl-6,10-dioxo-7-((*S*)-1-(2-(3-oxobenzo[*d*]isothiazol-2(3*H*)-yl)acetyl)pyrrolidine-2-carboxamido)-1,3,4,5,6,7,8,10-octahydrobenzo[*j*][1]oxa[8]thia[5]azacyclododecine-4-carboxamide (**46**)

Compound **46** was synthesized following the procedure described for the synthesis of compound **39** using compound **108** (20 mg, 36 μmol, 1.0 equiv), 2-(3-oxo-2,3-dihydro-1,2-benzothiazol-2-yl)acetic acid (15 mg, 72 μmol, 2.0 equiv), HATU (27 mg, 72 μmol, 2.0 equiv), and DIPEA (25 μL, 0.14 mmol, 4.0 equiv). The crude product was purified using reverse-phase HPLC with a gradient 25–75% MeCN/water to give **46** (8 mg, 12 μmol, 33% over two steps) as a colorless powder. HRMS (ESI) *m*/*z*: calcd for C_30_H_34_N_5_O_7_S_2_ [M + H]^+^, 640.1900; found, 640.1882. ^1^H NMR (500 MHz, CDCl_3_): δ 8.12 (d, *J* = 8.7 Hz, 1H), 8.03 (d, *J* = 7.9 Hz, 1H), 7.93 (dd, *J* = 7.9, 1.5 Hz, 1H), 7.63 (t, *J* = 8.1 Hz, 1H), 7.51 (d, *J* = 8.1 Hz, 1H), 7.45 (d, *J* = 8.7 Hz, 1H), 7.44 (t, *J* = 8.7 Hz, 1H), 7.37 (td, *J* = 7.6, 1.3 Hz, 1H), 7.29 (td, *J* = 7.9, 1.5 Hz, 1H), 7.16 (dd, *J* = 7.6, 1.3 Hz, 1H), 5.19–5.14 (m, 1H), 5.12 (dd, *J* = 11.1, 2.5 Hz, 1H), 4.97 (d, *J* = 16.5 Hz, 1H), 4.82 (ddd, *J* = 8.7, 2.5, 2.2 Hz, 1H), 4.72 (d, *J* = 16.5 Hz, 1H), 4.62 (dd, *J* = 8.0, 2.5 Hz, 1H), 4.44 (dd, *J* = 11.2, 2.2 Hz, 1H), 4.40 (d, *J* = 10.1 Hz, 1H), 3.94–3.86 (m, 1H), 3.79 (d, *J* = 10.1 Hz, 1H), 3.78–3.72 (m, 1H), 3.14–3.09 (m, 2H), 3.08 (s, 3H), 2.95 (s, 3H), 2.51–2.43 (m, 1H), 2.32–2.25 (m, 1H), 2.11–2.06 (m, 1H), 2.00–1.96 (m, 1H). ^13^C NMR (126 MHz, CDCl_3_): δ 170.6, 169.6, 168.5, 168.4, 167.8, 165.8, 141.7, 137.4, 132.4, 132.2, 132.0, 132.0, 129.8, 127.6, 126.7, 125.4, 123.4, 120.2, 66.5, 60.4, 53.5, 49.6, 47.2, 45.3, 38.7, 37.1, 35.9, 35.7, 26.9, 25.4.

#### (4*S*,7*R*)-7-((*S*)-1-(2-(1,3-Dioxoisoindolin-2-yl)acetyl)pyrrolidine-2-carboxamido)-*N*,*N*-dimethyl-6,10-dioxo-1,3,4,5,6,7,8,10-octahydrobenzo[*j*][1]oxa[8]thia[5]azacyclododecine-4-carboxamide (**47**)

Compound **47** was synthesized following the procedure described for the synthesis of compound **39** using compound **108** (25 mg, 46 μmol, 1.0 equiv), 2-(1,3-dioxo-2,3-dihydro-1*H*-isoindol-2-yl)acetic acid (19 mg, 92 μmol, 2.0 equiv), HATU (35 mg, 92 μmol, 2.0 equiv), and DIPEA (31 μL, 0.18 mmol, 4.0 equiv). The crude product was purified using reverse-phase HPLC with a gradient 15–75% MeCN/water to give **47** (6 mg, 9 μmol, 20% over two steps) as a colorless powder. HRMS (ESI) *m*/*z*: calcd for C_31_H_34_N_5_O_8_S [M + H]^+^, 636.2123; found, 636.2108. ^1^H NMR (600 MHz, CDCl_3_): δ 8.14 (d, *J* = 8.6 Hz, 1H), 7.93 (dd, *J* = 7.8, 1.5 Hz, 1H), 7.88–7.85 (m, 2H), 7.76–7.72 (m, 2H), 7.33 (td, *J* = 7.5, 1.5 Hz, 1H), 7.26–7.22 (m, 2H), 7.13 (dd, *J* = 7.5, 1.5 Hz, 1H), 5.14–5.07 (m, 2H), 4.98 (d, *J* = 16.4 Hz, 1H), 4.83 (ddd, *J* = 8.6, 2.9, 2.0 Hz, 1H), 4.63 (dd, *J* = 8.1, 2.0 Hz, 1H), 4.43 (d, *J* = 9.9 Hz, 1H), 4.37 (d, *J* = 16.4 Hz, 1H), 4.36 (dd, *J* = 114, 2.0 Hz, 1H), 3.97–3.93 (m, 1H), 3.71–3.68 (m, 1H), 3.68 (d, *J* = 9.9 Hz, 1H), 3.07–3.02 (m, 1H), 3.06 (s, 3H), 2.99–2.95 (m, 1H), 2.94 (s, 3H), 2.51–2.45 (m, 1H), 2.34–2.29 (m, 1H), 2.15–2.10 (m, 1H), 1.99–1.95 (m, 1H). ^13^C NMR (151 MHz, CDCl_3_): δ 170.6, 169.7, 168.5, 168.0, 167.9, 167.8, 137.5, 134.3, 134.3, 132.6, 132.6, 132.4, 132.3, 132.3, 129.8, 127.6, 123.6, 66.9, 60.7, 53.5, 50.0, 47.1, 39.9, 38.9, 37.1, 35.9, 35.7, 27.1, 25.5.

#### (4*S*,7*R*)-*N*,*N*-Dimethyl-7-((2*S*)-1-(2-(4-methyl-2,5-dioxo-4-phenylimidazolidin-1-yl)acetyl)pyrrolidine-2-carboxamido)-6,10-dioxo-1,3,4,5,6,7,8,10-octahydrobenzo[*j*][1]oxa[8]thia[5] Azacyclododecine-4-carboxamide (**48**)

Compound **48** was synthesized following the procedure described for the synthesis of compound **39** using compound **108** (30 mg, 54 μmol, 1.0 equiv), 2-(1,3-dioxo-2,3-dihydro-1*H*-isoindol-2-yl)acetic acid (27 mg, 0.11 mmol, 2.0 equiv), HATU (42 mg, 0.11 mmol, 2.0 equiv), and DIPEA (38 μL, 0.22 mmol, 4.0 equiv). The crude product was purified using reverse-phase HPLC with a gradient 15–75% MeCN/water to give **48** (14 mg, 20 μmol, 37% over two steps) as a colorless powder. The product was obtained as an inseparable mixture of diastereoisomers in a ratio of 1:1. HRMS (ESI) *m*/*z*: calcd for C_33_H_39_N_6_O_8_S [M + H]^+^, 679.2550; found, 679.2540. ^1^H NMR (500 MHz, CDCl_3_, mixture of two diastereoisomers in a ratio 1:1): δ 8.14 (d, *J* = 8.7 Hz, 1H), 8.02–7.96 (m, 2H), 7.94 (dd, *J* = 7.9, 1.5 Hz, 1H), 7.53–7.49 (m, 2H), 7.49–7.45 (m, 2H), 7.44–7.39 (m, 2H), 7.36–7.27 (m, 8H), 7.26–7.18 (m, 4H), 6.48 (s, 1H), 6.26 (s, 1H), 5.19–5.11 (m, 2H), 5.08 (dd, *J* = 11.3, 2.9 Hz, 1H), 5.06 (dd, *J* = 11.3, 2.9 Hz, 1H), 4.94 (dt, *J* = 8.7, 2.9 Hz, 1H), 4.90 (dt, *J* = 8.7, 2.9 Hz, 1H), 4.67–4.64 (m, 1H), 4.66 (d, *J* = 16.3 Hz, 1H), 4.64–4.60 (m, 1H), 4.53 (d, *J* = 16.1 Hz, 1H), 4.47–4.43 (m, 2H), 4.43–4.39 (m, 1H), 4.26 (d, *J* = 9.8 Hz, 1H), 4.21 (d, *J* = 16.3 Hz, 1H), 4.19 (d, *J* = 16.1 Hz, 1H), 3.84 (d, *J* = 9.8 Hz, 1H), 3.82–3.78 (m, 1H), 3.75 (d, *J* = 9.8 Hz, 1H), 3.73–3.69 (m, 1H), 3.60–3.48 (m, 2H), 3.07 (s, 3H), 3.05 (s, 3H), 3.04–2.98 (m, 3H), 2.97 (s, 3H), 2.95–2.92 (m, 1H), 2.92 (s, 3H), 2.49–2.39 (m, 2H), 2.29–2.17 (m, 2H), 2.12–2.01 (m, 2H), 1.95–1.85 (m, 2H), 1.83 (s, 3H), 1.80 (s, 3H). ^13^C NMR (126 MHz, CDCl3): δ 175.1, 174.9, 170.6, 170.6, 169.9, 169.8, 168.8, 168.6, 167.9, 167.8, 167.3, 167.1, 156.2, 156.0, 138.4, 138.2, 137.4, 137.2, 132.6, 132.5, 132.5, 132.4, 132.3, 132.0, 129.8, 129.7, 128.9, 128.9, 128.8, 128.8, 128.6, 128.5, 127.6, 127.5, 125.5, 125.5, 125.4, 125.4, 66.9, 66.9, 64.3, 64.2, 60.3, 60.2, 53.3, 53.3, 50.2, 50.0, 47.0, 47.0, 40.5, 40.4, 38.7, 38.2, 37.1, 37.1, 36.1, 35.9, 35.7, 35.7, 27.0, 26.8, 25.4, 25.2, 25.2, 25.0.

#### (4*S*,7*R*)-*N*,*N*-Dimethyl-6,10-dioxo-7-((*S*)-1-(1-phenyl-1*H*-1,2,4-triazole-3-carbonyl)pyrrolidine-2-carboxamido)-1,3,4,5,6,7,8,10-octahydrobenzo[*j*][1]oxa[8]thia[5]azacyclododecine-4-carboxamide (**49**)

Compound **49** was synthesized following the procedure described for the synthesis of compound **39** using compound **108** (20 mg, 36 μmol, 1.0 equiv), 1-phenyl-1*H*-1,2,4-triazole-3-carboxylic acid (15 mg, 72 μmol, 2.0 equiv), HATU (27 mg, 72 μmol, 2.0 equiv), and DIPEA (25 μL, 0.14 mmol, 4.0 equiv). The crude product was purified using reverse-phase HPLC with a gradient 25–75% MeCN/water in water to give **49** (7 mg, 10 μmol, 31% over two steps) as a colorless powder. HRMS (ESI) *m*/*z*: calcd for C_30_H_34_N_7_O_6_S [M + H]^+^, 620.2291; found, 620.2272. ^1^H NMR (600 MHz, CDCl_3_, mixture of 2 rotamers in a ratio of 0.5:0.5): δ 9.01 (br s, 0.5H), 8.70 (s, 0.5H), 8.22 (d, *J* = 7.5 Hz, 0.5H), 8.01 (d, *J* = 8.6 Hz, 0.5H), 7.91 (d, *J* = 7.8 Hz, 0.5H), 7.81 (d, *J* = 7.8 Hz, 0.5H), 7.77 (d, *J* = 8.0 Hz, 1H), 7.69–7.63 (m, 2H), 7.54 (t, *J* = 7.8 Hz, 1H), 7.49–7.43 (m, 2H), 7.42–7.27 (m, 3 H), 5.15 (dd, *J* = 11.1, 2.7 Hz, 0.5H), 5.11 (td, *J* = 9.6, 4.4 Hz, 0.5H), 5.04–5.00 (m, 0.5H), 4.90–4.88 (m, 1H), 4.84–4.79 (m, 1.5H), 4.73–4.70 (m, 0.5H), 4.51–4.47 (m, 1H), 4.26–4.20 (m, 1H), 4.18–4.11 (m, 0.5H), 4.01 (d, *J* = 10.4 Hz, 0.5H), 3.97 (d, *J* = 10.4 Hz, 0.5H), 3.95–3.90 (m, 1H), 3.24 (dd, *J* = 14.0, 3.0 Hz, 0.5H), 3.11–3.03 (m, 1.5 H), 3.00 (s, 1.5H), 2.88 (s, 1.5H), 2.73 (s, 1.5H), 2.46 (s, 1.5H), 2.39–2.32 (m, 0.5H), 2.30–2.18 (m, 2H), 2.15–1.99 (m, 1.5H). ^13^C NMR (151 MHz, CDCl_3_ mixture of 2 rotamers): δ 173.1 and 171.3 (1C), 170.1 and 169.3 (1C), 168.7 and 168.4 (1C), 168.3 and 167.8 (1C), 159.8 and 156.6 (1C), 158.9 and 157.7 (1C), 142.4 and 141.3 (1C), 137.9 and 137.3 (1C), 136.5 and 136.4 (1C), 132.7 and 132.5 (1C), 132.4 and 132.0 (1C), 131.7 and 131.6 (1C), 129.9 (1C), 129.7 (2C), 129.0 and 128.8 (1C), 127.6 and 127.5 (1C), 120.6 (1C), 120.3 (1C), 66.7 and 66.5 (1C), 62.1 and 61.7 (1C), 55.3 and 53.5 (1C), 50.2 and 50.0 (1C), 47.8 (1C), 38.3 and 38.2 (1C), 37.1 and 36.9 (1C), 35.8 and 35.1 (1C), 35.5 (1C), 31.8 and 21.9 (1C), 27.7 and 25.6 (1C).

#### (4*S*,7*R*)-7-((*S*)-1-(2-(2-Cyanophenoxy)benzoyl)pyrrolidine-2-carboxamido)-*N*,*N*-dimethyl-6,10-dioxo-1,3,4,5,6,7,8,10-octahydrobenzo[*j*][1]oxa[8]thia[5]azacyclododecine-4-carboxamide (**50**)

Compound **50** was synthesized following the procedure described for the synthesis of compound **39** using compound **108** (20 mg, 36 μmol, 1.0 equiv), 2-(2-cyanophenoxy)benzoic acid (17 mg, 72 μmol, 2.0 equiv), HATU (27 mg, 72 μmol, 2.0 equiv), and DIPEA (25 μL, 0.14 mmol, 4.0 equiv). The crude product was purified using reverse-phase HPLC with a gradient 25–85% MeCN/water to give **50** (9 mg, 13 μmol, 36% over two steps) as a colorless powder. HRMS (ESI) *m*/*z*: calcd for C_35_H_36_N_5_O_7_S [M + H]^+^, 670.2335; found, 670.2321. ^1^H NMR (500 MHz, CDCl_3_): δ 7.94–7.88 (m, 2H), 7.61 (dd, *J* = 7.8, 1.7 Hz, 1H), 7.57–7.52 (m, 2H), 7.49 (t, *J* = 8.2 Hz, 1H), 7.47–7.41 (m, 2H), 7.31 (t, *J* = 7.8 Hz, 1H), 7.30–7.22 (m, 2H), 7.16 (t, *J* = 7.8 Hz, 1H)), 7.02 (d, *J* = 8.2 Hz, 1H), 6.95 (d, *J* = 8.2 Hz, 1H), 5.17 (dd, *J* = 11.1, 2.5 Hz, 1H), 5.11 (td, *J* = 9.5, 4.5 Hz, 1H), 4.85 (dt, *J* = 8.3, 2.5 Hz, 1H), 4.48–4.40 (m, 2H), 4.27 (d, *J* = 9.8 Hz, 1H), 3.73–3.63 (m, 2H), 3.58–3.51 (m, 1H), 3.07 (s, 3H), 3.02 (dd, *J* = 14.9, 4.5 Hz, 1H), 2.95 (dd, *J* = 14.9, 9.5 Hz, 1H), 2.86 (s, 3H), 2.32–2.25 (m, 1H), 2.25–2.18 (m, 1H), 2.16–2.09 (m, 1H), 2.03–1.95 (m, 1H). ^13^C NMR (126 MHz, CDCl_3_): δ 172.0, 169.3, 168.8, 168.0, 167.8, 159.4, 150.6, 137.1, 134.8, 133.6, 132.8, 132.2, 132.1, 131.5, 129.6, 129.4, 129.2, 127.8, 125.7, 123.3, 120.4, 117.6, 116.3, 103.1, 66.7, 60.9, 53.5, 50.1, 49.4, 38.1, 37.2, 36.0, 35.6, 28.8, 25.3.

#### (4*S*,7*R*)-7-((*S*)-1-(6-(1*H*-Imidazol-1-yl)picolinoyl)pyrrolidine-2-carboxamido)-*N*,*N*-dimethyl-6,10-dioxo-1,3,4,5,6,7,8,10-octahydrobenzo[*j*][1]oxa[8]thia[5]azacyclododecine-4-carboxamide (**51**)

Compound **51** was synthesized following the procedure described for the synthesis of compound **39** using compound **108** (20 mg, 36 μmol, 1.0 equiv), 6-(1*H*-imidazol-1-yl)pyridine-2-carboxylic acid hydrochloride (16 mg, 72 μmol, 2.0 equiv), HATU (27 mg, 72 μmol, 2.0 equiv), and DIPEA (25 μL, 0.14 mmol, 4.0 equiv). The crude product was purified using reverse-phase HPLC with a gradient 25–75% MeCN/water to give **51** (7 mg, 11 μmol, 31% over two steps) as a colorless powder. HRMS (ESI) *m*/*z*: calcd for C_30_H_34_N_7_O_6_S [M + H]^+^, 620.2286; found, 620.2273. ^1^H NMR (500 MHz, CDCl_3_): δ 8.36 (s, 1H), 8.16 (d, *J* = 7.9 Hz, 1H), 8.13 (d, *J* = 9.5 Hz, 1H), 8.01 (t, *J* = 7.9 Hz, 1H), 7.90 (dd, *J* = 7.8, 1.4 Hz, 1H), 7.64–7.61 (m, 1H), 7.50–7.44 (m, 3H), 7.38 (dd, *J* = 7.8, 1.3 Hz, 1H), 7.34 (td, *J* = 7.8, 1.4 Hz, 1H), 7.24–7.21 (m, 1H), 5.25 (dd, *J* = 11.0, 2.6 Hz, 1H), 5.17 (ddd, *J* = 9.9, 9.5, 4.7 Hz, 1H), 4.95 (ddd, *J* = 8.7, 2.6, 2.1 Hz, 1H), 4.57 (dd, *J* = 8.0, 4.4 Hz, 1H), 4.50 (dd, *J* = 11.0, 2.1 Hz, 1H), 4.28–4.16 (m, 2H), 4.13 (d, *J* = 9.7 Hz, 1H), 3.95 (d, *J* = 9.7 Hz, 1H), 3.27 (dd, *J* = 14.8, 9.9 Hz, 1H), 3.13 (dd, *J* = 14.8, 4.7 Hz, 1H), 3.09 (s, 3H), 2.81 (s, 3H), 2.40–2.25 (m, 2H), 2.24–2.12 (m, 1H), 2.09–1.99 (m, 1H). ^13^C NMR (126 MHz, CDCl_3_): δ 172.2, 169.2, 169.1, 167.9, 165.5, 151.5, 147.0, 140.3, 136.9, 135.0, 132.5, 132.4, 131.7, 131.0, 130.0, 127.7, 124.4, 116.2, 114.0, 66.6, 62.8, 53.4, 50.7, 49.8, 37.6, 37.0, 35.9, 35.9, 28.1, 26.2.

#### (4*S*,7*R*)-*N*,*N*-Dimethyl-6,10-dioxo-7-((*S*)-1-((1-phenylcyclopropane-1-carbonyl)glycyl)pyrrolidine-2-carboxamido)-1,3,4,5,6,7,8,10-octahydrobenzo[*j*][1]oxa[8]thia[5]azacyclododecine-4-carboxamide (**52**)

Compound **52** was synthesized following the procedure described for the synthesis of compound **39** using compound **108** (25 mg, 46 μmol, 1.0 equiv), 2-[(1-phenylcyclopropyl)formamido]acetic acid (21 mg, 92 μmol, 2.0 equiv), HATU (35 mg, 92 μmol, 2.0 equiv), and DIPEA (31 μL, 0.18 mmol, 4.0 equiv). The crude product was purified using reverse-phase HPLC with a gradient 25–75% MeCN/water to give **52** (6 mg, 9 μmol, 20%) as a colorless powder. HRMS (ESI) *m*/*z*: calcd for C_33_H_40_N_5_O_7_S [M + H]^+^, 650.2643; found, 650.2627. ^1^H NMR (600 MHz, CDCl_3_): δ 7.99 (dd, *J* = 7.9, 1.5 Hz, 1H), 7.53 (td, *J* = 7.6, 1.5 Hz, 1H), 7.51–7.47 (m, 2H), 7.47–7.42 (m, 3H), 7.39–7.33 (m, 3H), 7.32–7.27 (m, 1H), 6.70 (dd, *J* = 7.5, 3.5 Hz, 1H), 5.09 (dd, *J* = 11.1, 2.6 Hz, 1H), 5.05 (ddd, *J* = 10.5, 9.3, 4.2 Hz, 1H), 4.80 (ddd, *J* = 9.2, 2.6, 2.2 Hz, 1H), 4.53 (d, *J* = 9.8 Hz, 1H), 4.50–4.43 (m, 2H), 4.41 (dd, *J* = 11.1, 2.2 Hz, 1H), 3.76–3.70 (m, 2H), 3.60–3.54 (m, 1H), 3.54–3.48 (m, 1H), 3.02 (s, 3H), 2.78 (s, 3H), 2.69 (dd, *J* = 15.0, 4.2 Hz, 1H), 2.42 (dd, *J* = 15.0, 10.5 Hz, 1H), 2.34–2.25 (m, 2H), 2.05–1.97 (m, 1H), 1.96–1.87 (m, 1H), 1.64–1.61 (m, 2H), 1.16–1.11 (m, 1H), 1.06–1.01 (m, 1H). ^13^C NMR (151 MHz, CDCl_3_): δ 174.2, 171.0, 170.5, 169.7, 168.4, 167.7, 140.0, 137.3, 132.9, 132.6, 132.5, 131.1, 131.1, 129.7, 128.9, 128.9, 127.9, 127.8, 67.0, 60.4, 53.4, 50.2, 46.8, 42.7, 38.9, 37.3, 36.2, 35.9, 30.6, 27.4, 25.5, 16.3, 15.5.

#### (4*S*,7*R*)-7-((*S*)-1-(2-((3-Cyano-5-methyl-6-oxo-1,6-dihydropyridin-2-yl)thio)acetyl)pyrrolidine-2-carboxamido)-*N*,*N*-dimethyl-6,10-dioxo-1,3,4,5,6,7,8,10-octahydrobenzo[*j*][1]oxa[8]thia[5]azacyclododecine-4-carboxamide (**53**)

Compound **53** was synthesized following the procedure described for the synthesis of compound **39** using compound **108** (20 mg, 36 μmol, 1.0 equiv), 2-[(3-cyano-4-methyl-6-oxo-1,6-dihydropyridin-2-yl)sulfanyl]acetic acid (16 mg, 72 μmol, 2.0 equiv), HATU (27 mg, 72 μmol, 2.0 equiv), and DIPEA (24 μL, 0.14 mmol, 4.0 equiv). The crude product was purified using reverse-phase HPLC with a gradient 15–75% MeCN/water to give **53** (7 mg, 11 μmol, 30%) as a colorless powder. HRMS (ESI) *m*/*z*: calcd for C_30_H_35_N_6_O_7_S_2_ [M + H]^+^, 655.2009; found, 655.2001. ^1^H NMR (500 MHz, CDCl_3_): δ 7.88 (dd, *J* = 7.8, 1.4 Hz, 1H), 7.69 (d, *J* = 9.1 Hz, 1H), 7.58 (d, *J* = 8.7 Hz, 1H), 7.41 (td, *J* = 7.8, 1.4 Hz, 1H), 7.35 (dd, *J* = 7.8, 1.4 Hz, 1H), 7.30–7.26 (m, 1H), 6.33 (s, 1H), 5.17 (dd, *J* = 11.2, 2.6 Hz, 1H), 5.15–5.11 (m, 1H), 4.86 (ddd, *J* = 8.7, 2.6, 2.2 Hz, 1H), 4.47 (dd, *J* = 7.6, 3.5 Hz, 1H), 4.43 (dd, *J* = 11.2, 2.2 Hz, 1H), 4.33 (d, *J* = 15.3 Hz, 1H), 4.27 (d, *J* = 10.0 Hz, 1H), 4.00 (d, *J* = 10.0 Hz, 1H), 3.80–3.72 (m, 1H), 3.76 (d, *J* = 15.3 Hz, 1H), 3.65–3.58 (m, 1H), 3.15–3.08 (m, 2H), 3.07 (s, 3H), 2.95 (s, 3H), 2.35 (s, 3H), 2.34–2.27 (m, 2H), 2.09–2.01 (m, 2H). *The NHor the OH proton of the pyridone ring (depending on the tautomeric state) was not detectable in this spectrum.*^13^C NMR (126 MHz, CDCl_3_): δ 171.0, 169.8, 169.2, 168.7, 167.8, 163.3, 154.6, 153.0, 137.2, 132.6, 132.3, 131.8, 129.6, 127.6, 115.1, 113.4, 97.1, 66.3, 61.1, 53.4, 50.0, 48.1, 38.1, 37.3, 36.2, 35.7, 34.2, 27.9, 25.3, 20.7.

#### 2-((*S*)-2-(((4*S*,7*R*)-4-(Dimethylcarbamoyl)-6,10-dioxo-1,3,4,5,6,7,8,10-octahydrobenzo[*j*][1]oxa[8]thia[5]azacyclododecin-7-yl)carbamoyl)pyrrolidine-1-carbonyl)benzoic Acid (**54**)

Compound **108** (25 mg, 46 μmol, 1.0 equiv) was dissolved in 4 M HCl in 1,4-dioxane (3 mL) and stirred for 1 h at rt. After evaporation of the volatiles under reduced pressure, the resulting salt was dissolved in DMSO (1 mL). Phthalic acid (15 mg, 92 μmol, 2.0 equiv), HATU (35 mg, 92 μmol, 2.0 equiv), and DIPEA (30 μL, 0.18 mmol, 4.0 equiv) were added, and the reaction mixture was stirred for 2 h at rt. EtOAc (75 mL) was added, and the organic phase was washed with a 1 M aqueous HCl (25 mL) and brine (25 mL). The organic phase was dried over MgSO_4_, filtered, and concentrated under reduced pressure. The crude product was purified using reverse-phase HPLC with a gradient 15–75% MeCN/water (containing 0.1% TFA) to give **54** (10 mg, 18 μmol, 39% yield) as a colorless powder. HRMS (ESI) *m*/*z*: calcd for C_29_H_32_N_4_NaO_8_S [M + Na]^+^, 619.1839; found, 619.1830. ^1^H NMR (500 MHz, CDCl_3_): δ 12.96 (s br, 1H), 8.90 (d, *J* = 10.1 Hz, 1H), 8.83 (d, *J* = 9.2 Hz, 1H), 8.20–8.14 (m, 1H), 8.03 (dd, *J* = 7.8, 1.5 Hz, 1H), 7.68 (td, *J* = 7.5, 1.2 Hz, 1H), 7.57 (td, *J* = 7.7, 1.2 Hz, 1H), 7.39 (dd, *J* = 7.4, 1.5 Hz, 1H), 7.31 (d, *J* = 7.3 Hz, 1H), 7.26 (dd, *J* = 11.7, 7.5 Hz, 2H), 5.37 (ddd, *J* = 11.7, 10.1, 3.7 Hz, 1H), 5.22–5.16 (m, 2H), 4.98–4.92 (m, 1H), 4.70 (d, *J* = 9.6 Hz, 1H), 4.51–4.46 (m, 1H), 3.58 (d, *J* = 9.6 Hz, 1H), 3.24 (s, 3H), 3.13–3.01 (m, 3H), 3.08 (s, 3H), 2.93 (dd, *J* = 15.1, 11.7 Hz, 1H), 2.74–2.69 (m, 1H), 2.00–1.85 (m, 3H). ^13^C NMR (126 MHz, CDCl_3_): δ 171.6, 171.4, 171.2, 169.9, 168.5, 167.3, 139.6, 137.1, 134.1, 132.9, 132.8, 132.7, 130.9, 129.4, 129.0, 127.7, 126.3, 125.9, 68.1, 59.6, 53.6, 50.7, 48.8, 39.8, 38.0, 36.5, 35.9, 26.4, 24.5.

#### 2-((2-((*S*)-2-(((4*S*,7*R*)-4-(Dimethylcarbamoyl)-6,10-dioxo-1,3,4,5,6,7,8,10-octahydrobenzo[*j*][1]oxa[8]thia[5]azacyclododecin-7-yl)carbamoyl)pyrrolidin-1-yl)-2-oxoethyl)thio)benzoic Acid (**55**)

Compound **55** was synthesized following the procedure described for the synthesis of compound **54** using compound **108** (25 mg, 46 μmol, 1.0 equiv), 2-((carboxymethyl)thio)benzoic acid (19 mg, 92 μmol, 2.0 equiv), EDC·HCl (18 mg, 92 μmol, 2.0 equiv), and DIPEA (30 μL, 0.18 mmol, 4.0 equiv). The crude product was purified using reverse-phase HPLC with a gradient 15–75% MeCN/water (containing 0.1% TFA) to give **55** (6 mg, 9 μmol, 20%) as a colorless powder. HRMS (ESI) *m*/*z*: calcd for C_30_H_35_N_4_O_8_S_2_ [M + H]^+^, 643.1896; found, 643.1887. ^1^H NMR (500 MHz, CDCl_3_): δ 7.98 (d, *J* = 8.6 Hz, 1H), 7.95–7.89 (m, 2H), 7.64–7.58 (m, 2H), 7.46 (td, *J* = 7.6, 1.6 Hz, 1H), 7.40 (td, *J* = 7.8, 1.3 Hz, 1H), 7.33 (dd, *J* = 7.8, 1.3 Hz, 1H), 7.32–7.26 (m, 2H), 5.20 (td, *J* = 9.2, 4.5 Hz, 1H), 5.14 (dd, *J* = 11.2, 2.7 Hz, 1H), 4.88 (ddd, *J* = 8.6, 2.7, 2.2 Hz, 1H), 4.50 (dd, *J* = 8.2, 3.0 Hz, 1H), 4.46 (d, *J* = 10.1 Hz, 1H), 4.41 (dd, *J* = 11.2, 2.2 Hz, 1H), 3.97 (d, *J* = 14.8 Hz, 1H), 3.93 (d, *J* = 10.1 Hz, 1H), 3.83 (d, *J* = 14.8 Hz, 1H), 3.76–3.70 (m, 1H), 3.59–3.52 (m, 1H), 3.10 (s, 3H), 3.06–2.94 (m, 2H), 2.94 (s, 3H), 2.39–2.32 (m, 1H), 2.25–2.17 (m, 1H), 2.01–1.90 (m, 2H). *The COOHproton was not detectable in this spectrum.*^13^C NMR (126 MHz, CDCl_3_): δ 171.2, 170.0, 170.0, 168.9, 168.5, 167.8, 137.2, 132.6, 132.5, 132.2, 132.1, 132.1, 131.6, 131.5, 131.2, 129.6, 127.6, 126.8, 66.7, 60.8, 53.6, 50.1, 48.0, 38.5, 38.3, 37.3, 36.1, 35.4, 27.7, 25.1.

#### 1-(3-((*S*)-2-(((4*S*,7*R*)-4-(Dimethylcarbamoyl)-6,10-dioxo-1,3,4,5,6,7,8,10-octahydrobenzo[*j*][1]oxa[8]thia[5]azacyclododecin-7-yl)carbamoyl)pyrrolidine-1-carbonyl)phenyl)-6-oxo-1,4,5,6-tetrahydropyridazine-3-carboxylic Acid (**56**)

Compound **56** was synthesized following the procedure described for the synthesis of compound **54** using compound **108** (20 mg, 36 μmol, 1.0 equiv), 1-(3-carboxyphenyl)-6-oxo-1,4,5,6-tetrahydropyridazine-3-carboxylic acid (24 mg, 92 μmol, 2.0 equiv), EDC·HCl (14 mg, 72 μmol, 2.0 equiv), and DIPEA (24 μL, 0.14 mmol, 4.0 equiv). The crude product was purified using reverse-phase HPLC with a gradient 15–75% MeCN/water (containing 0.1% TFA) to give **56** (5 mg, 7 μmol, 19%) as a colorless powder. HRMS (ESI) *m*/*z*: calcd for C_33_H_37_N_6_O_9_S [M + H]^+^, 693.2343; found, 693.2345. ^1^H NMR (500 MHz, DMSO-*d*_6_): δ 13.11 (s br, 1H), 9.13 (d, *J* = 6.8 Hz, 1H), 8.06 (d, *J* = 10.0 Hz, 1H), 8.04–7.96 (m, 1H), 7.94 (dd, *J* = 8.0, 1.5 Hz, 1H), 7.87–7.83 (m, 1H), 7.70–7.67 (m, 1H), 7.59–7.54 (m, 2H), 7.52 (dd, *J* = 7.8, 1.4 Hz, 1H), 7.42 (td, *J* = 8.0, 1.5 Hz, 1H), 4.92 (dt, *J* = 10.0, 5.1 Hz, 1H), 4.87 (dd, *J* = 11.3, 2.9 Hz, 1H), 4.80 (d, *J* = 9.2 Hz, 1H), 4.52–4.47 (m, 1H), 4.45 (ddd, *J* = 6.8, 2.9, 2.1 Hz, 1H), 4.37 (dd, *J* = 11.3, 2.1 Hz, 1H), 3.97–3.88 (m, 1H), 3.80 (d, *J* = 9.2 Hz, 1H), 3.77–3.69 (m, 1H), 3.16–3.05 (m, 1H), 3.02–2.90 (m, 2H), 2.86 (s, 3H), 2.85–2.77 (m, 2H), 2.80–2.66 (m, 1H), 2.64 (s, 3H), 2.12–1.97 (m, 2H), 1.93–1.73 (m, 2H). ^13^C NMR (126 MHz, DMSO-*d*_6_): δ 172.9, 169.3, 168.7, 167.2, 167.1, 166.2, 163.4, 149.6, 141.5, 138.4, 133.4, 133.3, 132.2, 131.6, 129.7, 129.5, 129.4, 128.1, 127.8, 126.2, 66.4, 61.2, 53.7, 51.0, 50.5, 39.1, 36.9, 35.9, 35.7, 28.6, 27.4, 26.0, 22.4.

#### 3-((*S*)-2-(((4*S*,7*R*)-4-(Dimethylcarbamoyl)-6,10-dioxo-1,3,4,5,6,7,8,10-octahydrobenzo[*j*][1]oxa[8]thia[5]azacyclododecin-7-yl)carbamoyl)pyrrolidine-1-carbonyl)-5-methylthiophene-2-carboxylic Acid (**57**)

Compound **108** (25 mg, 46 μmol, 1.0 equiv) was dissolved in 4 M HCl in 1,4-dioxane (3 mL) and stirred for 1 h at rt. After evaporation of the volatiles under reduced pressure, the resulting salt was suspended in DCM (1 mL). In a separate 5 mL vial, 5-methylthiophene-2,3-dicarboxylic acid (25 mg, 0.13 mmol, 2.8 equiv) was dissolved in DCM (1 mL) and SOCl_2_ (1 mL). The vial was sealed, and the mixture was stirred at 80 °C for 2 h in a preheated oil bath. After evaporation of the solvent under reduced pressure, the resulting acyl chloride was dissolved in DCM (1 mL) and added to the suspension of the deprotected compound **108**. Triethylamine (33 μL, 0.23 mmol, 4.0 equiv) was then added, and the mixture was stirred for 16 additional hours at rt. EtOAc (50 mL) was then added, and the mixture was washed with a 1 M aqueous HCl (25 mL) and brine (25 mL). The organic phase was dried over MgSO_4_, filtered, and then concentrated under reduced pressure. The crude product was purified using reverse-phase HPLC with a gradient 15–75% MeCN/water (containing 0.1% TFA) to give **57** (10 mg, 16 μmol, 35% yield) as a colorless powder. The compound was isolated as a single regioisomer. The formation of a minor regioisomer was detected by LC–MS, but the isolation of a significant amount was not possible on this reaction scale. HRMS (ESI) *m*/*z*: calcd for C_28_H_33_N_4_O_8_S_2_ [M + H]^+^, 617.1740; found, 617.1739. ^1^H NMR (500 MHz, CDCl_3_): δ 8.52 (d, *J* = 8.9 Hz, 1H), 8.50 (d, *J* = 9.8 Hz, 1H), 7.95 (dd, *J* = 7.9, 1.4 Hz, 1H), 7.37 (td, *J* = 7.9, 1.4 Hz, 1H), 7.26–7.24 (m, 1H), 7.20 (td, *J* = 7.6, 1.3 Hz, 1H), 6.73 (s, 1H), 5.28 (ddd, *J* = 11.3, 9.8, 3.9 Hz, 1H), 5.12 (dd, *J* = 10.9, 2.2 Hz, 1H), 5.07 (ddd, *J* = 8.9, 2.6, 2.2 Hz, 1H), 4.92–4.84 (m, 1H), 4.48 (d, *J* = 10.1 Hz, 1H), 4.45 (dd, *J* = 10.9, 2.6 Hz, 1H), 3.64 (d, *J* = 10.1 Hz, 1H), 3.32–3.24 (m, 1H), 3.22–3.13 (m, 1H), 3.16 (s, 3H), 3.06–2.99 (m, 1H), 3.00 (s, 3H), 2.80 (dd, *J* = 14.9, 11.3 Hz, 1H), 2.73–2.61 (m, 1H), 2.54 (s, 3H), 2.04–1.85 (m, 3H). *The COOHproton was not detectable in this spectrum.*^13^C NMR (126 MHz, CDCl_3_): δ 171.7, 171.0, 169.7, 167.5, 167.3, 163.8, 149.6, 144.3, 137.0, 132.5, 132.5, 132.3, 129.4, 127.6, 125.7, 124.7, 67.5, 59.6, 54.0, 50.4, 48.4, 39.1, 37.8, 36.5, 35.7, 26.6, 24.3, 15.8.

#### (4*S*,7*R*)-*N*,*N*-Dimethyl-7-((*S*)-1-(3-(methylsulfonamido)thiophene-2-carbonyl)pyrrolidine-2-carboxamido)-6,10-dioxo-1,3,4,5,6,7,8,10-octahydrobenzo[*j*][1]oxa[8]thia[5]azacyclododecine-4-carboxamide (**58**)

Compound **58** was synthesized following the procedure described for the synthesis of compound **39** using **108** (20 mg, 36 μmol, 1.0 equiv), 3-methanesulfonamidothiophene-2-carboxylic acid (16 mg, 72 μmol, 2.0 equiv), HATU (27 mg, 72 μmol, 2.0 equiv), and DIPEA (25 μL, 0.14 mmol, 4.0 equiv). The crude product was purified using reverse-phase HPLC with a gradient 15–75% MeCN/water to give **58** (6 mg, 9 μmol, 25%) as a colorless powder. HRMS (ESI) *m*/*z*: calcd for C_27_H_33_N_5_NaO_8_S_3_ [M + H]^+^, 674.1389; found, 674.1384. ^1^H NMR (500 MHz, CDCl_3_): δ 10.26 (s, 1H), 7.90 (dd, *J* = 7.9, 1.5 Hz, 1H), 7.80 (d, *J* = 9.3 Hz, 1H), 7.53–7.48 (m, 2H), 7.46–7.43 (m, 1H), 7.41–7.35 (m, 2H), 7.34 (d, *J* = 8.5 Hz, 1H), 5.23–5.15 (m, 2H), 4.92 (dt, *J* = 8.7, 2.3 Hz, 1H), 4.54–4.46 (m, 2H), 4.15 (d, *J* = 9.7 Hz, 1H), 4.10–4.04 (m, 1H), 4.04–3.96 (m, 2H), 3.21 (s, 3H), 3.13–3.07 (m, 2H), 3.07 (s, 3H), 2.76 (s, 3H), 2.40–2.33 (m, 1H), 2.29–2.24 (m, 1H), 2.22–2.15 (m, 1H), 2.11–2.04 (m, 1H). ^13^C NMR (126 MHz, CDCl_3_): δ 171.6, 169.3, 169.0, 168.0, 164.6, 144.2, 136.8, 132.7, 132.5, 131.6, 129.9, 129.6, 127.7, 120.4, 111.6, 66.6, 62.6, 53.4, 49.7, 49.4, 40.4, 37.5, 37.1, 35.8, 35.7, 28.4, 26.0.

#### (4*S*,7*R*)-7-((*S*)-1-Acetylpyrrolidine-2-carboxamido)-*N*,*N*-dimethyl-6,10-dioxo-14-phenyl-1,3,4,5,6,7,8,10-octahydrobenzo[*j*][1]oxa[8]thia[5]azacyclododecine-4-carboxamide (**59**)

A 5 mL vial was charged with compound **109** (35 mg, 56 μmol, 1.0 equiv), XPhos Pd G3 (7 mg, 8 μmol, 15 mol %), K_3_PO_4_ (35 mg, 0.17 mmol, 3.0 equiv), and phenylboronic acid (14 mg, 0.11 mmol, 2.0 equiv). After the tube was evacuated and refilled with argon three times, a mixture of degassed THF/H_2_O 3:1 (0.5 mL) was added. The vial was sealed, and the reaction mixture was stirred at 65 °C for 4 h in a preheated oil bath. The reaction mixture was allowed to cool down to rt, diluted with MeOH (10 mL), and filtered through a 0.45 μm syringe filter. After evaporation of the volatiles under reduced pressure the crude product was purified using reverse-phase HPLC with a gradient 15–75% MeCN/water to give **59** (9 mg, 16 μmol, 29%) as a colorless solid. HRMS (ESI) *m*/*z*: calcd for C_29_H_35_N_4_O_6_S [M + H]^+^, 567.2277; found, 567.2266. ^1^H NMR (500 MHz, CDCl_3_): δ 8.03 (d, *J* = 8.5 Hz, 1H), 7.86–7.83 (m, 1H), 7.50 (d, *J* = 9.0 Hz, 1H), 7.46–7.36 (m, 5H), 7.36–7.31 (m, 2H), 5.36 (dd, *J* = 11.2, 2.5 Hz, 1H), 5.05 (ddd, *J* = 9.0, 8.8, 4.7 Hz, 1H), 4.80 (ddd, *J* = 8.5, 2.5, 2.4 Hz, 1H), 4.47 (dd, *J* = 7.8, 3.3 Hz, 1H), 4.34 (dd, *J* = 11.2, 2.4 Hz, 1H), 4.19 (d, *J* = 10.3 Hz, 1H), 3.88 (d, *J* = 10.3 Hz, 1H), 3.75–3.70 (m, 1H), 3.48–3.39 (m, 1H), 3.00 (s, 3H), 2.91 (dd, *J* = 14.4, 4.8 Hz, 1H), 2.87 (s, 3H), 2.78 (dd, *J* = 14.4, 8.8 Hz, 1H), 2.39–2.31 (m, 1H), 2.24–2.13 (m, 1H), 2.05 (s, 3H), 2.00–1.90 (m, 2H). ^13^C NMR (126 MHz, CDCl_3_): δ 171.8, 171.7, 169.3, 168.7, 168.6, 144.2, 140.4, 134.5, 134.2, 131.4, 131.0, 129.2, 129.2, 128.1, 128.1, 127.5, 127.0, 66.2, 60.1, 53.8, 49.3, 48.3, 37.0, 36.5, 35.8, 34.9, 27.7, 25.2, 22.5.

#### (4*S*,7*R*)-7-((*S*)-1-Acetylpyrrolidine-2-carboxamido)-14-(benzo[*c*][1,2,5]oxadiazol-4-yl)-*N*,*N*-dimethyl-6,10-dioxo-1,3,4,5,6,7,8,10-octahydrobenzo[*j*][1]oxa[8]thia[5]azacyclododecine-4-carboxamide (**60**)

Compound **60** was synthesized following the procedure described for the synthesis of compound **59** using **109** (35 mg, 56 μmol, 1.0 equiv), XPhos Pd G3 (7 mg, 8 μmol, 15 mol %), K_3_PO_4_ (35 mg, 0.17 mmol, 3.0 equiv), and (2,1,3-benzoxadiazol-4-yl)boronic acid (18 mg, 0.11 mmol, 2.0 equiv). The crude product was purified using reverse-phase HPLC with a gradient 15–75% MeCN/water to give **60** (8 mg, 13 μmol, 24% yield) as a colorless powder. HRMS (ESI) *m*/*z*: calcd for C_29_H_33_N_6_O_7_S [M + H]^+^, 609.2131; found, 609.2126. ^1^H NMR (500 MHz, CDCl_3_): δ 7.99 (dd, *J* = 7.7, 1.6 Hz, 1H), 7.89 (d, *J* = 9.0 Hz, 1H), 7.78 (d, *J* = 8.4 Hz, 1H), 7.71 (d, *J* = 9.1 Hz, 1H), 7.62 (d, *J* = 6.6 Hz, 1H), 7.53–7.47 (m, 2H), 7.47–7.42 (m, 1H), 5.37 (dd, *J* = 11.2, 2.6 Hz, 1H), 5.03 (ddd, *J* = 9.1, 8.7, 4.8 Hz, 1H), 4.80 (ddd, *J* = 8.4, 2.6, 2.4 Hz, 1H), 4.40 (dd, *J* = 7.8, 3.9 Hz, 1H), 4.36 (dd, *J* = 11.2, 2.4 Hz, 1H), 4.25 (d, *J* = 10.7 Hz, 1H), 3.79 (d, *J* = 10.7 Hz, 1H), 3.74–3.63 (m, 1H), 3.48–3.41 (m, 1H), 2.97 (s, 3H), 2.89–2.85 (m, 1H), 2.85 (s, 3H), 2.80 (dd, *J* = 14.7, 8.7 Hz, 1H), 2.34–2.24 (m, 1H), 2.20–2.14 (m, 1H), 2.01 (s, 3H), 2.05–1.90 (m, 2H). ^13^C NMR (126 MHz, CDCl_3_): δ 172.0, 171.5, 169.1, 168.6, 168.0, 149.7, 149.3, 137.5, 135.1, 134.5, 132.5, 131.9, 131.5, 131.3, 128.7, 127.5, 116.0, 66.5, 60.3, 53.7, 49.6, 48.3, 37.0, 36.3, 35.8, 35.2, 28.0, 25.3, 22.4.

#### (4*S*,7*R*)-7-((*S*)-1-Acetylpyrrolidine-2-carboxamido)-14-(benzo[*c*][1,2,5]oxadiazol-5-yl)-*N*,*N*-dimethyl-6,10-dioxo-1,3,4,5,6,7,8,10-octahydrobenzo[*j*][1]oxa[8]thia[5]azacyclododecine-4-carboxamide (**61**)

Compound **61** was synthesized following the procedure described for the synthesis of compound **60** using **109** (18 mg, 29 μmol, 1.0 equiv), XPhos Pd G3 (4 mg, 4 μmol, 15 mol %), K_3_PO_4_ (25 mg, 0.12 mmol, 4.0 equiv), and benzo[*c*][1,2,5]oxadiazol-5-ylboronic acid (14 mg, 87 μmol, 3.0 equiv). The crude product was purified using reverse-phase HPLC with a gradient 15–75% MeCN/water to give **61** (6 mg, 10 μmol, 33% yield) as a colorless powder. HRMS (ESI) *m*/*z*: calcd for C_29_H_33_N_6_O_7_S [M + H]^+^, 609.2131; found, 609.2113. ^1^H NMR (500 MHz, CDCl_3_): δ 8.06 (d, *J* = 8.7 Hz, 1H), 7.98 (dd, *J* = 7.6, 1.7 Hz, 1H), 7.93 (s, 1H), 7.90 (dd, *J* = 9.2, 1.1 Hz, 1H), 7.60–7.53 (m, 2H), 7.46 (t, *J* = 7.6 Hz, 1H), 7.41 (dd, *J* = 7.6, 1.7 Hz, 1H), 5.38 (dd, *J* = 11.2, 2.6 Hz, 1H), 5.08 (td, *J* = 8.9, 4.6 Hz, 1H), 4.86 (ddd, *J* = 8.7, 2.6, 2.3 Hz, 1H), 4.50 (dd, *J* = 7.9, 3.1 Hz, 1H), 4.40 (dd, *J* = 11.2, 2.3 Hz, 1H), 4.29 (d, *J* = 10.6 Hz, 1H), 3.85 (d, *J* = 10.6 Hz, 1H), 3.77–3.70 (m, 1H), 3.53–3.42 (m, 1H), 3.02 (s, 3H), 2.94 (dd, *J* = 14.6, 4.6 Hz, 1H), 2.89 (s, 3H), 2.76 (dd, *J* = 14.6, 8.9 Hz, 1H), 2.43–2.35 (m, 1H), 2.28–2.15 (m, 1H), 2.08 (s, 3H), 2.09–1.98 (m, 1H), 2.01–1.91 (m, 1H). ^13^C NMR (126 MHz, CDCl_3_): δ 171.9, 171.6, 169.1, 168.5, 168.0, 149.1, 148.4, 143.8, 141.7, 134.4, 134.3, 133.5, 132.3, 131.9, 127.7, 115.9, 115.8, 66.7, 60.0, 53.6, 49.3, 48.3, 37.0, 36.4, 35.8, 35.1, 27.6, 25.2, 22.6.

#### (4*S*,7*R*)-7-((*S*)-1-Acetylpyrrolidine-2-carboxamido)-14-(5-cyanothiophen-2-yl)-*N*,*N*-dimethyl-6,10-dioxo-1,3,4,5,6,7,8,10-octahydrobenzo[*j*][1]oxa[8]thia[5]azacyclododecine-4-carboxamide (**62**)

Compound **62** was synthesized following the procedure described for the synthesis of compound **59** using **109** (35 mg, 56 μmol, 1.0 equiv), XPhos Pd G3 (7 mg, 8 μmol, 15 mol %), K_3_PO_4_ (35 mg, 0.17 mmol, 3.0 equiv), and 5-cyanothiophene-2-boronic acid (17 mg, 0.11 mmol, 2.0 equiv). The crude product was purified using reverse-phase HPLC with a gradient 15–75% MeCN/water to give **62** (4 mg, 7 μmol, 13% yield) as a colorless powder. HRMS (ESI) *m*/*z*: calcd for C_28_H_32_N_5_O_6_S_2_ [M + H]^+^, 598.1787; found, 598.1794. ^1^H NMR (500 MHz, CDCl_3_): δ 8.00 (d, *J* = 9.0 Hz, 1H), 7.98 (dd, *J* = 7.7, 1.5 Hz, 1H), 7.64 (d, *J* = 3.8 Hz, 1H), 7.59 (d, *J* = 9.1 Hz, 1H), 7.51 (dd, *J* = 7.7, 1.5 Hz, 1H), 7.42 (t, *J* = 7.7 Hz, 1H), 7.39 (d, *J* = 3.8 Hz, 1H), 5.38 (dd, *J* = 11.1, 2.6 Hz, 1H), 5.13 (td, *J* = 9.1, 4.6 Hz, 1H), 4.88 (dt, *J* = 9.0, 2.4 Hz, 1H), 4.48 (dd, *J* = 7.7, 3.2 Hz, 1H), 4.38 (dd, *J* = 11.1, 2.4 Hz, 1H), 4.36 (d, *J* = 10.4 Hz, 1H), 3.92 (d, *J* = 10.4 Hz, 1H), 3.77–3.71 (m, 1H), 3.52–3.44 (m, 1H), 3.06 (s, 3H), 3.05–2.98 (m, 1H), 2.93 (s, 3H), 2.91 (dd, *J* = 14.5, 9.1 Hz, 1H), 2.42–2.35 (m, 1H), 2.29–2.16 (m, 1H), 2.10 (s, 3H), 2.08–1.92 (m, 2H). ^13^C NMR (126 MHz, CDCl_3_): δ 171.9, 171.6, 169.2, 168.6, 167.7, 148.5, 137.3, 135.5, 135.2, 134.1, 133.0, 132.0, 128.1, 127.6, 114.0, 110.2, 66.8, 60.1, 53.5, 49.5, 48.3, 37.1, 36.5, 35.8, 35.1, 27.6, 25.3, 22.6.

#### 3-((4*S*,7*R*)-7-((*S*)-1-Acetylpyrrolidine-2-carboxamido)-4-(dimethylcarbamoyl)-6,10-dioxo-1,3,4,5,6,7,8,10-octahydrobenzo[*j*][1]oxa[8]thia[5]azacyclododecin-14-yl)benzoic Acid (**63**)

Compound **63** was synthesized following the procedure described for the synthesis of compound **59** using **109** (35 mg, 56 μmol, 1.0 equiv), XPhos Pd G3 (7 mg, 8 μmol, 15 mol %), K_3_PO_4_ (35 mg, 0.17 mmol, 3.0 equiv), and 3-carboxyphenylboronic acid (23 mg, 0.11 mmol, 2.0 equiv). The crude product was purified using reverse-phase HPLC with a gradient 15–75% MeCN (containing 0.1% TFA)/water to give **63** (8 mg, 13 μmol, 23% yield) as a colorless powder. HRMS (ESI) *m*/*z*: calcd for C_30_H_35_N_4_O_8_S [M + H]^+^, 611.2176; found, 611.2151. ^1^H NMR (500 MHz, CDCl_3_): δ 8.16 (d, *J* = 8.4 Hz, 1H), 8.08 (s, 1H), 8.02 (d, *J* = 7.7 Hz, 1H), 7.86 (d, *J* = 7.7 Hz, 1H), 7.64 (d, *J* = 7.6 Hz, 1H), 7.58 (d, *J* = 9.0 Hz, 1H), 7.47 (t, *J* = 7.7 Hz, 1H), 7.30 (t, *J* = 7.7 Hz, 1H), 7.26–7.22 (m, 1H), 5.34 (dd, *J* = 11.3, 2.4 Hz, 1H), 5.08 (td, *J* = 9.0, 4.6 Hz, 1H), 4.82 (dt, *J* = 8.4, 2.4 Hz, 1H), 4.54 (dd, *J* = 7.9, 3.4 Hz, 1H), 4.39 (dd, *J* = 11.3, 2.4 Hz, 1H), 4.20 (d, *J* = 10.4 Hz, 1H), 3.80 (d, *J* = 10.4 Hz, 1H), 3.73–3.68 (m, 1H), 3.50–3.44 (m, 1H), 3.00 (s, 3H), 2.93–2.86 (m, 1H), 2.87 (s, 3H), 2.76 (dd, *J* = 14.5, 9.0 Hz, 1H), 2.36–2.29 (m, 1H), 2.25–2.16 (m, 1H), 2.09 (s, 3H), 2.08–1.94 (m, 2H). *The COOHproton was not detectable in this spectrum.*^13^C NMR (126 MHz, CDCl_3_): δ 172.3, 171.8, 169.5, 169.2, 168.8, 168.3, 142.9, 140.6, 134.5, 134.2, 134.1, 131.5, 131.2, 130.6, 129.3, 129.2, 128.3, 127.1, 66.2, 60.1, 53.8, 49.4, 48.4, 37.1, 36.5, 35.9, 35.0, 28.0, 25.2, 22.4.

#### 4-((4*S*,7*R*)-7-((*S*)-1-Acetylpyrrolidine-2-carboxamido)-4-(dimethylcarbamoyl)-6,10-dioxo-1,3,4,5,6,7,8,10-octahydrobenzo[*j*][1]oxa[8]thia[5]azacyclododecin-14-yl)benzoic Acid (**64**)

Compound **64** was synthesized following the procedure described for the synthesis of compound **59** using **109** (65 mg, 0.11 mmol, 1.0 equiv), XPhos Pd G3 (14 mg, 16 μmol, 15 mol %), K_3_PO_4_ (70 mg, 0.33 mmol, 3.0 equiv), and 4-carboxyphenylboronic acid (36 mg, 0.22 mmol, 2.0 equiv). The crude product was purified using reverse-phase HPLC with a gradient 15–75% MeCN (containing 0.1% TFA)/water to give **64** (17 mg, 28 μmol, 25% yield) as a colorless powder. HRMS (ESI) *m*/*z*: calcd for C_30_H_35_N_4_O_8_S [M + H]^+^, 611.2176; found, 611.2157. ^1^H NMR (500 MHz, CDCl_3_): δ 8.17 (d, *J* = 8.6 Hz, 1H), 8.08–8.03 (m, 2H), 7.94 (dd, *J* = 7.7, 1.6 Hz, 1H), 7.61 (d, *J* = 9.2 Hz, 1H), 7.54–7.48 (m, 2H), 7.38 (t, *J* = 7.7 Hz, 1H), 7.31 (dd, *J* = 7.7, 1.6 Hz, 1H), 5.40 (dd, *J* = 11.3, 2.6 Hz, 1H), 5.13 (ddd, *J* = 9.4, 9.2, 4.5 Hz, 1H), 4.89 (ddd, *J* = 8.6, 2.6, 2.4 Hz, 1H), 4.54 (dd, *J* = 7.8, 3.7 Hz, 1H), 4.40 (dd, *J* = 11.3, 2.4 Hz, 1H), 4.35 (d, *J* = 10.3 Hz, 1H), 3.78 (d, *J* = 10.3 Hz, 1H), 3.76–3.71 (m, 1H), 3.52–3.47 (m, 1H), 3.04 (s, 3H), 3.00–2.90 (m, 1H), 2.91 (s, 3H), 2.77 (dd, *J* = 14.6, 9.4 Hz, 1H), 2.38–2.30 (m, 1H), 2.29–2.16 (m, 1H), 2.12 (s, 3H), 2.04–1.96 (m, 2H). *The COOHproton was not detectable in this spectrum.*^13^C NMR (126 MHz, CDCl_3_): δ 172.2, 171.8, 169.4, 169.4, 168.7, 168.3, 145.7, 143.2, 134.2, 133.8, 131.7, 131.4, 129.8, 129.8, 129.3, 129.3, 128.8, 127.2, 66.5, 60.1, 53.7, 49.6, 48.4, 37.1, 36.5, 35.9, 35.2, 28.0, 25.2, 22.5.

#### Methyl 4-((4*S*,7*R*)-7-((*S*)-1-Acetylpyrrolidine-2-carboxamido)-4-(dimethylcarbamoyl)-6,10-dioxo-1,3,4,5,6,7,8,10-octahydrobenzo[*j*][1]oxa[8]thia[5]azacyclododecin-14-yl)benzoate (**65**)

Compound **65** was synthesized following the procedure described for the synthesis of compound **59** using **109** (20 mg, 32 μmol, 1.0 equiv), XPhos Pd G3 (4 mg, 5 μmol, 15 mol %), K_3_PO_4_ (27 mg, 0.13 mmol, 4.0 equiv), and 4-methoxycarbonylphenylboronic acid (15 mg, 96 μmol, 3.0 equiv). The crude product was purified using reverse-phase HPLC with a gradient 15–75% MeCN/water to give **65** (7 mg, 11 μmol, 35% yield) as a colorless powder. HRMS (ESI) *m*/*z*: calcd for C_31_H_37_N_4_O_8_S [M + H]^+^, 625.2332; found, 625.2314. ^1^H NMR (500 MHz, CDCl_3_): δ 8.13–8.09 (m, 2H), 8.07 (d, *J* = 8.5 Hz, 1H), 7.92 (dd, *J* = 7.5, 1.8 Hz, 1H), 7.58–7.53 (m, 2H), 7.51 (d, *J* = 9.0 Hz, 1H), 7.44–7.34 (m, 2H), 5.37 (dd, *J* = 11.2, 2.6 Hz, 1H), 5.07 (ddd, *J* = 9.0, 8.9, 4.6 Hz, 1H), 4.84 (ddd, *J* = 8.5, 2.6, 2.4 Hz, 1H), 4.50 (dd, *J* = 8.0, 3.0 Hz, 1H), 4.38 (dd, *J* = 11.2, 2.4 Hz, 1H), 4.21 (d, *J* = 10.4 Hz, 1H), 3.98 (s, 3H), 3.83 (d, *J* = 10.4 Hz, 1H), 3.80–3.70 (m, 1H), 3.50–3.42 (m, 1H), 3.02 (s, 3H), 2.93 (dd, *J* = 14.5, 4.6 Hz, 1H), 2.89 (s, 3H), 2.76 (dd, *J* = 14.5, 8.9 Hz, 1H), 2.42–2.34 (m, 1H), 2.27–2.16 (m, 1H), 2.08 (s, 3H), 2.05–1.97 (m, 1H), 2.00–1.91 (m, 1H). ^13^C NMR (126 MHz, CDCl_3_): δ 171.8, 171.7, 169.2, 168.6, 168.4, 167.0, 145.2, 143.2, 134.3, 133.8, 131.5, 129.3 (4C), 127.2, 66.4, 60.1, 53.7, 52.3, 49.3, 48.3, 37.0, 36.5, 35.8, 35.0, 27.6, 25.2, 22.5.

#### 2-(4-((4*S*,7*R*)-7-((*S*)-1-Acetylpyrrolidine-2-carboxamido)-4-(dimethylcarbamoyl)-6,10-dioxo-1,3,4,5,6,7,8,10-octahydrobenzo[*j*][1]oxa[8]thia[5]azacyclododecin-14-yl)phenyl)acetic Acid (**66**)

Compound **66** was synthesized following the procedure described for the synthesis of compound **59** using **109** (20 mg, 32 μmol, 1.0 equiv), XPhos Pd G3 (4 mg, 5 μmol, 15 mol %), K_3_PO_4_ (27 mg, 0.13 mmol, 4.0 equiv), and 4-(carboxymethyl)phenylboronic acid (17 mg, 96 μmol, 3.0 equiv). The crude product was purified using reverse-phase HPLC with a gradient 15–75% MeCN (containing 0.1% TFA)/water to give **66** (6 mg, 10 μmol, 31% yield) as a colorless powder. HRMS (ESI) *m*/*z*: calcd for C_31_H_37_N_4_O_8_S [M + H]^+^, 625.2332; found, 625.2316. ^1^H NMR (500 MHz, CDCl_3_): δ 8.05 (d, *J* = 8.3 Hz, 1H), 7.87 (dd, *J* = 6.7, 2.5 Hz, 1H), 7.60 (d, *J* = 9.0 Hz, 1H), 7.42–7.37 (m, 2H), 7.37–7.30 (m, 4H), 5.37 (dd, *J* = 11.3, 2.6 Hz, 1H), 5.09 (td, *J* = 9.0, 4.7 Hz, 1H), 4.83 (ddd, *J* = 8.3, 2.6, 2.3 Hz, 1H), 4.55–4.46 (m, 1H), 4.38 (dd, *J* = 11.3, 2.3 Hz, 1H), 4.21 (d, *J* = 10.4 Hz, 1H), 3.89 (d, *J* = 10.4 Hz, 1H), 3.77–3.71 (m, 1H), 3.69–3.66 (m, 2H), 3.52–3.44 (m, 1H), 3.03 (s, 3H), 2.97–2.91 (m, 1H), 2.89 (s, 3H), 2.84–2.79 (m, 1H), 2.36–2.30 (m, 1H), 2.24–2.18 (m 1H), 2.10 (s, 3H), 2.06–1.96 (m, 2H). *The COOHproton was not detectable in this spectrum.*^13^C NMR (126 MHz, CDCl_3_): δ 174.6, 172.0, 171.9, 169.4, 168.7, 168.7, 143.7, 139.3, 134.4, 134.3, 132.9, 131.4, 131.1, 129.4, 129.4, 129.1, 129.1, 127.1, 66.2, 60.2, 53.9, 49.4, 48.4, 40.5, 37.1, 36.3, 35.9, 34.8, 28.0, 25.1, 22.3.

#### 5-((4*S*,7*R*)-7-((*S*)-1-Acetylpyrrolidine-2-carboxamido)-4-(dimethylcarbamoyl)-6,10-dioxo-1,3,4,5,6,7,8,10-octahydrobenzo[*j*][1]oxa[8]thia[5]azacyclododecin-14-yl)thiophene-2-carboxylic Acid (**67**)

Compound **67** was synthesized following the procedure described for the synthesis of compound **59** using **109** (20 mg, 32 μmol, 1.0 equiv), XPhos Pd G3 (4 mg, 5 μmol, 15 mol %), K_3_PO_4_ (27 mg, 0.13 mmol, 4.0 equiv), and 5-carboxythiophene-2-boronic acid (16 mg, 96 μmol, 3.0 equiv). The crude product was purified using reverse-phase HPLC with a gradient 15–75% MeCN (containing 0.1% TFA)/water to give **67** (7 mg, 11 μmol, 34% yield) as a colorless powder. HRMS (ESI) *m*/*z*: calcd for C_28_H_33_N_4_O_8_S_2_ [M + H]^+^, 617.1740; found, 617.1745. ^1^H NMR (500 MHz, CDCl_3_): δ 8.19 (d, *J* = 8.6 Hz, 1H), 7.98 (dd, *J* = 7.7, 1.6 Hz, 1H), 7.73 (d, *J* = 9.4 Hz, 1H), 7.56 (d, *J* = 3.9 Hz, 1H), 7.45 (dd, *J* = 7.7, 1.6 Hz, 1H), 7.42–7.34 (m, 2H), 5.41 (dd, *J* = 11.2, 2.6 Hz, 1H), 5.21 (ddd, *J* = 10.0, 9.4, 4.3 Hz, 1H), 4.93 (ddd, *J* = 8.6, 2.6, 2.2 Hz, 1H), 4.65 (d, *J* = 10.2 Hz, 1H), 4.55 (dd, *J* = 8.0, 3.3 Hz, 1H), 4.42 (dd, *J* = 11.2, 2.2 Hz, 1H), 3.95 (d, *J* = 10.2 Hz, 1H), 3.79–3.71 (m, 1H), 3.56–3.50 (m, 1H), 3.12–3.06 (m, 1H), 3.06 (s, 3H), 2.95 (s, 3H), 2.91 (dd, *J* = 14.7, 10.0 Hz, 1H), 2.31–2.22 (m, 2H), 2.17 (s, 3H), 2.12–1.98 (m, 2H). *The COOHproton was not detectable in this spectrum.*^13^C NMR (126 MHz, CDCl_3_): δ 172.6, 171.9, 169.4, 168.8, 167.8, 164.6, 148.8, 135.6, 135.3, 135.2, 134.2, 133.5, 132.7, 131.7, 128.6, 127.3, 66.8, 60.2, 53.6, 50.0, 48.6, 37.2, 36.4, 36.0, 35.7, 28.4, 25.2, 22.4.

#### (4*S*,7*R*)-14-(4-(1*H*-Tetrazol-5-yl)phenyl)-7-((*S*)-1-acetylpyrrolidine-2-carboxamido)-*N*,*N*-dimethyl-6,10-dioxo-1,3,4,5,6,7,8,10-octahydrobenzo[*j*][1]oxa[8]thia[5]azacyclododecine-4-carboxamide (**68**)

A 5 mL vial was charged with compound **109** (18 mg, 29 μmol, 1.0 equiv), XPhos Pd G3 (4 mg, 5 μmol, 0.15 equiv), K_3_PO_4_ (25 mg, 0.12 mmol, 4.0 equiv), and 4-(1*H*-tetrazol-5-yl)phenylboronic acid (16 mg, 87 μmol, 3.0 equiv). After the tube was evacuated and refilled with argon three times, a mixture of degassed 1,4-dioxane:H_2_O 1:1 (0.5 mL) was added. The vial was sealed, and the reaction mixture was heated in a microwave reactor at 120 °C for 90 min. The reaction mixture was cooled down to rt, diluted with MeOH (10 mL), and filtered through a 0.45 μm syringe filter. After evaporation of the volatiles under reduced pressure, the crude product was purified using reverse-phase HPLC with a gradient 15–75% MeCN (containing 0.1% TFA)/water to give **68** (3 mg, 5 μmol, 17%) as a colorless solid. HRMS (ESI) *m*/*z*: calcd for C_30_H_35_N_8_O_6_S [M + H]^+^, 635.2400; found, 635.2417. ^1^H NMR (500 MHz, CDCl_3_): δ 8.08 (d, *J* = 7.9 Hz, 2H), 8.01 (d, *J* = 8.1 Hz, 1H), 7.94 (d, *J* = 9.3 Hz, 1H), 7.84 (dd, *J* = 7.7, 1.3 Hz, 1H), 7.57 (d, *J* = 7.9 Hz, 2H), 7.43–7.33 (m, 1H), 7.32–7.29 (m 1H), 5.40 (dd, *J* = 11.5, 2.6 Hz, 1H), 5.08 (td, *J* = 9.3, 4.8 Hz, 1H), 4.85 (ddd, *J* = 8.1, 2.6, 2.3 Hz, 1H), 4.47 (dd, *J* = 7.8, 4.3 Hz, 1H), 4.43 (dd, *J* = 11.6, 2.3 Hz, 1H), 4.15 (d, *J* = 10.4 Hz, 1H), 3.76 (d, *J* = 10.4 Hz, 1H), 3.74–3.66 (m, 1H), 3.52–3.46 (m, 1H), 3.01 (s, 3H), 2.98–2.93 (m, 1H), 2.93 (s, 3H), 2.91–2.84 (m, 1H), 2.29–2.18 (m, 2H), 2.17–2.06 (m, 1H), 2.04 (s, 3H), 2.00–1.94 (m, 1H). *The tetrazole NHproton was not detectable in this spectrum.*^13^C NMR (126 MHz, CDCl_3_): δ 172.3, 171.4, 169.7, 168.9, 168.3, 156.2, 143.2, 142.7, 134.2, 134.1, 131.4, 131.3, 130.0, 130.0, 127.4, 127.2, 127.2, 123.3, 66.1, 60.4, 54.1, 49.7, 48.5, 37.3, 36.5, 36.2, 35.2, 28.6, 25.2, 22.4.

#### (4*S*,7*R*)-7-((*S*)-1-Acetylpyrrolidine-2-carboxamido)-*N*,*N*-dimethyl-14-(4-nitrophenyl)-6,10-dioxo-1,3,4,5,6,7,8,10-octahydrobenzo[*j*][1]oxa[8]thia[5]azacyclododecine-4-carboxamide (**69**)

Compound **69** was synthesized following the procedure described for the synthesis of compound **59** using **109** (20 mg, 32 μmol, 1.0 equiv), XPhos Pd G3 (4 mg, 5 μmol, 15 mol %), K_3_PO_4_ (27 mg, 0.13 mmol, 4.0 equiv), and 4-nitrophenyl boronic acid (16 mg, 96 μmol, 3.0 equiv). The crude product was purified using reverse-phase HPLC with a gradient 15–75% MeCN/water to give **69** (6 mg, 10 μmol, 31% yield) as a colorless powder. HRMS (ESI) *m*/*z*: calcd for C_29_H_34_N_5_O_8_S [M + H]^+^, 612.2128; found, 612.2142. ^1^H NMR (500 MHz, CDCl_3_): δ 8.31–8.26 (m, 2H), 8.02 (d, *J* = 8.6 Hz, 1H), 7.93 (dd, *J* = 7.7, 1.5 Hz, 1H), 7.67–7.60 (m, 2H), 7.52 (d, *J* = 9.0 Hz, 1H), 7.41 (t, *J* = 7.7 Hz, 1H), 7.33 (dd, *J* = 7.7, 1.5 Hz, 1H), 5.35 (dd, *J* = 11.2, 2.6 Hz, 1H), 5.06 (ddd, *J* = 9.0, 8.9, 4.6 Hz, 1H), 4.83 (ddd, *J* = 8.6, 2.6, 2.4 Hz, 1H), 4.47 (dd, *J* = 7.8, 3.1 Hz, 1H), 4.37 (dd, *J* = 11.2, 2.4 Hz, 1H), 4.20 (d, *J* = 10.5 Hz, 1H), 3.76 (d, *J* = 10.5 Hz, 1H) 3.74–3.67 (m, 1H), 3.49–3.40 (m, 1H), 3.00 (s, 3H), 2.91 (dd, *J* = 14.5, 4.6 Hz, 1H), 2.87 (s, 3H), 2.75 (dd, *J* = 14.5, 8.9 Hz, 1H), 2.41–2.32 (m, 1H), 2.25–2.14 (m, 1H), 2.06 (s, 3H), 2.03–1.89 (m, 2H). ^13^C NMR (126 MHz, CDCl_3_): δ 171.9, 171.6, 169.2, 168.6, 168.2, 147.5, 147.2, 141.9, 134.2, 133.6, 132.1, 131.7, 130.3, 130.3, 127.4, 123.3, 123.3, 66.6, 60.1, 53.6, 49.3, 48.3, 37.0, 36.5, 35.8, 35.0, 27.6, 25.2, 22.5.

#### (4*S*,7*R*)-7-((*S*)-1-Acetylpyrrolidine-2-carboxamido)-*N*,*N*-dimethyl-14-(4-(methylsulfonyl)phenyl)-6,10-dioxo-1,3,4,5,6,7,8,10-octahydrobenzo[*j*][1]oxa[8]thia[5]azacyclododecine-4-carboxamide (**70**)

Compound **70** was synthesized following the procedure described for the synthesis of compound **59** using **109** (20 mg, 32 μmol, 1.0 equiv), XPhos Pd G3 (4 mg, 5 μmol, 15 mol %), K_3_PO_4_ (27 mg, 0.13 mmol, 4.0 equiv), and 4-methanesulfonyl)phenylboronic acid (19 mg, 96 μmol, 3.0 equiv). The crude product was purified using reverse-phase HPLC with a gradient 15–75% MeCN/water to give **70** (7 mg, 11 μmol, 34% yield) as a colorless powder. HRMS (ESI) *m*/*z*: calcd for C_30_H_37_N_4_O_8_S_2_ [M + H]^+^, 645.2053; found, 645.2063. ^1^H NMR (500 MHz, CDCl_3_): δ 8.02 (d, *J* = 8.8 Hz, 1H), 8.00–7.96 (m, 2H), 7.93 (dd, *J* = 7.7, 1.5 Hz, 1H), 7.70–7.65 (m, 2H), 7.52 (d, *J* = 9.0 Hz, 1H), 7.40 (t, *J* = 7.7 Hz, 1H), 7.31 (dd, *J* = 7.7, 1.5 Hz, 1H), 5.36 (dd, *J* = 11.3, 2.6 Hz, 1H), 5.06 (td, *J* = 9.0, 4.6 Hz, 1H), 4.83 (ddd, *J* = 8.8, 2.6, 2.4 Hz, 1H), 4.47 (dd, *J* = 7.9, 3.0 Hz, 1H), 4.36 (dd, *J* = 11.3, 2.4 Hz, 1H), 4.21 (d, *J* = 10.5 Hz, 1H), 3.76 (d, *J* = 10.5 Hz, 1H) 3.73–3.68 (m, 1H), 3.51–3.40 (m, 1H), 3.15 (s, 3H), 3.01 (s, 3H), 2.93 (dd, *J* = 14.5, 4.6 Hz, 1H), 2.88 (s, 3H), 2.75 (dd, *J* = 14.5, 9.0 Hz, 1H), 2.41–2.32 (m, 1H), 2.23–2.14 (m, 1H), 2.06 (s, 3H), 2.02–1.90 (m, 2H). ^13^C NMR (126 MHz, CDCl_3_): δ 171.9, 171.6, 169.2, 168.5, 168.2, 146.2, 142.2, 139.8, 134.2, 133.8, 132.0, 131.7, 130.3, 130.3, 127.4, 127.2, 127.2, 66.6, 60.0, 53.6, 49.3, 48.3, 44.6, 37.0, 36.5, 35.8, 35.0, 27.6, 25.2, 22.6.

#### (4*S*,7*R*)-14-(4-Acetylphenyl)-7-((*S*)-1-acetylpyrrolidine-2-carboxamido)-*N*,*N*-dimethyl-6,10-dioxo-1,3,4,5,6,7,8,10-octahydrobenzo[*j*][1]oxa[8]thia[5]azacyclododecine-4-carboxamide (**71**)

Compound **71** was synthesized following the procedure described for the synthesis of compound **59** using **109** (18 mg, 29 μmol, 1.0 equiv), XPhos Pd G3 (4 mg, 5 μmol, 15 mol %), K_3_PO_4_ (25 mg, 0.11 mmol, 4.0 equiv), and 4-acetylphenylboronic acid (14 mg, 87 μmol, 3.0 equiv). The crude product was purified using reverse-phase HPLC with a gradient 15–75% MeCN/water to give **71** (7 mg, 12 μmol, 41% yield) as a colorless powder. HRMS (ESI) *m*/*z*: calcd for C_31_H_37_N_4_O_7_S [M + H]^+^, 609.2383; found, 609.2369. ^1^H NMR (500 MHz, CDCl_3_): δ 8.04–7.98 (m, 3H), 7.90 (dd, *J* = 7.6, 1.6 Hz, 1H), 7.58–7.54 (m, 2H), 7.52 (d, *J* = 9.1 Hz, 1H), 7.38 (t, *J* = 7.6 Hz, 1H), 7.34 (dd, *J* = 7.6, 1.6 Hz, 1H), 5.36 (dd, *J* = 11.2, 2.5 Hz, 1H), 5.05 (ddd, *J* = 9.1, 8.9, 4.8 Hz, 1H), 4.82 (ddd, *J* = 8.6, 2.5. 2.4 Hz, 1H), 4.47 (dd, *J* = 7.8, 3.3 Hz, 1H), 4.35 (dd, *J* = 11.2, 2.4 Hz, 1H), 4.21 (d, *J* = 10.4 Hz, 1H), 3.81 (d, *J* = 10.4 Hz, 1H), 3.75–3.69 (m, 1H), 3.48–3.40 (m, 1H), 3.00 (s, 3H), 2.91 (dd, *J* = 14.5, 4.8 Hz, 1H), 2.87 (s, 3H), 2.76 (dd, *J* = 14.5, 8.9 Hz, 1H), 2.67 (s, 3H), 2.39–2.32 (m, 1H), 2.23–2.14 (m, 1H), 2.06 (s, 3H), 2.02–1.89 (m, 2H). ^13^C NMR (126 MHz, CDCl_3_): δ 197.8, 171.8, 171.7, 169.3, 168.6, 168.4, 145.4, 143.1, 136.3, 134.2, 133.8, 131.6, 131.6, 129.5, 129.5, 128.1, 128.1, 127.2, 66.4, 60.1, 53.7, 49.3, 48.3, 37.0, 36.5, 35.8, 35.0, 27.7, 26.7, 25.2, 22.5.

#### 4-((4*S*,7*R*)-7-((*S*)-1-Acetylpyrrolidine-2-carboxamido)-4-(dimethylcarbamoyl)-6,10-dioxo-1,3,4,5,6,7,8,10-octahydrobenzo[*j*][1]oxa[8]thia[5]azacyclododecin-14-yl)-2-methylbenzoic Acid (**72**)

Compound **72** was synthesized following the procedure described for the synthesis of compound **59** using **109** (35 mg, 56 μmol, 1.0 equiv), XPhos Pd G3 (7 mg, 8 μmol, 15 mol %), K_3_PO_4_ (47 mg, 0.22 mmol, 4.0 equiv), and 4-carboxy-3-methylphenylboronic acid (20 mg, 0.11 mmol, 2.0 equiv). The crude product was purified using reverse-phase HPLC with a gradient 15–75% MeCN (containing 0.1% TFA)/water to give **72** (10 mg, 16 μmol, 29% yield) as a colorless powder. HRMS (ESI) *m*/*z*: calcd for C_31_H_37_N_4_O_8_S [M + H]^+^, 625.2332; found, 625.2319. ^1^H NMR (500 MHz, CDCl_3_): δ 8.13 (d, *J* = 8.4 Hz, 1H), 8.05 (d, *J* = 8.2 Hz, 1H), 7.90 (dd, *J* = 7.6, 1.6 Hz, 1H), 7.61 (d, *J* = 9.0 Hz, 1H), 7.39–7.30 (m, 4H), 5.39 (dd, *J* = 11.3, 2.6 Hz, 1H), 5.11 (ddd, *J* = 9.1, 9.0, 4.6 Hz, 1H), 4.87 (ddd, *J* = 8.4, 2.6, 2.3 Hz, 1H), 4.54 (dd, *J* = 7.8, 3.7 Hz, 1H), 4.40 (dd, *J* = 11.3, 2.3 Hz, 1H), 4.26 (d, *J* = 10.4 Hz, 1H), 3.86 (d, *J* = 10.4 Hz, 1H), 3.79–3.71 (m, 1H), 3.54–3.45 (m, 1H), 3.05 (s, 3H), 2.95 (dd, *J* = 14.5, 4.6 Hz, 1H), 2.91 (s, 3H), 2.80 (dd, *J* = 14.5, 9.1 Hz, 1H), 2.67 (s, 3H), 2.40–2.33 (m, 1H), 2.27–2.19 (m, 1H), 2.12 (s, 3H), 2.07–1.94 (m, 2H). *The COOHproton was not detectable in this spectrum.*^13^C NMR (126 MHz, CDCl_3_): δ 172.2, 171.8, 170.7, 169.4, 168.7, 168.5, 144.9, 143.1, 141.0, 134.2, 133.8, 132.7, 131.5, 131.5, 131.3, 127.6, 127.2, 126.7, 66.4, 60.1, 53.8, 49.4, 48.4, 37.1, 36.3, 35.9, 34.9, 27.9, 25.2, 22.4, 22.1.

#### 4-((4*S*,7*R*)-7-((*S*)-1-Acetylpyrrolidine-2-carboxamido)-4-(dimethylcarbamoyl)-6,10-dioxo-1,3,4,5,6,7,8,10-octahydrobenzo[*j*][1]oxa[8]thia[5]azacyclododecin-14-yl)-2-methoxybenzoic Acid (**73**)

Compound **73** was synthesized following the procedure described for the synthesis of compound **59** using **109** (18 mg, 29 μmol, 1.0 equiv), XPhos Pd G3 (4 mg, 5 μmol, 15 mol %), K_3_PO_4_ (23 mg, 0.11 mmol, 4.0 equiv), and 4-borono-2-methoxybenzoic acid (16 mg, 87 μmol, 3.0 equiv). The crude product was purified using reverse-phase HPLC with a gradient 15–75% MeCN (containing 0.1% TFA)/water to give **73** (6 mg, 9 μmol, 31% yield) as a colorless powder. HRMS (ESI) *m*/*z*: calcd for C_31_H_37_N_4_O_9_S [M + H]^+^, 641.2281; found, 641.2263. ^1^H NMR (600 MHz, CDCl_3_): δ 8.24 (d, *J* = 8.0 Hz, 1H), 7.95 (d, *J* = 8.5 Hz, 1H), 7.93 (dd, *J* = 7.8, 1.4 Hz, 1H), 7.65 (d, *J* = 9.0 Hz, 1H), 7.43 (t, *J* = 7.8 Hz, 1H), 7.38 (dd, *J* = 7.8, 1.4 Hz, 1H), 7.35 (s, 1H), 7.18 (d, *J* = 8.0, 1H), 5.39 (dd, *J* = 11.2, 2.7 Hz, 1H), 5.11 (ddd, *J* = 9.1, 9.0, 4.6 Hz, 1H), 4.86 (ddd, *J* = 8.5, 2.7, 2.3 Hz, 1H), 4.48 (dd, *J* = 7.9, 3.8 Hz, 1H), 4.40 (dd, *J* = 11.2, 2.3 Hz, 1H), 4.26 (d, *J* = 10.3 Hz, 1H), 4.12 (s, 3H), 3.81 (d, *J* = 10.3 Hz, 1H), 3.77–3.70 (m, 1H), 3.52–3.46 (m, 1H), 3.05 (s, 3H), 2.96 (dd, *J* = 14.5, 4.6 Hz, 1H), 2.91 (s, 3H), 2.83 (dd, *J* = 14.5, 9.1 Hz, 1H), 2.38–2.32 (m, 1H), 2.26–2.19 (m, 1H), 2.10 (s, 3H), 2.06–1.96 (m, 2H). *The COOHproton was not detectable in this spectrum.*^13^C NMR (151 MHz, CDCl_3_): δ 172.1, 171.7, 169.3, 168.7, 168.3, 165.1, 157.4, 147.5, 142.5, 133.9, 133.7, 133.6, 131.9, 131.7, 127.4, 123.1, 116.8, 112.9, 66.5, 60.2, 56.9, 53.8, 49.4, 48.4, 37.1, 36.3, 35.9, 35.1, 27.9, 25.2, 22.4.

#### 4-((4*S*,7*R*)-7-((*S*)-1-Acetylpyrrolidine-2-carboxamido)-4-(dimethylcarbamoyl)-6,10-dioxo-1,3,4,5,6,7,8,10-octahydrobenzo[*j*][1]oxa[8]thia[5]azacyclododecin-14-yl)-2-fluorobenzoic Acid (**74**)

Compound **74** was synthesized following the procedure described for the synthesis of compound **59** using **109** (20 mg, 32 μmol, 1.0 equiv), XPhos Pd G3 (4 mg, 5 μmol, 15 mol %), K_3_PO_4_ (27 mg, 0.13 mmol, 4.0 equiv), and 4-borono-2-fluorobenzoic acid (17 mg, 96 μmol, 3.0 equiv). The crude product was purified using reverse-phase HPLC with a gradient 15–75% MeCN (containing 0.1% TFA)/water to give **74** (7 mg, 11 μmol, 36% yield) as a colorless powder. HRMS (ESI) *m*/*z*: calcd for C_30_H_34_FN_4_O_8_S [M + H]^+^, 629.2081; found, 629.2090. ^1^H NMR (601 MHz, CDCl_3_): δ 8.12 (d, *J* = 8.5 Hz, 1H), 7.97–7.91 (m, 2H), 7.69 (d, *J* = 9.2 Hz, 1H), 7.39 (t, *J* = 7.7 Hz, 1H), 7.34–7.29 (m, 2H), 7.27–7.23 (m, 1H), 5.41 (dd, *J* = 11.3, 2.6 Hz, 1H), 5.17 (ddd, *J* = 9.5, 9.2, 4.5 Hz, 1H), 4.90 (dt, *J* = 8.5, 2.6 Hz, 1H), 4.55 (dd, *J* = 8.1, 3.5 Hz, 1H), 4.42–4.34 (m, 2H), 3.77 (d, *J* = 10.1 Hz, 1H), 3.76–3.73 (m, 1H), 3.54–3.47 (m 1H), 3.06 (s, 3H), 2.99 (dd, *J* = 14.6, 4.5 Hz, 1H), 2.93 (s, 3H), 2.80 (dd, *J* = 14.6, 9.5 Hz, 1H), 2.35–2.29 (m, 1H), 2.28–2.19 (m, 1H), 2.14 (s, 3H), 2.12–1.96 (m, 2H). *The COOHproton was not detectable in this spectrum.*^13^C NMR (151 MHz, CDCl_3_): δ 172.5, 171.8, 169.6, 168.8, 168.1, 166.3, 161.6 (d, *J* = 260 Hz), 147.7 (d, *J* = 9 Hz), 141.8, 134.0, 133.6, 132.3, 132.1, 131.6, 127.4, 125.1, 117.9 (d, *J* = 24 Hz), 117.1 (d, *J* = 9 Hz), 66.6, 60.2, 53.7, 49.7, 48.5, 37.2, 36.3, 36.0, 35.2, 28.2, 25.1, 22.3.

#### 4-((4*S*,7*R*)-7-((*S*)-1-Acetylpyrrolidine-2-carboxamido)-4-(dimethylcarbamoyl)-6,10-dioxo-1,3,4,5,6,7,8,10-octahydrobenzo[*j*][1]oxa[8]thia[5]azacyclododecin-14-yl)-2-nitrobenzoic Acid (**75**)

Compound **75** was synthesized following the procedure described for the synthesis of compound **59** using **109** (25 mg, 40 μmol, 1.0 equiv), XPhos Pd G3 (5 mg, 6 μmol, 15 mol %), K_3_PO_4_ (34 mg, 0.16 mmol, 4.0 equiv), and 4-carboxy-3-nitrophenylboronic acid (16 mg, 80 μmol, 2.0 equiv). The crude product was purified using reverse-phase HPLC with a gradient 15–75% MeCN (containing 0.1% TFA)/water to give **75** (7 mg, 11 μmol, 28% yield) as a colorless powder. HRMS (ESI) *m*/*z*: calcd for C_30_H_34_N_5_O_10_S [M + H]^+^, 656.2026; found, 656.2020. ^1^H NMR (500 MHz, CDCl_3_): δ 8.00 (dd, *J* = 7.8, 1.4 Hz, 1H), 7.94 (s, 1H), 7.85 (d, *J* = 8.0 Hz, 1H), 7.82–7.73 (m, 3H), 7.43 (td, *J* = 7.8, 1.4 Hz, 1H), 7.33 (dd, *J* = 7.8, 1.4 Hz, 1H), 5.45 (dd, *J* = 11.3, 2.6 Hz, 1H), 5.21 (ddd, *J* = 10.0, 9.7, 4.3 Hz, 1H), 4.94 (ddd, *J* = 8.0, 2.6, 2.3 Hz, 1H), 4.53 (dd, *J* = 8.1, 3.7 Hz, 1H), 4.42 (d, *J* = 10.5 Hz, 1H), 4.38 (dd, *J* = 11.3, 2.3 Hz, 1H), 3.77–3.72 (m, 1H), 3.70 (d, *J* = 10.5 Hz, 1H), 3.56–3.49 (m, 1H), 3.09 (s, 3H), 3.01 (dd, *J* = 14.8, 4.3 Hz, 1H), 2.96 (s, 3H), 2.82 (dd, *J* = 14.8, 10.0 Hz, 1H), 2.36–2.17 (m, 2H), 2.15 (s, 3H), 2.11–1.96 (m, 2H). *The COOHproton was not detectable in this spectrum.*^13^C NMR (126 MHz, CDCl_3_): δ 172.8, 171.7, 169.8, 168.8, 167.8, 166.3, 148.6, 144.8, 140.8, 134.1, 133.9, 133.1, 132.6, 131.7, 130.1, 127.7, 125.7, 124.3, 66.7, 60.2, 53.5, 49.8, 48.5, 37.4, 36.2, 36.2, 35.4, 28.3, 25.1, 22.2.

#### 4-((4*S*,7*R*)-7-((*S*)-1-Acetylpyrrolidine-2-carboxamido)-4-(dimethylcarbamoyl)-6,10-dioxo-1,3,4,5,6,7,8,10-octahydrobenzo[*j*][1]oxa[8]thia[5]azacyclododecin-14-yl)-2-(trifluoromethyl)benzoic Acid (**76**)

Compound **76** was synthesized following the procedure described for the synthesis of compound **59** using **108** (25 mg, 40 μmol, 1.0 equiv), XPhos Pd G3 (5 mg, 6 μmol, 15 mol %), K_3_PO_4_ (34 mg, 0.16 mmol, 4.0 equiv), and 4-borono-2-(trifluoromethyl)benzoic acid (19 mg, 80 μmol, 2.0 equiv). The crude product was purified using reverse-phase HPLC with a gradient 15–75% MeCN (containing 0.1% TFA)/water to give **76** (8 mg, 12 μmol, 30% yield) as a colorless powder. HRMS (ESI) *m*/*z*: calcd for C_31_H_34_F_3_N_4_O_8_S [M + H]^+^, 679.2049; found, 679.2037. ^1^H NMR (500 MHz, CDCl_3_): δ 7.99–7.94 (m, 2H), 7.92 (s, 1H), 7.88 (d, *J* = 7.9 Hz, 1H), 7.74 (d, *J* = 7.9 Hz, 1H), 7.65 (d, *J* = 9.2 Hz, 1H), 7.43 (t, *J* = 7.7 Hz, 1H), 7.33 (d, *J* = 7.7 Hz, 1H), 5.41 (dd, *J* = 11.4, 2.7 Hz, 1H), 5.16 (td, *J* = 9.5, 4.4 Hz, 1H), 4.91 (ddd, *J* = 9.2, 2.7, 2.3 Hz, 1H), 4.53 (dd, *J* = 7.9, 3.8 Hz, 1H), 4.40 (dd, *J* = 11.4, 2.3 Hz, 1H), 4.28 (d, *J* = 10.4 Hz, 1H), 3.78–3.70 (m, 1H), 3.76 (d, *J* = 10.4 Hz, 1H), 3.54–3.47 (m, 1H), 3.07 (s, 3H), 3.05–2.94 (m, 1H), 2.95 (s, 3H), 2.81 (dd, *J* = 14.6, 9.5 Hz, 1H), 2.40–2.31 (m, 1H), 2.29–2.20 (m, 1H), 2.14 (s, 3H), 2.07–1.98 (m, 2H). *The COOHproton was not detectable in this spectrum.*^13^C NMR (126 MHz, CDCl_3_): δ 172.6, 171.6, 169.6, 168.8, 168.1, 168.0, 144.0, 141.7, 134.1, 134.0, 132.4, 132.2, 131.7, 130.6, 129.7, 128.9 (q, *J* = 33 Hz, 1H), 127.7 (q, *J* = 5 Hz, 1H), 127.5, 123.1 (q, *J* = 274 Hz, 1H), 66.6, 60.2, 53.6, 49.6, 48.4, 37.2, 36.2, 36.0, 35.1, 28.0, 25.2, 22.3.

#### 4-((4*S*,7*R*)-7-((*S*)-1-Acetylpyrrolidine-2-carboxamido)-4-(dimethylcarbamoyl)-6,10-dioxo-1,3,4,5,6,7,8,10-octahydrobenzo[*j*][1]oxa[8]thia[5]azacyclododecin-14-yl)-2,6-dimethylbenzoic Acid (**77**)

Compound **77** was synthesized following the procedure described for the synthesis of compound **59** using **109** (25 mg, 40 μmol, 1.0 equiv), XPhos Pd G3 (5 mg, 6 μmol, 15 mol %), K_3_PO_4_ (34 mg, 0.16 mmol, 4.0 equiv), and 2,6-dimethyl-4-(4,4,5,5-tetramethyl-1,3,2-dioxaborolan-2-yl)benzoic acid (23 mg, 80 μmol, 2.0 equiv). The crude product was purified using reverse-phase HPLC with a gradient 15–75% MeCN (containing 0.1% TFA)/water to give **77** (9 mg, 14 μmol, 35% yield) as a colorless powder. HRMS (ESI) *m*/*z*: calcd for C_32_H_39_N_4_O_8_S [M + H]^+^, 639.2489; found, 639.2491. ^1^H NMR (500 MHz, CDCl_3_): δ 8.04 (d, *J* = 8.4 Hz, 1H), 7.87 (dd, *J* = 7.6, 1.7 Hz, 1H), 7.59 (d, *J* = 9.0 Hz, 1H), 7.36 (t, *J* = 7.6 Hz, 1H), 7.32 (dd, *J* = 7.6, 1.7 Hz, 1H), 7.18–7.13 (m, 2H), 5.37 (dd, *J* = 11.3, 2.6 Hz, 1H), 5.10 (ddd, *J* = 8.9, 8.4, 4.6 Hz, 1H), 4.86 (ddd, *J* = 9.0, 2.6, 2.3 Hz, 1H), 4.52 (dd, *J* = 8.0, 3.9 Hz, 1H), 4.39 (dd, *J* = 11.3, 2.3 Hz, 1H), 4.20 (d, *J* = 10.3 Hz, 1H), 3.90 (d, *J* = 10.3 Hz, 1H), 3.79–3.72 (m, 1H), 3.53–3.45 (m, 1H), 3.06 (s, 3H), 2.97 (dd, *J* = 14.5, 4.6 Hz, 1H), 2.93 (s, 3H), 2.84 (dd, *J* = 14.5, 8.9 Hz, 1H), 2.45 (s, 6H), 2.40–2.32 (m, 1H), 2.28–2.19 (m, 1H), 2.13 (s, 3H), 2.06–1.98 (m, 2H). *The COOHproton was not detectable in this spectrum.*^13^C NMR (126 MHz, CDCl_3_): δ 172.1, 171.9, 171.7, 169.5, 168.7, 168.7, 143.4, 141.8, 135.3, 134.2, 134.0, 132.0, 131.5, 131.2, 128.6, 128.6, 127.1, 127.1, 66.4, 60.2, 53.8, 49.3, 48.3, 37.1, 36.2, 35.9, 34.7, 27.9, 25.2, 22.4, 20.1, 20.1.

#### 4-((4*S*,7*R*)-7-((*S*)-1-Acetylpyrrolidine-2-carboxamido)-4-(dimethylcarbamoyl)-6,10-dioxo-1,3,4,5,6,7,8,10-octahydrobenzo[*j*][1]oxa[8]thia[5]azacyclododecin-14-yl)-2,6-difluorobenzoic Acid (**78**)

Compound **78** was synthesized following the procedure described for the synthesis of compound **59** using **109** (25 mg, 40 μmol, 1.0 equiv), XPhos Pd G3 (5 mg, 6 μmol, 15 mol %), K_3_PO_4_ (34 mg, 0.16 mmol, 4.0 equiv), and 4-borono-2,6-difluorobenzoic acid (16 mg, 80 μmol, 2.0 equiv). The crude product was purified using reverse-phase HPLC with a gradient 15–75% MeCN (containing 0.1% TFA)/water to give **78** (5 mg, 8 μmol, 20% yield) as a colorless powder. HRMS (ESI) *m*/*z*: calcd for C_30_H_33_F_2_N_4_O_8_S [M + H]^+^, 647.1987; found, 647.1981. ^1^H NMR (500 MHz, CDCl_3_): δ 8.02 (d, *J* = 8.5 Hz, 1H), 7.94 (d, *J* = 7.7 Hz, 1H), 7.69 (d, *J* = 9.1 Hz, 1H), 7.39 (t, *J* = 7.7 Hz, 1H), 7.30 (d, *J* = 7.7 Hz, 1H), 7.14–7.09 (m, 2H), 5.42 (dd, *J* = 11.4, 2.6 Hz, 1H), 5.19 (ddd, *J* = 9.4, 9.1, 4.8 Hz, 1H), 4.91 (ddd, *J* = 8.5, 2.6, 2.3 Hz, 1H), 4.58 (dd, *J* = 7.8, 3.5 Hz, 1H), 4.39 (dd, *J* = 11.4, 2.3 Hz, 1H), 4.35 (d, *J* = 10.5 Hz, 1H), 3.80 (d, *J* = 10.5 Hz, 1H), 3.78–3.74 (m, 1H), 3.57–3.50 (m, 1H), 3.09 (s, 3H), 3.02 (dd, *J* = 14.6, 4.8 Hz, 1H), 2.96 (s, 3H), 2.84 (dd, *J* = 14.6, 9.4 Hz, 1H), 2.38–2.31 (m, 1H), 2.31–2.20 (m, 1H), 2.17 (s, 3H), 2.14–1.99 (m, 2H). *The COOHproton was not detectable in this spectrum.*^13^C NMR (126 MHz, CDCl_3_): δ 173.1, 171.7, 169.7, 168.8, 168.0, 163.2, 160.3 (d, *J* = 265 Hz), 160.3 (d, *J* = 250 Hz), 146.0 (t, *J* = 10 Hz), 140.9, 133.9, 133.5, 132.3, 131.7, 127.5, 113.1 (d, *J* = 26 Hz), 113.1 (d, *J* = 21 Hz), 109.7 (t, *J* = 17 Hz), 66.5, 60.2, 53.8, 49.7, 48.5, 37.3, 36.2, 36.1, 34.9, 32.3, 28.1, 25.1, 22.9, 22.2.

## Supporting Information Available

Additional results and procedures, NMR spectra for compounds **2**–**78**, purity reports for compounds **2**–**78**, NMR spectra of compounds **S1**–**S24**, and purity reports for compounds **S1**–**S24** (PDF)
Structures (SMILES codes) and biological properties for compounds **2**–**78** and **S1**–**S24** (CSV)
PDB coordinates for docked complexes of macrocycles **41**–**63** and additional compounds **S6** and **S8**–**S22** into Keap1 (ZIP)

## Accession Codes

Coordinates and structure factors for the complex of Keap1 with compounds **2**, **39**, **60**, **63**, and **64** have been deposited with the PDB with accession codes 7Q5H, 7Q6Q, 7Q6S, 7Q8R, and 7Q96, respectively.

## Abbreviations

BLI: biolayer interferometry
bRo5: beyond rule of 5
Cl_int_: intrinsic clearance
clog *S*: calculated solubility
DBA: direct binding assay
DBAD: di-*tert*-butyl azodicarboxylate
DIPEA: *N*,*N*-di-*iso*-propylethylamine
DNP: Dictionary of Natural Products
EDC: *N*-(3-dimethylaminopropyl)-*N*′-ethylcarbodiimide
ER: efflux ratio
FEP: free energy perturbation
FP: fluorescence polarization
ISA: inhibition in solution assay
HATU: 1-[bis(dimethylamino)methylene]-1*H*-1,2,3-triazolo[4,5-*b*]pyridinium 3-oxide hexafluorophosphate
HOBt: 1-hydroxybenzotriazole
IMHB: intramolecular hydrogen bond
ITC: isothermal titration calorimetry
Keap1: Kelch-like ECH associated protein-1
*m*-CPBA: *meta*-chloroperoxybenzoic acid
NHS: *N*-hydroxysuccinimide
NAMFIS: NMR analysis of molecular flexibility in solution
Neh2: Nrf2–ECH homology 2
Nrf2: nuclear factor, erythroid 2-like 2
NRotB: number of rotatable bonds
*P*_app_ AB + inh: efflux-inhibited permeability across a Caco-2 cell monolayer
Pd G3: third-generation palladium precatalyst
pin: pinacolato
PPI: protein–protein interaction
PRCG: Polak–Ribiere-type conjugate gradient
RCE: redundant conformer elimination
SPR: surface plasmon resonance
TCE: 2,2,2-trichloroethyl
TPSA: topological polar surface area
VT: variable temperature
