# Correction to “Importance of Binding Site Hydration and Flexibility Revealed When Optimizing a Macrocyclic Inhibitor of the Keap1–Nrf2 Protein–Protein Interaction”

**DOI:** 10.1021/acs.jmedchem.3c00037

**Published:** 2023-01-26

**Authors:** Fabio Begnini, Stefan Geschwindner, Patrik Johansson, Lisa Wissler, Richard J. Lewis, Emma Danelius, Andreas Luttens, Pierre Matricon, Jens Carlsson, Stijn Lenders, Beate König, Anna Friedel, Peter Sjö, Stefan Schiesser, Jan Kihlberg

Page 3515. By mistake we forgot to acknowledge the European Synchrotron Radiation Facility for provision of the facilities that allowed us to determine the crystal structures reported in the Article. The complete, corrected Acknowledgments section is presented below.

