# Supplementary material for: Importance of Binding Site Hydration and Flexibility Revealed When Optimizing a Macrocyclic Inhibitor of the Keap1–Nrf2 Protein–Protein Interaction
Source: J Med Chem. 2022 Feb 2;65(4):3473–517. doi: 10.1021/acs.jmedchem.1c01975 (PMC8883477; doi:10.1021/acs.jmedchem.1c01975)
Supplement: Supplementary file 1 — jm1c01975_si_001.pdf [file jm1c01975_si_001.pdf]

Supporting Information for:

Importance of binding site hydration and flexibility revealed when optimizing a macrocyclic inhibitor of the Keap1-Nrf2 protein-protein interaction

Fabio Begnini,[1] Stefan Geschwindner,[2] Patrik Johansson,[2] Lisa Wissler,[2] Richard J. Lewis,[3] Emma Danelius,[1] Andreas Luttens,[4] Pierre Matricon,[4] Jens Carlsson,[4] Stijn Lenders,[1] Beate König,[1] Anna Friedel,[1] Peter Sjö,[5] Stefan Schiesser[3,*] and Jan Kihlberg[1,*]

[1] Department of Chemistry - BMC, Uppsala University, Box 576, 75123 Uppsala, Sweden

[2] Mechanistic and Structural Biology, Discovery Sciences, R&D, AstraZeneca, 43183 Mölndal, Sweden

[3] Department of Medicinal Chemistry, Research and Early Development, Respiratory and Immunology (R&I), BioPharmaceuticals R&D, AstraZeneca, 43183 Mölndal, Sweden

[4] Science for Life Laboratory, Department of Cell and Molecular Biology, Uppsala University 75124 Uppsala, Sweden

[5] Drugs for Neglected Diseases initiative (DNDi), 15 Chemin Camille-Vidart, 1202 Geneva, Switzerland

* Corresponding authors

stefan.schiesser@astrazeneca.com, ORCID 0000-0002-8668-2844

jan.kihlberg@kemi.uu.se, ORCID 0000-0002-4205-6040

Table of Contents

SUPPORTING TABLES 3

SUPPORTING FIGURES 14

SUPPORTING SCHEMES 20

SUPPORTING PROCEDURES 23

SUPPORTING PROCEDURE S1 – MOLECULAR DYNAMICS (MD) SIMULATIONS AND FREE ENERGY CALCULATIONS. 23

SUPPORTING PROCEDURE S2 – SYNTHESIS OF COMPOUNDS **S1-S24**. 24

SUPPORTING PROCEDURE S3 – GRAM SCALE SYNTHESIS OF COMPOUND **107** AND SCALE-UP SYNTHESIS OF COMPOUND **1** 39

SUPPORTING PROCEDURE S4 – GRAM SCALE SYNTHESIS OF COMPOUND **10** 41

SUPPORTING PROCEDURE S5 – GRAM SCALE SYNTHESIS OF COMPOUND **12** 43

SUPPORTING PROCEDURE S6 – SYNTHESIS OF COMPOUND **109** 46

NMR SPECTRA FOR COMPOUNDS **2–78** 50

PURITY REPORTS FOR COMPOUNDS **2–78** 127

NMR SPECTRA FOR COMPOUNDS **S1–S24** 164

PURITY REPORTS FOR COMPOUNDS **S1-S24** 188

SUPPLEMENTARY REFERENCES 199

Supporting Tables

Table S1. Amide proton temperature dependent chemical shifts and temperature coefficients for compounds **1**, **6** and **7**.

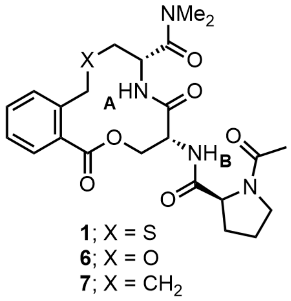

1; X = S
6; X = O
7; X = CH_2

		Compound **1**		Compound **6**		Compound **7**	
T [°C]	T [K]	NH-A	NH-B	NH-A	NH-B	NH-A	NH-B
25	289	8.113	8.941	7.731	8.463	7.846	8.895
35	299	8.068	8.877	7.701	8.429	7.809	8.833
45	309	8.021	8.811	7.668	8.394	7.770	8.778
55	319	7.974	8.746	7.637	8.358	7.735	8.712
65	329	7.927	8.679	7.605	8.326	7.698	8.654
75	339	7.880	8.614	7.577	8.289	7.653	8.596
85	349	7.834	8.546	7.521	8.251	7.615	8.535
	R^2	0.99	0.99	0.99	0.99	0.99	0.99
	$\Delta\delta_{NH}/\Delta T$ [ppb/K]	4.66	6.58	3.50	3.53	3.80	6.00

Table S2. ^{1}H NMR assignment (δ, ppm) of compound **1** in DMSO-d_6 + 20% H_2O (v/v). The structure of compound **1** with the corresponding enumeration used for assignment of the ^{1}H NMR signals is shown.

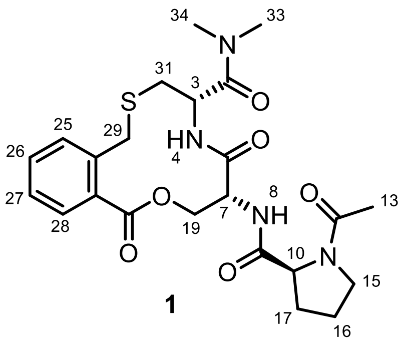

1

Proton No.	δ
8	9.01
4	8.17
28	7.87
26	7.53
25	7.43
27	7.39
3	4.91 – 4.86
19′′	4.87
29′′	4.66
7	4.45 – 4.41
19′	4.31
10	4.31 – 4.28
29′	3.72
15′′,15′	3.51 – 3.47
31′′	2.95 – 2.92
31′	2.90 – 2.87
33-Me, 34-Me	2.84, 2.75
17′′	2.07 – 2.01
16′′	2.00 – 1.95
13-Me	1.92
17′, 16′	1.86 – 1.79

Table S3. Experimentally determined and back-calculated distances (NAMFIS output) interproton distances (Å).

Distances				
Dist. No	Proton a	Proton b	Exp. (Å)	Calc. (Å)
1	25	29′	2.20	2.50
2	29′	4	4.50	3.93
3	29′′	8	2.70	2.65
4	29′′	4	2.40	2.56
5	25	29′′	3.40	3.12
6	29′′	7	3.80	4.56
7	4	8	2.50	2.48
8	7	8	2.60	2.91
9	4	7	3.10	2.98
10	4	33-Me	3.80	3.55
11	7	13-Me	4.60	4.36
12	7	33-Me	4.40	4.39
13	13-Me	33-Me	5.40	5.55
14	13-Me	34-Me	4.20	4.60
15	29′′	31′	3.30	2.99
16	29′′	31′′	3.20	3.25
17	29′	31′	2.70	2.79
18	29′′	31′′	2.70	3.03

RMSD Distances = 0.31

Coupling constants				
^{3}J No.	Proton a	Proton b	Exp. (Hz)	Calc. (Hz)
1	7	8	6.7	7.2
2	4	3	9.8	9.1

RMSD Coupling constants = 0.62

Table S4. RMSD (macrocycle heavy atoms) matrix for the solution conformations of compound **1**.

	Conf. 1	Conf. 2	Conf. 3	Conf. 4	Conf. 5	Conf. 6	X-ray
Conf. 1	0.00	0.69	0.78	0.71	0.78	0.81	0.62
Conf. 2		0.00	0.45	0.22	0.72	0.92	0.37
Conf. 3			0.00	0.42	0.85	1.02	0.55
Conf. 4				0.00	0.67	0.91	0.43
Conf. 5					0.00	0.69	0.70
Conf. 6						0.00	0.92
X-ray							0.00

Table S5. RMSD (all heavy atoms) matrix for the solution conformations of compound **1**.

	Conf 1	Conf 2	Conf 3	Conf 4	Conf 5	Conf 6	X-ray
Conf 1	0	2.74	3.00	2.76	2.92	3.47	2.74
Conf 2		0	1.09	2.53	2.48	2.89	2.57
Conf 3			0	3.16	3.13	3.25	3.18
Conf 4				0	1.27	2.51	1.23
Conf 5					0	2.09	1.11
Conf 6						0	2.22
X-ray							0

Table S6: IC$_{50}$ data for compounds **64**, **77**, and **78** in Eurofins CEREP secondary pharmacology panel.

				**64**	**77**	**78**
Acetylcholinesterase	human	inhibition	IC$_{50}$ (μM)	>100	>100	>100
Adenosine A$_1$ receptor	human	binding to antagonist site	IC$_{50}$ (μM)	>100	>100	>100
Adenosine A$_{2A}$ receptor	human	agonist activity	EC$_{50}$ (μM)	>100	>100	>100
Adenosine transporter	guinea pig	binding	IC$_{50}$ (μM)	>100	>100	>100
Adrenergic α_{1A} receptor	human	binding to antagonist site	IC$_{50}$ (μM)	>100	>100	>100
Adrenergic α_{1B} receptor	human	antagonist activity	IC$_{50}$ (μM)	>100	>100	>100
Adrenergic a$_{2A}$ receptor	human	agonist activity	EC$_{50}$ (μM)	>30	>30	>30
Adrenergic a$_{2A}$ receptor	human	antagonist activity	IC$_{50}$ (μM)	>30	>30	>30
Adrenergic α_{2C} receptor	human	binding to antagonist site	IC$_{50}$ (μM)	>100	>100	>100
Adrenergic b$_1$ receptor	human	agonist activity	EC$_{50}$ (μM)	>100	>100	>100
Adrenergic b$_2$ receptor	human	binding to agonist site	IC$_{50}$ (μM)	>100	>100	>100
ALK4	human	inhibition	IC$_{50}$ (μM)	>100	>100	77
Androgen receptor	human	binding to agonist site	IC$_{50}$ (μM)	>100	>100	>100
Angiotensin II receptor AT1	human	binding to antagonist site	IC$_{50}$ (μM)	>100	>100	>100
Aurora A kinase	human	inhibition	IC$_{50}$ (μM)	>100	>100	>100
Bradykinin receptor 2	human	binding to agonist site	IC$_{50}$ (μM)	>100	>100	>100
Cannabinoid receptor 1	human	agonist activity	EC$_{50}$ (μM)	>100	>100	>100
Cannabinoid receptor 1	human	antagonist activity	IC$_{50}$ (μM)	>100	>100	>100
Cholecystokinin A receptor	human	binding to agonist site	IC$_{50}$ (μM)	>100	>100	>100
c-kit kinase	human	inhibition	IC$_{50}$ (μM)	>100	>100	>100
COX1	human	inhibition	IC$_{50}$ (μM)	>100	>100	>100
COX2	human	inhibition	IC$_{50}$ (μM)	>100	>100	>100
Cathepsin S	human	inhibition	IC$_{50}$ (μM)	>100	>100	>100

Dopamine transporter	human	binding	IC_{50} (μM)	>100	>100	>100
Dopamine receptor D_1	human	binding	IC_{50} (μM)	>100	>100	>100
Dopamine receptor D_{2L}	human	binsing	IC_{50} (μM)	>100	>100	>100
Dopamine receptor D_3	human	agonist activity	EC_{50} (μM)	>100	>100	>100
Dopamine receptor D_3	human	antagonist activity	IC_{50} (μM)	>100	>100	>100
Endothelin receptor A	human	binding to agonist site	IC_{50} (μM)	>100	>100	>100
Epidermal growth factor receptor	human	inhibition	IC_{50} (μM)	>100	>100	>100
Fibroblast growth factor receptor	human	inhibition	IC_{50} (μM)	>100	>100	>100
$GABA_A$ receptor	rat	binding to agonist site	IC_{50} (μM)	>100	>100	>100
Ghrelin receptor	human	binding to agonist site	IC_{50} (μM)	>100	>100	>100
Glucocorticoid receptor	human	binding to agonist site	IC_{50} (μM)	>100	>100	>100
Glycin receptor	rat	binding to agonist site	IC_{50} (μM)	>100	>100	>100
Glycogen synthase kinase 3b	human	inhibition	IC_{50} (μM)	>100	>100	>100
hERG	human	inhibition	IC_{50} (μM)	>40	>40	>40
Histamine receptor H_1	human	binding to antagonist site	IC_{50} (μM)	>100	>100	>100
Histamine receptor H_2	human	agonist activity	IC_{50} (μM)	>100	>100	63
Histamine receptor H_2	human	antagonist activity	EC_{50} (μM)	>100	>100	>100
5-HT_{1A}	human	binding to agonist site	IC_{50} (μM)	>100	>100	>100
5-HT_{1B}	human	binding to antagonist site	IC_{50} (μM)	>100	>100	>100
5-HT_{1D}	rat	binding to agonist site	IC_{50} (μM)	>100	>100	>100
5-HT_{2B}	human	agonist activity	IC_{50} (μM)	>100	>100	>100
5-HT_{2C}	human	agonist activity	EC_{50} (μM)	>100	>100	>100
5-HT_{3A}	human	binding	IC_{50} (μM)	>100	>100	>100
5-HT_4	human	binding to antagonist site	IC_{50} (μM)	>100	>100	>100
5-HT_7	human	agonist activity	IC_{50} (μM)	>100	>100	36
5-HT_7	human	antagonist activity	IC_{50} (μM)	>100	>100	>100

Insulin receptor kinase	human	inhibition	IC_{50} (μM)	>100	>100	>100
Kinase insert domain receptor kinase	human	inhibition	IC_{50} (μM)	>100	>100	>100
L-type calcium channel (Ca_V-L)	rat	binding to diltiazem site	IC_{50} (μM)	>100	>100	>100
L-type calcium channel (Ca_V-L)	rat	binding to verapamil site	IC_{50} (μM)	>100	>100	>100
Lymphocyte-specific protein tyrosine kinase	human	inhibition	IC_{50} (μM)	>100	>100	>100
Matrix metalloproteinase 2	human	inhibition	IC_{50} (μM)	>100	>100	>100
Melatonin receptor 2	human	binding	IC_{50} (μM)	>100	>100	>100
Mitogen activated protein kinase kinase kinase 7	human	inhibition	IC_{50} (μM)	>100	>100	>100
Monoamine oxidase A	human	inhibition	IC_{50} (μM)	>100	>100	>100
Muscarinic acetylcholine receptor 1	human	binding to antagonist site	IC_{50} (μM)	>100	>100	>100
Muscarinic acetylcholine receptor 2	human	binding to antagonist site	IC_{50} (μM)	>100	>100	>100
Muscarinic acetylcholine receptor 5	human	binding to antagonist site	IC_{50} (μM)	>100	>100	>100
Na^+/K^+ ATPase	porcin	inhibition	IC_{50} (μM)	>100	>100	>100
Neurokinin receptor 1	human	binding to agonist site	IC_{50} (μM)	>100	>100	>100
Neurotrophic receptor kinase 1	human	inhibition	IC_{50} (μM)	>100	>100	75
Nicotinic acetylcholine receptor a_1	human	binding	IC_{50} (μM)	>100	>100	>100
Nicotinic acetylcholine receptor a4b2	human	binding	IC_{50} (μM)	>100	>100	>100
Nicotinic acetylcholine receptor a_7	human	binding	IC_{50} (μM)	>100	>100	>100
NMDA receptor	rat	binding to agonist site	IC_{50} (μM)	>100	>100	>100
Norepinephrine transporter (NET)	human	binding	IC_{50} (μM)	>100	>100	>100
d_2 Opioid receptor	human	binding to agonist site	IC_{50} (μM)	>100	>100	>100
μ-Opioid receptor	human	binding to agonist site	IC_{50} (μM)	>100	>100	>100

Opioid receptor k1	human	binding to agonist site	IC_{50} (μM)	>100	>100	>100
Phosphodiesterase 3A	human	inhibition	IC_{50} (μM)	>100	>100	>100
Phosphodiesterase 4D2	human	inhibition	IC_{50} (μM)	>100	>100	>100
Phophodiesterase 6	bovine	inhibition	IC_{50} (μM)	>100	>100	>100
Phosphodiesterase 10A2	human	inhibition	IC_{50} (μM)	>100	>100	>100
PPARg	human	binding to agonist site	IC_{50} (μM)	>100	>100	>100
Pyruvate dehydrogenase kinase 1	human	inhibition	IC_{50} (μM)	>100	>100	70
Retinoic receptor a	human	binding to agonist site	IC_{50} (μM)	>100	>100	>100
Rho associated, coiled coil containing protein kinase 1	human	inhibition	IC_{50} (μM)	>100	>100	>100
Rho associated, coiled coil containing protein kinase 2	human	inhibition	IC_{50} (μM)	>100	>100	>100
Serotonin transporter	human	binding	IC_{50} (μM)	>100	>100	>100
Sigma 1	human	binding to agonist site	IC_{50} (μM)	>100	>100	>100
Somatostatin receptor 4	human	binding to agonist site	IC_{50} (μM)	>100	>100	>100
TSPO	human	binding to antagonist site	IC_{50} (μM)	>100	>100	>100
TXA2 synthase	human	inhibition	IC_{50} (μM)	>100	38	20
Vasopressin receptor 1A	human	binding to agonist site	IC_{50} (μM)	>100	>100	>100
v-src sarcoma kinase	human	inhibition	IC_{50} (μM)	>100	>100	>100

Table S7. Dissociation constants and thermodynamics data determined by ITC for selected compounds. The data was obtained from at least three measurements.

Compound	K_D (M)	SD[a] K_D (M)	ΔG (kcal/mol)	ΔH (kcal/mol)	–TΔS (kcal/mol)
**1**	3.70×10^{-06}	8.00×10^{-08}	–7.55	–14.05	6.50
**2**	1.01 10^{-06}	1.91×10^{-08}	–8.19	–11.45	3.27
**7**	4.13×10^{-06}	4.38×10^{-07}	–7.35	–14.80	7.45
**12**	1.56×10^{-06}	n.d.[b]	–7.93	–12.05	4.14
**38**	9.13×10^{-07}	2.00×10^{-07}	–8.25	–15.90	7.66
**39**	1.44×10^{-06}	1.91×10^{-07}	–7.98	–16.05	8.10
**41**	1.04×10^{-06}	2.12×10^{-08}	–8.17	–14.85	6.69
**42**	2.16×10^{-06}	9.19×10^{-08}	–7.74	–13.65	5.91
**43**	2.57×10^{-06}	2.40×10^{-07}	–7.62	–13.80	6.18
**44**	3.44×10^{-06}	3.89×10^{-07}	–7.28	–12.95	5.68
**46**	4.97×10^{-06}	4.24×10^{-08}	–7.24	–14.20	6.98
**47**	7.75×10^{-07}	8.98×10^{-08}	–8.34	–14.80	6.47
**60**	8.86×10^{-07}	1.34×10^{-07}	–8.27	–9.06	0.80
**63**	8.87×10^{-07}	5.23×10^{-08}	–8.26	–7.54	–0.72
**64**	6.77×10^{-08}	1.75×10^{-08}	–9.80	–11.10	1.30
**67**	4.61×10^{-08}	1.10×10^{-08}	–10.01	–12.20	2.18
**68**	3.83×10^{-07}	1.92×10^{-07}	–8.80	–8.30	–0.51
**72**	4.31×10^{-08}	2.90×10^{-09}	–10.05	–11.65	1.57
**73**	3.64×10^{-08}	1.73×10^{-08}	–10.19	–9.13	–1.06
**74**	3.29×10^{-08}	7.21×10^{-09}	–10.20	–11.30	1.08
**75**	2.88×10^{-08}	9.90×10^{-09}	–10.35	–12.25	1.90
**76**	3.69×10^{-08}	1.59×10^{-08}	–10.20	–9.93	–0.23
**77**	2.92×10^{-08}	1.49×10^{-08}	–10.33	–9.10	–1.23
**78**	2.88×10^{-08}	1.04×10^{-08}	–10.31	–11.50	1.19

[a]SD = Standard deviation; [b]n.d. = not determined

Table S8. Data collection and refinement statistics for the complex between Keap1 and compounds **2**, **39, 60**, **63** and **64**. Values within parenthesis refer to the highest resolution shell. Data completeness reported using a spherical / ellipsoidal resolution cutoff.

Compound	**2**	**39**	**60**	**63**	**64**
Data collection					
Space group	P212121	P212121	P212121	P212121	P212121
Cell dimensions(Å)	75.4 75.5 202.2	75.7 75.9 204.8	75.4 75.6 202.1	75.4 75.4 202.7	75.9 75.8 204.3
Resolution (Å)	2.31-70.8 (2.31-2.54)	2.55-102.4 (2.91-3.06)	2.13-101.1 (2.13-2.37)	2.28-70.7 (2.28-2.53)	2.40-102.2 (2.40-2.65)
R_{merge}	0.08 (1.23)	0.09 (1.09)	0.07 (1.24)	0.06 (1.29)	0.08 (1.33)
<I / σI>	13.0 (1.4)	12.5 (1.5)	14.0 (1.4)	17.8 (1.6)	14.6 (1.5)
Completeness (%)	74.2 (15.2) / 94.5 (55.2)	78.5 (18.3) / 99.9 (99.7)	65.8 (12.4) / 87.4 (62.0)	71.9 (13.8) / 93.8 (65.2)	72.3 (14.3) / 94.0 (61.9)
Redundancy	6.3 (7.0)	6.5 (6.9)	6.7 (7.1)	6.0 (7.4)	6.6 (7.1)
CC(1/2)	1.00 (0.54)	1.00 (0.65)	1.00 (0.55)	1.00 (0.52)	1.00 (0.50)
Refinement					
Resolution (Å)	2.31-70.8	2.55-102.4	2.14-101.1	2.28-70.7	2.42-102.2
Number of reflections	39248	30712	42464	38468	34145
R_{work} / R_{free}	0.191 / 0.226	0.180 / 0.210	0.212 / 0.232	0.205 / 0.229	0.196 / 0.217
No. atoms					
Protein	4372	4372	4372	4372	4372
Waters	271	153	186	198	91
Ligand	36	43	44	44	44
Average *B*-factors					
Protein (Å^2)	63.0	65.6	66.1	68.2	77.5
Waters (Å^2)	62.9	60.7	65.5	64.7	68.6
Ligand (Å^2)	61.8	87.8	82.8	79.4	88.3

R.m.s deviations					
Bond lengths (Å)	0.010	0.010	0.008	0.008	0.008
Bond angles (°)	1.16	1.17	1.00	1.01	0.98

Supporting Figures

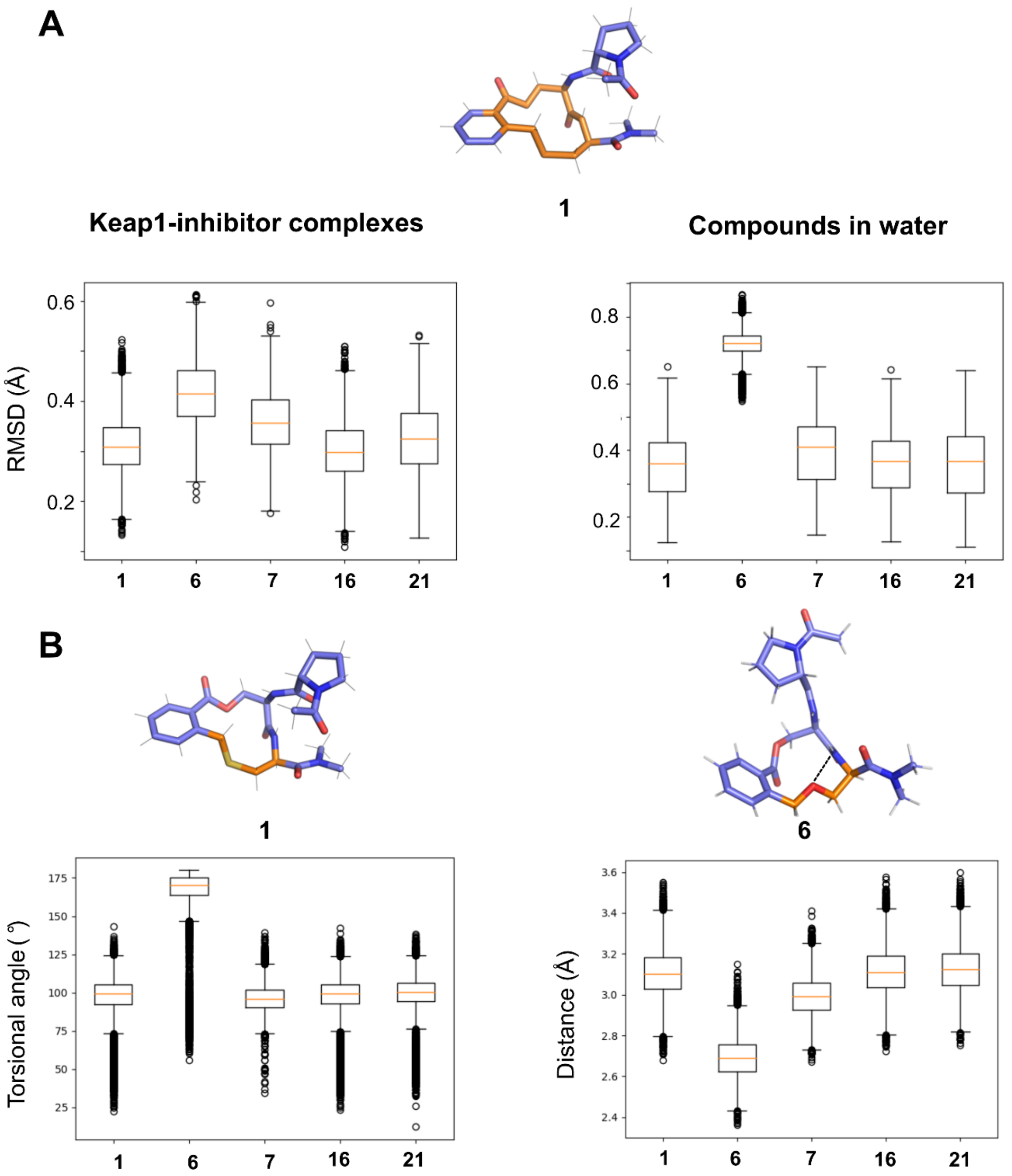

Figure S1. (**A**) Conformation of the Keap1 bound conformation of compound **1** with ring atoms colored in orange and plots of the distribution of RMSD values for corresponding ring atoms in compounds **1**, **6**, **7**, **16** and **21** in MD snapshots with respect to this conformation. (**B**) *Left panel:* Distribution of absolute torsional angle values from MD simulations of compounds **1**, **6**, **7**, **16** and **21** in water, for the torsional angle indicated by the heavy atoms colored in orange. *Right panel:* Distribution for the distance highlighted with a dashed line for compounds **1**, **6**, **7**, **16** and **21** in water.

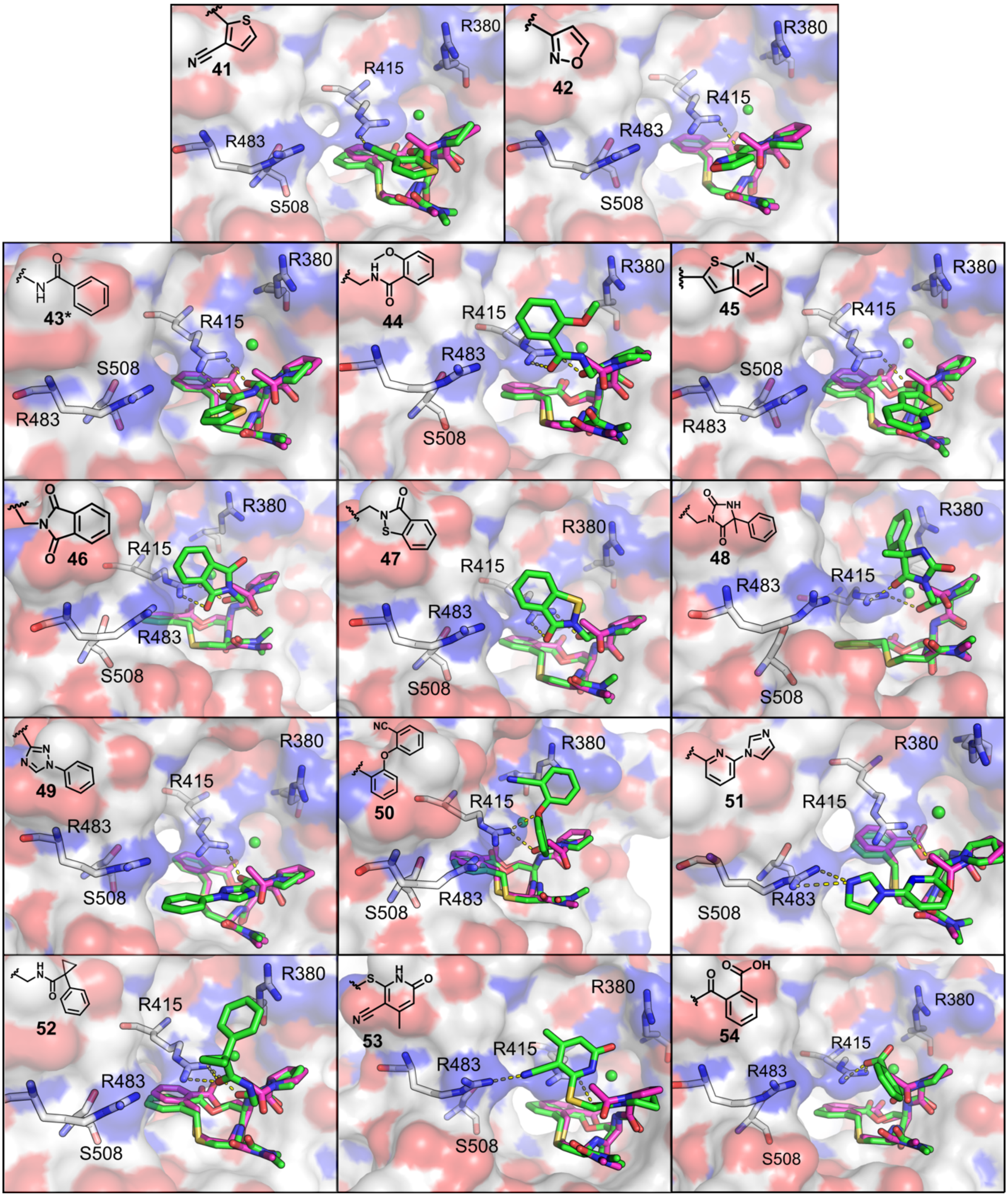

Figure S2. Overlay of docked poses for compounds **41**–**54** and the target-bound crystal structure of compound **1** (magenta) from PDB ID:6Z6A. Keap1 is shown as a white surface with oxygen atoms in red and nitrogen atoms in blue in the inserted figures; selected residues in Keap1 are shown as white sticks with oxygen atoms in red and nitrogen atoms in blue; a chloride ion is shown in green; polar contacts are shown as yellow dashed lines. *Compound **43** was docked as a thiophene, but synthesized as phenyl derivative due to commercial availability of the starting material.

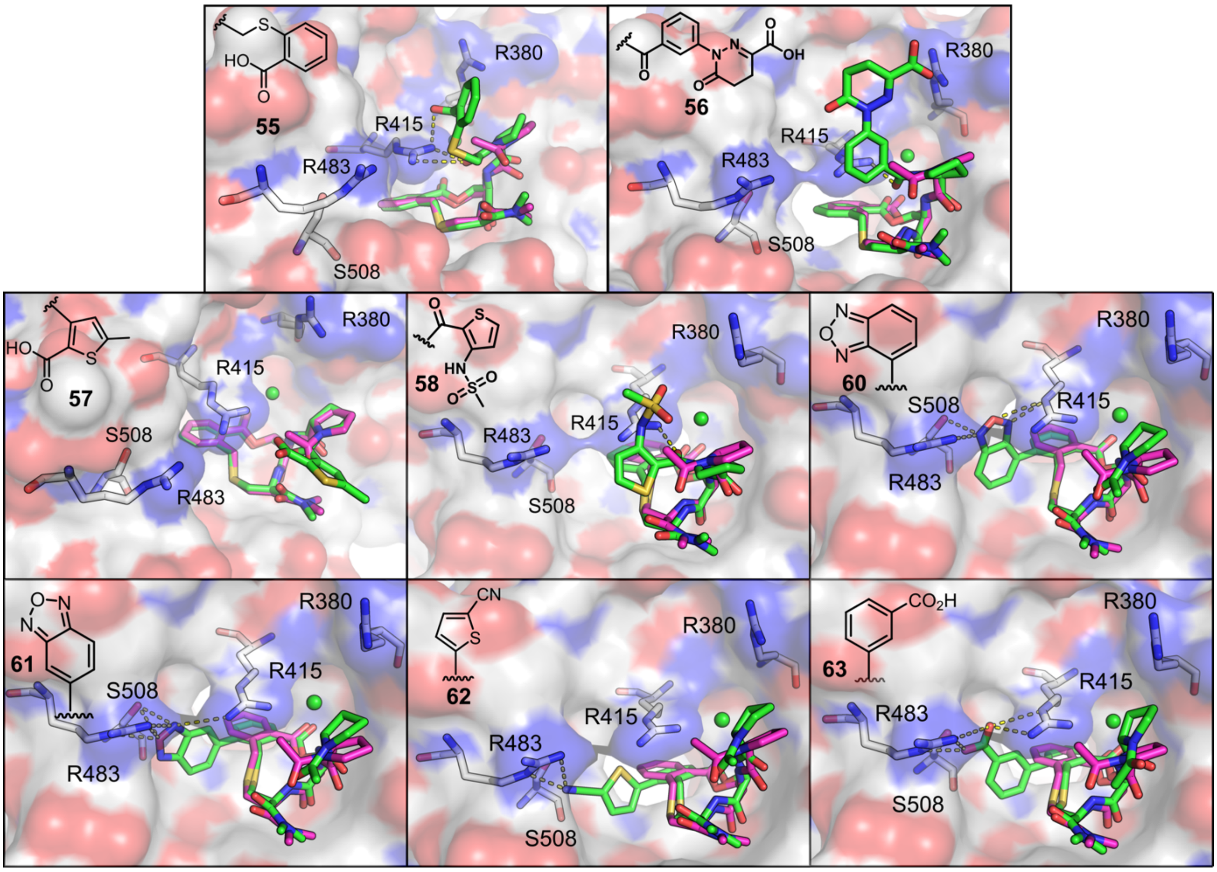

Figure S3. Overlay of docked poses for compounds **55**–**58** and **60**–**63** (green) and the target-bound crystal structure of compound **1** (magenta) from PDB ID:6Z6A. Keap1 is shown as a white surface with oxygen atoms in red and nitrogen atoms in blue in the inserted figures; selected residues in Keap1 are shown as white sticks; a chloride ion is shown in green; polar contacts (as detected by PyMol) are shown as yellow dashed lines.

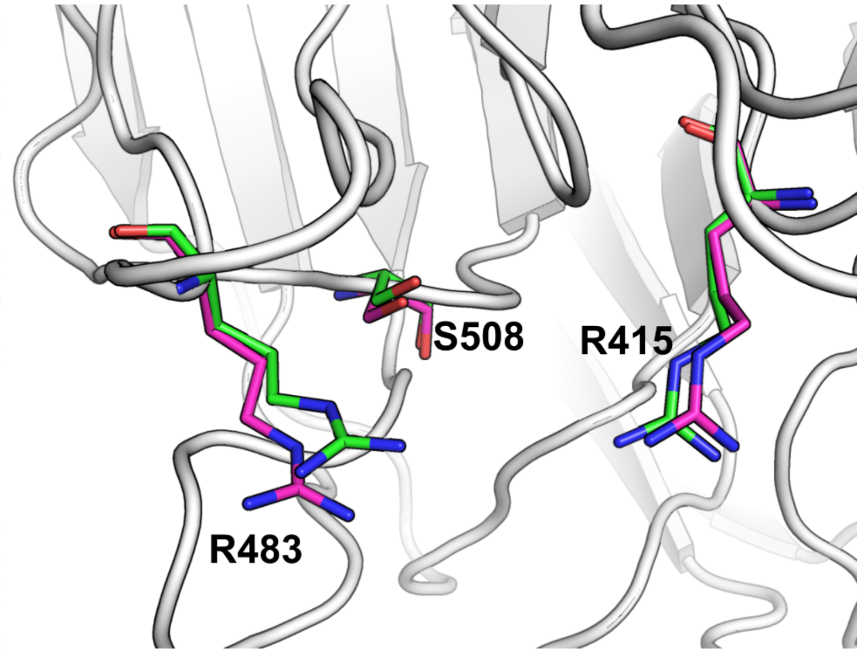

Figure S4. Alternative close-up view showing the orientations of residues R415, R483 and S508 of Keap1 in the complexes with compounds **63** and **64**. The ligands have been removed for clarity, but the side chains of the three residues are colored as for each ligand, i.e. in green (**63**) and magenta (**64**). Keap1 (from PDB ID: 6Z6A) is showed as a grey cartoon.

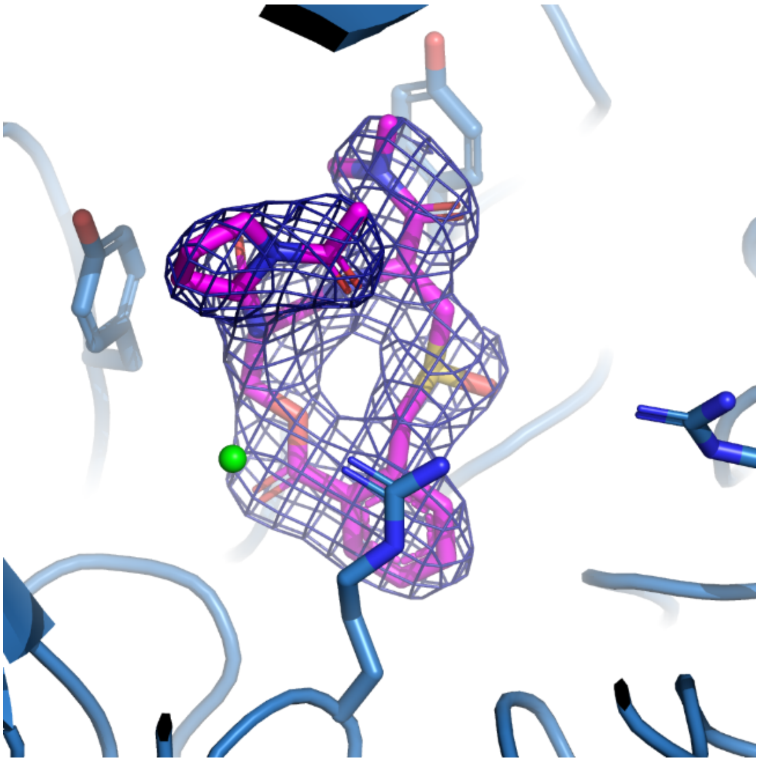

Figure S5. 2Fo-Fc electron density of compound **2** contoured at 1.0σ in the crystalline complex with Keap1.

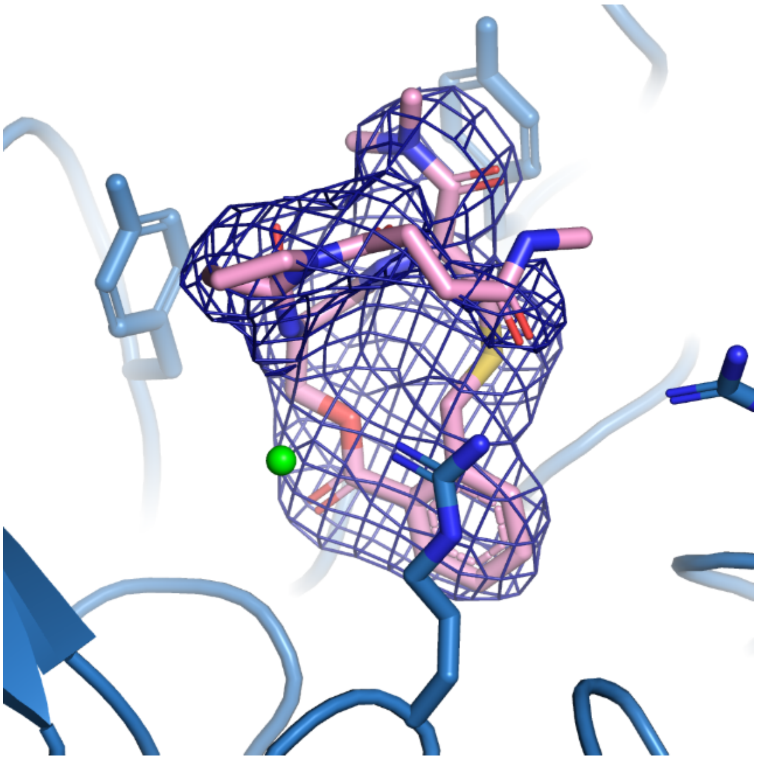

Figure S6. 2Fo-Fc electron density of compound **39** contoured at 1.0σ in the crystalline complex with Keap1.

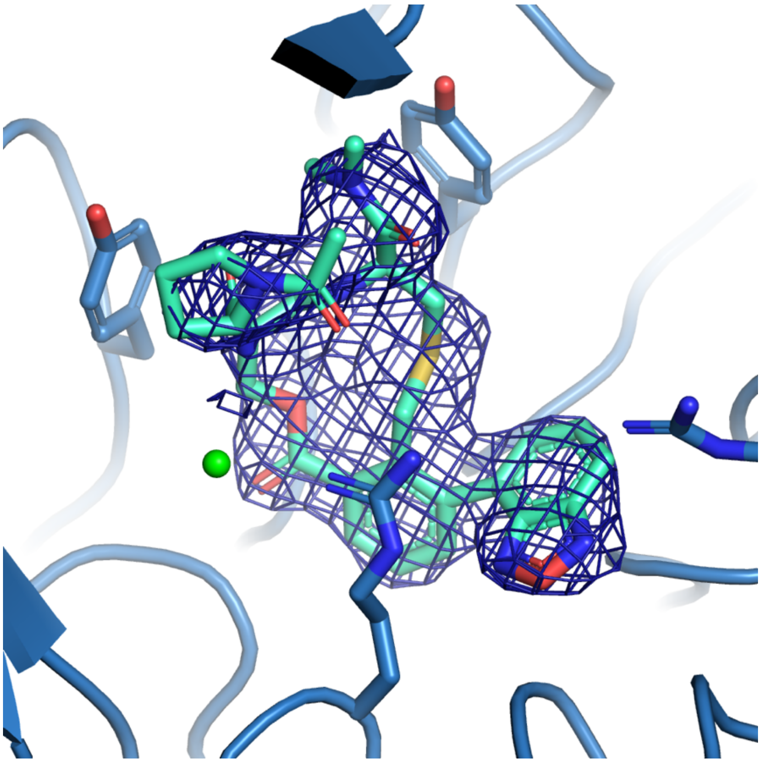

Figure S7. 2Fo-Fc electron density of compound **60** contoured at 1.0σ in the crystalline complex with Keap1.

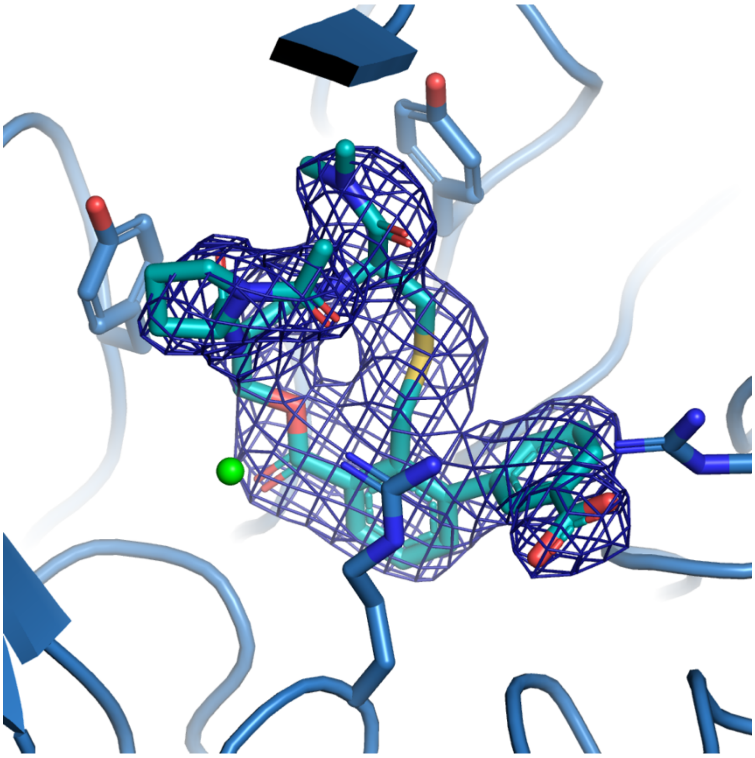

Figure S8. 2Fo-Fc electron density of compound **63** contoured at 1.0σ in the crystalline complex with Keap1.

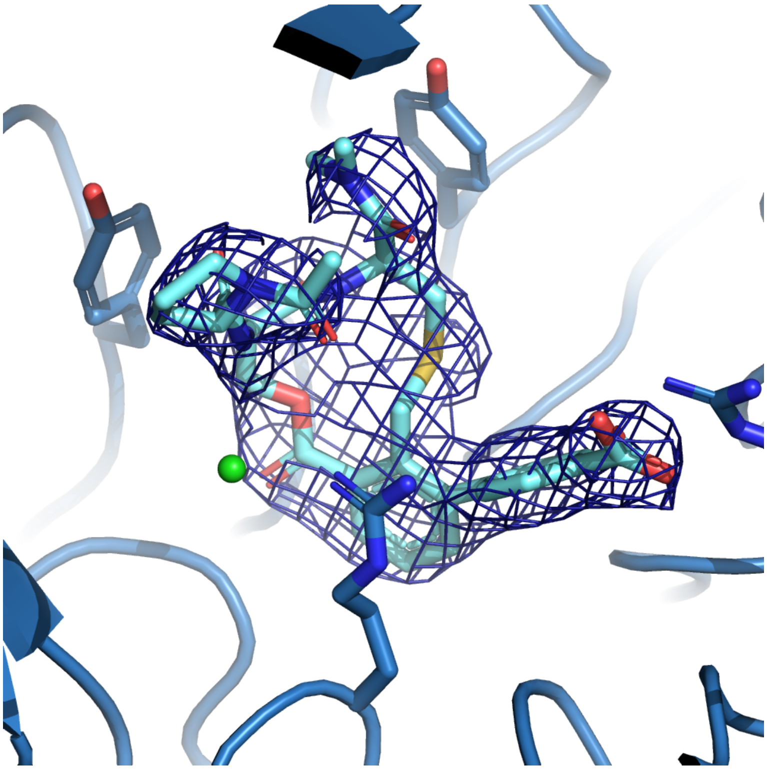

Figure S9. 2Fo-Fc electron density of compound **64** contoured at 1.0σ in the crystalline complex with Keap1.

Supporting Schemes

Scheme S1. Synthesis of and binding activity of compounds **S1–S24.**[a,b]

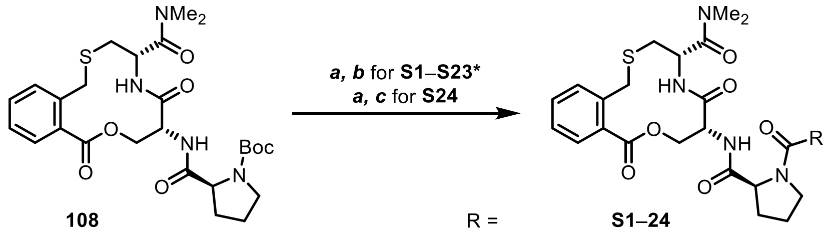

108 R = **S1–24**

*yields calculated over 2 steps

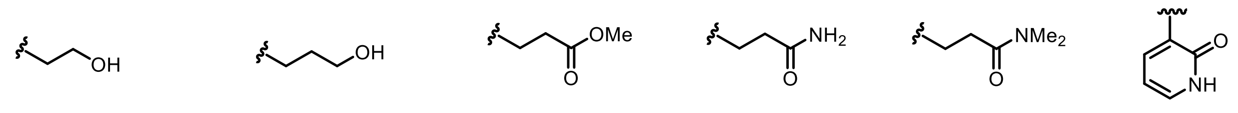

**S1**	**S2**	**S3**	**S4**	**S5**	**S6**
Yield = (24%)	(39%)	(26%)	(28%)	(22%)	(23%)
K_D = 14 ± 1 μM	18 ± 1 μM	17 ± 1 μM	16 ± 3 μM	31 ± 7 μM	11 ± 1 μM

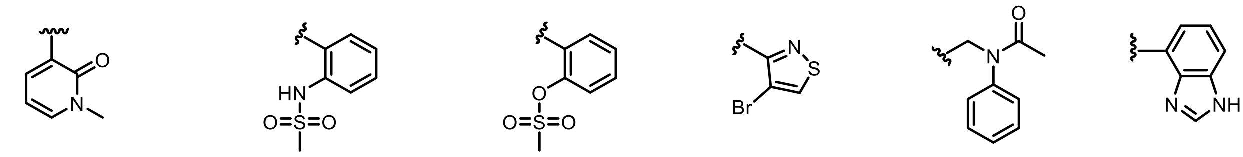

**S7**	**S8**	**S9**	**S10**	**S11**	**S12**
(25%)	(31%)	(20%)	(31%)	(35%)	(55%)
15 ± 2 μM	15 ± 1 μM	14 ± 1 μM	29 ± 1 μM	64 ± 3 μM	13 ± 1 μM

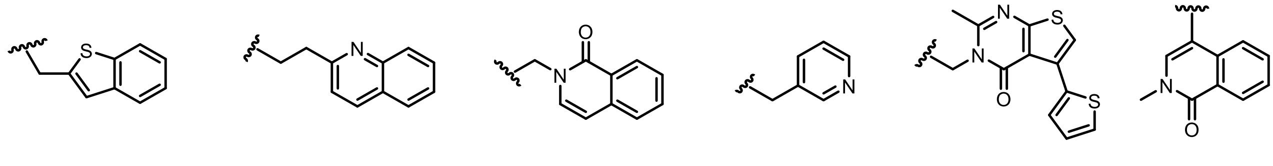

**S13**	**S14**	**S15**	**S16**	**S17**	**S18**
(56%)	(41%)	(69%)	(45%)	(45%)	(56%)
35 ± 1 μM	13 ± 1 μM	51 ± 3 μM	51 ± 2 μM	122 ± 17 μM	46 ± 5 μM

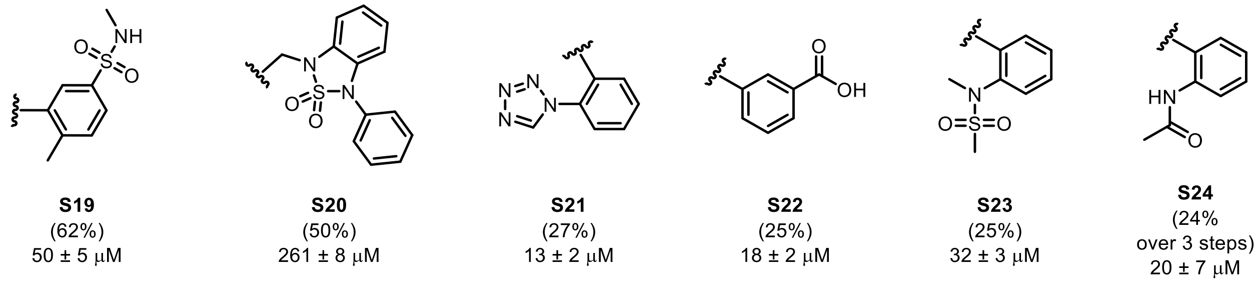

**S19**	**S20**	**S21**	**S22**	**S23**	**S24**
(62%)	(50%)	(27%)	(25%)	(25%)	(24% over 3 steps)
50 ± 5 μM	261 ± 8 μM	13 ± 2 μM	18 ± 2 μM	32 ± 3 μM	20 ± 7 μM

[a]Reagents and conditions: (a) 4 M HCl in 1,4–dioxane, rt, 1 h. (b) R–OH, HATU or EDC·HCl, DIPEA, DMSO, rt, 2 h. (c) anthranilic acid, HATU, DIPEA, DMSO, rt, 2 h, *then* Ac–Cl, triethylamine, DCM, rt, 2h. [b]Dissociation constants (K_D) obtained from surface plasmon resonance (SPR) using an inhibition in solution assay (ISA) format are reported as mean values ± standard deviation, derived from a minimum of 3 independent experiments.

Scheme S2. Gram scale synthesis of compound **107** and scale-up synthesis of compound **1**[a]

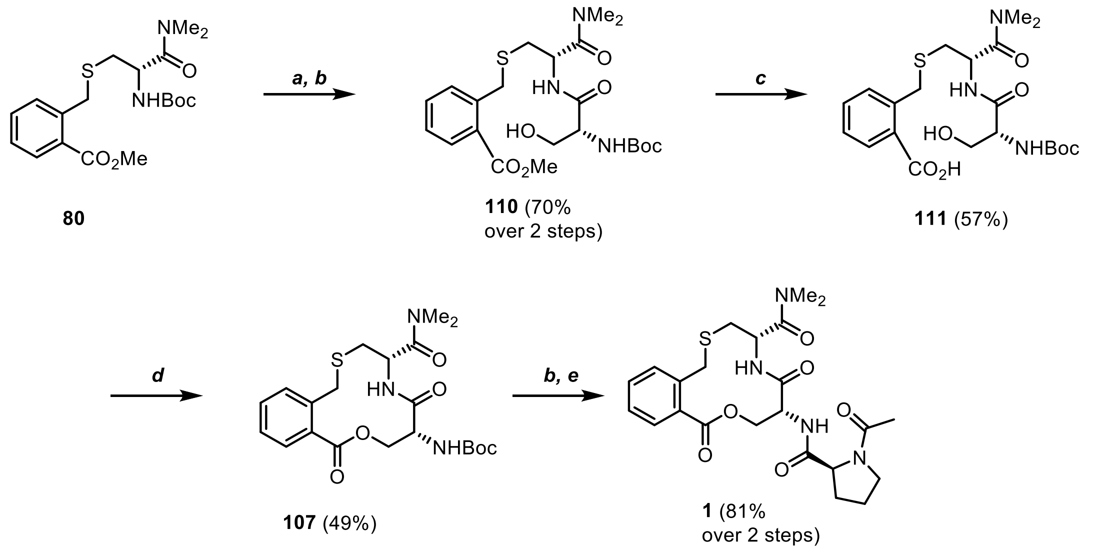

[a]Reagents and conditions: (a) 4 M HCl in 1,4–dioxane, rt, 1 h. (b) Boc-D-Ser-OH, EDC·HCl, MeCN, rt, 2 h. (c) LiOH, MeOH:H_2O 1:1.5, 40 °C, 16 h. (d) $PEtPh_2$, DBAD, toluene, 0 °C to rt, 2 h. (e) Ac-L-Pro-OH, EDC·HCl, DIPEA, MeCN, rt, 2 h.

Scheme S3. Gram scale synthesis of compound **10**[a]

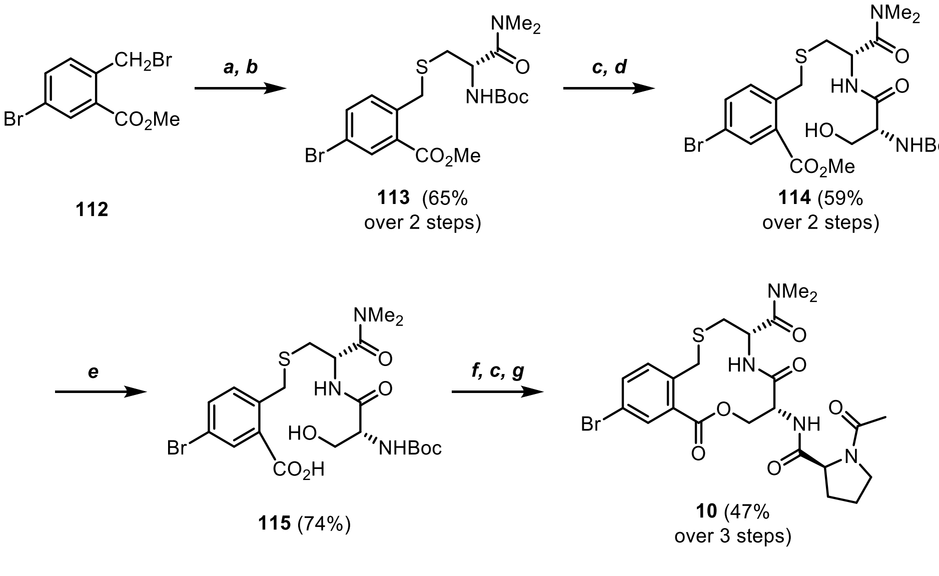

[a]Reagents and conditions: (a) Boc-D-Cys-OH, Et_3N, THF:DMF 3:1, rt, 16 h. (b) Me_2N·HCl, EDC·HCl, HOBt·xH_2O, DIPEA, THF:DMF 3:1, rt, 2 h. (c) 4 M HCl in 1,4–dioxane, rt, 1 h. (d) Boc-D-Ser-OH, EDC·HCl, MeCN, rt, 2 h. (e) LiOH, MeOH:H_2O 1:1.5, 40 °C, 16 h. (f) PPh_3, DBAD, THF, rt, 4 h. (g) Ac-L-Pro-OH, EDC·HCl, DIPEA, MeCN, rt, 2 h.

Scheme S4. Gram scale synthesis of compound **12**[a]

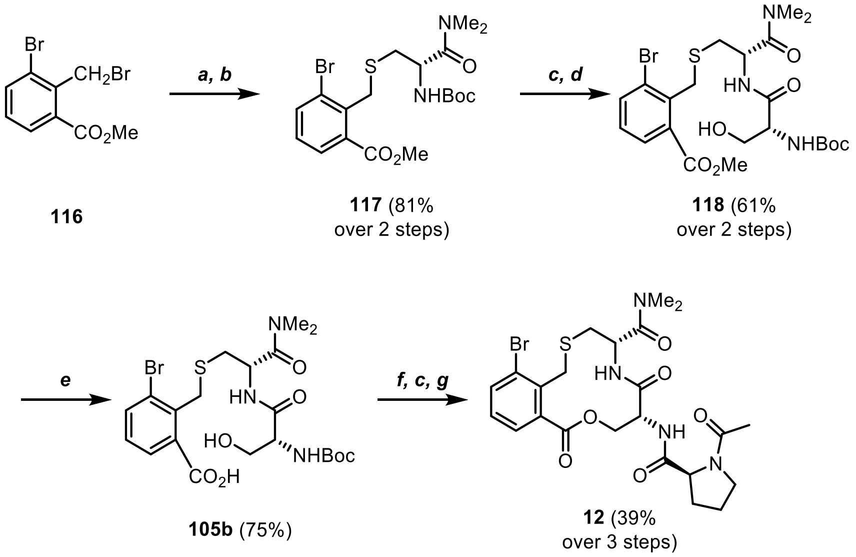

[a]Reagents and conditions: (a) Boc-D-Cys-OH, Et_3N, THF:DMF 3:1, rt, 16 h. (b) Me_2N·HCl, EDC·HCl, HOBt·xH_2O, DIPEA, THF:DMF 3:1, rt, 2 h. (c) 4 M HCl in 1,4–dioxane, rt, 1 h. (d) Boc-D-Ser-OH, EDC·HCl, MeCN, rt, 2 h. (e) LiOH, MeOH:H_2O 1:1.5, 40 °C, 16 h. (f) PPh_3, DBAD, THF, rt, 4 h. (g) Ac-L-Pro-OH, EDC·HCl, DIPEA, MeCN, rt, 2 h.

Scheme S5. Synthesis of compound **109**

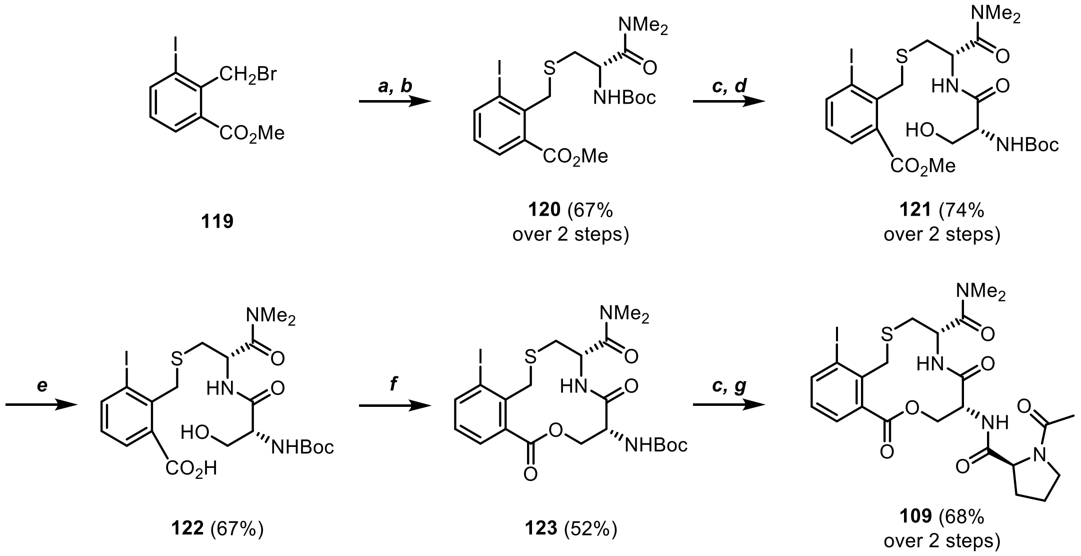

Reagents and conditions: (a) Boc-D-Cys-OH, Et_3N, THF:DMF 4:1, rt, 16 h. (b) Me_2N·HCl, HATU, DIPEA, THF:DMF 4:1, rt, 2 h. (c) 4 M HCl in 1,4–dioxane, rt, 1 h (d) Boc-D-Ser-OH, EDC·HCl, MeCN, rt, 2 h. (e) LiOH, MeOH:H_2O 1:1.5, 40 °C, 16 h. (f) $PEtPh_2$, DBAD, toluene, 0 °C to rt, 2 h. (g) Ac-L-Pro-OH, HATU, DIPEA, MeCN, rt, 2 h.

Supporting Procedures

Supporting Procedure S1 – Molecular dynamics (MD) simulations and free energy calculations.

MD simulations were based on a crystal structure of the Kelch-like ECH-associated protein 1 (Keap1) homodimer bound to compound **1** with the presence of a chloride ion in the ligand binding site (PDB ID: 6Z6A) in both the receptor and aqueous solution. The monomer which was not involved in binding to compound **1** was removed. Other compounds were modelled based on the binding mode of compound **1**. The simulations were carried out with the MD engine Q[1]. The OPLSAA_2005 force field[2] was used to parametrize the compounds and force field parameters were obtained from the program hetgrp_ffgen (Schrödinger, LLC, New York, NY, 2017). Water molecules were represented with the TIP3P model.[3] The simulations were performed under spherical boundary conditions with a sphere radius of 21 Å centered on the compounds. In these conditions, atoms outside the sphere were excluded from non-bonded interactions. Ionizable residues close to the sphere edge were set to their neutral form and atoms within 3 Å of the sphere edge were restrained to their initial coordinates. The surface-constrained all atom solvent (SCAAS)[4] model was used, with radial and polarization restraints applied for solvent molecules at the sphere edge. Solvent bonds and angles were constrained with the SHAKE algorithm[5]. A cutoff of 10 Å was used for non-bonded interactions except for ligand atoms, and electrostatic interactions beyond this cutoff were treated with the local reaction field (LRF) approximation.[6] non-bonded pair lists were updated every 25 steps with a time step of 1 fs for all simulations. Ionizable residues in the binding site were set to their most probable protonation state in aqueous solution at pH 7. His432, His436 and His575 were protonated on the δ position whereas His437 and His552 were protonated on the ε position. Alchemical transformations of a compound into another were divided into four major steps: (i) Partial charges were changed, (ii-iii) for all transformation except the one from **1** to **7**, a soft-core potential was introduced for atoms to annihilate, followed by removal of Lennard-Jones terms for these atoms,[7] and (iv) remaining Lennard-Jones and bonded terms were changed. These transformation steps were further divided into 11, 11, 21 and 41 steps by mapping the potential (U), based on a linear combination of potential energy functions describing the initial (A) and final (B) states of the transformation steps:

$$U = (1 - \lambda)U_A + \lambda U_B \tag{1}$$

where λ is varied from zero to one. Free energy differences were obtained using the Zwanzig equation.[8] At each λ window, receptor-ligand complexes were equilibrated for 750 ps, where the system was heated towards 300 K and harmonic positional restraints on solute atoms were gradually released. Equilibrations were followed by 500 ps of production simulations with energies collected every 50 fs. In addition, simulations of the compounds in aqueous solution were also carried out at the same temperature using a water droplet of the same size. In these simulations, a weak harmonic restraint was applied to a central ligand atom to prevent it from approaching the sphere edge. These systems were equilibrated for 350 ps followed by 100 ps productions. All calculations involved three independent replicates and relative binding free energies were obtained from a thermodynamic cycle, as described in a previous work.[9] Free energy differences were calculated using a bootstrapping strategy where one of the three replicates was randomly selected at each transformation step (1000 times). Free energy results are represented as mean ± SD resulting from the 1000 rounds of bootstrapping. Structural analyses of MD simulation data were based on three extended production runs of 5 ns for each system.

Supporting Procedure S2 – Synthesis of compounds S1-S24.

(4S,7R)-7-((S)-1-(3-Hydroxypropanoyl)pyrrolidine-2-carboxamido)-N,N-dimethyl-6,10-dioxo-1,3,4,5,6,7,8,10-octahydrobenzo[j][1]oxa[8]thia[5]azacyclododecine-4-carboxamide (**S1**).

Compound **108** (25 mg, 46 µmol, 1.0 eq) was dissolved in 4 M HCl in 1,4-dioxane (2 mL) and the mixture was stirred for 1 hour at rt. After evaporation of the volatiles under reduced pressure, the resulting salt was dissolved in DMSO (1 mL). 3-Hydroxypropionic acid (30% wt in water, 25 µL, 92 µmol, 2.0 eq), EDC·HCl (18 mg, 92 µmol, 2.0 eq) and DIPEA (31 µL, 0.18 mmol, 4.0 eq) were added and the mixture was stirred for 16 hours at rt. EtOAc (75 mL) was added and the mixture was washed with 1 M aqueous HCl solution (25 mL), saturated aqueous $NaHCO_3$ solution (25 mL), and brine (25 mL). The organic phase was dried over $MgSO_4$, filtered, and concentrated under reduced pressure. The crude product was purified using reverse phase HPLC with a gradient 20 – 70% MeCN/water to give to give **S1** (6 mg, 11 µmol, 24% yield over 2 steps) as a colorless powder. HRMS (ESI) m/z calcd for $C_{25}H_{33}N_4O_8S$ $[M+H]^+$ 549.2019, found 549.1998. ^1H NMR (500 MHz, $CDCl_3$) δ 7.90 (dd, J = 7.8, 1.4 Hz,

1H), 7.86 (d, J = 9.2 Hz, 1H), 7.65 (d, J = 9.1 Hz, 1H), 7.47 (td, J = 7.5, 1.5 Hz, 1H), 7.40 (dd, J = 7.5, 1.5 Hz, 1H), 7.35 (td, J = 7.8, 1.4 Hz, 1H), 5.23 – 5.14 (m, 2H), 4.94 (dt, J = 9.1, 2.2 Hz, 1H), 4.41 (dd, J = 10.9, 2.2 Hz, 1H), 4.34 (dd, J = 7.1, 3.9 Hz, 1H), 4.12 (d, J = 9.7 Hz, 1H), 3.99 (d, J = 9.7 Hz, 1H), 3.94 – 3.86 (m, 1H), 3.77 – 3.66 (m, 2H), 3.52 – 3.43 (m, 1H), 3.20 – 3.12 (m, 1H), 3.14 (s, 3H), 3.06 (dd, J = 14.6, 4.7 Hz, 1H), 2.93 (s, 3H), 2.72 – 2.63 (m, 1H), 2.49 – 2.39 (m, 1H), 2.35 – 2.27 (m, 1H), 2.24 – 2.16 (m, 1H), 2.01 – 1.94 (m, 2H). *The $CH_2O\underline{H}$ proton was not detectable in this spectrum.* ^{13}C NMR (126 MHz, $CDCl_3$) δ 172.9, 171.5, 169.8, 169.3, 167.8, 136.7, 132.5, 132.4, 131.7, 130.1, 127.7, 66.8, 60.6, 57.5, 53.0, 49.3, 47.7, 37.2, 37.1, 37.1, 36.1, 35.7, 28.1, 25.2.

(4S,7R)-7-((S)-1-(4-Hydroxybutanoyl)pyrrolidine-2-carboxamido)-N,N-dimethyl-6,10-dioxo-1,3,4,5,6,7,8,10-octahydrobenzo[j][1]oxa[8]thia[5]azacyclododecine-4-carboxamide (**S2**).
Compound **S2** was synthesized following the procedure described for the synthesis of compound **S1** using compound **108** (30 mg, 55 μmol, 1.0 eq), 4-hydroxybutanoic acid (20 μL, 0.22 mmol, 4.0 eq), EDC·HCl (21 mg, 0.11 mmol, 2.0 eq), and DIPEA (50 μL, 0.28 mmol, 5.0 eq). The crude product was purified using reverse phase HPLC with a gradient 15 – 65% MeCN/water to give **S2** (9 mg, 17 μmol, 31% over 2 steps) as a colorless powder. HRMS (ESI) m/z calcd for $C_{25}H_{35}N_4O_7S$ $[M+H]^+$ 535.2226, found 535.2216. ^{1}H NMR (500 MHz, $CDCl_3$) δ 7.95 (d, J = 7.8 Hz, 1H), 7.90 (d, J = 8.8 Hz, 1H), 7.78 (d, J = 9.1 Hz, 1H), 7.49 (t, J = 7.5 Hz, 1H), 7.41 (d, J = 7.5 Hz, 1H), 7.37 (t, J = 7.8 Hz, 1H), 5.29 – 5.12 (m, 2H), 4.93 (dt, J = 9.1, 2.3 Hz, 1H), 4.48 – 4.42 (m, 2H), 4.27 (d, J = 9.8 Hz, 1H), 4.00 (d, J = 9.8 Hz, 1H), 3.77 – 3.69 (m, 1H), 3.68 – 3.62 (m, 1H), 3.55 – 3.47 (m, 2H), 3.14 (s, 3H), 3.13 – 3.04 (m, 2H), 2.95 (s, 3H), 2.66 – 2.56 (m, 1H), 2.41 – 2.33 (m, 2H), 2.25 – 2.15 (m, 1H), 2.02 – 1.91 (m, 3H), 1.79 – 1.73 (m, 1H). *The $CH_2O\underline{H}$ proton was not detectable in this spectrum.* ^{13}C NMR (126 MHz, $CDCl_3$) δ 174.3, 171.7, 169.8, 169.2, 167.7, 137.0, 132.5, 132.2, 131.8, 130.0, 127.7, 66.8, 61.8, 60.5, 53.2, 49.7, 47.7, 37.8, 37.3, 36.1, 35.8, 30.9, 27.7, 27.1, 25.2.

Methyl-4-((S)-2-(((4S,7R)-4-(dimethylcarbamoyl)-6,10-dioxo-1,3,4,5,6,7,8,10-octahydrobenzo[j][1]oxa[8]thia[5]azacyclododecin-7-yl)carbamoyl)pyrrolidin-1-yl)-4-oxobutanoate (**S3**).
Compound **S3** was synthesized following the procedure described for the synthesis of compound **S1** using compound **108** (30 mg, 55 μmol, 1.0 eq), monomethyl succinate (14 mg, 0.11 mmol, 2.0 eq), HATU (42 mg, 0.11 mmol, 2.0 eq), and DIPEA (38 μL, 0.22 mmol, 4.0 eq).

The crude product was purified using reverse phase HPLC with a gradient 15 – 75% MeCN/water to give **S3** (8 mg, 14 μmol, 26% over 2 steps) as a colorless powder. HRMS (ESI) m/z calcd for $C_{26}H_{35}N_4O_8S$ $[M+H]^+$ 563.2176, found 563.2184. ^{1}H NMR (500 MHz, $CDCl_3$) δ 7.94 (dd, J = 7.8, 1.5 Hz, 1H), 7.88 (d, J = 8.8 Hz, 1H), 7.61 (d, J = 9.0 Hz, 1H), 7.46 (td, J = 7.5, 1.5 Hz, 1H), 7.37 (dd, J = 7.5, 1.5 Hz, 1H), 7.34 (td, J = 7.8, 1.5 Hz, 1H), 5.17 – 5.11 (m, 2H), 4.81 (td, J = 8.8, 2.4 Hz, 1H), 4.46 (dd, J = 7.9, 3.2 Hz, 1H), 4.43 (dd, J = 11.2, 2.4 Hz, 1H), 4.36 (d, J = 10.0 Hz, 1H), 3.93 (d, J = 10.0 Hz, 1H), 3.74 – 3.68 (m, 1H), 3.66 (s, 3H), 3.55 – 3.49 (m, 1H), 3.08 (s, 3H), 3.06 – 3.03 (m, 2H), 2.90 (s, 3H), 2.80 – 2.68 (m, 3H), 2.51 – 2.43 (m, 1H), 2.38 – 2.32 (m, 1H), 2.26 – 2.18 (m, 1H), 2.03 – 1.89 (m, 3H). ^{13}C NMR (126 MHz, $CDCl_3$) δ 174.0, 173.0, 171.6, 169.6, 168.9, 167.8, 137.4, 132.7, 132.3, 132.1, 129.9, 127.8, 66.7, 60.4, 53.4, 51.9, 49.7, 47.6, 38.1, 37.2, 36.0, 35.9, 29.6, 28.6, 27.7, 25.4.

(4S,7R)-7-((S)-1-(4-Amino-4-oxobutanoyl)pyrrolidine-2-carboxamido)-N,N-dimethyl-6,10-dioxo-1,3,4,5,6,7,8,10-octahydrobenzo[j][1]oxa[8]thia[5]azacyclododecine-4-carboxamide (**S4**).

Compound **S4** was synthesized following the procedure described for the synthesis of compound **S1** using compound **108** (40 mg, 73 μmol, 1.0 eq), 4-amino-4-oxobutanoic (17 mg, 0.15 mmol, 2.0 eq), EDC·HCl (29 mg, 0.15 mmol, 2.0 eq), and DIPEA (50 μL, 0.29 mmol, 4.0 eq). The crude product was purified using reverse phase HPLC with a gradient 25 – 75% MeCN/water to give **S4** (11 mg, 20 μmol, 28% over 2 steps) as a colorless powder. HRMS (ESI) m/z calcd for $C_{25}H_{34}N_5O_7S$ $[M+H]^+$ 548.2179, found 548.2170. ^{1}H NMR (500 MHz, $CDCl_3$) δ 8.15 (d, J = 9.6 Hz, 1H), 7.88 (dd, J = 7.8, 1.4 Hz, 1H), 7.53 (br s, 1H), 7.48 (td, J = 7.6, 1.5 Hz, 1H), 7.40 (dd, J = 7.6, 1.5 Hz, 1H), 7.35 (td, J = 7.8, 1.4 Hz, 1H), 7.25 – 7.20 (m, 1H), 5.57 (s br, 1H), 5.22 (dd, J = 10.8, 2.2 Hz, 1H), 5.14 (ddd, J = 10.5, 9.6, 4.8 Hz, 1H), 4.99 – 4.92 (m, 1H), 4.37 (dd, J = 10.8, 1.8 Hz, 1H), 4.21 – 4.15 (m, 1H), 3.99 (d, J = 9.5 Hz, 1H), 3.94 (d, J = 9.5 Hz, 1H), 3.62 – 3.51 (m, 2H), 3.21 (dd, J = 14.7, 10.5 Hz, 1H), 3.13 (s, 3H), 3.09 (dd, J = 14.7, 4.8 Hz, 1H), 2.95 (s, 3H), 2.87 – 2.79 (m, 1H), 2.58 – 2.51 (m, 1H), 2.48 – 2.41 (m, 1H), 2.34 – 2.28 (m, 1H), 2.24 – 2.13 (m, 2H), 2.10 – 2.04 (m, 1H), 1.97 – 1.89 (m, 1H). ^{13}C NMR (126 MHz, $CDCl_3$) δ 176.0, 172.3, 171.7, 169.8, 169.7, 167.7, 136.6, 132.6, 132.5, 131.6, 130.2, 127.7, 66.6, 61.1, 52.9, 50.0, 47.4, 37.4, 37.2, 36.2, 35.9, 29.9, 29.4, 28.7, 25.3.

(4S,7R)-7-((S)-1-(4-(Dimethylamino)-4-oxobutanoyl)pyrrolidine-2-carboxamido)-N,N-dimethyl-6,10-dioxo-1,3,4,5,6,7,8,10-octahydrobenzo[j][1]oxa[8]thia[5]azacyclododecine-4-carboxamide (**S5**).

Compound **S5** was synthesized following the procedure described for the synthesis of compound **S1** using compound **108** (30 mg, 55 μmol, 1.0 eq), 4-(dimethylamino)-4-oxobutanoic acid (17 mg, 0.11 mmol, 2.0 eq), HATU (42 mg, 0.11 mmol, 2.0 eq), and DIPEA (38 μL, 0.22 mmol, 4.0 eq). The crude product was purified using reverse phase HPLC with a gradient 25 – 75% MeCN/water to give **S5** (7 mg, 12 μmol, 22% over 2 steps) as a colorless powder. HRMS (ESI) m/z calcd for $C_{27}H_{38}N_5O_7S$ $[M+H]^+$ 576.2492, found 576.2490. ^{1}H NMR (500 MHz, $CDCl_3$) δ 7.92 (dd, J = 7.9, 1.5 Hz, 1H), 7.80 (d, J = 9.2 Hz, 1H), 7.72 (d, J = 8.6 Hz, 1H), 7.45 (td, J = 7.7, 1.5 Hz, 1H), 7.37 (dd, J = 7.7, 1.5 Hz, 1H), 7.34 (td, J = 7.9, 1.5 Hz, 1H), 5.18 – 5.13 (m, 1H), 5.14 – 5.09 (m, 1H), 4.83 (dt, J = 8.6, 2.3 Hz, 1H), 4.44 (dd, J = 11.0, 2.3 Hz, 1H), 4.40 (dd, J = 7.7, 4.1 Hz, 1H), 4.29 (d, J = 9.9 Hz, 1H), 3.89 (d, J = 9.9 Hz, 1H), 3.74 – 3.68 (m, 1H), 3.64 – 3.59 (m, 1H), 3.09 – 3.04 (m, 2H), 3.07 (s, 3H), 3.03 (s, 3H), 2.92 (s, 3H), 2.90 (s, 3H), 2.87 – 2.74 (m, 2H), 2.58 – 2.45 (m, 2H), 2.32 – 2.26 (m, 1H), 2.28 – 2.14 (m, 1H), 2.05 – 1.94 (m, 2H). ^{13}C NMR (126 MHz, $CDCl_3$) δ 173.3, 172.2, 172.1, 169.4, 169.0, 167.7, 137.1, 132.5, 132.1, 131.9, 130.0, 127.7, 66.6, 60.5, 53.3, 49.6, 47.5, 38.0, 37.1, 35.9, 35.7, 35.5, 29.5, 27.9, 27.8, 25.2, 22.8.

(4S,7R)-N,N-dimethyl-6,10-dioxo-7-((S)-1-(2-oxo-1,2-dihydropyridine-3-carbonyl)pyrrolidine-2-carboxamido)-1,3,4,5,6,7,8,10-octahydrobenzo[j][1]oxa[8]thia[5]azacyclododecine-4-carboxamide (**S6**).

Compound **S6** was synthesized following the procedure described for the synthesis of compound **S1** using compound **108** (25 mg, 46 μmol, 1.0 eq), 2-oxo-1,2-dihydropyridine-3-carboxylic acid (13 mg, 92 μmol, 2.0 eq), HATU (35 mg, 92 μmol, 2.0 eq), and DIPEA (31 μL, 0.18 mmol, 4.0 eq). The crude product was purified using reverse phase HPLC with a gradient 25 – 75% MeCN/water to give **S6** (6 mg, 11 μmol, 23% over 2 steps) as a colorless powder. HRMS (ESI) m/z calcd for $C_{27}H_{32}N_5O_7S$ $[M+H]^+$ 570.2017, found 570.2002. ^{1}H NMR (600 MHz, DMSO-d_6) δ 11.97 (s, 1H), 8.96 (d, J = 6.8 Hz, 1H), 8.06 (d, J = 9.7 Hz, 1H), 7.92 (dd, J = 7.6, 1.4 Hz, 1H), 7.70 (dd, J = 6.6, 2.2 Hz, 1H), 7.56 (td, J = 7.5, 1.5 Hz, 1H), 7.54 – 7.50 (m, 1H), 7.48 (dd, J = 7.5, 1.5 Hz, 1H), 7.42 (td, J = 7.6, 1.4 Hz, 1H), 6.27 (t, J = 6.6 Hz, 1H), 4.97 (ddd, J = 10.8, 9.7, 4.1 Hz, 1H), 4.88 (dd, J = 11.0, 2.7 Hz, 1H), 4.73 (d, J = 9.5 Hz, 1H), 4.51 – 4.46 (m, 2H), 4.37 (dd, J = 11.0, 2.4 Hz, 1H), 3.79 (d, J = 9.5 Hz, 1H), 3.60 – 3.55 (m, 1H),

3.34 – 3.29 (m, 1H), 2.97 (s, 3H), 2.95 (dd, J = 14.8, 4.1 Hz, 1H), 2.89 (dd, J = 14.8, 10.7 Hz, 1H), 2.79 (s, 3H), 2.11 – 2.04 (m, 1H), 2.04 – 1.98 (m, 1H), 1.95 – 1.90 (m, 1H), 1.83 – 1.77 (m, 1H). ^{13}C NMR (151 MHz, DMSO-d_6) δ 173.1, 169.5, 169.0, 167.2, 166.6, 159.3, 142.0, 138.3, 138.0, 133.2, 133.1, 132.1, 129.9, 128.6, 128.0, 105.2, 66.6, 60.0, 53.6, 51.2, 47.8, 38.3, 36.9, 35.9, 35.4, 29.3, 25.2.

(4S,7R)-N,N-dimethyl-7-((S)-1-(1-methyl-2-oxo-1,2-dihydropyridine-3-carbonyl)pyrrolidine-2-carboxamido)-6,10-dioxo-1,3,4,5,6,7,8,10, octahydrobenzo[j][1]oxa[8]thia[5]azacyclododecine-4-carboxamide (**S7**).

Compound **S7** was synthesized following the procedure described for the synthesis of compound **S1** using compound **108** (30 mg, 55 µmol, 1.0 eq), 1-methyl-2-oxo-1,2-dihydropyridine-3-carboxylic acid (17 mg, 0.11 mmol, 2.0 eq), HATU (42 mg, 0.11 mmol, 2.0 eq), and DIPEA (38 µL, 0.22 mmol, 4.0 eq). The crude product was purified using reverse phase HPLC with a gradient 25 – 75% MeCN/water to give **S7** (8 mg, 14 µmol, 25% over 2 steps) as a colorless powder. HRMS (ESI) m/z calcd for $C_{28}H_{34}N_5O_7S$ $[M+H]^+$ 584.2179, found 584.2171. ^{1}H NMR (500 MHz, $CDCl_3$) δ 8.56 (d, J = 7.3 Hz, 1H), 7.92 (d, J = 7.8 Hz, 1H), 7.64 – 7.56 (m, 2H), 7.41 (t, J = 7.8 Hz, 1H), 7.37 (d, J = 7.8 Hz, 1H), 7.30 (dd, J = 6.8, 2.0 Hz, 1H), 7.26 – 7.20 (m, 1H), 6.25 (t, J = 6.8 Hz, 1H), 5.21 (td, J = 9.2, 4.2 Hz, 1H), 5.03 (dd, J = 11.3, 2.9 Hz, 1H), 4.86 (ddd, J = 7.3, 2.9, 2.1 Hz, 1H), 4.81 (dd, J = 8.5, 3.6 Hz, 1H), 4.59 (d, J = 10.6 Hz, 1H), 4.52 (dd, J = 11.3, 2.1 Hz, 1H), 3.86 (d, J = 10.6 Hz, 1H), 3.64 – 3.57 (m, 1H), 3.43 – 3.38 (m, 1H), 3.17 (s, 3H), 3.08 (s, 3H), 3.04 (dd, J = 14.6, 4.2 Hz, 1H), 2.92 (s, 3H), 2.87 (dd, J = 14.6, 9.2 Hz, 1H), 2.40 – 2.33 (m, 1H), 2.30 – 2.18 (m, 1H), 2.14 –1.92 (m, 2H). ^{13}C NMR (126 MHz, $CDCl_3$) δ 172.5, 169.9, 169.0, 167.4, 166.9, 159.8, 140.0, 139.1, 137.7, 132.1, 132.0, 131.9, 130.3, 128.1, 127.3, 106.2, 66.6, 60.2, 53.9, 49.8, 47.8, 37.9, 37.9, 37.3, 35.9, 35.3, 29.1, 24.7.

(4S,7R)-N,N-Dimethyl-7-((S)-1-(2-(methylsulfonamido)benzoyl)pyrrolidine-2-carboxamido)-6,10-dioxo-1,3,4,5,6,7,8,10-octahydrobenzo[j][1]oxa[8]thia[5]azacyclododecine-4-carboxamide (**S8**).

Compound **S8** was synthesized following the procedure described for the synthesis of compound **S1** using **108** (20 mg, 36 µmol, 1.0 eq), 2-(methylsulfonamido)benzoic acid (15 mg, 72 µmol, 2.0 eq), HATU (27 mg, 72 µmol, 2.0 eq), and DIPEA (24 µL, 0.14 mmol, 4.0 eq). The crude product was purified using reverse phase HPLC with a gradient 15 – 75%

MeCN/water to give **S8** (7 mg, 11 μmol, 31%) as a colorless powder. HRMS (ESI) m/z calcd for $C_{29}H_{35}N_5NaO_8S$ $[M+Na]^+$ 668.1825, found 668.1819. ^1H NMR (500 MHz, $CDCl_3$) δ 9.15 (s, 1H), 8.22 (d, J = 9.2 Hz, 1H), 7.90 (d, J = 7.5 Hz, 1H), 7.63 (d, J = 8.2 Hz, 1H), 7.57 – 7.41 (m, 4H), 7.38 (t, J = 7.5 Hz, 1H), 7.28 (d, J = 8.7 Hz, 1H), 7.14 (t, J = 7.5 Hz, 1H), 5.23 (dd, J = 11.0, 2.6 Hz, 1H), 5.14 (td, J = 9.2, 5.1 Hz, 1H), 4.96 (ddd, J = 8.7, 2.6, 2.1 Hz, 1H), 4.50 (dd, J = 11.0, 2.1 Hz, 1H), 4.44 – 4.39 (m, 1H), 4.15 (d, J = 9.8 Hz, 1H), 3.94 (d, J = 9.8 Hz, 1H), 3.81 – 3.72 (m, 1H), 3.70 – 3.60 (m, 1H), 3.26 (s, 3H), 3.17 – 3.04 (m, 2H), 3.02 (s, 3H), 2.61 (s, 3H), 2.37 – 2.27 (m, 1H), 2.25 – 2.13 (m, 2H), 1.92 – 1.84 (m, 1H). ^{13}C NMR (126 MHz, $CDCl_3$) δ 171.9, 169.1, 169.1, 168.9, 168.1, 137.3, 136.9, 133.9, 132.9, 132.6, 132.1, 131.6, 129.8, 128.4, 127.8, 123.1, 121.0, 66.6, 62.1, 53.3, 51.2, 49.7, 40.0, 37.6, 37.1, 35.8, 35.7, 29.2, 26.0.

2-((S)-2-(((4S,7R)-4-(Dimethylcarbamoyl)-6,10-dioxo-1,3,4,5,6,7,8,10-octahydrobenzo[j][1]oxa[8]thia[5]azacyclododecin-7-yl)carbamoyl)pyrrolidine-1-carbonyl)phenyl methanesulfonate (**S9**).

Compound **S9** was synthesized following the procedure described for the synthesis of compound **S1** using **108** (20 mg, 36 μmol, 1.0 eq), 2-(methanesulfonyloxy)benzoic acid (15 mg, 72 μmol, 2.0 eq), HATU (27 mg, 72 μmol, 2.0 eq), and DIPEA (25 μL, 0.14 mmol, 4.0 eq). The crude product was purified using reverse phase HPLC with a gradient 15 – 80% MeCN/water to give **S9** (5 mg, 8 μmol, 20% over 2 steps) as a colorless powder. HRMS (ESI) m/z calcd for $C_{29}H_{34}N_4NaO_9S_2$ $[M+H]^+$ 669.1665, found 669.1670. ^1H NMR (500 MHz, $CDCl_3$) δ 7.89 (dd, J = 7.8, 1.5 Hz, 1H), 7.77 – 7.71 (m, 2H), 7.64 – 7.57 (m, 1H), 7.51 – 7.43 (m, 3H), 7.38 – 7.29 (m, 3H), 5.18 (dd, J = 11.1, 2.7 Hz, 1H), 5.13 (td, J = 8.2, 4.8 Hz, 1H), 4.92 (dt, J = 8.2, 2.7 Hz, 1H), 4.61 – 4.52 (m, 2H), 4.16 (d, J = 10.1 Hz, 1H), 3.93 (d, J = 10.1 Hz, 1H), 3.63 – 3.58 (m, 1H), 3.38 – 3.31 (m, 1H), 3.16 (s, 3H), 3.06 (s, 3H), 3.06 – 2.99 (m, 2H), 2.81 (s, 3H), 2.41 – 2.34 (m, 1H), 2.25 – 2.17 (m, 1H), 2.15 – 2.07 (m, 1H), 1.93 – 1.86 (m, 1H). ^{13}C NMR (126 MHz, $CDCl_3$) δ 171.6, 169.0, 168.8, 168.2, 167.6, 145.0, 137.2, 132.5, 132.0, 131.7, 131.2, 130.0, 130.0, 129.1, 127.6, 127.4, 123.2, 66.6, 61.1, 53.7, 49.6, 49.5, 38.1, 37.4, 37.0, 35.8, 35.5, 28.4, 25.2.

(4S,7R)-7-((S)-1-(4-bromoisothiazole-3-carbonyl)pyrrolidine-2-carboxamido)-N,N-dimethyl-6,10-dioxo-1,3,4,5,6,7,8,10-octahydrobenzo[j][1]oxa[8]thia[5]azacyclododecine-4-carboxamide (**S10**).

Compound **S9** was synthesized following the procedure described for the synthesis of compound **S1** using **108** (25 mg, 46 μmol, 1.0 eq), 4-bromo-1,2-thiazole-3-carboxylic acid (19 mg, 92 μmol, 2.0 eq), HATU (35 mg, 92 μmol, 2.0 eq) and DIPEA (31 μL, 0.18 mmol, 4.0 eq). The crude product was purified by reverse phase HPLC using a gradient from 20% to 75% MeCN in water to give to give **S10** (9 mg, 14 μmol, 31%) as a colorless powder. HRMS (ESI) m/z calcd for $C_{25}H_{29}BrN_5O_6S$ $[M+H]^+$ 638.0737, found 638.0730. ^1H NMR (500 MHz, $CDCl_3$) δ 8.59 (s, 1H), 7.87 (dd, J = 7.8, 1.4 Hz, 1H), 7.67 (d, J = 8.2 Hz, 1H), 7.60 (d, J = 9.1 Hz, 1H), 7.45 (td, J = 7.5, 1.5 Hz, 1H), 7.36 (dd, J = 7.5, 1.5 Hz, 1H), 7.31 (td, J = 7.8, 1.4 Hz, 1H), 5.18 (dd, J = 11.2, 2.6 Hz, 1H), 5.09 (td, J = 9.1, 5.0 Hz, 1H), 4.91 (ddt, J = 8.2, 2.6, 2.2 Hz, 1H), 4.67 (dd, J = 7.3, 4.8 Hz, 1H), 4.47 (dd, J = 11.2, 2.2 Hz, 1H), 4.26 (d, J = 10.0 Hz, 1H), 3.91 (d, J = 10.0 Hz, 1H), 3.89 – 3.81 (m, 1H), 3.62 – 3.54 (m, 1H), 3.13 – 3.05 (m, 2H), 3.02 (s, 3H), 2.77 (s, 3H), 2.49 – 2.40 (m, 1H), 2.20 – 2.10 (m, 2H), 1.98 – 1.89 (m, 1H). ^{13}C NMR (126 MHz, $CDCl_3$) δ 171.3, 169.0, 168.7, 167.9, 162.8, 160.4, 147.8, 137.1, 132.5, 132.1, 131.8, 130.0, 127.6, 108.5, 66.5, 61.2, 53.7, 49.9, 49.5, 38.1, 37.0, 35.8, 35.7, 28.0, 25.3.

(4S,7R)-7-((S)-1-(N-acetyl-N-phenylglycyl)pyrrolidine-2-carboxamido)-N,N-dimethyl-6,10-dioxo-1,3,4,5,6,7,8,10-octahydrobenzo[j][1]oxa[8]thia[5]azacyclododecine-4-carboxamide (**S11**).

Compound **S11** was prepared following the procedure described for the synthesis of compound **S1** using compound **108** (25 mg, 46 μmol, 1.0 eq), 2-(N-phenylacetamido)acetic acid (18 mg, 92 μmol, 2.0 eq), HATU (35 mg, 92 μmol, 2.0 eq) and DIPEA (31 μL, 0.18 mmol, 4.0 eq). The crude product was purified by reverse phase HPLC using a gradient from 15% to 75% MeCN in water to give **S11** (10 mg, 16 μmol, 35%) as a colorless powder. HRMS (ESI) m/z calcd for $C_{31}H_{38}N_5O_7S$ $[M+H]^+$ 624.2486, found 624.2470. ^1H NMR (600 MHz, $CDCl_3$) δ 7.98 (d, J = 8.4 Hz, 1H), 7.86 (dd, J = 7.8, 1.5 Hz, 1H), 7.45 (td, J = 7.5, 1.5 Hz, 1H), 7.42 – 7.27 (m, 8H), 5.08 (td, J = 8.4, 4.7 Hz, 1H), 5.02 (dd, J = 11.2, 3.2 Hz, 1H), 4.85 (dt, J = 8.2, 2.5 Hz, 1H), 4.61 – 4.54 (m, 1H), 4.58 (d, J = 15.5 Hz, 1H) 4.44 (dd, J = 11.2, 2.0 Hz, 1H), 4.39 (d, J = 15.5 Hz, 1H), 4.32 (d, J = 10.3 Hz, 1H), 3.85 – 3.78 (m, 1H), 3.77 (d, J = 10.3 Hz, 1H), 3.71 – 3.63 (m, 1H), 3.03 (s, 3H), 2.93 (dd, J = 14.8, 4.7 Hz, 1H), 2.86 (s, 3H), 2.72 (dd, J = 14.8, 8.4 Hz, 1H), 2.40 – 2.32 (m, 1H), 2.27 – 2.17 (m, 1H), 2.08 – 1.93 (m, 2H), 1.88 (s, 3H). ^{13}C NMR (151 MHz, $CDCl_3$) δ 171.6, 171.2, 169.6, 169.5, 168.8, 168.1, 143.9, 137.5, 132.6, 132.2, 132.1, 130.2, 129.6, 129.6, 128.1, 128.1, 128.0, 127.7, 66.9, 60.7, 53.7, 52.0, 49.8, 47.3, 37.8, 37.1, 35.9, 35.2, 27.9, 25.3, 22.2.

(4S,7R)-7-((S)-1-(1H-benzo[d]imidazole-4-carbonyl)pyrrolidine-2-carboxamido)-N,N-dimethyl-6,10-dioxo-1,3,4,5,6,7,8,10-octahydrobenzo[j][1]oxa[8]thia[5]azacyclododecine-4-carboxamide (**S12**).

Compound **S12** was prepared following the procedure described for the synthesis of compound **S1** using compound **108** (25 mg, 46 μmol, 1.0 eq), 1H-benzo[d]imidazole-4-carboxylic acid (15 mg, 92 μmol, 2.0 eq), HATU (35 mg, 92 μmol, 2.0 eq) and DIPEA (31 μL, 0.18 mmol, 4.0 eq). The crude product was purified by reverse phase HPLC using a gradient from 15% to 75% MeCN in water to give **S12** (15 mg, 25 μmol, 55%) as a colorless powder. HRMS (ESI) m/z calcd for $C_{29}H_{33}N_6O_6S$ $[M+H]^+$ 593.2177, found 593.2164. ^{1}H NMR (600 MHz, $CDCl_3$) δ 12.00 (s, 1H), 8.23 (d, J = 9.3 Hz, 1H), 8.16 (s, 1H), 7.92 (d, J = 8.0 Hz, 1H), 7.83 (dd, J = 7.9, 1.5 Hz, 1H), 7.57 (d, J = 8.0 Hz, 1H), 7.46 (td, J = 7.5, 1.4 Hz, 1H), 7.40 (dd, J = 7.5, 1.4 Hz, 1H), 7.34 (dd, J = 7.9, 1.5 Hz, 1H), 7.23 (t, J = 8.0 Hz, 1H), 7.15 (d, J = 9.0 Hz, 1H), 5.30 – 5.26 (m, 1H), 5.25 (dd, J = 10.8, 2.4 Hz, 1H), 5.01 (ddd, J = 9.0, 2.4, 2.1 Hz, 1H), 4.45 (dd, J = 10.8, 2.1 Hz, 1H), 4.36 (dd, J = 8.0, 4.3 Hz, 1H), 4.05 (d, J = 9.4 Hz, 1H), 3.99 – 3.90 (m, 2H), 3.75 (d, J = 9.4 Hz, 1H), 3.25 (s, 3H), 3.18 (dd, J = 14.6, 11.1 Hz, 1H), 3.12 (dd, J = 14.6, 4.8 Hz, 1H), 2.98 (s, 3H), 2.37 – 2.30 (m, 1H), 2.25 – 2.15 (m, 2H), 2.00 – 1.94 (m, 1H). ^{13}C NMR (151 MHz, $CDCl_3$) δ 172.3, 170.4, 170.2, 168.0, 168.0, 144.5, 143.2, 136.0, 133.0, 132.5, 132.4, 131.3, 130.6, 127.8, 123.4, 122.6, 120.4, 117.7, 66.9, 62.3, 52.9, 51.3, 48.9, 37.4, 36.2, 35.5, 35.1, 29.3, 25.9.

(4S,7R)-7-((S)-1-(2-(benzo[b]thiophen-2-yl)acetyl)pyrrolidine-2-carboxamido)-N,N-dimethyl-6,10-dioxo-1,3,4,5,6,7,8,10-octahydrobenzo[j][1]oxa[8]thia[5]azacyclododecine-4-carboxamide (**S13**).

Compound **S13** was prepared following the procedure described for the synthesis of compound **S1** using compound **108** (25 mg, 46 μmol, 1.0 eq), 2-(1-benzothiophen-2-yl)acetic acid (19 mg, 92 μmol, 2.0 eq), HATU (35 mg, 92 μmol, 2.0 eq) and DIPEA (31 μL, 0.18 mmol, 4.0 eq). The crude product was purified by reverse phase HPLC using a gradient from 15% to 75% MeCN in water to give **S13** (16 mg, 26 μmol, 56%) as a colorless powder. HRMS (ESI) m/z calcd for $C_{31}H_{35}N_4O_6S_2$ $[M+H]^+$ 623.1993, found 623.1997. ^{1}H NMR (600 MHz, $CDCl_3$) δ 8.12 (d, J = 8.9 Hz, 1H), 7.92 (dd, J = 7.8, 1.5 Hz, 1H), 7.76 (dd, J = 7.8, 1.2 Hz, 1H), 7.69 (dd, J = 7.5, 1.6 Hz, 1H), 7.44 (d, J = 9.1 Hz, 1H), 7.38 – 7.25 (m, 4H), 7.15 (d, J = 1.1 Hz, 1H), 7.03 (dd, J = 7.7, 1.3 Hz, 1H), 5.13 (dd, J = 11.1, 2.5 Hz, 1H), 5.13 – 5.10 (m, 1H), 4.83 (ddd, J = 8.9, 2.5,

2.1 Hz, 1H), 4.58 (dd, J = 8.0, 2.7 Hz, 1H), 4.42 (d, J = 10.0 Hz, 1H), 4.39 (dd, J = 11.1, 2.2 Hz, 1H), 4.23 (dd, J = 16.4, 1.1 Hz, 1H), 4.01 (dd, J = 16.4, 1.1 Hz, 1H), 3.92 – 3.86 (m, 1H), 3.59 – 3.53 (m, 1H), 3.50 (d, J = 10.0 Hz, 1H), 3.05 (s, 3H), 2.97 (dd, J = 14.7, 4.5 Hz, 1H), 2.93 (s, 3H), 2.82 (dd, J = 14.7, 9.5 Hz, 1H), 2.45 – 2.40 (m, 1H), 2.30 – 2.20 (m, 1H), 2.05 – 1.95 (m, 1H), 1.94 – 1.89 (m, 1H). ^{13}C NMR (151 MHz, $CDCl_3$) δ 170.9, 170.9, 169.6, 168.5, 167.7, 140.1, 139.5, 137.3, 137.0, 132.6, 132.3, 132.2, 129.5, 127.5, 124.2, 124.0, 123.4, 123.1, 122.1, 66.7, 60.4, 53.3, 49.8, 47.9, 38.6, 37.1, 36.7, 35.8, 35.7, 27.2, 25.3.

(4S,7R)-N,N-dimethyl-6,10-dioxo-7-((S)-1-(3-(quinolin-2-yl)propanoyl)pyrrolidine-2-carboxamido)-1,3,4,5,6,7,8,10-octahydrobenzo[j][1]oxa[8]thia[5]azacyclododecine-4-carboxamide (**S14**).

Compound **S14** was prepared following the procedure described for the synthesis of compound **S1** using compound **108** (25 mg, 46 µmol, 1.0 eq), 3-(-(quinolin-2-yl)propanoic acid (enamine code EN300-12576, 19 mg, 92 µmol, 2.0 eq), HATU (35 mg, 92 µmol, 2.0 eq) and DIPEA (31 µL, 0.18 mmol, 4.0 eq). The crude product was purified by reverse phase HPLC using a gradient from 15% to 75% MeCN in water to give **S14** (12 mg, 19 µmol, 41%) as a colorless powder. HRMS (ESI) m/z calcd for $C_{33}H_{38}N_5O_6S$ $[M+H]^+$ 632.2537, found 632.2521. ^{1}H NMR (600 MHz, $CDCl_3$) δ 8.03 (d, J = 8.4 Hz, 1H), 7.98 – 7.92 (m, 3H), 7.77 (dd, J = 8.0, 1.5 Hz, 1H), 7.74 (d, J = 9.1 Hz, 1H), 7.66 (ddd, J = 8.5, 6.8, 1.5 Hz, 1H), 7.48 (ddd, J = 8.5, 6.8, 1.2 Hz, 1H), 7.42 (td, J = 7.5, 1.5 Hz, 1H), 7.38 – 7.29 (m, 3H), 5.18 – 5.12 (m, 2H), 4.85 (dt, J = 8.7, 2.4 Hz, 1H), 4.44 – 4.40 (m, 2H), 4.35 (d, J = 9.9 Hz, 1H), 3.94 (d, J = 9.9 Hz, 1H), 3.78 – 3.69 (m, 1H), 3.62 – 3.56 (m, 1H), 3.33 – 3.24 (m, 1H), 3.23 – 3.15 (m, 1H), 3.15 – 3.08 (m, 2H), 3.07 (s, 3H), 3.05 – 3.00 (m, 1H), 2.95 – 2.91 (m, 1H), 2.90 (s, 3H), 2.38 – 2.31 (m, 1H), 2.24 – 2.14 (m, 1H), 2.01 – 1.88 (m, 2H). ^{13}C NMR (151 MHz, $CDCl_3$) δ 173.7, 171.7, 169.4, 168.9, 167.7, 161.6, 147.9, 137.3, 136.0, 132.5, 132.2, 132.0, 129.9, 129.2, 128.7, 127.6, 127.6, 126.9, 125.7, 122.1, 66.6, 60.3, 53.3, 49.7, 47.6, 38.2, 37.1, 36.0, 35.9, 33.3, 33.0, 27.5, 25.3.

(4S,7R)-N,N-dimethyl-6,10-dioxo-7-((S)-1-(2-(1-oxoisoquinolin-2(1H)-yl)acetyl)pyrrolidine-2-carboxamido)-1,3,4,5,6,7,8,10-octahydrobenzo[j][1]oxa[8]thia[5]azacyclododecine-4-carboxamide (**S15**).

Compound **S15** was prepared following the procedure described for the synthesis of compound **S1** using compound **108** (25 mg, 46 µmol, 1.0 eq), 2-(1-oxo-1,2-dihydroisoquinolin-2-yl)acetic acid (enamine code EN300-188561, 19 mg, 92 µmol, 2.0 eq), HATU (35 mg, 92 µmol, 2.0 eq)

and DIPEA (31 µL, 0.18 mmol, 4.0 eq). The crude product was purified by reverse phase HPLC using a gradient from 15% to 75% MeCN in water to give **S15** (20 mg, 32 µmol, 69%) as a colorless powder. HRMS (ESI) m/z calcd for $C_{32}H_{36}N_5O_7S$ $[M+H]^+$ 634.2330, found 634.2314. ^1H NMR (600 MHz, $CDCl_3$) δ 8.38 – 8.34 (m, 1H), 7.96 (d, J = 8.8 Hz, 1H), 7.87 (dd, J = 7.8, 1.4 Hz, 1H), 7.66 – 7.63 (m, 1H), 7.54 – 7.50 (m, 2H), 7.48 – 7.44 (m, 1H), 7.39 (d, J = 7.3 Hz, 1H), 7.36 (td, J = 7.6, 1.3 Hz, 1H), 7.24 (td, J = 7.8, 1.4 Hz, 1H), 7.10 (dd, J = 7.6, 1.3 Hz, 1H), 6.34 (d, J = 7.3 Hz, 1H), 5.22 (d, J = 15.2 Hz, 1H), 5.16 (td, J = 8.6, 4.5 Hz, 1H), 5.10 (dd, J = 11.2, 2.6 Hz, 1H), 4.79 (ddd, J = 8.8, 2.6, 2.3 Hz, 1H), 4.61 (d, J = 15.2 Hz, 1H), 4.58 (dd, J = 8.0, 2.9 Hz, 1H), 4.40 (d, J = 10.2 Hz, 1H), 4.39 (dd, J = 11.2, 2.3 Hz, 1H), 3.94 – 3.87 (m, 1H), 3.83 – 3.77 (m, 1H), 3.61 (d, J = 10.2 Hz, 1H), 3.12 (s, 3H), 3.05 (dd, J = 14.6, 4.5 Hz, 1H), 3.00 – 2.95 (m, 1H), 2.97 (s, 3H), 2.44 – 2.39 (m, 1H), 2.33 – 2.27 (m, 1H), 2.14 – 2.08 (m, 1H), 2.01 – 1.94 (m, 1H). ^{13}C NMR (151 MHz, $CDCl_3$) δ 170.8, 169.8, 169.1, 168.5, 167.6, 162.5, 137.5, 137.2, 133.7, 132.4, 132.3, 132.2, 131.8, 129.6, 127.8, 127.6, 126.7, 125.9, 125.7, 105.5, 66.5, 60.4, 53.5, 50.4, 49.8, 47.2, 38.4, 37.2, 35.9, 35.4, 27.0, 25.5.

(4S,7R)-N,N-dimethyl-6,10-dioxo-7-((S)-1-(2-(pyridin-3-yl)acetyl)pyrrolidine-2-carboxamido)-1,3,4,5,6,7,8,10-octahydrobenzo[j][1]oxa[8]thia[5]azacyclododecine-4-carboxamide (**S16**).

Compound **S16** was prepared following the procedure described for the synthesis of compound **S1** using compound **108** (25 mg, 46 µmol, 1.0 eq), 2-(pyridin-3-yl)acetic acid (enamine code EN300-53587, 13 mg, 92 µmol, 2.0 eq), HATU (35 mg, 92 µmol, 2.0 eq) and DIPEA (31 µL, 0.18 mmol, 4.0 eq). The crude product was purified by reverse phase HPLC using a gradient from 15% to 75% MeCN in water to give **S16** (12 mg, 20 µmol, 45%) as a colorless powder. HRMS (ESI) m/z calcd for $C_{28}H_{34}N_5O_6S$ $[M+H]^+$ 568.2224, found 568.2217. ^1H NMR (600 MHz, $CDCl_3$) δ 8.50 (dd, J = 4.8, 2.0 Hz, 1H), 8.47 – 8.44 (m, 1H), 8.15 – 8.08 (m, 1H), 7.92 (dd, J = 7.8, 1.4 Hz, 1H), 7.62 (dt, J = 7.9, 2.0 Hz, 1H), 7.50 (d, J = 9.0 Hz, 1H), 7.44 (td, J = 7.8, 1.4 Hz, 1H), 7.31 (td, J = 7.7, 1.3 Hz, 1H), 7.28 (dd, J = 7.7, 1.3 Hz, 1H), 7.18 (ddd, J = 7.9, 4.8, 2.0 Hz, 1H), 5.14 – 5.09 (m, 1H), 5.10 (dd, J = 11.2, 2.7 Hz, 1H), 4.84 (ddd, J = 9.0, 2.7, 2.2 Hz, 1H), 4.55 (dd, J = 8.0, 2.7 Hz, 1H), 4.41 (dd, J = 11.2, 2.2 Hz, 1H), 4.33 (d, J = 10.0 Hz, 1H), 3.94 (d, J = 16.2 Hz, 1H), 3.89 – 3.83 (m, 1H), 3.70 (d, J = 16.2 Hz, 1H), 3.58 – 3.53 (m, 1H), 3.52 (d, J = 10.0 Hz, 1H), 3.07 (s, 3H), 2.98 (dd, J = 14.7, 4.5 Hz, 1H), 2.94 (s, 3H), 2.65 (dd, J = 14.7, 9.3 Hz, 1H), 2.43 – 2.37 (m, 1H), 2.33 – 2.22 (m, 1H), 2.06 – 2.00 (m, 1H), 1.98 – 1.89 (m, 1H). ^{13}C NMR (151 MHz, $CDCl_3$) δ 171.4, 171.0, 169.6, 168.6, 167.8,

150.8, 148.2, 137.8, 137.2, 132.6, 132.2, 132.1, 130.3, 129.7, 127.6, 123.2, 66.7, 60.4, 53.3, 49.7, 47.8, 38.3, 38.2, 37.1, 35.8, 35.6, 27.4, 25.3.

(4S,7R)-N,N-dimethyl-7-((S)-1-(2-(2-methyl-4-oxo-5-(thiophen-2-yl)thieno[2,3-d]pyrimidin-3(4H)-yl)acetyl)pyrrolidine-2-carboxamido)-6,10-dioxo-1,3,4,5,6,7,8,10-octahydrobenzo[j][1]oxa[8]thia[5]azacyclododecine-4-carboxamide (**S17**).

Compound **S17** was prepared following the procedure described for the synthesis of compound **S1** using compound **108** (30 mg, 55 μmol, 1.0 eq), 2-[2-methyl-4-oxo-5-(thiophen-2-yl)-3H,4H-thieno[2,3-d]pyrimidin-3-yl]acetic acid (enamine code EN300-10475, 34 mg, 0.11 mmol, 2.0 eq), HATU (42 mg, 0.11 mmol, 2.0 eq) and DIPEA (38 μL, 0.22 mmol, 4.0 eq). The crude product was purified by reverse phase HPLC using a gradient from 25% to 85% MeCN in water to give **S17** (19 mg, 25 μmol, 45%) as a colorless powder. HRMS (ESI) m/z calcd for $C_{34}H_{37}N_6O_7S_3$ $[M+H]^+$ 737.1886, found 737.1870. ^{1}H NMR (500 MHz, $CDCl_3$) δ 8.15 (d, J = 7.9 Hz, 1H), 7.85 (dd, J = 7.6, 1.3 Hz, 1H), 7.53 (dd, J = 3.7, 1.1 Hz, 1H), 7.51 (d, J = 7.7 Hz, 1H), 7.48 (td, J = 7.8, 1.5 Hz, 1H), 7.33 (td, J = 7.6, 1.3 Hz, 1H), 7.27 (dd, J = 5.2, 1.1 Hz, 1H), 7.20 (dd, J = 7.8, 1.5 Hz, 1H), 7.17 (s, 1H), 7.05 (dd, J = 5.2, 3.7 Hz, 1H), 5.12 (d, J = 16.1 Hz, 1H), 5.06 (dd, J = 11.4, 2.8 Hz, 1H), 5.06 – 5.00 (m, 1H), 4.84 (d, J = 16.1 Hz, 1H), 4.73 (ddd, J = 7.9, 2.4, 2.1 Hz, 1H), 4.62 (dd, J = 8.0, 2.3 Hz, 1H), 4.44 (dd, J = 11.4, 2.1 Hz, 1H), 4.09 (d, J = 10.4 Hz, 1H), 3.91 (d, J = 10.4 Hz, 1H), 3.87 – 3.76 (m, 2H), 3.07 (dd, J = 14.3, 4.7 Hz, 1H), 3.03 (s, 3H), 2.92 (dd, J = 14.3, 6.5 Hz, 1H), 2.88 (s, 3H), 2.47 – 2.41 (m, 1H), 2.29 (s, 3H), 2.22 – 2.15 (m, 1H), 2.12 – 2.05 (m, 1H), 1.99 – 1.91 (m, 1H). ^{13}C NMR (126 MHz, $CDCl_3$) δ 171.1, 169.2, 168.4, 168.3, 167.7, 165.2, 158.2, 156.1, 137.3, 136.6, 132.5, 131.8, 131.6, 131.5, 129.9, 128.1, 127.8, 127.3, 125.5, 119.7, 118.0, 66.0, 60.4, 54.1, 49.3, 47.1, 45.7, 37.5, 36.9, 35.8, 35.1, 26.9, 25.2, 23.4

(4S,7R)-N,N-dimethyl-7-((S)-1-(2-methyl-1-oxo-1,2-dihydroisoquinoline-4-carbonyl)pyrrolidine-2-carboxamido)-6,10-dioxo-1,3,4,5,6,7,8,10-octahydrobenzo[j][1]oxa[8]thia[5]azacyclododecine-4-carboxamide (**S18**).

Compound **S18** was prepared following the procedure described for the synthesis of compound **S1** using compound **108** (20 mg, 36 μmol, 1.0 eq), 2-methyl-1-oxo-1,2-dihydroisoquinoline-4-carboxylic acid (enamine code EN300-180165, 15 mg, 72 μmol, 2.0 eq), HATU (27 mg, 72 μmol, 2.0 eq) and DIPEA (25 μL, 0.14 mmol, 4.0 eq). The crude product was purified by reverse phase HPLC using a gradient from 25% to 85% MeCN in water to give **S18** (13 mg, 20

μmol, 56%) as a colorless powder. HRMS (ESI) m/z calcd for $C_{32}H_{36}N_5O_7S$ $[M+H]^+$ 634.2335, found 634.2329. ^{1}H NMR (500 MHz, $CDCl_3$) δ 8.46 (d J = 7.8 Hz, 1H), 8.00 (d, J = 7.9 Hz, 1H), 7.89 (s, 1H), 7.83 (d, J = 7.6, 1H), 7.71 (d, J = 8.5 Hz, 1H), 7.65 (t, J = 8.0 Hz, 1H), 7.59 (d, J = 8.0 Hz, 1H), 7.51 (t, J = 7.8 Hz, 1H), 7.32 (t, J = 7.7, 1H), 7.26 – 7.21 (m, 1H), 7.10 (t, J = 7.6 Hz, 1H), 5.21 – 5.13 (m, 2H), 4.82 (dt, J = 7.9, 2.3 Hz, 1H), 4.80 – 4.75 (m, 1H), 4.53 (dd, J = 11.2, 2.3 Hz, 1H), 4.22 (d, J = 10.4 Hz, 1H), 3.95 (d, J = 10.4 Hz, 1H), 3.64 – 3.59 (m, 1H), 3.59 (s, 3H), 3.46 – 3.39 (m, 1H), 3.08 (s, 3H), 3.07 – 2.95 (m, 2H), 2.84 (s, 3H), 2.50 – 2.41 (m, 1H), 2.19 – 2.07 (m, 2H), 1.89 – 1.81 (m, 1H). ^{13}C NMR (126 MHz, $CDCl_3$) δ 171.7, 169.5, 168.6, 168.5, 167.9, 162.2, 137.4, 135.0, 133.5, 132.6, 132.4, 131.8, 131.6, 129.5, 128.2, 127.4, 127.2, 125.5, 123.8, 113.0, 66.2, 60.4, 53.9, 49.9, 49.5, 37.1, 37.1, 37.0, 35.9, 35.5, 27.6, 25.3.

(4S,7R)-N,N-dimethyl-7-((S)-1-(2-methyl-5-(N-methylsulfamoyl)benzoyl)pyrrolidine-2-carboxamido)-6,10-dioxo-1,3,4,5,6,7,8,10-octahydrobenzo[j][1]oxa[8]thia[5]azacyclododecine-4-carboxamide (**S19**).

Compound **S19** was prepared following the procedure described for the synthesis of compound **S1** using compound **108** (30 mg, 55 μmol, 1.0 eq), 2-methyl-5-(methylsulfamoyl)benzoic acid (enamine code EN300-16252, 25 mg, 0.11 mmol, 2.0 eq), HATU (42 mg, 0.11 mmol, 2.0 eq) and DIPEA (38 μL, 0.22 mmol, 4.0 eq). The crude product was purified by reverse phase HPLC using a gradient from 25% to 85% MeCN in water to give **S19** (22 mg, 34 μmol, 62%) as a colorless powder. HRMS (ESI) m/z calcd for $C_{30}H_{38}N_5O_8S$ $[M+H]^+$ 660.2162, found 660.2150. ^{1}H NMR (500 MHz, $CDCl_3$) δ 7.97 – 7.92 (m, 2H), 7.90 – 7.81 (m, 2H), 7.47 – 7.42 (m, 2H), 7.38 – 7.31 (m, 3H), 6.60 (d, J = 5.2 Hz, 1H), 5.25 – 5.18 (m, 2H), 4.96 (ddd, J = 8.8, 2.4, 2.1 Hz, 1H), 4.66 (dd, J = 8.1, 4.1 Hz, 1H), 4.51 (dd, J = 11.1, 2.1 Hz, 1H), 4.33 (d, J = 9.7 Hz, 1H), 3.89 (d, J = 9.7 Hz, 1H), 3.41 (m, 1H), 3.22 (s, 3H), 3.20 (m, 1H), 3.03 (s, 3H), 3.02 – 2.93 (m, 2H), 2.55 (d, J = 5.2 Hz, 3H), 2.53 – 2.47 (m, 1H), 2.35 (s, 3H), 2.28 – 2.19 (m, 1H), 2.15 – 2.05 (m, 1H), 1.95 – 1.87 (m, 1H). ^{13}C NMR (126 MHz, $CDCl_3$) δ 170.9, 170.5, 170.2, 168.9, 167.8, 138.1, 137.4, 137.3, 136.8, 132.6, 132.4, 132.0, 130.5, 129.8, 128.5, 127.7, 125.5, 66.9, 60.1, 53.5, 50.0, 49.4, 37.7, 37.4, 36.7, 35.2, 29.5, 27.8, 25.4, 19.1.

(4S,7R)-7-((S)-1-(2-(2,2-dioxido-3-phenylbenzo[c][1,2,5]thiadiazol-1(3H)-yl)acetyl)pyrrolidine-2-carboxamido)-N,N-dimethyl-6,10-dioxo-1,3,4,5,6,7,8,10-octahydrobenzo[j][1]oxa[8]thia[5]azacyclododecine-4-carboxamide (**S20**).

Compound **S20** was prepared following the procedure described for the synthesis of compound **S1** using compound **108** (20 mg, 36 μmol, 1.0 eq), 2-(2,2-dioxo-3-phenyl-1,3-dihydro-2,1,3-benzothiadiazol-1-yl)acetic acid (enamine code EN300-206532, 20 mg, 72 μmol, 2.0 eq), HATU (27 mg, 72 μmol, 2.0 eq) and DIPEA (25 μL, 0.14 mmol, 4.0 eq). The crude product was purified by reverse phase HPLC using a gradient from 25% to 85% MeCN in water to give **S20** (14 mg, 18 μmol, 50%) as a colorless powder. HRMS (ESI) m/z calcd for $C_{35}H_{39}N_6O_8S_2$ $[M+H]^+$ 735.2271, found 735.2272. ^1H NMR (500 MHz, $CDCl_3$) δ 8.00 (d, J = 8.7 Hz, 1H), 7.94 (dd, J = 7.8, 1H), 7.56 – 7.49 (m, 6H), 7.42 (td, J = 7.6 Hz, 1H), 7.34 – 7.27 (m, 2H), 7.10 – 7.05 (m, 1H), 6.94 – 6.88 (m, 2H), 6.64 – 6.60 (m, 1H), 5.20 – 5.16 (m, 1H), 5.15 (dd, J = 11.3, 2.5 Hz, 1H), 4.91 (d, J = 16.6 Hz, 1H), 4.84 (ddd, J = 8.7, 2.5, 2.0 Hz, 1H), 4.66 – 4.62 (m, 1H), 4.61 (d, J = 16.6 Hz, 1H), 4.50 (d, J = 10.2 Hz, 1H), 4.44 (dd, J = 11.3, 2.0 Hz, 1H), 3.89 – 3.82 (m, 2H), 3.81 – 3.74 (m, 1H), 3.07 (s, 3H), 3.05 – 2.97 (m, 2H), 2.95 (s, 3H), 2.46 – 2.37 (m, 1H), 2.37 – 2.29 (m, 1H), 2.14 – 2.06 (m, 1H), 2.03 – 1.92 (m, 1H). ^{13}C NMR (126 MHz, $CDCl_3$) δ 170.6, 169.6, 168.3, 168.0, 167.7, 137.4, 132.6, 132.4, 132.2, 132.2, 130.5, 130.2, 130.2, 129.9, 129.7, 129.5, 128.1, 128.1, 127.6, 122.3, 122.3, 110.4, 109.2, 66.7, 60.7, 53.6, 49.9, 47.5, 45.6, 38.6, 37.1, 35.9, 35.3, 27.2, 25.5.

(4S,7R)-7-((S)-1-(2-(1H-tetrazol-1-yl)benzoyl)pyrrolidine-2-carboxamido)-N,N-dimethyl-6,10-dioxo-1,3,4,5,6,7,8,10-octahydrobenzo[j][1]oxa[8]thia[5]azacyclododecine-4-carboxamide (**S21**).

Compound **S21** was prepared following the procedure described for the synthesis of compound **S1** using compound **108** (20 mg, 36 μmol, 1.0 eq), 2-(1H-1,2,3,4-tetrazol-1-yl)benzoic acid (enamine code EN300-09325, 14 mg, 72 μmol, 2.0 eq), HATU (27 mg, 72 μmol, 2.0 eq) and DIPEA (25 μL, 0.14 mmol, 4.0 eq). The crude product was purified by reverse phase HPLC using a gradient from 25% to 85% MeCN in water to give **S21** (6 mg, 10 μmol, 27%) as a colorless powder. HRMS (ESI) m/z calcd for $C_{29}H_{33}N_8O_6S$ $[M+H]^+$ 621.2244, found 621.2228. ^1H NMR (500 MHz, $CDCl_3$) δ 9.44 (s, 1H), 8.04 (d, J = 9.3 Hz, 1H), 7.89 – 7.85 (m, 1H), 7.73 (d, J = 8.0 Hz, 1H), 7.69 – 7.58 (m, 2H), 7.56 (d, J = 8.0 Hz, 1H), 7.46 (t, J = 7.6 Hz, 1H), 7.39 (d, J = 7.6 Hz, 1H), 7.37 – 7.30 (m, 2H), 5.15 (dd, J = 11.1, 2.5 Hz, 1H), 5.05 (ddd, J = 9.9, 9.3, 4.5 Hz, 1H), 4.84 (ddd, J = 8.3, 2.5, 2.1 Hz, 1H), 4.45 (dd, J = 11.1, 2.1 Hz, 1H), 4.26 (m, 1H), 4.20 (d, J = 9.6 Hz, 1H), 3.83 (d, J = 9.6 Hz, 1H), 3.59 – 3.46 (m, 1H), 3.42 – 3.33 (m, 1H), 3.08 (dd, J = 14.9, 4.5 Hz, 1H), 3.01 – 2.96 (m, 1H), 2.96 (s, 3H), 2.52 (s, 3H), 2.21 – 2.11 (m, 2H), 2.09 – 2.05 (m, 1H), 1.85 – 1.75 (m, 1H). ^{13}C NMR (126 MHz, $CDCl_3$) δ 171.6, 169.2,

168.8, 167.9, 166.2, 145.1, 137.0, 133.0, 132.6, 131.9, 131.5, 131.4, 131.4, 130.6, 129.3, 128.5, 127.8, 126.7, 61.5, 60.6, 53.4, 50.1, 50.0, 38.4, 37.1, 35.9, 35.6, 28.9, 25.6.

3-((S)-2-(((4S,7R)-4-(dimethylcarbamoyl)-6,10-dioxo-1,3,4,5,6,7,8,10-octahydrobenzo[j][1]oxa[8]thia[5]azacyclododecin-7-yl)carbamoyl)pyrrolidine-1-carbonyl)benzoic acid (**S22**).

Compound **108** (32 mg, 54 µmol, 1.0 eq) was dissolved in 4 M HCl in dioxane (3 mL) and stirred for 1 hour at rt. After evaporation of the volatiles under reduced pressure, the resulting salt was dissolved in DMSO (1 mL). Isophthalic acid (18 mg, 0.11 mmol, 2.0 eq), HATU (42 mg, 0.11 mmol, 2.0 eq) and DIPEA (38 µL, 0.22 mmol, 4.0 eq) were added and the reaction was stirred for 2 additional hours at rt. EtOAc (75 mL) was then added and the mixture was washed with 1 M aqueous HCl (25 mL) and brine (25 mL). The organic phase was dried over $MgSO_4$, filtered and then concentrated under reduced pressure. The crude product was purified by reverse phase HPLC using a gradient from 15% to 75% MeCN (containing 0.1% TFA) in water to give **S22** (8 mg, 13 µmol, 25%) as a colorless powder. HRMS (ESI) m/z calcd for $C_{29}H_{32}NaN_4O_8S$ [M+Na]$^+$ 619.1839, found 619.1850. ^{1}H NMR (500 MHz, $CDCl_3$) δ 8.45 – 8.41 (m, 1H), 8.16 (d, J = 7.8 Hz, 1H), 7.97 (d, J = 9.0 Hz, 1H), 7.91 (d, J = 7.8 Hz, 1H), 7.88 (d, J = 7.8 Hz, 1H), 7.76 (d, J = 8.3 Hz, 1H), 7.52 (t, J = 7.7 Hz, 1H), 7.43 (d, J = 7.8 Hz, 1H), 7.36 – 7.28 (m, 2H), 5.23 – 5.14 (m, 2H), 4.93 (ddd, J = 8.3, 2.4, 2.1 Hz, 1H), 4.63 (dd, J = 7.7, 5.3 Hz, 1H), 4.56 (dd, J = 11.2, 2.1 Hz, 1H), 4.25 (d, J = 9.8 Hz, 1H), 3.90 (d, J = 9.8 Hz, 1H), 3.84 – 3.77 (m, 1H), 3.64 – 3.57 (m, 1H), 3.10 (s, 3H), 3.10 – 3.06 (m, 2H), 2.83 (s, 3H), 2.40 – 2.32 (m, 1H), 2.29 – 2.16 (m, 2H), 1.97 – 1.84 (m, 1H). *The COOH proton was not detectable in this spectrum.* ^{13}C NMR (126 MHz, $CDCl_3$) δ 172.1, 169.9, 169.5, 168.9, 168.3, 168.0, 137.0, 135.5, 132.6, 132.5, 132.3, 132.3, 131.8, 130.0, 129.8, 129.2, 128.6, 127.6, 66.6, 61.4, 53.7, 50.6, 49.9, 37.6, 37.3, 36.1, 35.5, 28.4, 25.8.

(4S,7R)-N,N-dimethyl-7-((S)-1-(2-(N-methylmethylsulfonamido)benzoyl)pyrrolidine-2-carboxamido)-6,10-dioxo-1,3,4,5,6,7,8,10-octahydrobenzo[j][1]oxa[8]thia[5]azacyclododecine-4-carboxamide (**S23**).

Compound **S23** was prepared following the procedure described for the synthesis of compound **S1** using **108** (20 mg, 36 µmol, 1.0 eq), 2-(N-methylmethanesulfonamido)benzoic acid (enamine code EN300-26724, 17 mg, 72 µmol, 2.0 eq), HATU (27 mg, 72 µmol, 2.0 eq) and DIPEA (25 µL, 0.14 mmol, 4.0 eq). The crude product was purified by reverse phase HPLC

using a gradient from 15% to 75% MeCN in water to give **S23** (6 mg, 9 μmol, 25%) as a colorless powder. HRMS (ESI) m/z calcd for $C_{30}H_{37}N_4NaO_8S_2$ $[M+Na]^+$ 681.1981, found 682.1992. ^1H NMR (500 MHz, $CDCl_3$) δ 7.96 – 7.87 (m, 2H), 7.71 (d, J = 8.2 Hz, 1H), 7.57 (d, J = 7.5 Hz, 1H), 7.50 – 7.42 (m, 2H), 7.40 – 7.29 (m, 4H), 5.21 (dd, J = 11.1, 2.6 Hz, 1H), 5.11 (td, J = 8.7, 4.7 Hz, 1H), 4.92 (ddd, J = 8.2, 2.6, 2.2 Hz, 1H), 4.56 (t, J = 7.4 Hz, 1H), 4.52 (dd, J = 11.1, 2.2 Hz, 1H), 4.32 (d, J = 10.0 Hz, 1H), 3.83 (d, J = 10.0 Hz, 1H), 3.62 – 3.48 (m, 2H), 3.30 (s, 3H), 3.06 (dd, J = 14.7, 4.7 Hz, 1H), 3.01 (s, 3H), 2.98 (dd, J = 14.7, 8.7 Hz, 1H), 2.94 (s, 3H), 2.69 (s, 3H), 2.38 – 2.30 (m, 1H), 2.25 – 2.18 (m, 1H), 2.08 – 2.00 (m, 1H), 1.94 – 1.84 (m, 1H). ^{13}C NMR (126 MHz, $CDCl_3$) δ 172.0, 169.1, 169.0, 168.9, 167.8, 138.5, 137.3, 137.2, 132.5, 132.1, 131.9, 130.7, 129.9, 128.5, 128.4, 128.2, 127.6, 66.5, 61.1, 53.5, 50.1, 50.0, 39.4, 38.2, 37.6, 37.0, 35.8, 35.7, 28.6, 25.4.

(4S,7R)-7-((S)-1-(2-acetamidobenzoyl)pyrrolidine-2-carboxamido)-N,N-dimethyl-6,10-dioxo-1,3,4,5,6,7,8,10-octahydrobenzo[j][1]oxa[8]thia[5]azacyclododecine-4-carboxamide (**S24**). Compound **108** (25 mg, 46 μmol, 1.0 eq) was dissolved in 4 M HCl in dioxane (2 mL) and the mixture was stirred for 1 hour at rt. After evaporation of the volatiles under reduced pressure, the resulting salt was dissolved in DMSO (1 mL). Anthranilic acid (13 mg, 92 μmol, 2.0 eq), HATU (35 mg, 92 μmol, 2.0 eq) and DIPEA (31 μL, 0.18 mmol, 4.0 eq) were added and the mixture was stirred for 2 hours at rt. EtOAc (50 mL) was then added and the mixture was washed with 1 M aqueous HCl solution (25 mL) and brine (25 mL). The organic phase was dried over $MgSO_4$, filtered and then concentrated under reduced pressure. The resulting oil was then dissolved in DCM (2 mL). Acetyl chloride (17 μL, 0.23 mmol, 5.0 eq) and triethylamine (65 μL, 0.46 mmol, 10 eq) were added and the reaction stirred for 2 additional hours at rt. EtOAc (50 mL) was then added and the mixture washed with 1 M aqueous HCl (50 mL) and brine (50 mL). The organic phase was dried over $MgSO_4$, filtered and then concentrated under reduced pressure. The crude product was purified by reverse phase HPLC using a gradient from 15% to 75% MeCN in water to give **S24** (7 mg, 11 μmol, 24% yield) as a colorless powder. HRMS (ESI) m/z calcd for $C_{30}H_{36}N_5O_7S$ $[M+H]^+$ 610.2335, found 610.2341. ^1H NMR (500 MHz, $CDCl_3$) δ 9.35 (s, 1H), 8.13 (d, J = 8.8 Hz, 1H), 8.03 (d, J = 8.2 Hz, 1H), 7.88 (dd, J = 7.8, 1.5 Hz, 1H), 7.52 – 7.28 (m, 6H), 7.15 (td, J = 7.6, 1.2 Hz, 1H), 5.19 (dd, J = 11.1, 2.6 Hz, 1H), 5.18 – 5.12 (m, 1H), 4.91 (ddd, J = 8.5, 2.6, 2.2 Hz, 1H), 4.56 (dd, J = 11.1, 2.2 Hz, 1H), 4.47 – 4.43 (m, 1H), 4.10 (d, J = 9.8 Hz, 1H), 3.97 (d, J = 9.8 Hz, 1H), 3.67 – 3.59 (m, 1H), 3.46 – 3.39 (m, 1H), 3.09 – 2.98 (m, 2H), 3.0 (s, 3H), 2.71 (s, 3H), 2.31 – 2.19 (m, 2H), 2.18

(s, 3H), 2.16 – 2.09 (m, 1H), 1.90 – 1.77 (m, 1H). ^{13}C NMR (126 MHz, $CDCl_3$) δ 172.0, 169.9, 169.4, 169.4, 169.0, 168.3, 136.8, 136.1, 132.5, 132.2, 131.5, 130.9, 130.2, 127.7, 127.4, 127.0, 125.0, 124.1, 66.6, 61.3, 53.7, 50.4, 49.6, 37.0, 36.8, 35.7, 35.3, 29.1, 25.6, 24.2.

Supporting Procedure S3 – Gram scale synthesis of compound 107 and scale-up synthesis of compound 1

Methyl-2-((4S,7R)-4-(dimethylcarbamoyl)-7-(hydroxymethyl)-11,11-dimethyl-6,9-dioxo-10-oxa-2-thia-5,8-diazadodecyl)benzoate (**110**).

Compound **80** (3.74 g, 9.46 mmol, 1.0 eq) was dissolved in MeOH (10 mL) and 4 M HCl in dioxane (20 mL) and stirred for 1 hour at rt. After evaporation of the solvent under reduced pressure, the resulting salt was suspended in water (40 mL). K_2CO_3 (2.61 g, 18.9 mmol, 2.0 eq) was then added and the aqueous phase was extracted with DCM (3 × 100 mL). The combined organic phase was dried over $MgSO_4$, filtered and then concentrated under reduced pressure and the resulting oil was dissolved in MeCN (40 mL). Boc-D-Ser-OH (2.91 g, 14.2 mmol, 1.5 eq) and EDC·HCl (2.71 g, 14.2 mmol, 1.5 eq) were added and the reaction mixture was stirred for 2 additional hours at rt. EtOAc (250 mL) was then added and the mixture was washed with 1 M aqueous HCl solution (100 mL), saturated aqueous NaHCO3 solution (100 mL) and brine (100 mL). The organic phase was dried over $MgSO_4$, filtered, and then concentrated under reduced pressure. The crude product was purified by flash chromatography on a silica gel column using 1-10% MeOH in DCM as eluent to give **110** (3.20 g, 6.62 mmol, 70%) as colourless powder. HRMS (ESI) m/z calcd for $C_{22}H_{34}N_3O_7S$ $[M+H]^+$ 484.2112, found 484.2091. ^{1}H NMR (500 MHz, $CDCl_3$) δ 7.93 (d, J = 7.7 Hz, 1H), 7.48 (m, 1H), 7.40 (m, 1H), 7.35 (m, 1H), 7.15 (d, J = 8.2 Hz, 1H), 5.53 (d, J = 7.9 Hz, 1H), 5.05 (ddd, J = 8.2, 7.5, 5.4 Hz, 1H), 4.26 (m, 1H), 4.22 (d, J = 13.1 Hz, 1H), 4.14 (d, J = 13.0 Hz, 1H), 4.09 (m, 1H), 3.94 (s, 3H), 3.66 (m, 1H), 3.27 (br s, 1H), 3.05 (s, 3H), 2.97 (s, 3H), 2.86 (dd, J = 14.2, 5.4 Hz, 1H), 2.70 (dd, J = 14.2, 7.5 Hz, 1H), 1.47 (s, 9H). ^{13}C NMR (126 MHz, $CDCl_3$) δ 171.0, 170.3, 167.8, 155.7, 139.8, 132.1, 131.2, 131.2, 129.5, 127.4, 80.4, 63.4, 55.5, 52.3, 48.8, 37.3, 36.1, 34.9, 33.9, 28.3.

2-((4S,7R)-4-(Dimethylcarbamoyl)-7-(hydroxymethyl)-11,11-dimethyl-6,9-dioxo-10-oxa-2-thia-5,8-diazadodecyl)benzoic acid (**111**).

Compound **110** (4.02 g, 8.32 mmol, 1.0 eq) was dissolved in MeOH (15 mL) and 0.25 M aqueous LiOH (25 mL) and the reaction mixture was stirred at 40 °C for 16 hours. After being allowed to cool to rt, the reaction mixture was acidified with 1 M aqueous HCl solution (20 mL) and extracted with DCM (3 × 125 mL). The organic phase was dried over $MgSO_4$, filtered and

then concentrated under reduced pressure. The obtained colorless solid (2.22 g, 4.74 mmol, 57%) was used in the next step without further purification. HRMS (ESI) m/z calcd for $C_{21}H_{32}N_3O_7S$ $[M+H]^+$ 470.1955, found 470.1945. ^{1}H NMR (500 MHz, DMSO-d_6, mixture of 2 rotamers in ratio 6:4) δ 13.20 (br s, 1H), 8.21 (d, J = 8.4 Hz, 0.4H), 8.12 (d, J = 8.4 Hz, 0.6H), 7.82 – 7.74 (m, 2H), 7.29 (t, J = 7.9 Hz, 1H), 6.67 (d, J = 8.3 Hz, 0.4H), 6.64 (d, J = 8.2 Hz, 0.6H), 4.89 (m, 1H), 4.33 (m, 1H), 4.25 (d, J = 12.7 Hz, 1H), 4.00 (m, 1H), 3.60 – 3.48 (m, 2H), 3.01 (s, 1.2H), 2.99 (s, 1.8H), 2.91 (dd, J = 13.1, 7.8 Hz, 1H), 2.84 (s, 1.2H), 2.83 (s, 1.8H), 2.68 (dd, J = 13.6, 6.1 Hz, 1H), 1.39 (s, 5.4H), 1.38 (s, 3.6H). The $CH_2O\underline{H}$-proton was not detectable in this spectrum. ^{13}C NMR (126 MHz, DMSO-d_6, mixture of 2 rotamers) δ 170.4 (1C), 170.0 and 169.9 (1C), 168.9 (1C), 155.64 and 155.60 (1C), 140.65 and 140.61 (1C), 132.0 (1C), 131.5 (1C), 131.3 (1C), 130.7 (1C), 127.6 (1C), 78.7 and 78.6 (1C), 62.3 (1C), 57.5 and 57.2 (1C), 48.9 and 48.8 (1C), 37.1 (1C), 35.8 (1C), 34.4 (1C), 33.5 and 33.4 (1C), 28.6 (3C).

Tert-butyl-((4S,7R)-4-(dimethylcarbamoyl)-6,10-dioxo-1,3,4,5,6,7,8,10-octahydrobenzo[j][1]oxa[8]thia[5]azacyclododecin-7-yl)carbamate (**107**).
Compound **111** (2.14 g, 4.56 mmol, 1.0 eq) was dissolved in toluene (115 mL) and cooled down to 0 °C. Ethyldiphenylphosphine (1.88 mL, 9.12 mmol, 2.0 eq) and di-tert-butylazodicarboxylate (2.10 g, 9.12 mmol, 2.0 eq) were added and the mixture was allowed to warm up to rt and stirred for 2 hours. After evaporation of the solvent under reduced pressure, the crude product was purified by flash chromatography on a silica gel column using 25-100% EtOAc in hexane as eluent to give **107** (1.01 g, 2.23 mmol, 49%) as colorless solid. The ^{1}H spectrum was in accordance with our previously published procedure.[10]

(4S,7R)-7-((S)-1-Acetylpyrrolidine-2-carboxamido)-N,N-dimethyl-6,10-dioxo-1,3,4,5,6,7,8,10-octahydrobenzo[j][1]oxa[8]thia[5]azacyclododecine-4-carboxamide (**1**).
Compound **107** (220 mg, 0.48 mmol, 1.0 eq) was dissolved in 4M HCl in dioxane (5 mL) and stirred for 1 hour at rt. After evaporation of the solvent under reduced pressure, the resulting salt was suspended in MeCN (10 mL). HATU (273 mg, 0.72 mmol, 1.5 eq), Ac-L-Pro-OH (150 mg, 0.96 mmol, 2.0 eq) and DIPEA (250 µL, 1.44 mmol, 3.0 eq) were added and the reaction mixture was stirred for 2 additional hours at rt. EtOAc (100 mL) was then added and the mixture was washed with 1 M aqueous HCl solution (25 mL), saturated aqueous NaHCO3 solution (25 mL) and brine (25 mL). The organic phase was dried over $MgSO_4$, filtered, and then concentrated under reduced pressure were added and reaction mixture stirred at rt for 2 hours. The crude reaction mixture was purified by flash chromatography on a silica gel column

using 1 - 10 % MeOH in DCM as eluent to give **1** (190 mg, 0.39 mmol, 81% yield) as a colorless powder. *The ^{1}H spectrum was in accordance with our previously published procedure.*[10]

Supporting Procedure S4 – Gram scale synthesis of compound 10

Methyl-(S)-5-bromo-2-(((2-((tert-butoxycarbonyl)amino)-3-(dimethylamino)-3-oxopropyl)thio)methyl)benzoate (**113**).

Boc-D-Cys-OH (8.04 g, 36.4 mmol, 2.0 eq) was dissolved in DMF (25 mL) and THF (75 mL). Triethylamine (10.3 mL, 72.8 mmol, 4.0 eq) and methyl-5-bromo-2-(bromomethyl)benzoate **112** (5.61 g, 18.2 mmol, 1.0 eq), were added and the mixture was stirred for 16 hours at rt. After evaporation of THF under reduced pressure, the mixture was diluted with EtOAc (300 mL), washed with 1 M aqueous HCl solution (2 × 150 mL) and brine (100 mL). The organic phase was dried over MgSO4, filtered, and then concentrated under reduced pressure. The obtained oil was then dissolved in DMF (25 mL) and THF (75 mL). Dimethylamine hydrochloride (2.97 g, 36.4 mmol, 2.0 eq), EDC·HCl (6.95 g, 36.4 mmol, 2.0 eq), HOBt·xH_2O (3.69 g, 27.3 mmol, 1.5 eq), and DIPEA (6.34 mL, 36.4 mmol, 2.0 eq) were added and the mixture was stirred for 2 additional hours at rt. After evaporation of THF under reduced pressure, the mixture was then diluted with EtOAc (300 mL) and washed with 1 M aqueous HCl solution (150 mL), saturated aqueous $NaHCO_3$ solution (150 mL) and brine (100 mL). The organic phase was dried over MgSO4, filtered, and then concentrated under reduced pressure. The crude product was purified by flash chromatography on a silica column using 25 – 50% EtOAc in hexane as eluent to give **113** (5.62 g, 11.8 mmol, 65%) as a yellow oil. HRMS (ESI) m/z calcd for $C_{19}H_{28}BrN_2O_5S$ $[M+H]^+$ 475.0897, found 475.0887. ^{1}H NMR (500 MHz, $CDCl_3$) δ 8.07 (d, J = 2.2 Hz, 1H), 7.57 (dd, J = 8.2, 2.2 Hz, 1H), 7.31 (d, J = 8.2 Hz, 1H), 5.38 (d, J = 8.8 Hz, 1H), 4.81 (ddd, J = 8.8, 7.5, 5.7 Hz, 1H), 4.13 – 4.09 (m, 2H), 3.93 (s, 3H), 3.11 (s, 3H), 2.99 (s, 3H), 2.78 (dd, J = 13.8, 7.5 Hz, 1H), 2.64 (dd, J = 13.8, 5.7 Hz, 1H), 1.46 (s, 9H). ^{13}C NMR (126 MHz, $CDCl_3$) δ 170.9, 166.3, 155.1, 139.4, 134.8, 134.1, 132.8, 131.0, 120.9, 79.9, 52.5, 49.6, 37.5, 35.9, 34.6, 34.3, 28.3.

Methyl 5-bromo-2-((4S,7R)-4-(dimethylcarbamoyl)-7-(hydroxymethyl)-11,11-dimethyl-6,9-dioxo-10-oxa-2-thia-5,8-diazadodecyl)benzoate (**114**).

Compound **113** (4.02 g, 8.48 mmol, 1.0 eq) was dissolved in 4 M HCl in dioxane (15 mL) and stirred for 1 hour at rt. After evaporation of the volatiles under reduced pressure After evaporation of the volatiles under reduced pressure, water (50 mL) and K_2CO_3 (2.32 g,

16.8 mmol, 2.0 eq) were added and the aqueous phase was extracted with DCM (3 × 150 mL). The organic phase was dried over $MgSO_4$, filtered and then concentrated under reduced pressure. The resulting oil was then dissolved in MeCN (45 mL) and Boc-D-Ser-OH (2.60 g, 12.7 mmol, 1.5 eq) and EDC·HCl (2.43 g, 12.7 mmol, 1.5 eq) were added and the mixture was stirred for 2 additional hours at rt. EtOAc (250 mL) was then added and the mixture washed with 1 M aqueous HCl solution (100 mL), saturated aqueous $NaHCO_3$ solution (100 mL) and brine (100 mL). The organic phase was dried over $MgSO_4$, filtered and then concentrated under reduced pressure. The crude product was purified by flash chromatography on a silica gel column using 1-5% MeOH in DCM as eluent to give **114** (2.81 g, 5.00 mmol, 59%) as a yellow oil. HRMS (ESI) m/z calcd for $C_{22}H_{33}BrN_3O_7S$ $[M+H]^+$ 562.1217, found 562.1209. ^1H NMR (400 MHz, $CDCl_3$) δ 8.03 (d, J = 2.2 Hz, 1H), 7.56 (dd, J = 8.2, 2.2 Hz, 1H), 7.30 – 7.23 (m, 2H), 5.54 (d, J = 7.8 Hz, 1H), 5.03 (m, 1H), 4.23 (m, 1H), 4.11 (d, J = 13.1 Hz, 1H), 4.05 (d, J = 13.1 Hz, 1H), 4.03 (m, 1H), 3.90 (s, 3H), 3.64 (m, 1H), 3.35 (br s, 1H), 3.04 (s, 3H), 2.94 (s, 3H), 2.80 (dd, J = 13.9, 6.3 Hz, 1H), 2.65 (dd, J = 13.9, 7.1 Hz, 1H), 1.43 (s, 9H). ^{13}C NMR (101 MHz, $CDCl_3$) δ 170.9, 170.2, 166.4, 155.7, 139.0, 135.0, 134.0, 132.8, 131.0, 121.0, 80.4, 63.3, 55.6, 52.5, 48.7, 37.3, 36.0, 34.3, 34.0, 28.3.

5-Bromo-2-((4S,7R)-4-(dimethylcarbamoyl)-7-(hydroxymethyl)-11,11-dimethyl-6,9-dioxo-10-oxa-2-thia-5,8-diazadodecyl)benzoic acid (**115**).

Compound **114** (2.80 g, 4.99 mmol, 1.0 eq) was dissolved in MeOH (25mL) and 0.25 M aqueous LiOH (35 mL). Subsequently, the mixture was warmed up to 40° C and stirred for 16 hours. After evaporation of MeOH under reduced pressure, the aqueous phase was acidified with 1 M aqueous HCl solution (20 mL) and extracted with DCM (3 x 100 mL). The combined organic layers were dried over $MgSO_4$, filtered and concentrated under reduced pressure. The obtained colorless solid (2.02 g, 3.67 mmol, 74%) was used in the next step without further purification. HRMS (ESI) m/z calcd for $C_{21}H_{31}BrN_3O_7S$ $[M+H]^+$ 548.1061, found 548.1061. ^1H NMR (500 MHz, DMSO-d_6, , *mixture of 2 rotamers in ratio 3:1*) δ 13.21 (br s, 1H), 8.21 (d, J = 8.6 Hz, 0.25H), 8.16 (d, J = 8.6 Hz, 0.75H), 7.94 (d, J = 2.2 Hz, 1H), 7.69 – 7.65 (m, 1H), 7.38 (d, J = 8.5, 0.75H), 7.36 (d, J = 8.5, 0.25H), 6.70 – 6.63 (m, 1H), 4.83 (ddd, J = 8.6, 8.0, 5.7 Hz, 1H), 4.11 (d, J = 13.1 Hz, 0.75H), 4.09 (d, J = 13.1 Hz, 0.25H), 4.05 – 3.95 (m, 2H), 3.59 – 3.48 (m, 2H), 2.95 (s, 0.75H), 2.94 (s, 2.25H), 2.84 (s, 0.75H), 2.82 (s, 2.25H), 2.76 (dd, J = 13.6, 8.0 Hz, 1H), 2.44 (dd, J = 13.6, 5.7 Hz, 1H), 1.39 (s, 6.75H), 1.38 (s, 2.25H). *The $CH_2O\underline{H}$ proton was not detectable in this spectrum.* ^{13}C NMR (126 MHz, DMSO-d_6, *mixture*

of 2 rotamers) *δ* 170.4 (1C), 169.9 (1C), 167.5 (1C), 155.6 (1C), 140.0 (1C), 134.6 (1C), 133.6 (1C), 133.6(1C), 132.9 (1C), 120.2 (1C), 78.7 (1C), 62.3 (1C), 57.5 and 57.4 (1C), 48.9 and 48.8 (1C), 37.1 and 37.0 (1C), 35.8 and 35.7 (1C), 33.6 and 33.5 (1C), 33.3 and 33.2 (1C), 28.7 and 28.6 (3C).

(4S,7R)-7-((S)-1-acetylpyrrolidine-2-carboxamido)-12-bromo-N,N-dimethyl-6,10-dioxo-1,3,4,5,6,7,8,10-octahydrobenzo[j][1]oxa[8]thia[5]azacyclododecine-4-carboxamide (**10**). Compound **115** (2.01 g, 3.67 mmol, 1.0 eq) was dissolved in THF (90 mL). Triphenylphosphine (1.92 g, 7.34 mmol, 2.0 eq) and di-*tert*-butylazodicarboxylate (1.69 g, 7.34 mmol, 2.0 eq) were then added and the mixture was stirred for 4 hours at rt. After evaporation of the solvent under reduced pressure, the crude mixture was purified to remove the di-*tert*-butylazodicarboxylate side products by flash chromatography on a silica gel column using 80% EtOAc in hexane as eluent. The obtained mixture of crude product and triphenylphosphine oxide was then dissolved in 4 M HCl in dioxane (15 mL) and stirred for 1 hour at rt. After removal of the volatiles under reduced pressure the obtained crude mixture was filtered on a short silica plug using 500 mL of 5% MeOH in EtOAc as eluent to remove to remove the triphenylphosphine oxide side products. Subsequently, the silica was flushed with 500 mL of 25% MeOH/DCM to elute the free amine. After evaporation of the solvent under reduced pressure, the obtained white solid was suspended in MeCN (40 mL). Ac-L-Pro-OH (1.15 g, 7.34 mmol, 2.0 eq *referred to* **115**), EDC·HCl (1.40 g, 7.34 mmol, 2.0 eq *referred to* **115**) and DIPEA (0.96 mL, 5.51 mmol, 1.5 eq *referred to* **115**) were added and the reaction was stirred for 2 hours at rt. The mixture was then diluted with EtOAc (150 mL), and washed with 1 M aqueous HCl solution (100 mL), saturated aqueous $NaHCO_3$ (100 mL) and brine (100 mL). The organic phase was dried over $MgSO_4$, filtered and then concentrated under reduced pressure. The crude reaction mixture was purified by flash chromatography on a silica gel column using 2-5% MeOH in DCM as eluent to give **10** (982 mg, 1.72 mmol, 47% yield over 3 steps) as colorless solid.

Supporting Procedure S5 – Gram scale synthesis of compound 12

Methyl-(S)-3-bromo-2-(((2-((tert-butoxycarbonyl)amino)-3-(dimethylamino)-3-oxopropyl)thio)methyl)benzoate (**117**).

Boc-D-Cys-OH (6.40 g, 29.0 mmol, 2.0 eq) was dissolved in DMF (20 mL) and THF (60 mL). Triethylamine (4.42 mL, 31.9 mmol, 2.2 eq) and methyl 3-bromo-2-(bromomethyl)benzoate

116 (4.47 g, 14.5 mmol, 1.0 eq) were added and the mixture was stirred for 16 hours at rt. After evaporation of THF under reduced pressure, the mixture was diluted with EtOAc (200 mL), washed with 1 M aqueous HCl solution (2 × 100 mL) and brine (100 mL). The organic phase was dried over MgSO4, filtered, and then concentrated under reduced pressure. The obtained oil was dissolved in DMF (20 mL) and THF (60 mL). Dimethylamine hydrochloride (1.77 g, 21.8 mmol, 1.5 eq), EDC·HCl (4.16 g, 21.8 mmol, 1.5 eq), HOBt·xH_2O (2.94 g, 21.8 mmol, 1.5 eq), and DIPEA (3.80 mL, 21.8 mmol, 1.5 eq) were then added and the reaction mixture stirred for 2 additional hours at rt. After evaporation of THF under reduced pressure, the resulting mixture was diluted with EtOAc (250 mL) and then washed with 1 M aqueous HCl solution (50 mL), saturated $NaHCO_3$ solution (50 mL) and brine (50 mL). The organic phase was dried over $MgSO_4$, filtered and concentrated under reduced pressure. The crude product was purified by flash chromatography on a silica column using 10 – 100% EtOAc in hexane as eluent to give **11** (5.54 g, 11.7 mmol, 81%) as a yellow oil. HRMS (ESI) m/z calcd for $C_{14}H_{20}BrN_2O_3S$ [M-Boc + H^+]$^+$ 375.0373 found 375.0370. ^{1}H NMR (400 MHz, $CDCl_3$) δ 7.79 (dd, J = 7.9, 1.4 Hz, 1H), 7.70 (dd, J = 7.9, 1.4 Hz, 1H), 7.15 (t, J = 7.9 Hz, 1H), 5.34 (d, J = 9.1 Hz, 1H), 4.85 – 4.76 (m, 1H), 4.39 – 4.35 (m, 2H), 3.90 (s, 3H), 3.12 (s, 3H), 2.96 (s, 3H), 2.95 – 2.90 (m, 1H), 2.82 (dd, J = 13.5, 6.5 Hz, 1H), 1.43 (s, 9H). ^{13}C NMR (101 MHz, $CDCl_3$) δ 171.0, 167.2, 155.1, 139.6, 136.5, 132.1, 130.1, 128.1, 126.5, 79.7, 52.6, 49.8, 37.4, 35.9, 35.7, 34.1, 28.3.

Methyl-3-bromo-2-((4S,7R)-4-(dimethylcarbamoyl)-7-(hydroxymethyl)-11,11-dimethyl-6,9-dioxo-10-oxa-2-thia-5,8-diazadodecyl)benzoate (**118**).

Compound **117** (5.03 g, 10.6 mmol, 1.0 eq) was dissolved in 4 M HCl in dioxane (25 mL) and the mixture was stirred for 1 hour at rt. After evaporation of the solvent under reduced pressure, the resulting salt was suspended in water (30 mL). K_2CO_3 (2.93 g, 21.2 mmol, 2.0 eq) was then added and the aqueous phase was extracted with DCM (3 × 150 mL). The combined organic phase was dried over $MgSO_4$, filtered and then concentrated under reduced pressure and the resulting oil was dissolved in MeCN (50 mL). Boc-D-Ser-OH (3.26 g, 15.9 mmol, 1.5 eq) and EDC·HCl (3.04 g, 15.9 mmol, 1.5 eq) were added and the reaction mixture was stirred for 2 additional hours at rt. EtOAc (300 mL) was then added and the mixture was washed with 1 M aqueous HCl solution (100 mL), saturated aqueous $NaHCO_3$ solution (100 mL) and brine (100 mL). The organic phase was dried over $MgSO_4$, filtered, and then concentrated under reduced pressure. The crude product was purified by flash chromatography on a silica gel

column using 1-10% MeOH in DCM as eluent to give **118** (3.63 g, 6.47 mmol, 61%) as a yellow oil. HRMS (ESI) m/z calcd for $C_{22}H_{33}BrN_3O_7S$ $[M+H]^+$ 562.1217, found 562.1203. ^1H NMR (400 MHz, $CDCl_3$) δ 7.80 (d, J = 7.9 Hz, 1H), 7.72 (d, J = 7.9 Hz, 1H), 7.17 (t, J = 7.9 Hz, 1H), 7.09 (m, 1H), 5.51 (m, 1H), 5.05 (m, 1H), 4.40 (d, J = 12.9 Hz, 1H), 4.31 (d, J = 12.9 Hz, 1H), 4.24 (m, 1H), 4.09 (m, 1H), 3.92 (s, 3H), 3.62 (m, 1H), 3.33 (br s, 1H), 3.09 (s, 3H), 3.00 (dd, J = 13.7, 5.3 Hz, 1H), 2.94 (s, 3H), 2.81 (dd, J = 13.9, 8.0 Hz, 1H), 1.45 (s, 9H). ^{13}C NMR (126 MHz, $CDCl_3$) δ 171.0, 170.2, 167.4, 155.7, 139.2, 136.7, 132.0, 130.2, 128.3, 126.7, 80.3, 63.4, 55.6, 52.8, 49.0, 37.3, 36.1, 34.8, 34.1, 28.3.

3-Bromo-2-((4S,7R)-4-(dimethylcarbamoyl)-7-(hydroxymethyl)-11,11-dimethyl-6,9-dioxo-10-oxa-2-thia-5,8-diazadodecyl)benzoic acid (**105b**).

Compound **118** (3.10 g, 5.51 mmol, 1.0 eq) was dissolved in MeOH (30 mL) and 0.25 M aqueous LiOH (40 mL). Subsequently, the mixture was warmed up to 40 °C and stirred for 16 hours in a pre-heated oil bath. After evaporation of MeOH under reduced pressure, the mixture was extracted with Et_2O (2 x 50 mL). The aqueous phase was then acidified with 1 M aqueous HCl solution (50 mL) and extracted with DCM (3 x 100 mL). The combined organic layers were dried over $MgSO_4$, filtered and concentrated under reduced pressure. The obtained colorless solid (2.26 g, 4.12 mmol, 75%) was used in the next step without further purification. HRMS (ESI) m/z calcd for $C_{21}H_{31}BrN_3O_7S$ $[M+H]^+$ 548.1061, found 548.1043. *The characterization of this compound is described in the methods section of the manuscript.*

(4S,7R)-7-((S)-1-acetylpyrrolidine-2-carboxamido)-14-bromo-N,N-dimethyl-6,10-dioxo-1,3,4,5,6,7,8,10-octahydrobenzo[j][1]oxa[8]thia[5]azacyclododecine-4-carboxamide (**12**) .

Compound **105b** (2.21 g, 4.03 mmol, 1.0 eq) was dissolved in THF (100 mL). Triphenylphosphine (2.11 g, 8.06 mmol, 2.0 eq) and di-*tert*-butylazodicarboxylate (1.83 g, 8.06 mmol, 2.0 eq) were then added and the mixture was stirred for 4 hours at rt. After evaporation of the solvent under reduced pressure, the crude mixture was purified to remove the di-*tert*-butylazodicarboxylate side products by flash chromatography on a silica gel column using 25 - 100% EtOAc in hexane as eluent. The obtained mixture of crude product and triphenylphosphine oxide and was then dissolved in 4 M HCl in dioxane (15 mL) and stirred for 2 hours at rt. After removal of the volatiles under reduced pressure, water (50 mL) was added and the aqueous phase was extracted with EtOAc (3 × 125 mL) to remove the triphenylphosphine oxide side products. Subsequently, the aqueous phase was neutralized with

K_2CO_3 (1.10 g, 8.06 mmol, 2.0 eq) and then extracted with DCM (3 × 100 mL). The organic phase was dried over $MgSO_4$, filtered and then concentrated under reduced pressure and the obtained solid was dissolved in MeCN (25 mL). Ac-L-Pro-OH (695 mg, 4.43 mmol, 1.1 eq *referred to* **105b**) and EDC·HCl (846 mg, 4.43 mmol, 1.1 eq *referred to* **105b**) were added and the resulting mixture was stirred for 2 additional hours at rt. MeCN was then removed under reduced pressure and the crude reaction mixture was purified by flash chromatography on a silica gel column using 1 – 10% MeOH/DCM as eluent to give **12** (900 mg, 1.58 mmol, 39% yield over 3 steps) as colorless solid. *The characterization of this compound is described in the methods section of the manuscript.*

Supporting Procedure S6 – Synthesis of compound 109

Methyl (S)-2-(((2-((tert-butoxycarbonyl)amino)-3-(dimethylamino)-3-oxopropyl)thio)methyl)-3-iodobenzoate (**120**).

Boc-D-Cys-OH (5.96 g, 27.0 mmol, 2.0 eq) was dissolved in DMF (20 mL) and THF (80 mL). Triethylamine (7.65 mL, 54.0 mmol, 4.0 eq) and methyl 2-(bromomethyl)-3-iodobenzoate **119** (4.80 g, 13.5 mmol, 1.0 eq) were added and the mixture was stirred for 16 hours at rt. After evaporation of THF under reduced pressure, the mixture was diluted with EtOAc (250 mL), washed with 1 M aqueous HCl solution (2 × 100 mL) and brine (100 mL). The organic phase was dried over MgSO4, filtered, and then concentrated under reduced pressure. The obtained oil was then dissolved in DMF (20 mL) and THF (80 mL). Dimethylamine hydrochloride (2.20 g, 27.0 mmol, 2.0 eq), HATU (7.71 g, 20.3 mmol, 1.5 eq) and DIPEA (8.23 mL, 47.3 mmol, 3.5 eq) were added and the mixture was stirred for 2 additional hours at rt. After evaporation of THF under reduced pressure, the mixture was then diluted with EtOAc (250 mL) and washed with 1 M aqueous HCl solution (150 mL), saturated aqueous $NaHCO_3$ solution (150 mL) and brine (100 mL). The organic phase was dried over MgSO4, filtered, and then concentrated under reduced pressure. The crude product was purified by flash chromatography on a silica column using 20 – 80% EtOAc in hexane as eluent to give **120** (4.75 g, 9.09 mmol, 67%) as a colourless oil. HRMS (ESI) m/z calcd for $C_{19}H_{28}IN_2O_5S$ $[M+H]^+$ 523.0764, found 523.0770. ^1H NMR (400 MHz, $CDCl_3$) δ 7.98 (dd, J = 7.9, 1.4 Hz, 1H), 7.79 (dd, J = 7.9, 1.4 Hz, 1H), 6.98 (t, J = 7.9 Hz, 1H), 5.45 – 5.40 (m, 1H), 4.83 – 4.79 (m, 1H), 4.38 – 4.35 (m, 2H), 3.89 (s, 3H), 3.14 (s, 3H), 2.99 – 2.91 (m, 1H), 2.97 (s, 3H), 2.84 (dd, J = 13.5, 6.5 Hz, 1H) 1.43 (s, 9H). ^{13}C NMR (101 MHz, $CDCl_3$) δ 171.2, 167.2, 155.1, 143.4, 142.0, 131.6, 130.8, 128.5, 103.5, 79.8, 52.6, 49.9, 39.5, 37.6, 36.0, 35.6, 28.3.

Methyl 2-((4S,7R)-4-(dimethylcarbamoyl)-7-(hydroxymethyl)-11,11-dimethyl-6,9-dioxo-10-oxa-2-thia-5,8-diazadodecyl)-3-iodobenzoate (**121**).

Compound **120** (4.70 g, 9.00 mmol, 1.0 eq) was dissolved in MeOH (5 mL) 4 M HCl in dioxane (15 mL) and stirred for 1 hour at rt. After evaporation of the volatiles under reduced pressure After evaporation of the volatiles under reduced pressure, water (40 mL) and K_2CO_3 (2.48 g, 18.0 mmol, 2.0 eq) were added and the aqueous phase was extracted with DCM (3 × 150 mL). The organic phase was dried over $MgSO_4$, filtered and then concentrated under reduced pressure. The resulting oil was then dissolved in MeCN (50 mL) and Boc-D-Ser-OH (2.78 g, 13.5 mmol, 1.5 eq) and EDC·HCl (2.58 g, 13.5 mmol, 1.5 eq) were added and the mixture was stirred for 2 additional hours at rt. EtOAc (300 mL) was then added and the mixture washed with 1 M aqueous HCl solution (150 mL), saturated aqueous $NaHCO_3$ solution (150 mL) and brine (100 mL). The organic phase was dried over $MgSO_4$, filtered and then concentrated under reduced pressure. The crude product was purified by flash chromatography on a silica gel column using 1-10% MeOH in DCM as eluent to give **121** (4.08 g, 6.70 mmol, 74%) as yellow oil. HRMS (ESI) m/z calcd for $C_{22}H_{33}IN_3O_7S$ $[M+H]^+$ 610.1084, found 610.1072. ^1H NMR (500 MHz, $CDCl_3$) δ 8.03 (dd, J = 7.9, 1.3 Hz, 1H), 7.85 (dd, J = 7.9, 1.3 Hz, 1H), 7.11 (d, J = 8.5 Hz, 1H), 7.03 (t, J = 7.9 Hz, 1H), 5.55 (d, J = 7.9 Hz, 1H), 5.08 (td, J = 8.5, 8.0, 5.2 Hz, 1H), 4.45 (d, J = 12.8 Hz, 1H), 4.32 (d, J = 12.8 Hz, 1H), 4.30 – 4.24 (m, 1H), 4.17 – 4.11 (m, 1H), 3.95 (s, 3H), 3.66 (dd, J = 11.4, 5.4 Hz, 1H), 3.13 (s, 3H), 3.06 (dd, J = 13.9, 5.2 Hz, 1H), 2.98 (s, 3H), 2.86 (dd, J = 13.9, 8.0 Hz, 1H), 1.48 (s, 9H). *The $CH_2O\underline{H}$ proton was not detectable in this spectrum.* ^{13}C NMR (126 MHz, $CDCl_3$) δ 171.0, 170.2, 167.4, 155.7, 143.6, 141.7, 131.4, 130.9, 128.7, 103.7, 80.4, 63.4, 55.6, 52.8, 49.1, 39.5, 37.3, 36.1, 34.8, 28.3.

2-((4S,7R)-4-(Dimethylcarbamoyl)-7-(hydroxymethyl)-11,11-dimethyl-6,9-dioxo-10-oxa-2-thia-5,8-diazadodecyl)-3-iodobenzoic acid (**122**).

Compound **121** (4.01 g, 6.57 mmol, 1.0 eq) was dissolved in MeOH (30 mL) and 0.25 M aqueous LiOH (30 mL). Subsequently, the mixture was warmed up to 40 °C and stirred for 16 hours. After evaporation of MeOH under reduced pressure, the aqueous phase was acidified with 1 M aqueous HCl solution (20 mL) and extracted with DCM (3 x 125 mL). The combined organic layers were dried over $MgSO_4$, filtered and concentrated under reduced pressure. The obtained colorless solid (2.60 g, 4.37 mmol, 67%)) was used in the next step without further purification. HRMS (ESI) m/z calcd for $C_{21}H_{31}IN_3O_7S$ $[M+H]^+$ 596.0927, found 596.0922. ^1H

NMR (400 MHz, DMSO-d_6 *mixture of 2 rotamers in ratio 3:1*) δ 8.17 (d, J = 8.5 Hz, 0.25H), 8.09 (d, J = 8.5 Hz, 0.75H), 8.01 (dd, J = 7.8, 1.3 Hz, 1H), 7.73 (dd, J = 7.8, 1.3 Hz, 1H), 7.06 (t, J = 7.8 Hz, 1H), 6.62 (d, J = 8.5 Hz, 0.25H), 6.59 (d, J = 8.5 Hz, 0.75H), 4.87 (ddd, J = 8.5, 7.6, 6.2 Hz, 1H), 4.32 (d, J = 12.4 Hz, 1H), 4.21 (dd, J = 12.4 Hz, 1H), 3.99 – 3.91 (m, 1H), 3.55 – 3.44 (m, 2H), 2.99 (s, 0.75H), 2.98 (s, 2.25H), 2.90 (dd, J = 13.4, 7.6 Hz, 1H), 2.82 (s, 0.75H), 2.81 (s, 2.25H), 2.66 (dd, J = 13.4, 6.2 Hz, 1H), 1.36 (s, 9H). *The COOH proton and the CH_2OH proton were not detectable in this spectrum.* ^{13}C NMR (101 MHz, DMSO-d_6 *mixture of 2 rotamers*) δ 170.3 and 170.2 (1C), 170.0 (1C), 168.7 (1C), 155.6 and 155.5 (1C), 143.1 (1C), 141.4 and 141.3 (1C), 133.4 (1C), 130.7 (1C), 129.3 (1C), 104.4 and 104.3 (1C), 78.7 and 78.6 (1C), 62.4 (1C), 57.5 (1C), 49.0 and 48.9 (1C), 39.0 (1C), 37.2 (1C), 35.8 (1C), 34.9 (1C), 28.6 (3C).

Tert-butyl-((4S,7R)-4-(dimethylcarbamoyl)-14-iodo-6,10-dioxo-1,3,4,5,6,7,8,10-octahydrobenzo[j][1]oxa[8]thia[5]azacyclododecin-7-yl)carbamate (**123**).

Compound **122** (2.05 g, 3.44 mmol, 1.0 eq) was dissolved in toluene (85 mL) and cooled down to 0 °C. Ethyldiphenylphosphine (1.42 mL, 6.88 mmol, 2.0 eq) and di-*tert*-butylazodicarboxylate (1.58 g, 6.88 mmol, 2.0 eq) were added and the mixture was allowed to warm up to rt and stirred for 2 hours. After evaporation of the solvent under reduced pressure, the crude product was purified by flash chromatography on a silica gel column using 25-100% EtOAc in hexane as eluent to give **123** (1.04 g, 1.80 mmol, 52%) as colorless solid. HRMS (ESI) m/z calcd for $C_{23}H_{30}IN_4O_6S$ $[M+H]^+$ 617.0931, found 617.0921. ^{1}H NMR (500 MHz, $CDCl_3$) δ 7.99 (dd, J = 7.7 Hz, 1H), 7.74 (d, J = 7.7 Hz, 1H), 7.37 (d, J = 8.5 Hz, 1H), 7.01 (t, J = 7.7 Hz, 1H), 5.58 (d, J = 7.0 Hz, 1H), 5.24 (dd, J = 11.3, 2.7 Hz, 1H), 5.20 – 5.14 (m, 1H), 4.65 (d, J = 8.3 Hz, 1H), 4.37 (dd, J = 11.3, 2.3 Hz, 1H), 4.27 – 4.17 (m, 2H), 3.14 – 3.10 (m, 2H), 3.10 (s, 3H), 2.95 (s, 3H), 1.46 (s, 9H). ^{13}C NMR (126 MHz, $CDCl_3$) δ 169.2, 169.2, 167.5, 155.1, 143.9, 139.0, 132.1, 131.4, 128.9, 103.3, 81.3, 67.1, 55.1, 48.8, 42.5, 37.1, 36.0, 35.8, 28.2.

(4S,7R)-7-((S)-1-Acetylpyrrolidine-2-carboxamido)-14-iodo-N,N-dimethyl-6,10-dioxo-1,3,4,5,6,7,8,10-octahydrobenzo[j][1]oxa[8]thia[5]azacyclododecine-4-carboxamide (**109**).

Compound **123** (1.02 g, 1.76 mmol, 1.0 eq) was dissolved in 4 M HCl in dioxane (10 mL) and stirred for 1 hour at rt. After evaporation of the solvent under reduced pressure, the resulting

salt was suspended in MeCN (20 mL). Ac-L-Pro-OH (552 mg, 3.52 mmol, 2.0 eq), HATU (1.34 g, 3.52 mmol, 2.0 eq) and DIPEA (0.62 mL, 3.52 mmol, 2.0 eq) were added and the reaction mixture was stirred for 2 additional hours at rt. EtOAc (150 mL) was then added and the mixture was washed with 1 M aqueous HCl solution (75 mL), saturated aqueous NaHCO3 solution (75 mL) and brine (100 mL). The organic phase was dried over $MgSO_4$, filtered, and then concentrated under reduced pressure. The crude product was purified by flash chromatography on a silica gel column using 1-10% MeOH in DCM as eluent to give **109** (739 mg, 1.20 mmol, 68% over two steps) as colourless powder. HRMS (ESI) m/z calcd for $C_{22}H_{33}IN_3O_7S$ $[M+H]^+$ 610.1084, found 610.1072. ^1H NMR (500 MHz, $CDCl_3$) δ 8.07 (d, J = 8.7 Hz, 1H), 8.03 (dd, J = 7.9, 1.4 Hz, 1H), 7.86 (dd, J = 7.9, 1.4 Hz, 1H), 7.62 (d, J = 8.9 Hz, 1H), 7.04 (t, J = 7.9 Hz, 1H), 5.30 (dd, J = 11.2, 2.6 Hz, 1H), 5.15 (td, J = 8.9, 4.6 Hz, 1H), 4.90 (ddd, J = 8.7, 2.6, 2.2 Hz, 1H), 4.53 – 4.47 (m, 2H), 4.40 (dd, J = 11.2, 2.2 Hz, 1H), 4.15 (d, J = 10.6 Hz, 1H), 3.81 – 3.75 (m, 1H), 3.53 – 3.45 (m, 1H), 3.12 (dd, J = 14.6, 4.7 Hz, 1H), 3.09 (s, 3H), 3.05 (dd, J = 14.6, 8.9 Hz, 1H), 2.95 (s, 3H), 2.41 – 2.35 (m, 1H), 2.27 – 2.18 (m, 1H), 2.16 (s, 3H), 2.05 – 1.93 (m, 2H). ^{13}C NMR (126 MHz, $CDCl_3$) δ 172.0, 171.6, 169.3, 168.7, 167.3, 144.0, 138.8, 131.9, 131.9, 128.9, 103.8, 66.9, 60.1, 53.4, 49.6, 48.3, 42.9, 37.1, 35.9, 35.9, 27.6, 25.2, 22.5.

NMR spectra for compounds 2–78

2 ^{1}H NMR (500 MHz, $CDCl_3$)

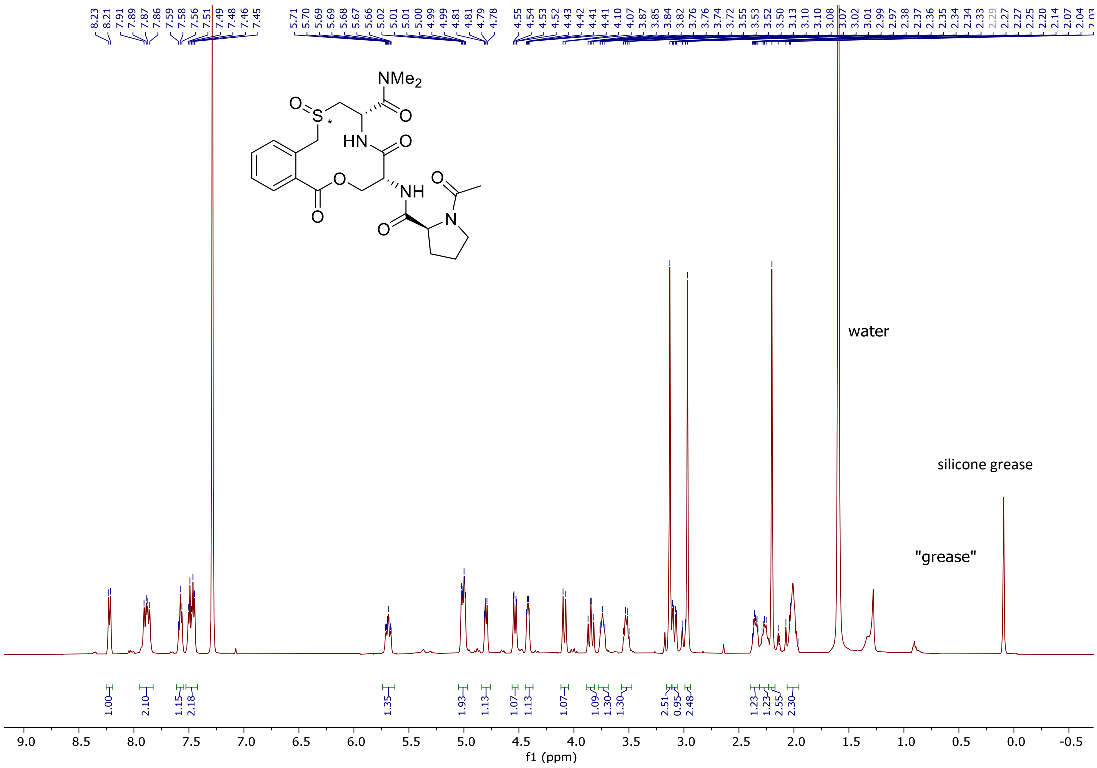

2 ^{13}C NMR (126 MHz, $CDCl_3$)

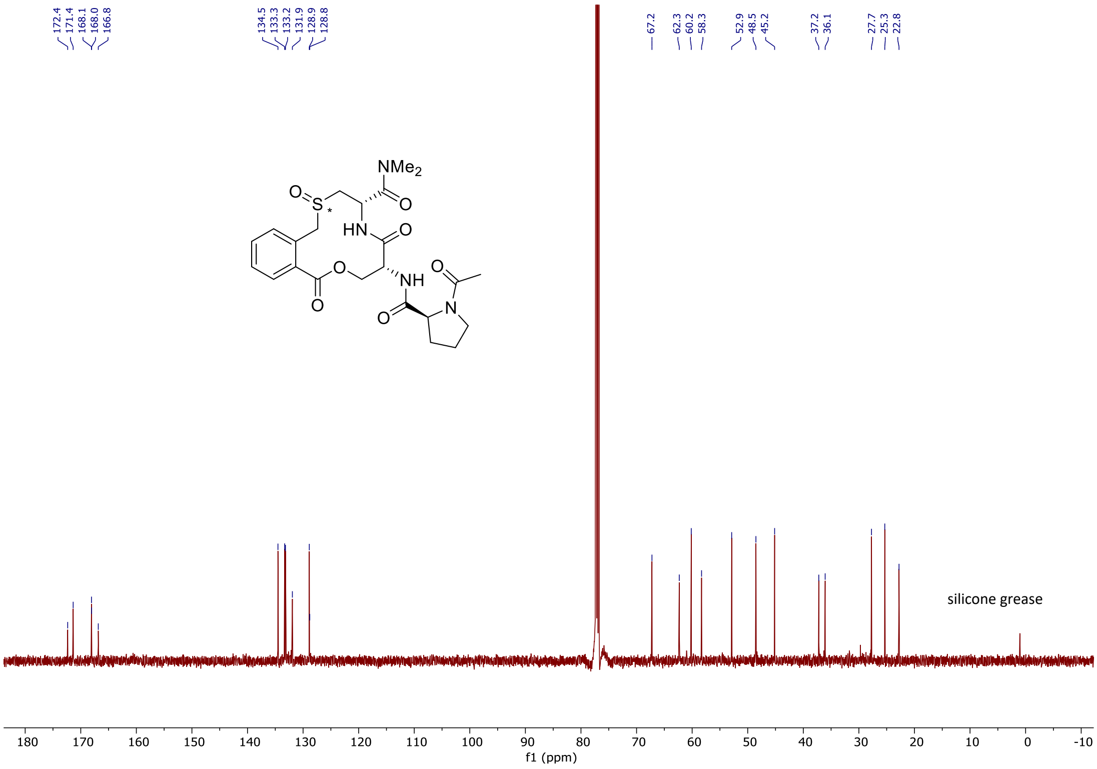

3 ^{1}H NMR (500 MHz, $CDCl_3$)

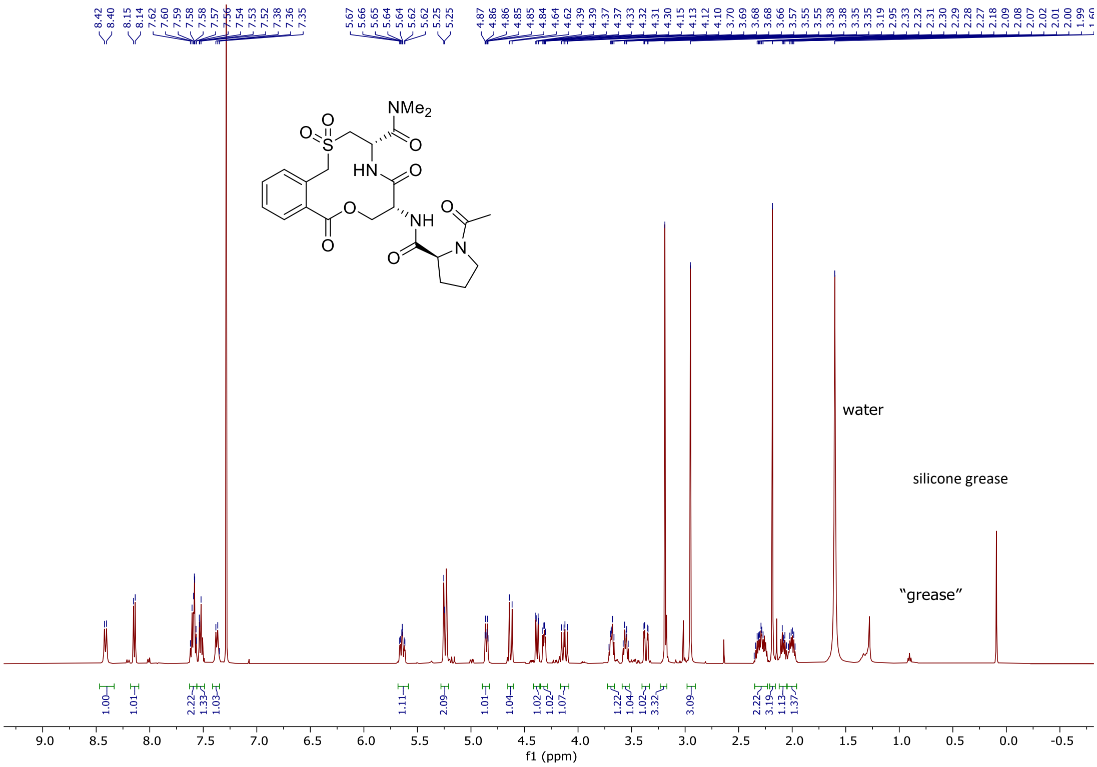

3 ^{13}C NMR (126 MHz, $CDCl_3$)

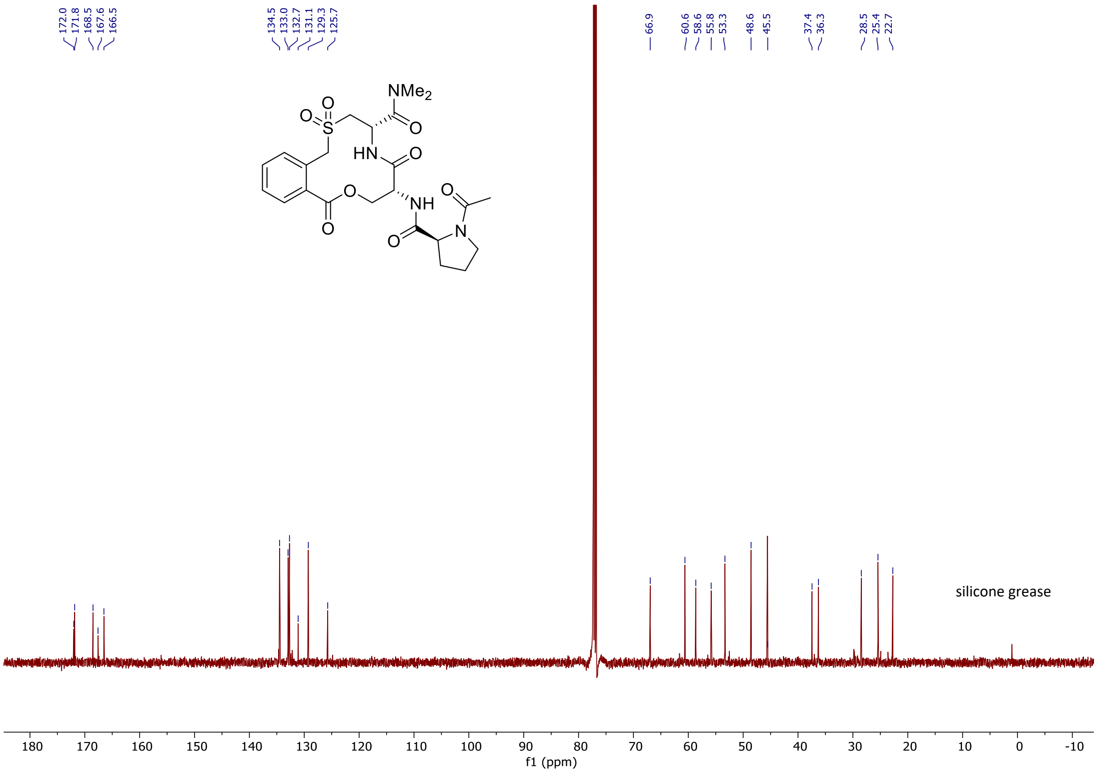

4 ^{1}H NMR (500 MHz, $CDCl_3$)

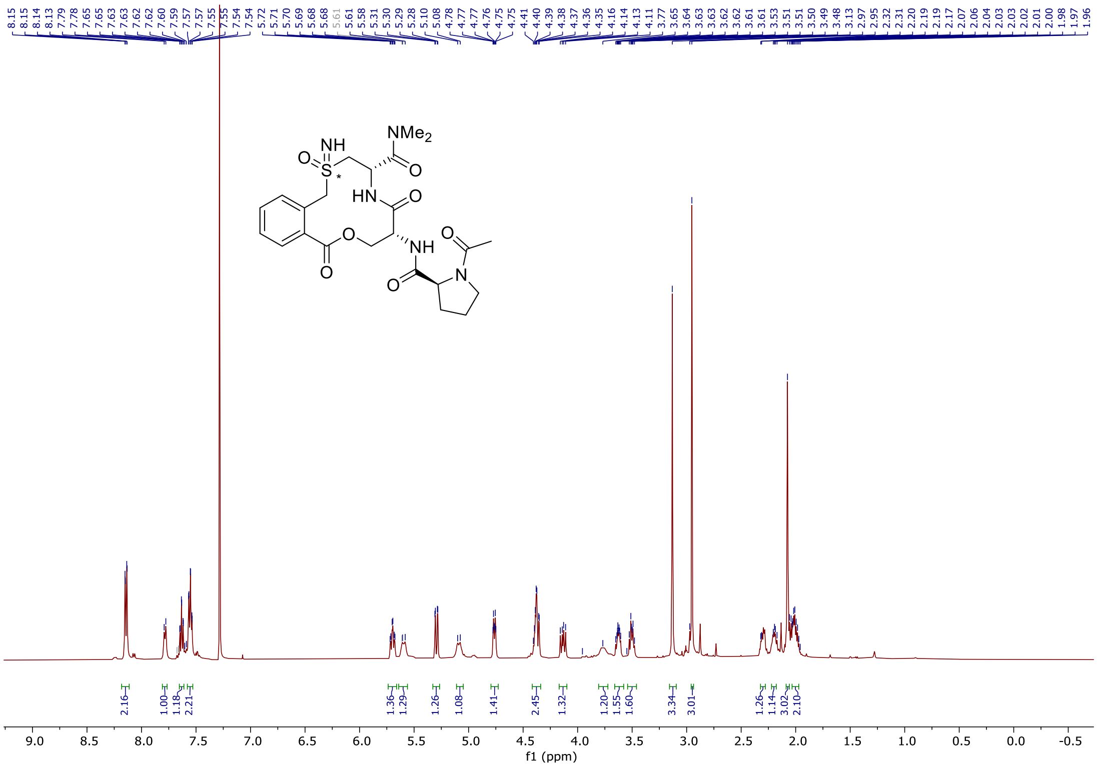

4 ^{13}C NMR (126 MHz, $CDCl_3$)

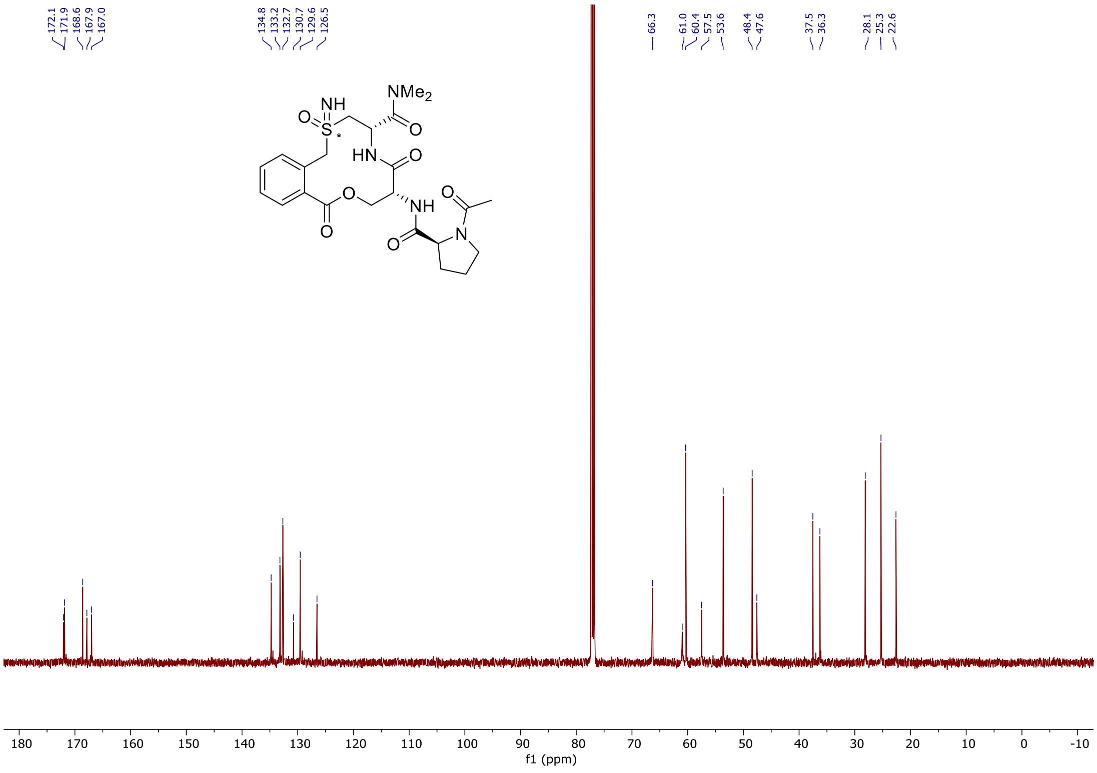

5 ^{1}H NMR (500 MHz, $CDCl_3$)

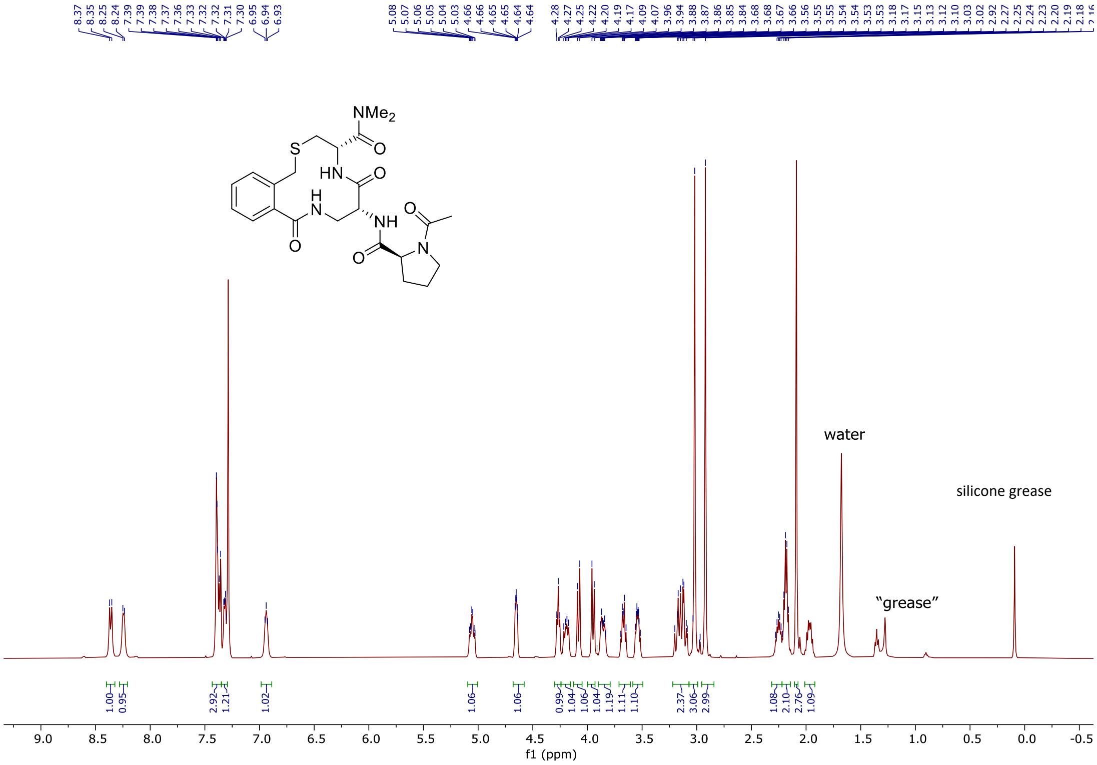

5 ^{13}C NMR (126 MHz, $CDCl_3$)

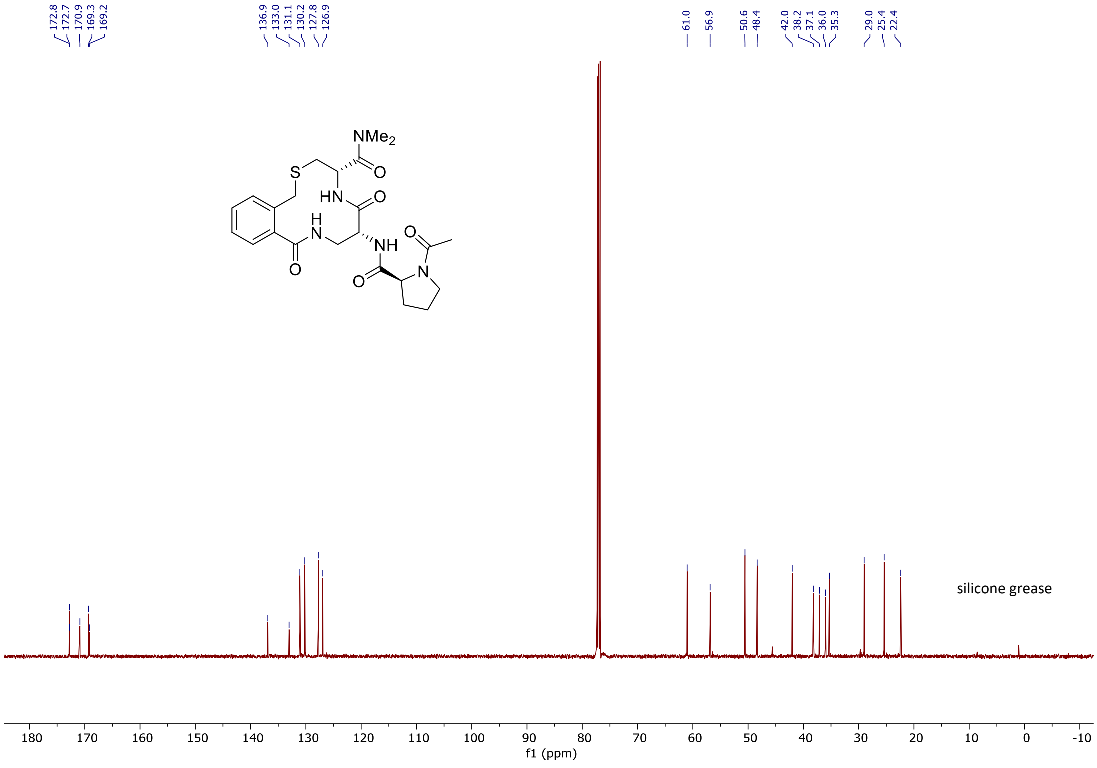

6 ^{1}H NMR (500 MHz, CDCl$_3$)

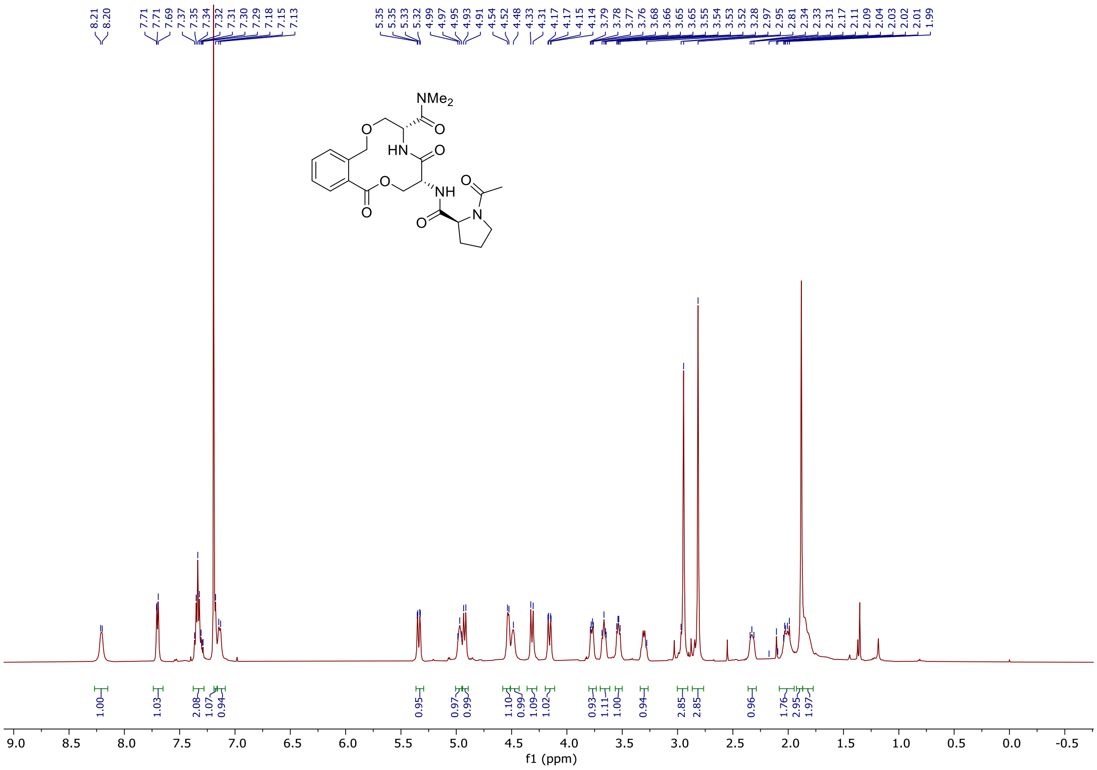

6 ^{13}C NMR (126 MHz, CDCl$_3$)

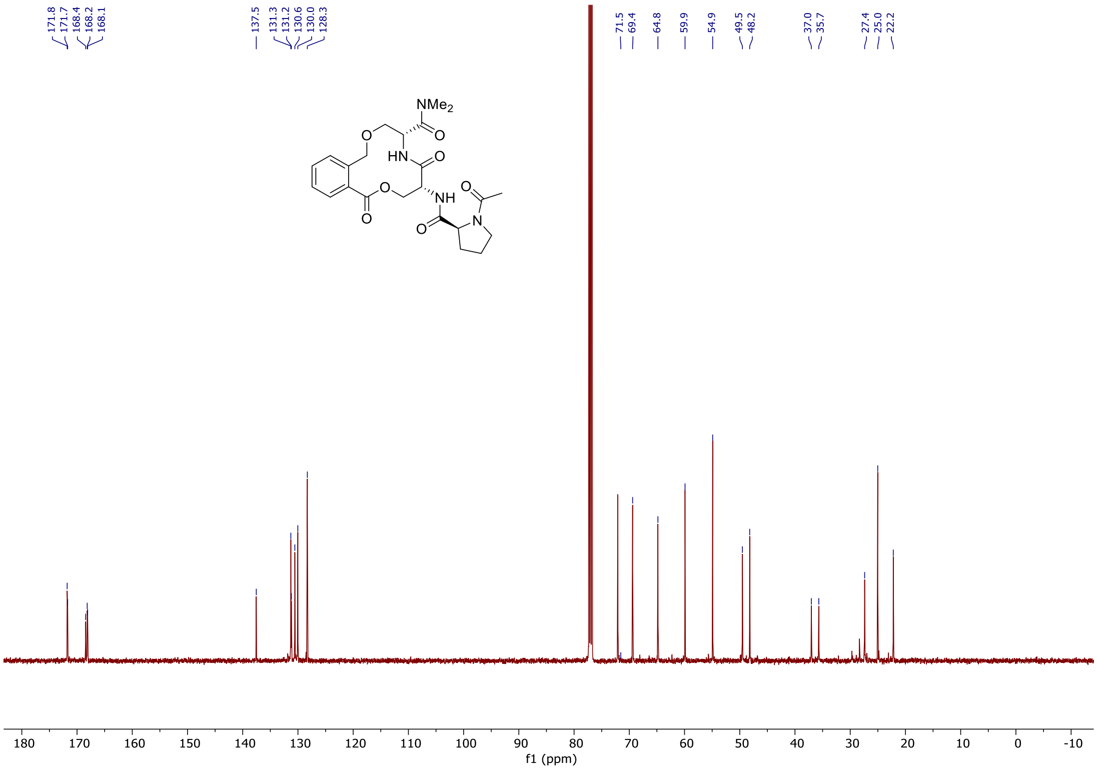

7 ^{1}H NMR (500 MHz, $CDCl_3$)

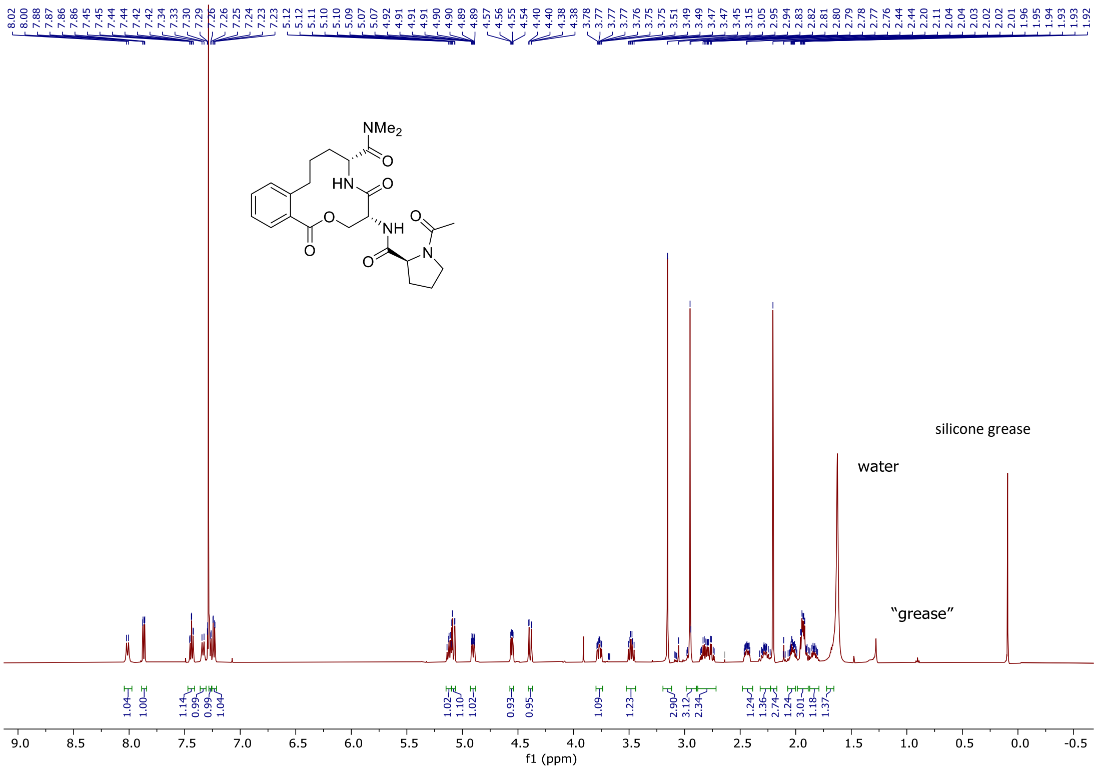

7 ^{13}C NMR (126 MHz, $CDCl_3$)

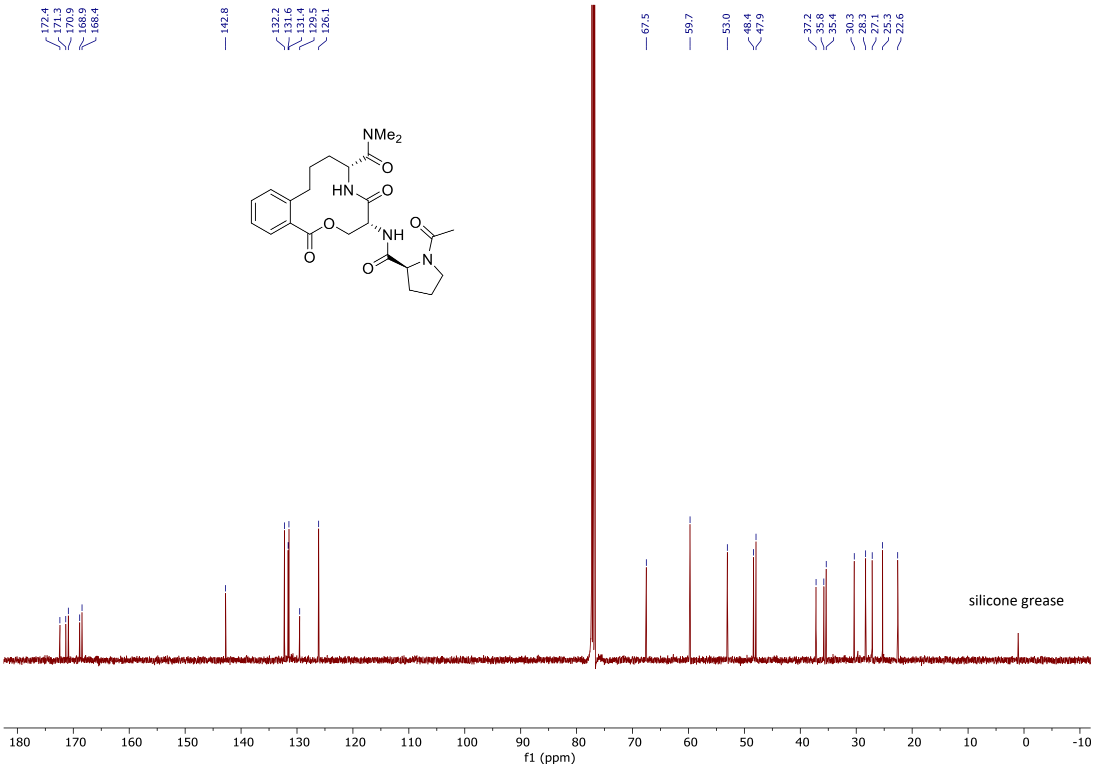

8 ^{1}H NMR (500 MHz, $CDCl_3$)

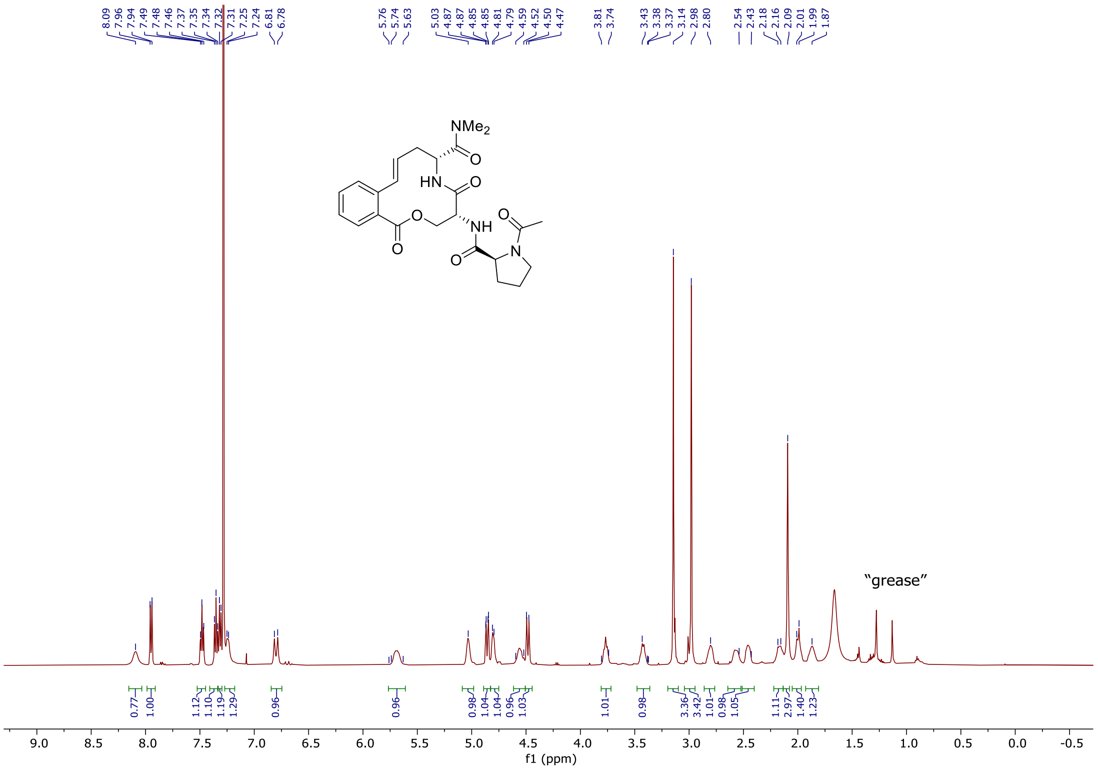

8 ^{13}C NMR (126 MHz, $CDCl_3$)

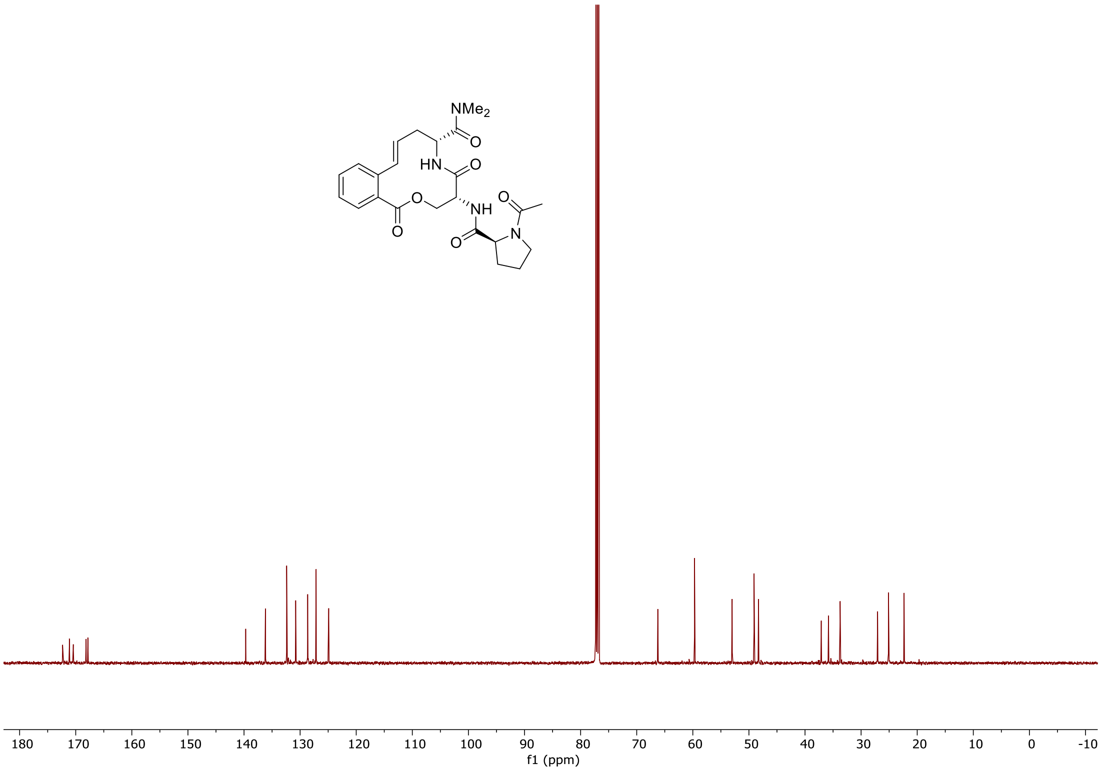

9 ^{1}H NMR (500 MHz, CDCl$_3$)

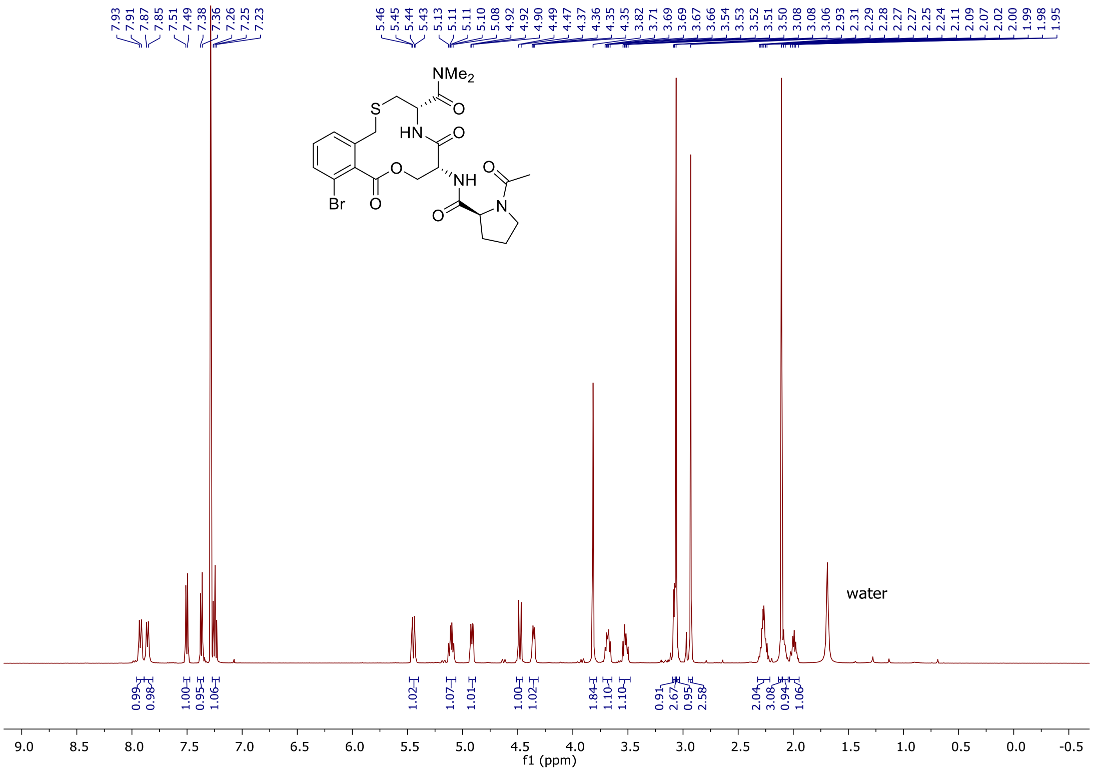

9 ^{13}C NMR (126 MHz, CDCl$_3$)

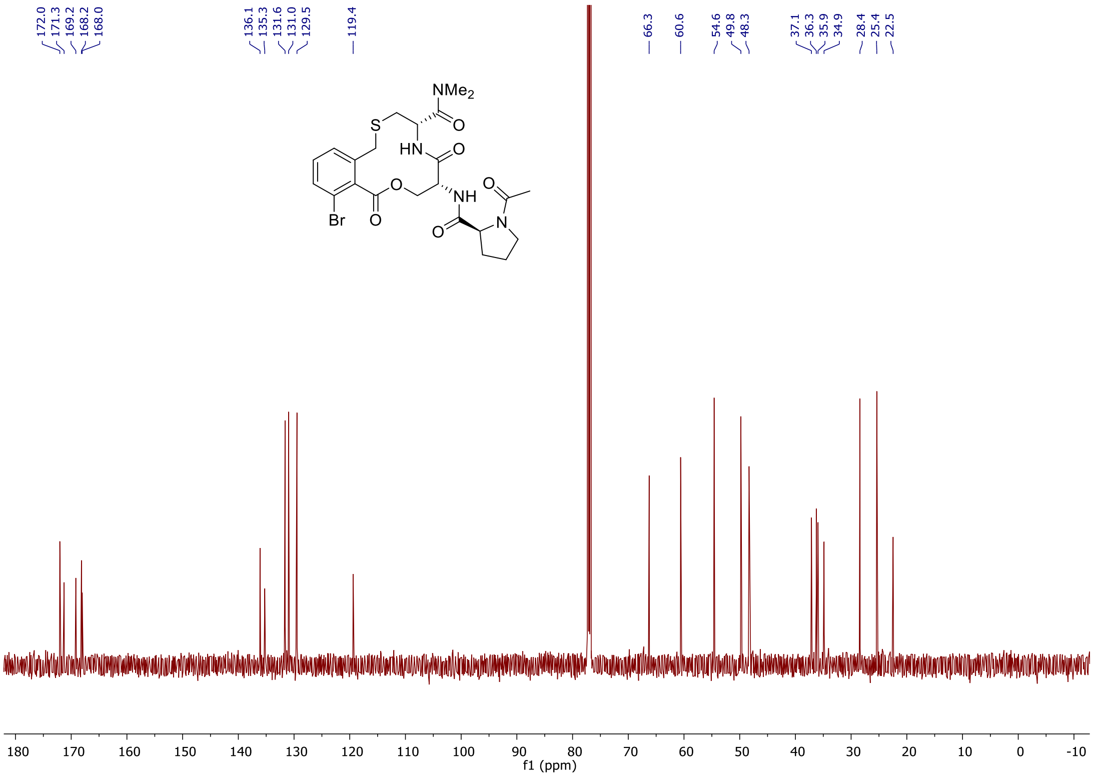

10 ^{1}H NMR (400 MHz, $CDCl_3$)

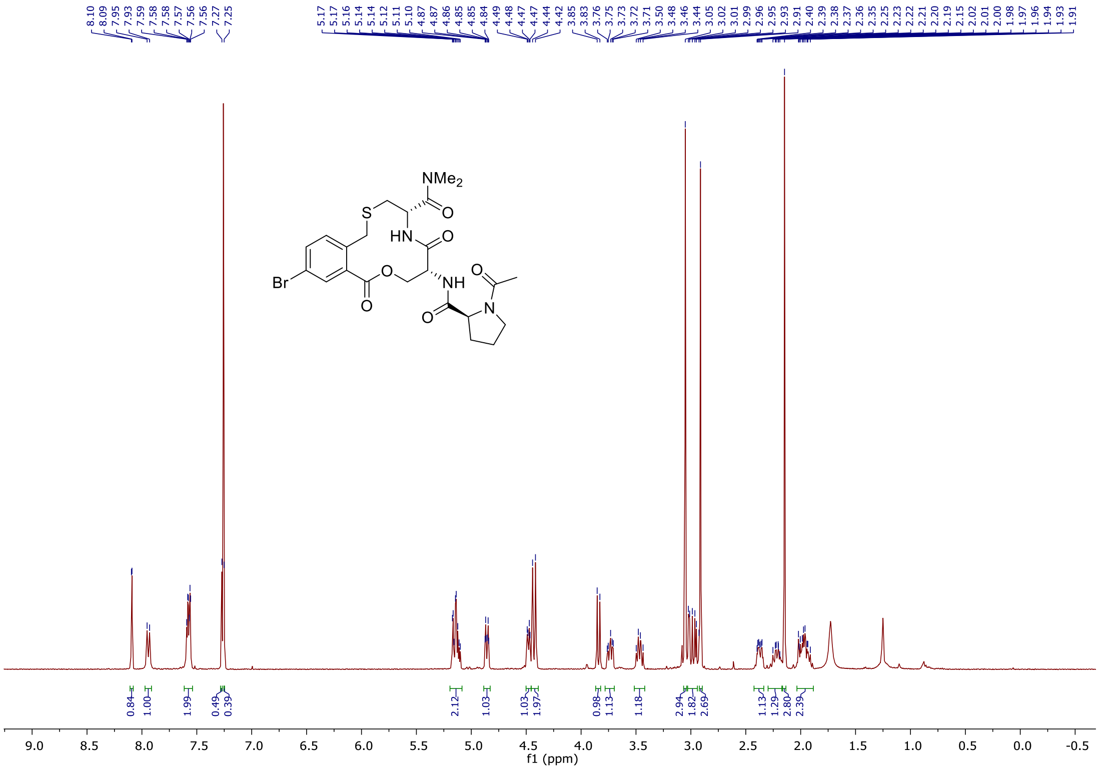

10 ^{13}C NMR (126 MHz, $CDCl_3$)

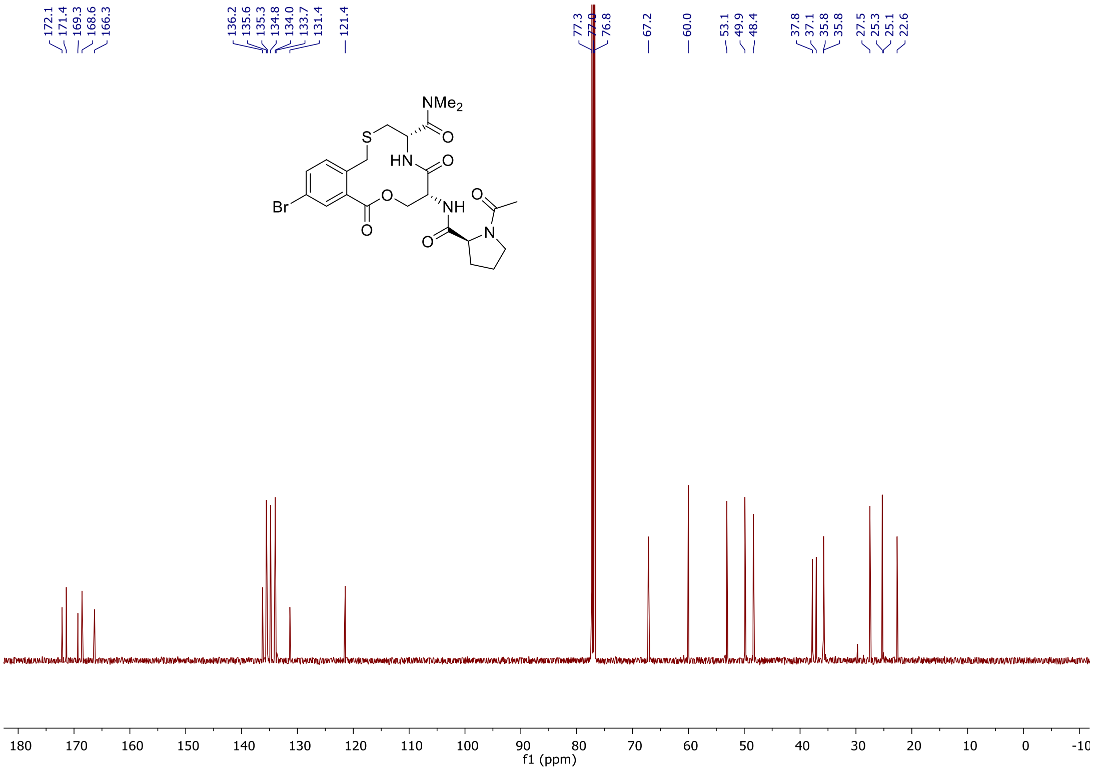

11 ^{1}H NMR (500 MHz, $CDCl_3$)

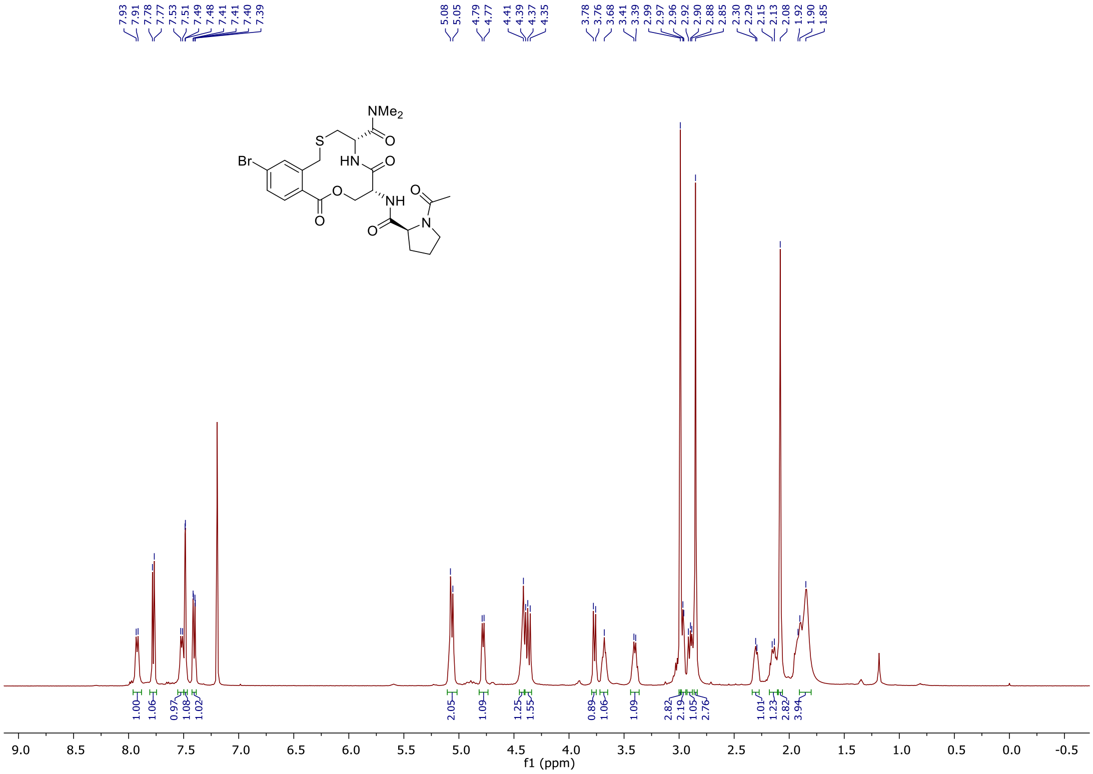

11 ^{13}C NMR (126 MHz, $CDCl_3$)

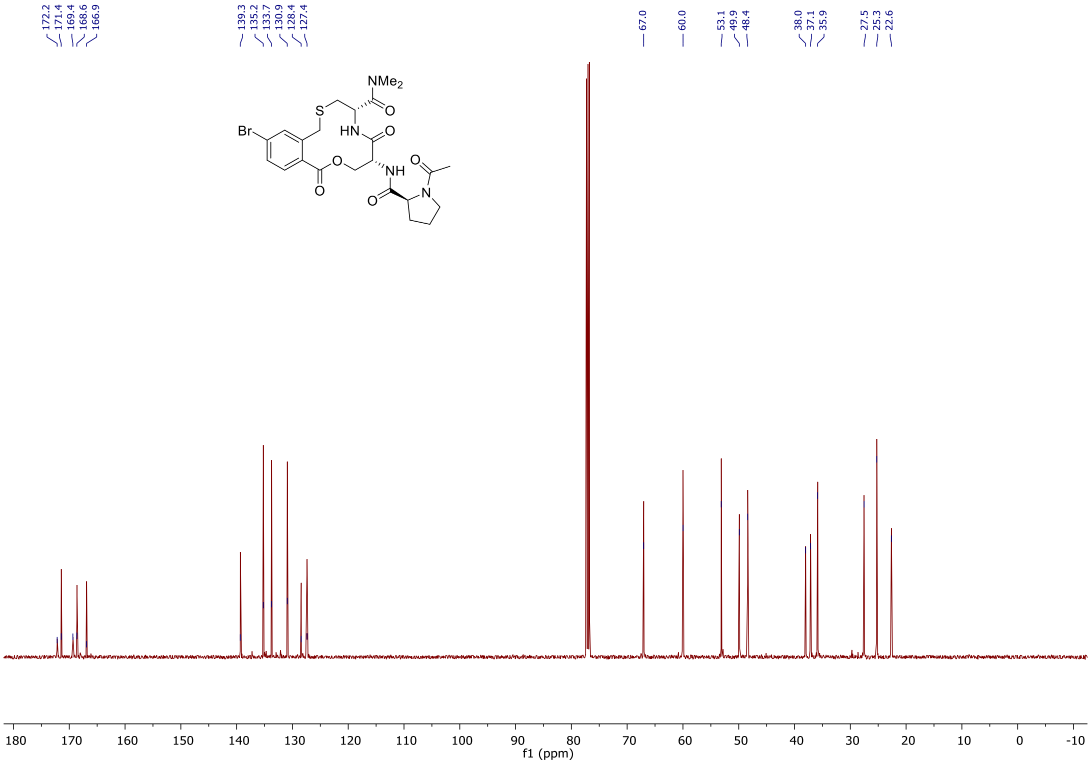

12 ^{1}H NMR (400 MHz, $CDCl_3$)

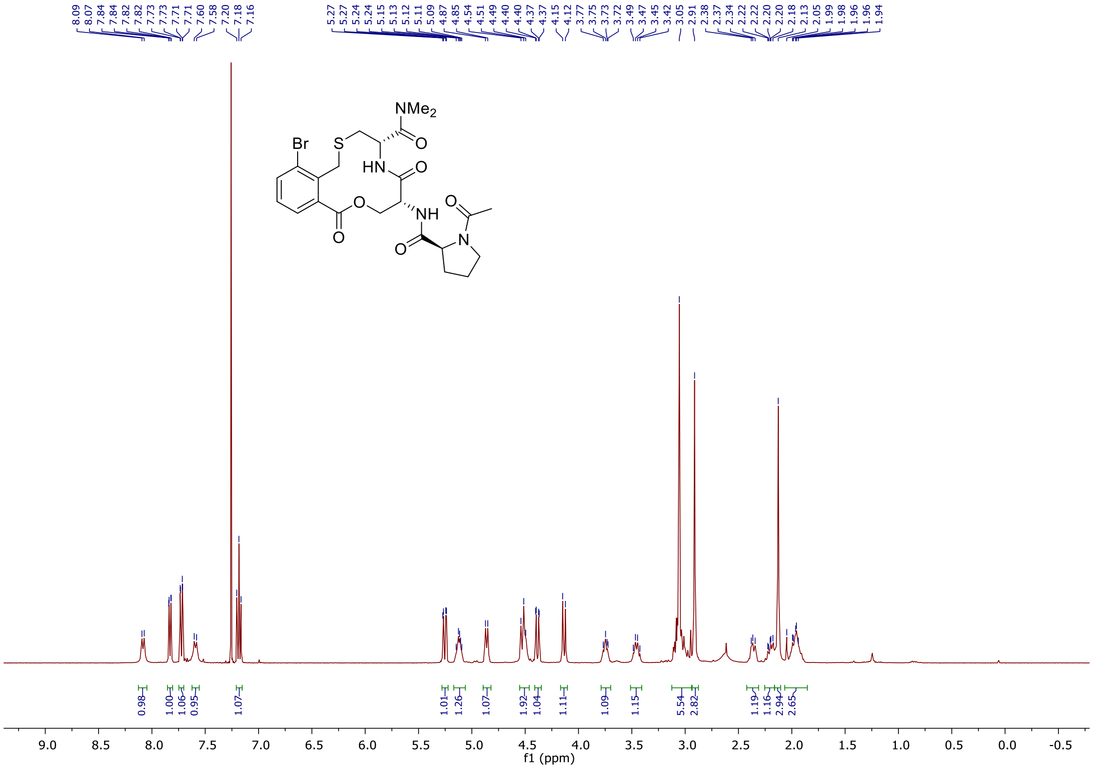

12 ^{13}C NMR (101 MHz, $CDCl_3$)

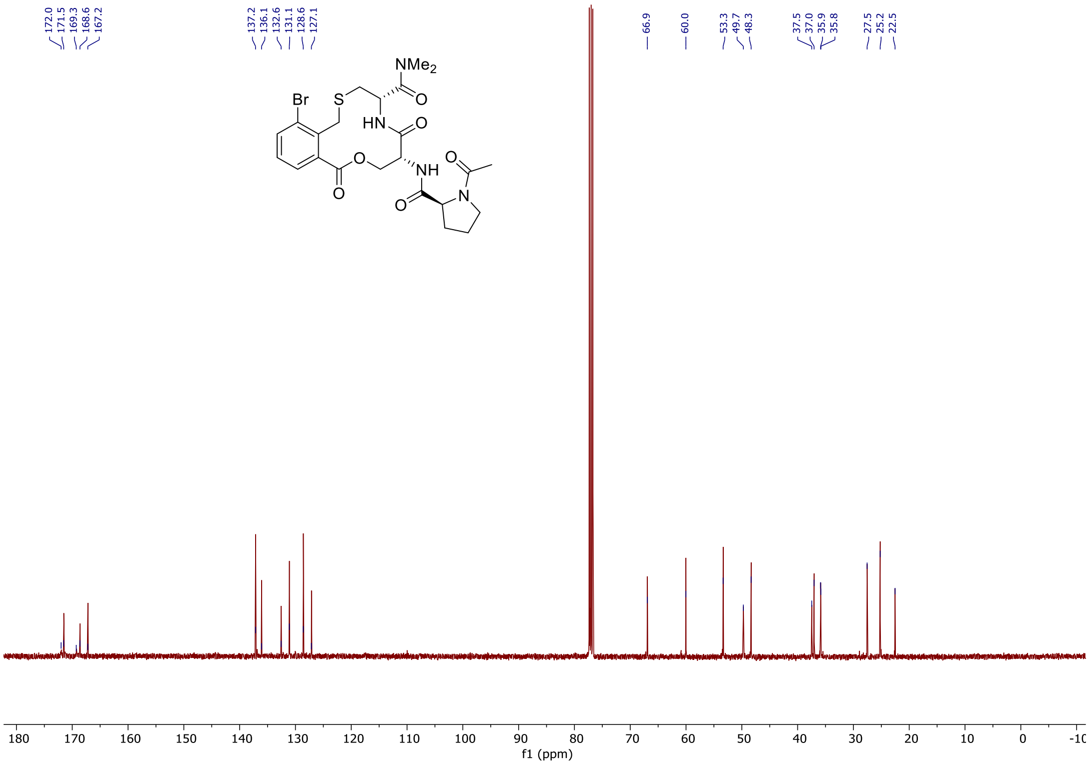

13 ^{1}H NMR (500 MHz, CDCl$_3$)

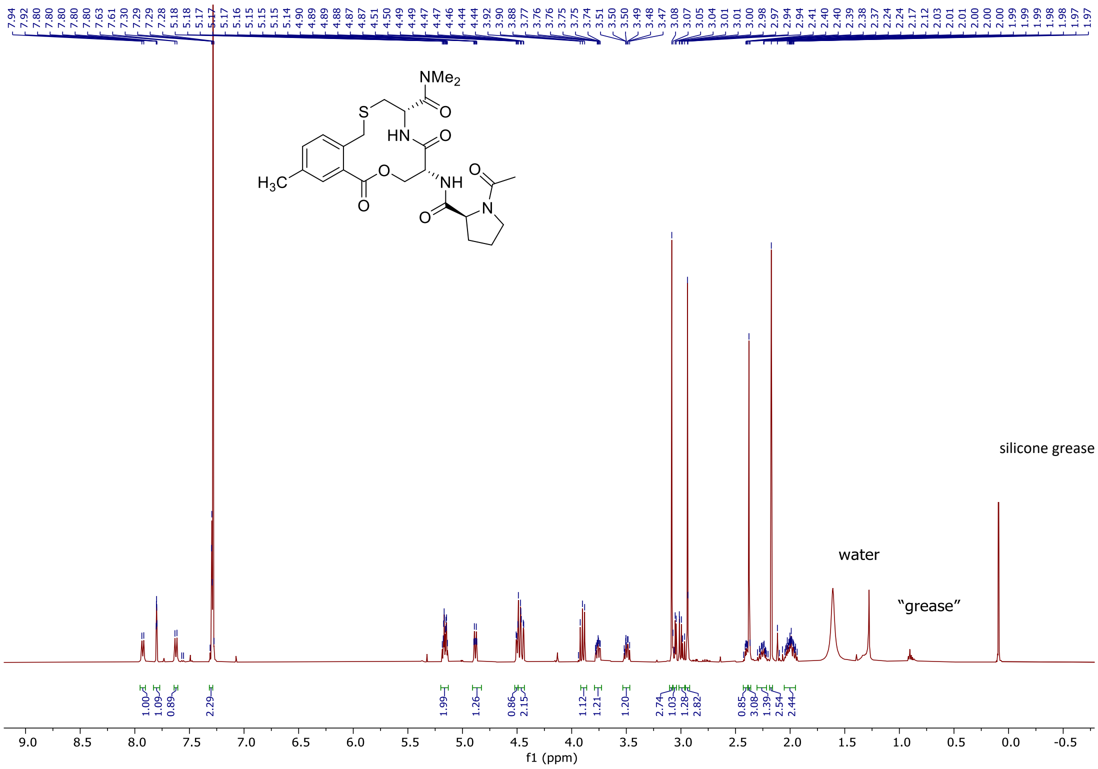

13 ^{13}C NMR (126 MHz, CDCl$_3$)

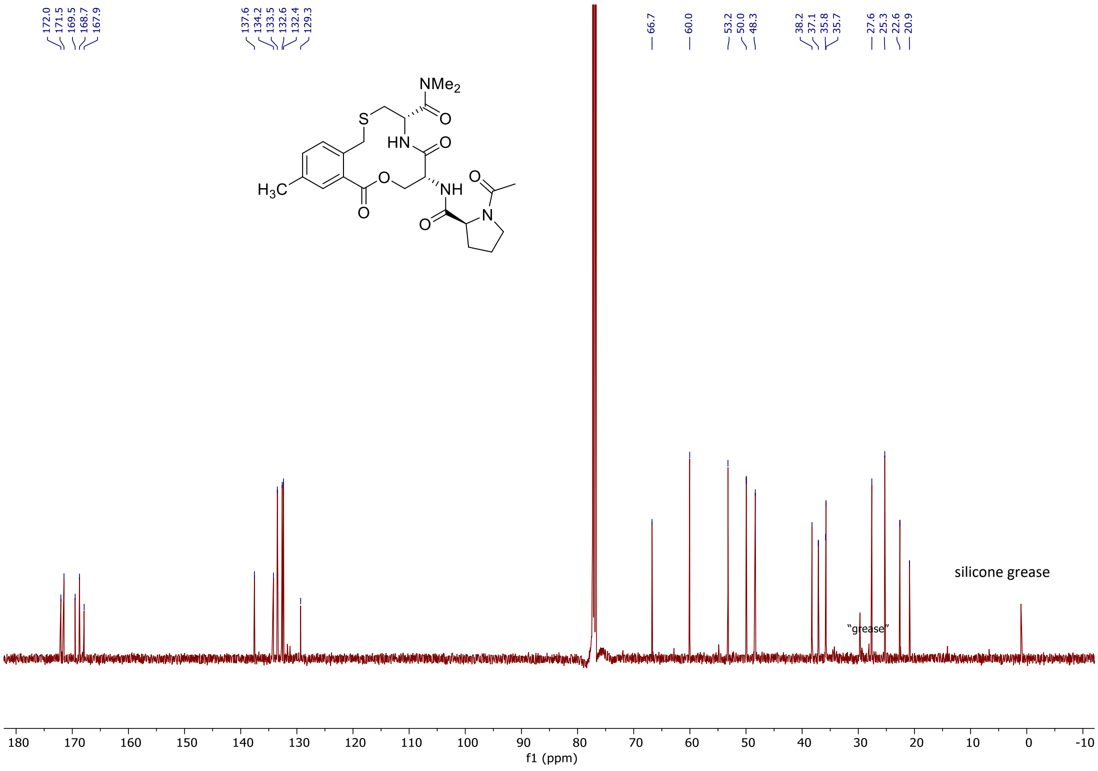

14 ^{1}H NMR (500 MHz, $CDCl_3$)

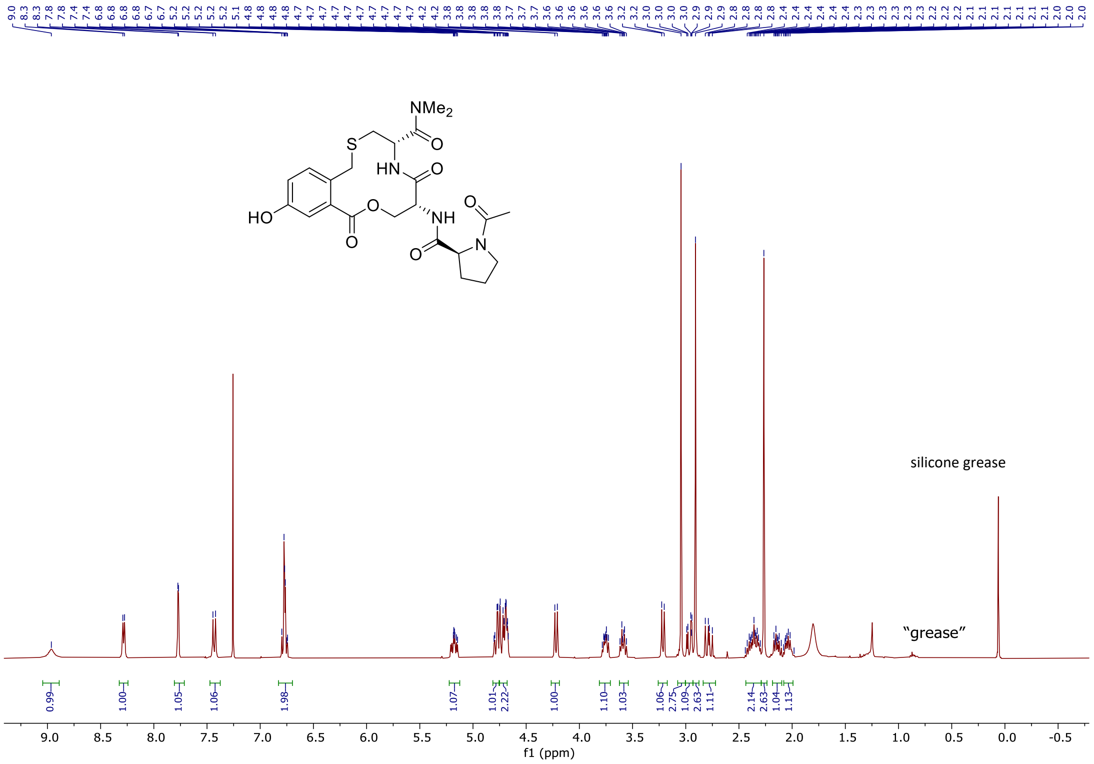

14 ^{13}C NMR (126 MHz, $CDCl_3$)

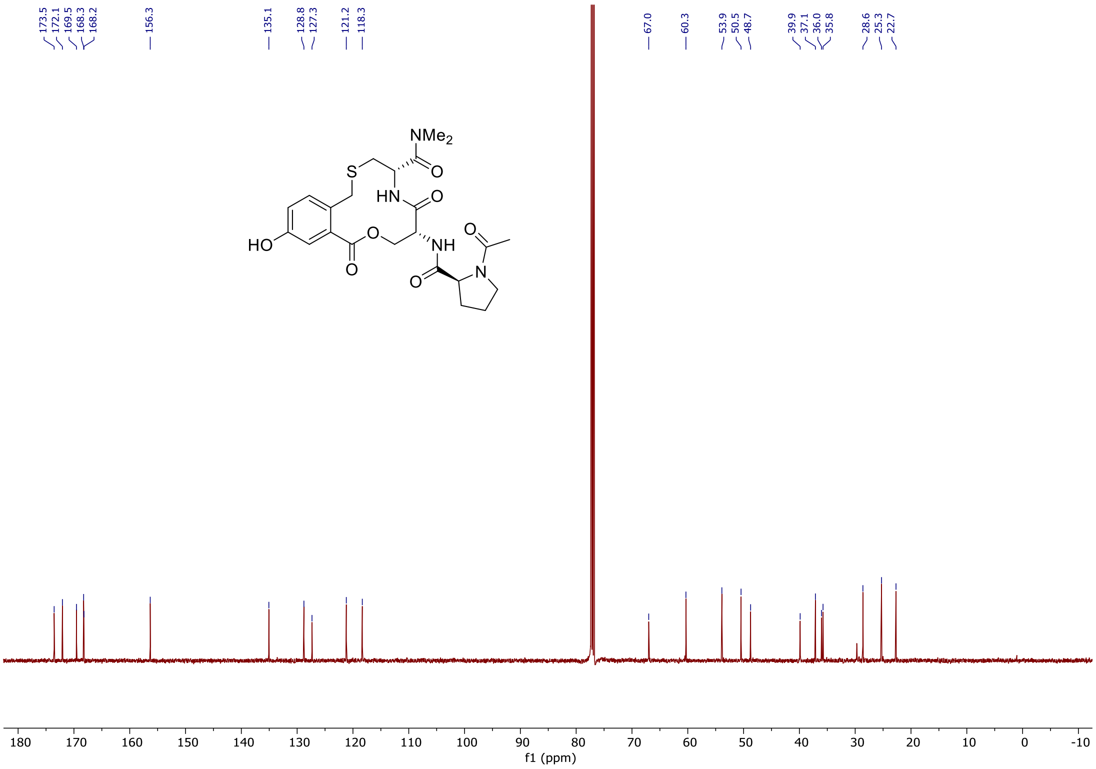

15 ^{1}H NMR (500 MHz, $CDCl_3$)

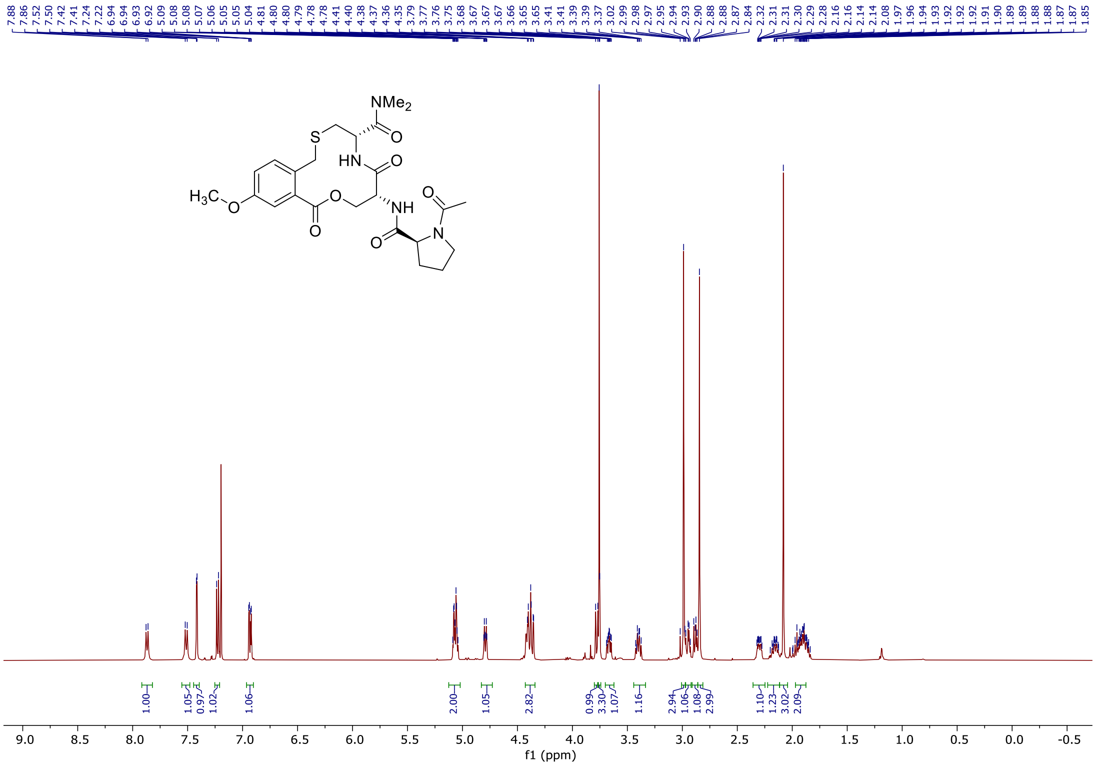

15 ^{13}C NMR (500 MHz, $CDCl_3$)

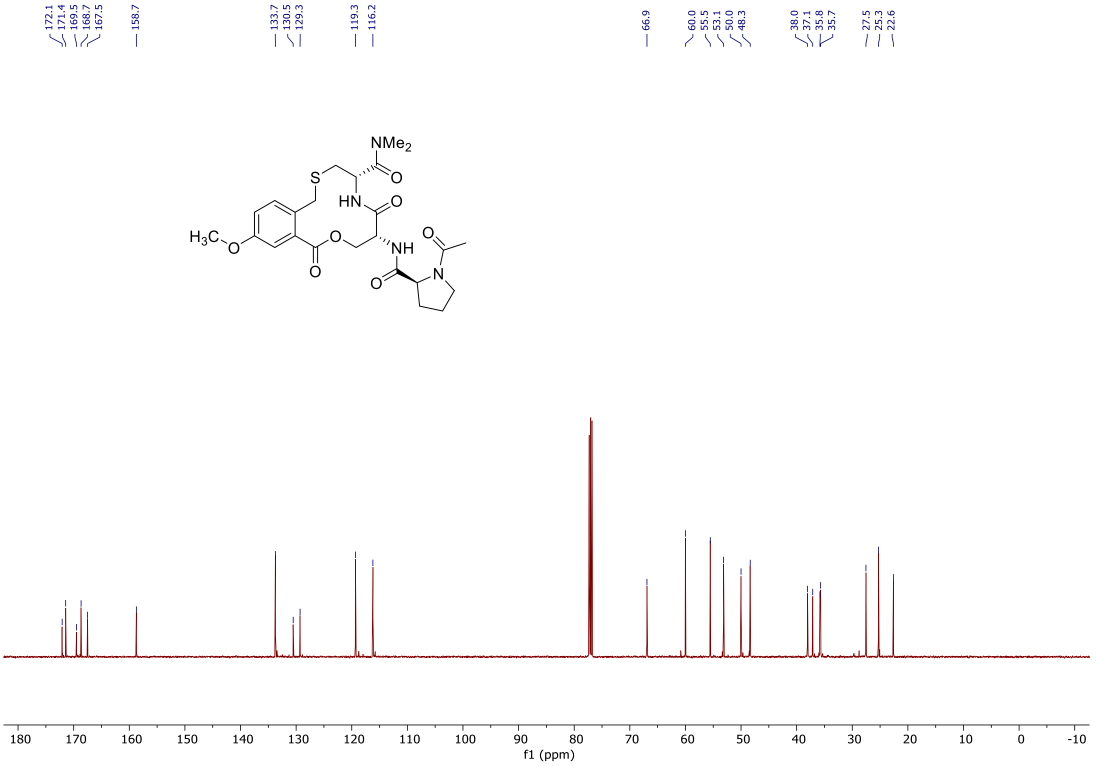

16 ^{1}H NMR (500 MHz, $CDCl_3$)

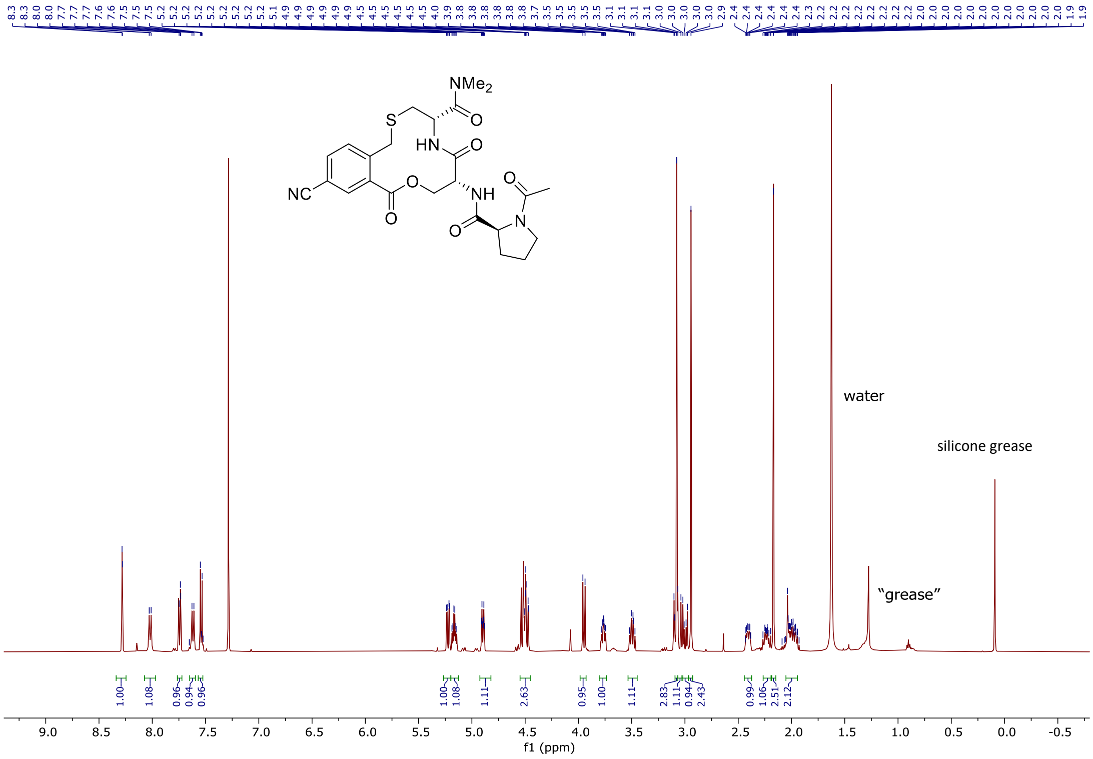

16 ^{13}C NMR (126 MHz, $CDCl_3$)

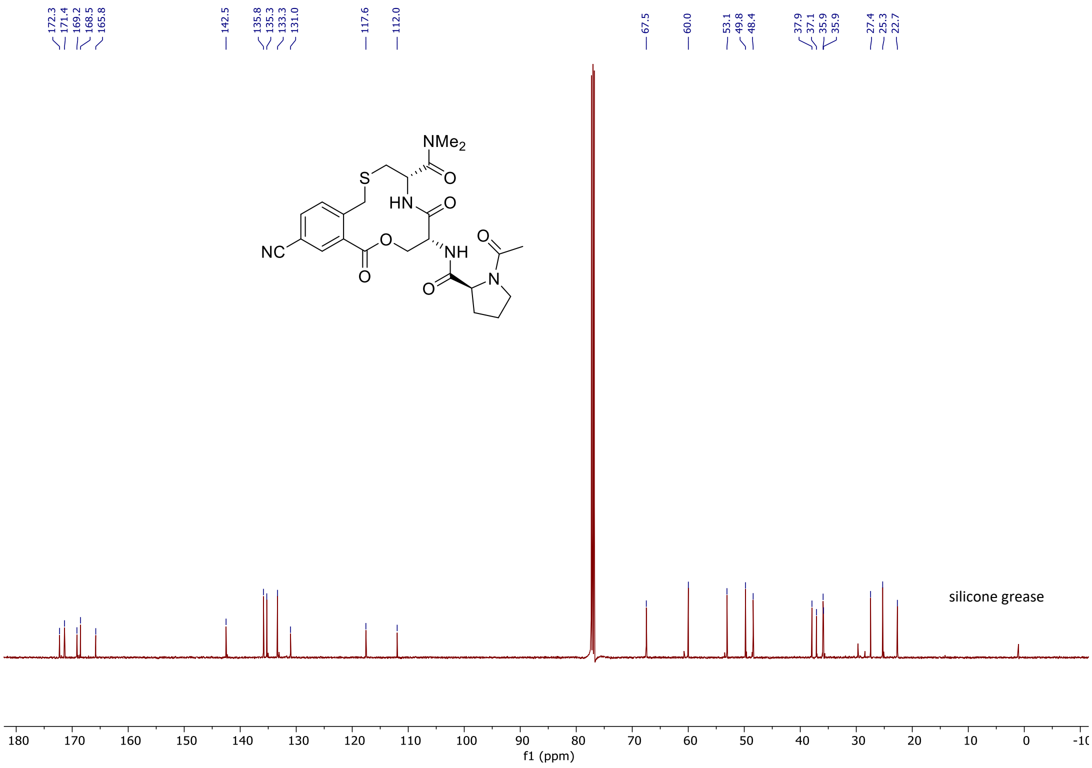

17 ^{1}H NMR (500 MHz, CDCl$_3$)

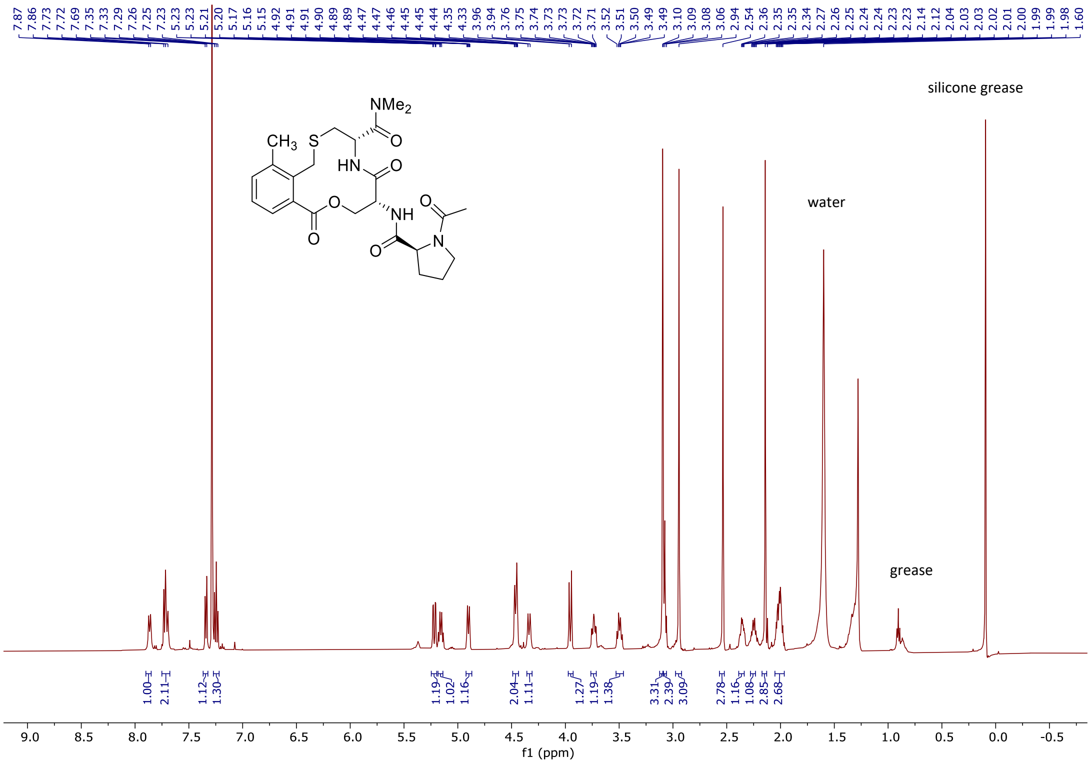

17 ^{13}C NMR (126 MHz, CDCl$_3$)

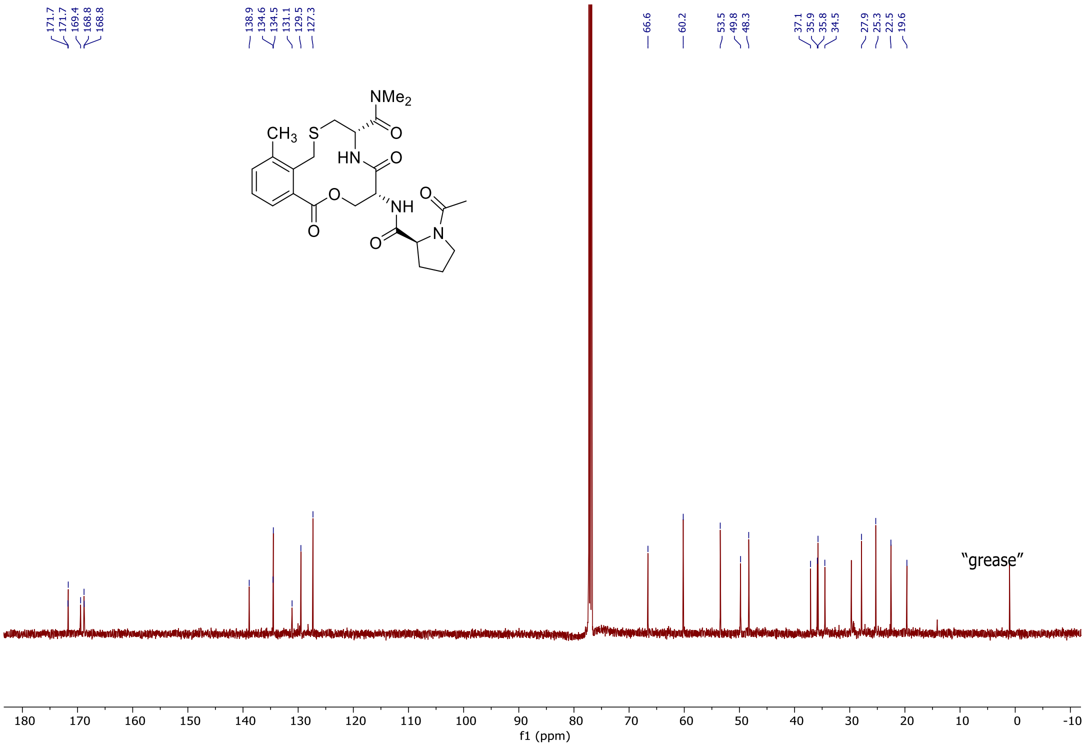

18 ^{1}H NMR (500 MHz, $CDCl_3$)

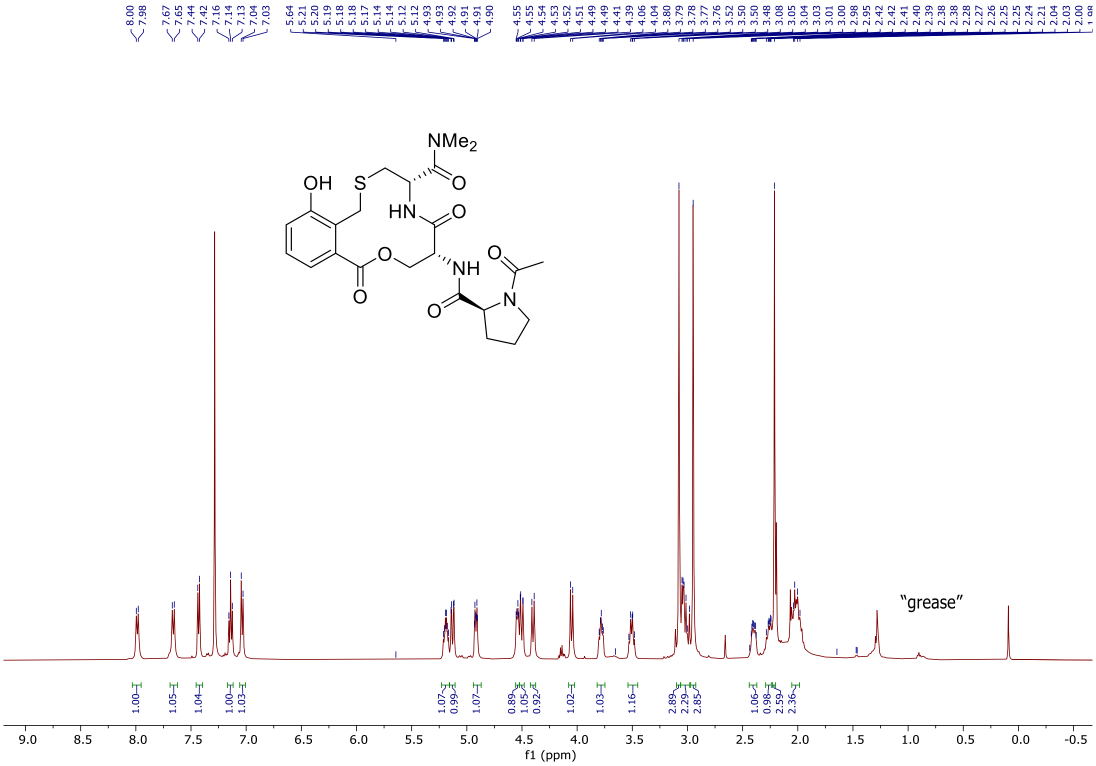

18 ^{13}C NMR (126 MHz, $CDCl_3$)

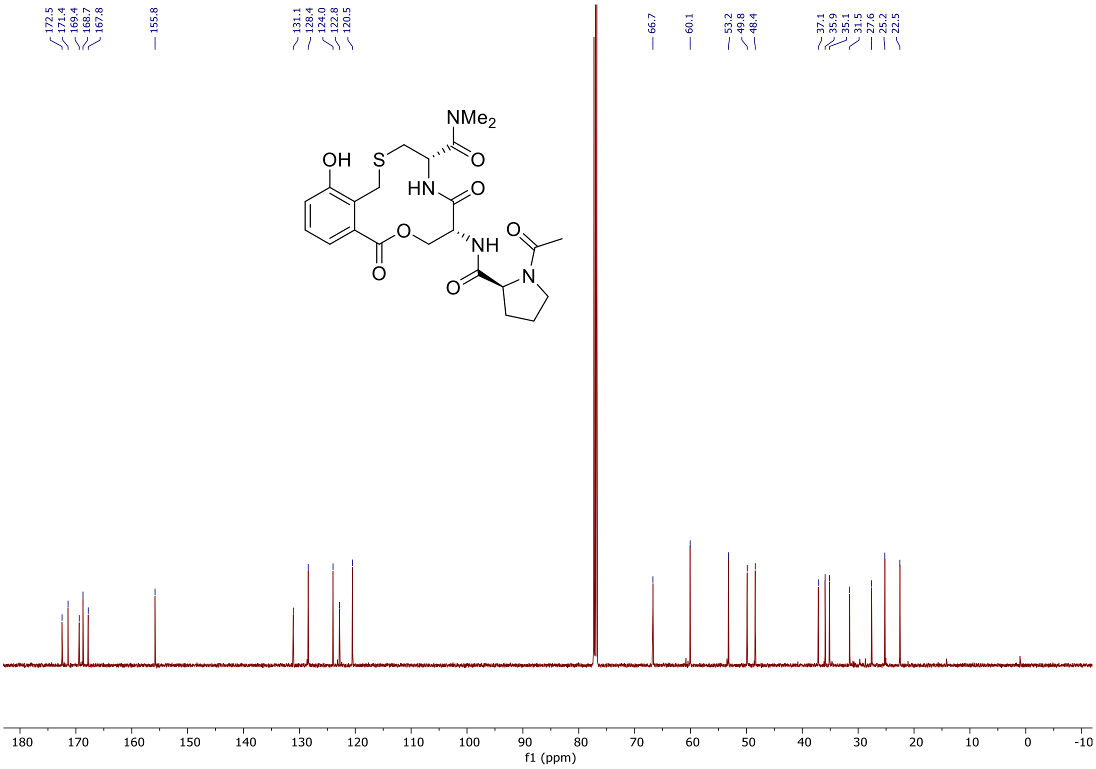

19 ^{1}H NMR (500 MHz, $CDCl_3$)

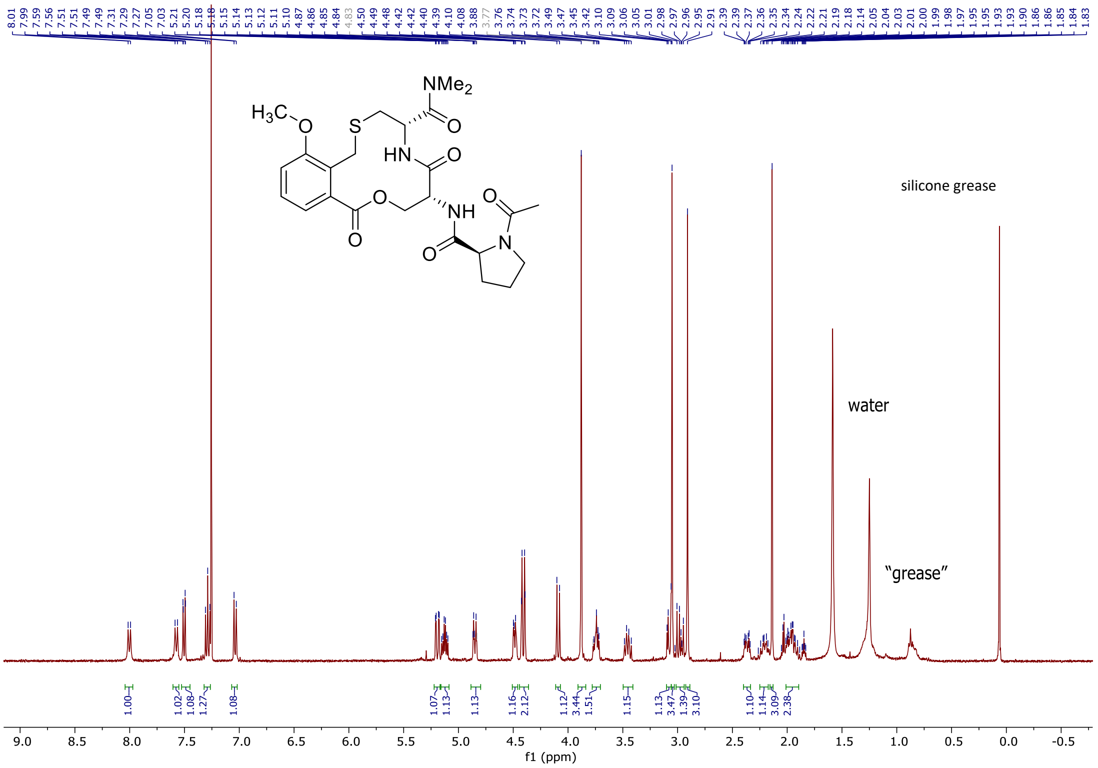

19 ^{13}C NMR (126 MHz, $CDCl_3$)

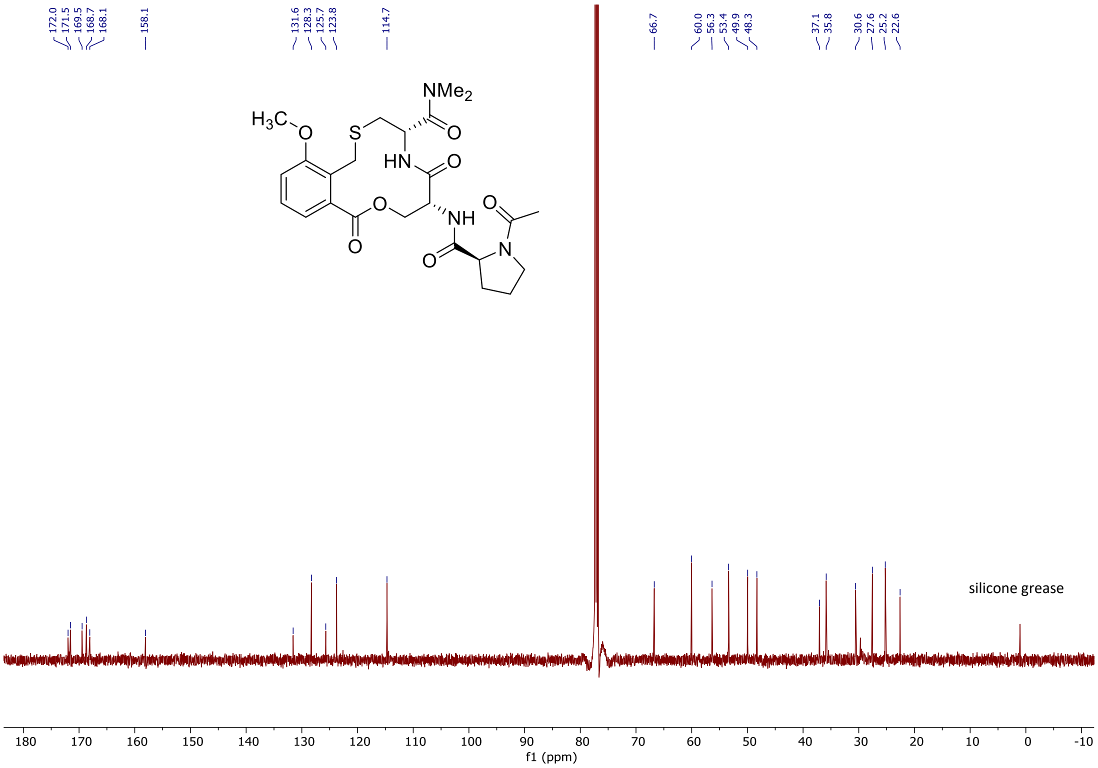

20 ^{1}H NMR (500 MHz, $CDCl_3$)

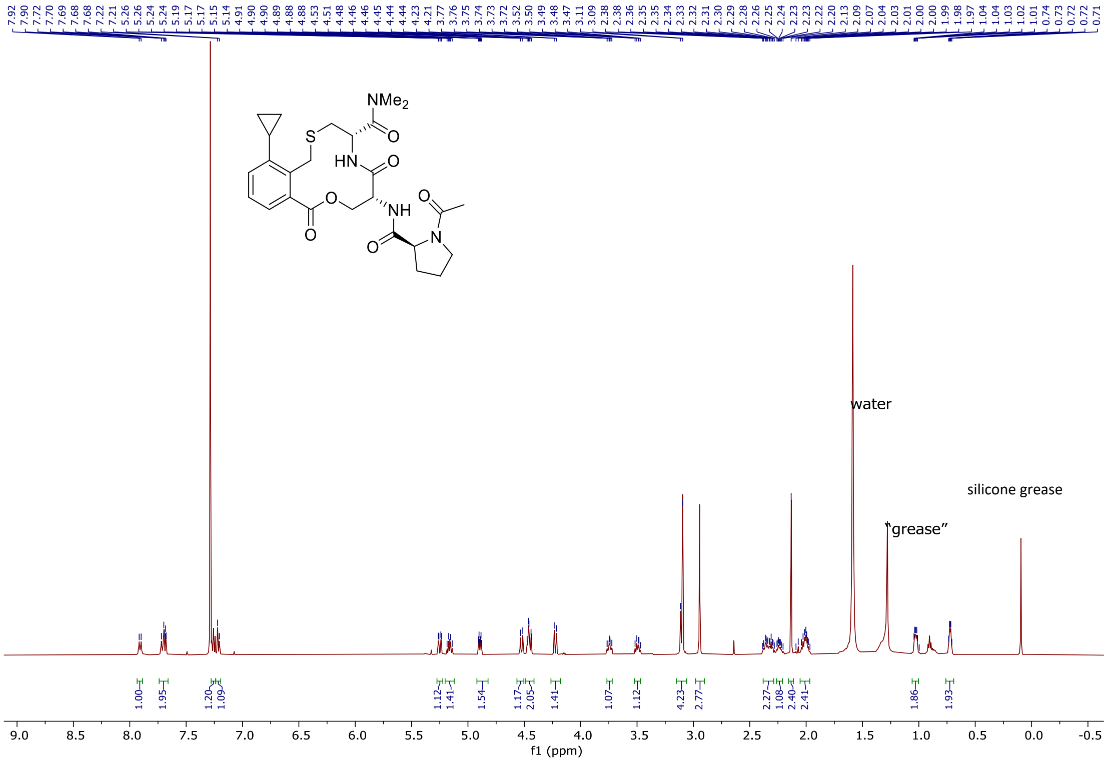

20 ^{13}C NMR (126 MHz, $CDCl_3$)

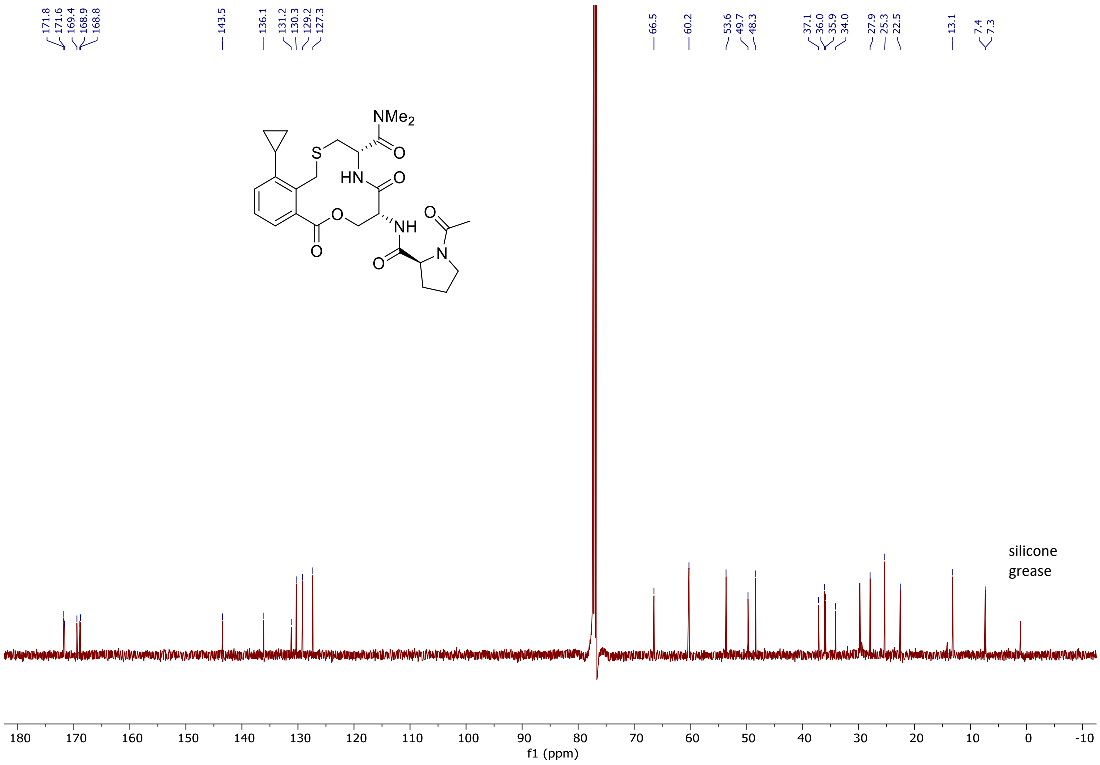

21 ^{1}H NMR (500 MHz, $CDCl_3$)

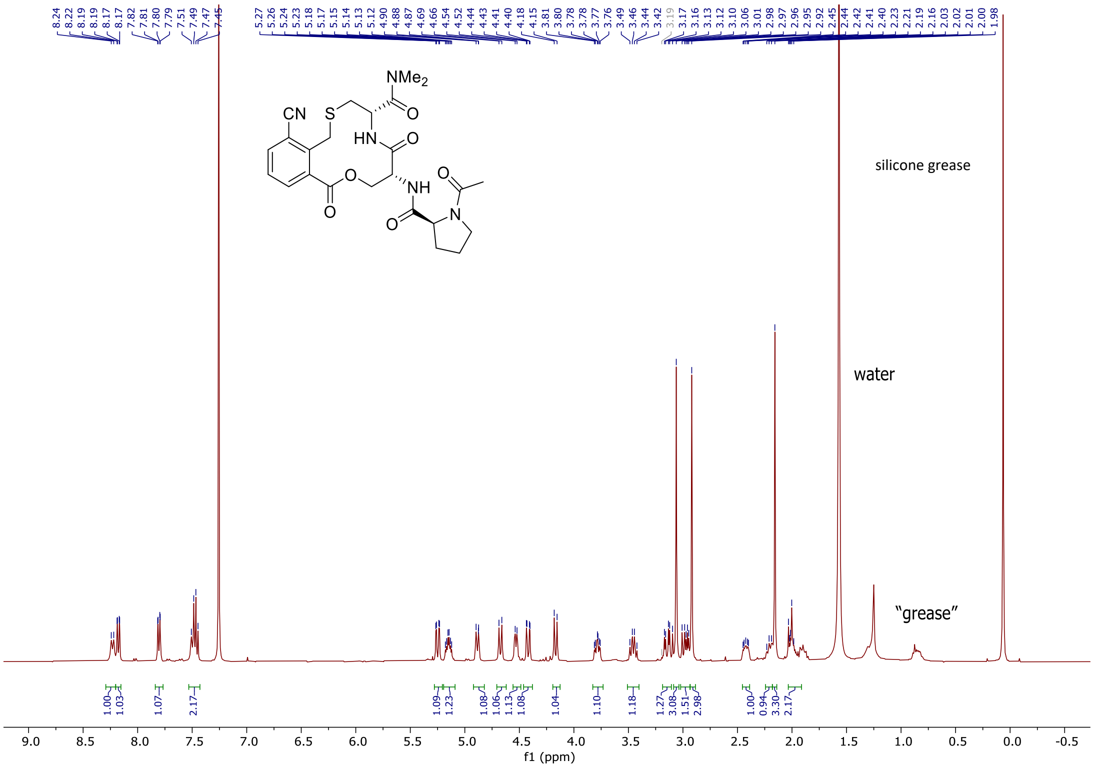

21 ^{13}C NMR (126 MHz, $CDCl_3$)

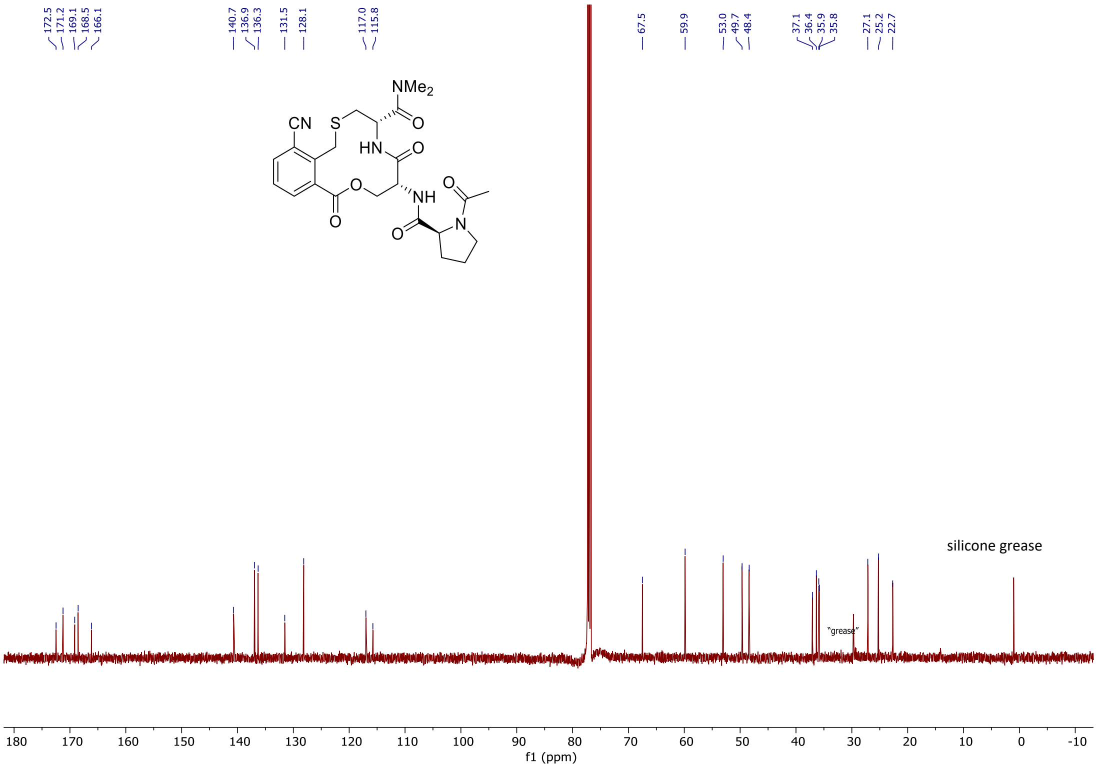

22 ^{1}H NMR (500 MHz, CDCl$_3$)

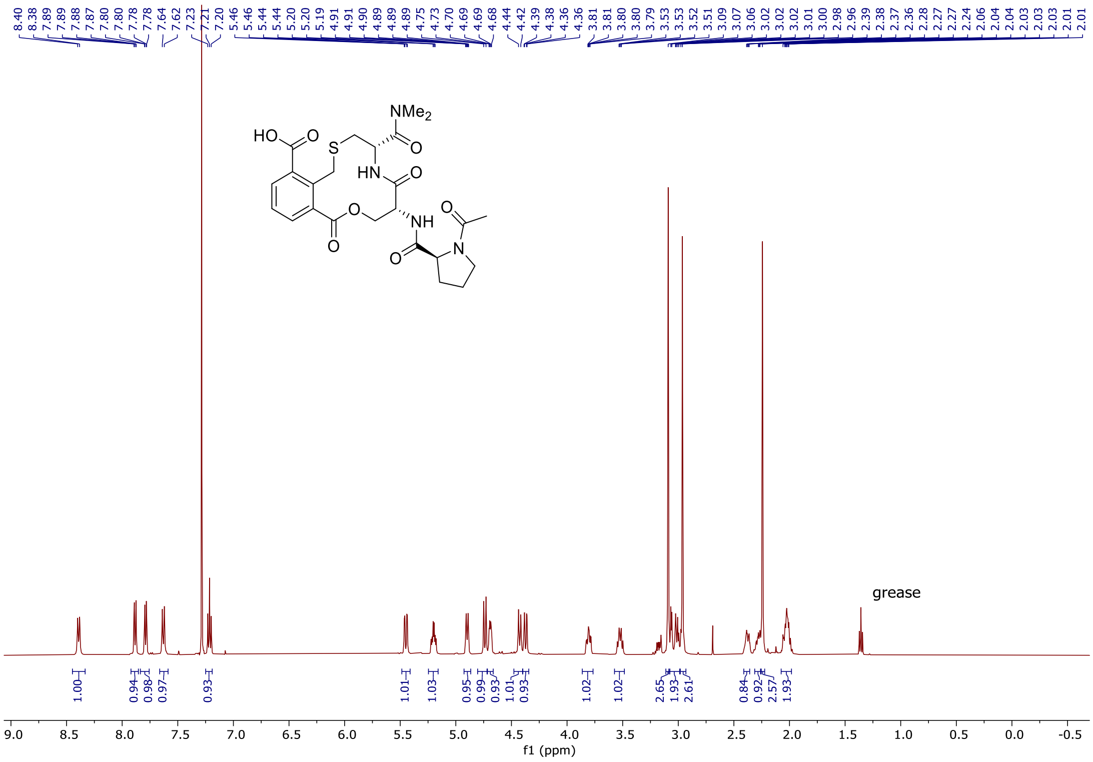

22 ^{13}C NMR (126 MHz, CDCl$_3$)

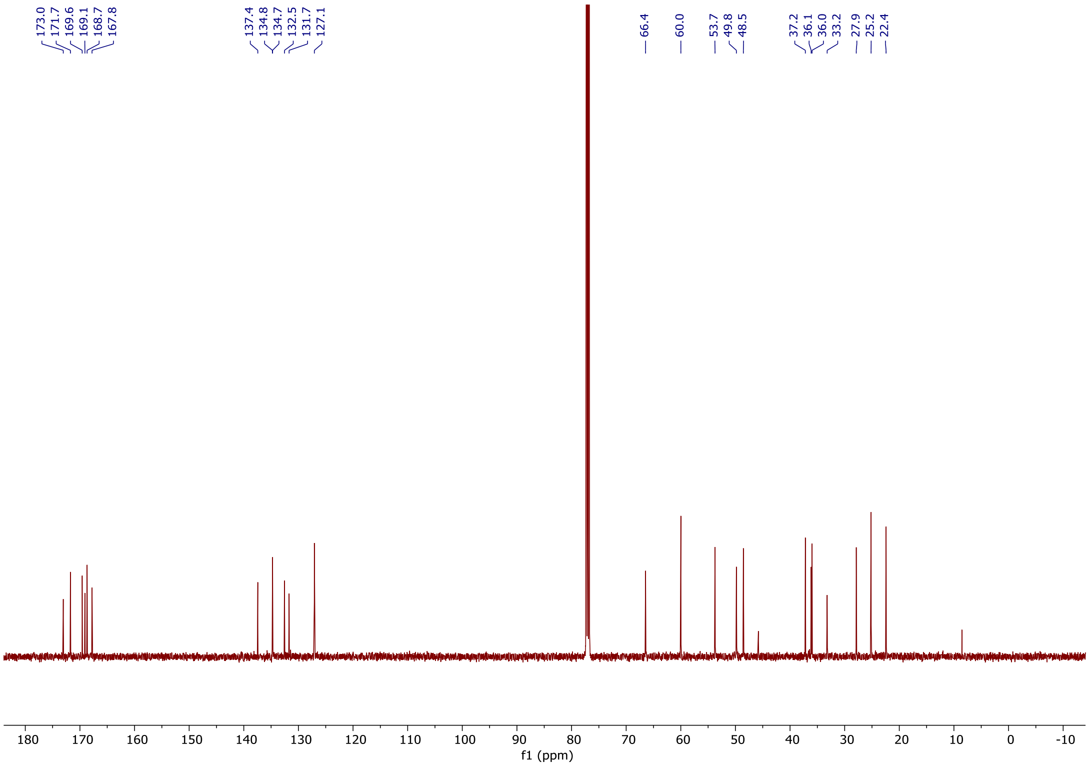

23 ^{1}H NMR (500 MHz, $CDCl_3$)

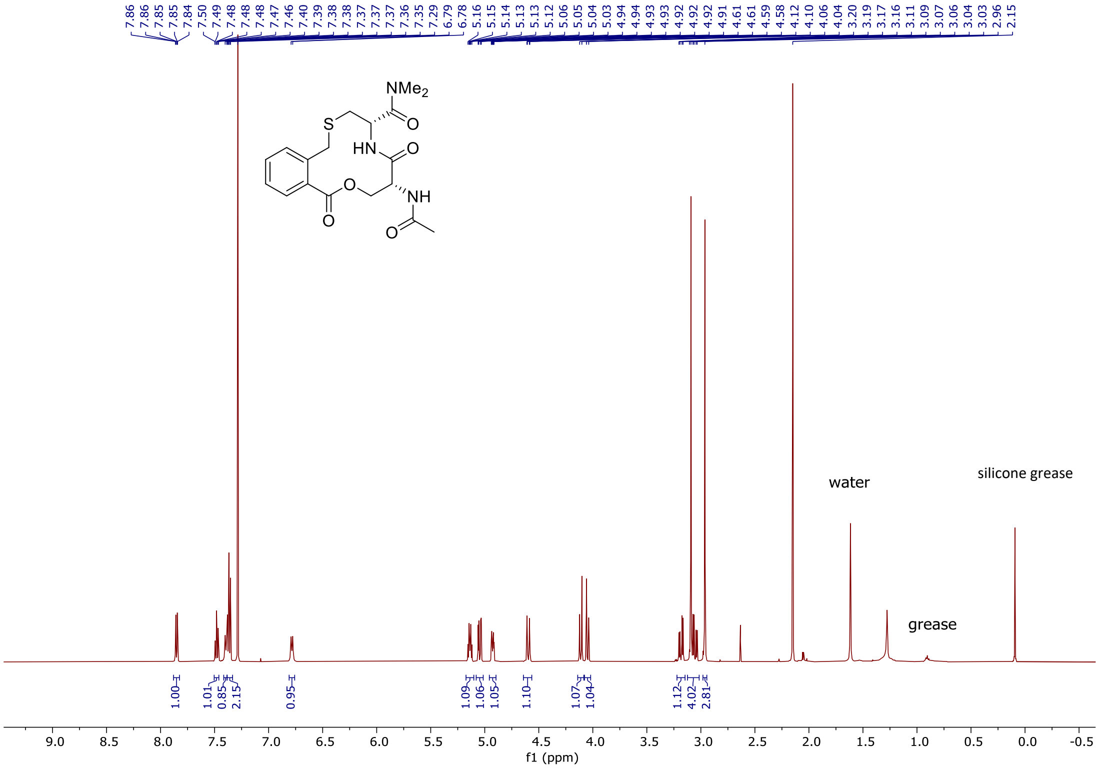

23 ^{13}C NMR (126 MHz, $CDCl_3$)

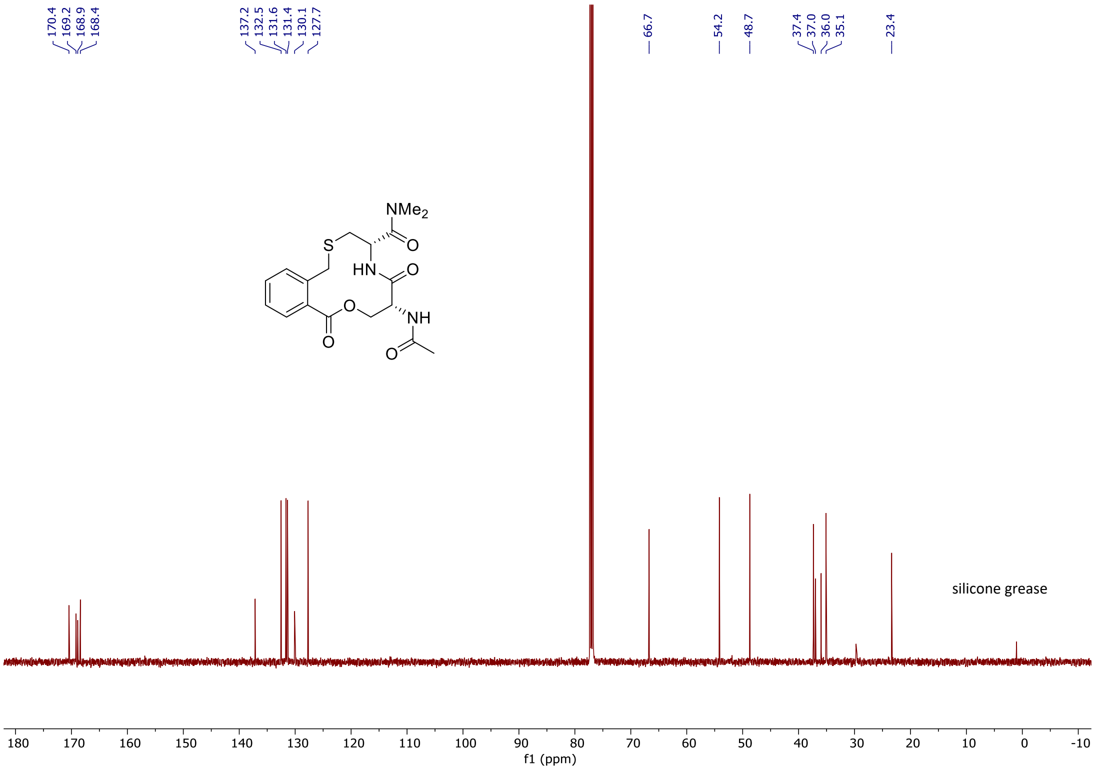

24 ^{1}H NMR (400 MHz, DMSO-d_6)

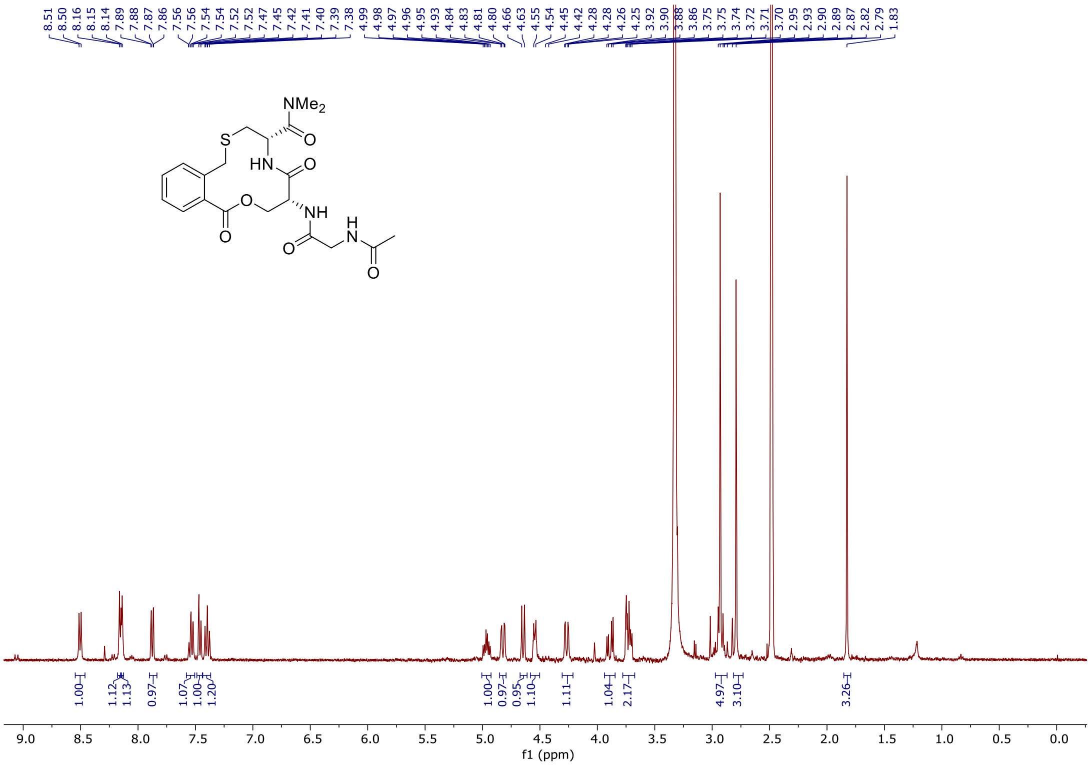

24 ^{13}C NMR (101 MHz, DMSO-d_6)

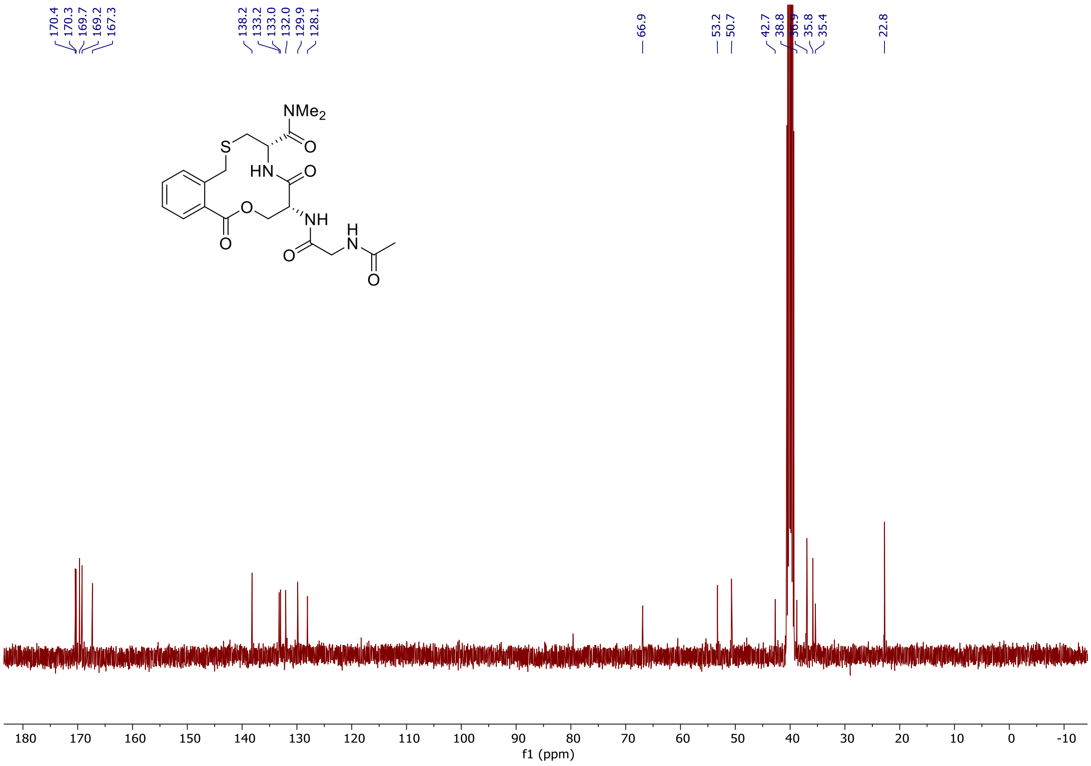

25 ^{1}H NMR (500 MHz, $CDCl_3$)

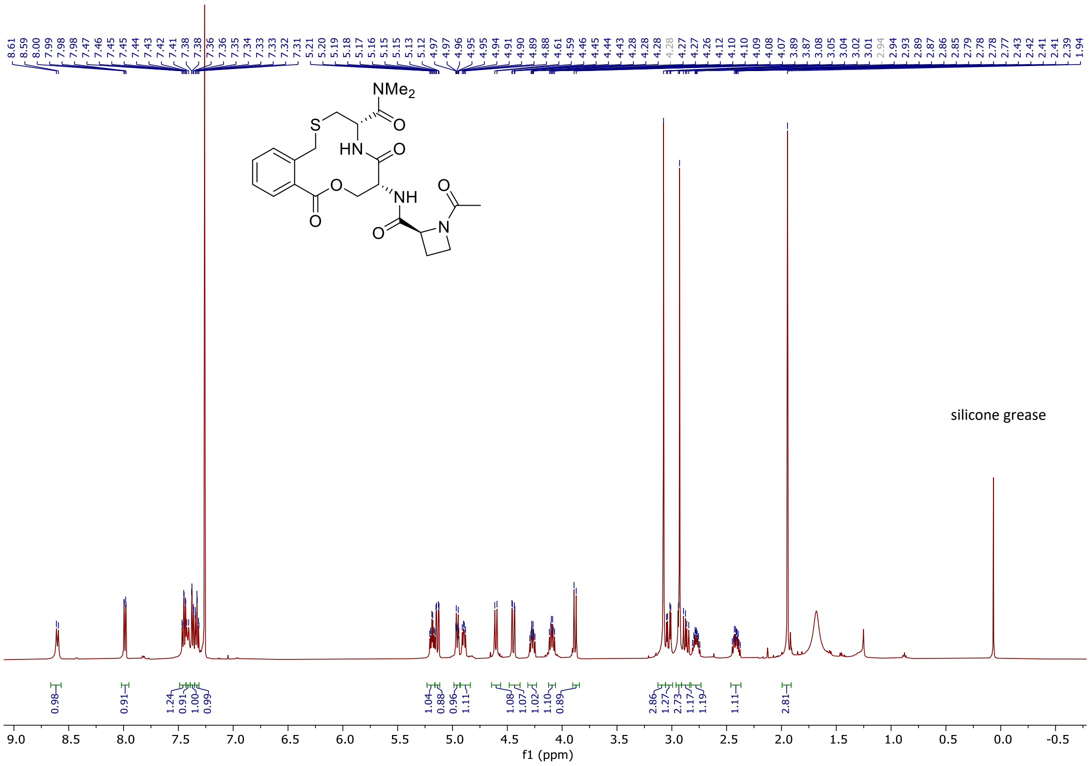

25 ^{13}C NMR (126 MHz, $CDCl_3$)

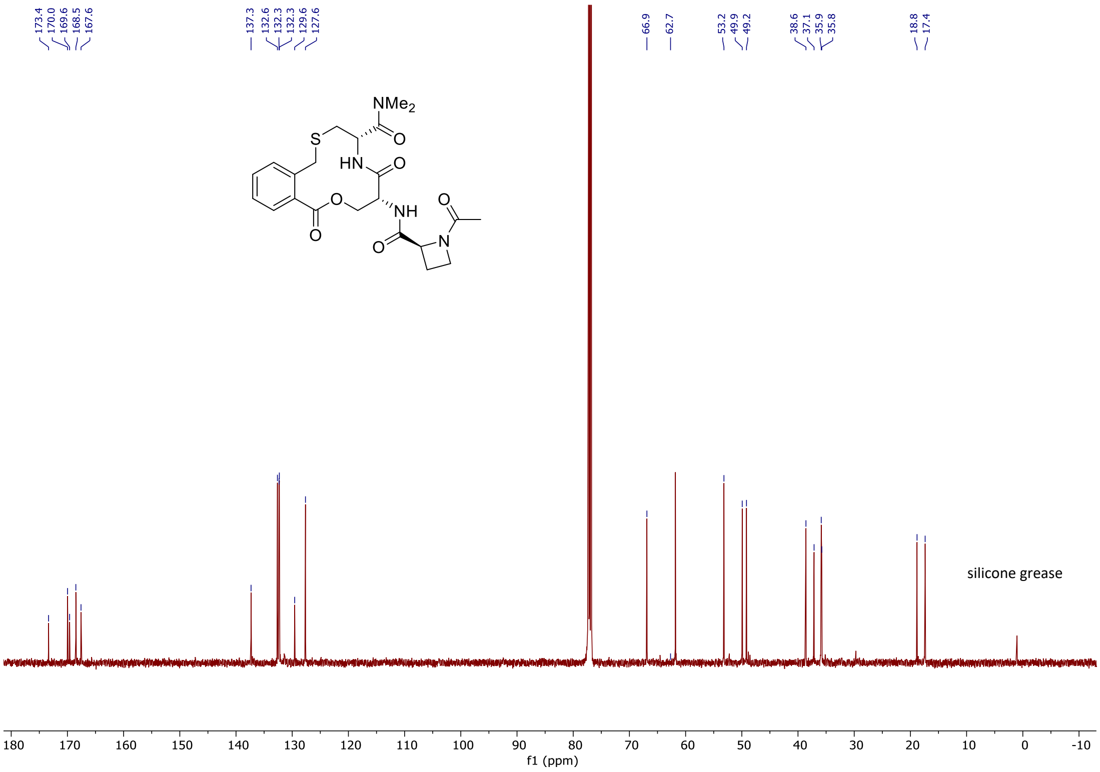

26 ^{1}H NMR (500 MHz, CDCl$_3$)

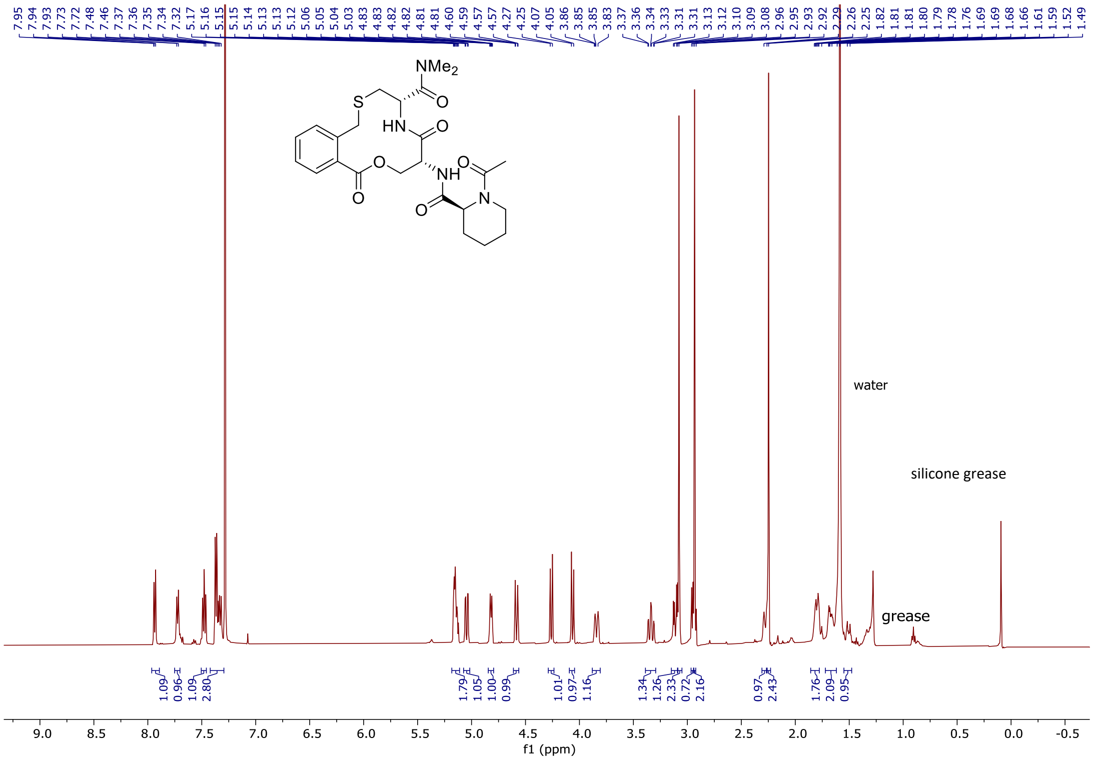

26 ^{13}C NMR (126 MHz, CDCl$_3$)

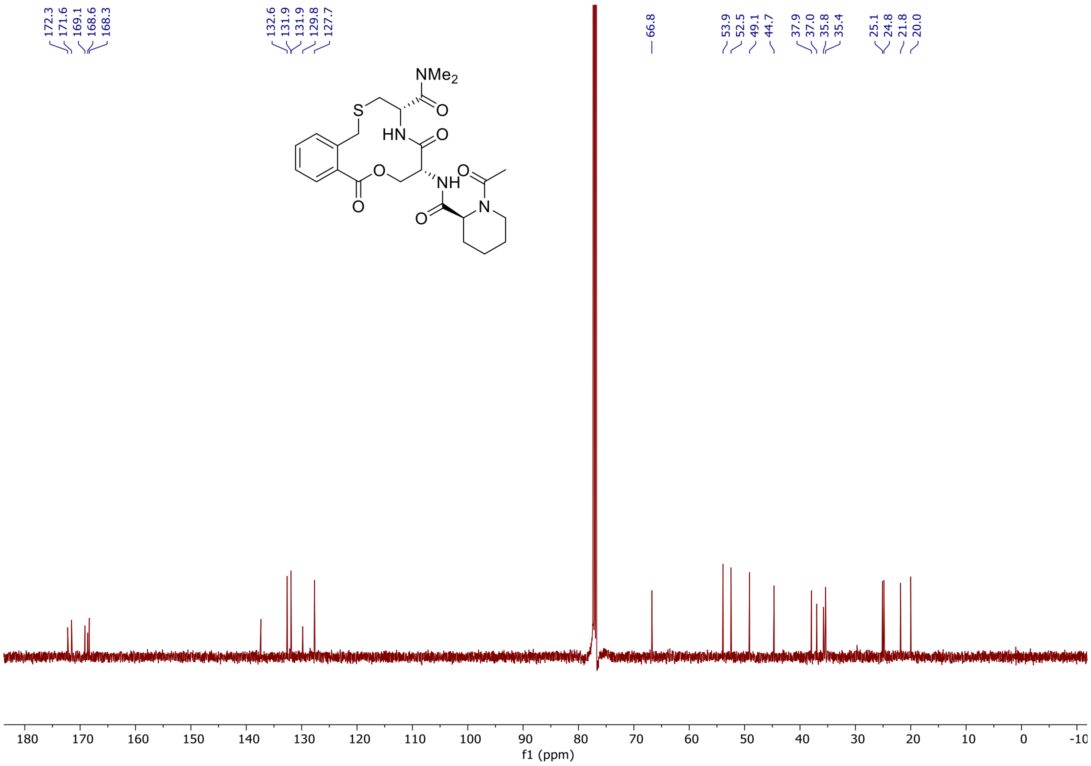

27 ^{1}H NMR (500 MHz, CDCl$_3$)

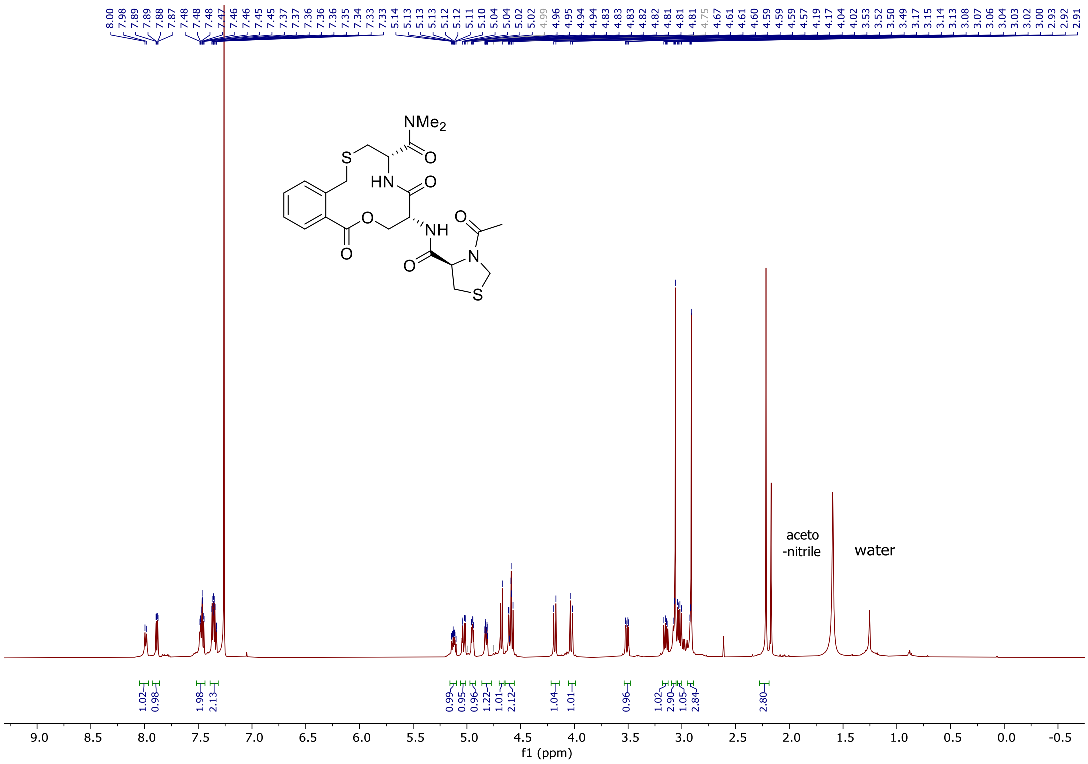

27 ^{13}C NMR (126 MHz, CDCl$_3$)

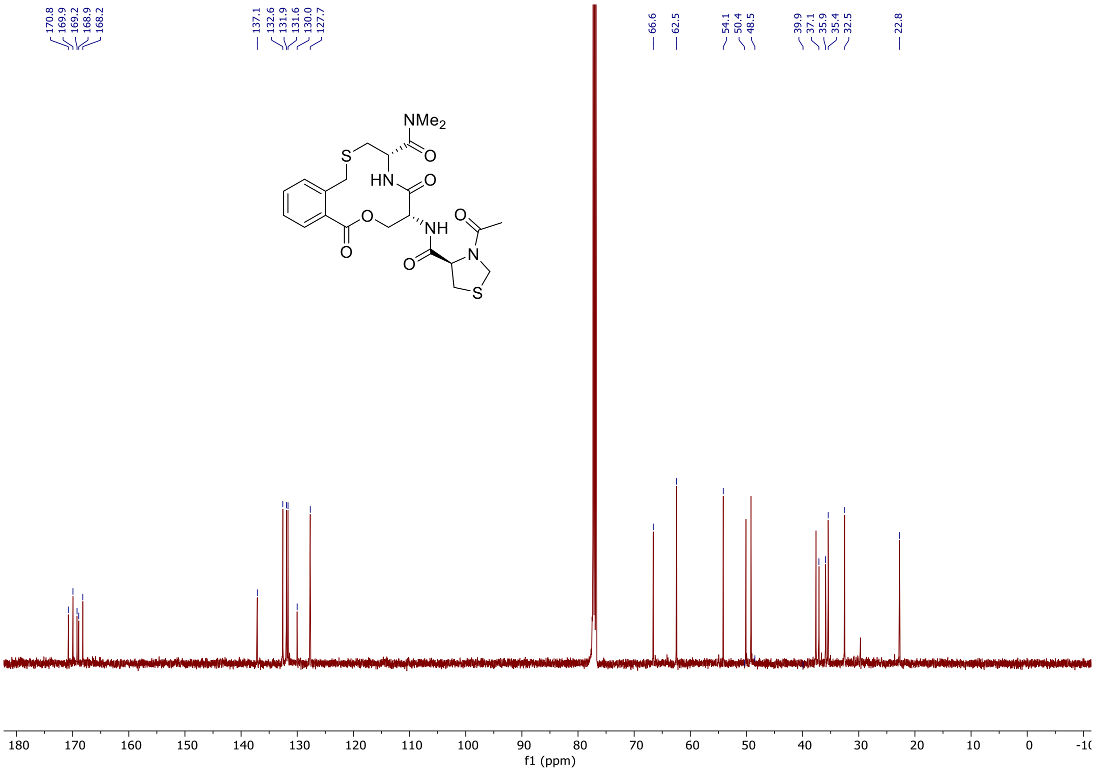

28 ^{1}H NMR (500 MHz, $CDCl_3$)

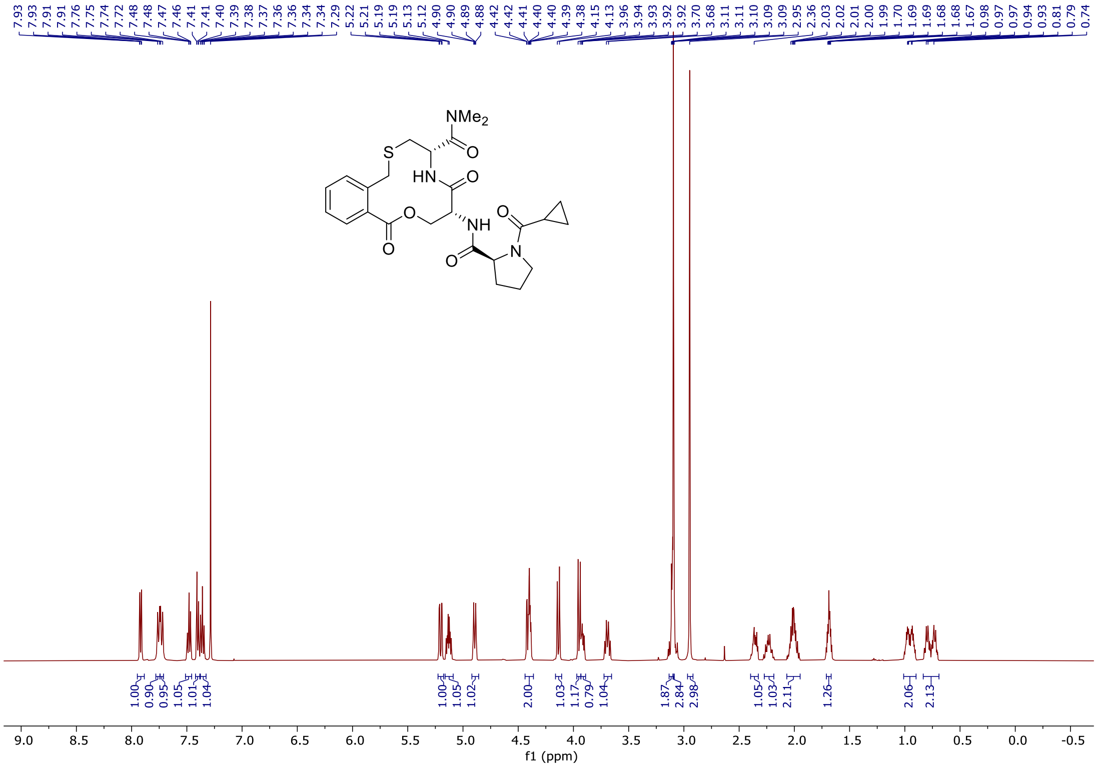

28 ^{13}C NMR (126 MHz, $CDCl_3$)

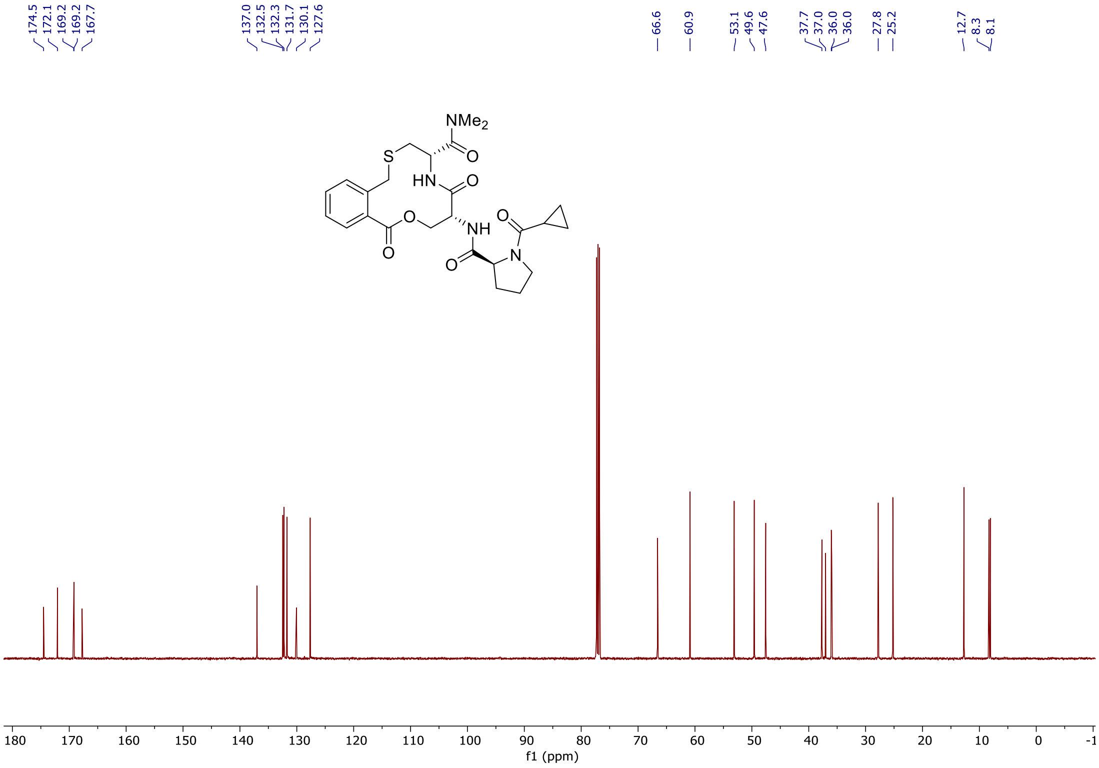

29 ^{1}H NMR (500 MHz, $CDCl_3$)

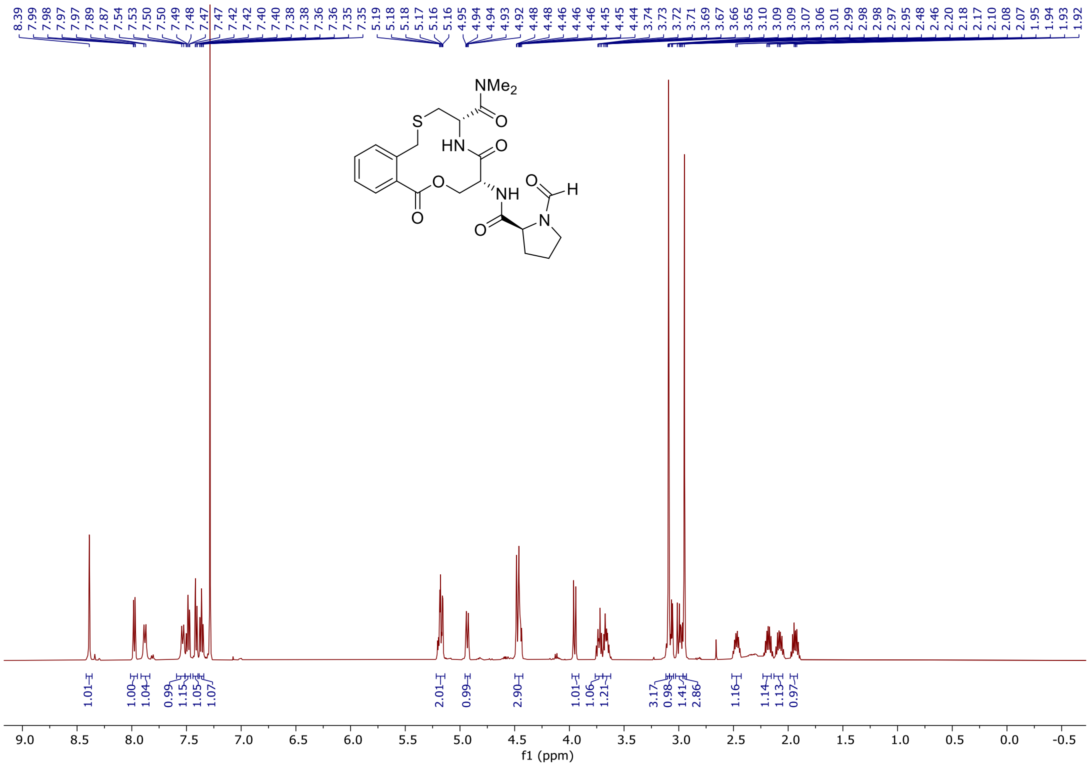

29 ^{13}C NMR (126 MHz, $CDCl_3$)

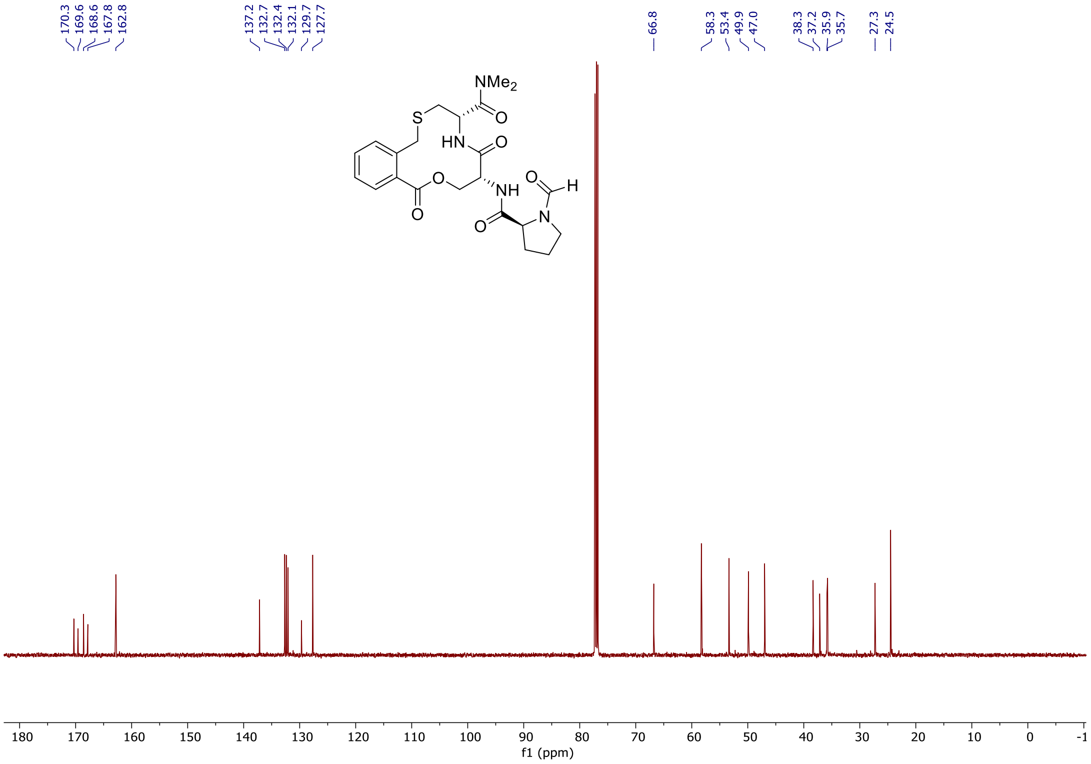

30 ^{1}H NMR (500 MHz, $CDCl_3$)

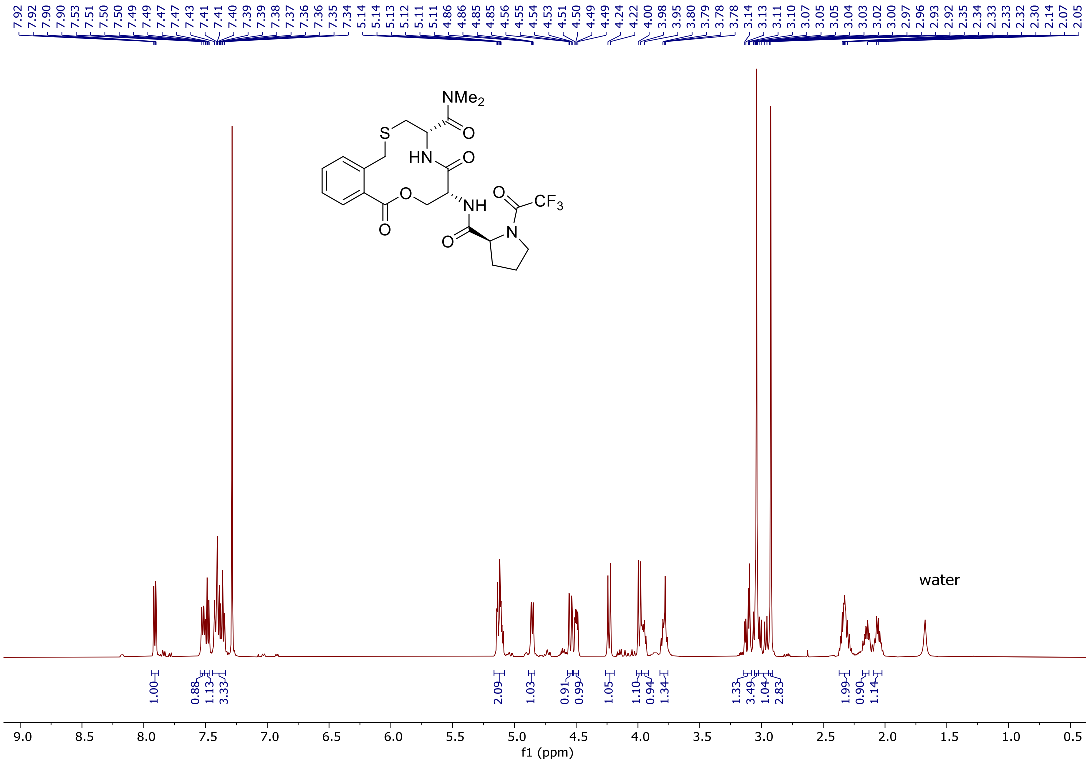

30 ^{13}C NMR (126 MHz, $CDCl_3$)

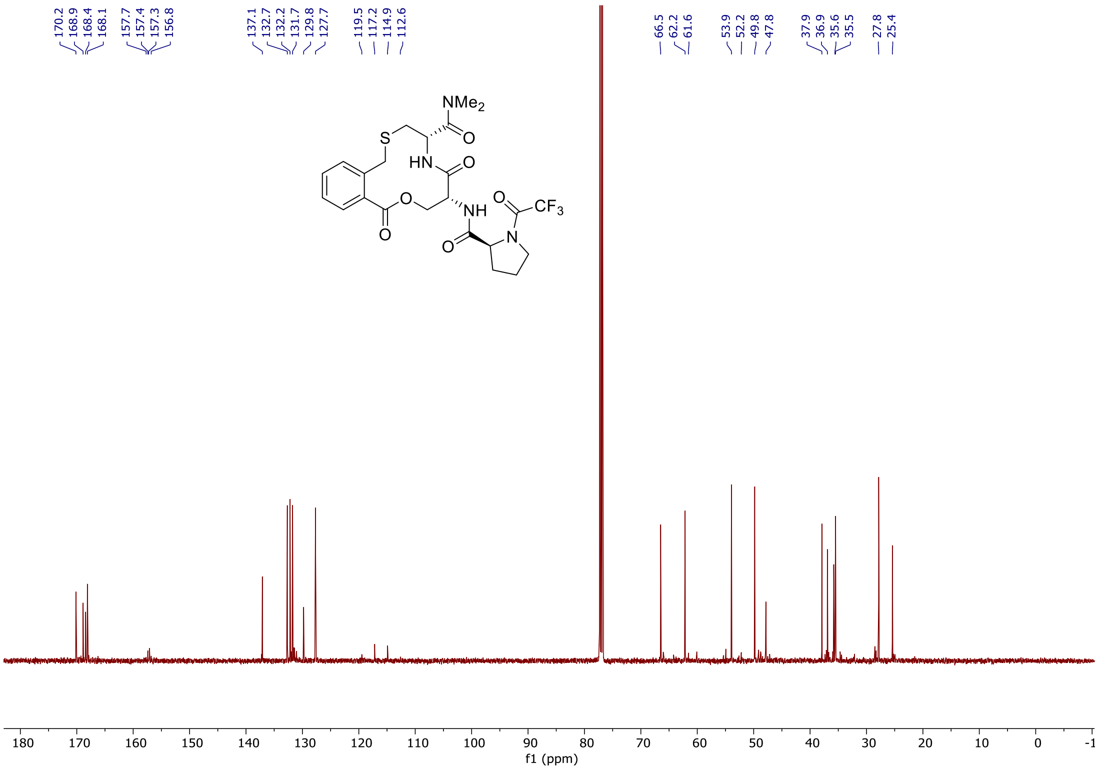

31 ^{1}H NMR (500 MHz, CDCl$_3$)

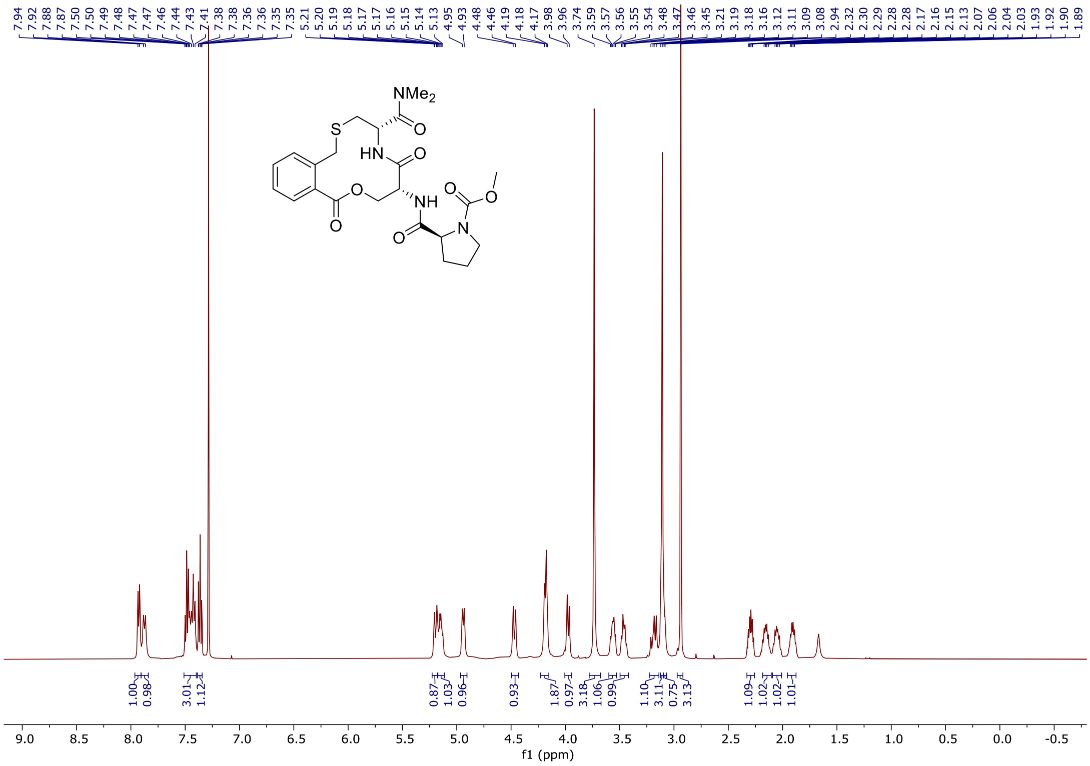

31 ^{13}C NMR (126 MHz, CDCl$_3$)

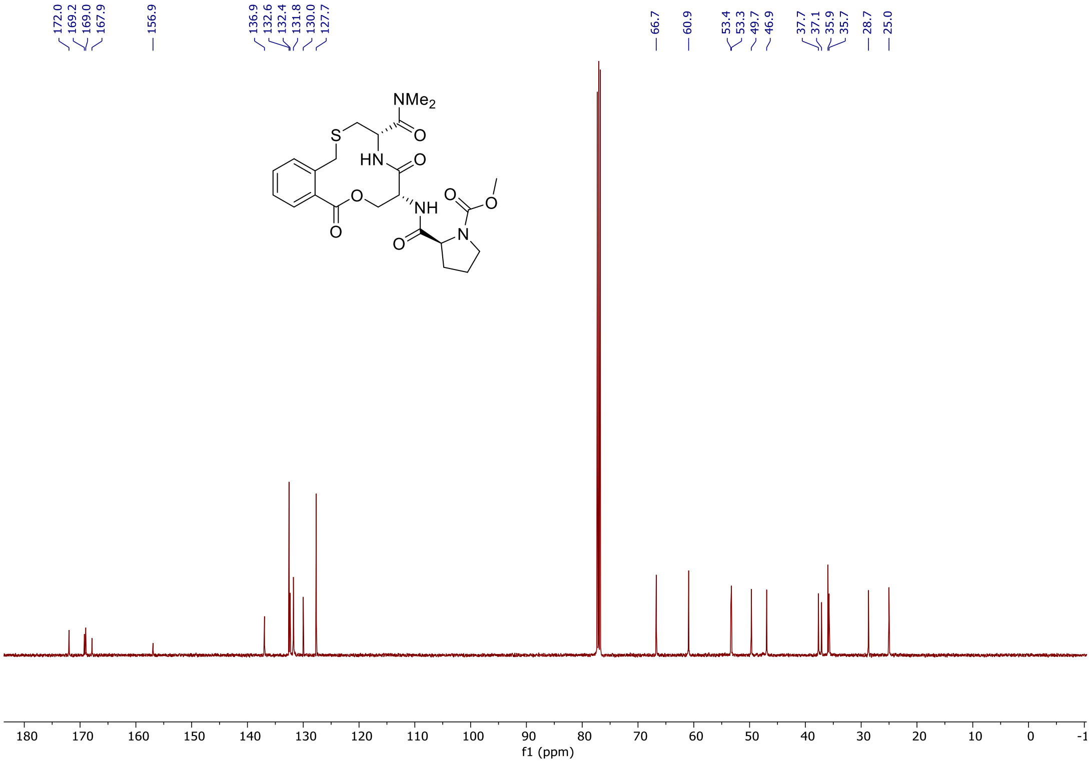

32 ^{1}H NMR (500 MHz, CDCl$_3$)

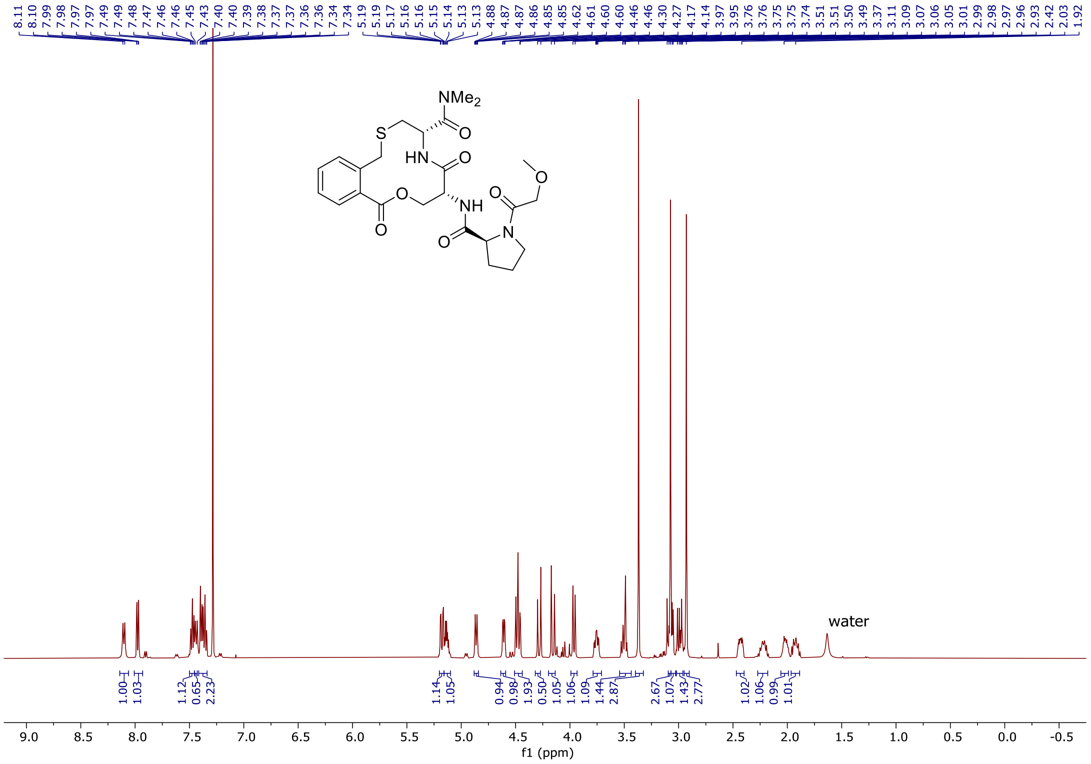

32 ^{13}C NMR (126 MHz, CDCl$_3$)

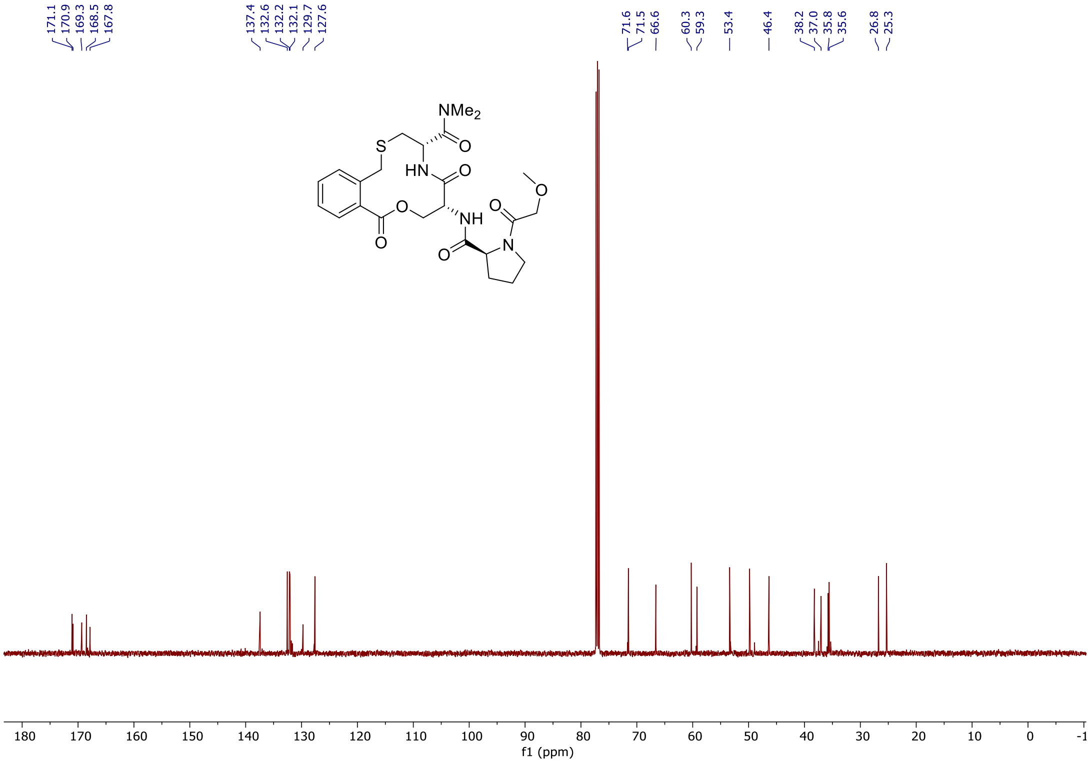

33 ^{1}H NMR (400 MHz, $CDCl_3$)

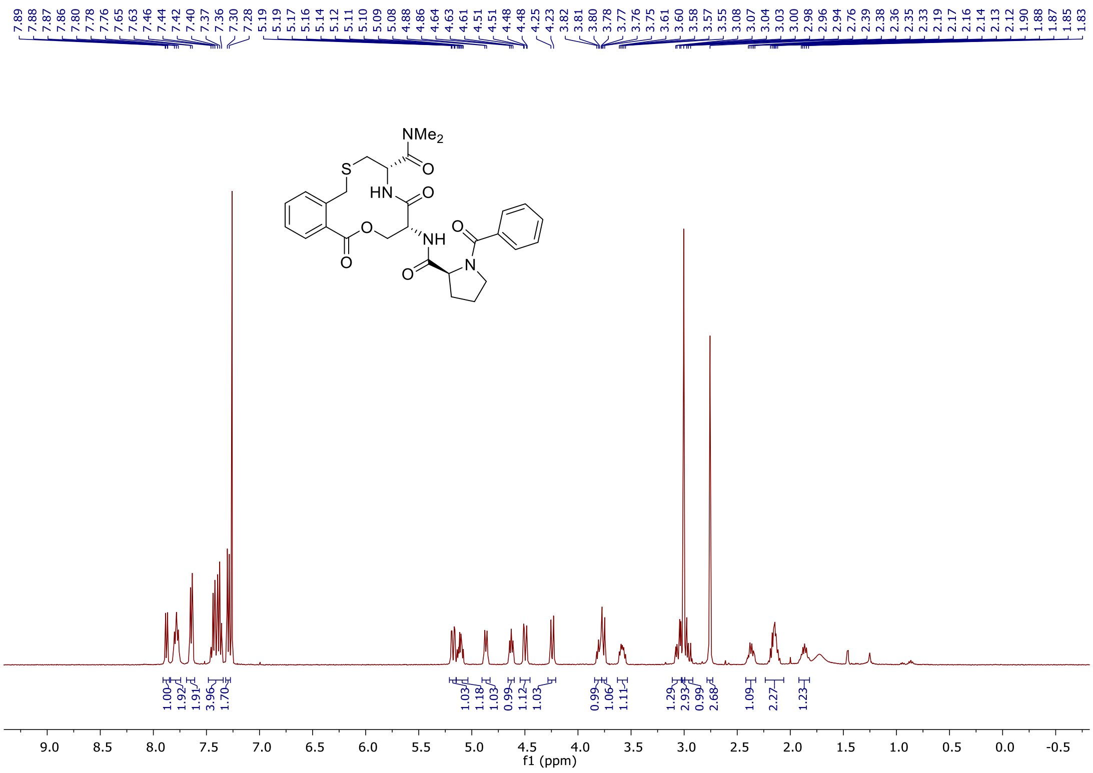

33 ^{13}C NMR (101 MHz, $CDCl_3$)

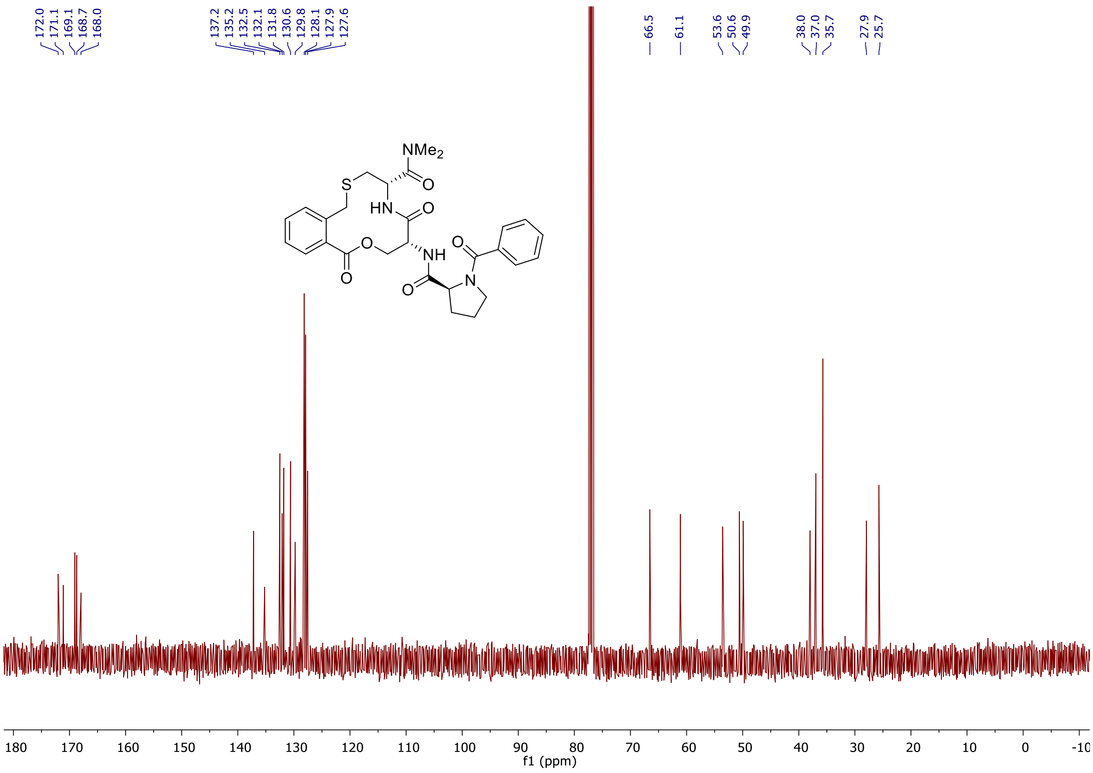

34 ^{1}H NMR (400 MHz, $CDCl_3$)

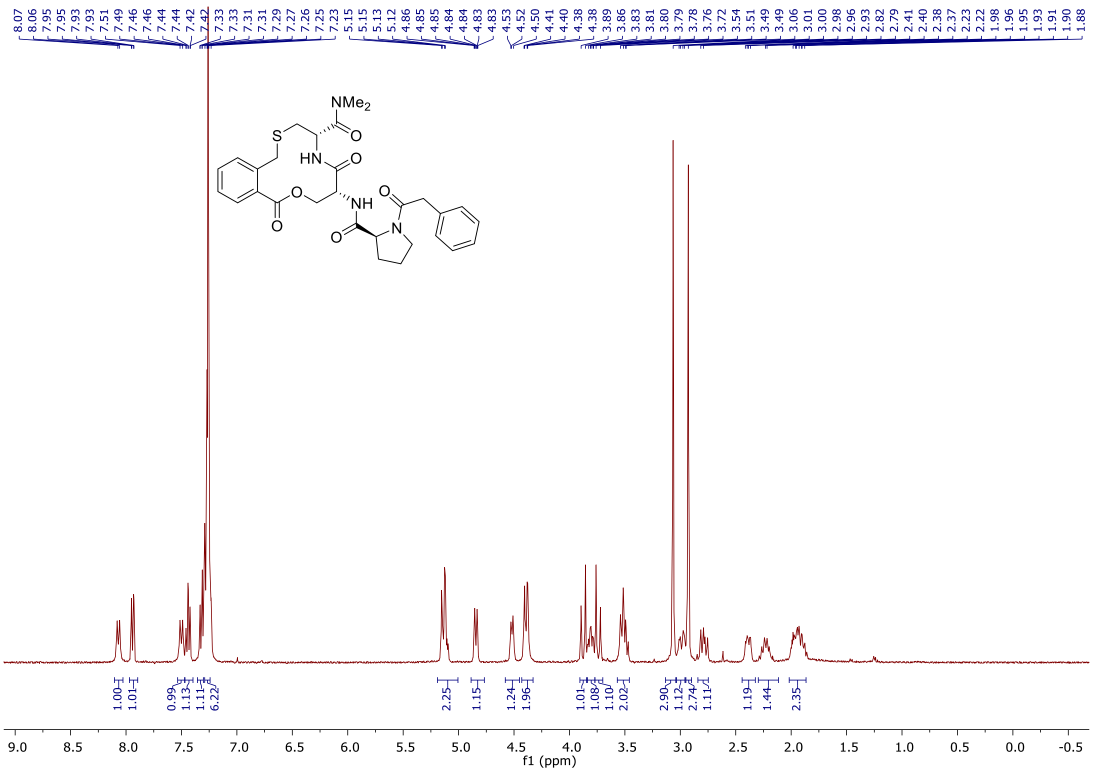

34 ^{13}C NMR (101 MHz, $CDCl_3$)

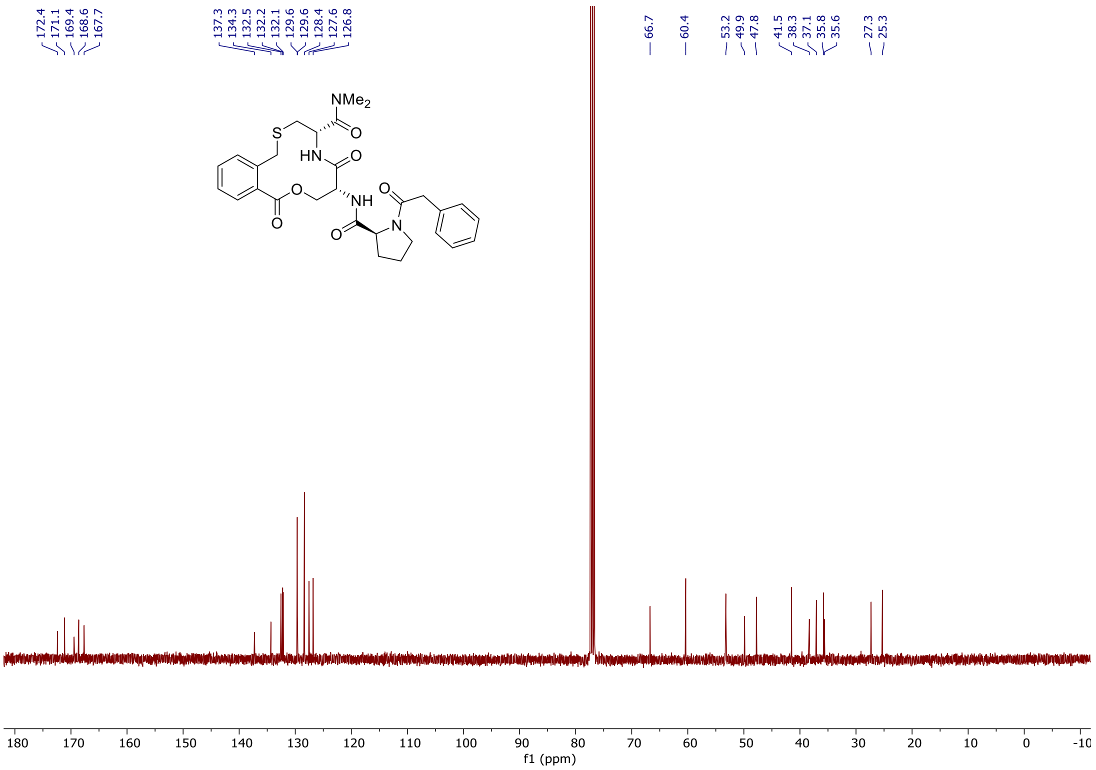

35 ^{1}H NMR (400 MHz, $CDCl_3$)

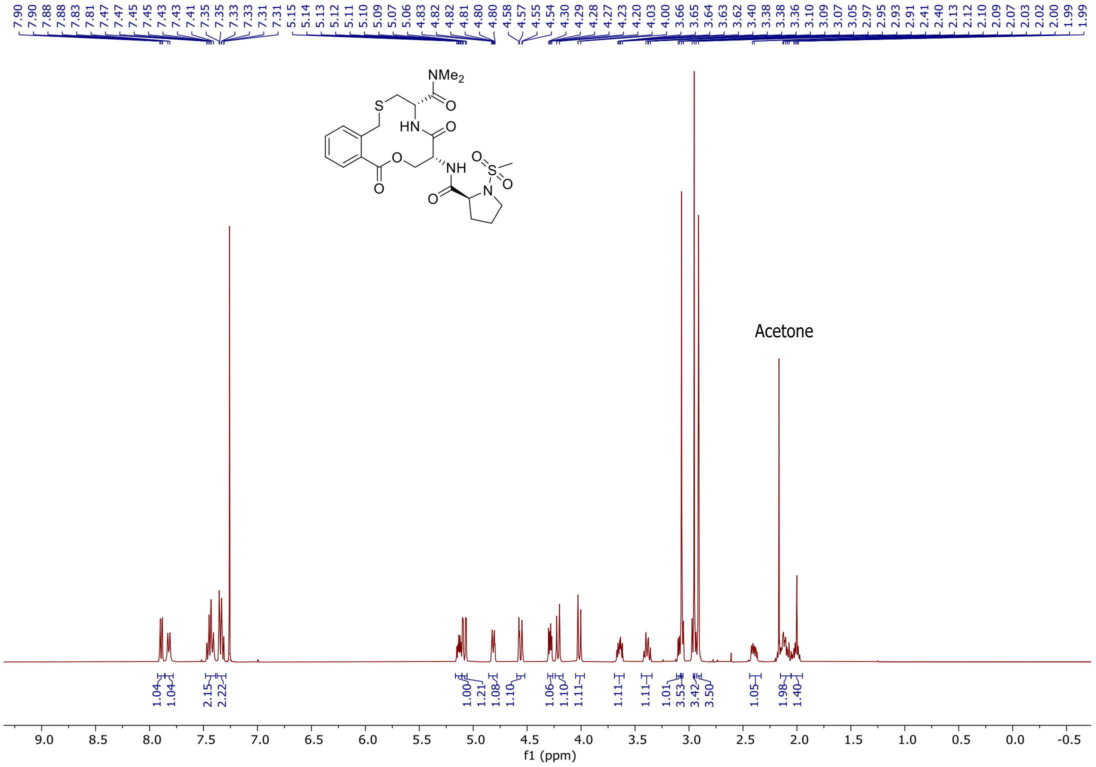

35 ^{13}C NMR (101 MHz, $CDCl_3$)

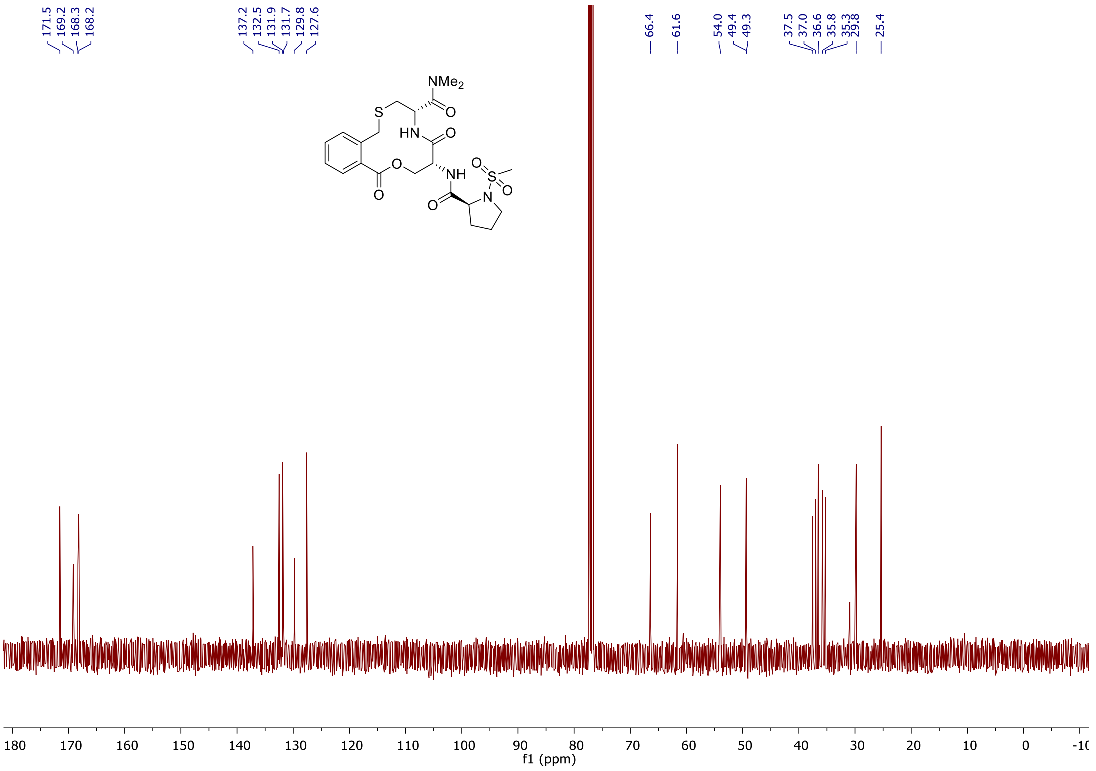

36 ^{1}H NMR (500 MHz, $CDCl_3$)

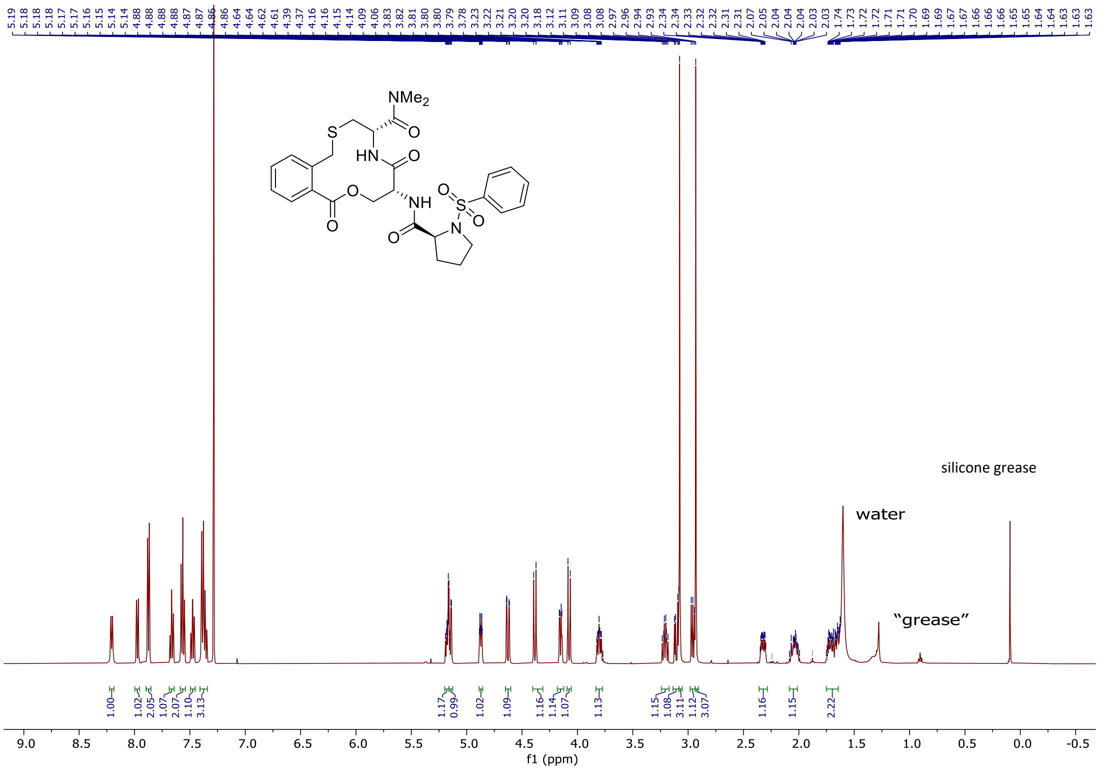

36 ^{13}C NMR (126 MHz, $CDCl_3$)

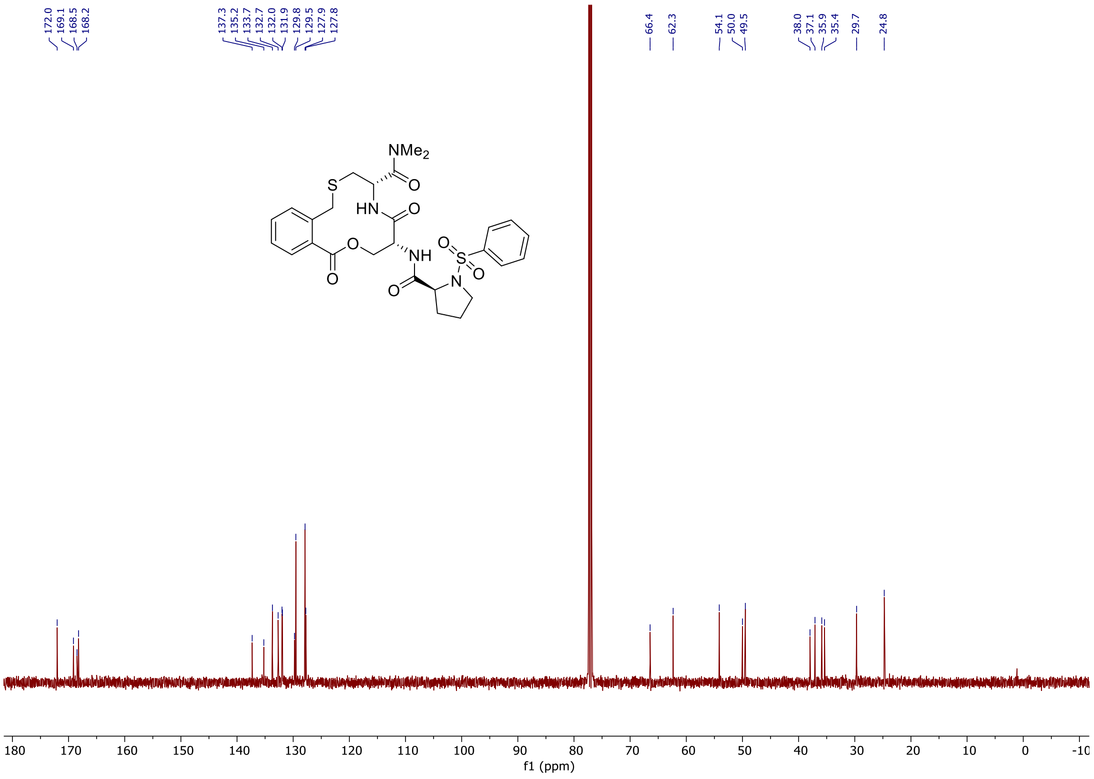

37 ^{1}H NMR (400 MHz, CDCl$_3$)

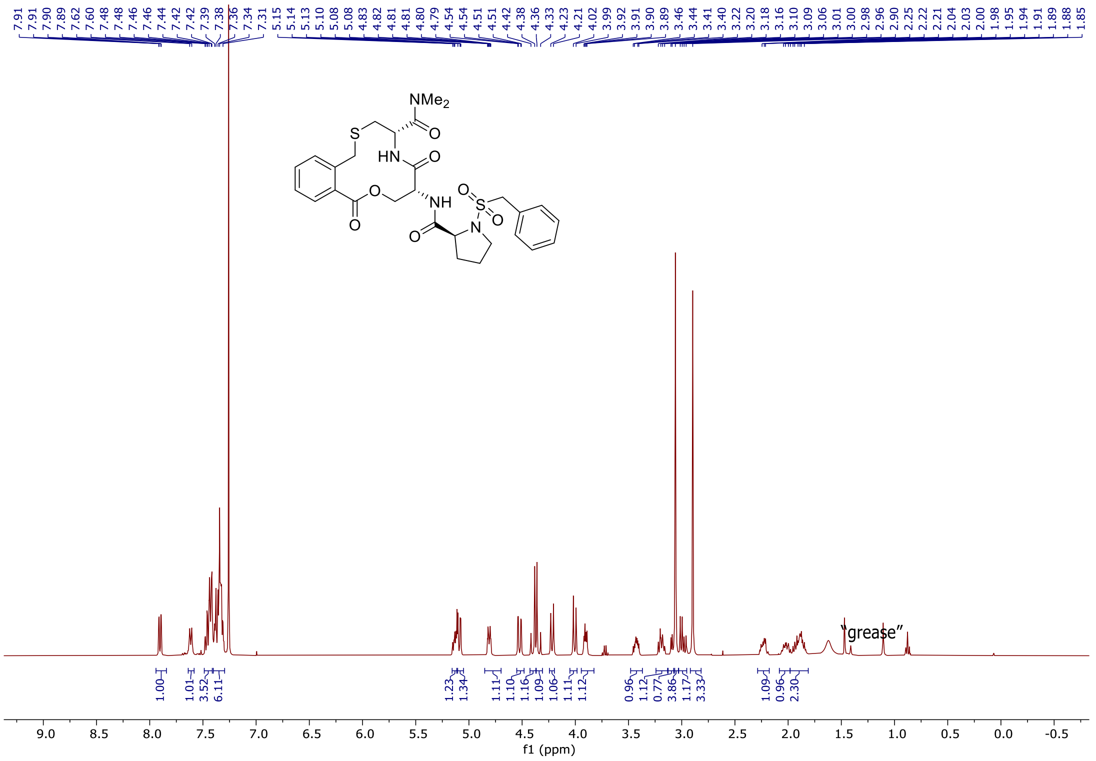

37 ^{13}C NMR (101 MHz, CDCl$_3$)

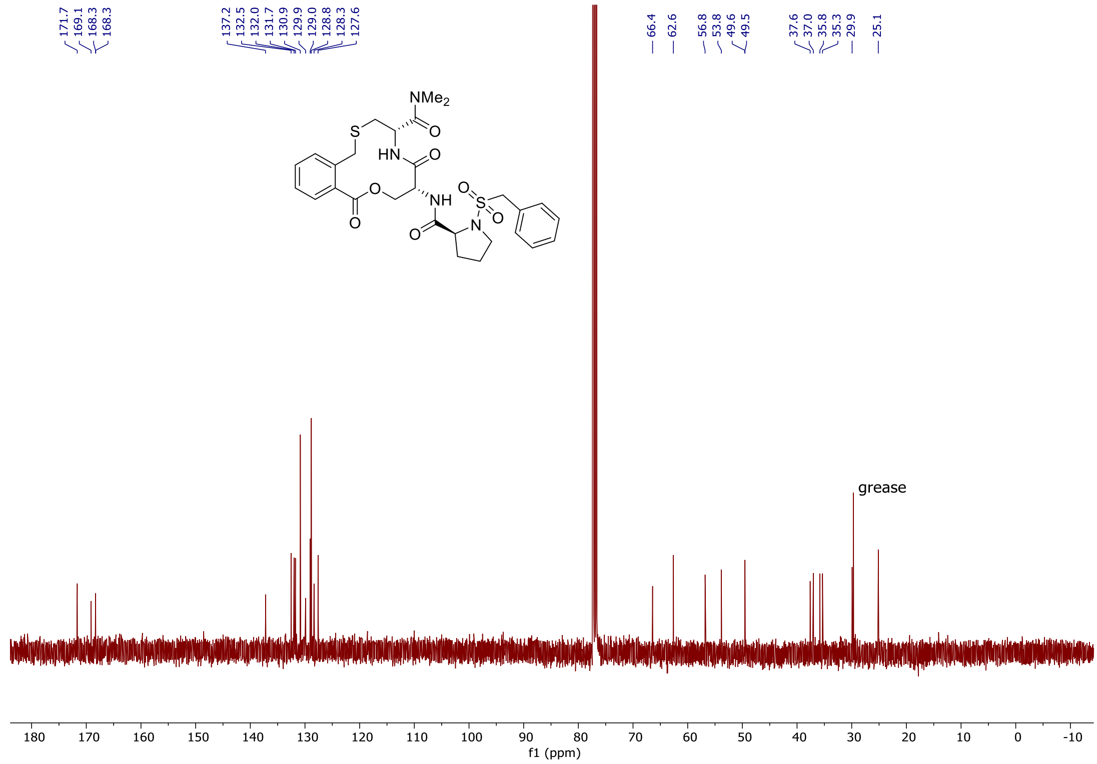

38 ^{1}H NMR (500 MHz, $CDCl_3$)

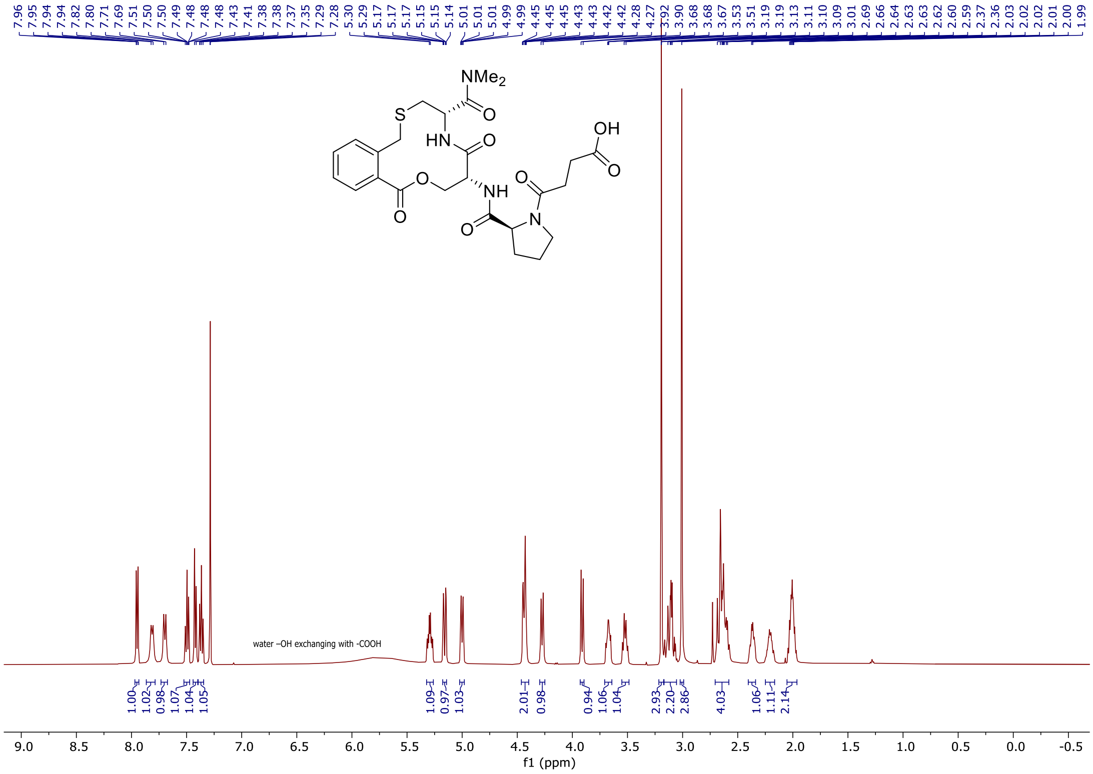

38 ^{13}C NMR (126 MHz, $CDCl_3$)

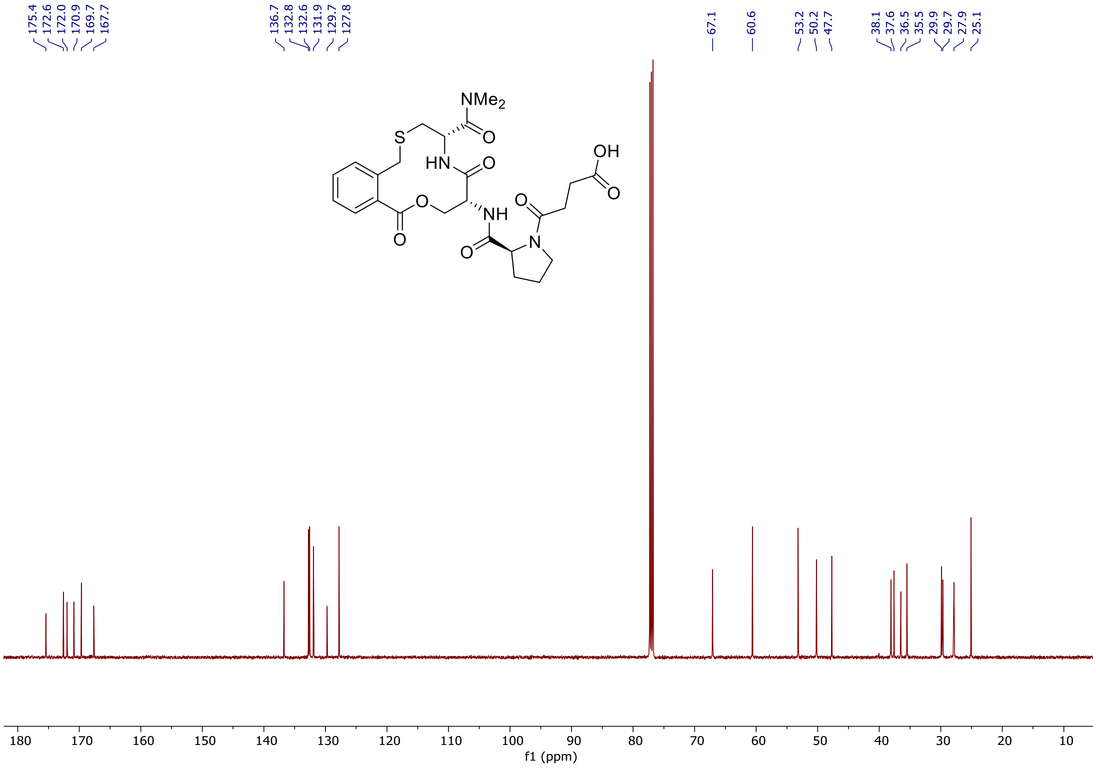

39 ^{1}H NMR (500 MHz, $CDCl_3$)

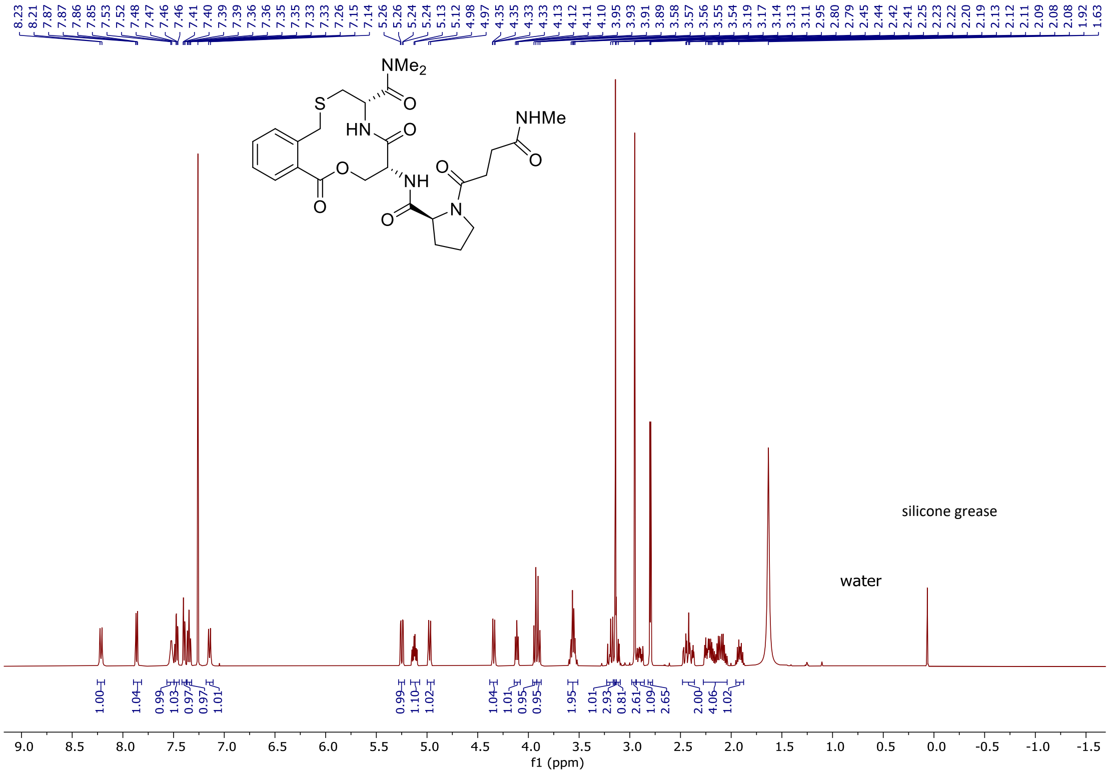

39 ^{13}C NMR (126 MHz, $CDCl_3$)

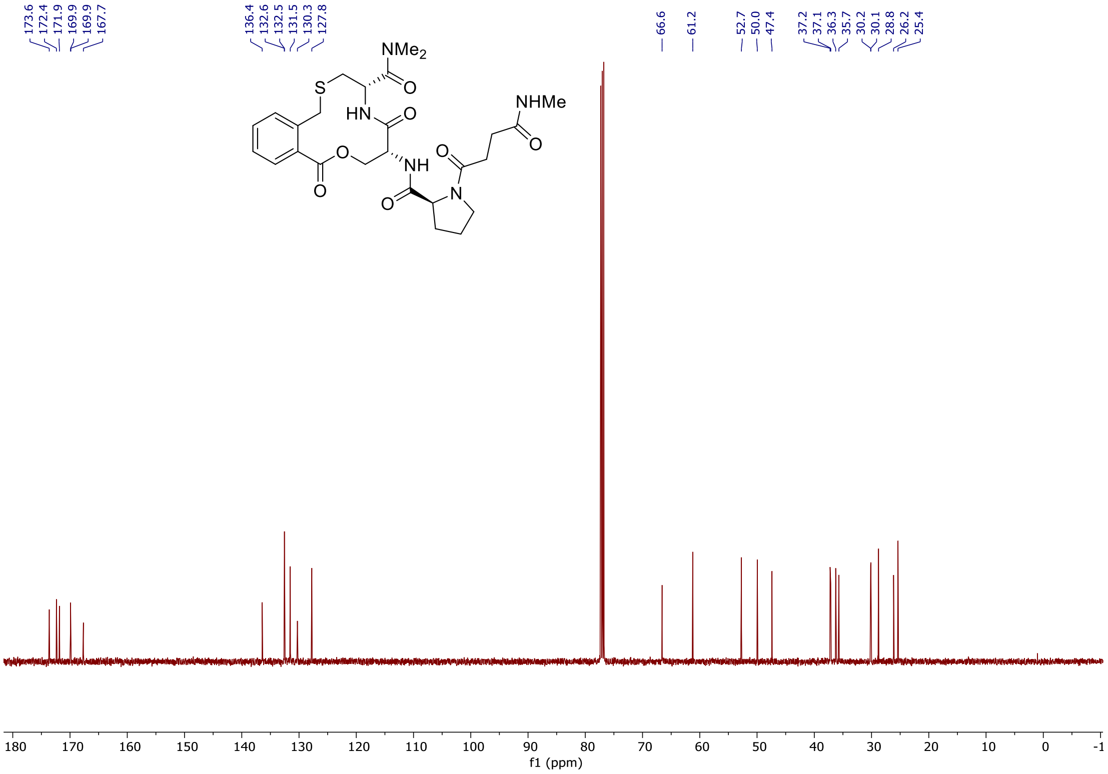

40 ^{1}H NMR (500 MHz, $CDCl_3$)

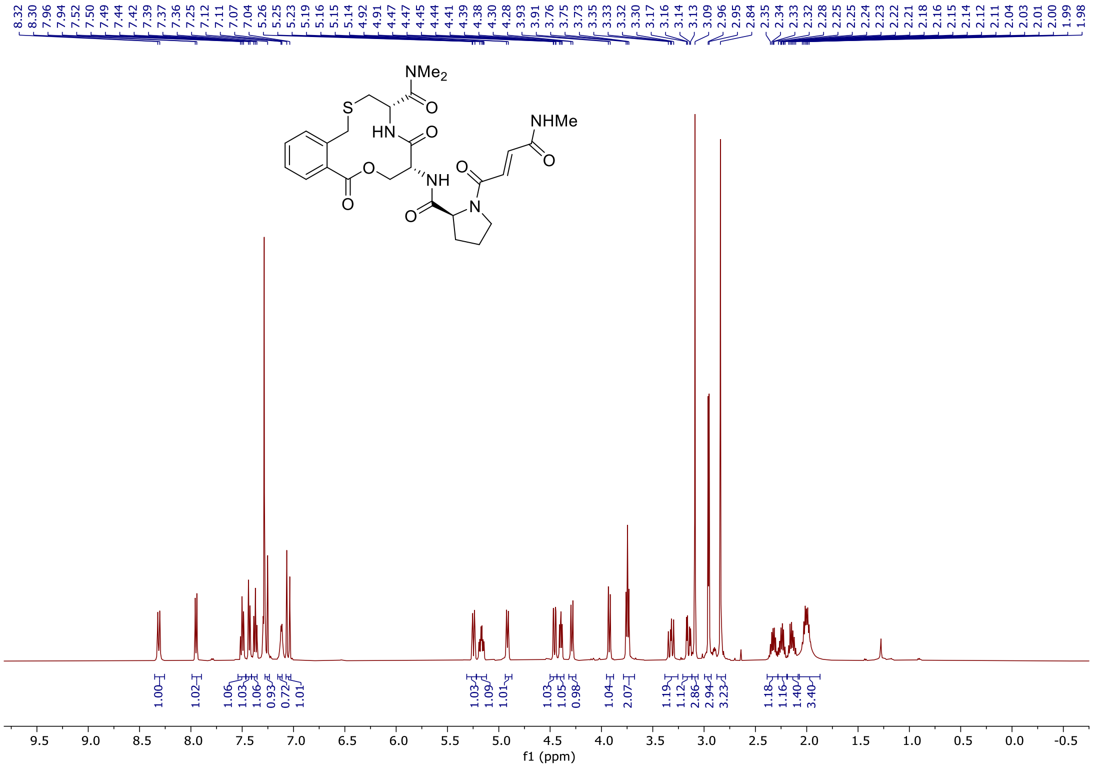

40 ^{13}C NMR (126 MHz, $CDCl_3$)

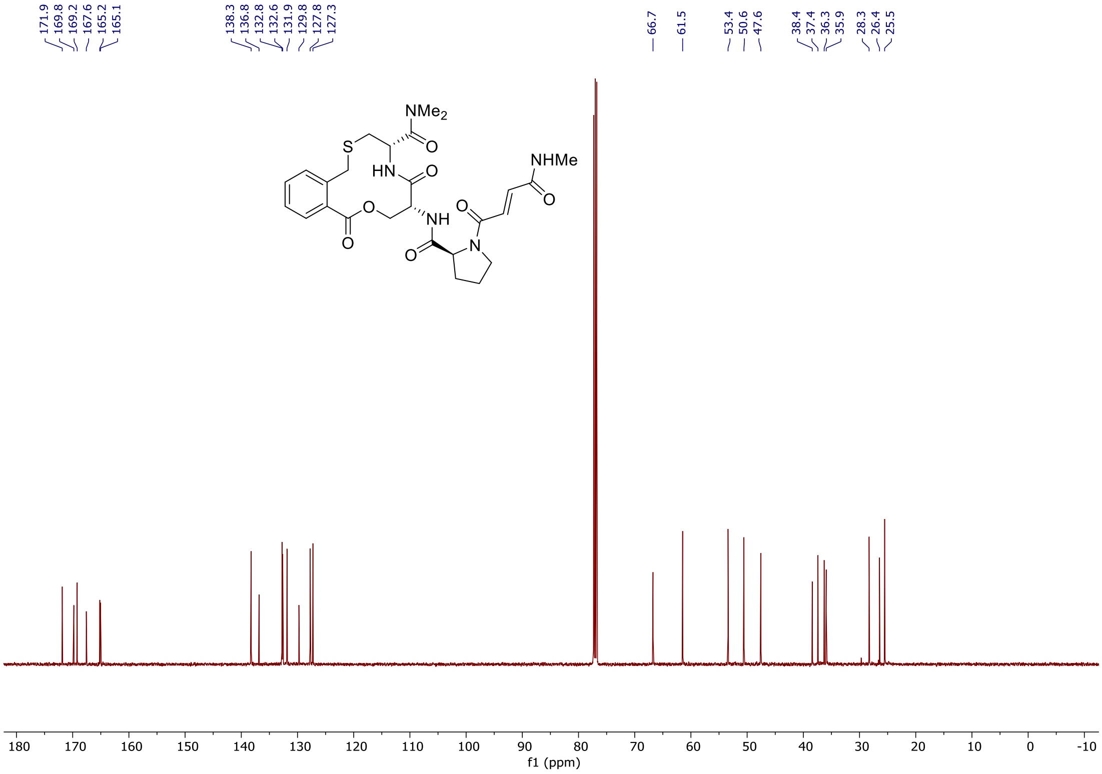

41 ^{1}H NMR (500 MHz, CDCl$_3$)

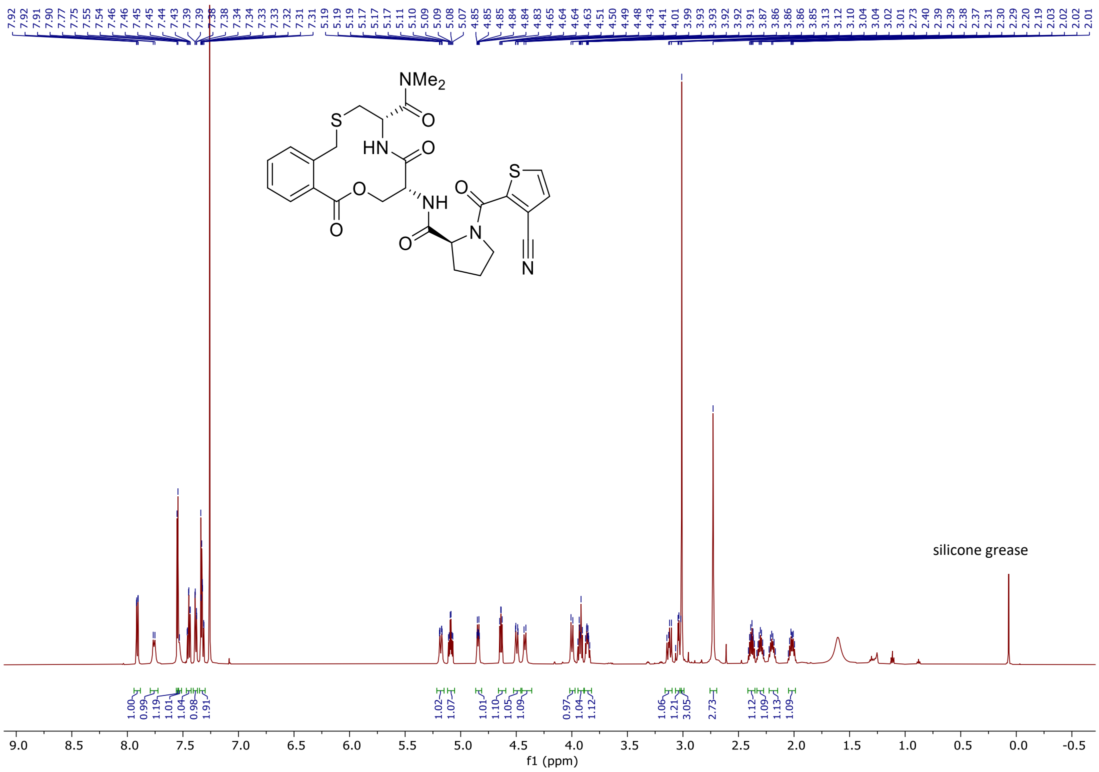

41 ^{13}C NMR (126 MHz, CDCl$_3$)

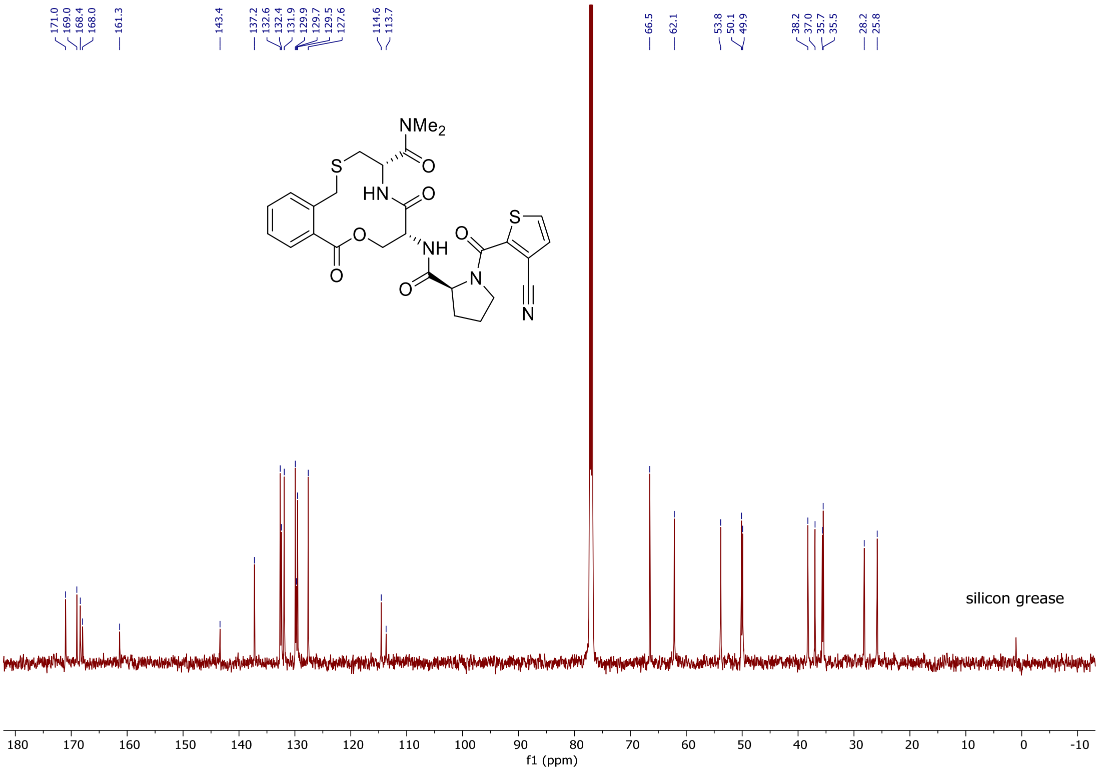

42 ^{1}H NMR (500 MHz, $CDCl_3$)

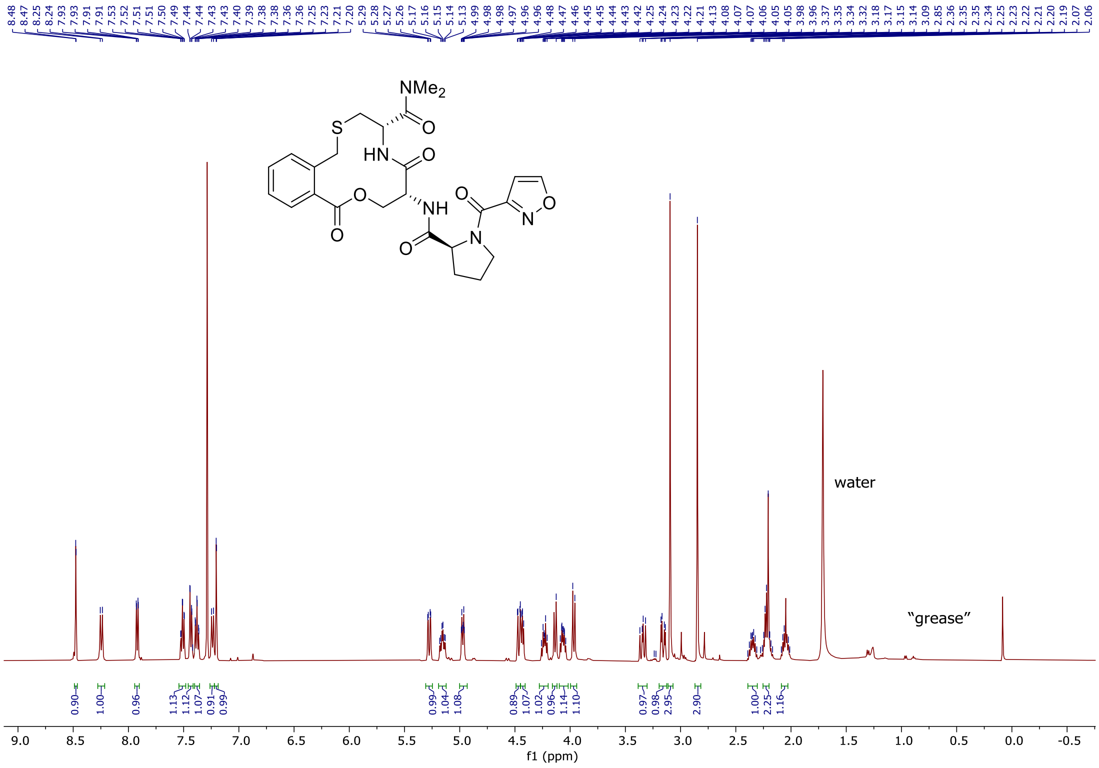

42 ^{13}C NMR (126 MHz, $CDCl_3$)

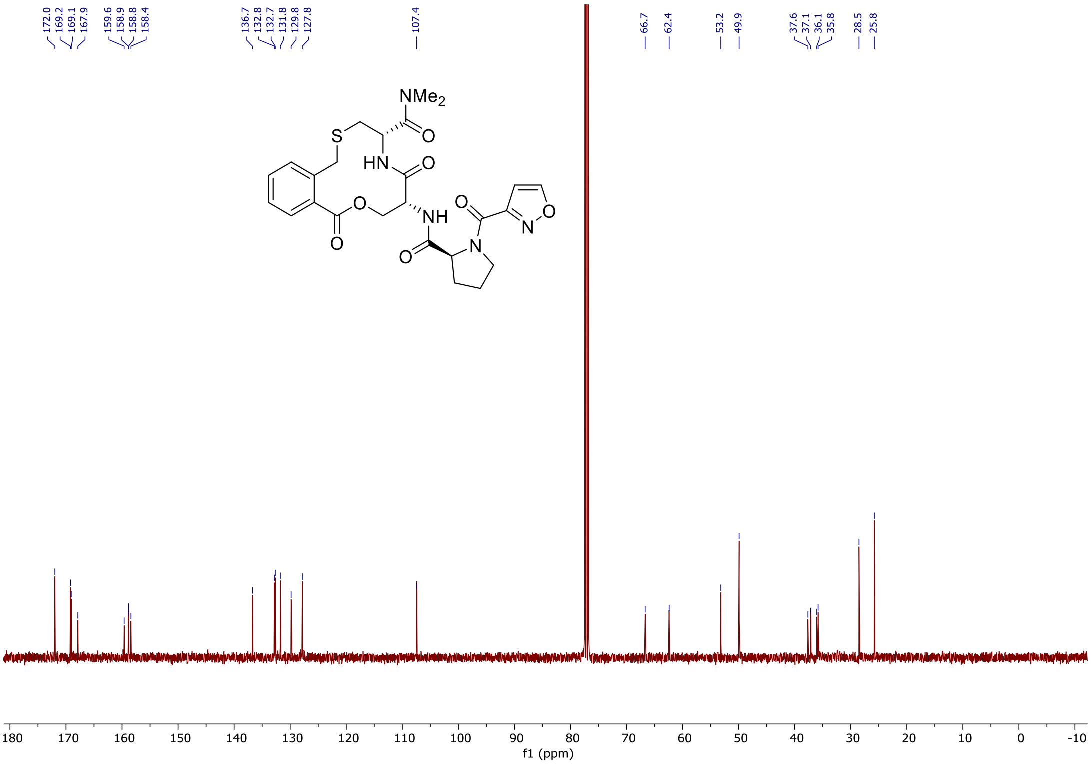

43 ^{1}H NMR (600 MHz, $CDCl_3$)

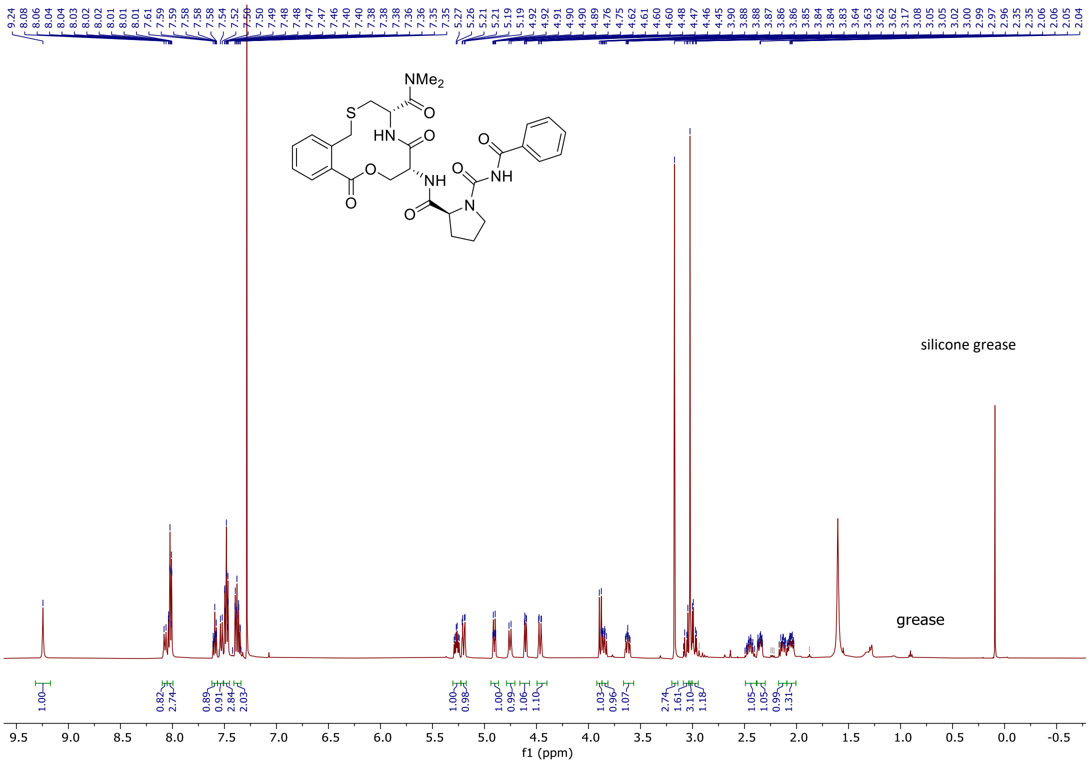

43 ^{13}C NMR (151 MHz, $CDCl_3$)

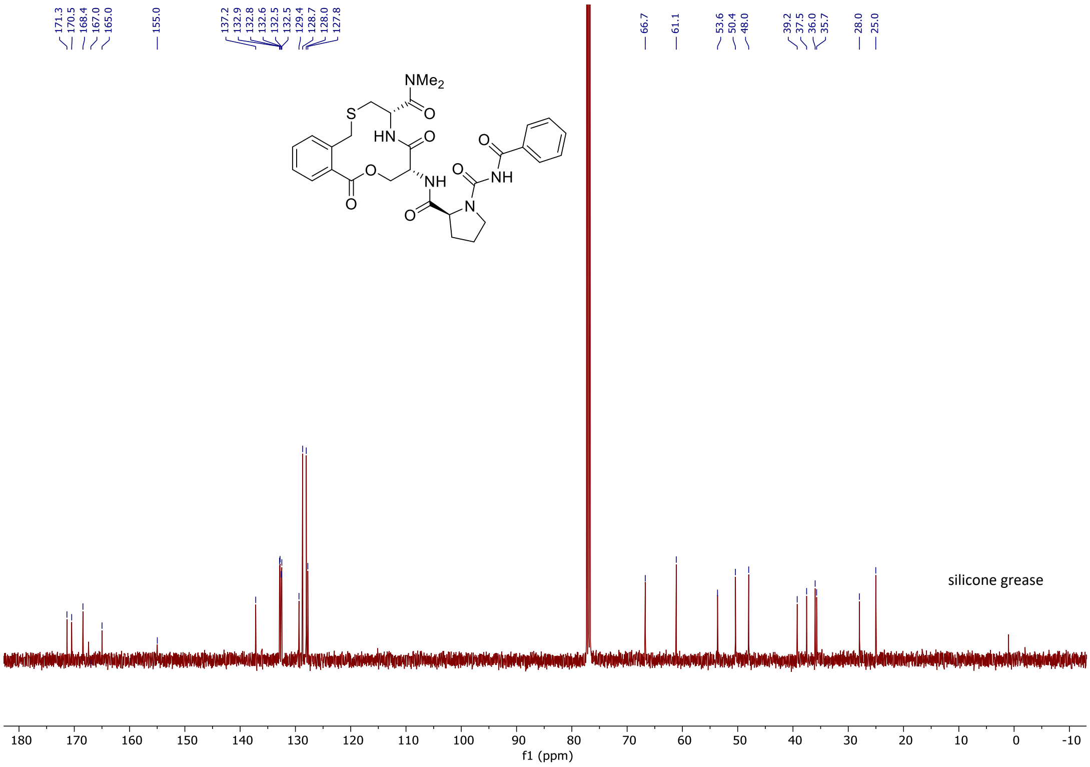

44 ^{1}H NMR (500 MHz, $CDCl_3$)

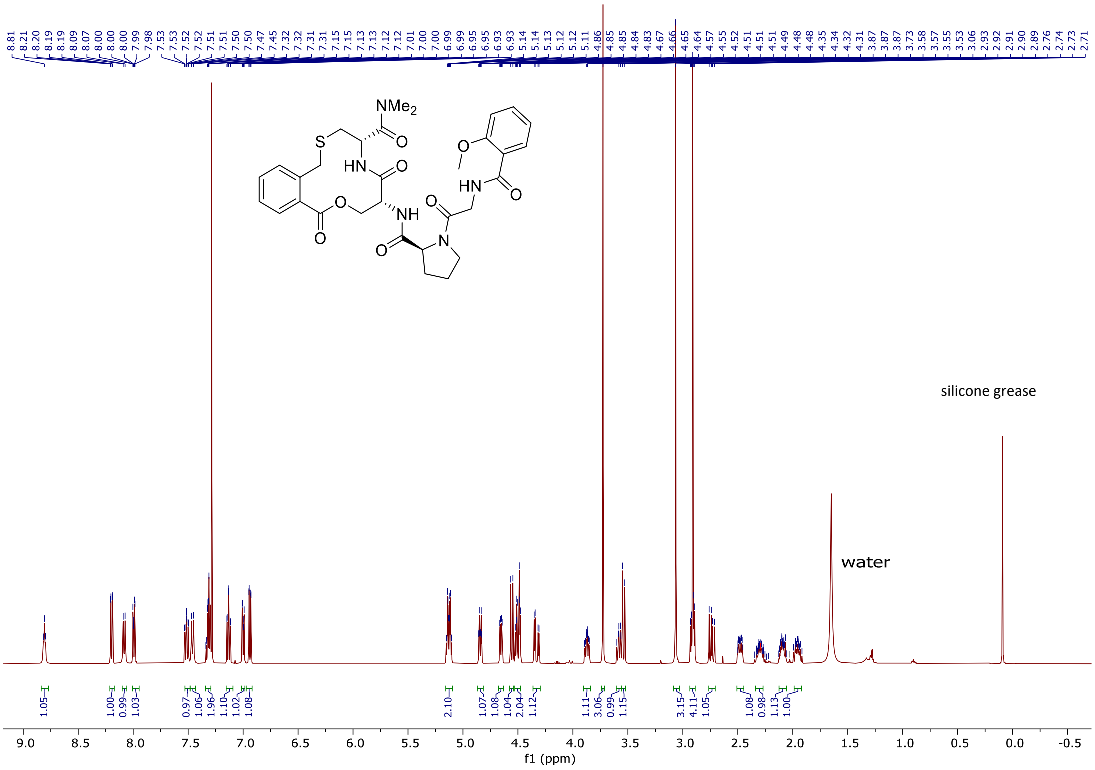

44 ^{13}C NMR (126 MHz, $CDCl_3$)

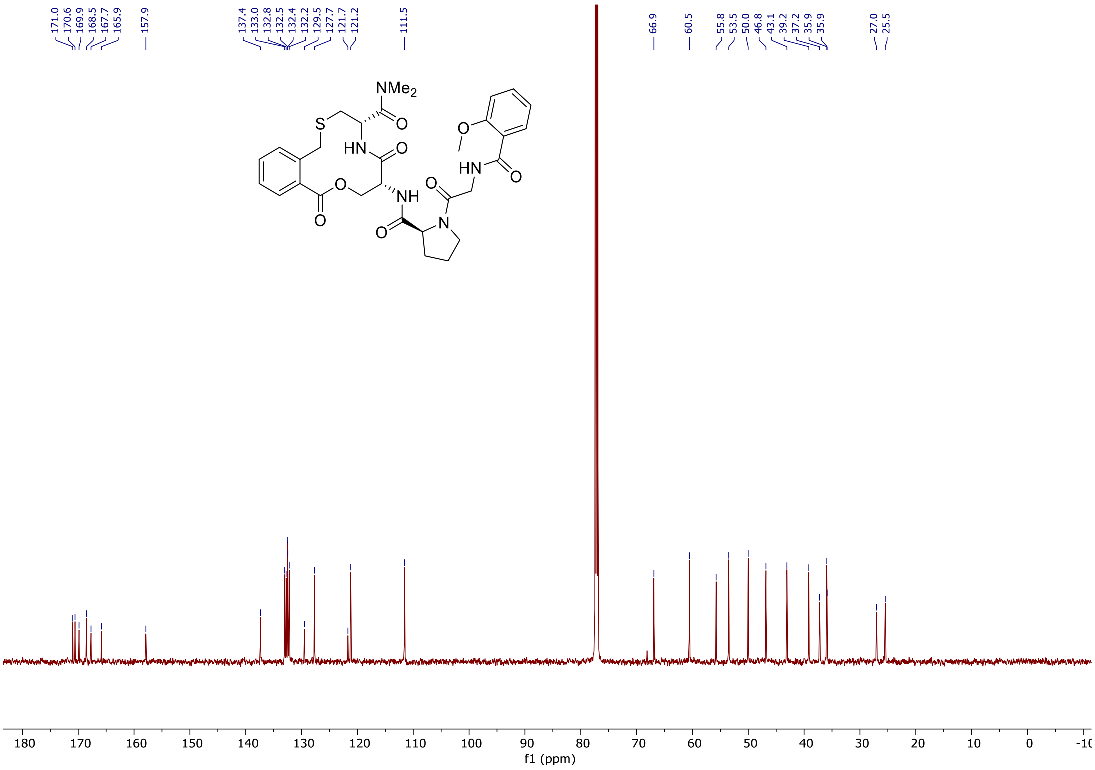

45 ^{1}H NMR (500 MHz, $CDCl_3$)

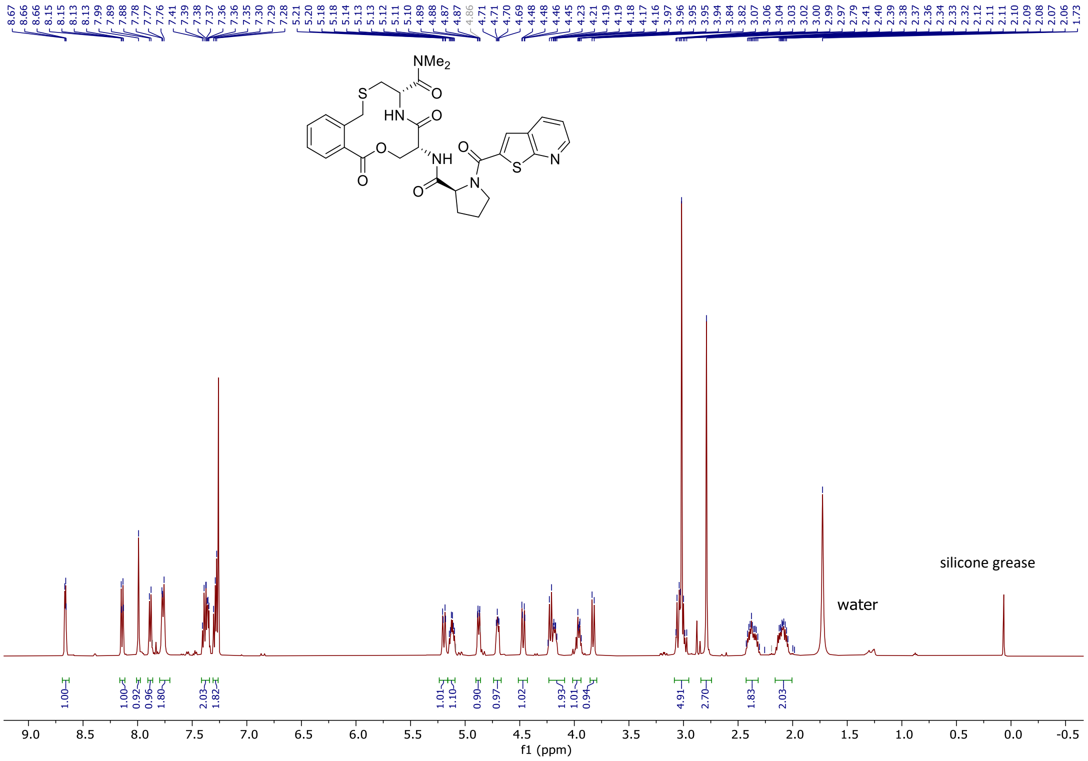

45 ^{13}C NMR (126 MHz, $CDCl_3$)

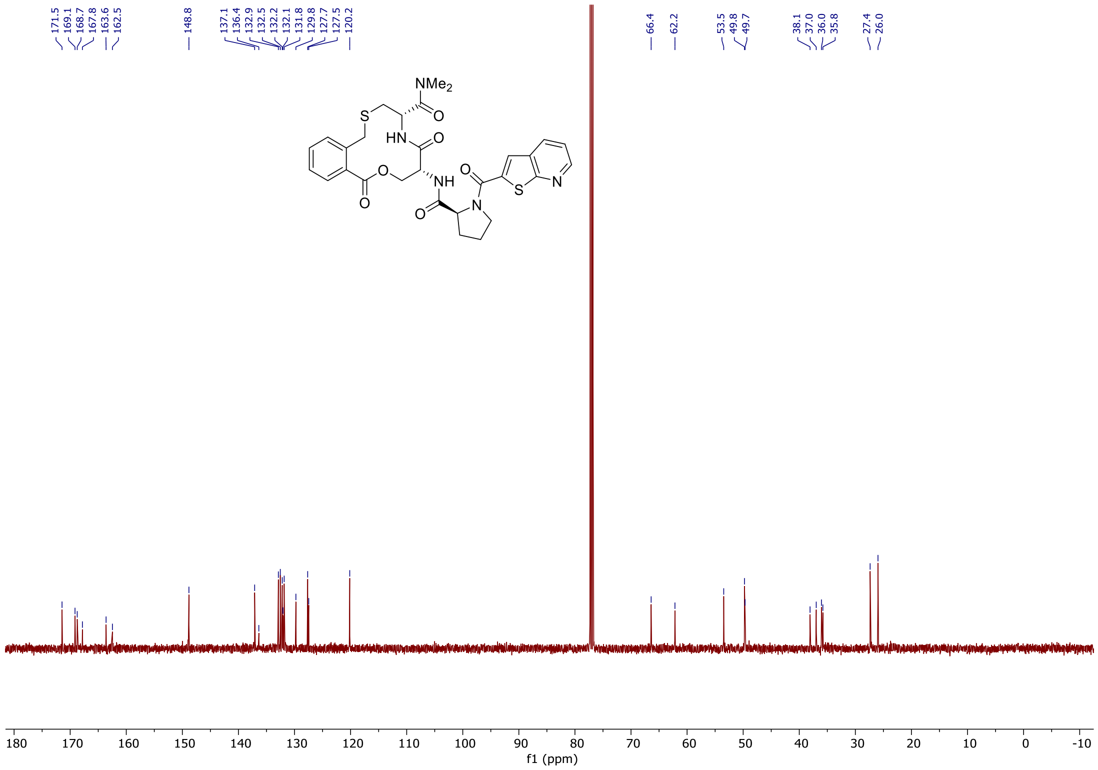

46 ^{1}H NMR (600 MHz, CDCl$_3$)

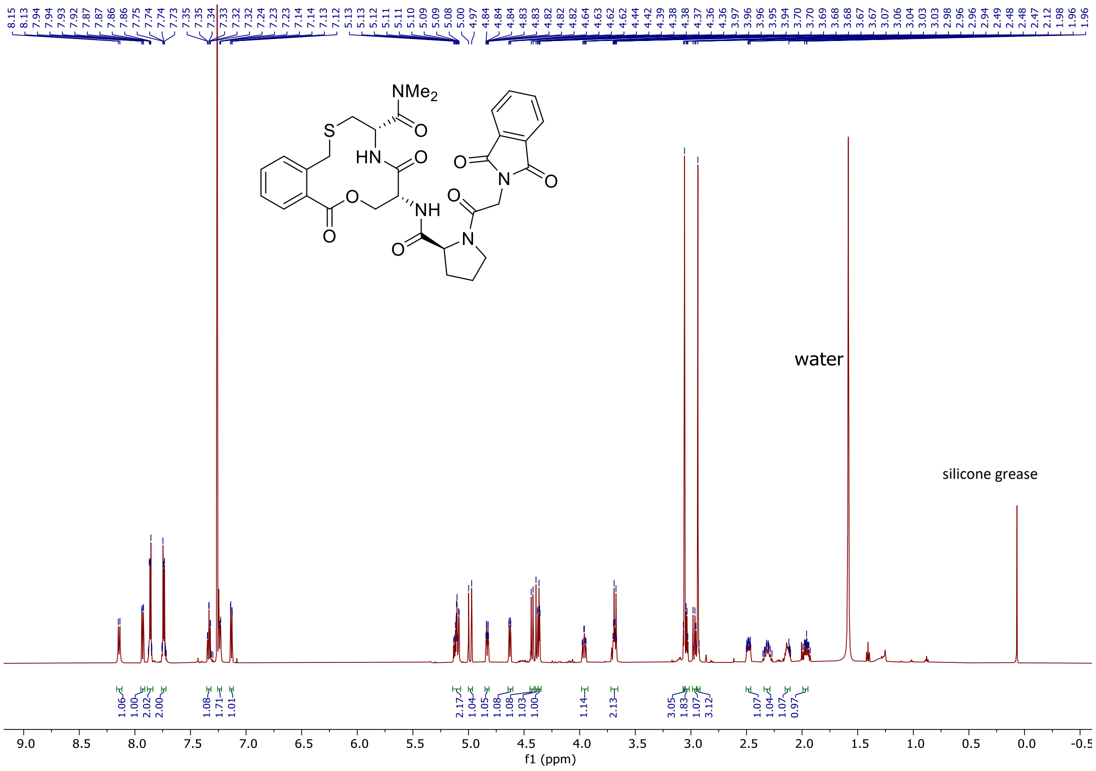

46 ^{13}C NMR (151 MHz, CDCl$_3$)

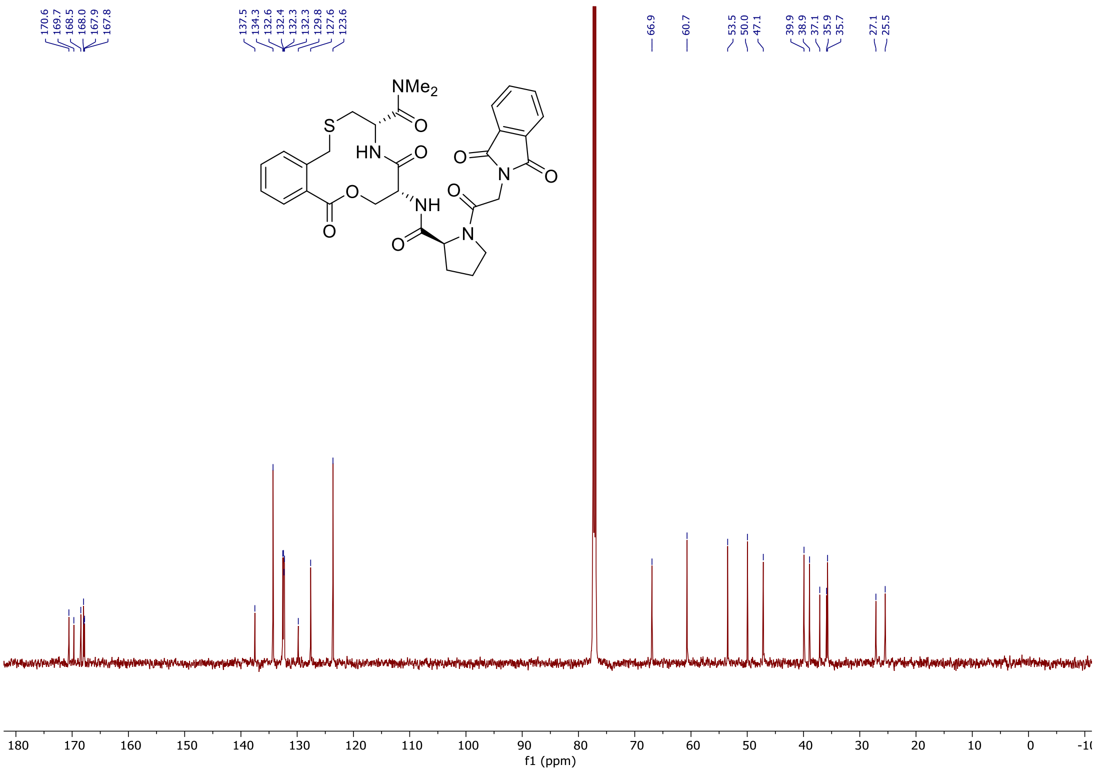

47 ^{1}H NMR (500 MHz, $CDCl_3$)

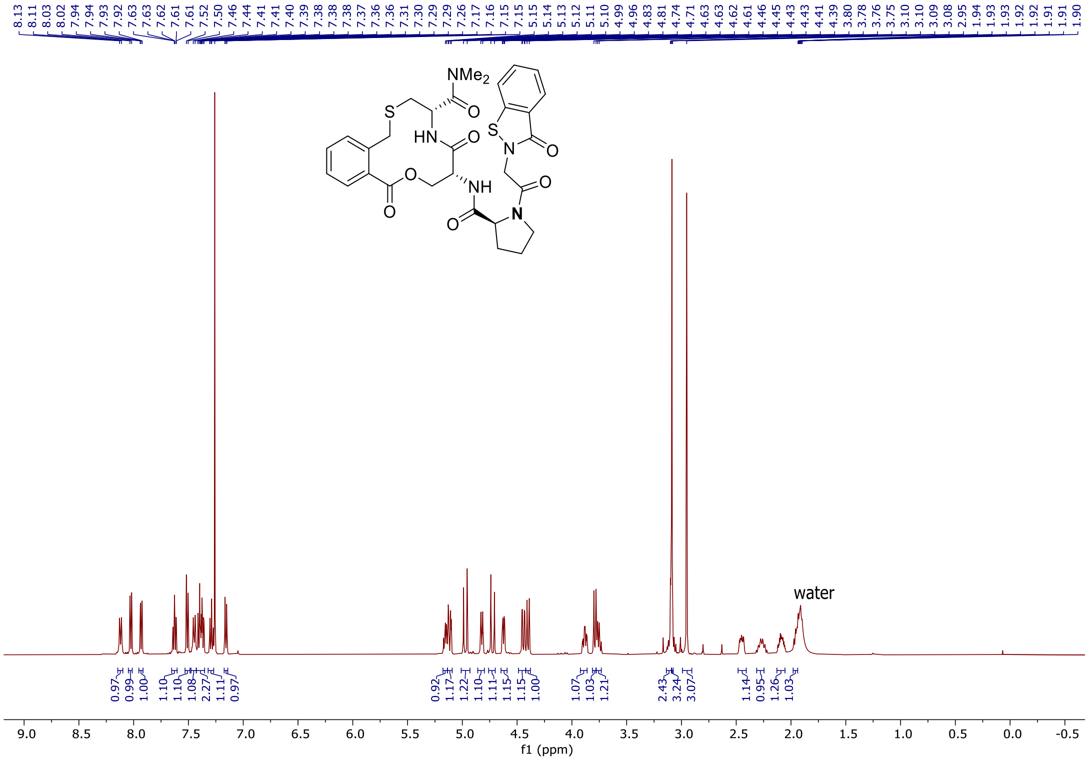

47 ^{13}C NMR (126 MHz, $CDCl_3$)

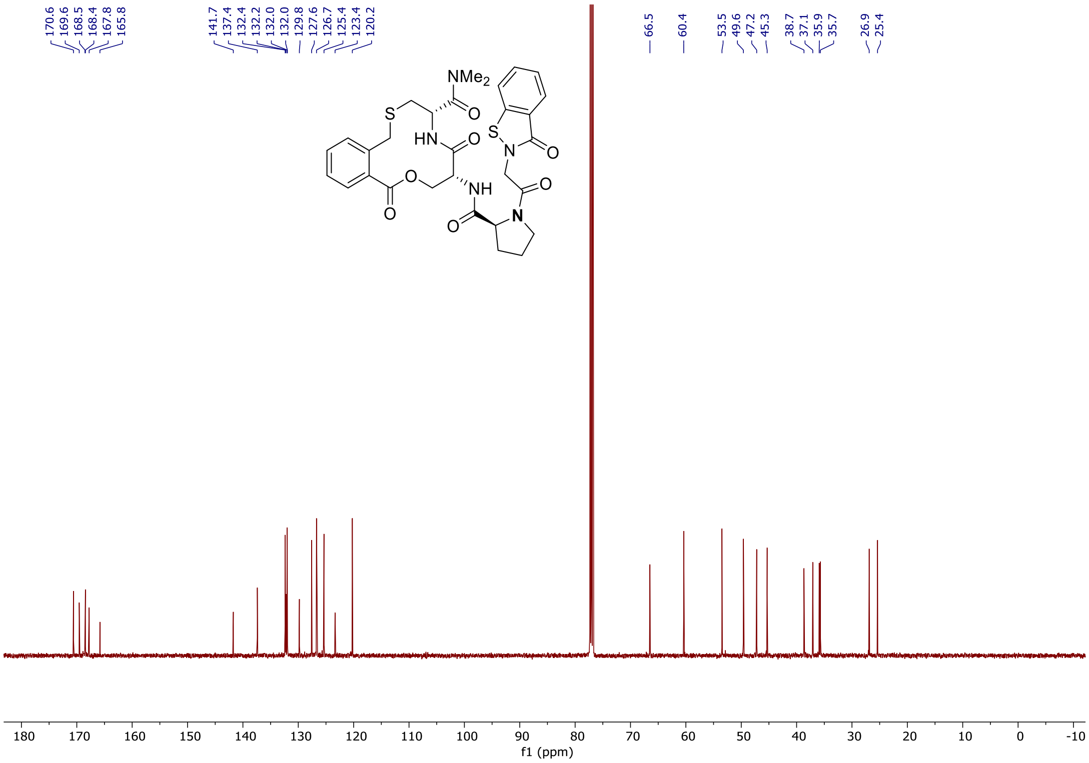

48 ^{1}H NMR (500 MHz, $CDCl_3$, *mixture of 2 diastereoisomers in 1:1 ratio*)

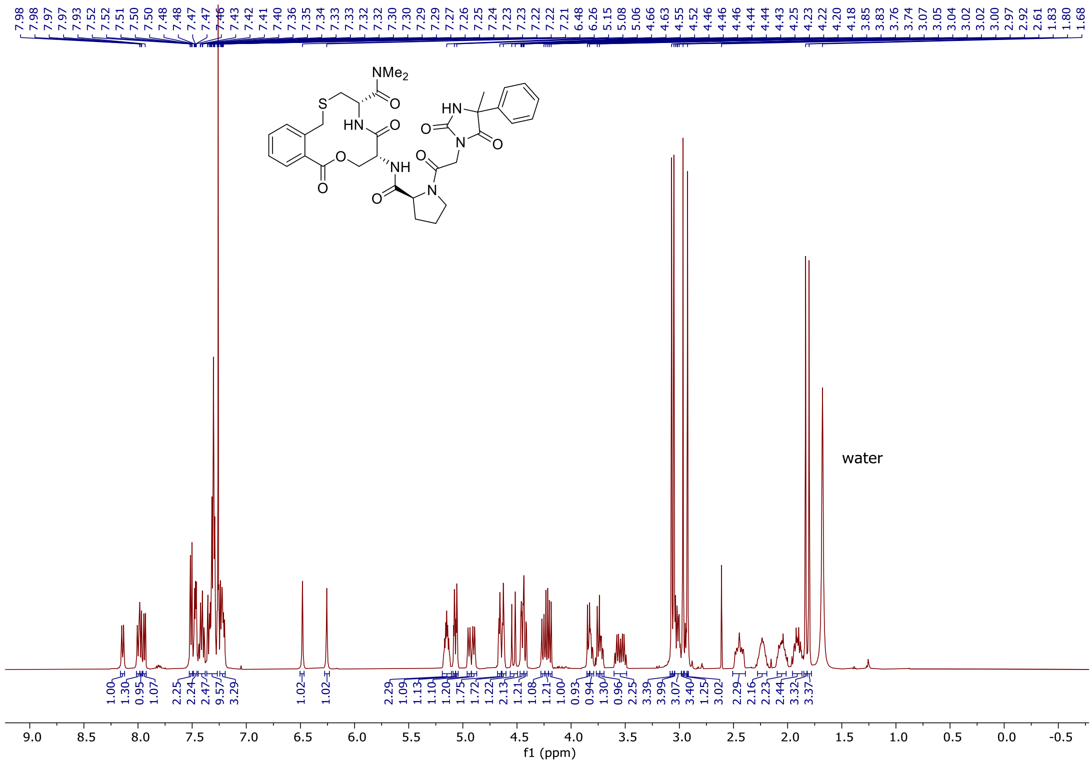

48 ^{13}C NMR (126 MHz, $CDCl_3$, *mixture of 2 diastereoisomers*)

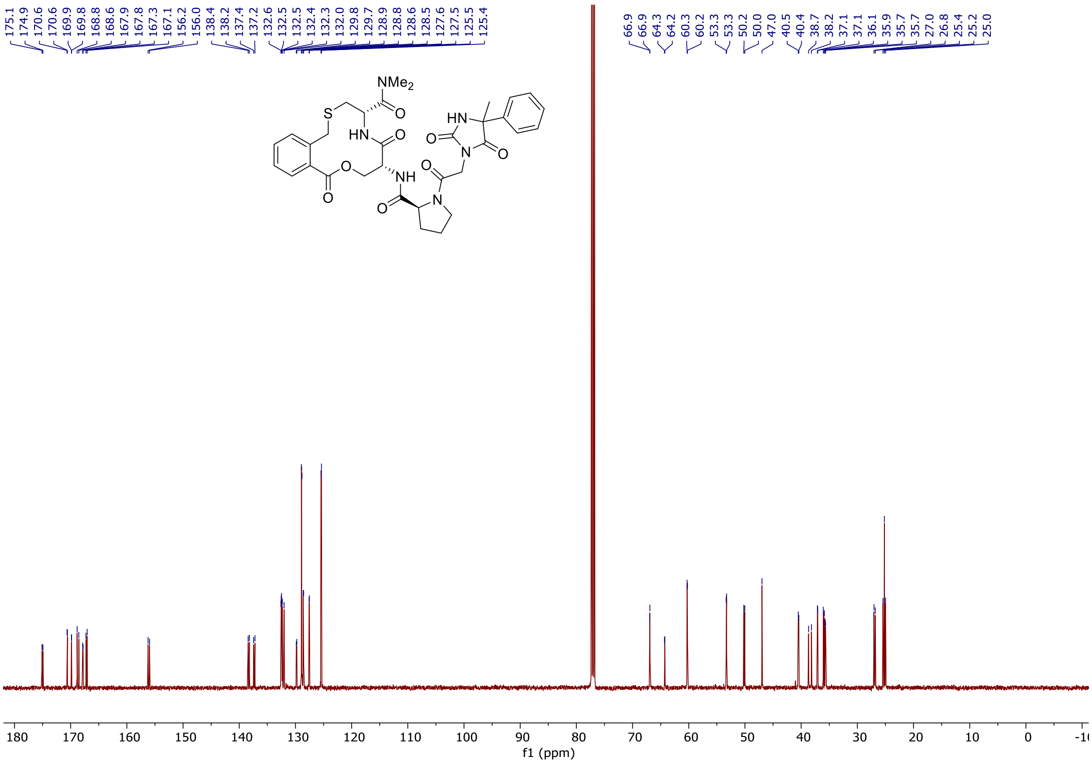

49 ^{1}H NMR (500 MHz, $CDCl_3$, *mixture of 2 rotamers in 1:1 ratio*)

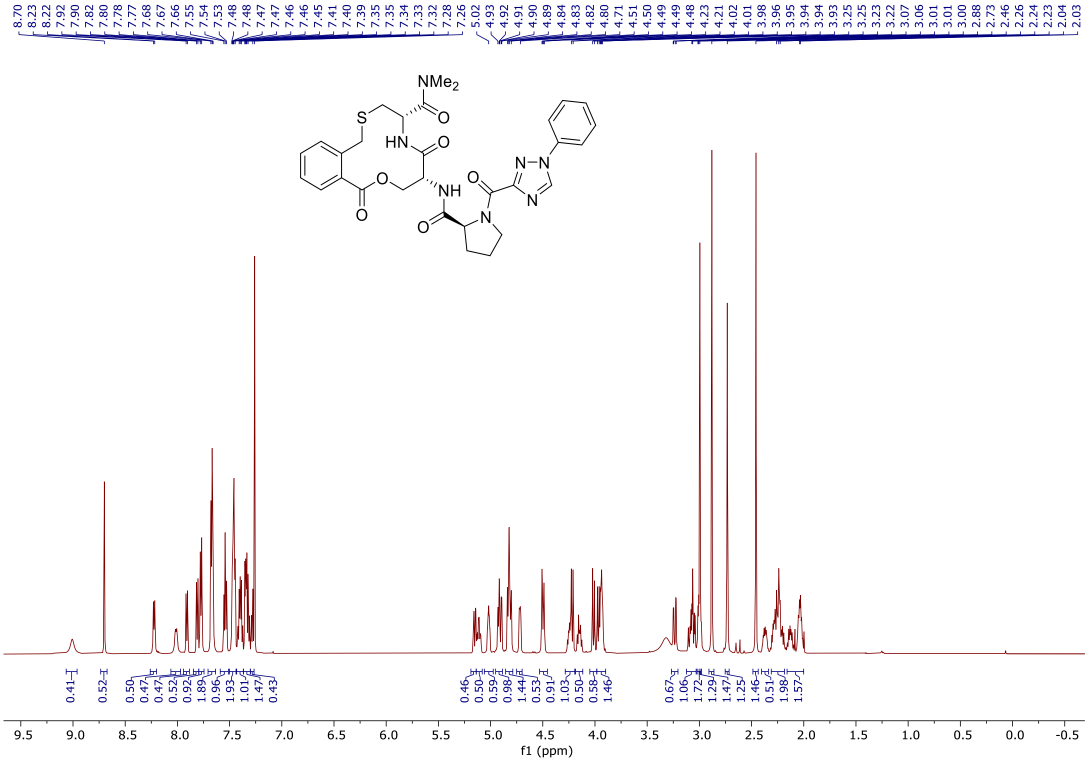

49 ^{13}C NMR (126 MHz, $CDCl_3$, *mixture of 2 rotamers*)

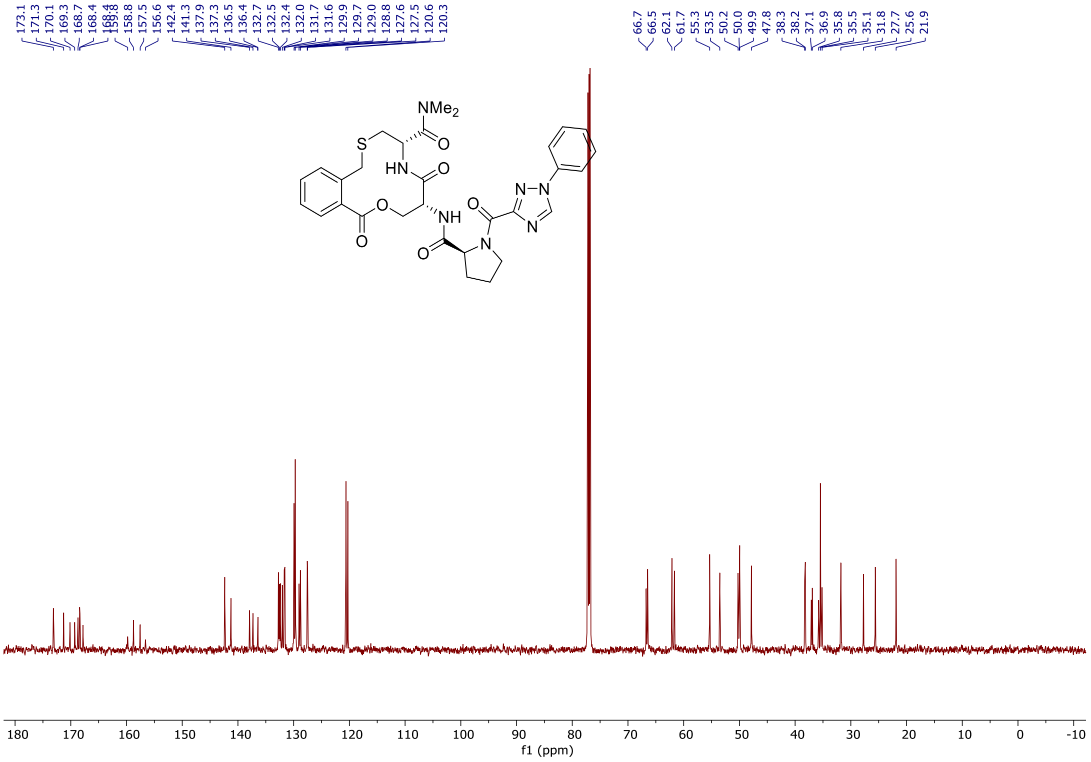

50 ^{1}H NMR (500 MHz, $CDCl_3$)

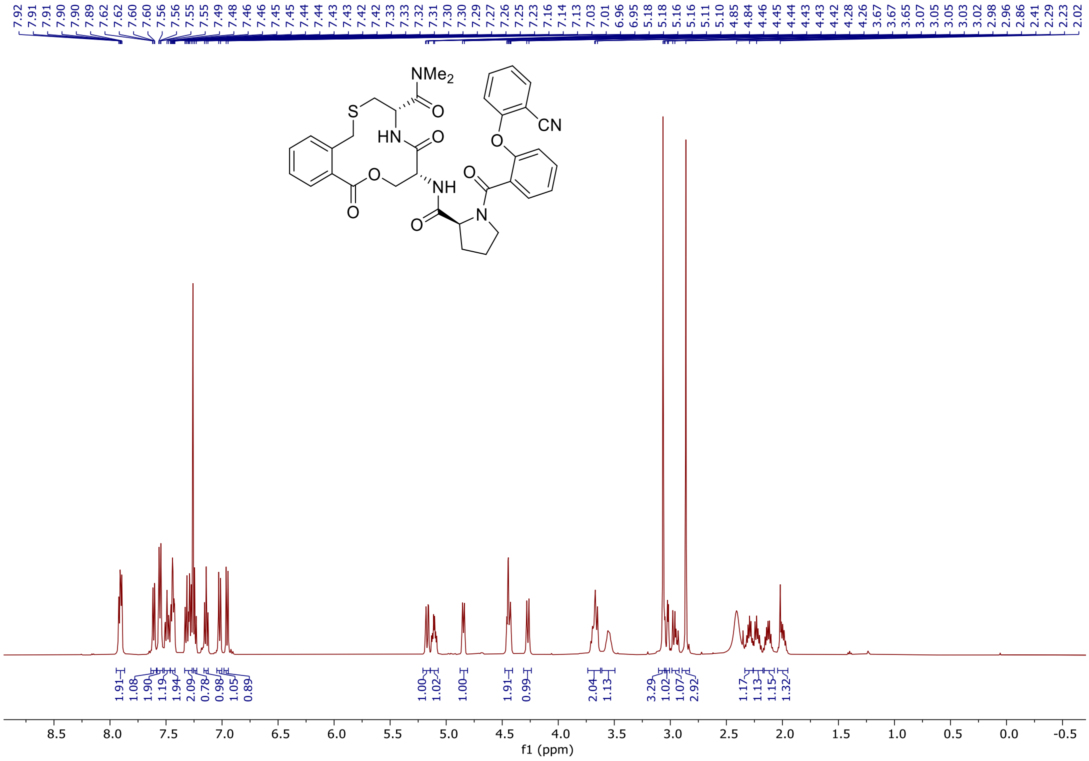

50 ^{13}C NMR (126 MHz, $CDCl_3$)

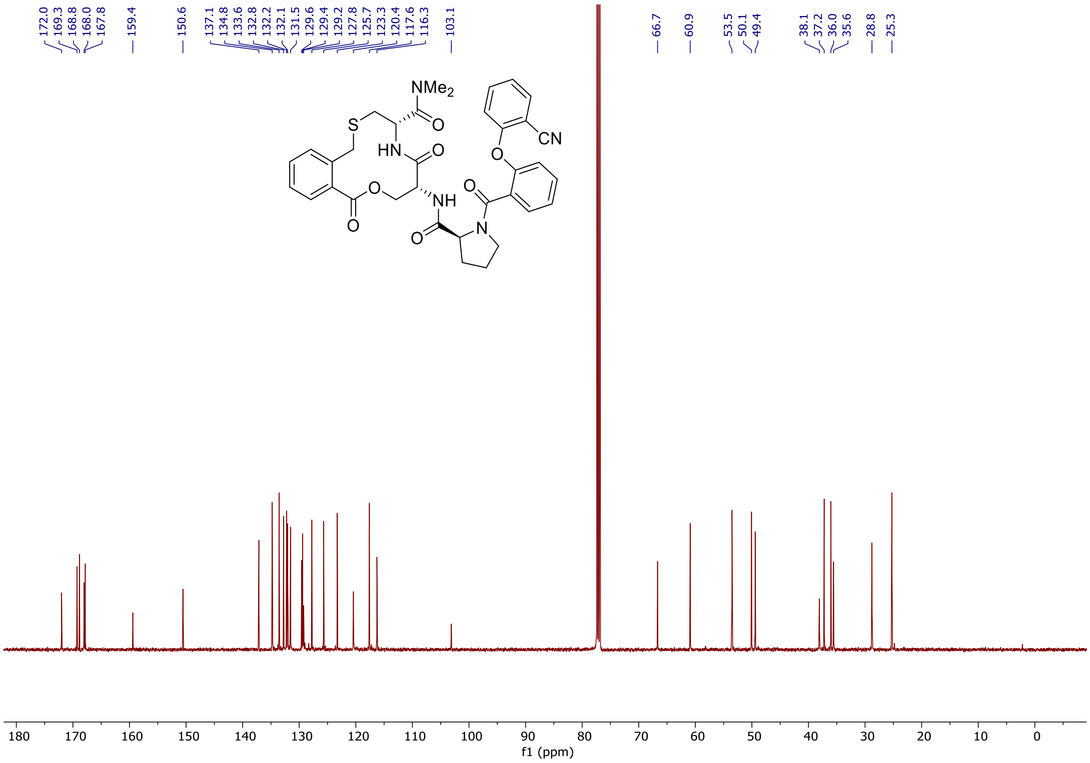

51 ^{1}H NMR (600 MHz, $CDCl_3$)

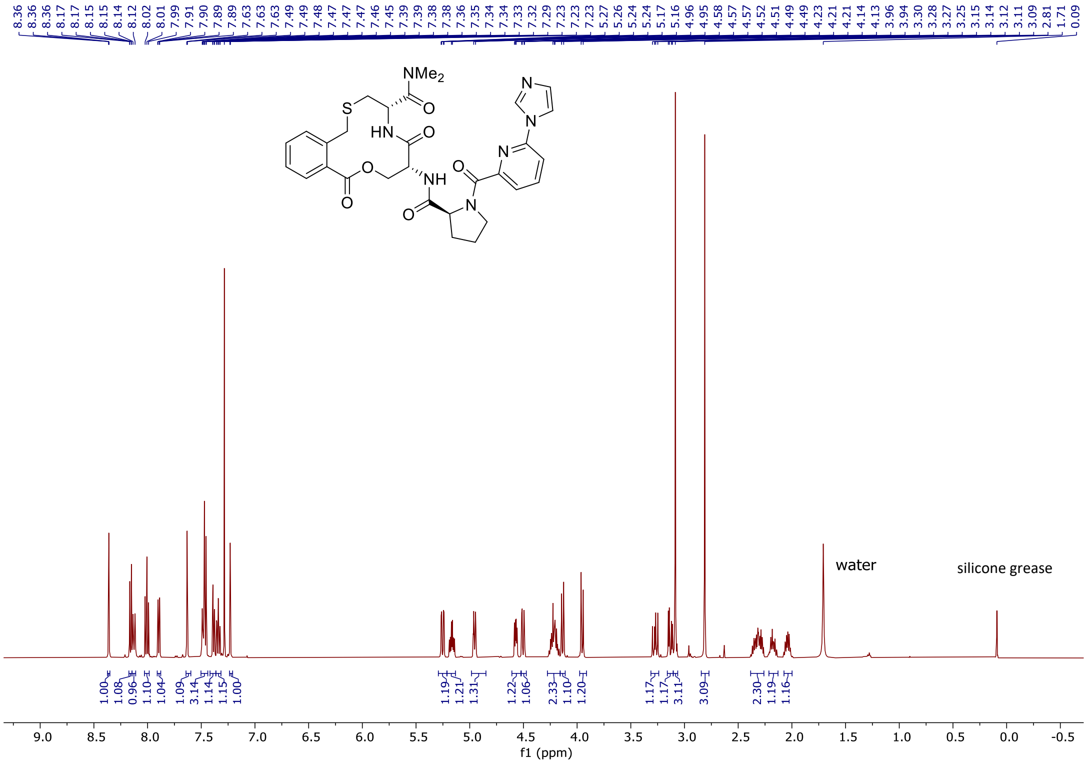

51 ^{13}C NMR (151 MHz, $CDCl_3$)

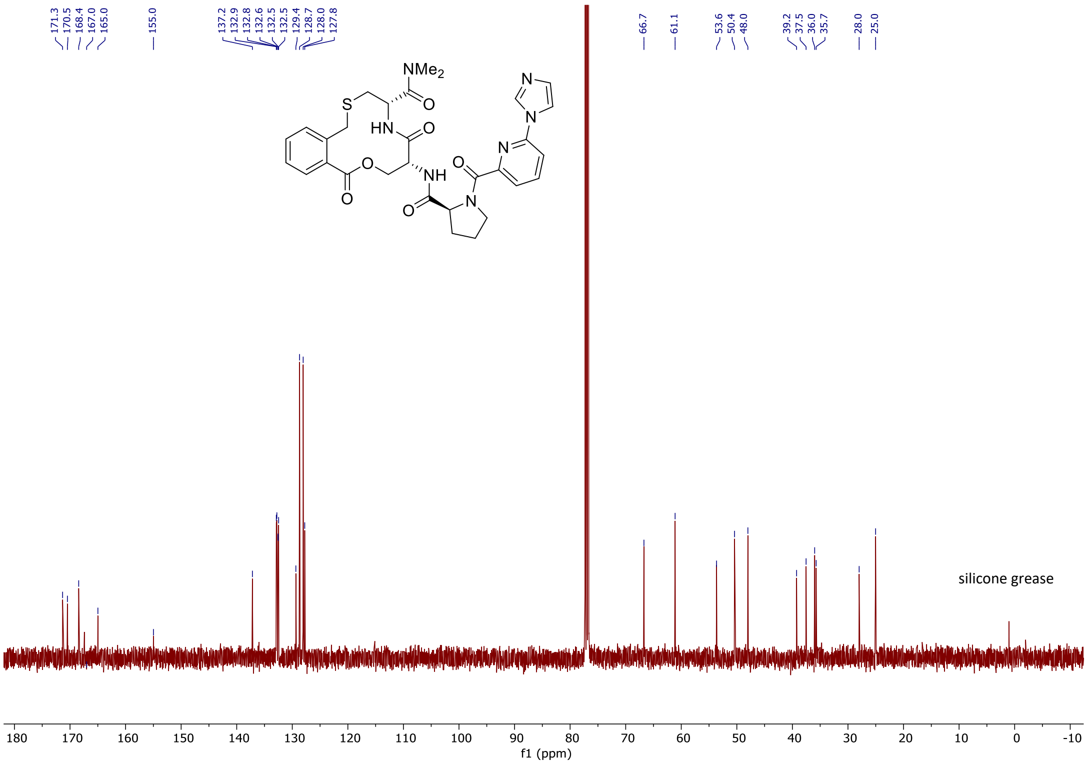

52 ^{1}H NMR (600 MHz, $CDCl_3$)

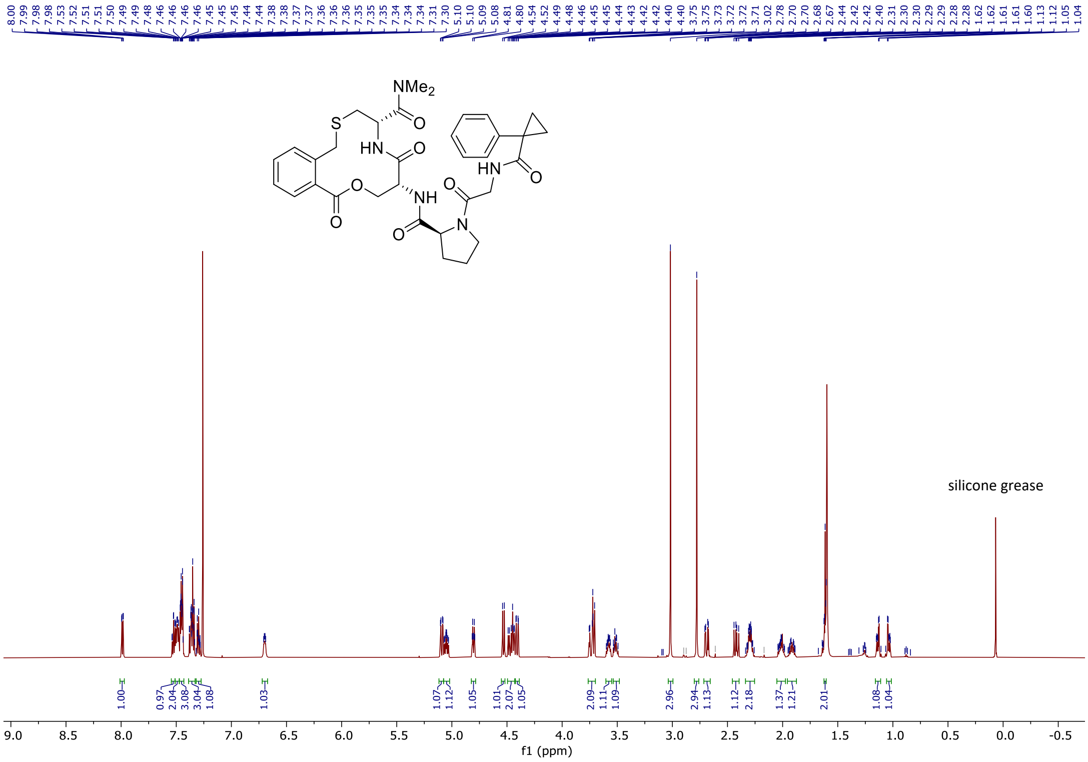

52 ^{13}C NMR (151 MHz, $CDCl_3$)

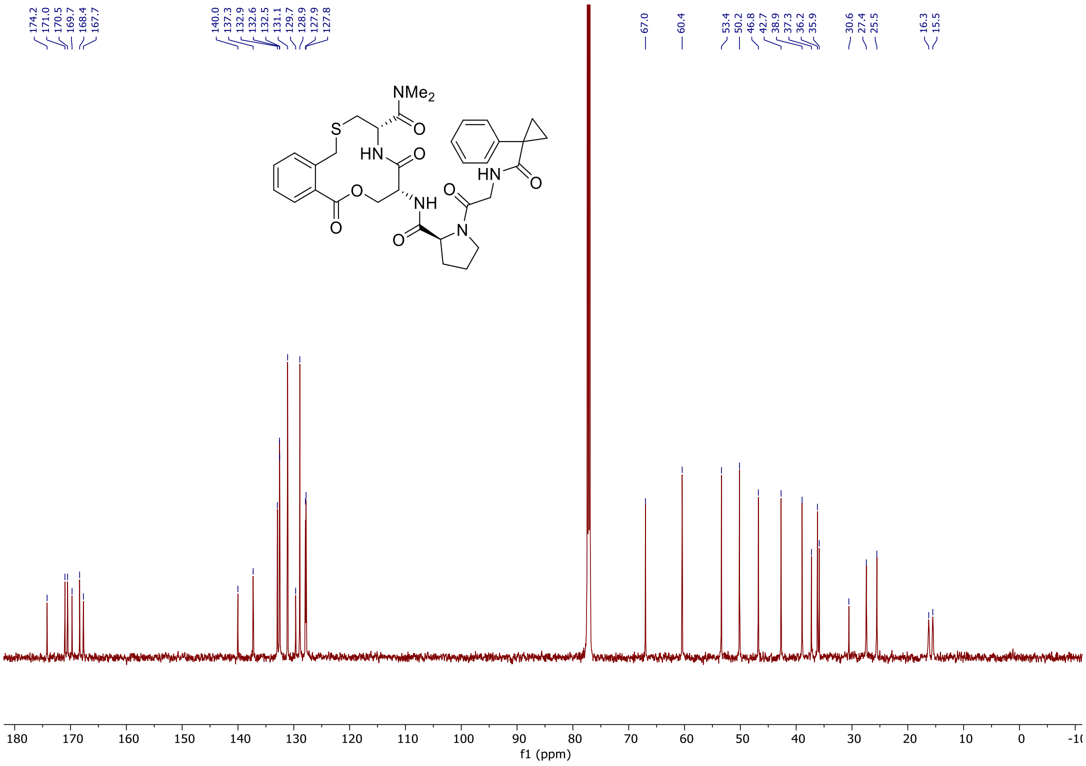

53 ^{1}H NMR (500 MHz, $CDCl_3$)

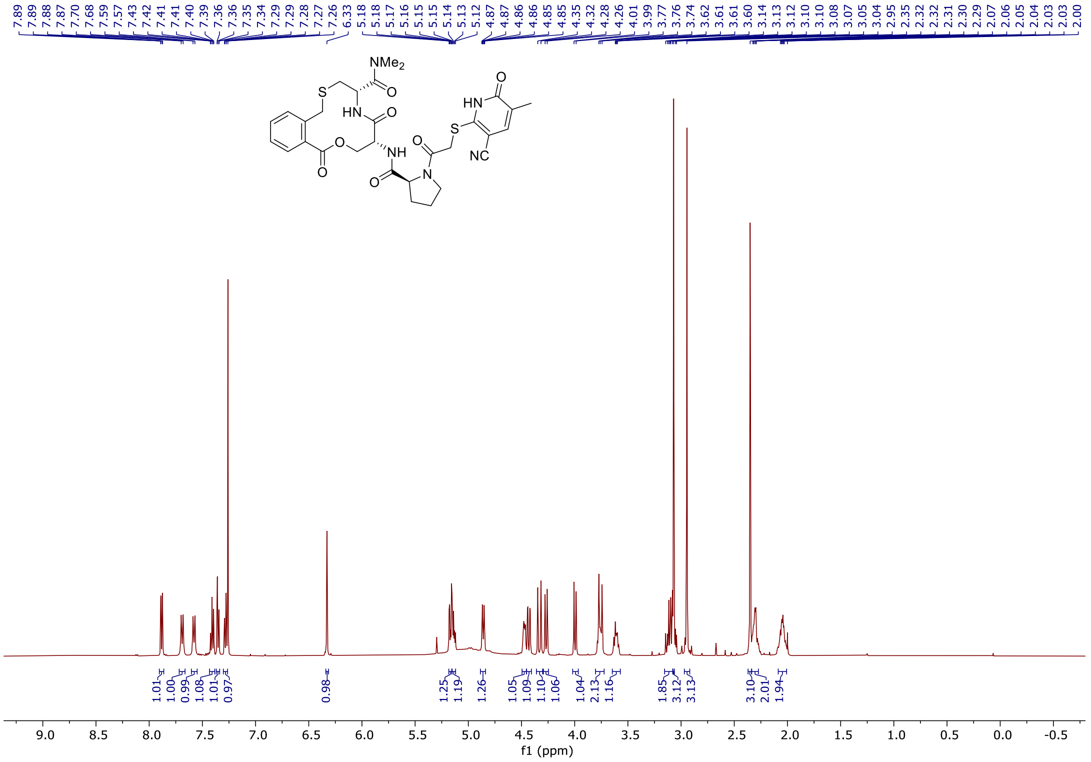

53 ^{13}C NMR (126 MHz, $CDCl_3$)

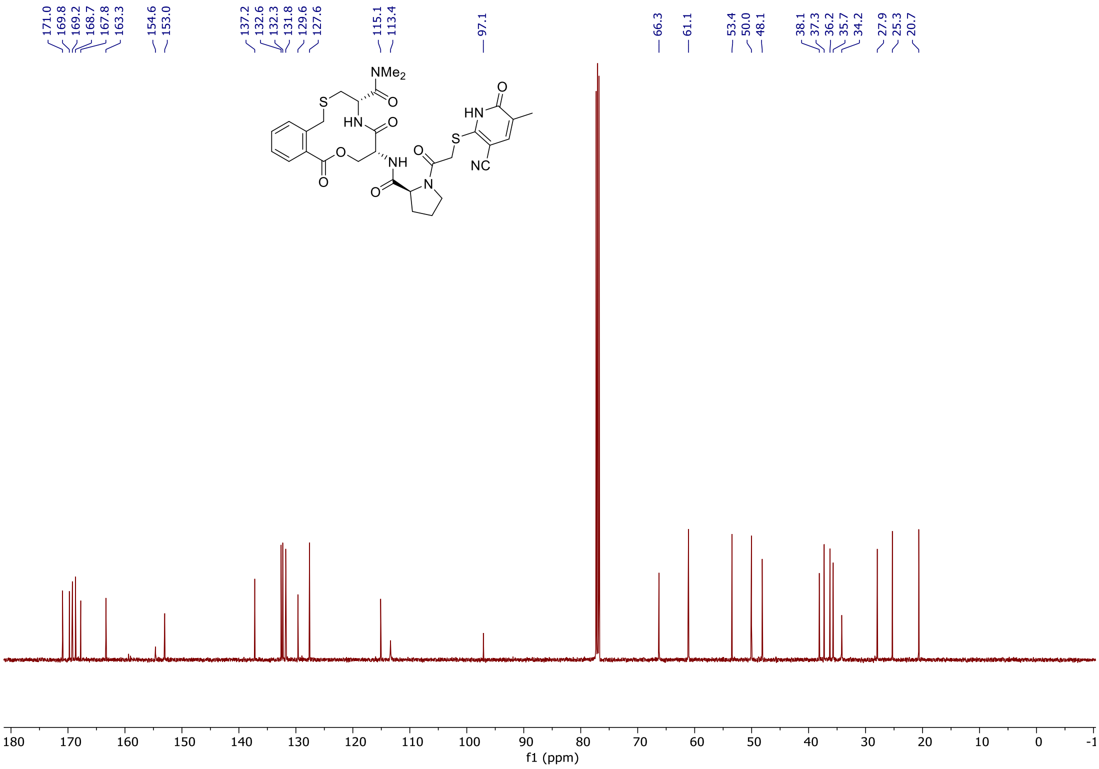

54 ^{1}H NMR (500 MHz, $CDCl_3$)

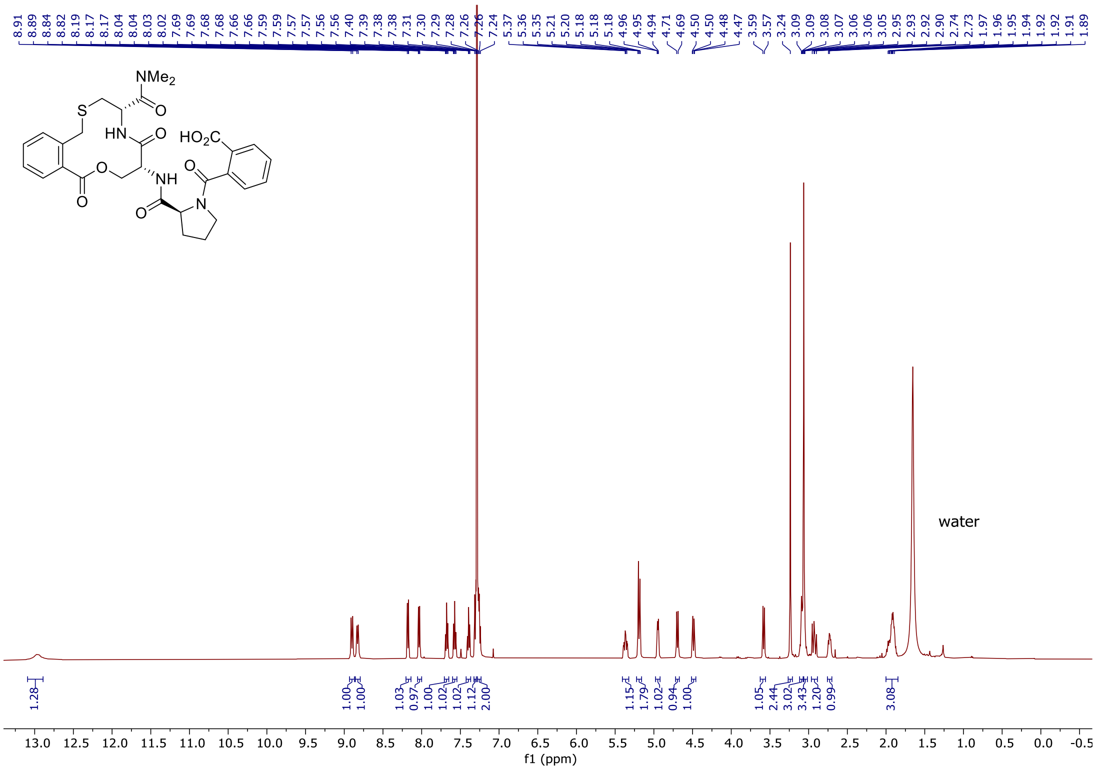

54 ^{13}C NMR (126 MHz, $CDCl_3$)

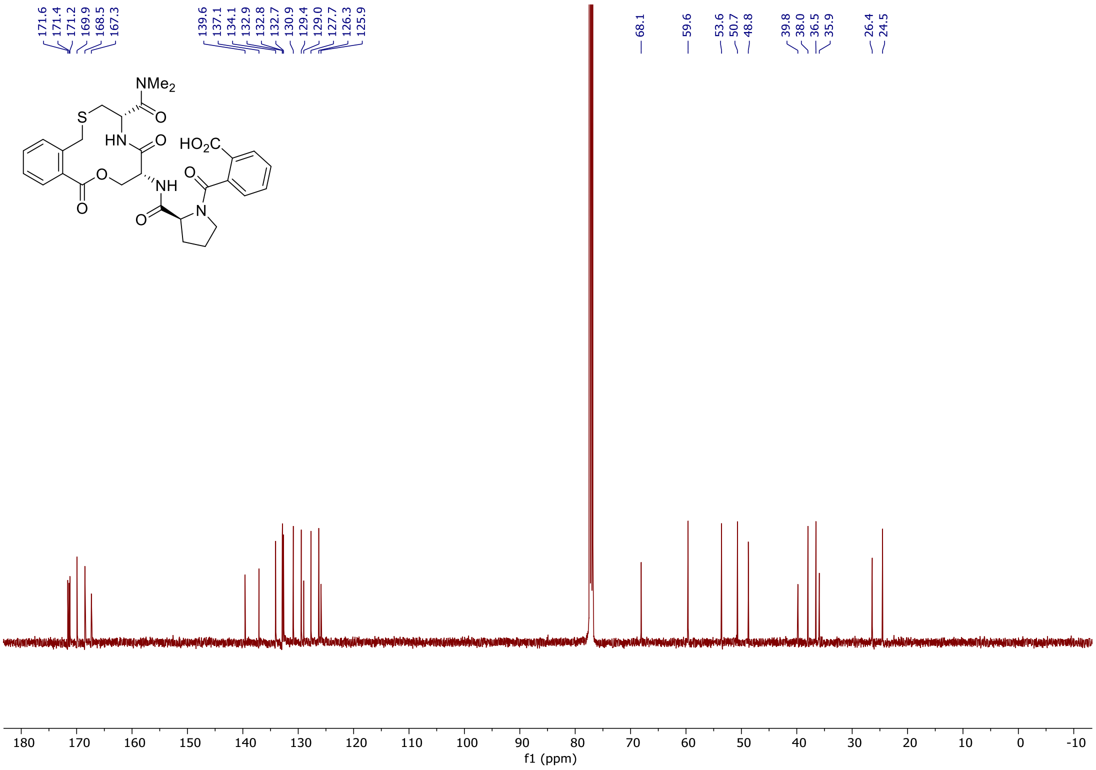

55 ^{1}H NMR (500 MHz, $CDCl_3$)

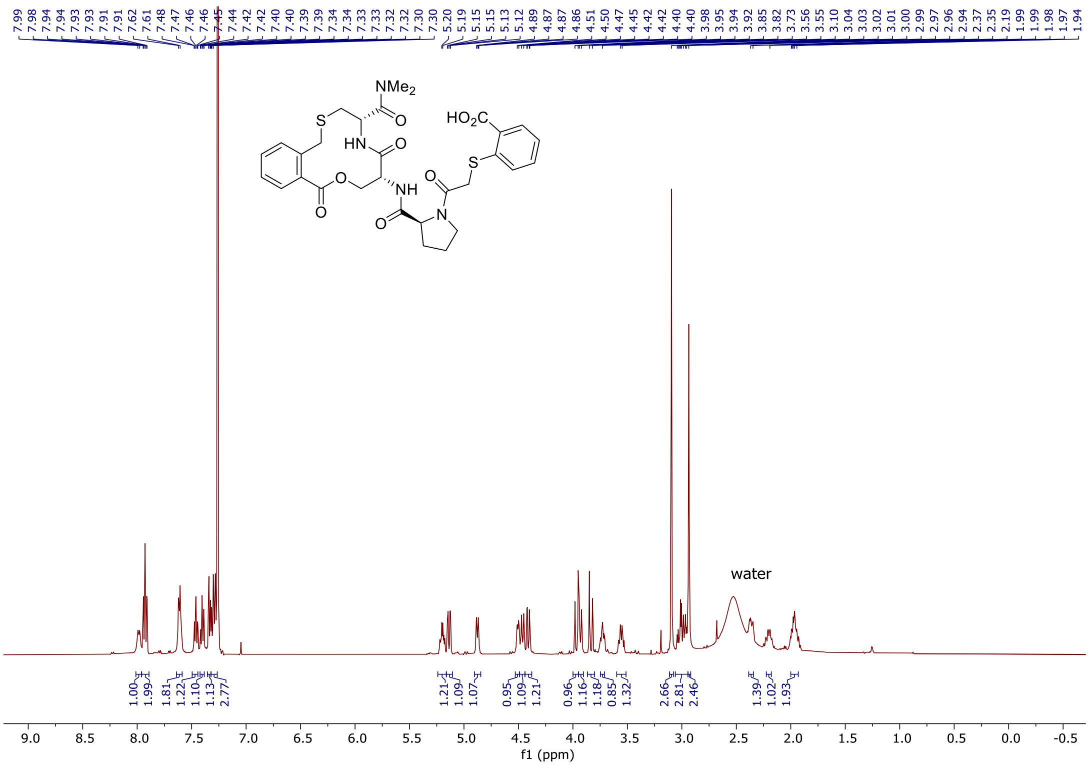

55 ^{13}C NMR (126 MHz, $CDCl_3$)

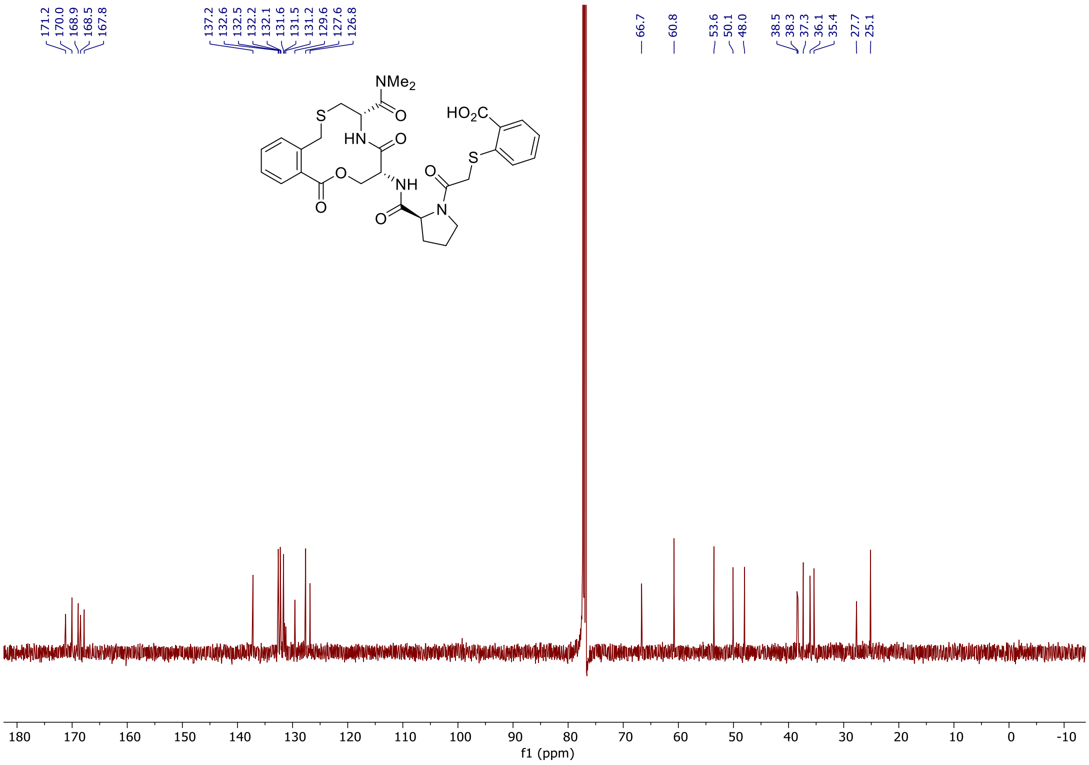

56 ^{1}H NMR (500 MHz, DMSO-d_6)

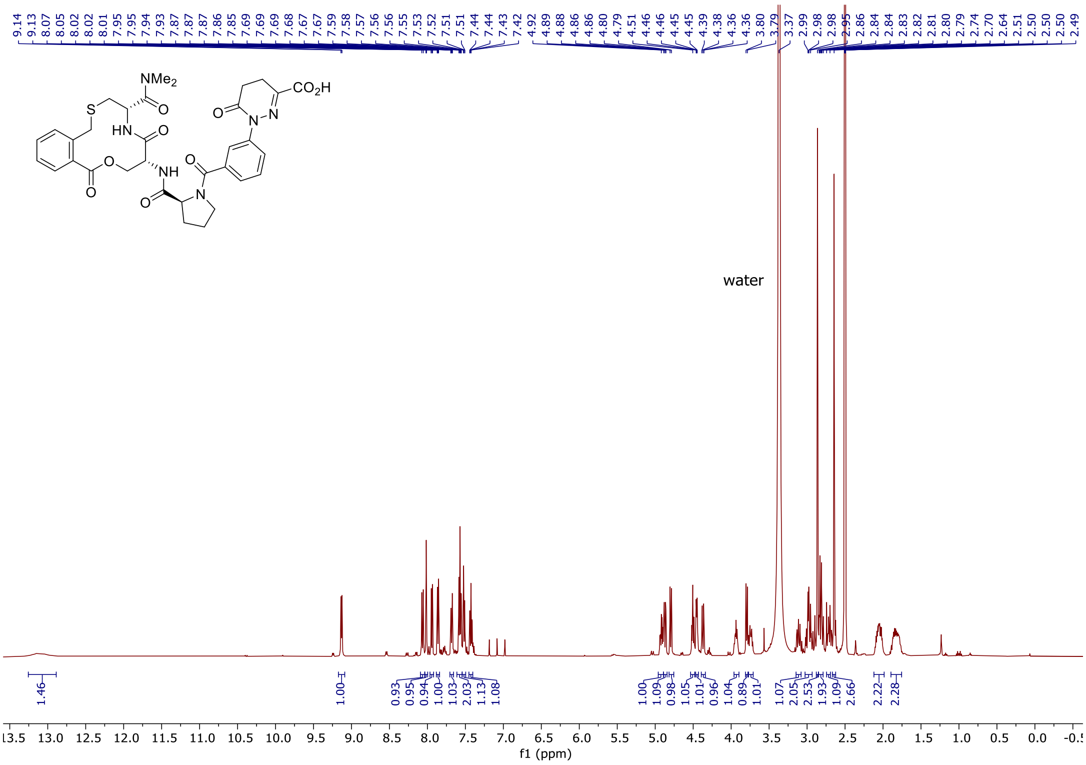

56 ^{13}C NMR (126 MHz, DMSO-d_6)

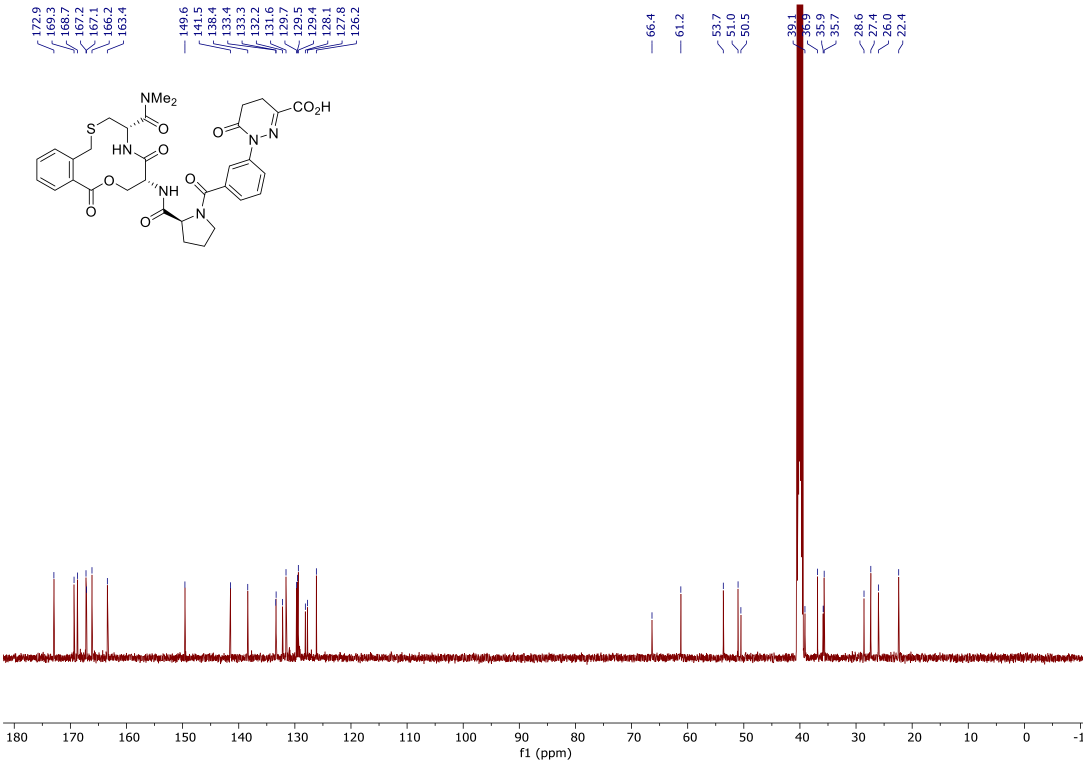

57 ^{1}H NMR (500 MHz, $CDCl_3$)

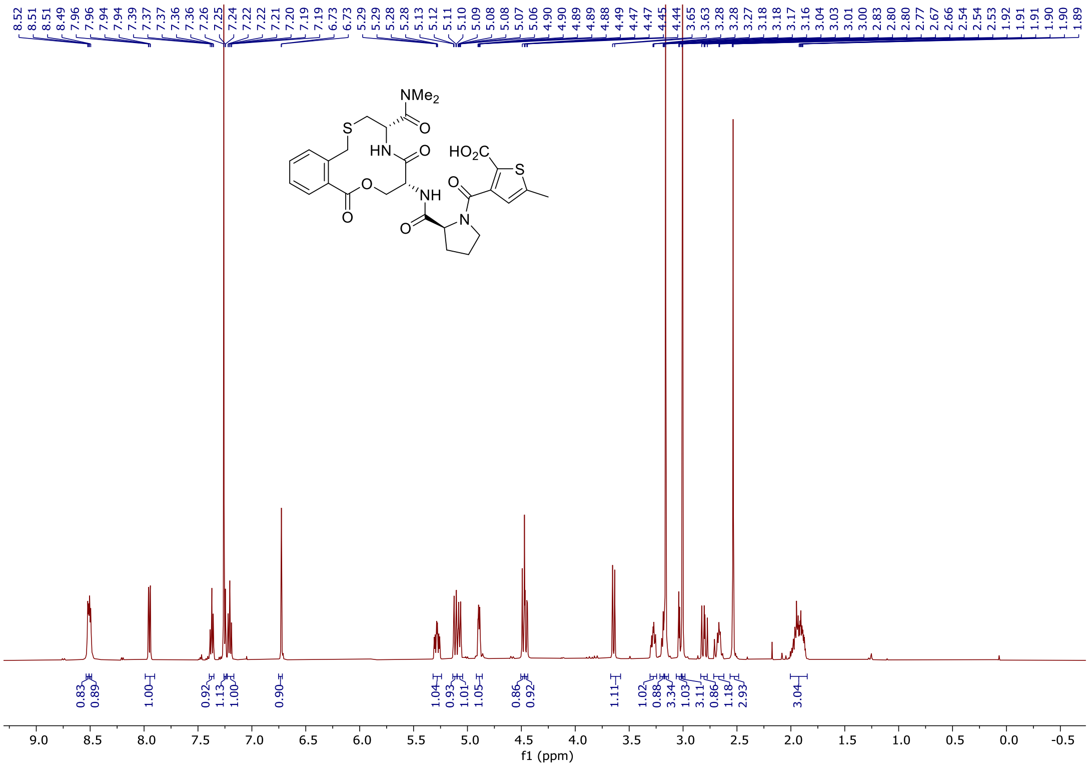

57 ^{13}C NMR (126 MHz, $CDCl_3$)

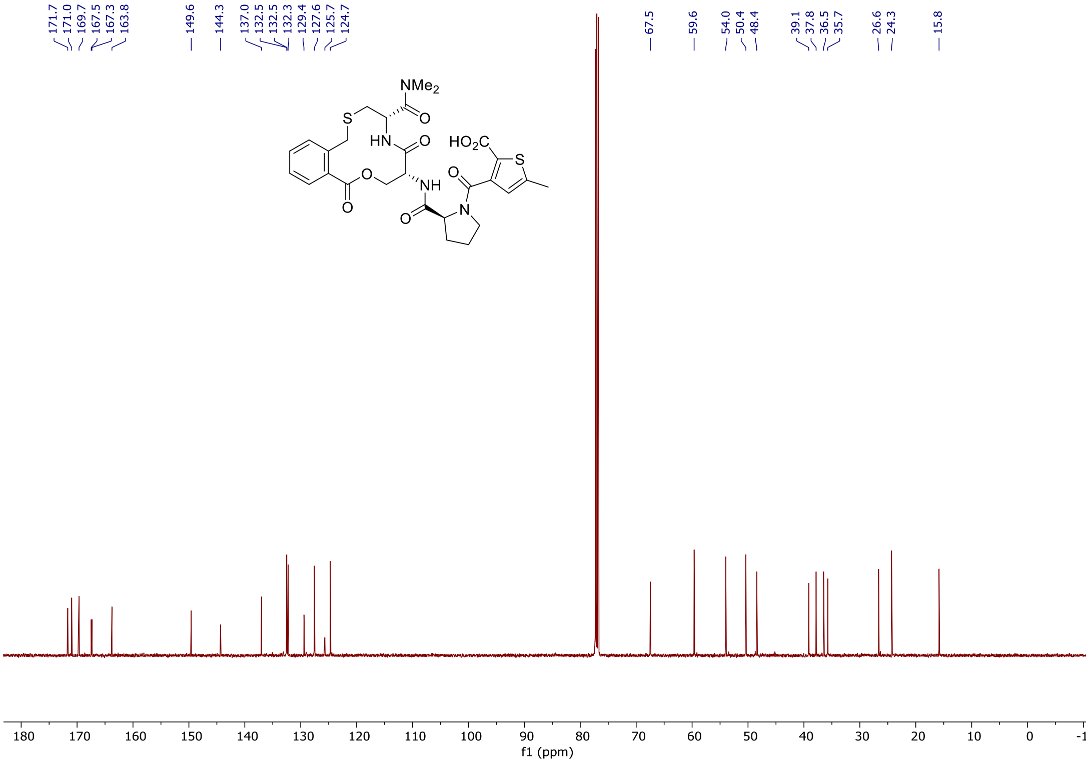

58 ^{1}H NMR (500 MHz, $CDCl_3$)

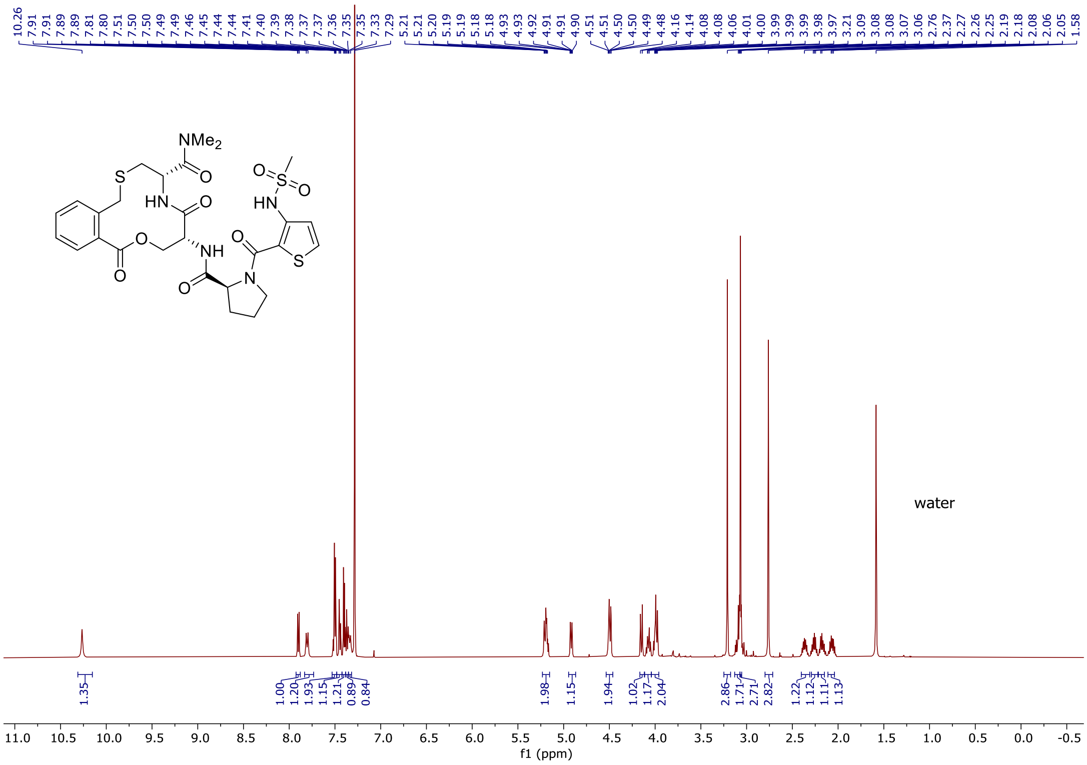

58 ^{13}C NMR (126 MHz, $CDCl_3$)

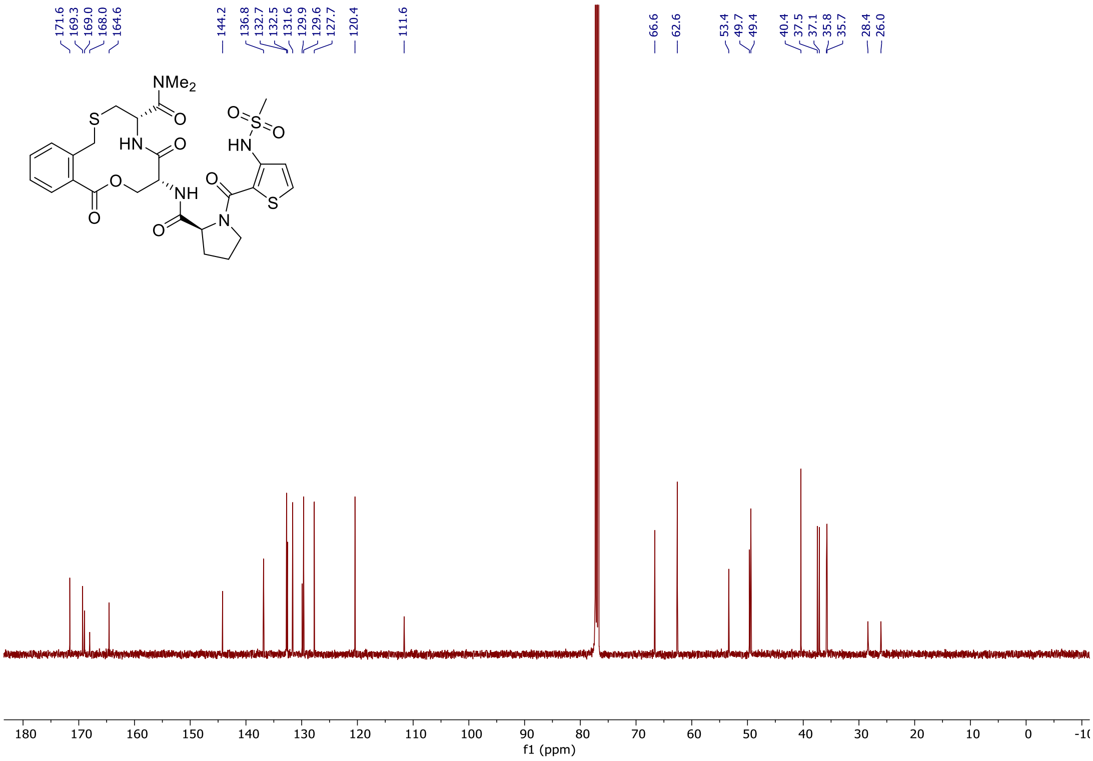

59 ^{1}H NMR (500 MHz, $CDCl_3$)

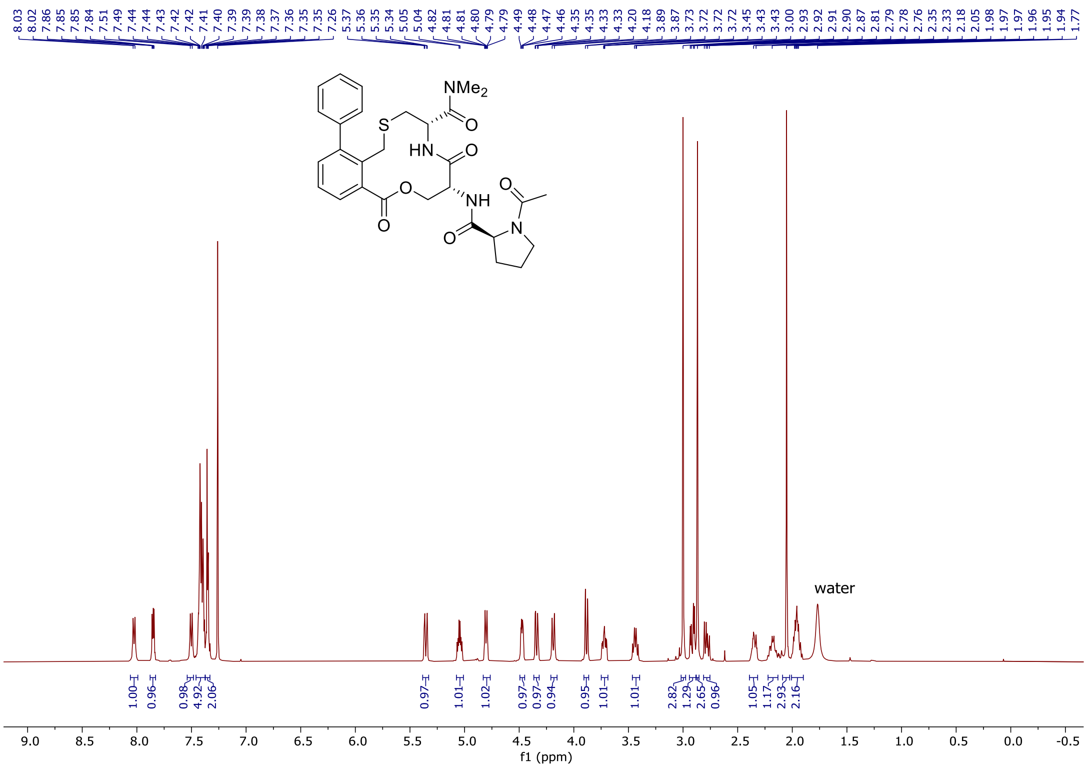

59 ^{13}C NMR (126 MHz, $CDCl_3$)

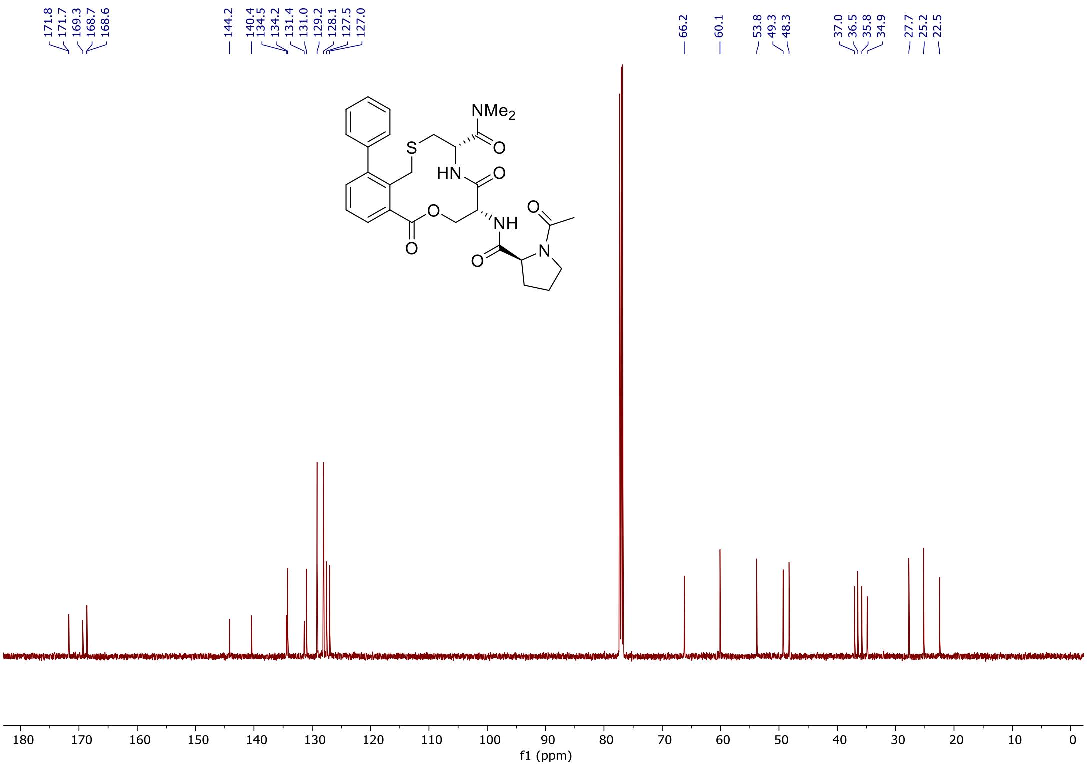

60 ^{1}H NMR (500 MHz, $CDCl_3$)

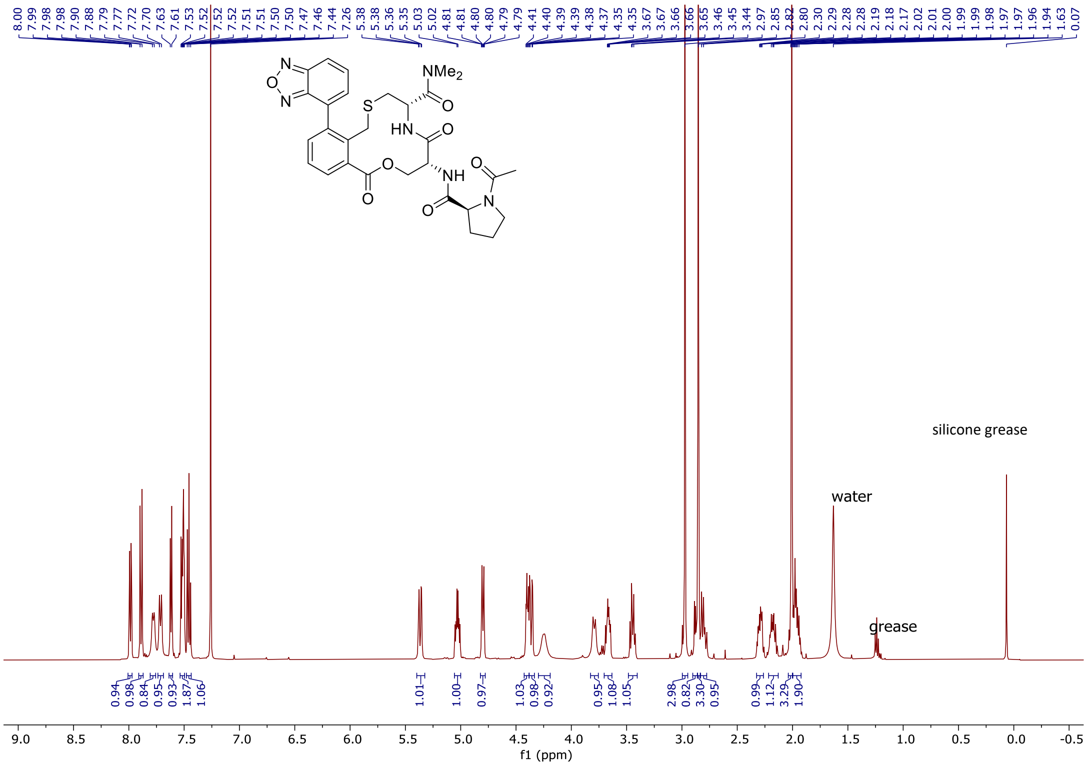

60 ^{13}C NMR (126 MHz, $CDCl_3$)

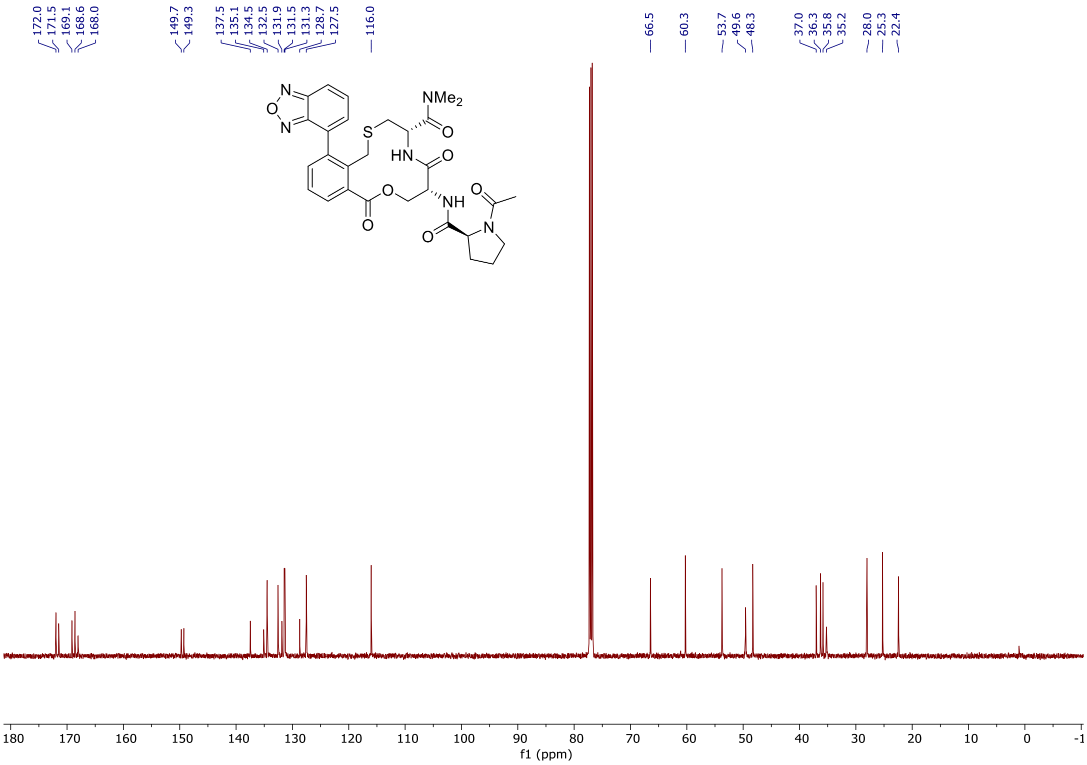

61 ^{1}H NMR (500 MHz, $CDCl_3$)

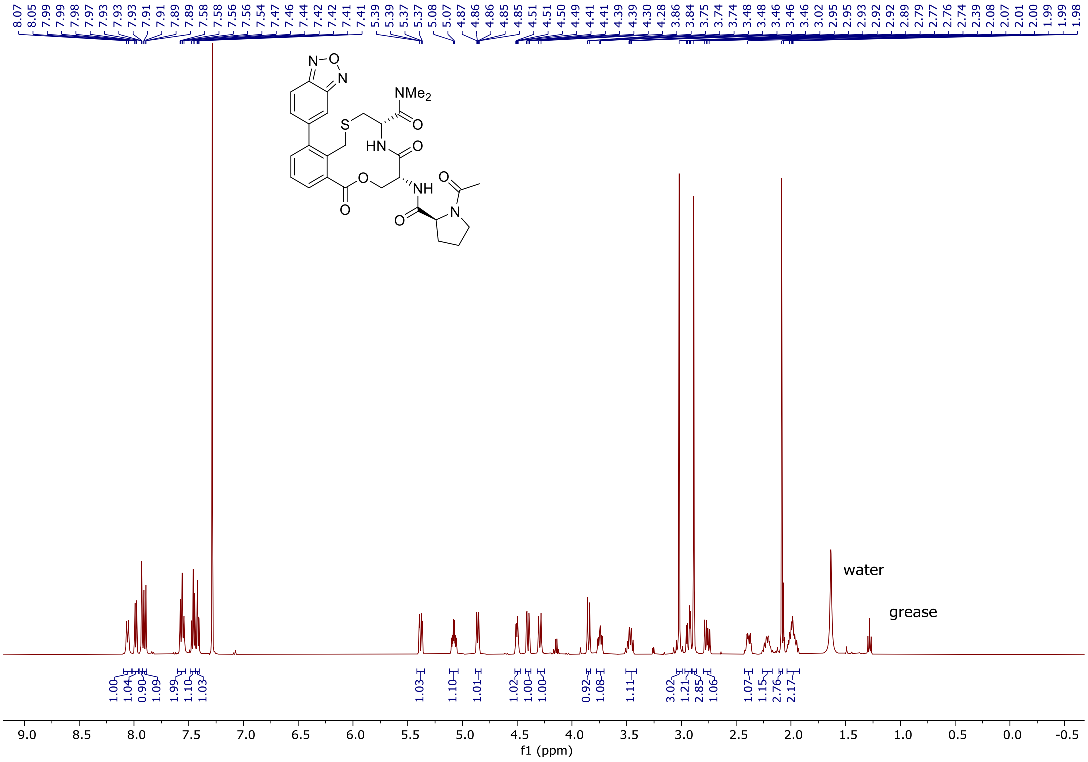

61 ^{13}C NMR (126 MHz, $CDCl_3$)

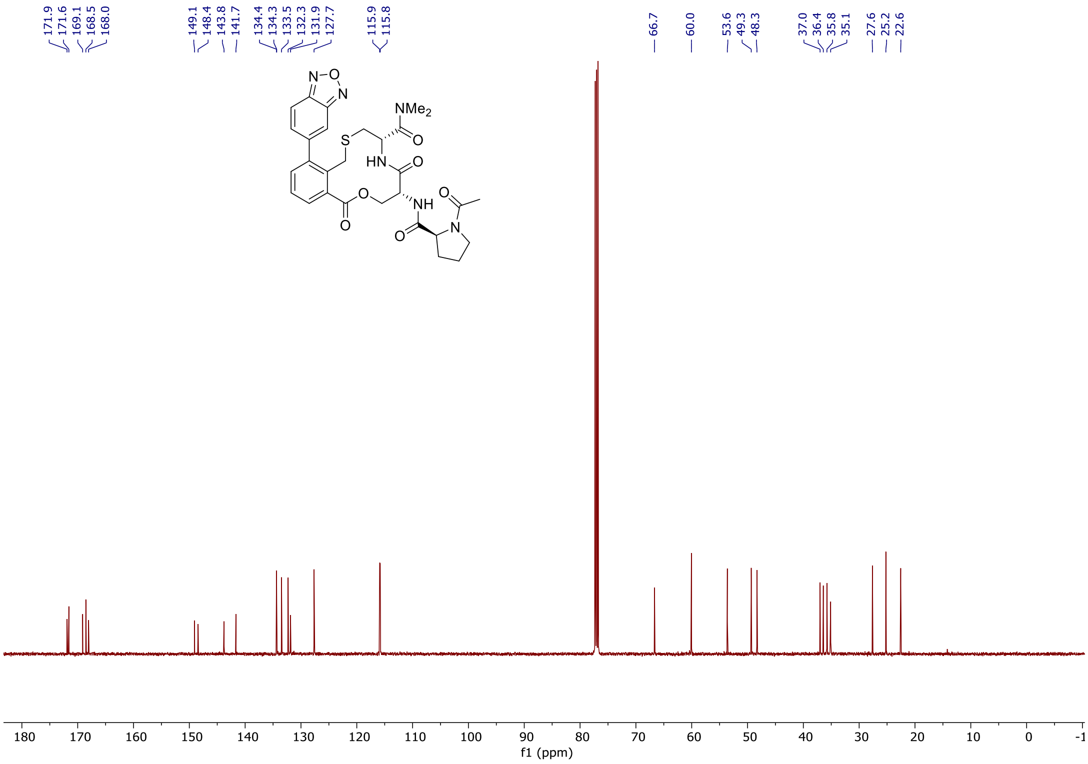

62 ^{1}H NMR (500 MHz, CDCl$_3$)

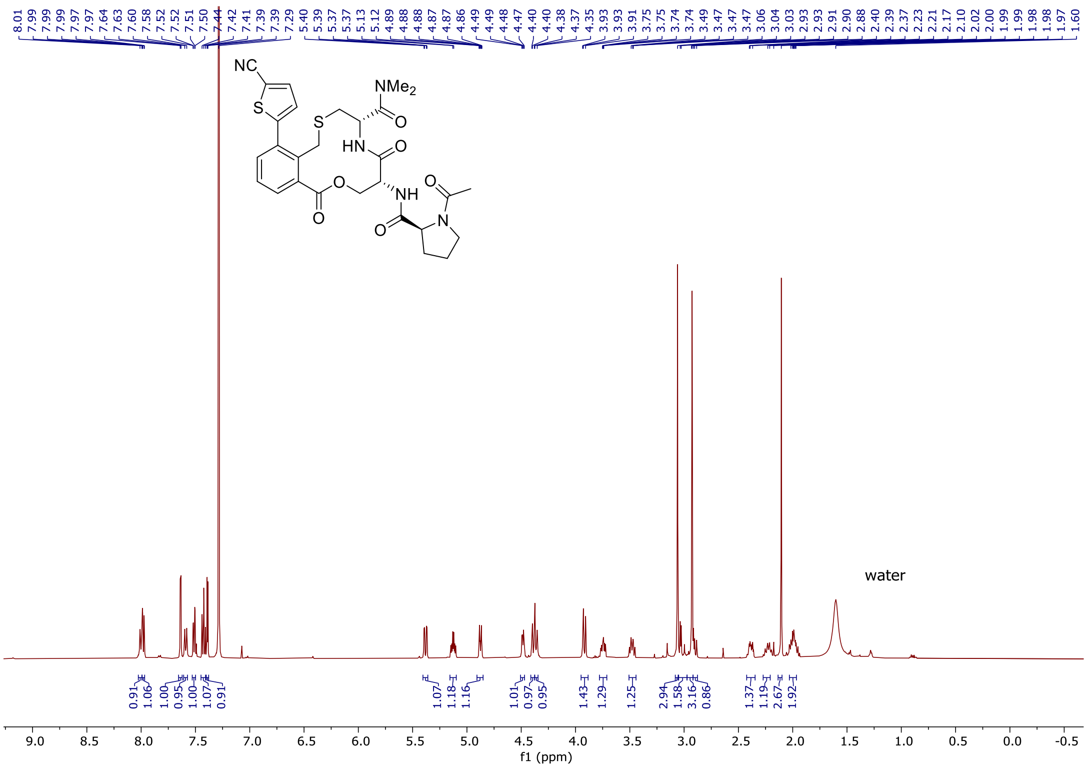

62 ^{13}C NMR (126 MHz, CDCl$_3$)

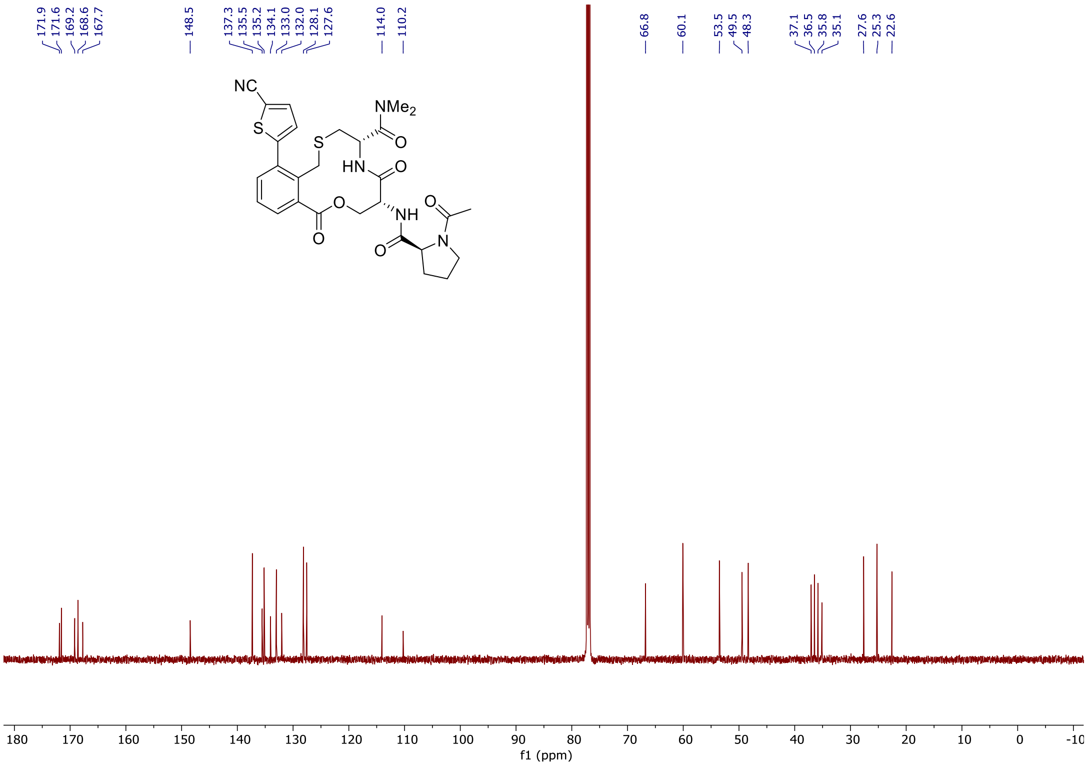

63 ^{1}H NMR (500 MHz, CDCl$_3$)

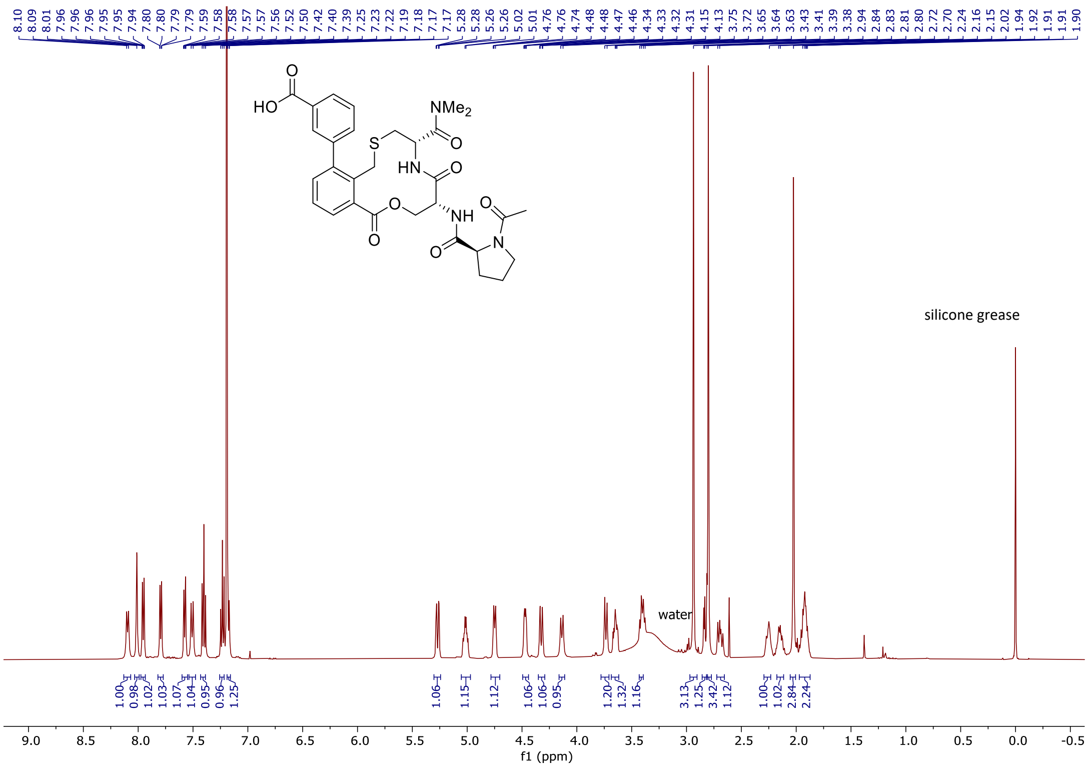

63 ^{13}C NMR (126 MHz, CDCl$_3$)

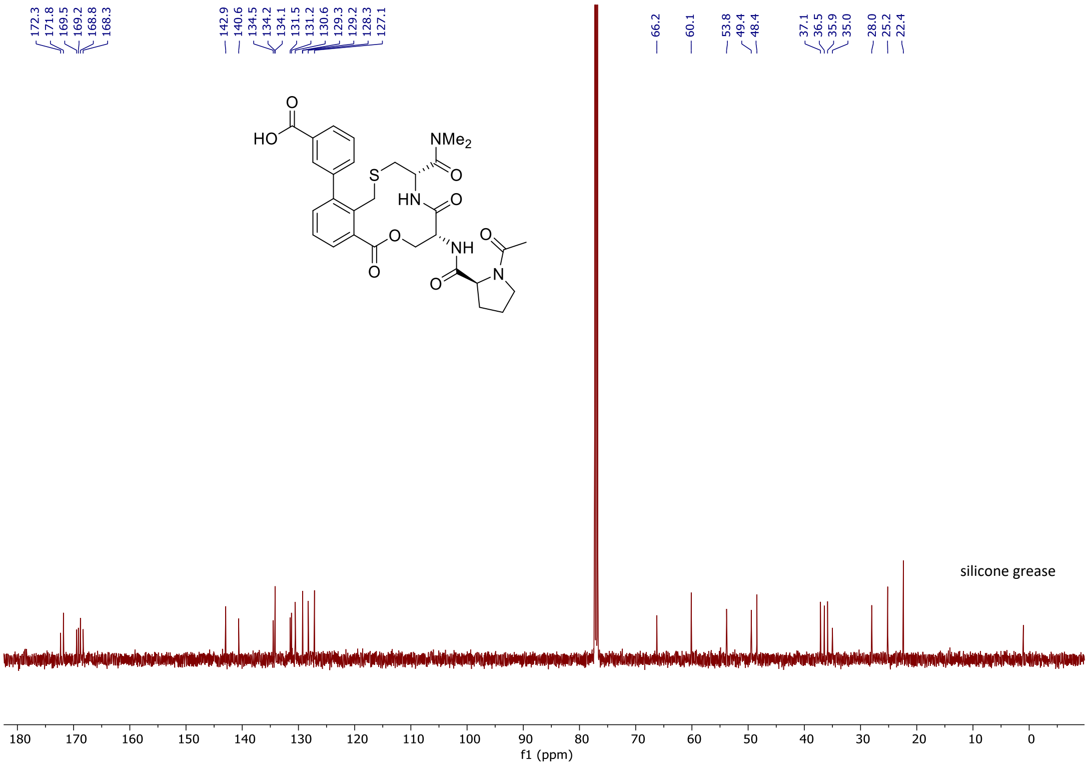

64 ^{1}H NMR (500 MHz, CDCl$_3$)

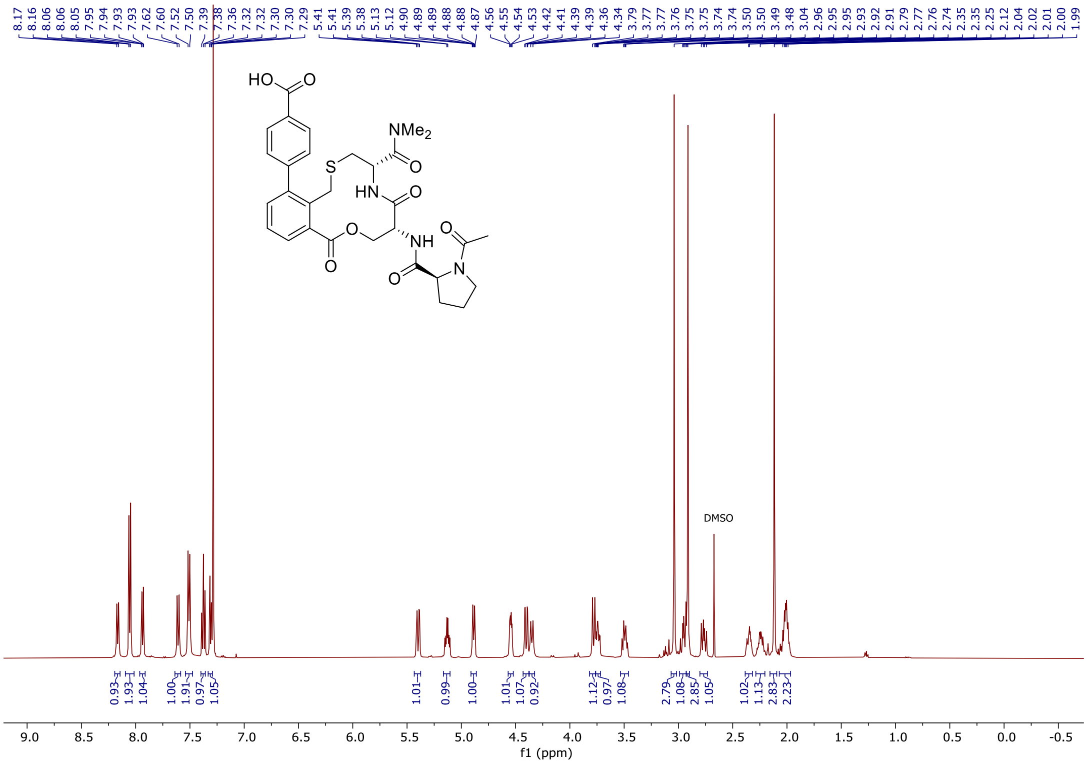

64 ^{1}H NMR (500 MHz, CDCl$_3$)

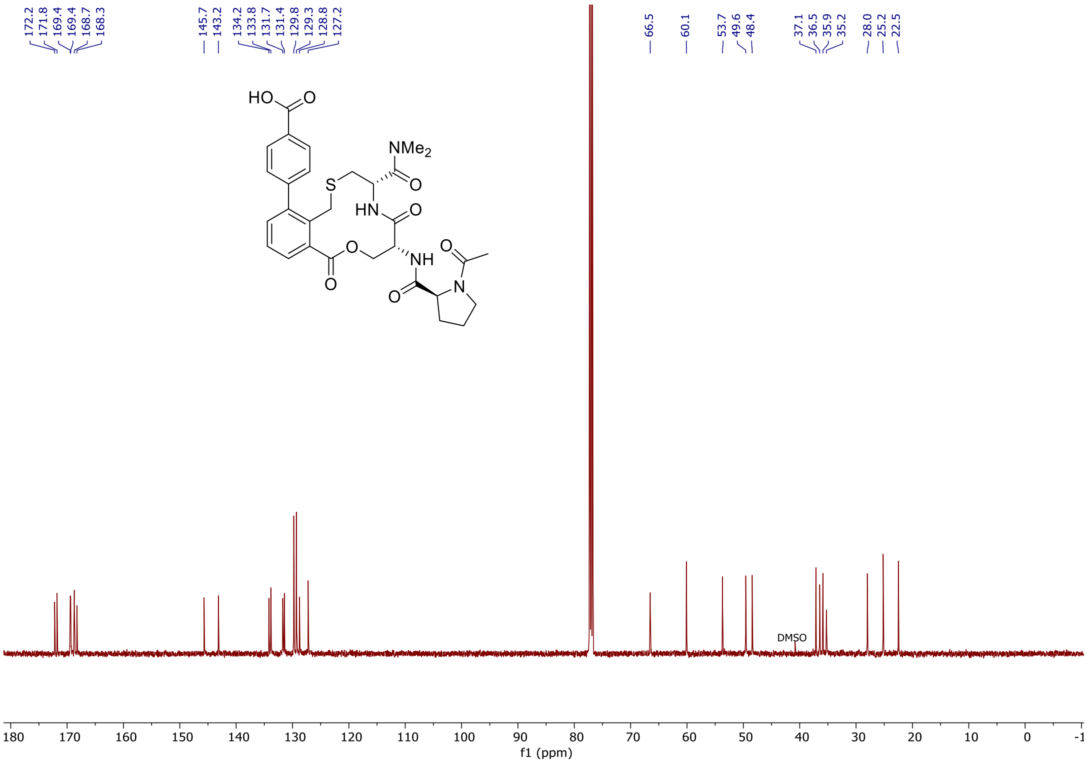

65 ^{1}H NMR (500 MHz, $CDCl_3$)

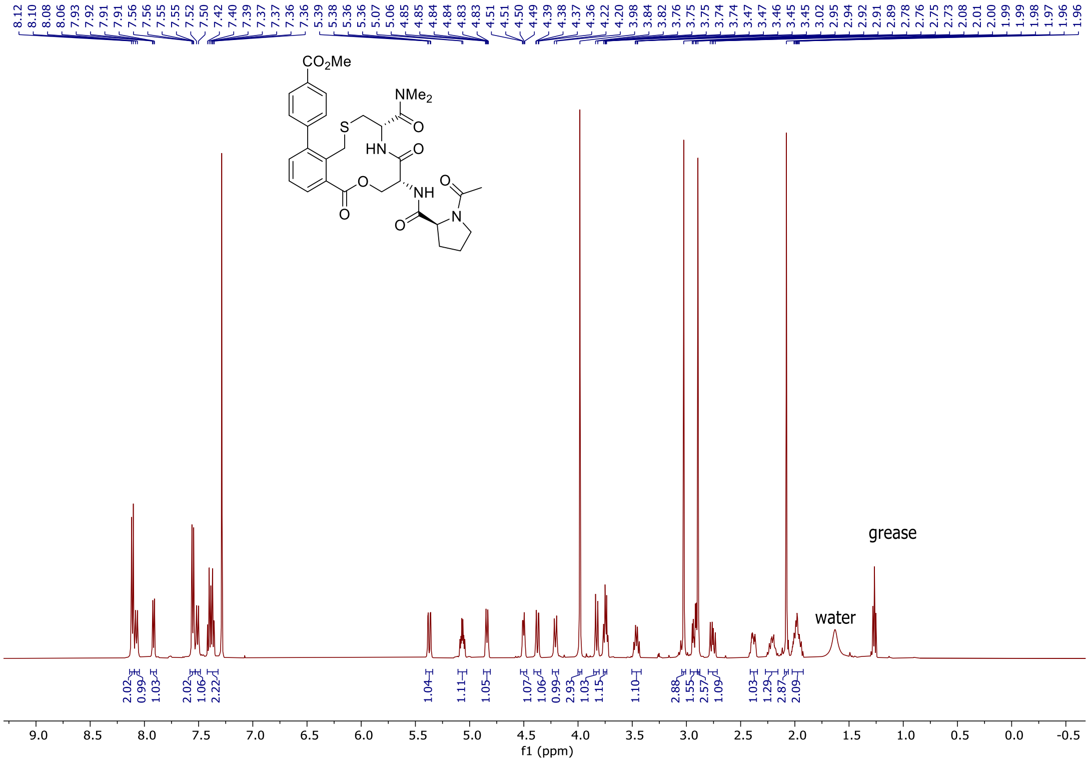

65 ^{13}C NMR (126 MHz, $CDCl_3$)

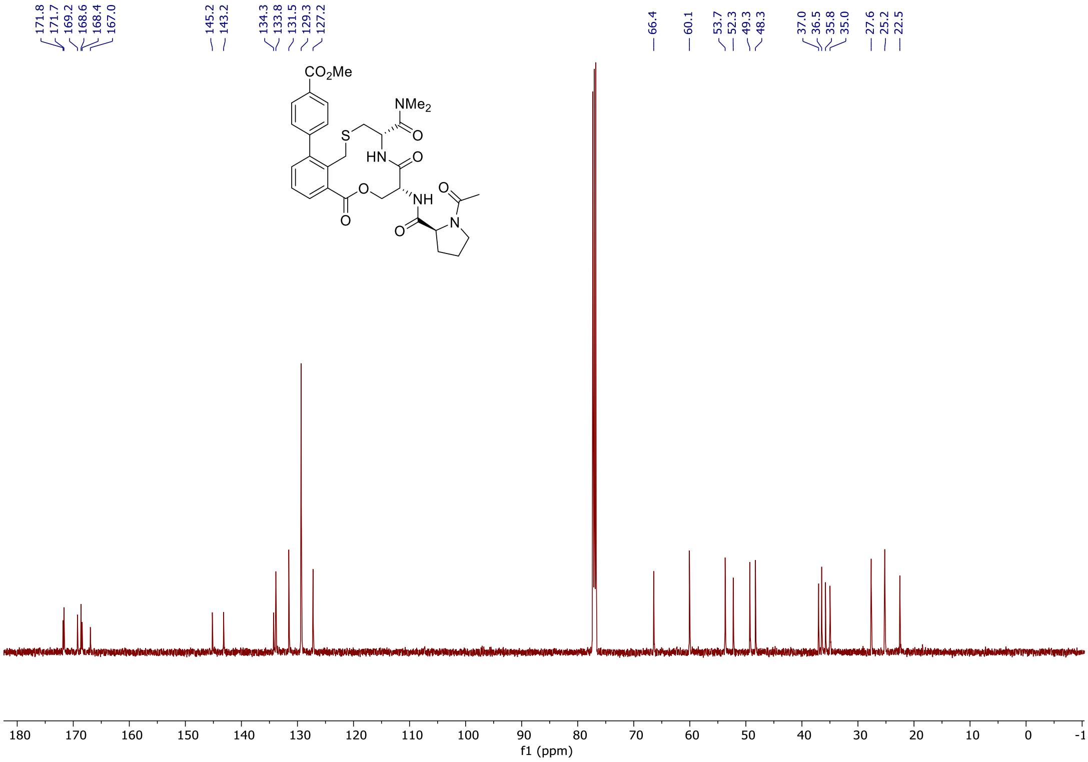

66 ^{1}H NMR (500 MHz, $CDCl_3$)

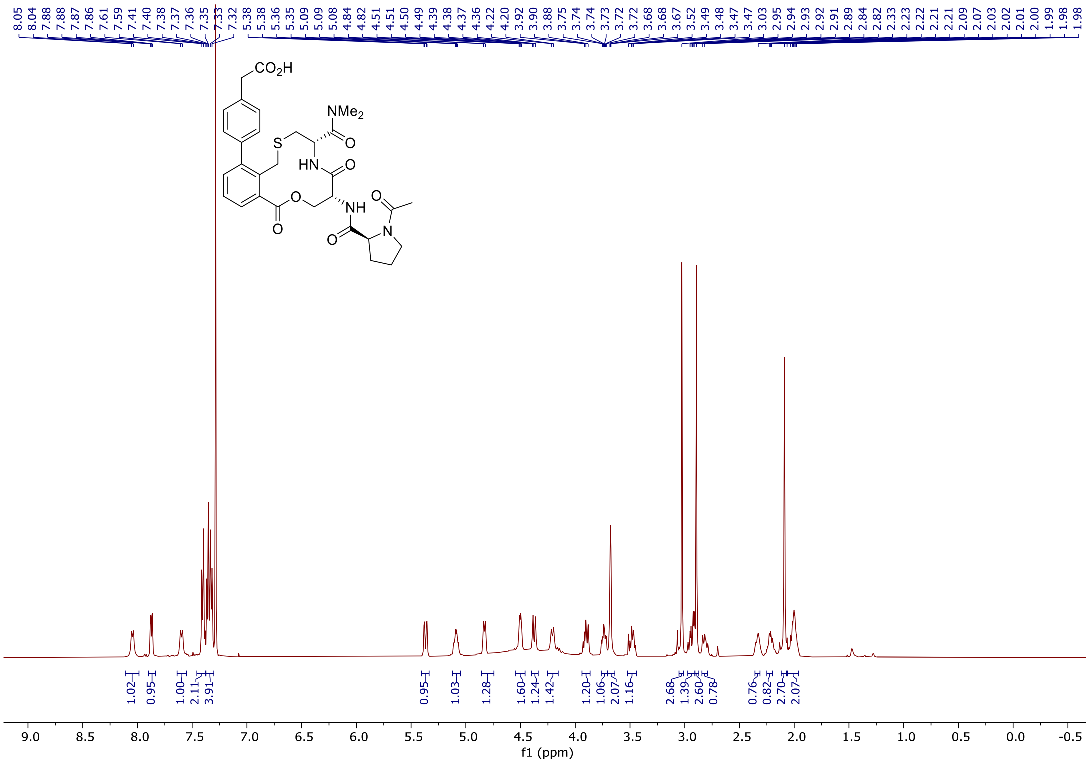

66 ^{13}C NMR (126 MHz, $CDCl_3$)

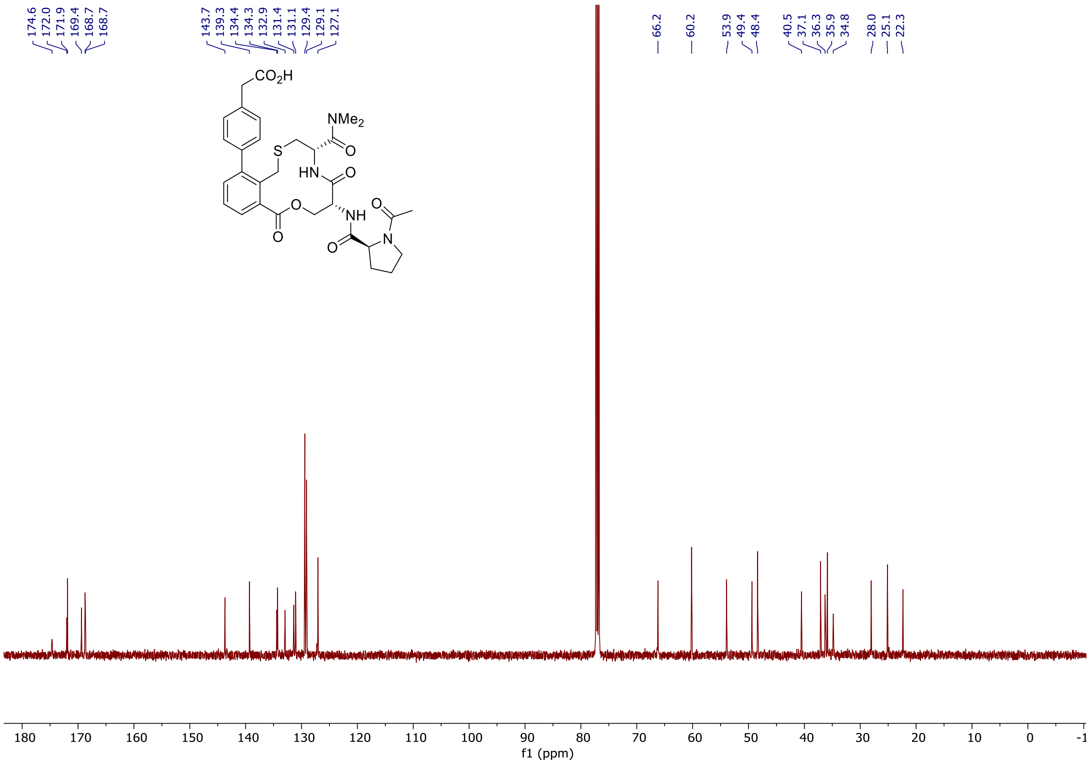

67 ^{1}H NMR (500 MHz, CDCl$_3$)

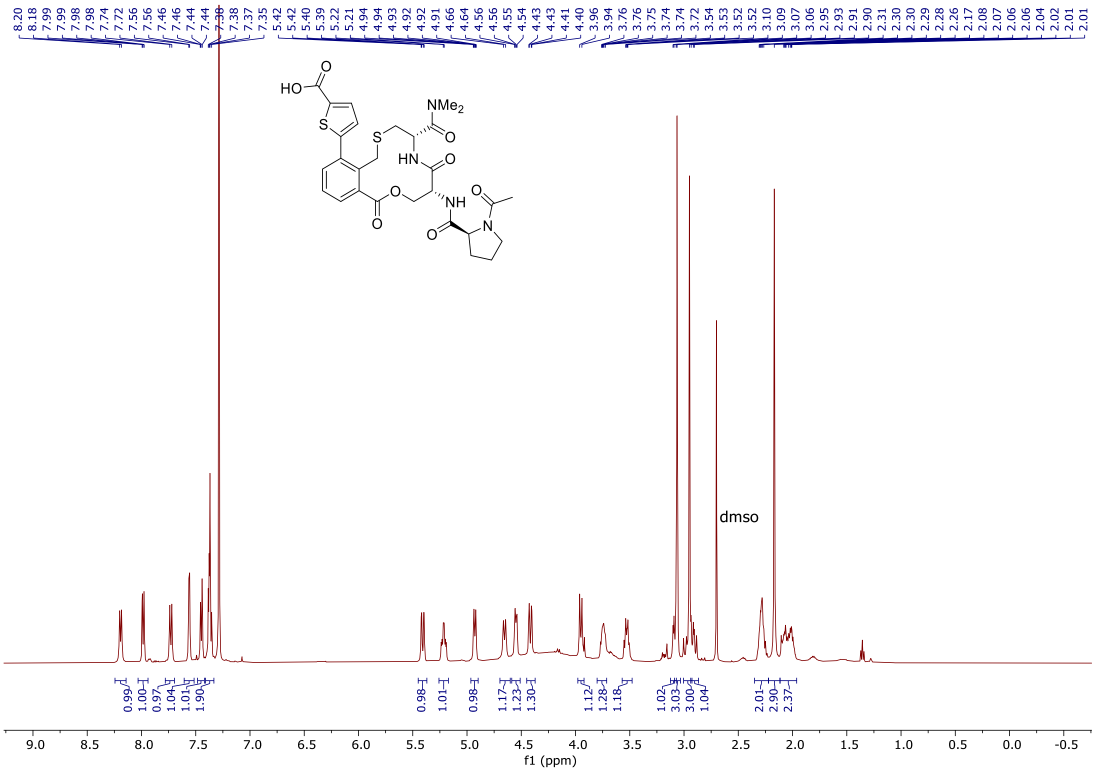

67 ^{1}H NMR (500 MHz, CDCl$_3$)

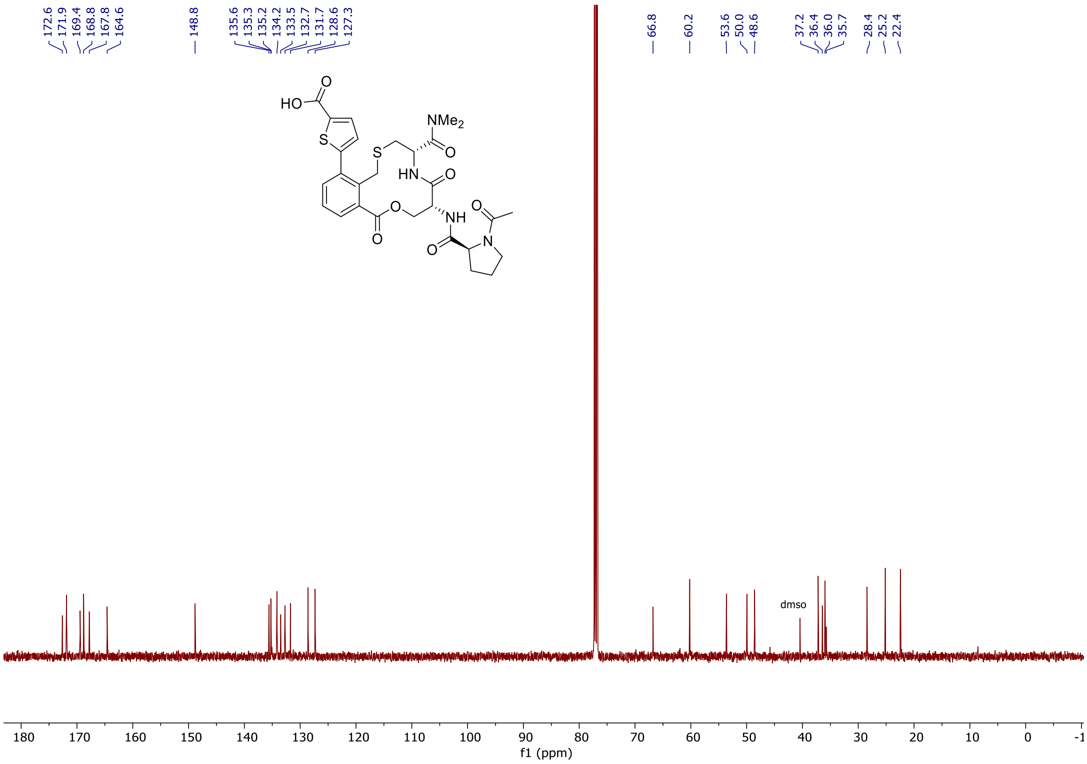

68 ^{1}H NMR (500 MHz, $CDCl_3$)

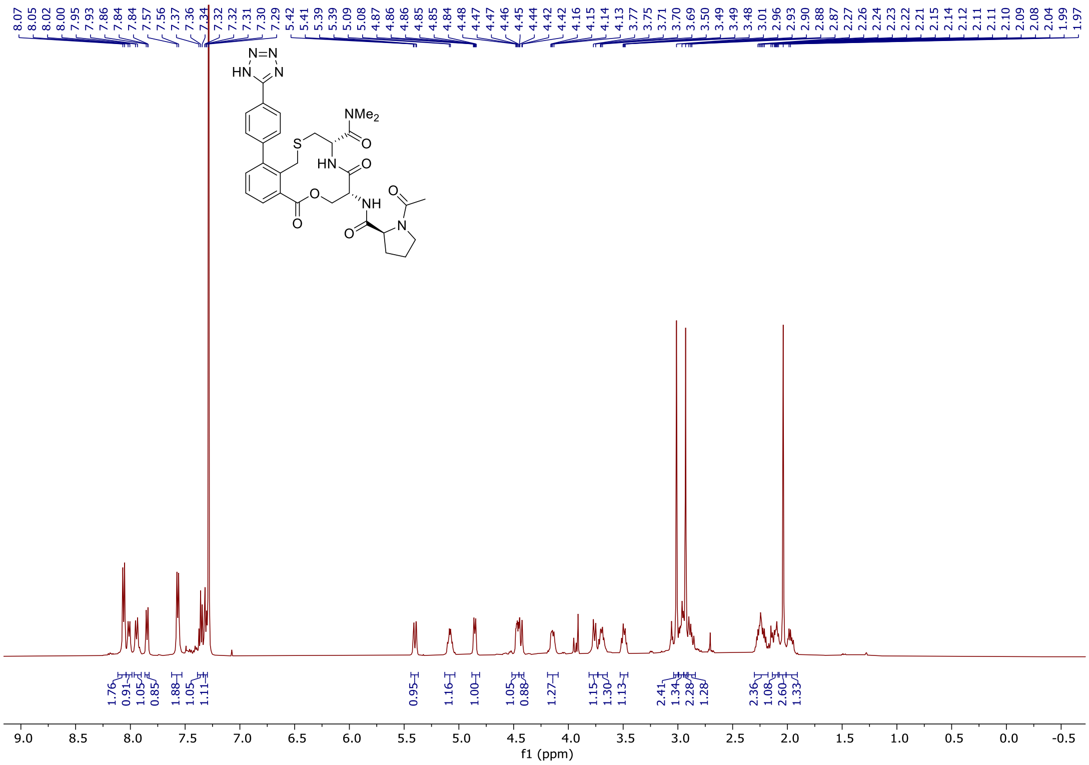

68 ^{13}C NMR (126 MHz, $CDCl_3$)

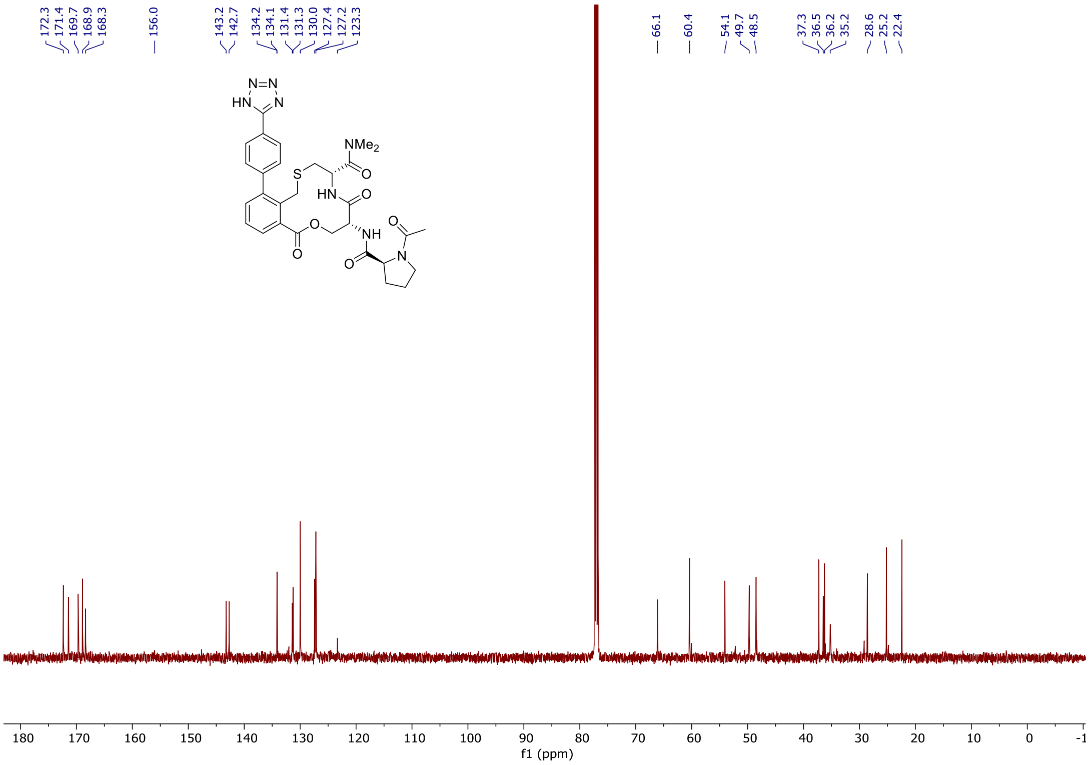

69 ^{1}H NMR (500 MHz, CDCl$_3$)

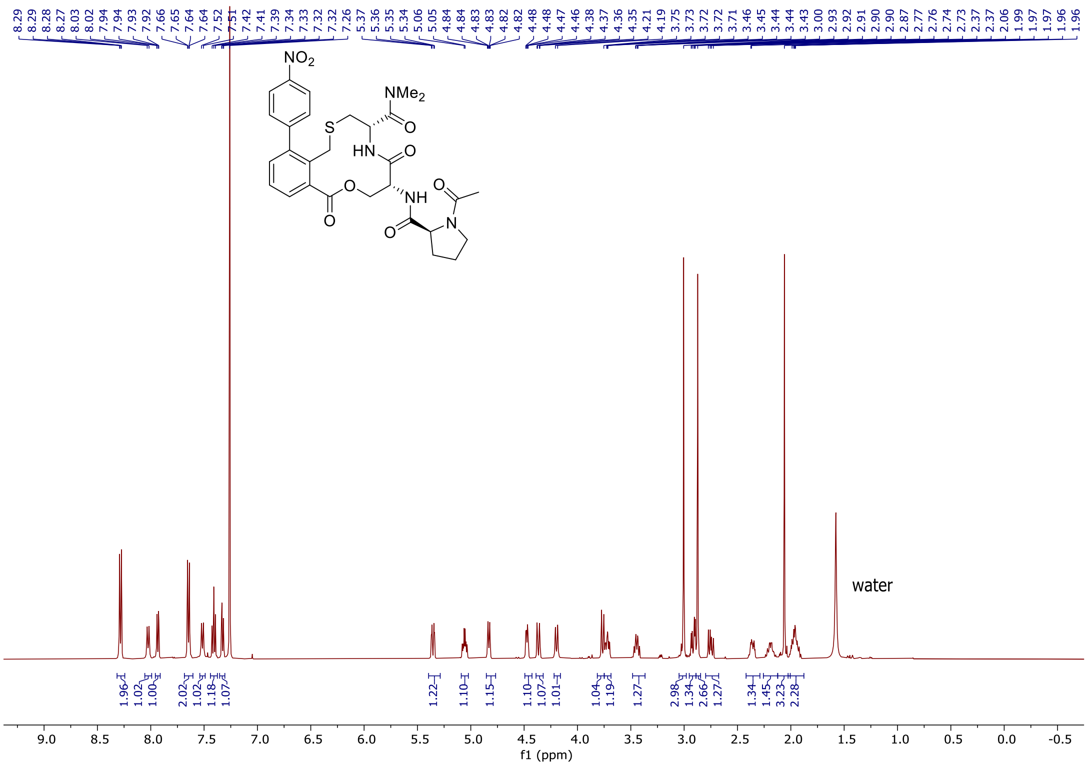

69 ^{13}C NMR (126 MHz, CDCl$_3$)

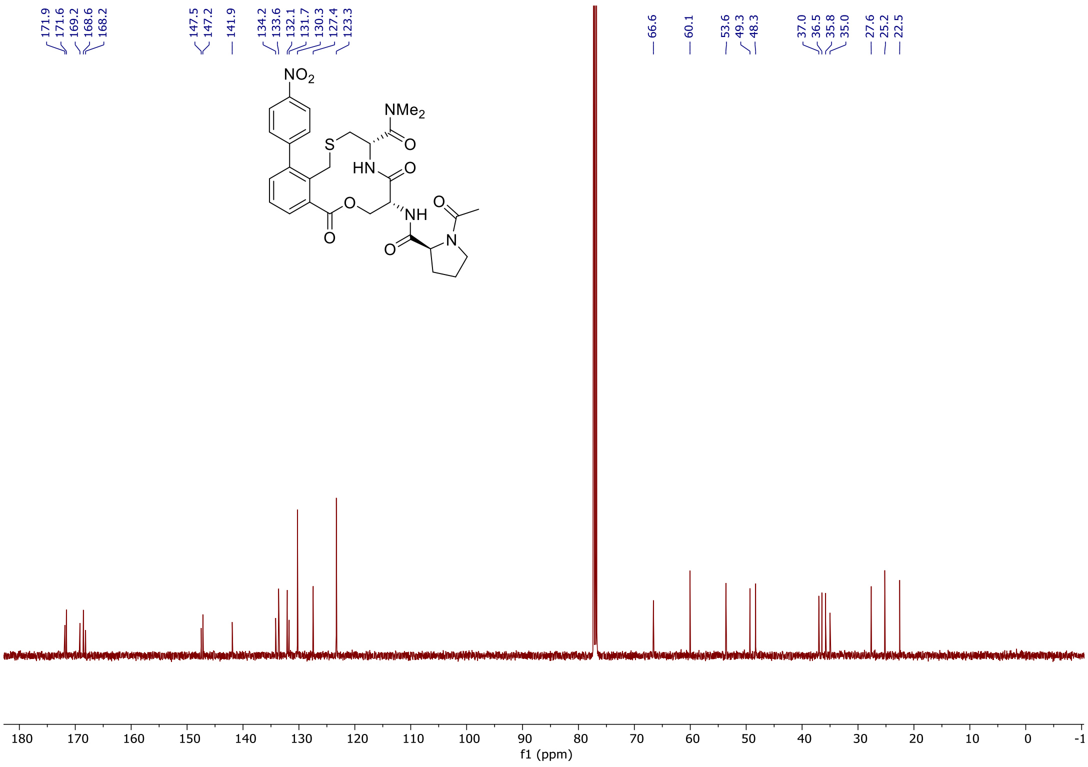

70 ^{1}H NMR (500 MHz, $CDCl_3$)

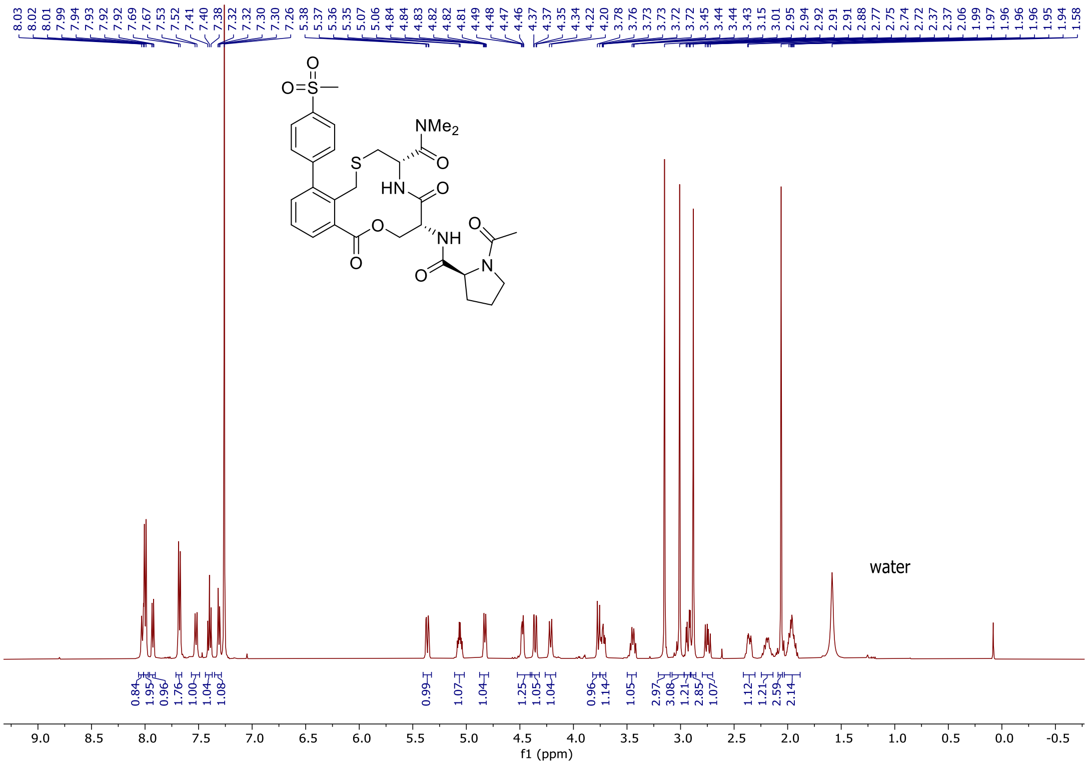

70 ^{13}C NMR (126 MHz, $CDCl_3$)

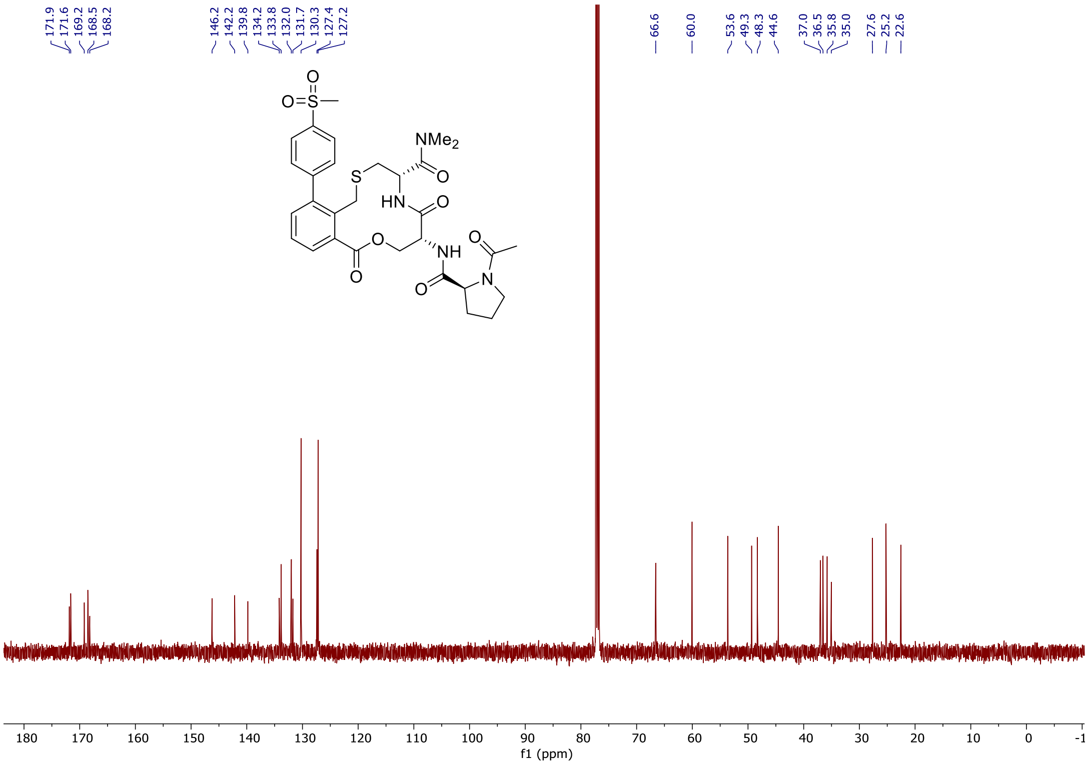

71 ^{1}H NMR (500 MHz, CDCl$_3$)

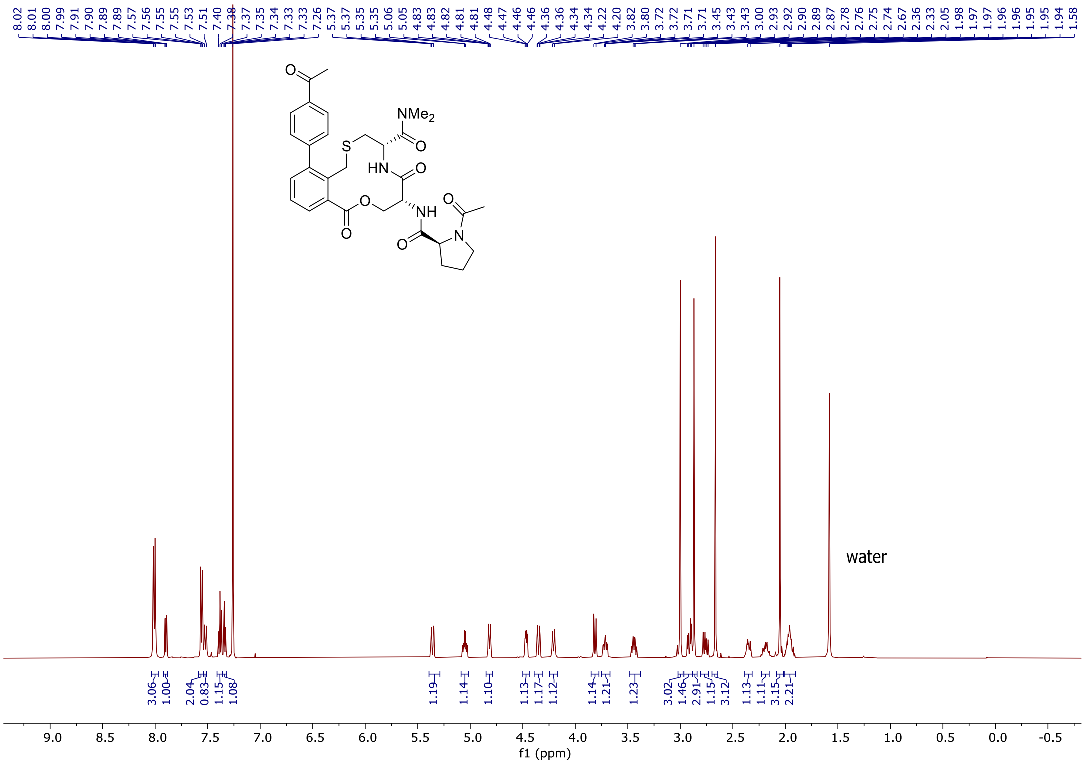

71 ^{13}C NMR (126 MHz, CDCl$_3$)

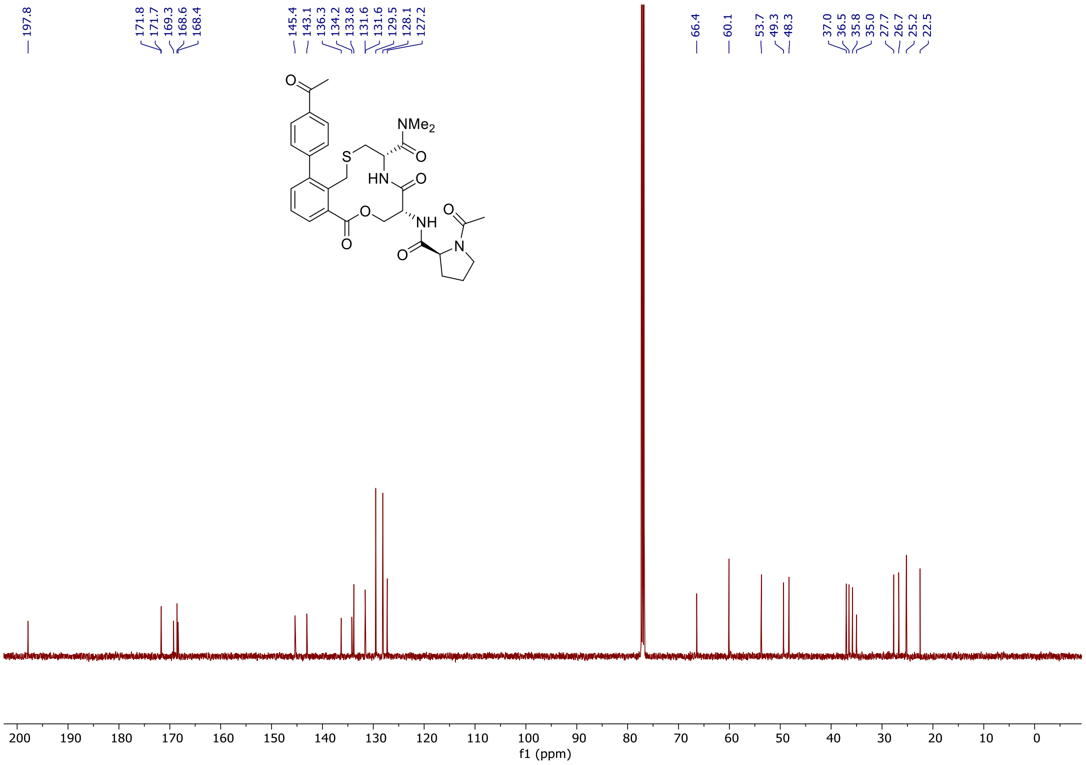

72 ^{1}H NMR (500 MHz, $CDCl_3$)

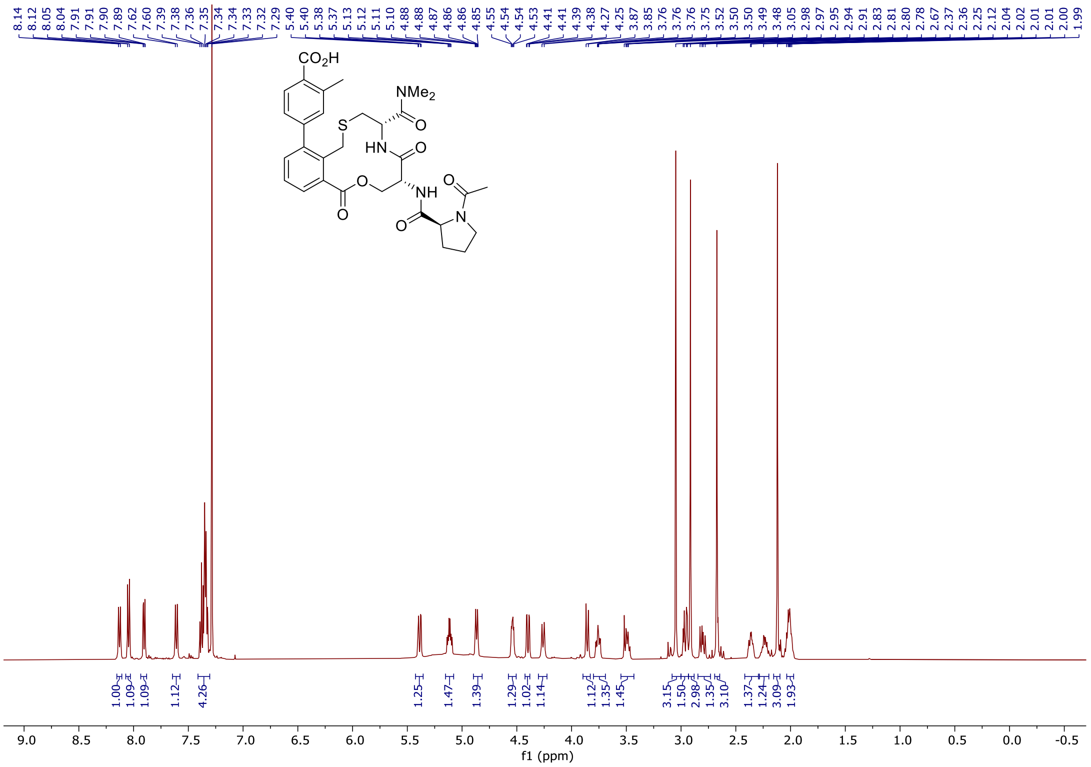

72 ^{13}C NMR (126 MHz, $CDCl_3$)

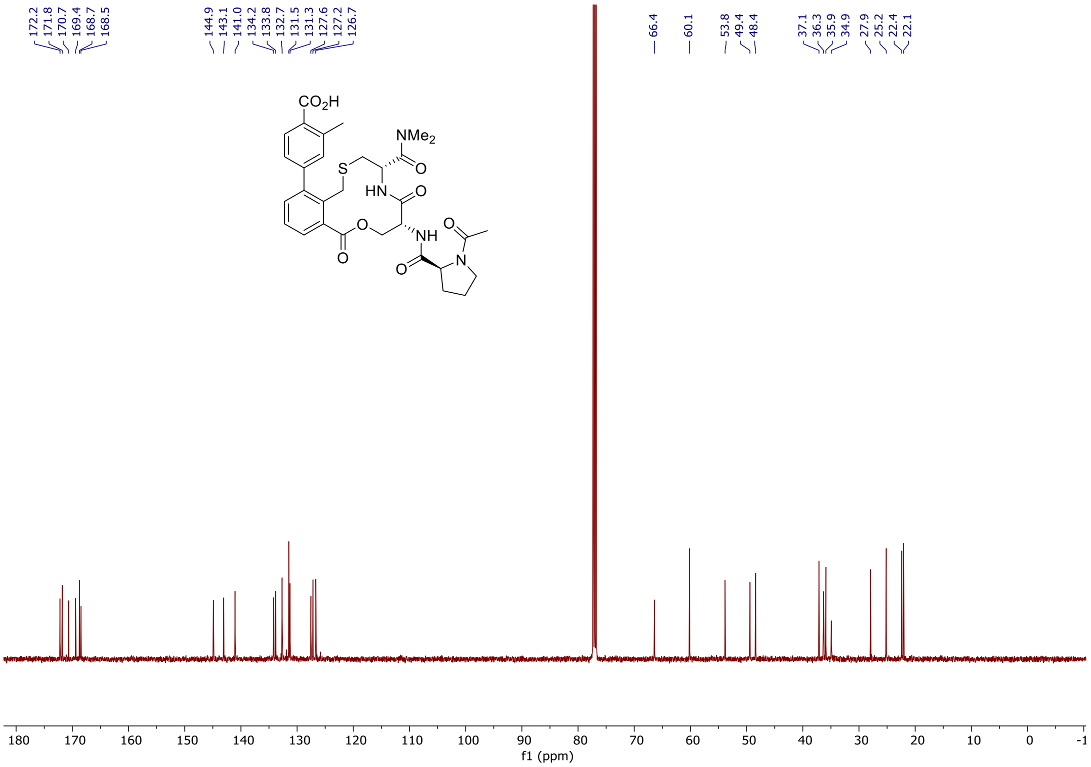

73 ^{1}H NMR (500 MHz, CDCl$_3$)

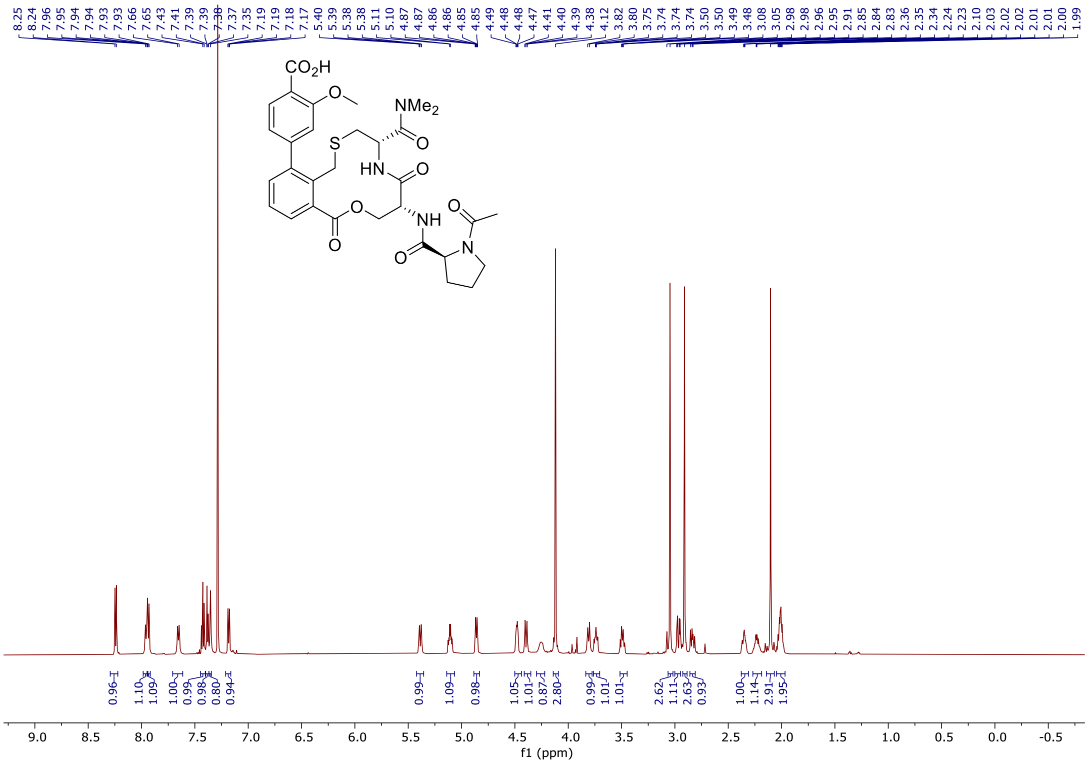

73 ^{1}H NMR (500 MHz, CDCl$_3$)

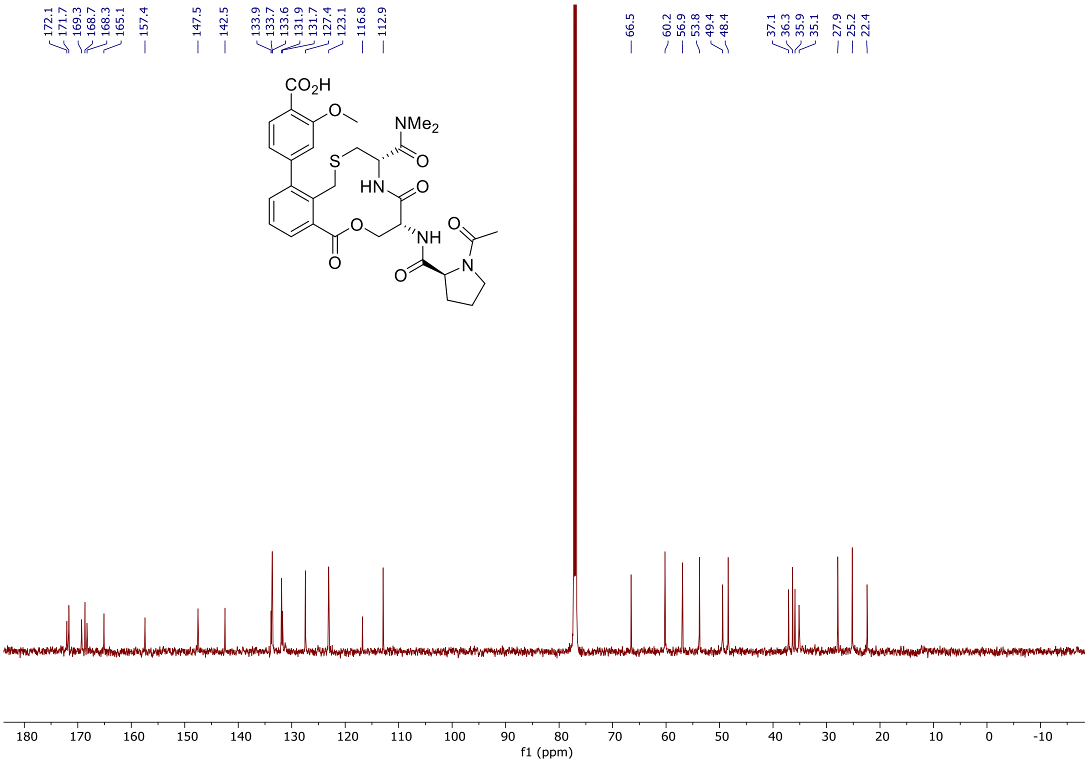

74 ^{13}C NMR (126 MHz, CDCl$_3$)

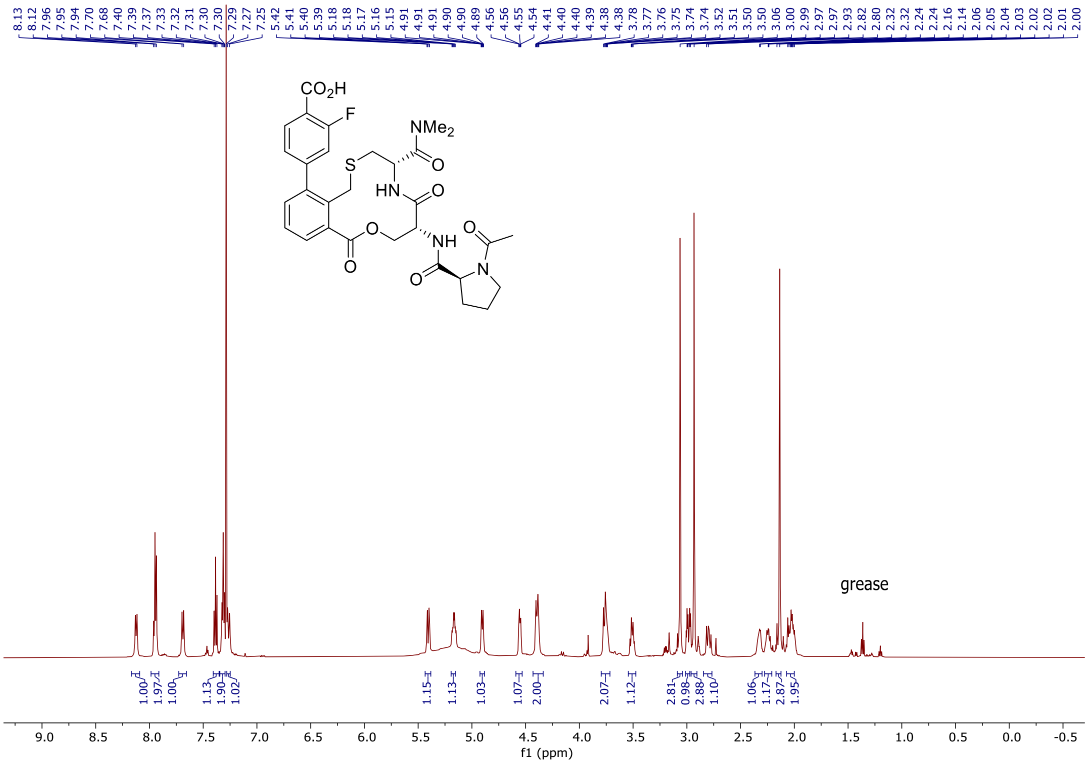

74 ^{1}H NMR (500 MHz, CDCl$_3$)

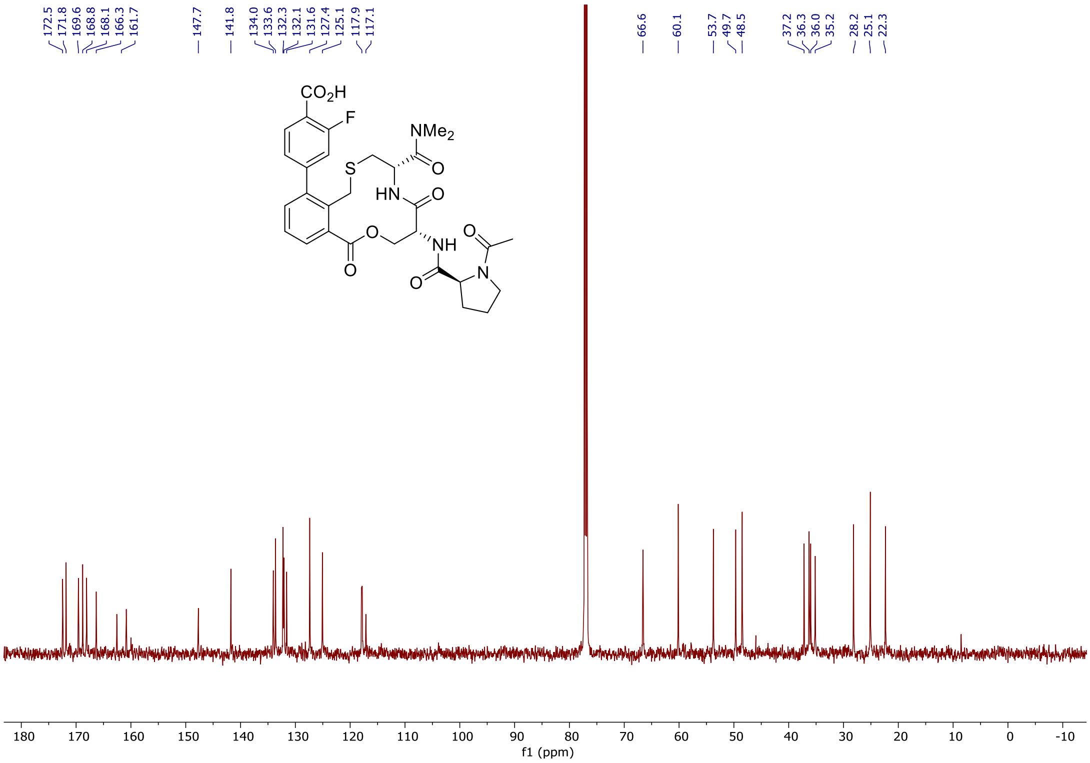

75 ^{13}C NMR (126 MHz, $CDCl_3$)

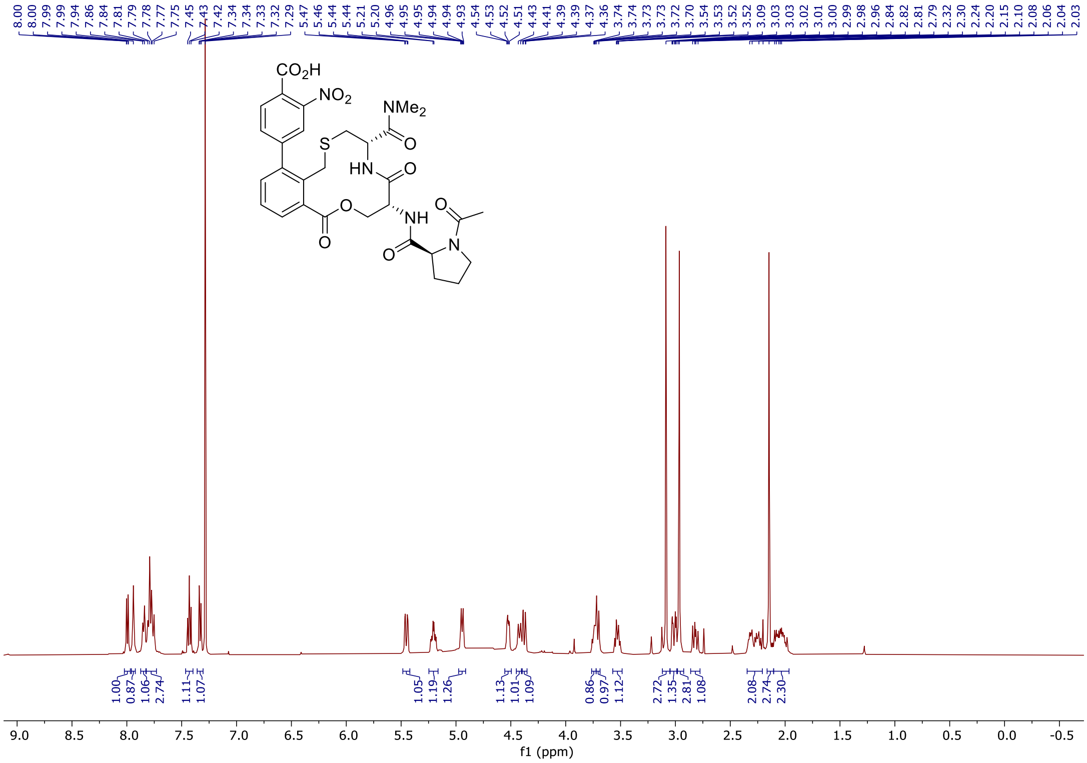

75 ^{1}H NMR (500 MHz, $CDCl_3$)

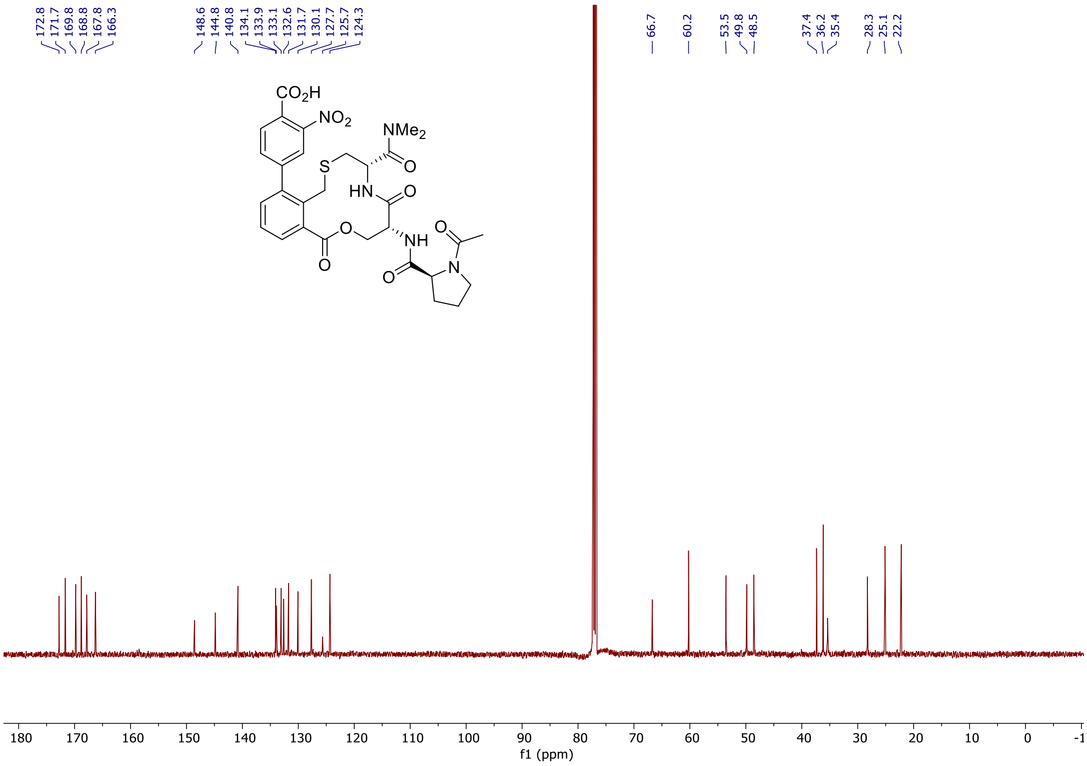

76 ^{1}H NMR (500 MHz, $CDCl_3$)

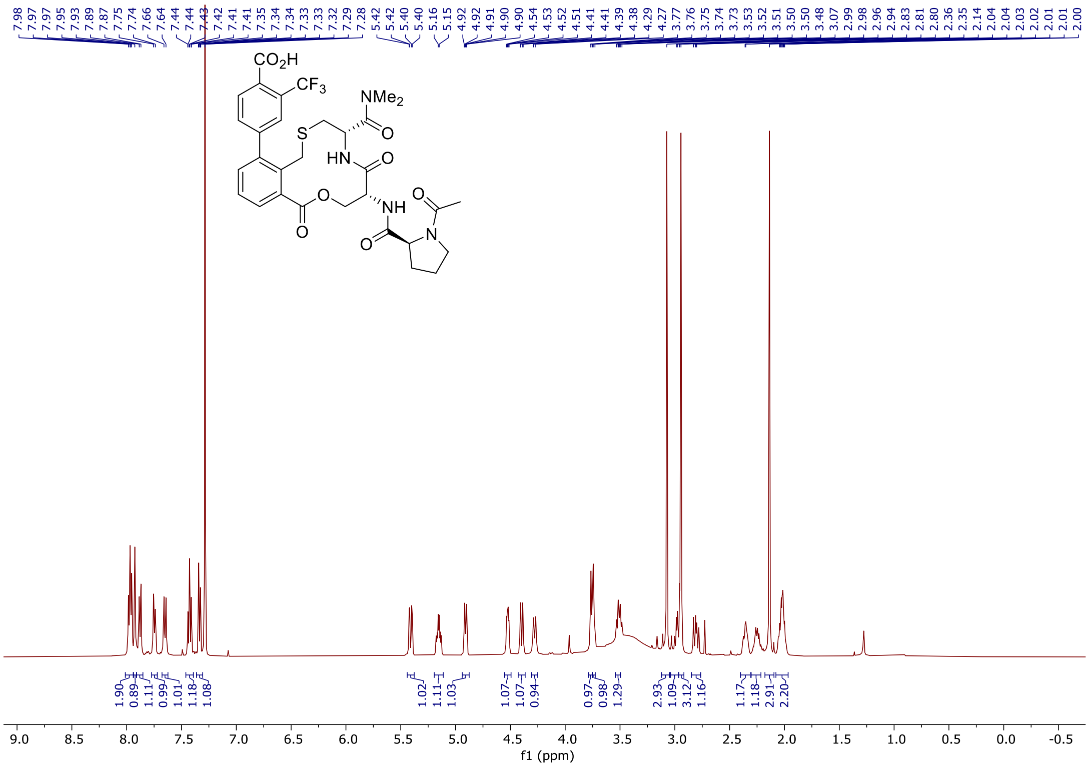

76 ^{13}C NMR (126 MHz, $CDCl_3$)

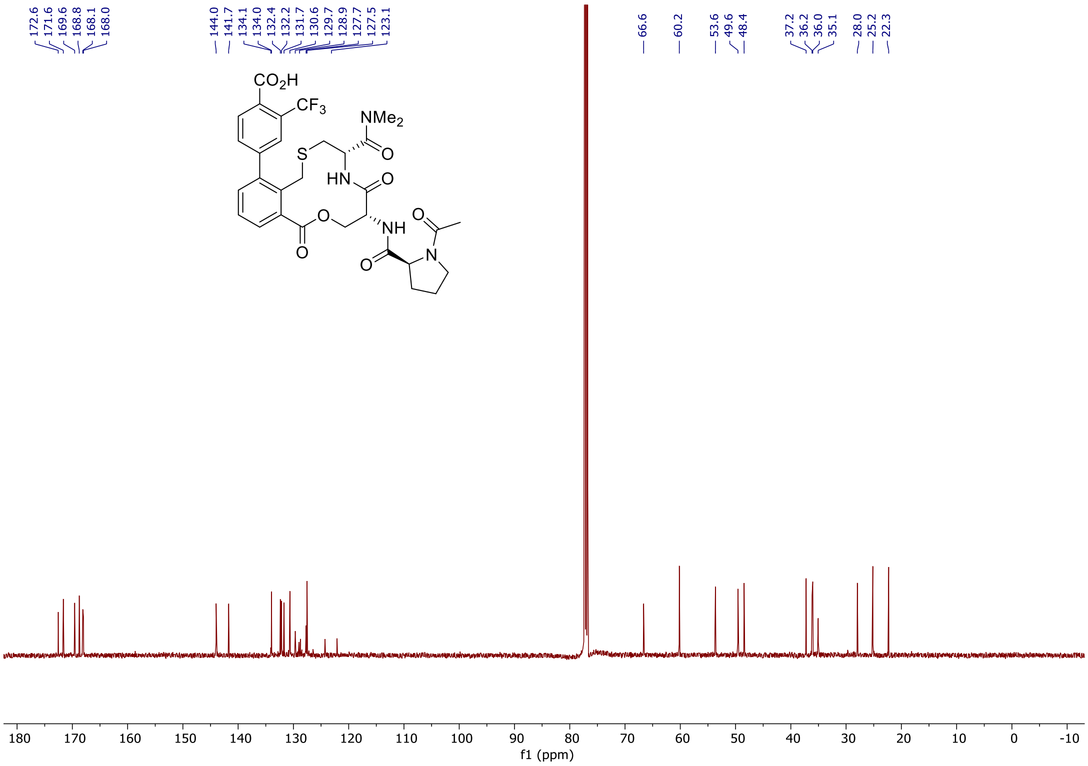

77 ^{1}H NMR (500 MHz, $CDCl_3$)

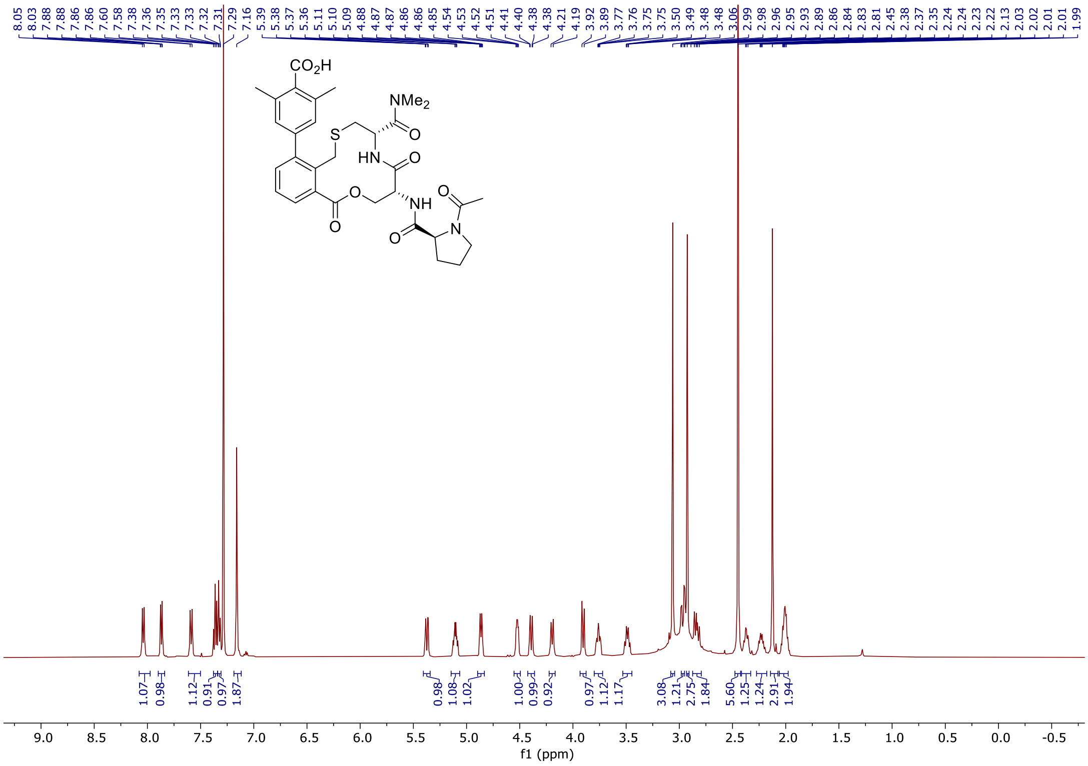

77 ^{13}C NMR (126 MHz, $CDCl_3$)

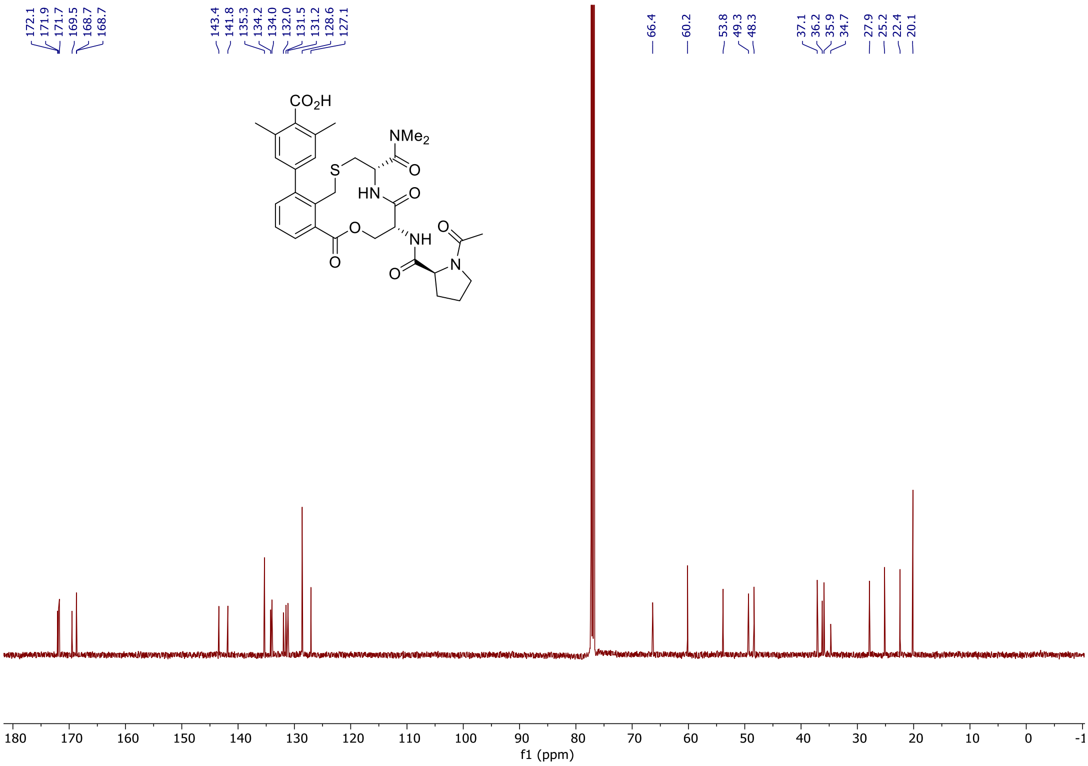

78 ^{1}H NMR (500 MHz, $CDCl_3$)

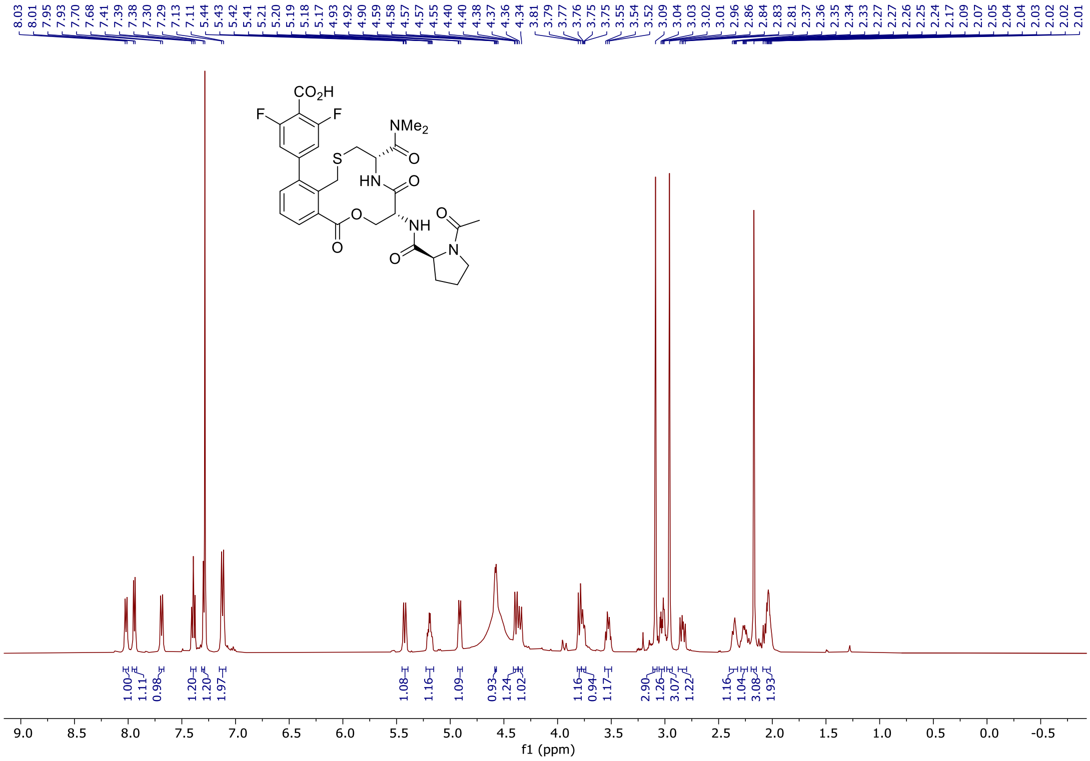

78 ^{13}C NMR (126 MHz, $CDCl_3$)

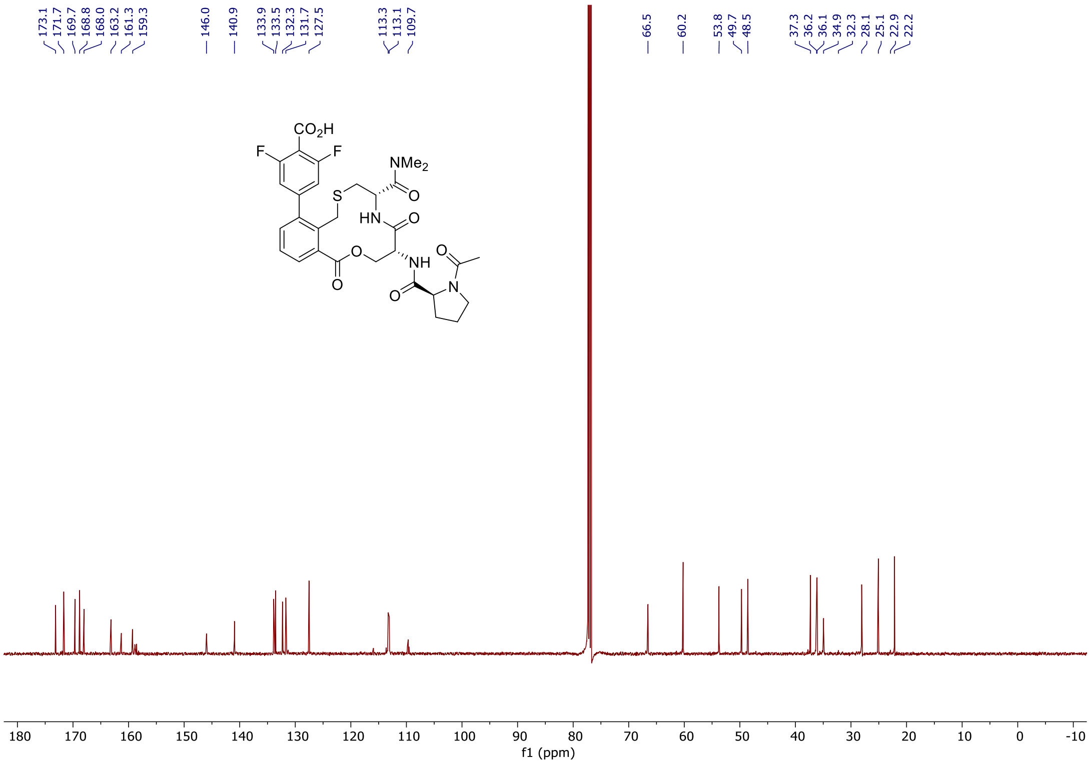

Purity reports for compounds 2–78

Compound **2**

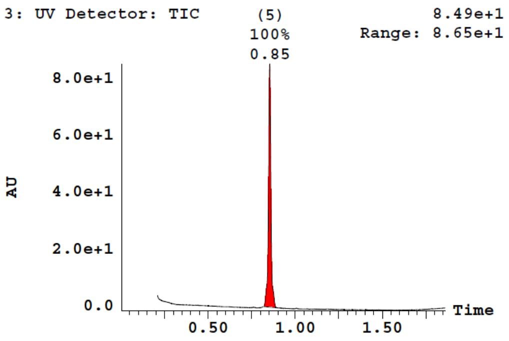

Peak ID	Time	Mass Found
5	0.83	

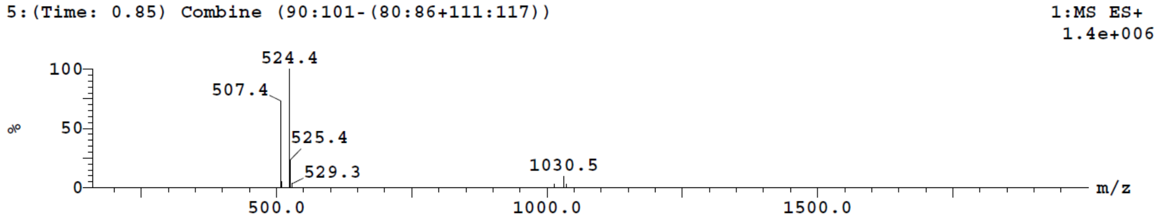

Compound **3**

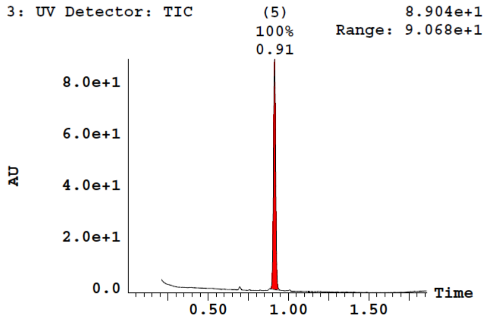

Peak ID	Time	Mass Found
5	0.90	

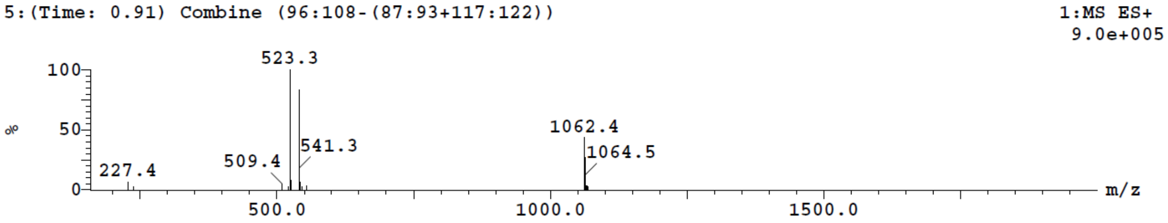

Compound **4**

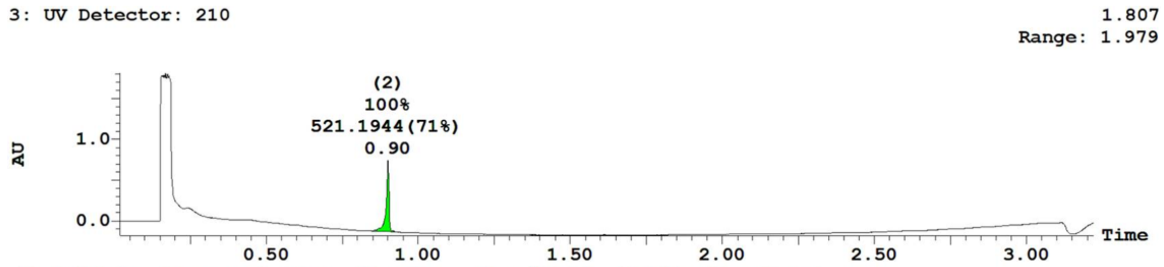

Peak Number	Compound Found	Time	AreaAbs	Area %Total	Height	Mass Found
2	Found	0.90	1e+004	99.52	9e+005	521.19
4		1.61	5e+001	0.48	3e+003	

Compound **5**

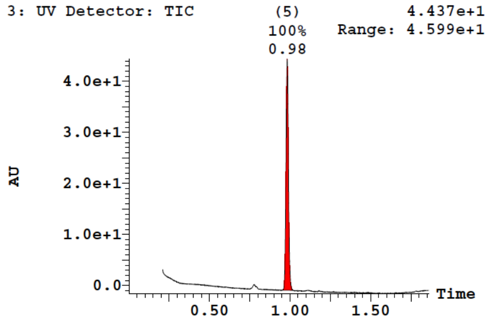

Peak ID	Time	Mass Found
5	0.99	

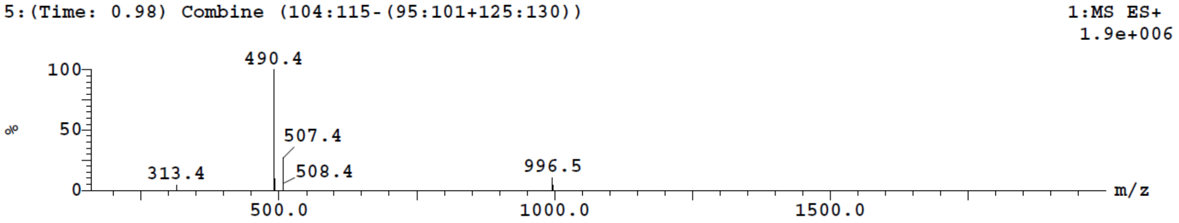

Compound **6**

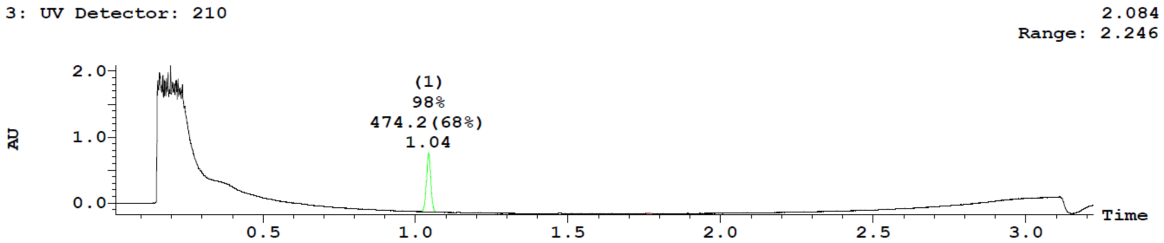

Peak Number	Compound Found	Time	AreaAbs	Area %Total	Width	Height	Mass Found
1	Found	1.04	1e+004	98.42	0	9e+005	474.21
4		1.76	2e+002	1.58	0	2e+004	

Compound **7**

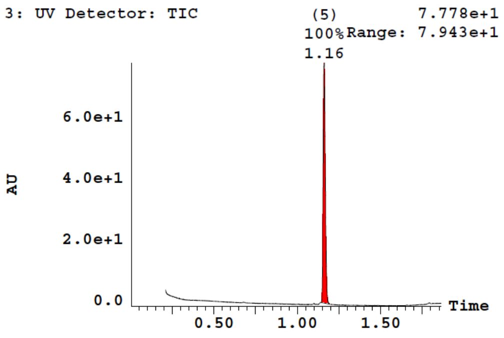

Peak ID	Time	Mass Found
5	1.16	

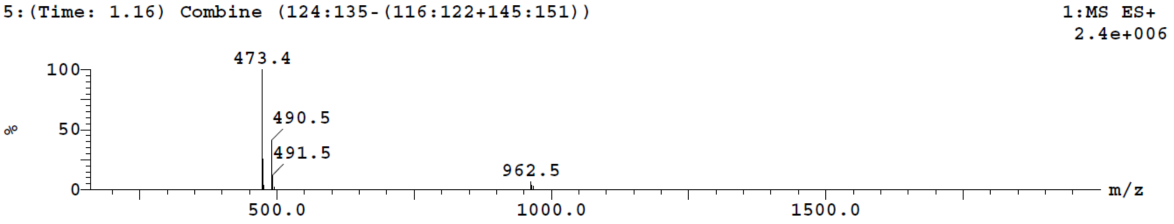

Compound **8**

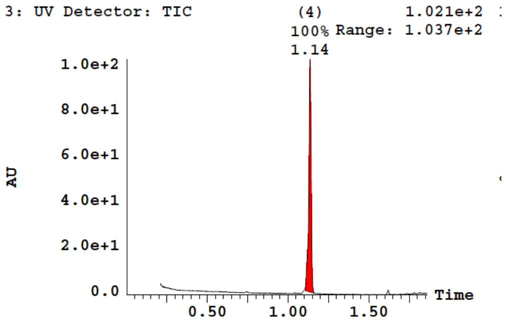

Peak ID	Time	Mass Found
4	1.14	

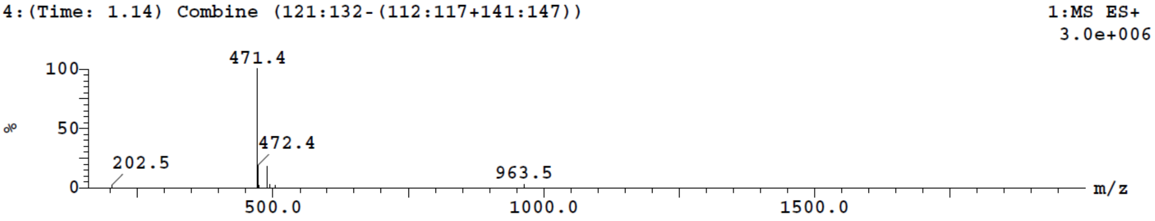

Compound **9**

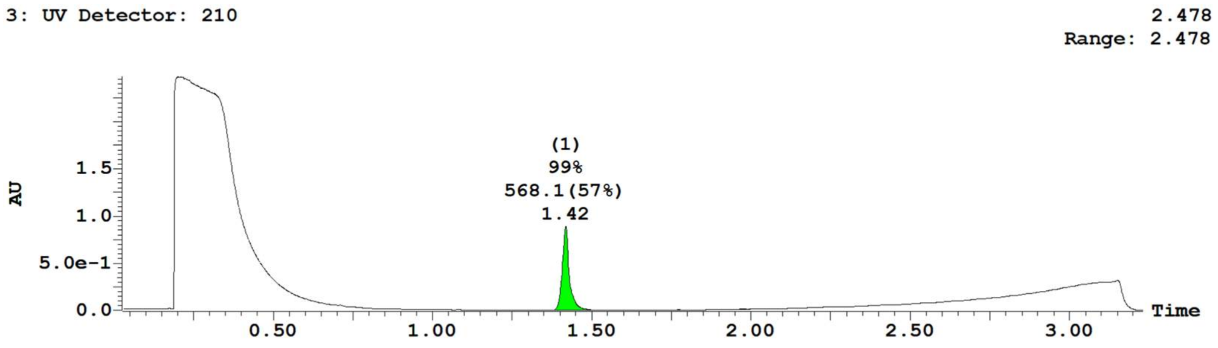

Compound **10**

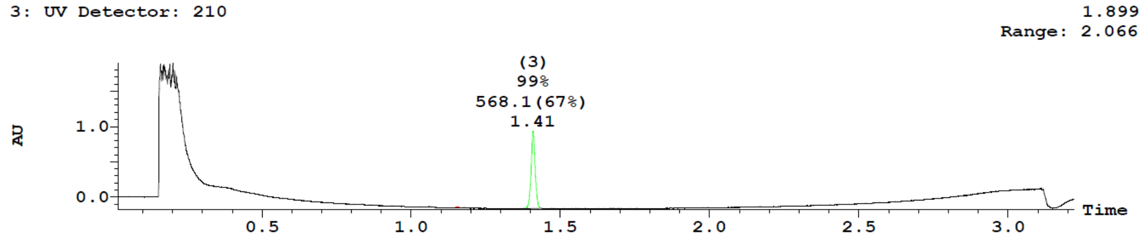

Peak Number	Compound	Time	AreaAbs	Area %Total	Width	Height	Mass Found
2		1.15	1e+002	0.77	0	2e+004	
3	Found	1.41	2e+004	99.14	0	1e+006	568.10
4		2.95	1e+001	0.08	0	7e+003	

Compound **11**

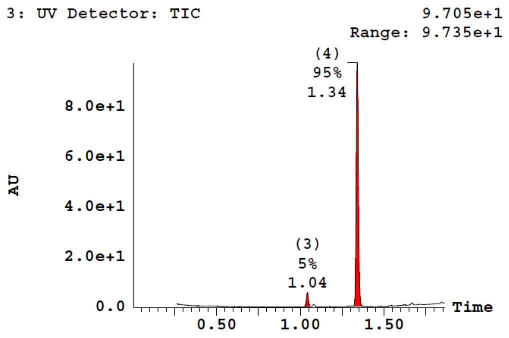

Peak ID	Time	Mass Found
4	1.35	

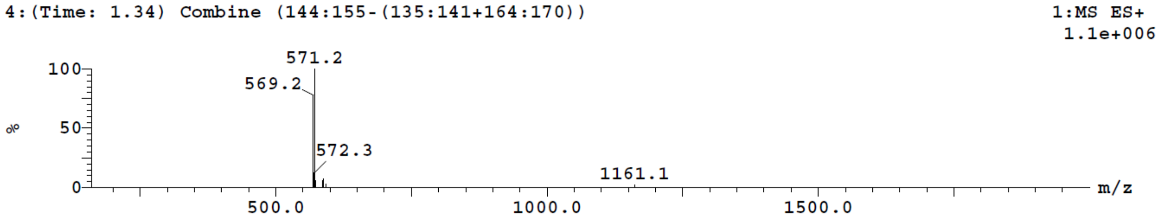

Compound **12**

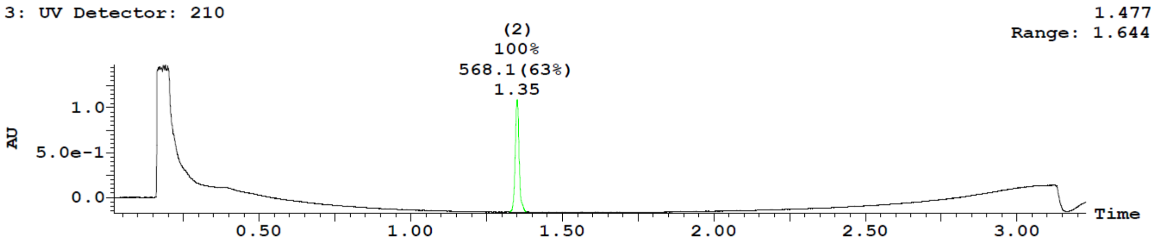

Peak Number	Compound	Time	AreaAbs	Area %Total	Height	Mass Found
2	Found	1.35	2e+004	100.00	1e+006	568.10

Compound **13**

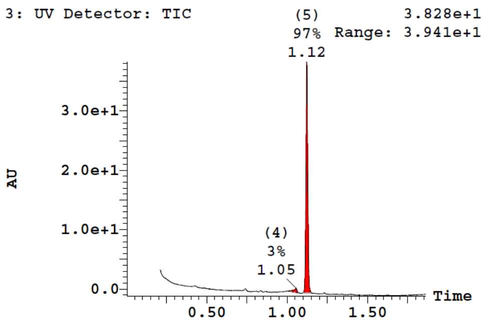

Peak ID	Time	Mass Found
5	1.12	

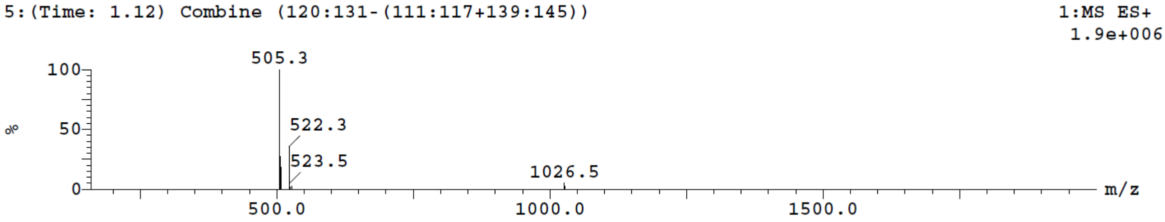

Compound **14**

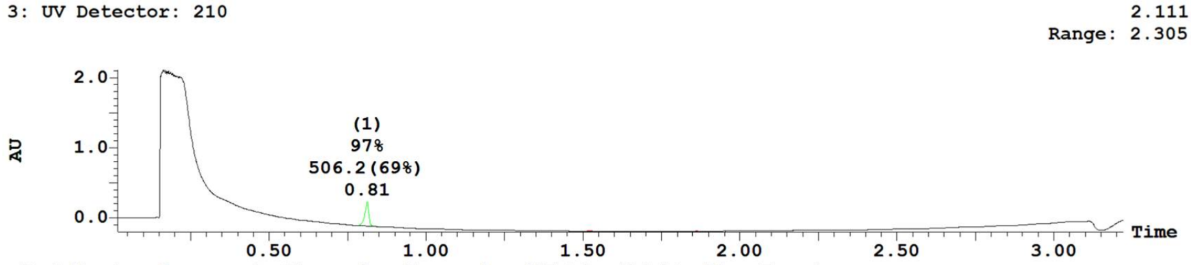

Peak Number	Compound	Time	AreaAbs	Area %Total	Height	Mass Found
1	Found	0.81	5e+003	97.22	3e+005	506.18
2		1.52	8e+001	1.59	7e+003	
3		1.62	3e+001	0.66	3e+003	
5		1.86	3e+001	0.52	2e+003	

Compound **15**

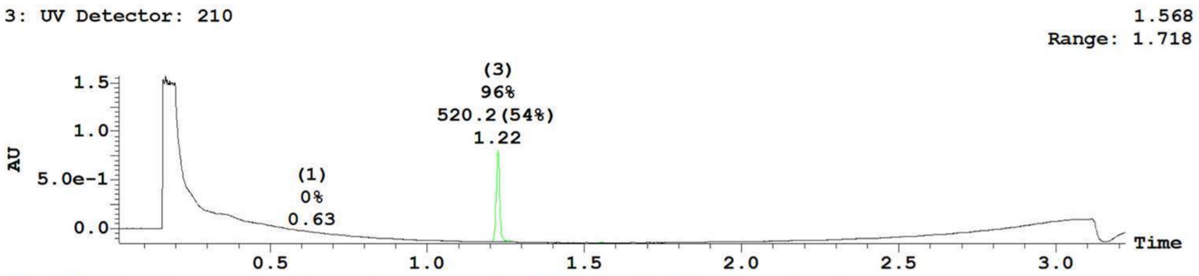

Peak Number	Compound	Time	AreaAbs	Area %Total	Height	Mass Found
1		0.63	2e+001	0.16	3e+003	
3	Found	1.22	1e+004	96.24	9e+005	520.20
4	Found	1.26	4e+002	2.86	2e+004	520.20
5	Found	1.55	7e+001	0.60	7e+003	520.20
7		2.71	8e+000	0.06	2e+003	
8		2.86	9e+000	0.07	2e+003	

Compound **16**

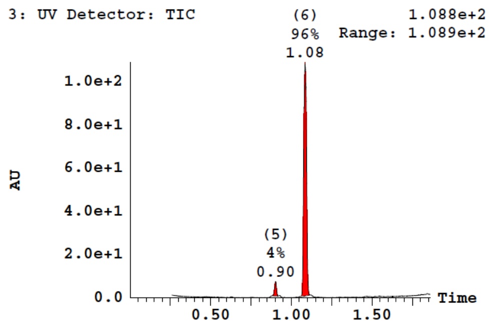

Peak ID	Time	Mass Found
6	1.08	

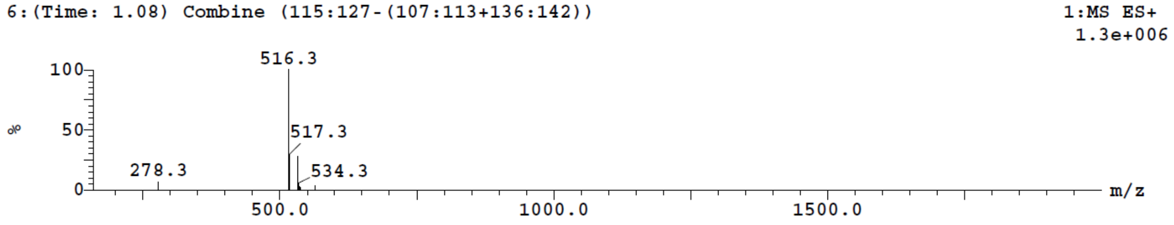

Compound **17**

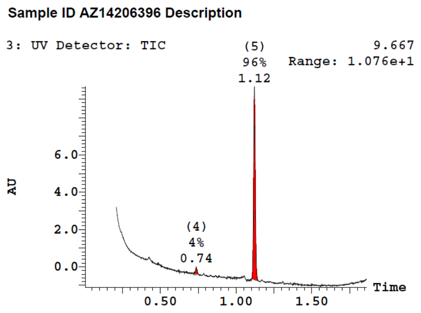

Peak ID	Time	Mass Found
5	1.12	

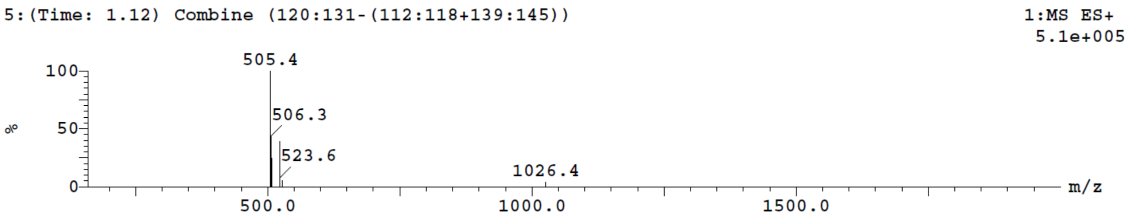

Compound **18**

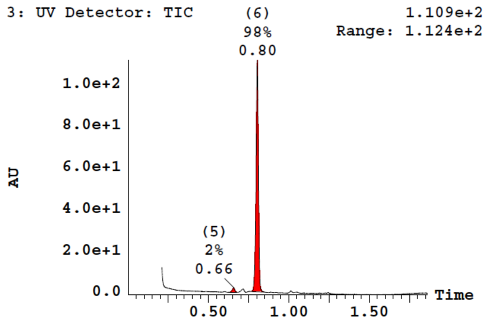

Peak ID	Time	Mass Found
6	0.81	

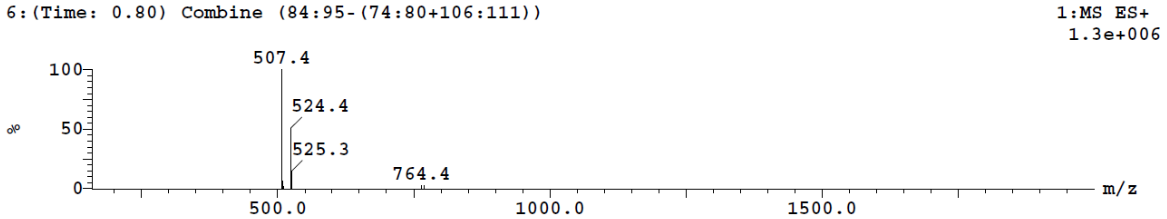

Compound **19**

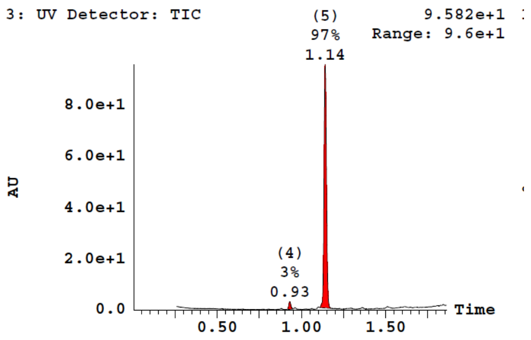

Peak ID	Time	Mass Found
5	1.14	

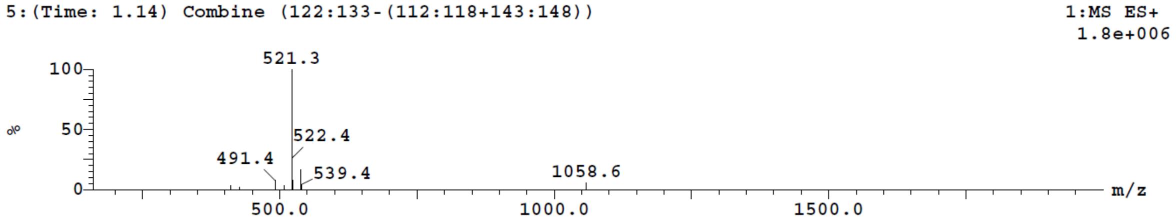

Compound **20**

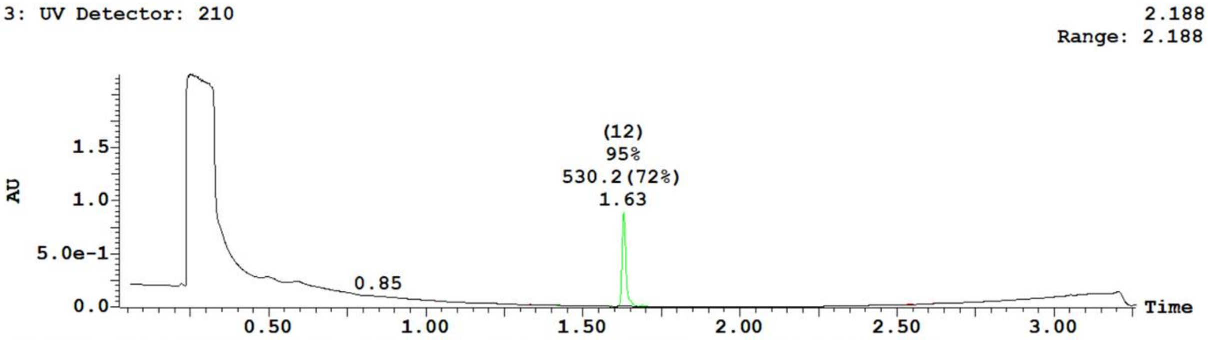

Peak Number	Compound	Time	AreaAbs	Area %Total	Width	Height	Mass Found
1		0.85	3e+001	0.19	0		
2		0.99	1e+001	0.09	0		
3	Found	1.10	1e+001	0.08	0		530.22
4		1.33	6e+001	0.42	0	4e+003	
5		1.37	2e+001	0.17	0	2e+003	
6		1.39	2e+001	0.18	0	1e+003	
7	Tentative	1.41	2e+001	0.13	0	1e+003	530.22
8	Tentative	1.49	2e+001	0.13	0	1e+003	530.22
9	Tentative	1.52	2e+001	0.13	0	1e+003	530.22
10		1.55	1e+001	0.08	0	1e+003	
11	Found	1.57	3e+001	0.25	0	2e+003	530.22
12	Found	1.63	1e+004	94.85	0	9e+005	530.22
13	Found	1.69	2e+002	1.77	0	1e+004	530.22
14	Found	1.79	7e+001	0.51	0	5e+003	530.22
15	Found	1.85	2e+001	0.15	0	2e+003	530.22
16		2.02	4e+001	0.30	0	3e+003	
17		2.54	3e+001	0.21	0	2e+003	
18		2.62	1e+001	0.09	0	2e+002	
21		2.80	9e+000	0.06	0	1e+003	
22		2.82	3e+001	0.21	0	2e+003	

Compound **21**

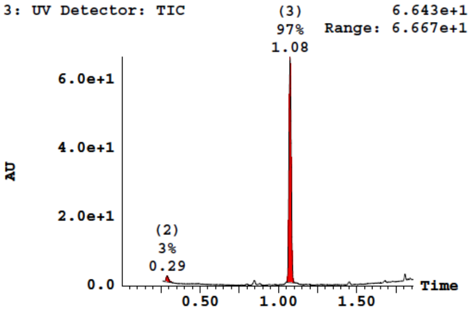

Peak ID	Time	Mass Found
3	1.07	

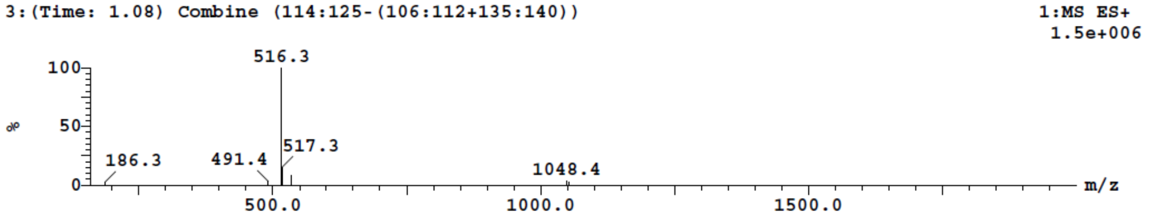

Compound **22**

Sample ID PLATE 5 F2 Description

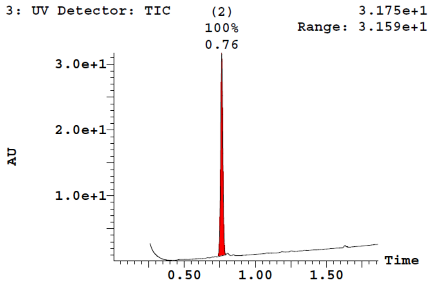

Peak ID	Time	Mass Found
2	0.77	

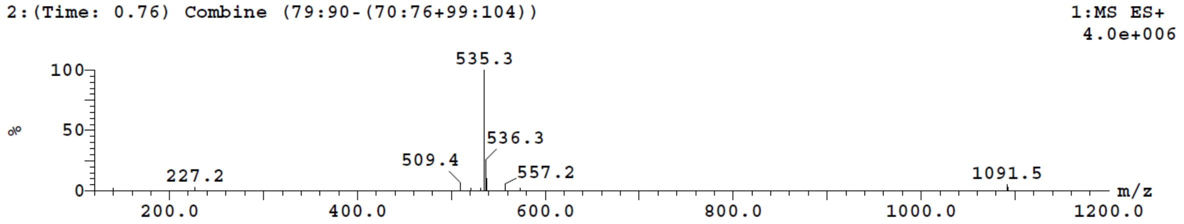

Compound **23**

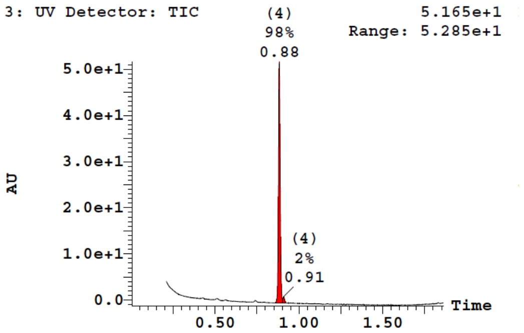

Peak ID	Time	Mass Found
4	0.89	

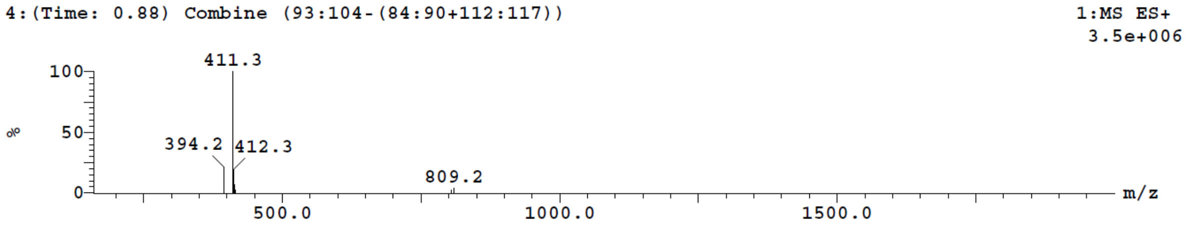

Compound **24**

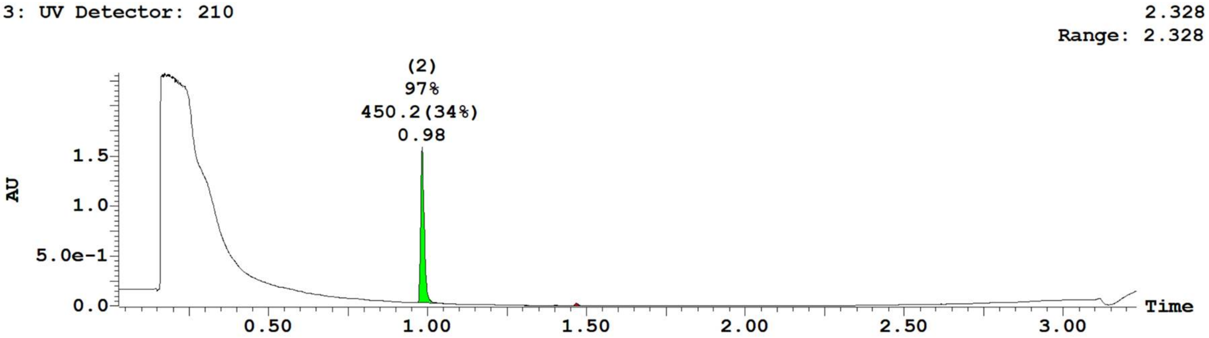

Compound **25**

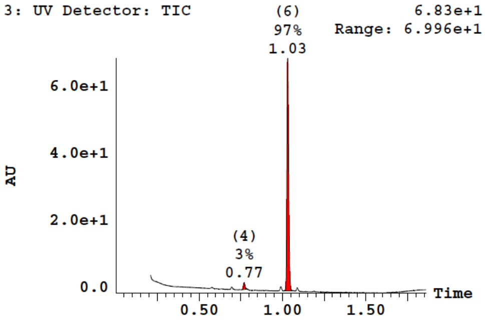

Peak ID	Time	Mass Found
6	1.03	

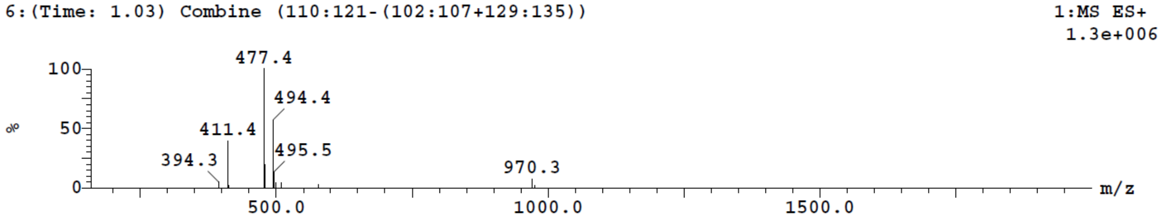

Compound **26**

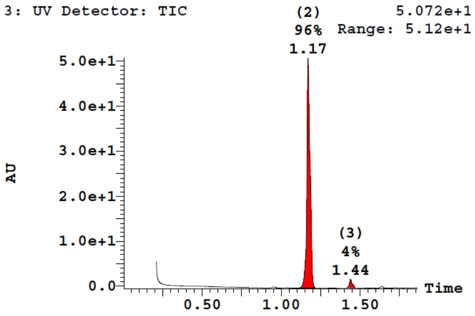

Peak ID	Time	Mass Found
2	1.18	

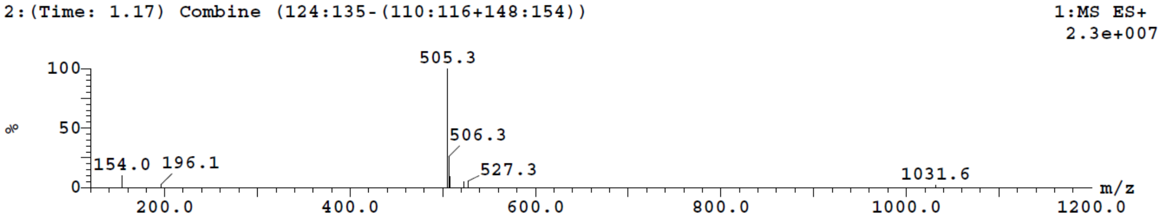

Compound **27**

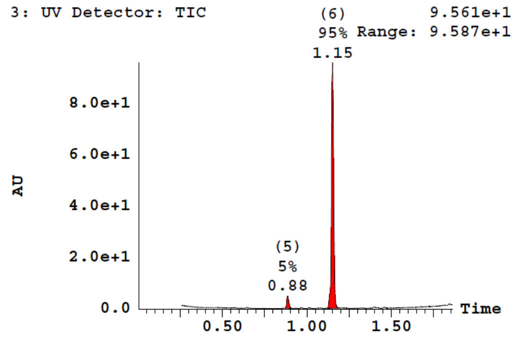

Peak ID	Time	Mass Found
6	1.15	

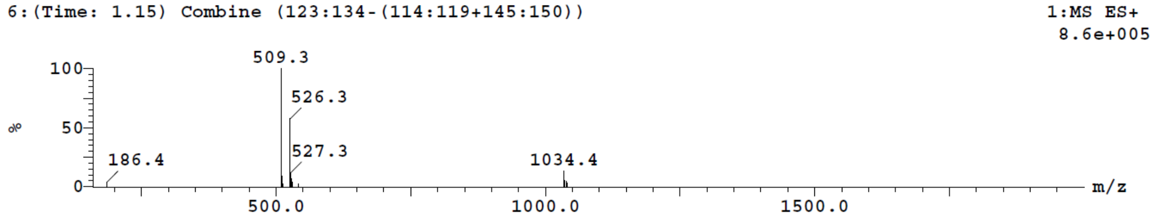

Compound **28**

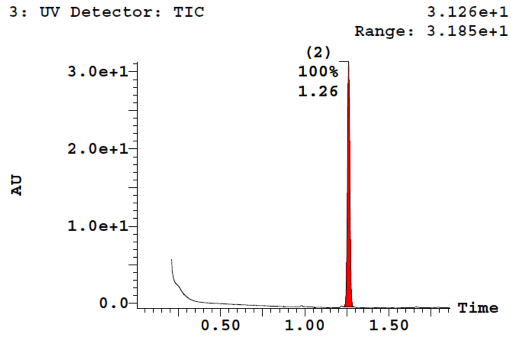

Peak ID	Time	Mass Found
2	1.27	

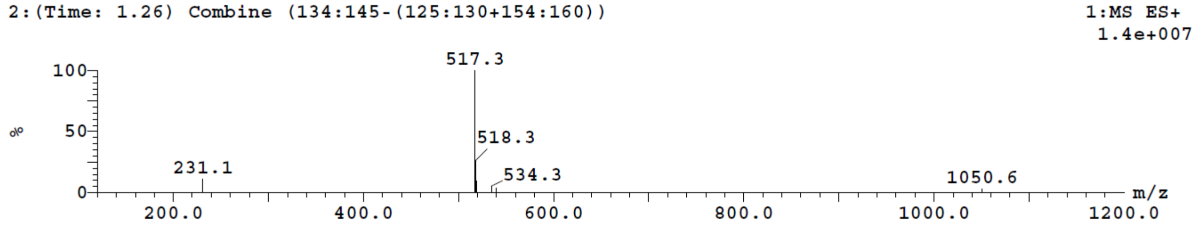

Compound **29**

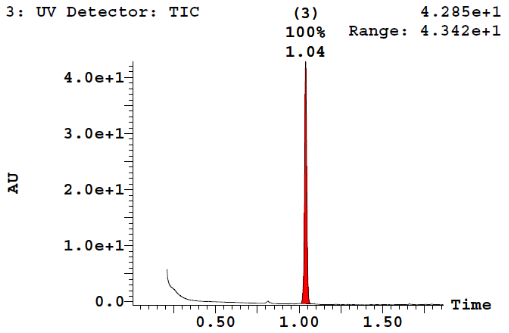

Peak ID	Time	Mass Found
3	1.04	

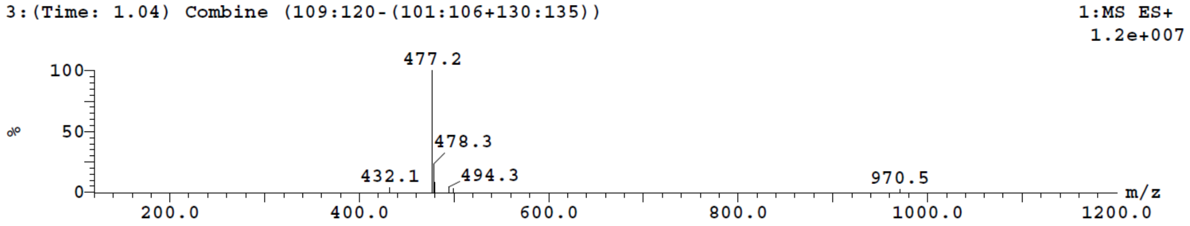

Compound **30**

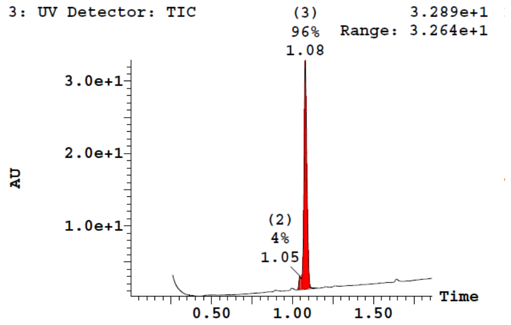

Peak ID	Time	Mass Found
3	1.08	

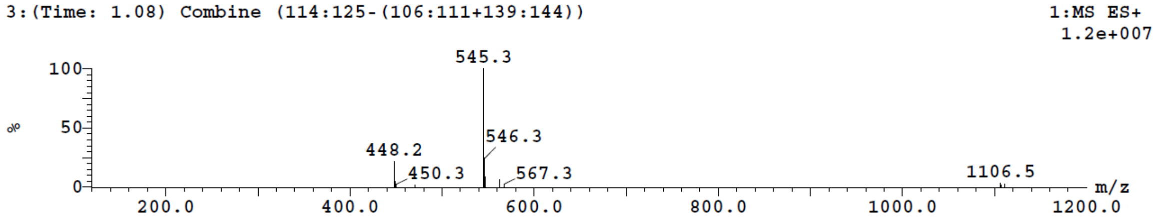

Compound **31**

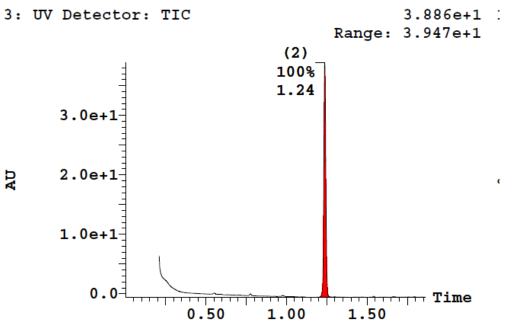

Peak ID	Time	Mass Found
2	1.24	

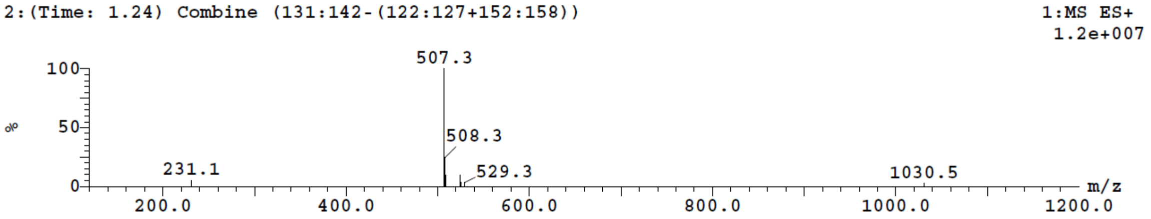

Compound **32**

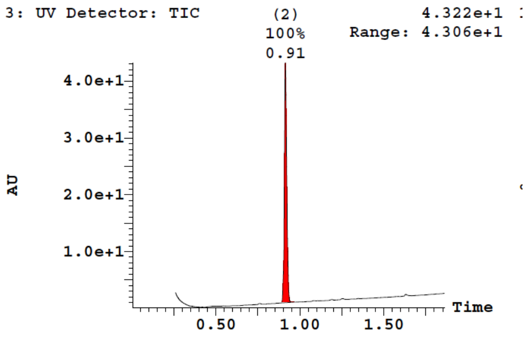

Peak ID	Time	Mass Found
2	0.92	

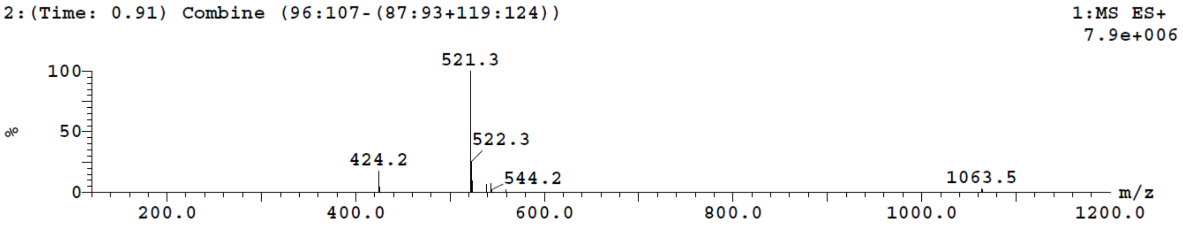

Compound **33**

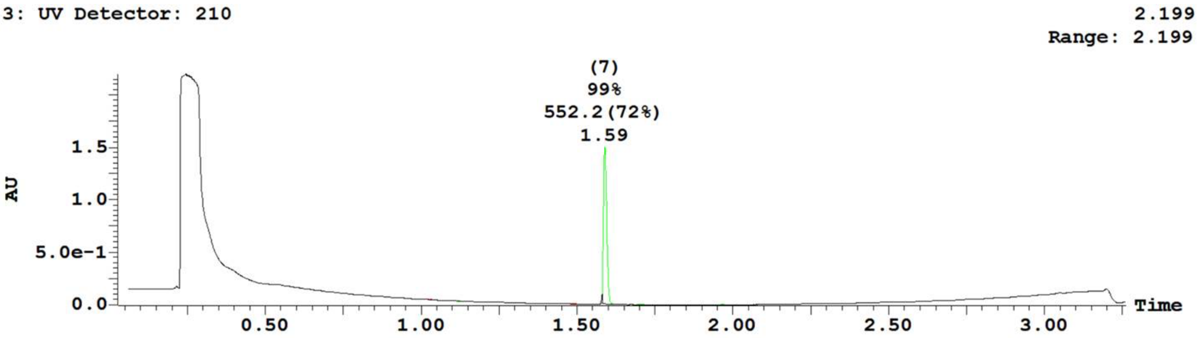

Peak Number	Compound	Time	AreaAbs	Area %Total	Width	Height	Mass Found
1		1.03	2e+001	0.13	0	2e+003	
2	Found	1.11	1e+001	0.05	0		552.20
3		1.21	3e+001	0.17	0	2e+003	
4		1.33	1e+001	0.06	0	9e+002	
5		1.40	1e+001	0.05	0	9e+002	
6		1.48	1e+001	0.07	0	9e+002	
7	Found	1.59	2e+004	98.68	0	1e+006	552.20
7	Found	1.62	2e+001	0.08	0	2e+003	552.20
8	Found	1.70	7e+001	0.40	0	5e+003	552.20
9	Found	1.77	2e+001	0.12	0	2e+003	552.20
10	Found	1.97	3e+001	0.18	0	3e+003	552.20

Compound **34**

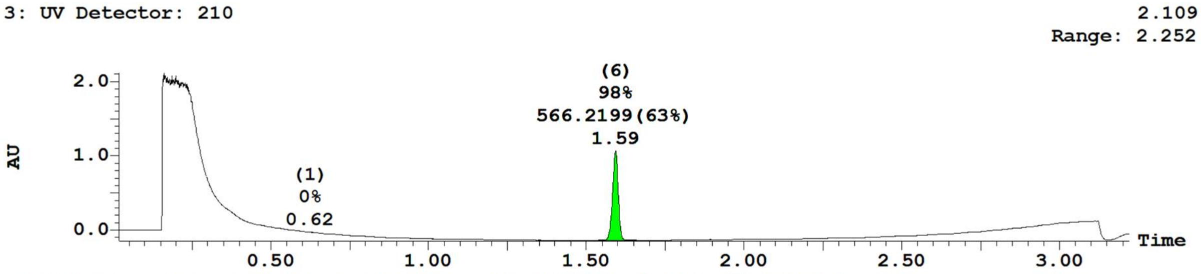

Peak Number	Compound	Time	AreaAbs	Area %Total	Height	Mass Found
1		0.62	4e+001	0.15	4e+003	
2		1.31	9e+001	0.36	6e+003	
3		1.35	4e+001	0.15	3e+003	
4	Found	1.51	2e+001	0.06	2e+003	566.22
5	Found	1.54	1e+002	0.38	8e+003	566.22
6	Found	1.59	3e+004	98.07	1e+006	566.22
7	Found	1.64	1e+002	0.53	8e+003	566.22
8		1.89	5e+001	0.20	4e+003	
9		2.32	3e+001	0.10	2e+003	

Compound **35**

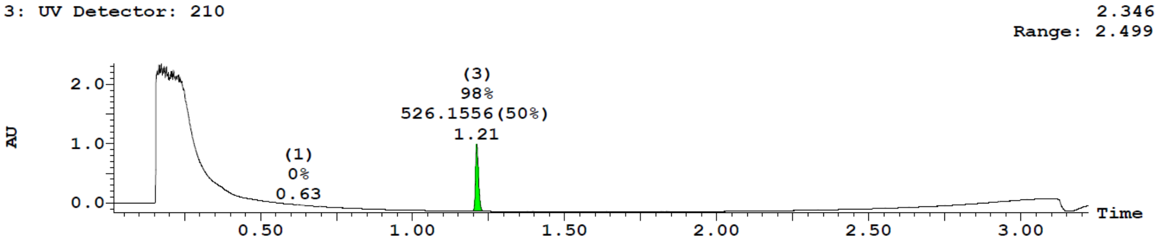

Peak Number	Compound	Time	AreaAbs	Area %Total	Height	Mass Found
1		0.63	5e+001	0.39	5e+003	
2		0.70	1e+001	0.08	2e+003	
3	Found	1.21	1e+004	97.77	1e+006	526.16
4		1.54	1e+002	0.95	9e+003	
5		1.64	1e+002	0.77	7e+003	
7		2.93	6e+000	0.05	2e+003	

Compound **36**

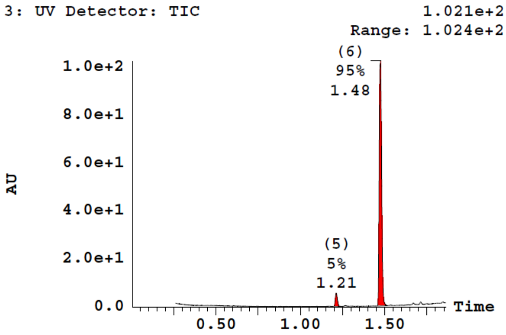

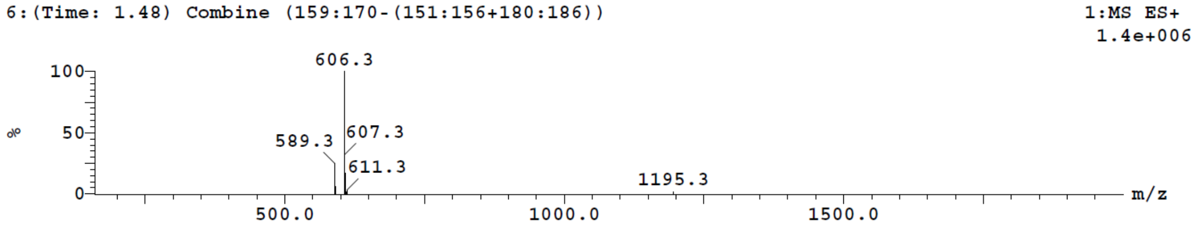

Compound **37**

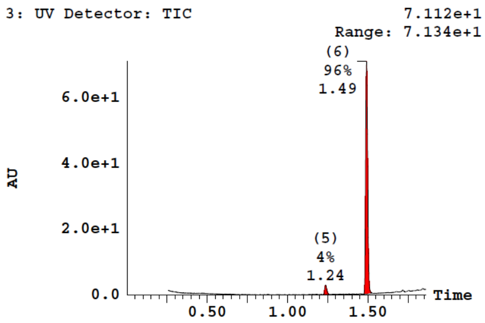

Peak ID	Time	Mass Found
6	1.50	

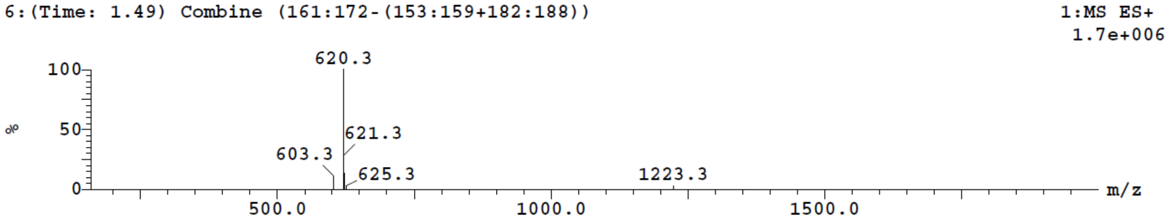

Compound **38**

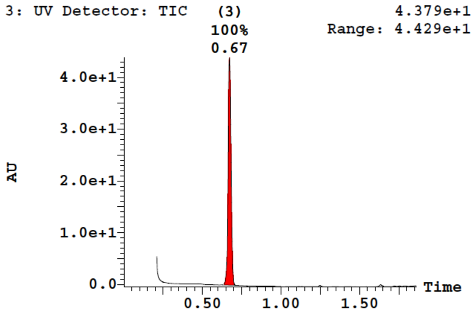

Peak ID	Time	Mass Found
3	0.67	

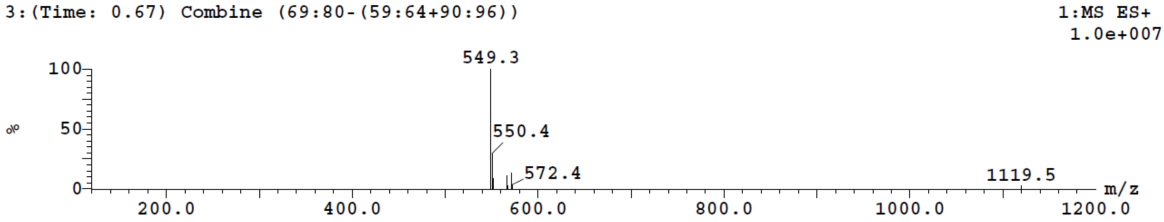

Compound **39**

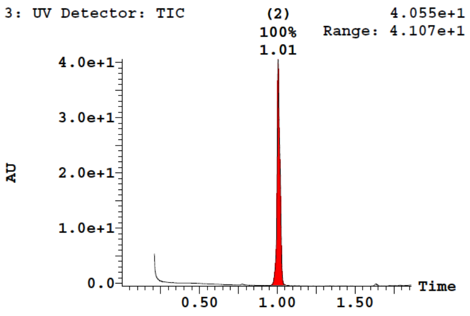

Sample Report (continued):

Peak ID	Time	Mass Found
2	1.00	

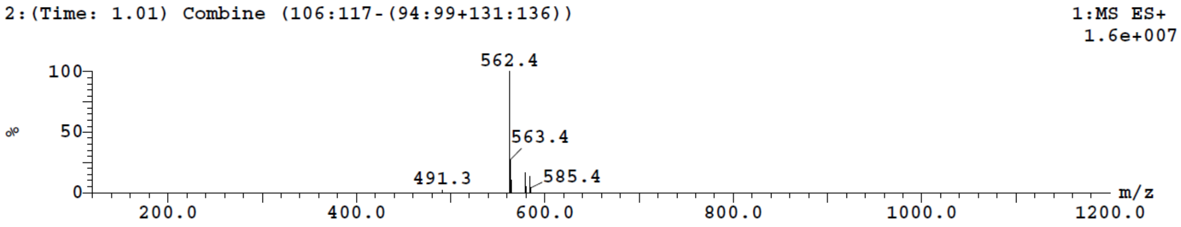

Compound **40**

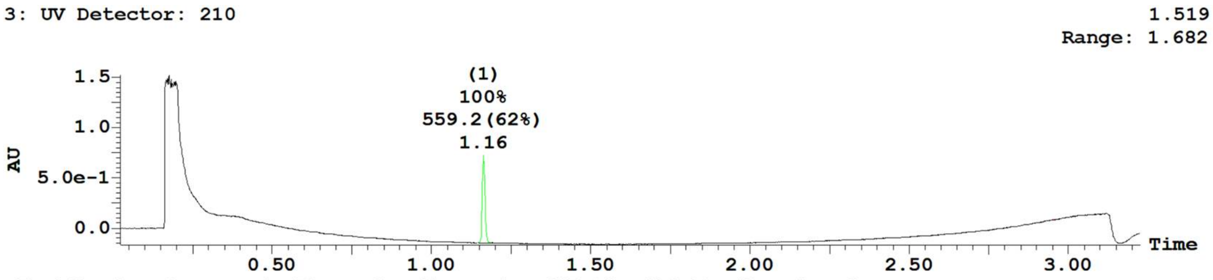

Peak Number	Compound	Time	AreaAbs	Area %Total	Height	Mass Found
1	Found	1.16	9e+003	99.64	9e+005	559.21
6		2.97	3e+001	0.36	8e+002	

Compound **41**

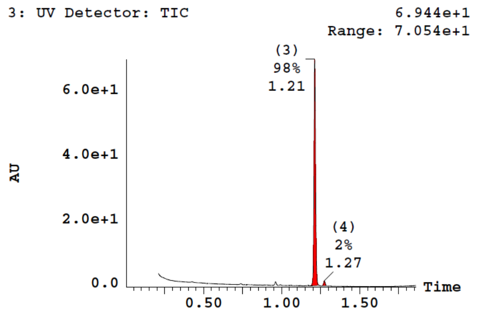

Peak ID	Time	Mass Found
3	1.21	

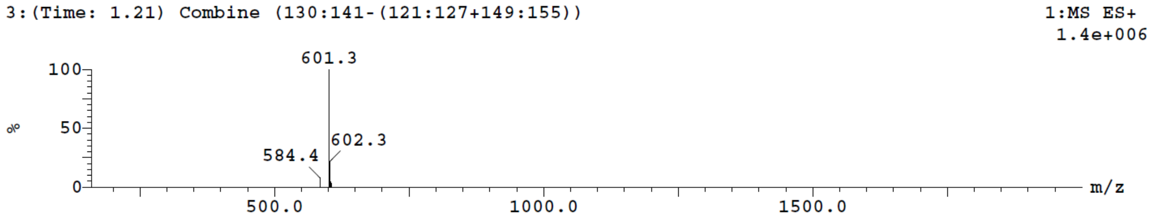

Compound **42**

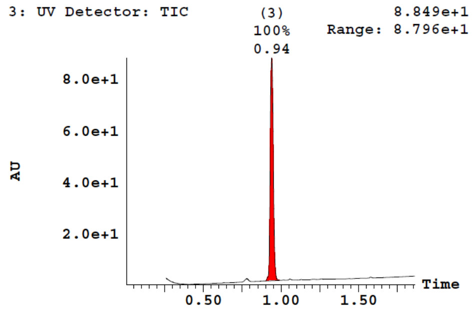

Peak ID	Time	Mass Found
3	0.94	

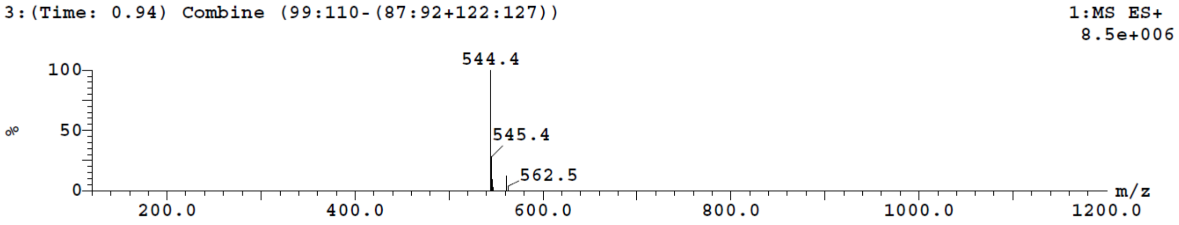

Compound **43**

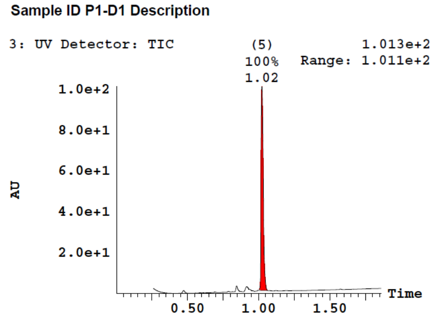

Peak ID	Time	Mass Found
5	1.03	

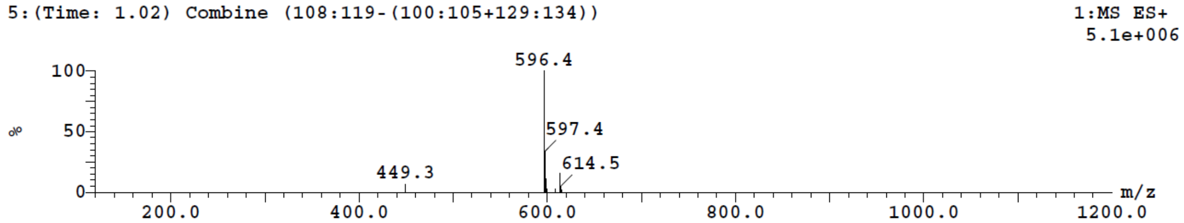

Compound **44**

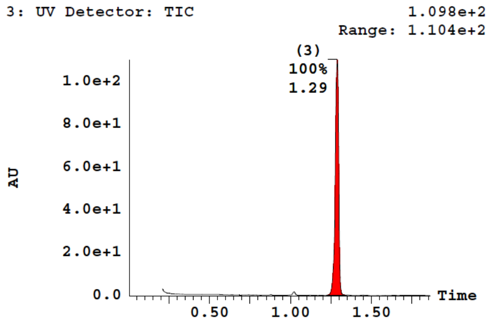

Peak ID	Time	Mass Found
3	1.28	

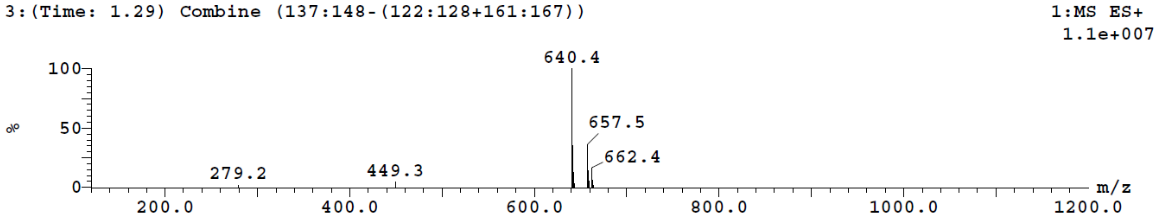

Compound **45**

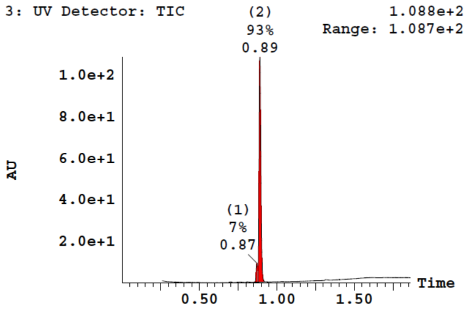

Peak ID	Time	Mass Found
2	0.89	

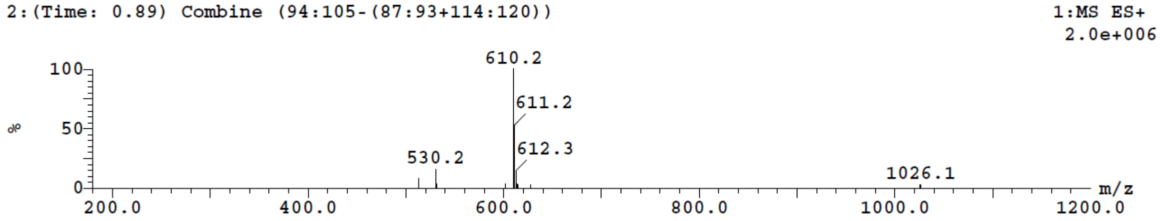

Compound **46**

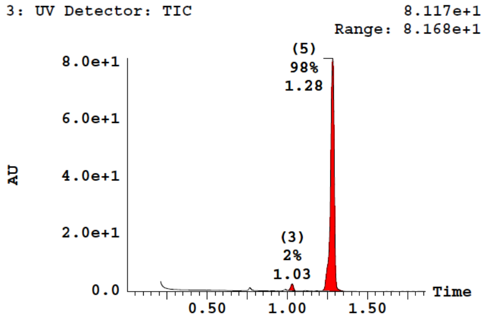

Peak ID	Time	Mass Found
5	1.28	

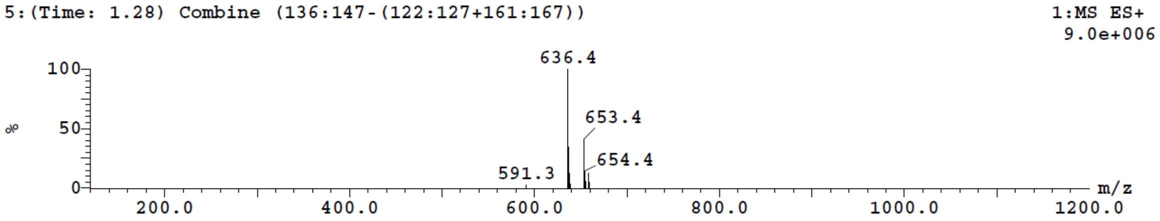

Compound **47**

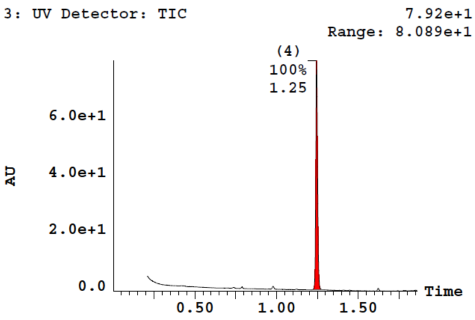

Peak ID	Time	Mass Found
4	1.25	

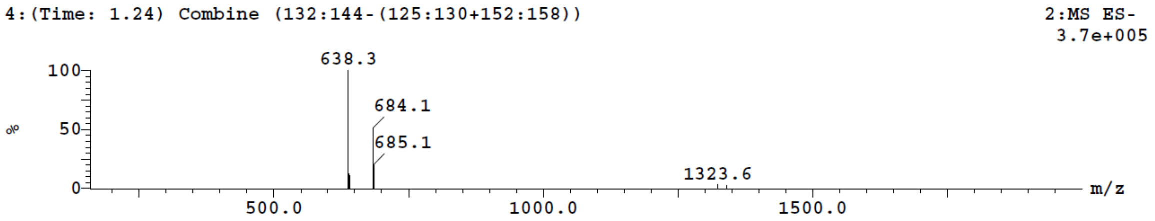

Compound **48**

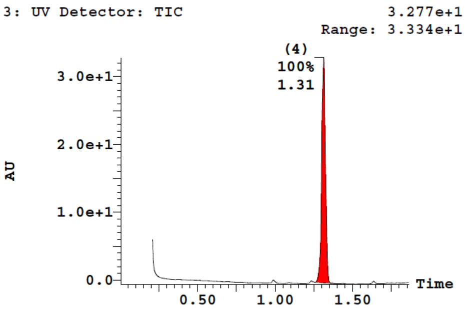

Peak ID	Time	Mass Found
4	1.30	

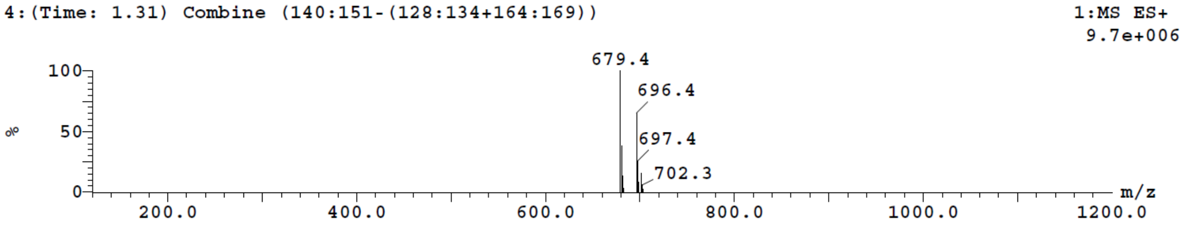

Compound **49**

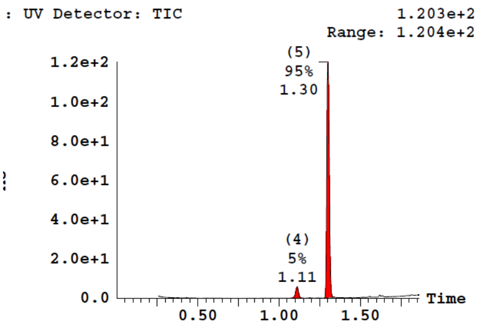

Peak ID	Time	Mass Found
5	1.30	

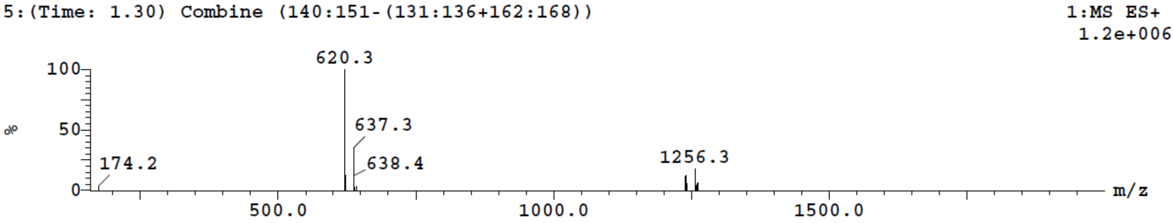

Compound **50**

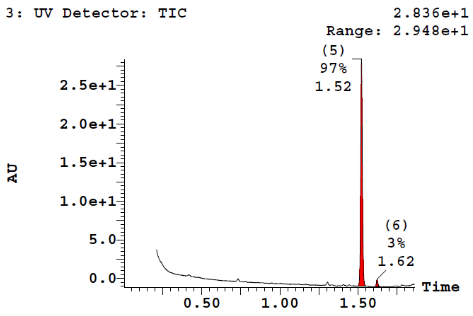

Peak ID	Time	Mass Found
5	1.52	

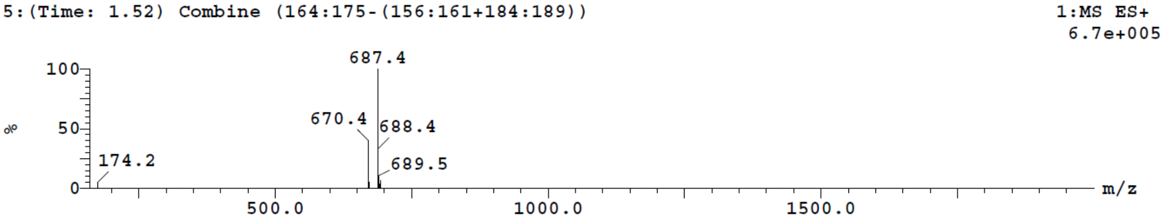

Compound **51**

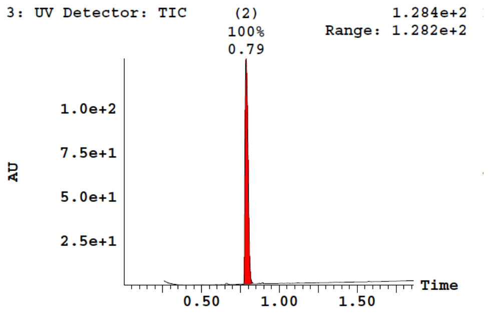

Peak ID	Time	Mass Found
2	0.79	

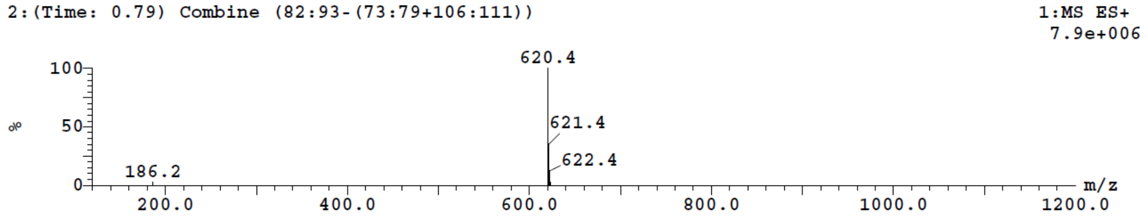

Compound **52**

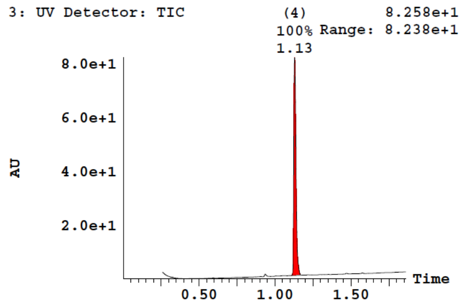

Peak ID	Time	Mass Found
4	1.12	

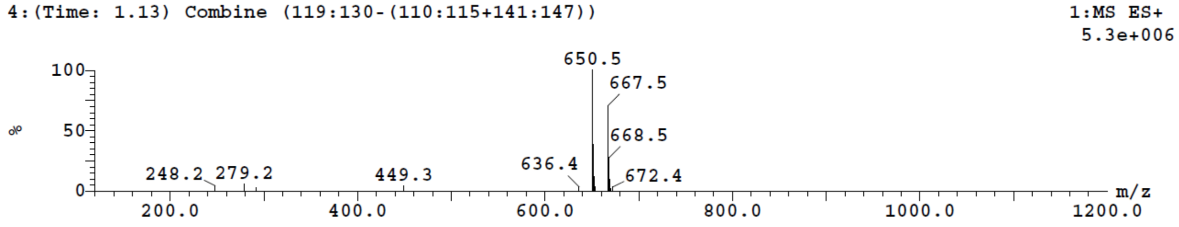

Compound **53**

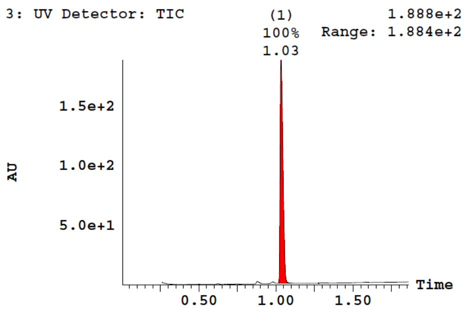

Peak ID	Time	Mass Found
1	1.03	

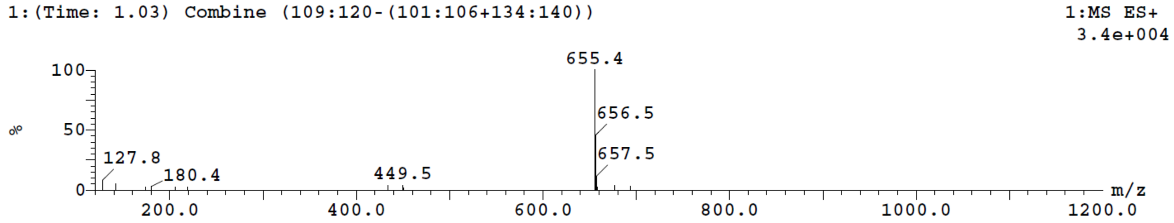

Compound **54**

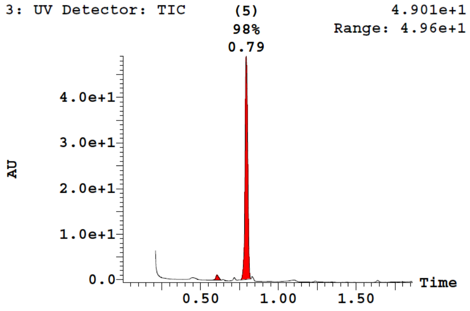

Peak ID	Time	Mass Found
5	0.80	

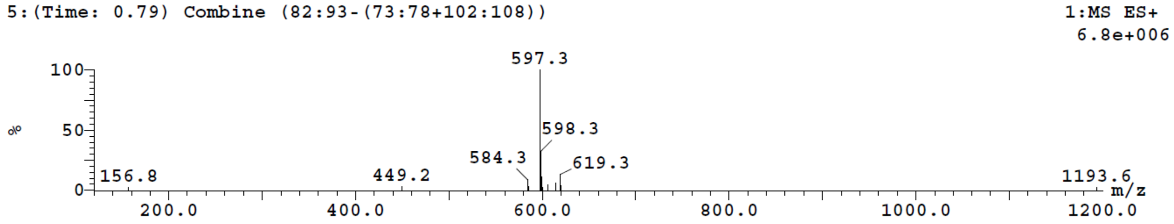

Compound **55**

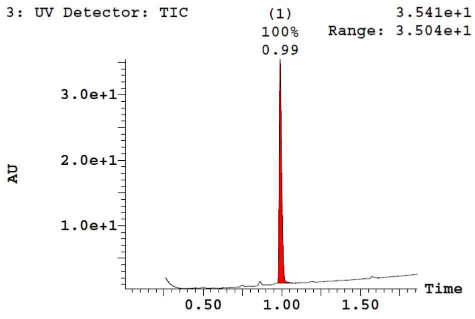

Peak ID	Time	Mass Found
1	0.99	

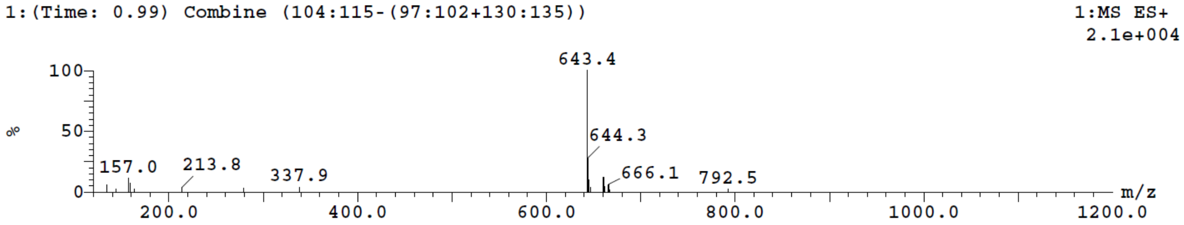

Compound **56**

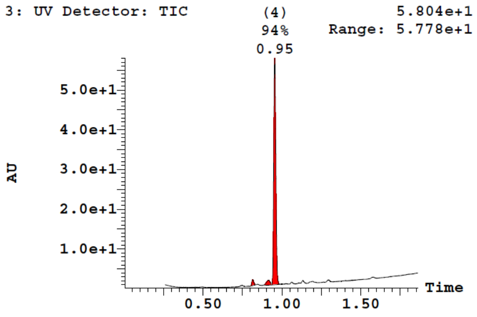

Peak ID	Time	Mass Found
4	0.96	

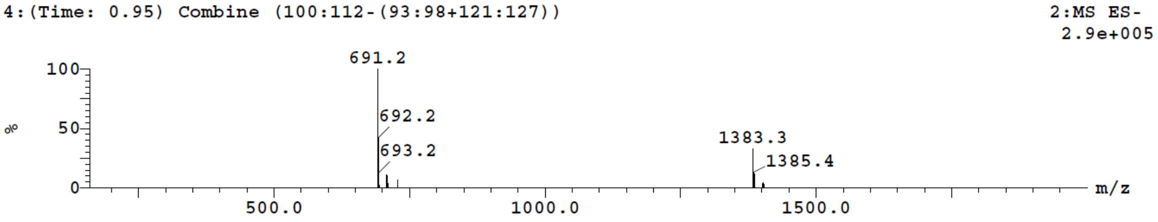

Compound **57**

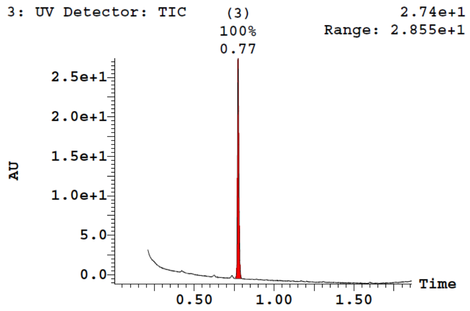

Peak ID	Time	Mass Found
3	0.77	

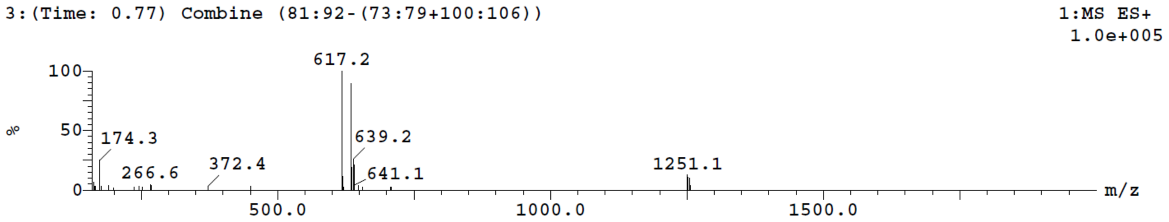

Compound **58**

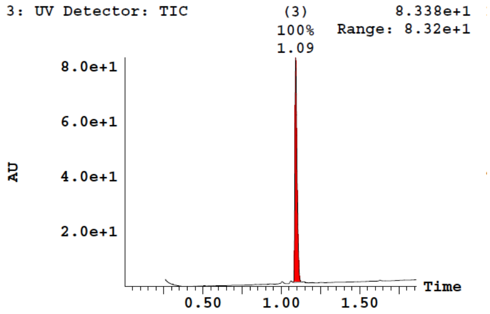

Peak ID	Time	Mass Found
3	1.09	

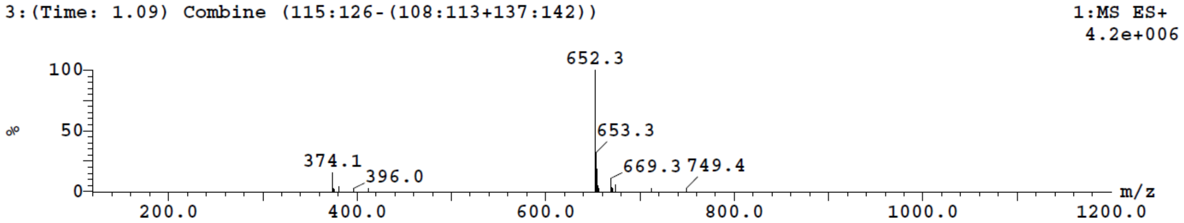

Compound **59**

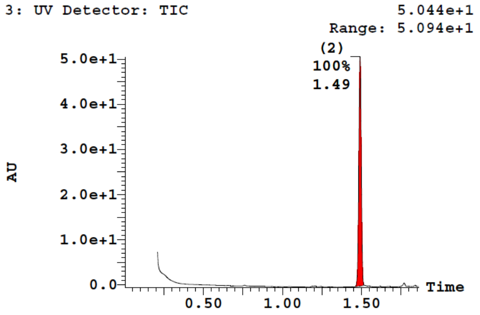

Peak ID	Time	Mass Found
2	1.49	

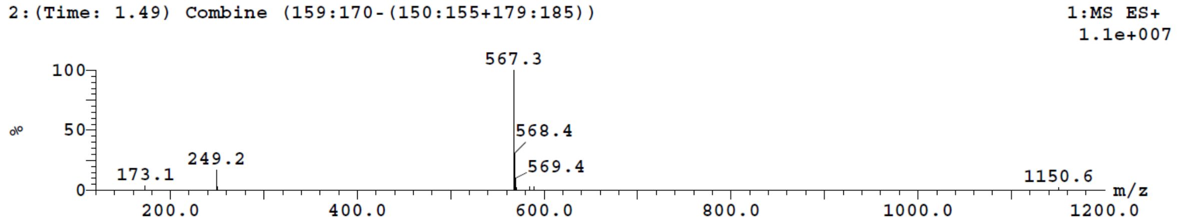

Compound **60**

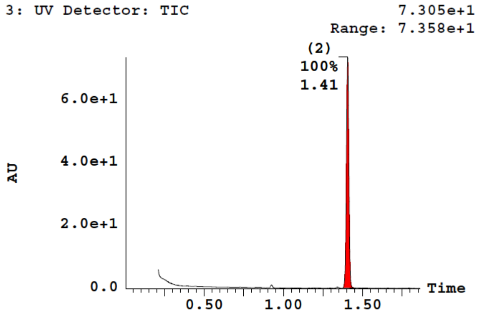

Peak ID	Time	Mass Found
2	1.40	

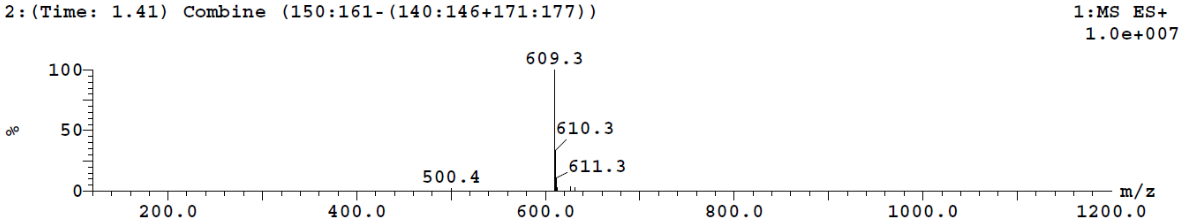

Compound **61**

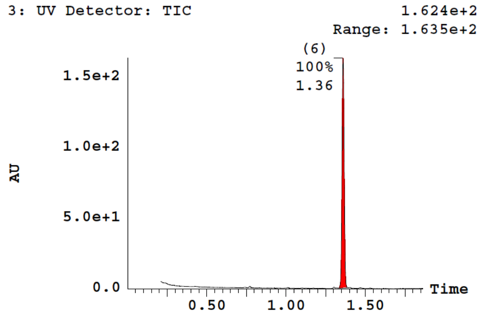

Peak ID	Time	Mass Found
6	1.36	

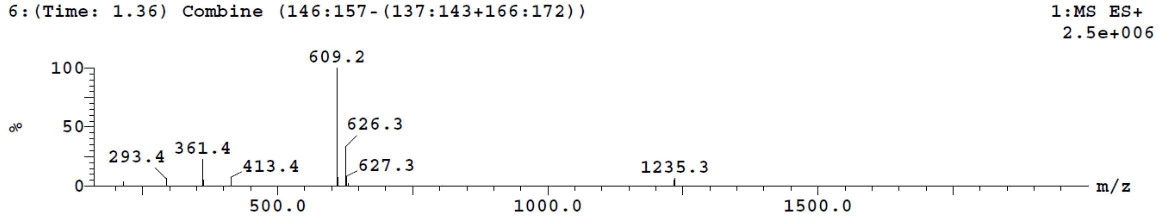

Compound **62**

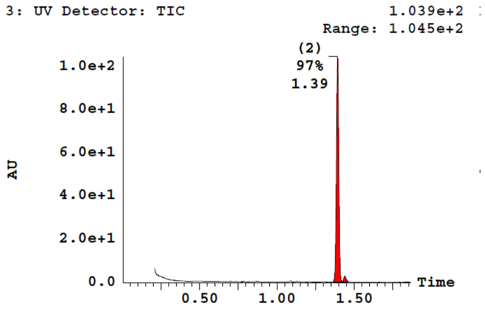

Peak ID	Time	Mass Found
2	1.38	

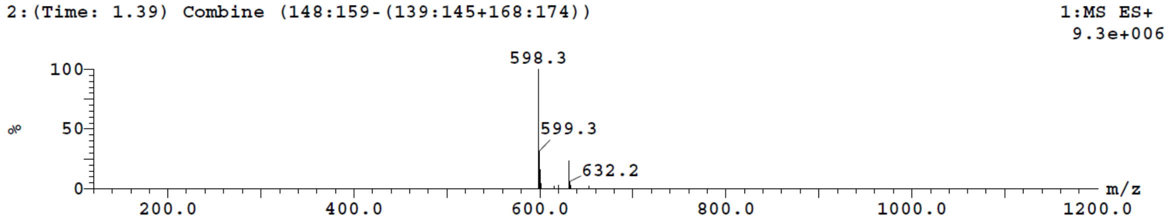

Peak ID Time Mass Found

Compound **63**

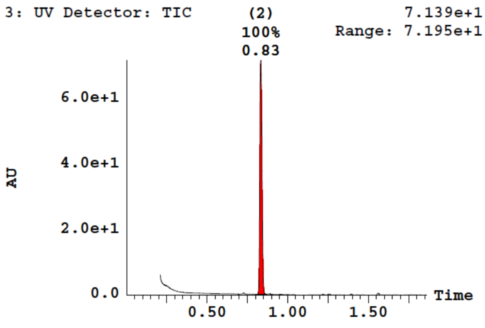

Peak ID	Time	Mass Found
2	0.84	

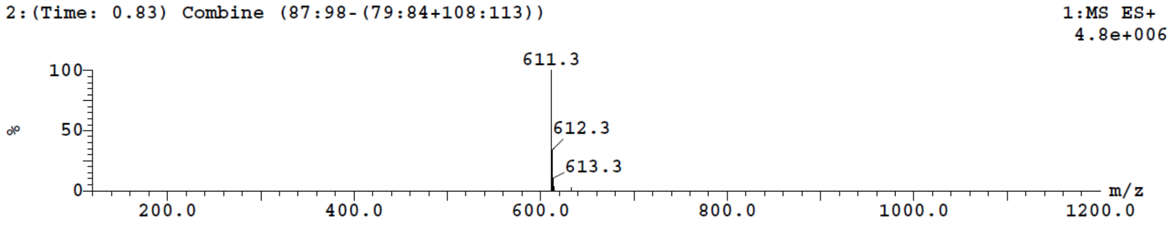

Compound **64**

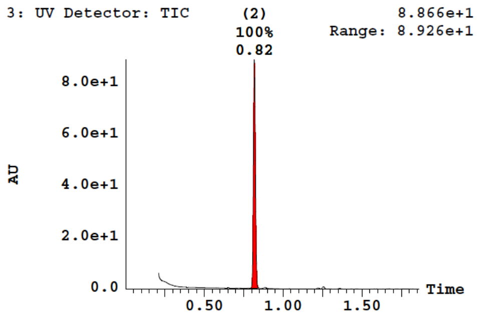

Sample Report (continued):

Peak ID	Time	Mass Found
2	0.81	

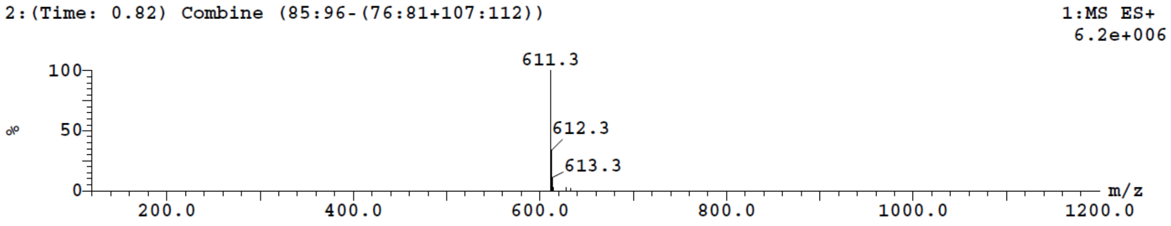

Compound **65**

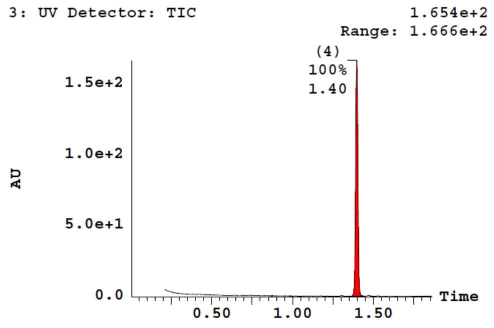

Peak ID	Time	Mass Found
4	1.39	

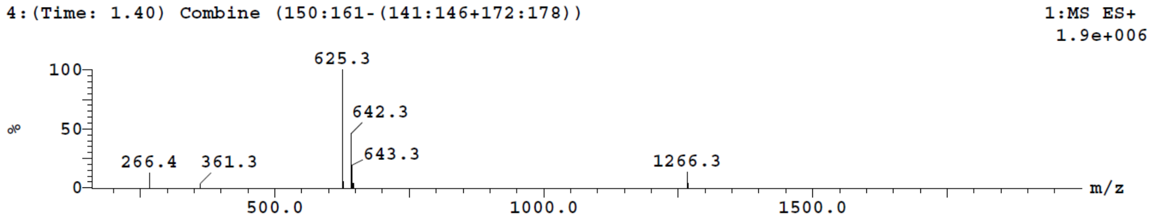

Compound **66**

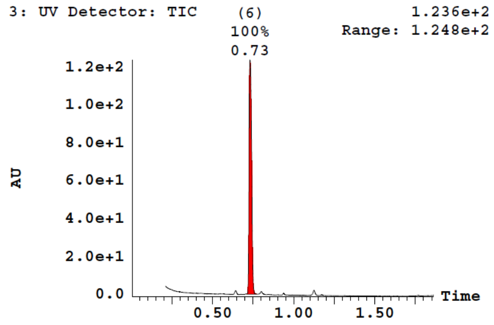

Peak ID	Time	Mass Found
6	0.73	

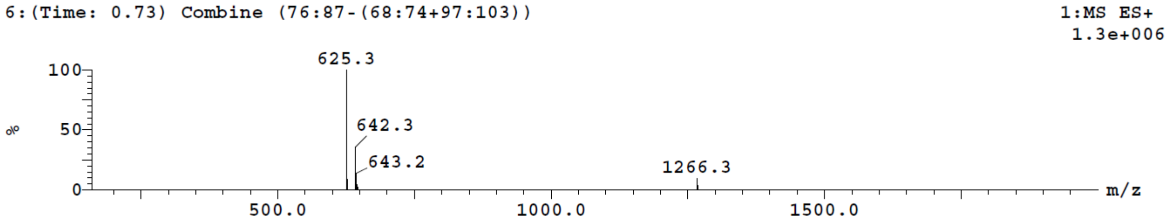

Compound **67**

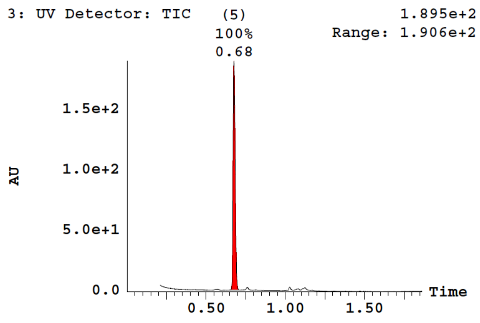

Sample Report (continued):

Peak ID	Time	Mass Found
5	0.68	

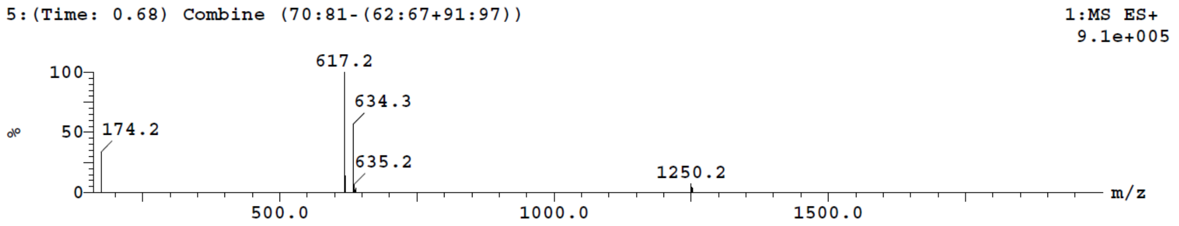

Compound **68**

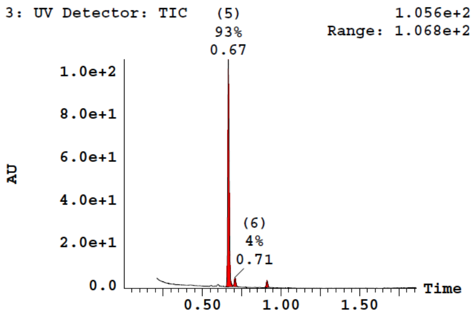

Peak ID	Time	Mass Found
5	0.67	

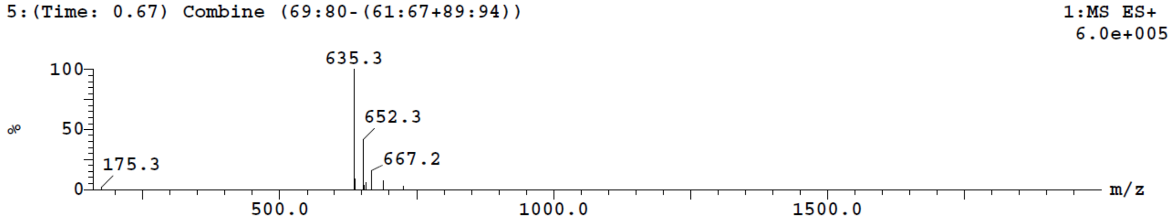

Compound **69**

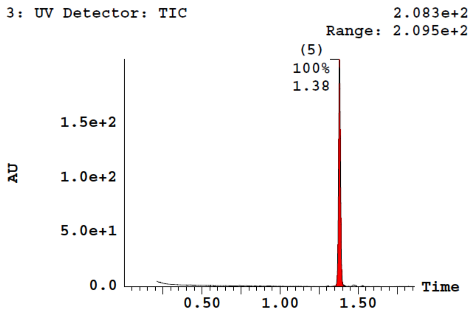

Peak ID	Time	Mass Found
5	1.38	

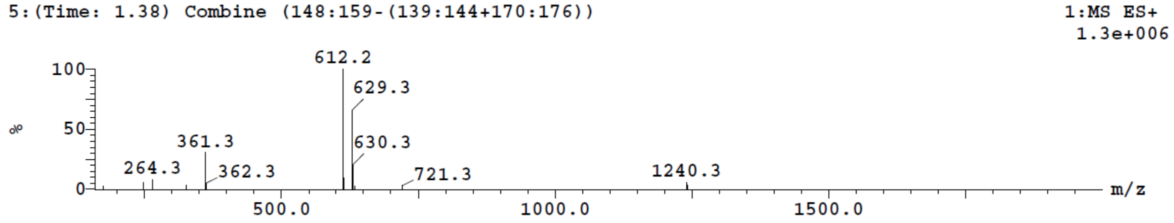

Compound **70**

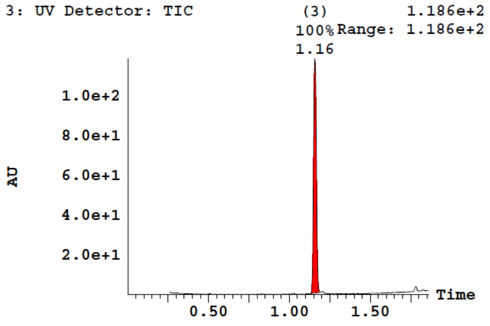

Peak ID	Time	Mass Found
3	1.17	

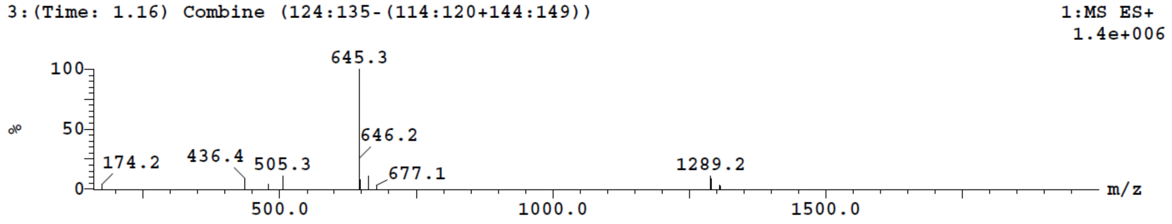

Compound **71**

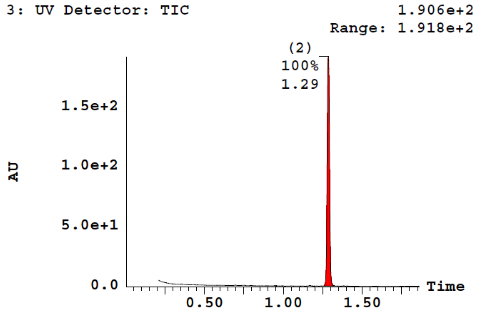

Peak ID	Time	Mass Found
2	1.27	

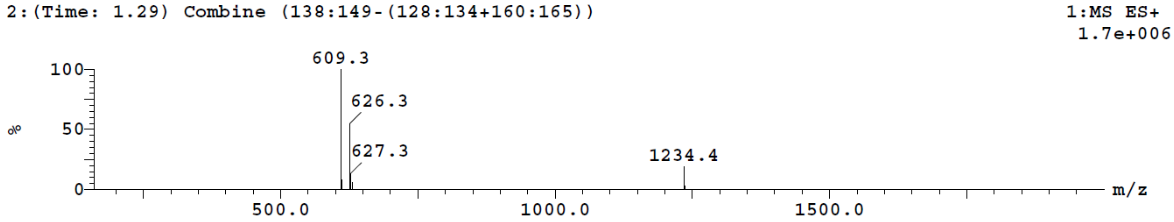

Compound **72**

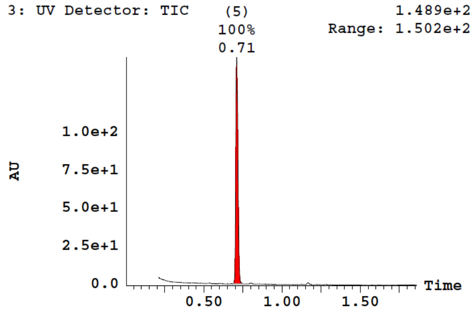

Peak ID	Time	Mass Found
5	0.71	

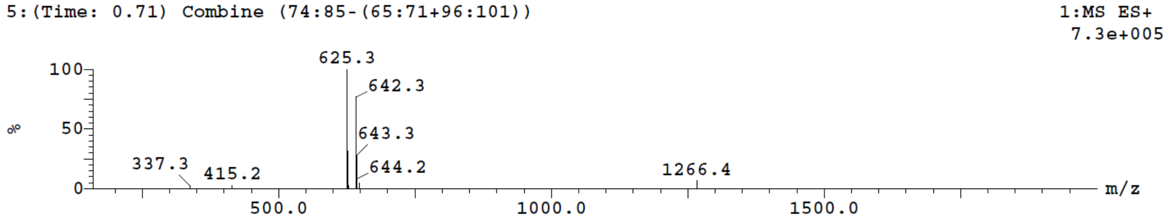

Compound **73**

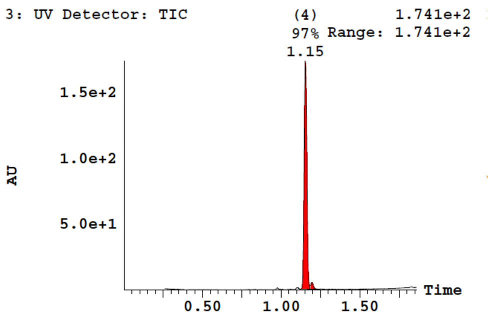

Peak ID	Time	Mass Found
4	1.15	

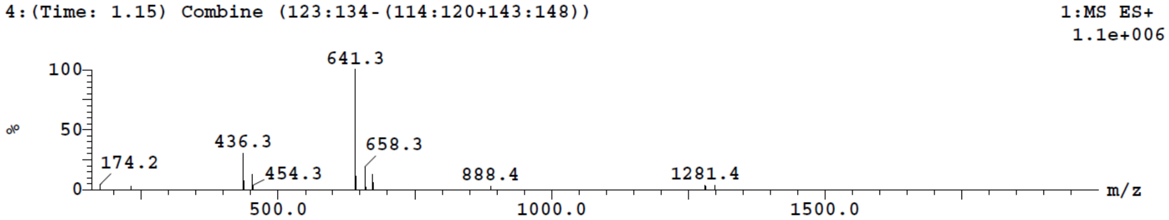

Compound **74**

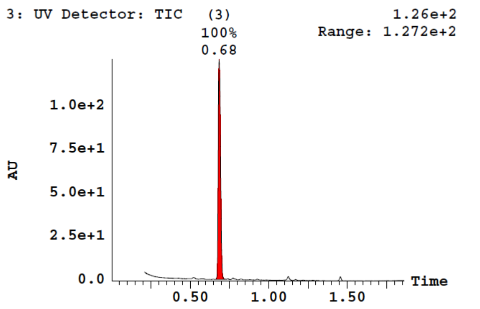

Peak ID	Time	Mass Found
3	0.69	

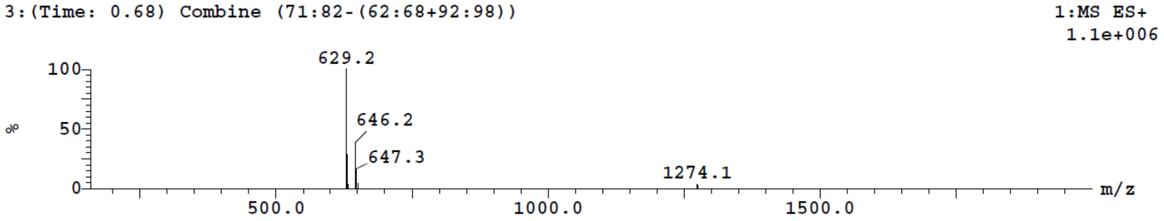

Compound **75**

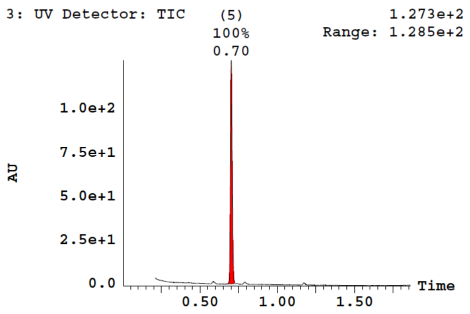

Peak ID	Time	Mass Found
5	0.67	

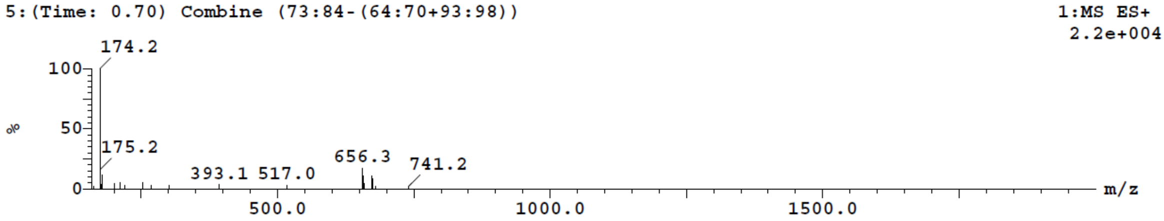

Compound **76**

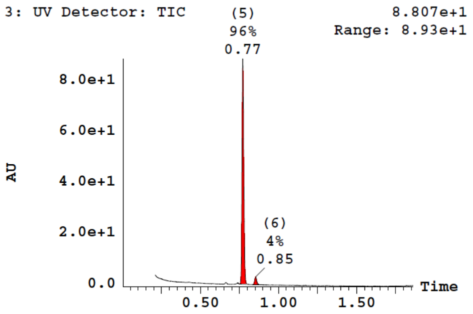

Peak ID	Time	Mass Found
5	0.77	

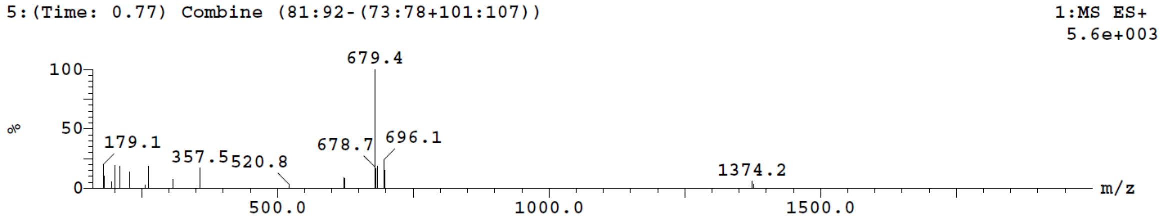

Compound **77**

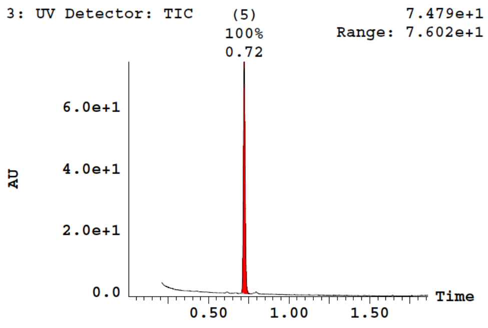

Peak ID	Time	Mass Found
5	0.72	

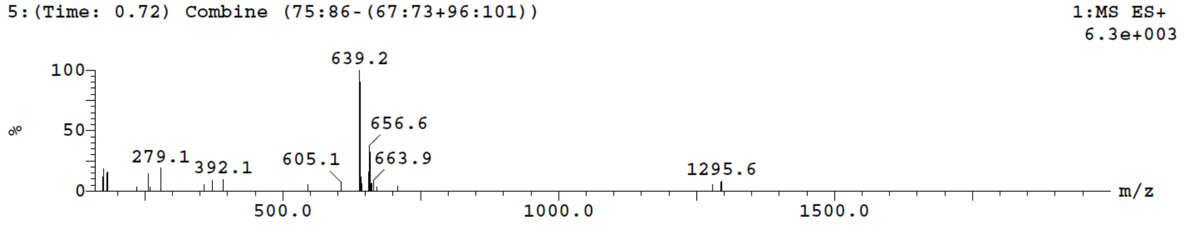

Compound **78**

Sample ID PLATE 7 A1 Description

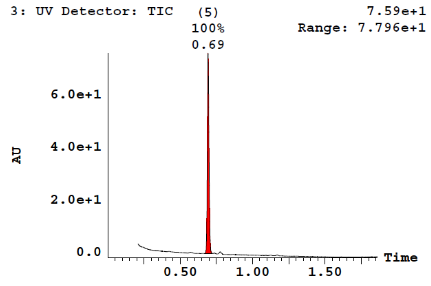

Peak ID	Time	Mass Found
5	0.69	

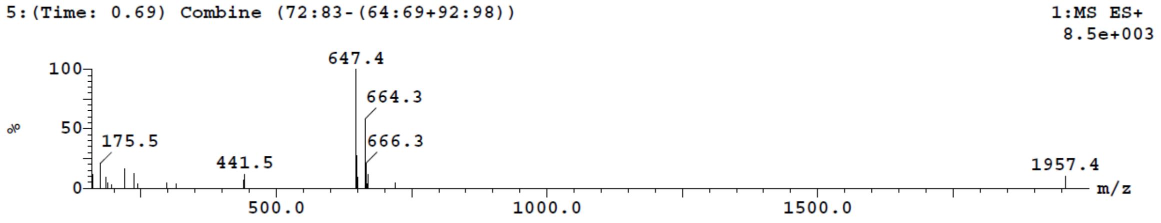

NMR spectra for compounds S1–S24

S1 ^{1}H NMR (500 MHz, $CDCl_3$)

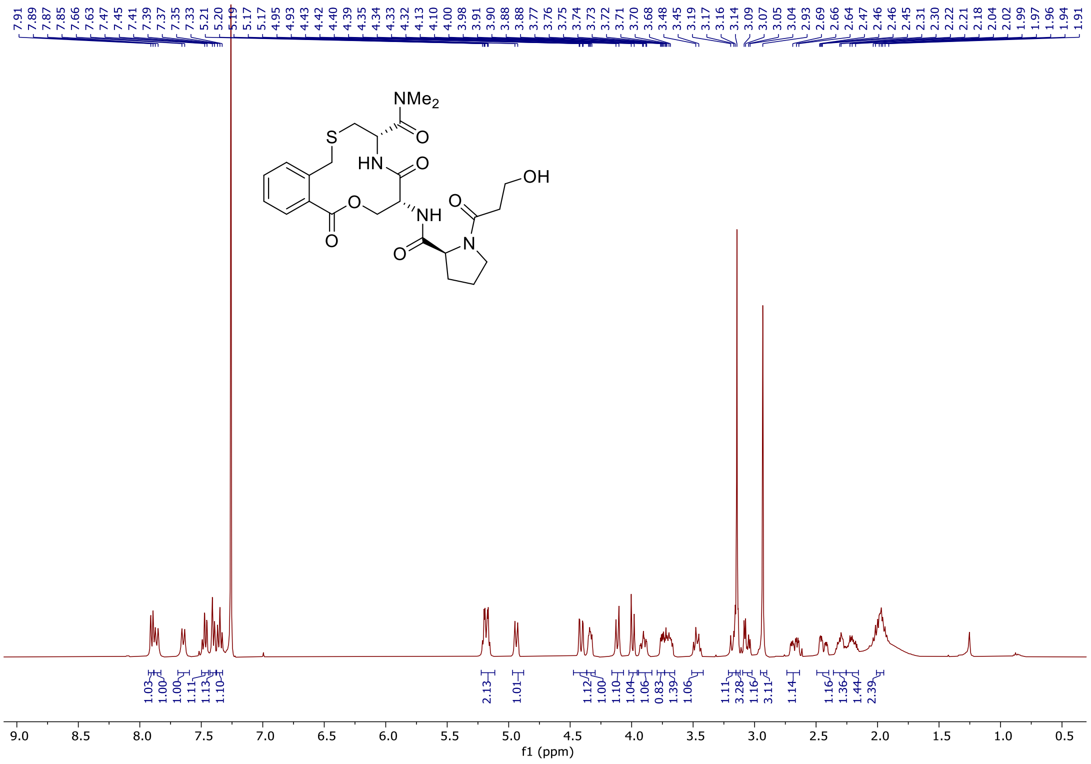

S1 ^{13}C NMR (126 MHz, $CDCl_3$)

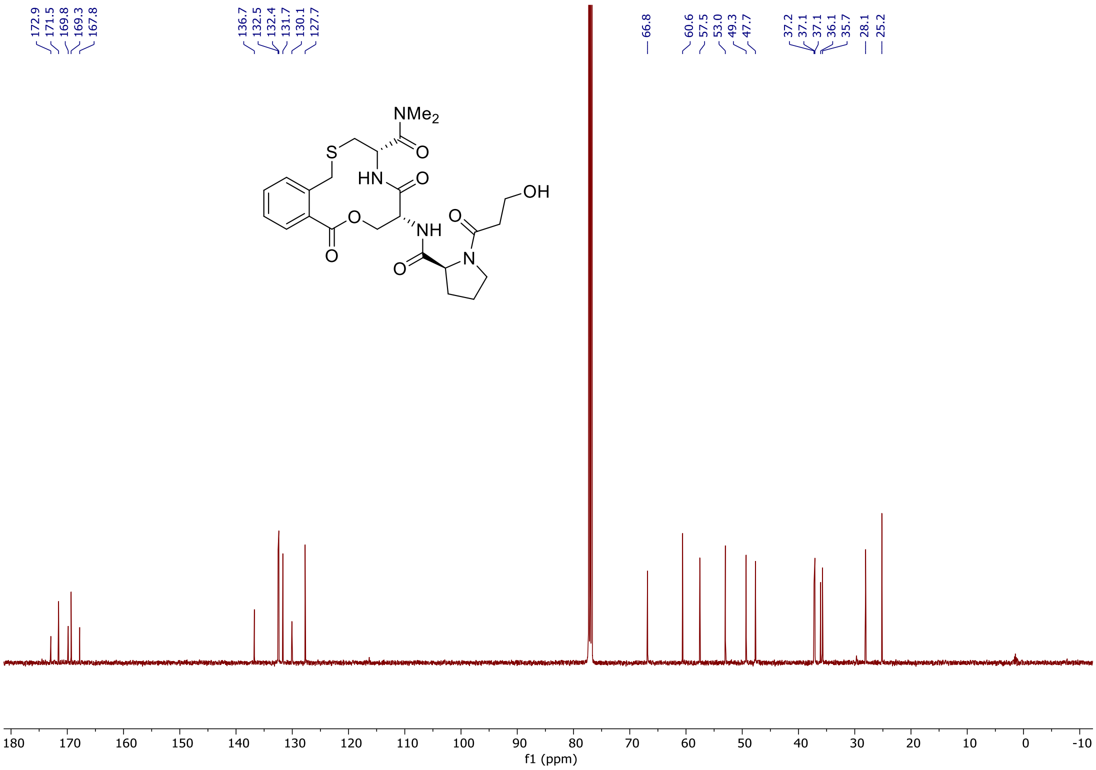

S2 ^{1}H NMR (500 MHz, $CDCl_3$)

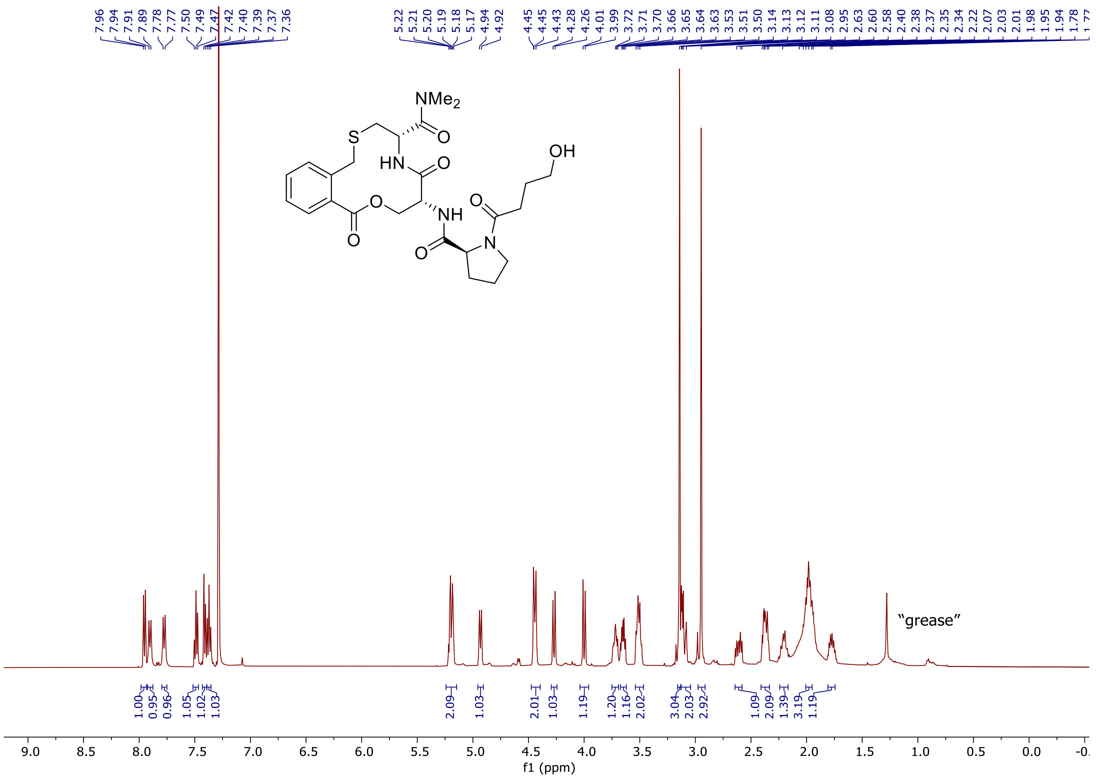

S2 ^{13}C NMR (126 MHz, $CDCl_3$)

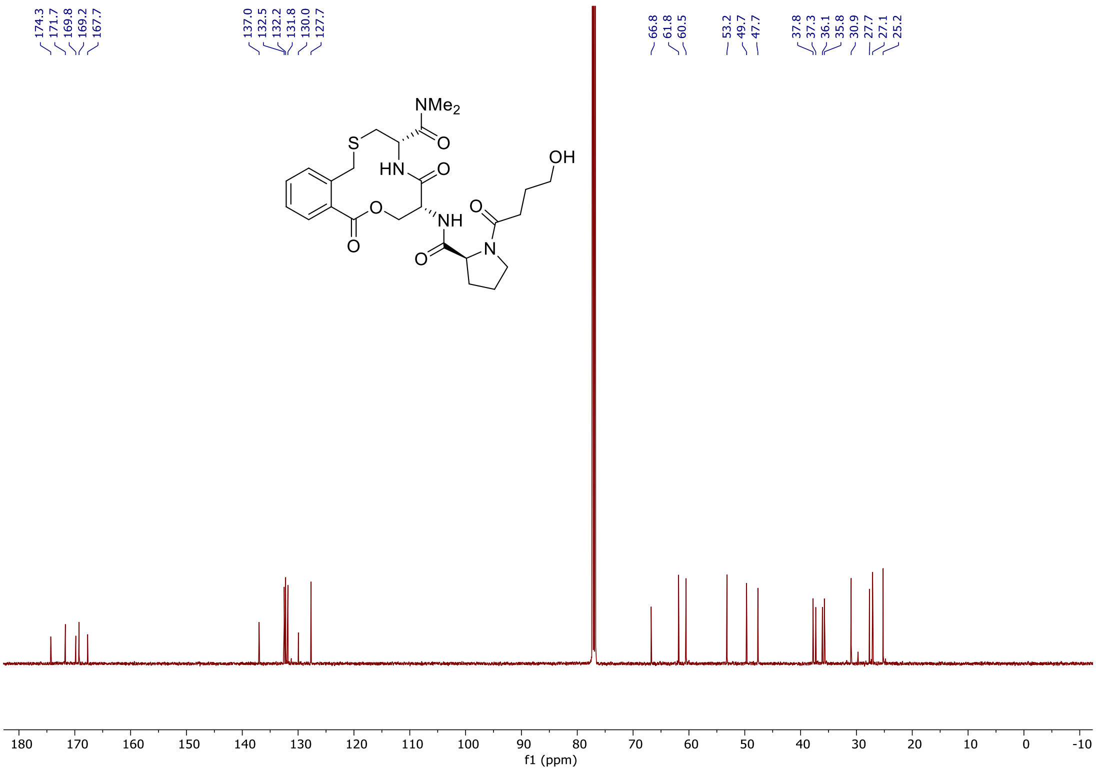

S3 ^{1}H NMR (500 MHz, CDCl$_3$)

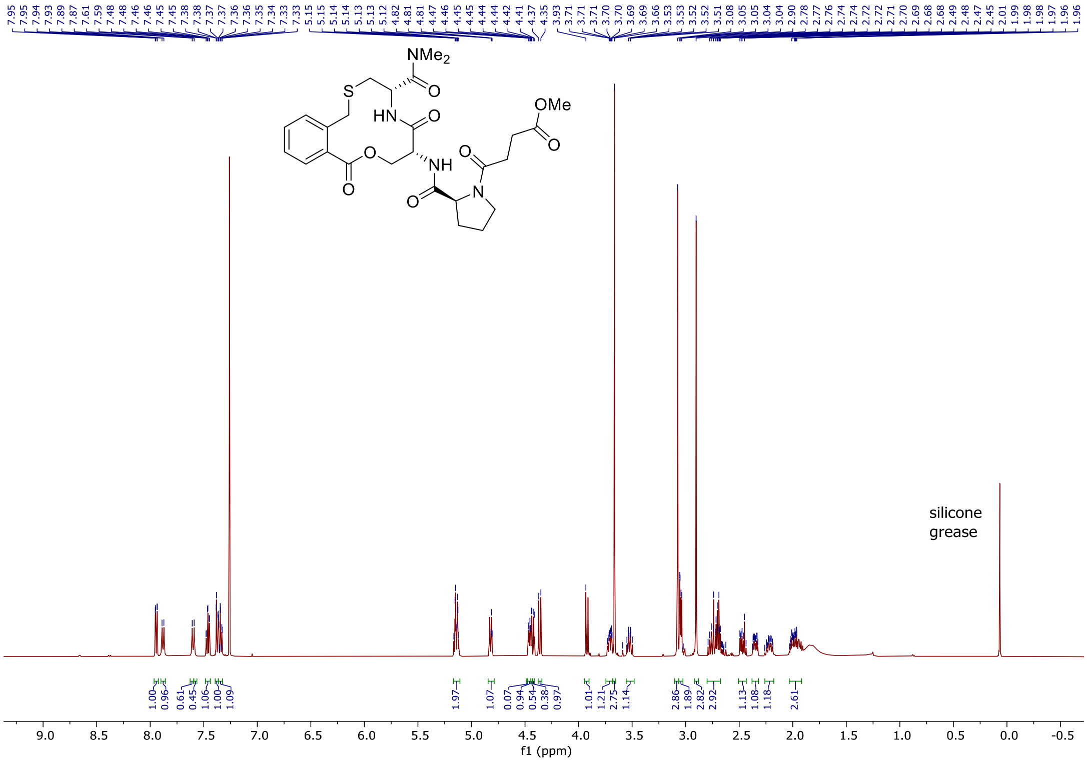

S3 ^{13}C NMR (126 MHz, CDCl$_3$)

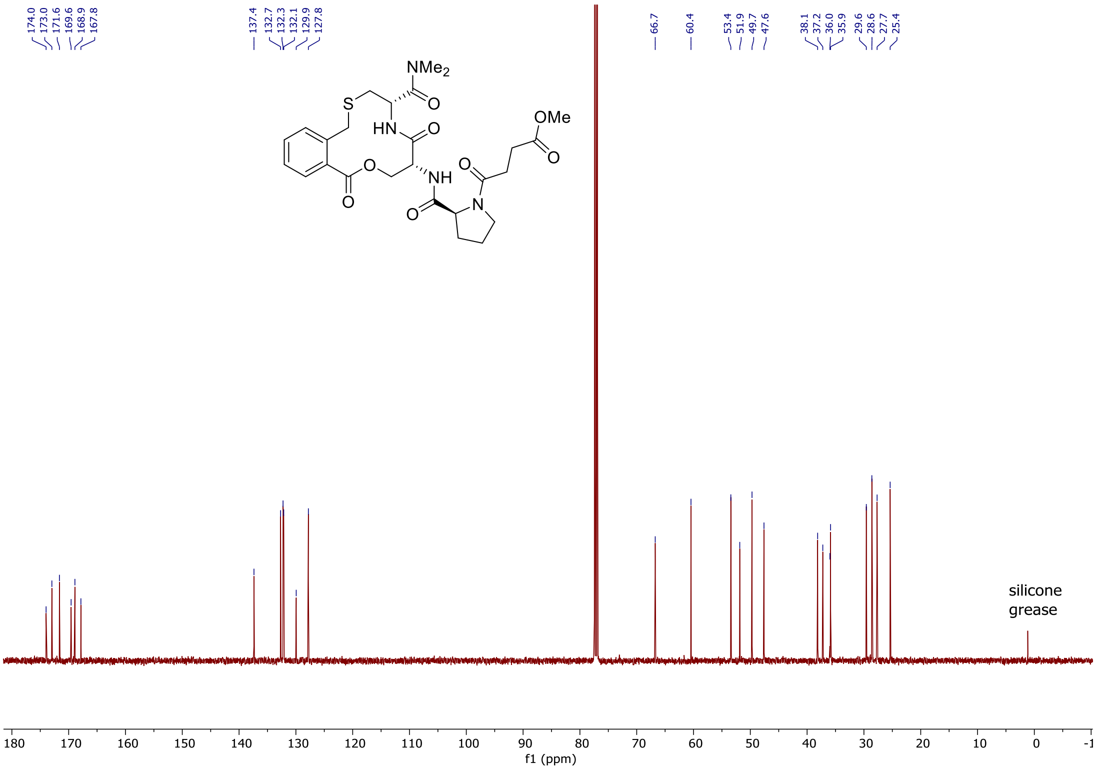

S4 ^{1}H NMR (500 MHz, CDCl$_3$)

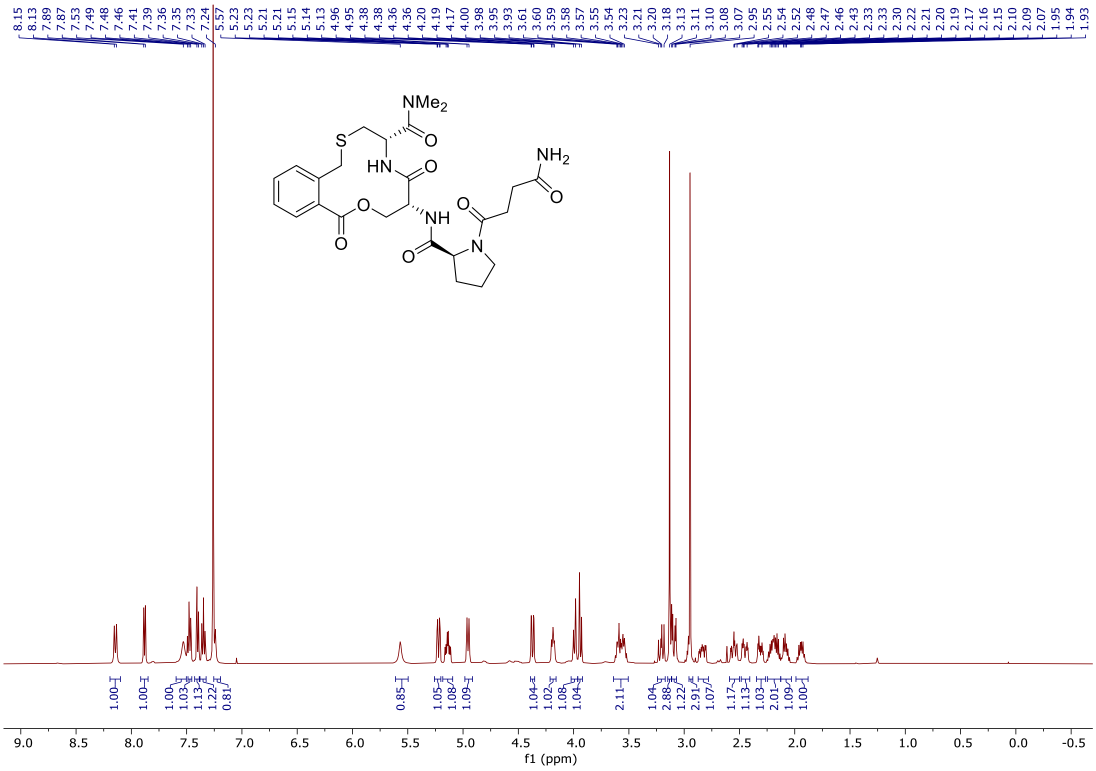

S4 ^{13}C NMR (126 MHz, CDCl$_3$)

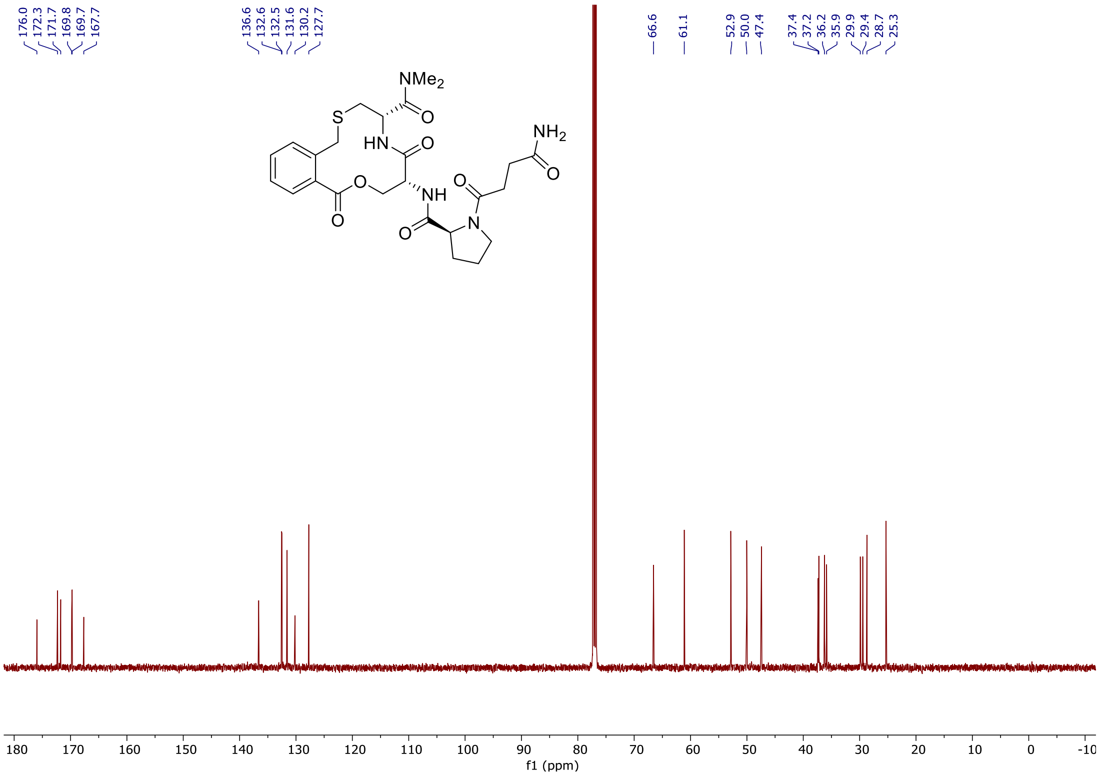

S5 ^{1}H NMR (500 MHz, $CDCl_3$)

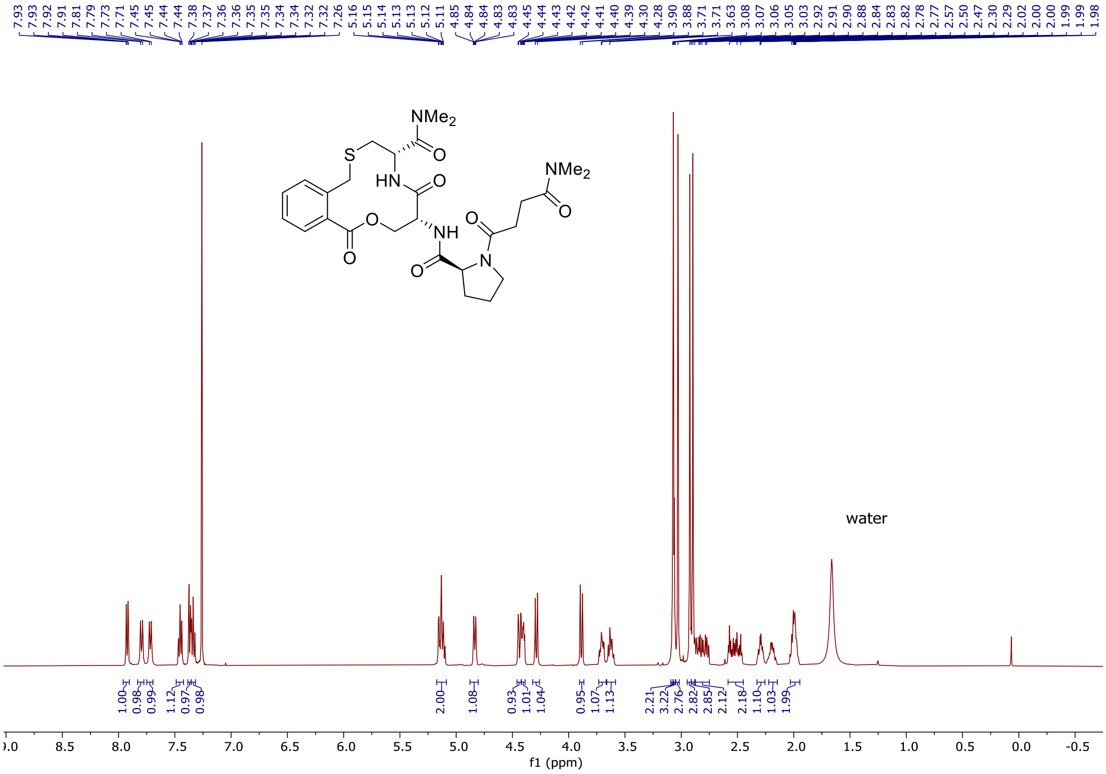

S5 ^{13}C NMR (126 MHz, $CDCl_3$)

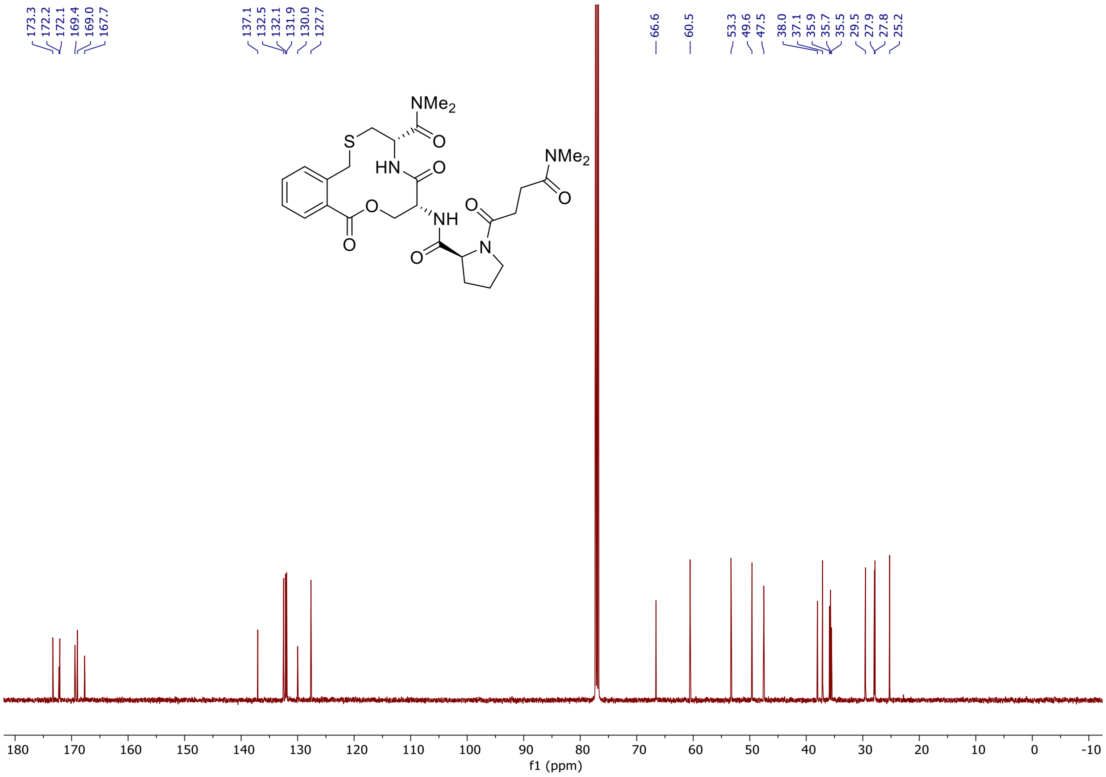

S6 ^{1}H NMR (600 MHz, DMSO-d_6)

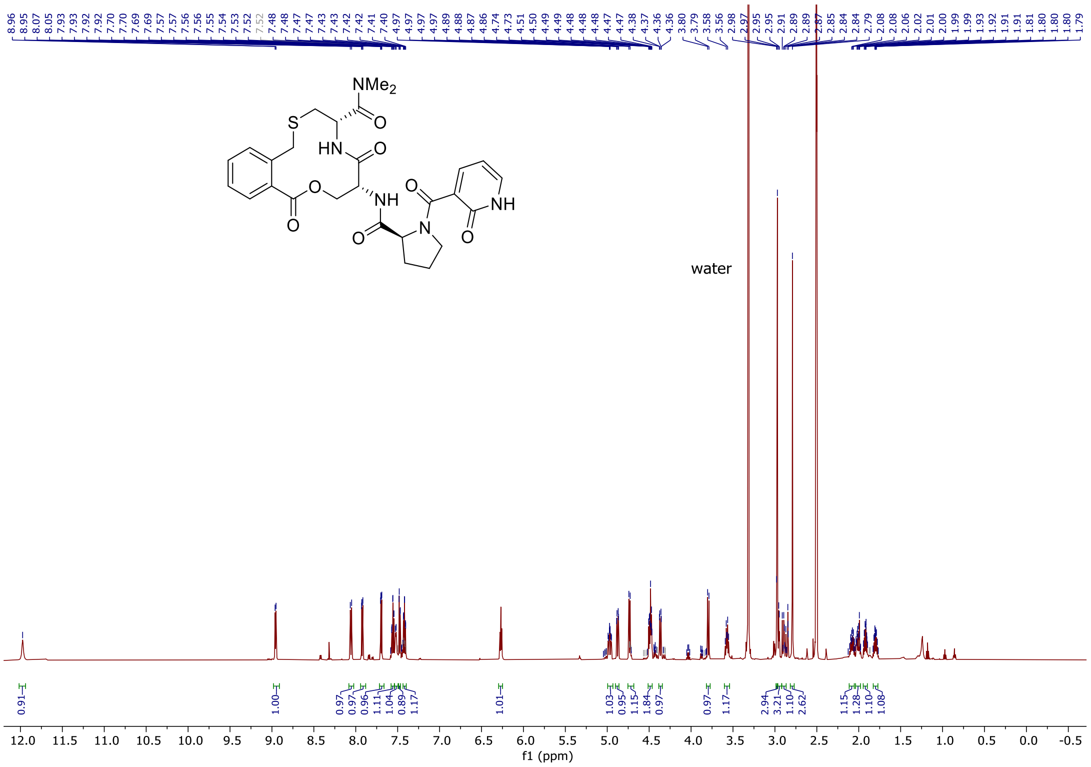

S6 ^{13}C NMR (151 MHz, DMSO-d_6)

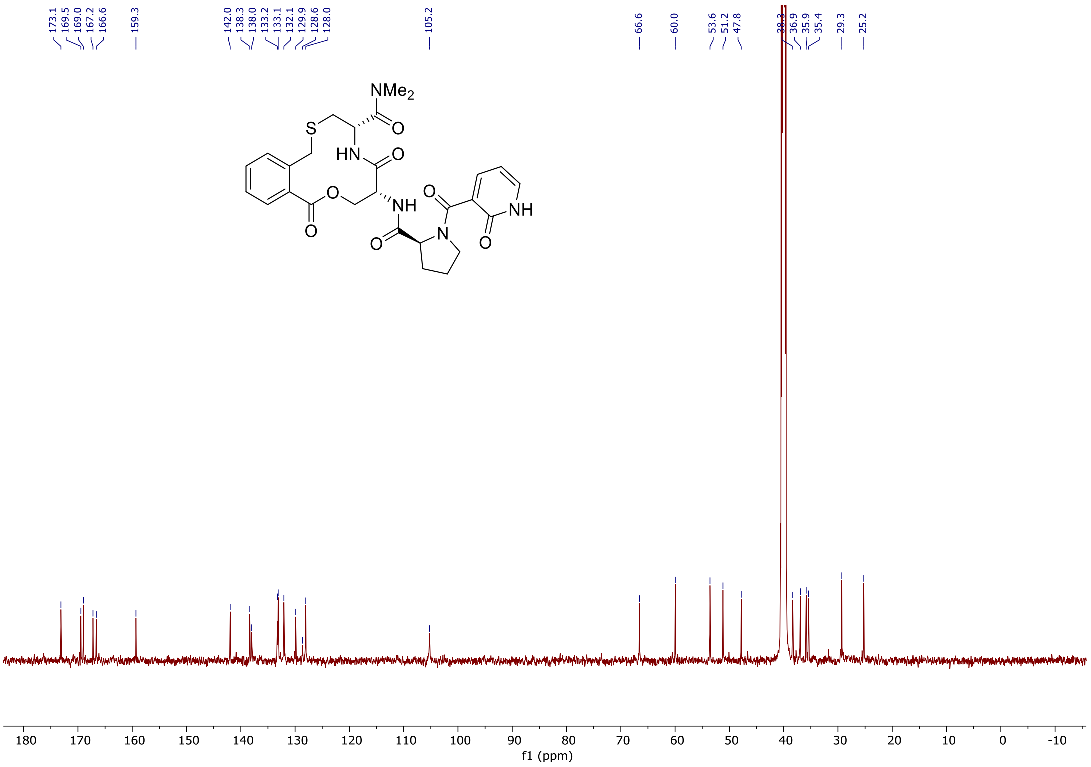

S7 ^{1}H NMR (600 MHz, $CDCl_3$)

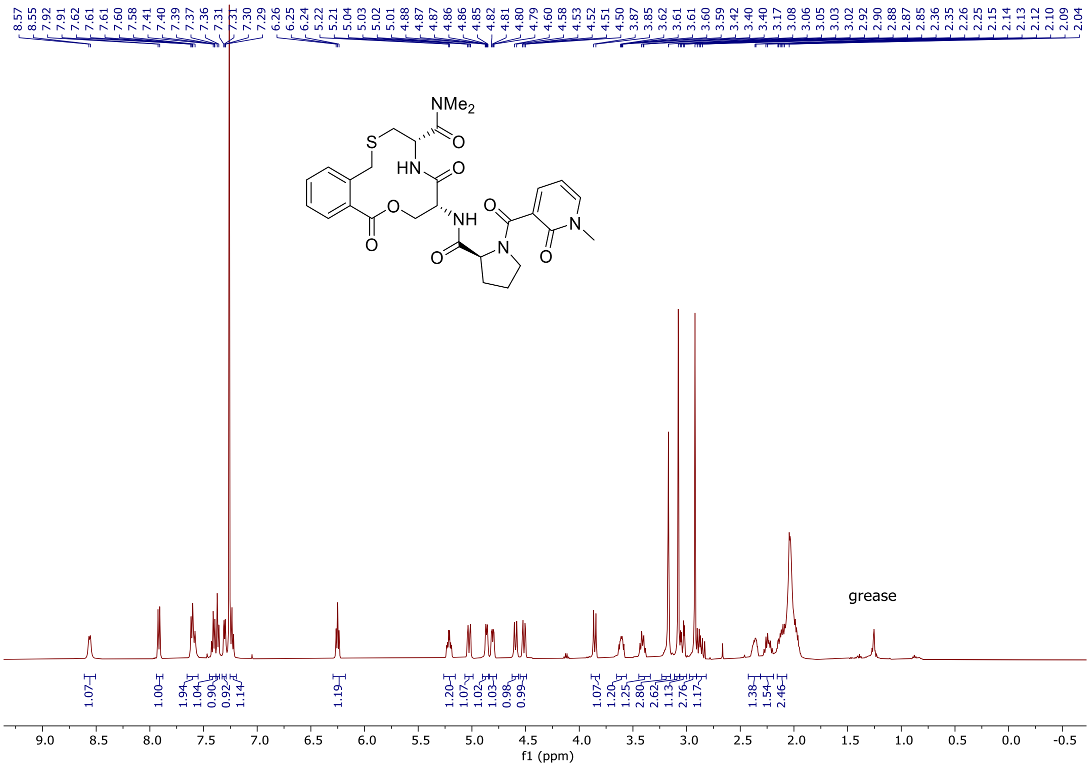

S7 ^{13}C NMR (151 MHz, $CDCl_3$)

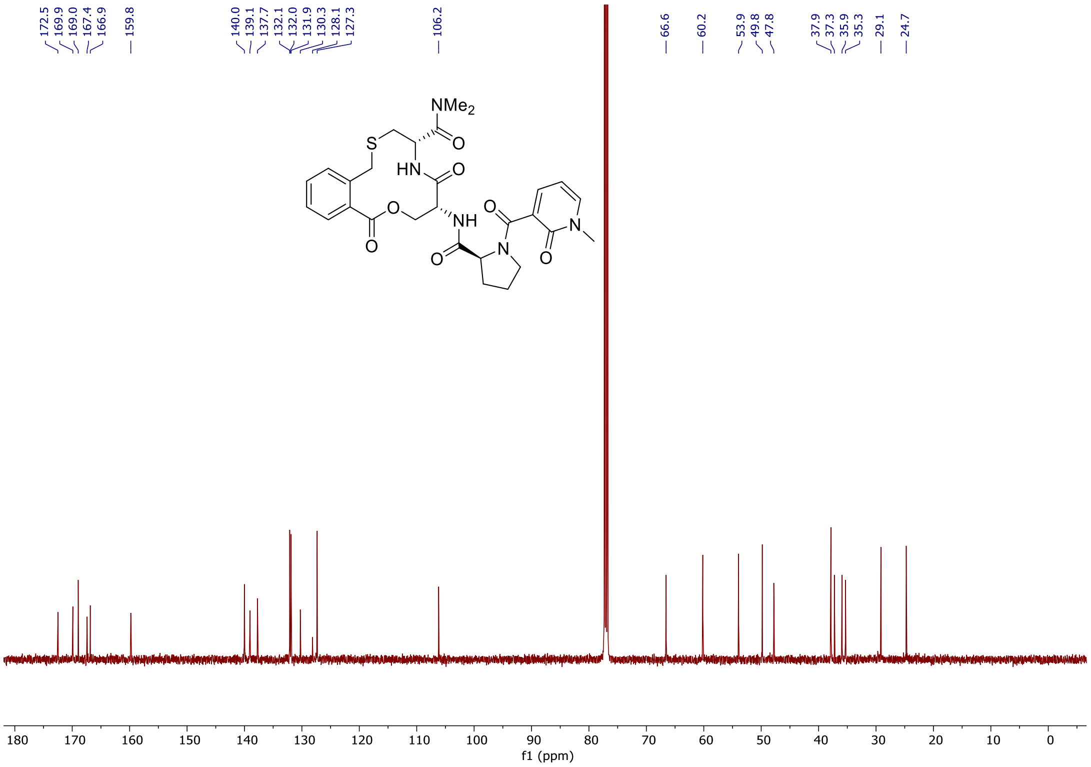

S8 ^{1}H NMR (500 MHz, $CDCl_3$)

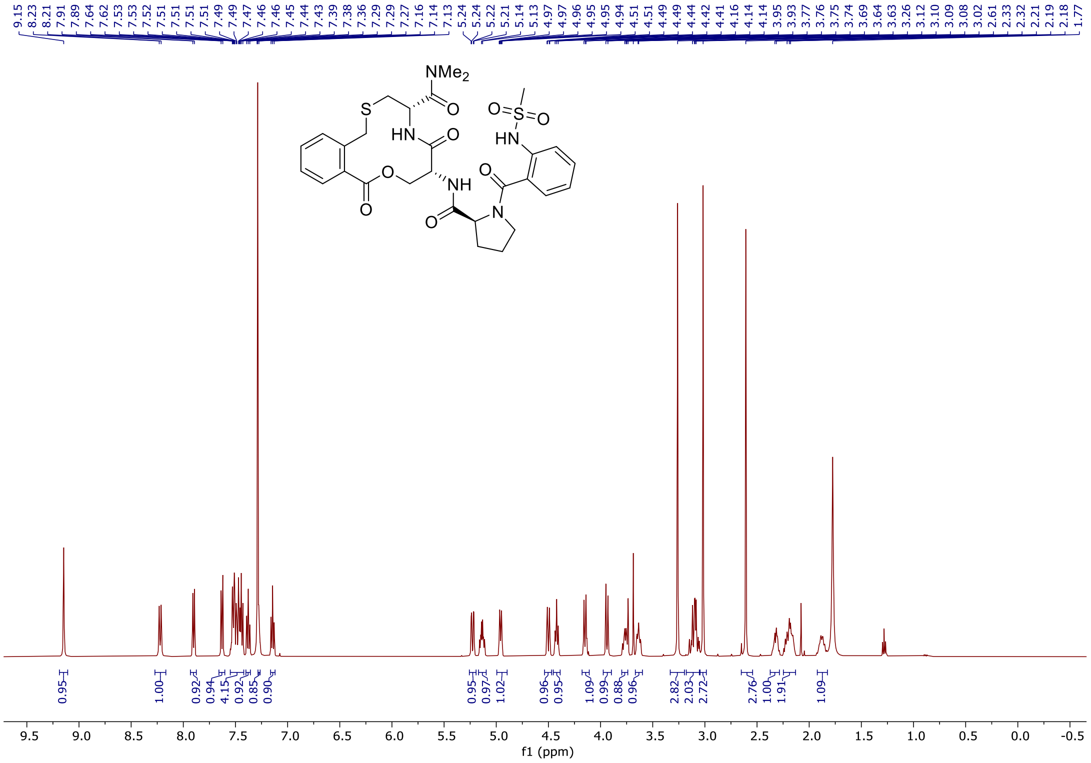

S8 ^{13}C NMR (126 MHz, $CDCl_3$)

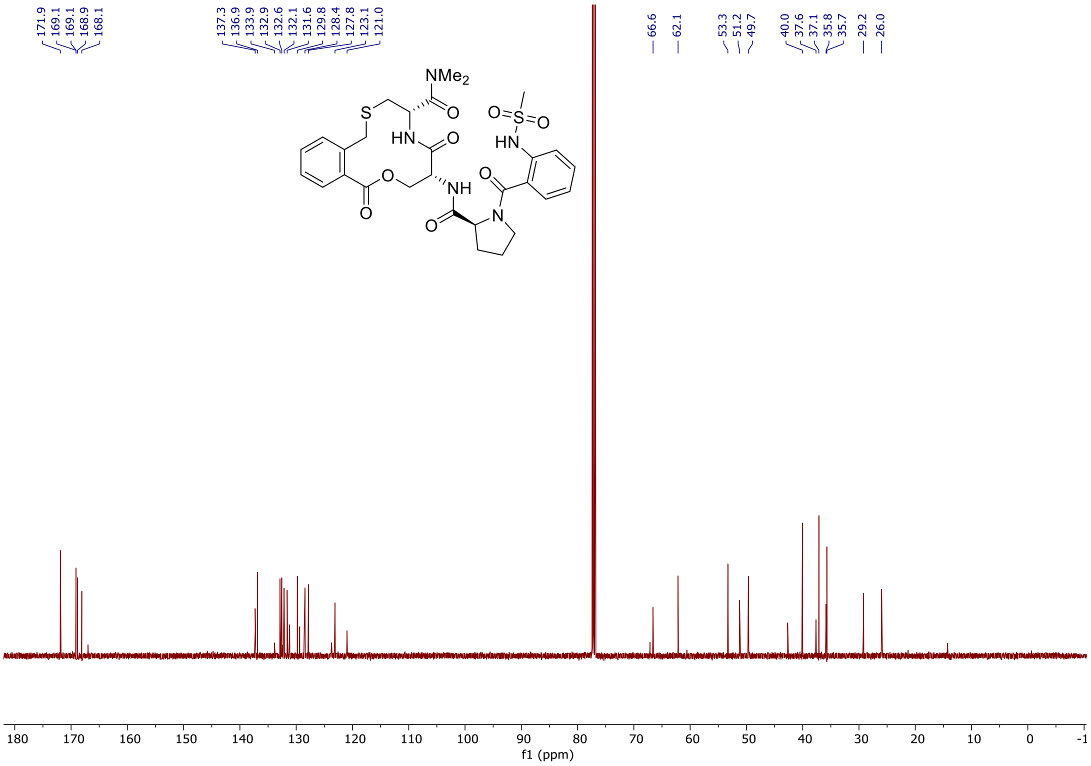

S9 ^{1}H NMR (500 MHz, $CDCl_3$)

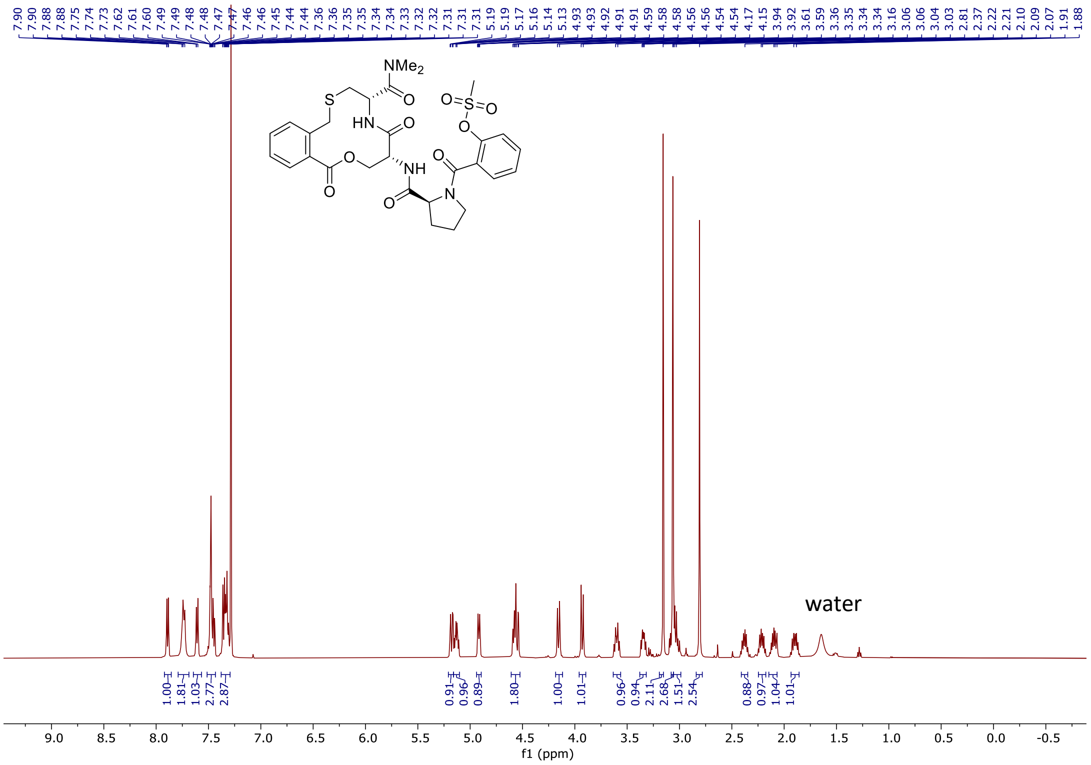

S9 ^{13}C NMR (126 MHz, $CDCl_3$)

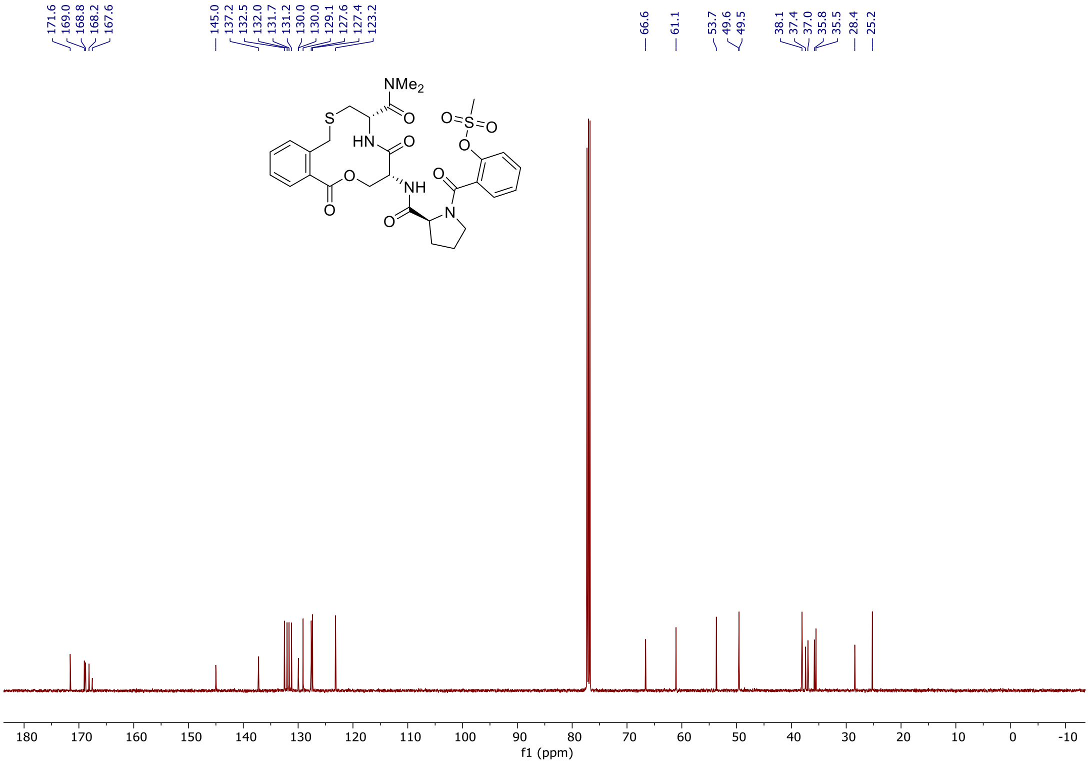

S10 ^{1}H NMR (500 MHz, $CDCl_3$)

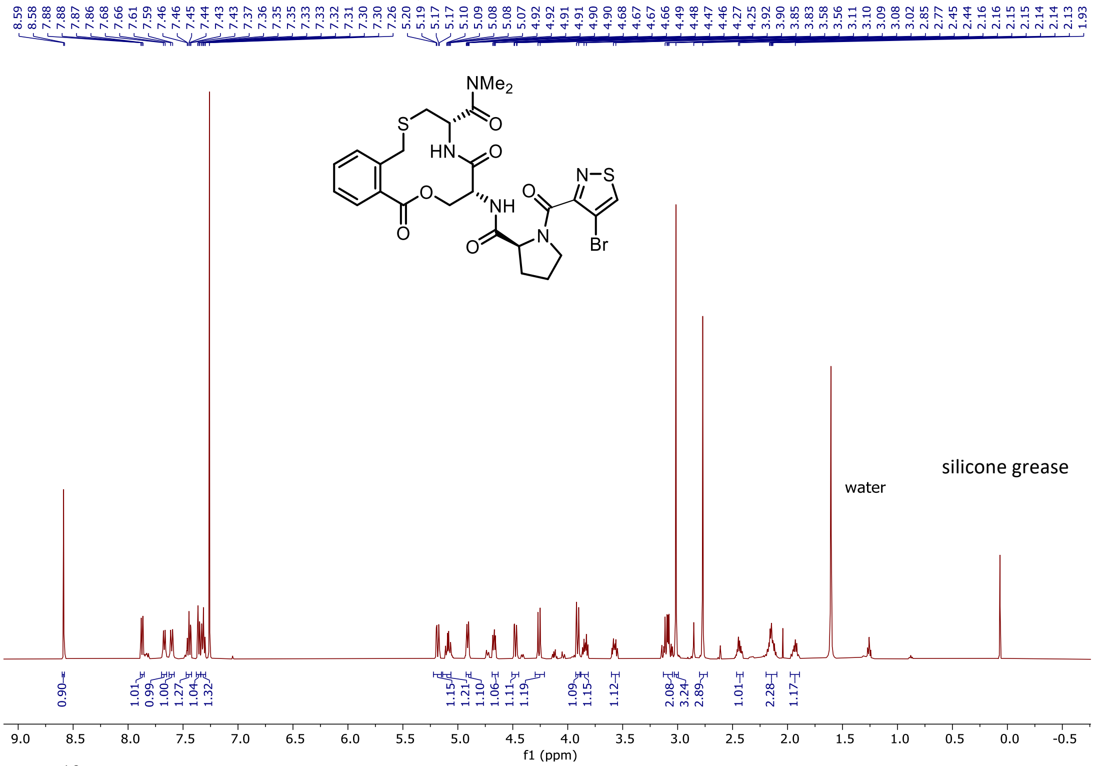

S10 ^{13}C NMR (126 MHz, $CDCl_3$)

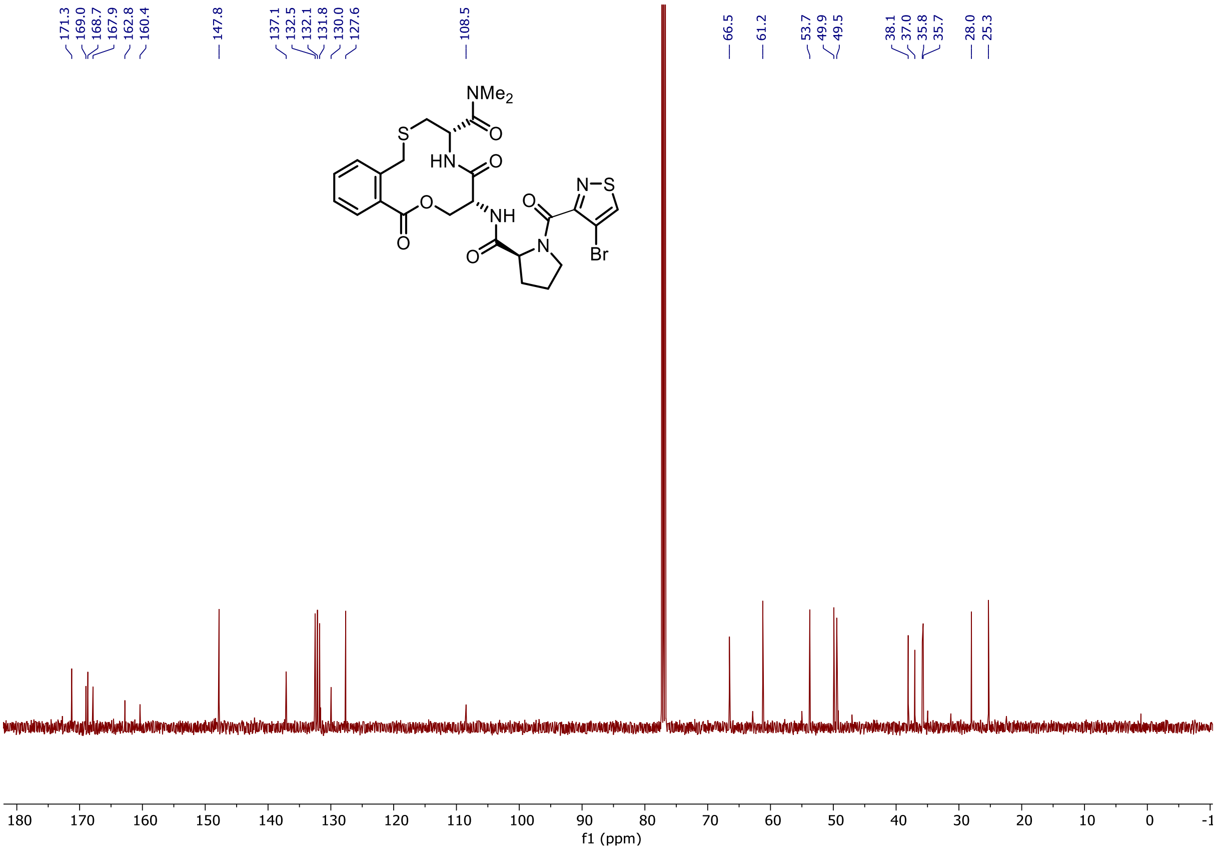

S11 ^{1}H NMR (600 MHz, $CDCl_3$)

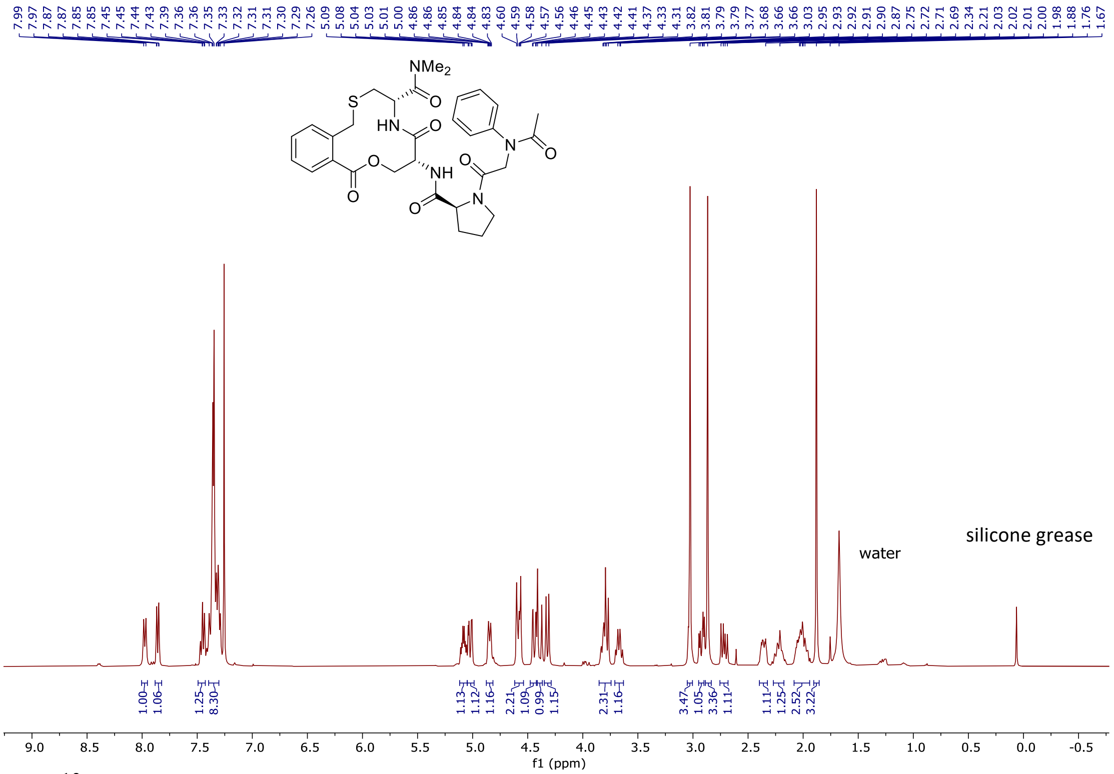

S11 ^{13}C NMR (151 MHz, $CDCl_3$)

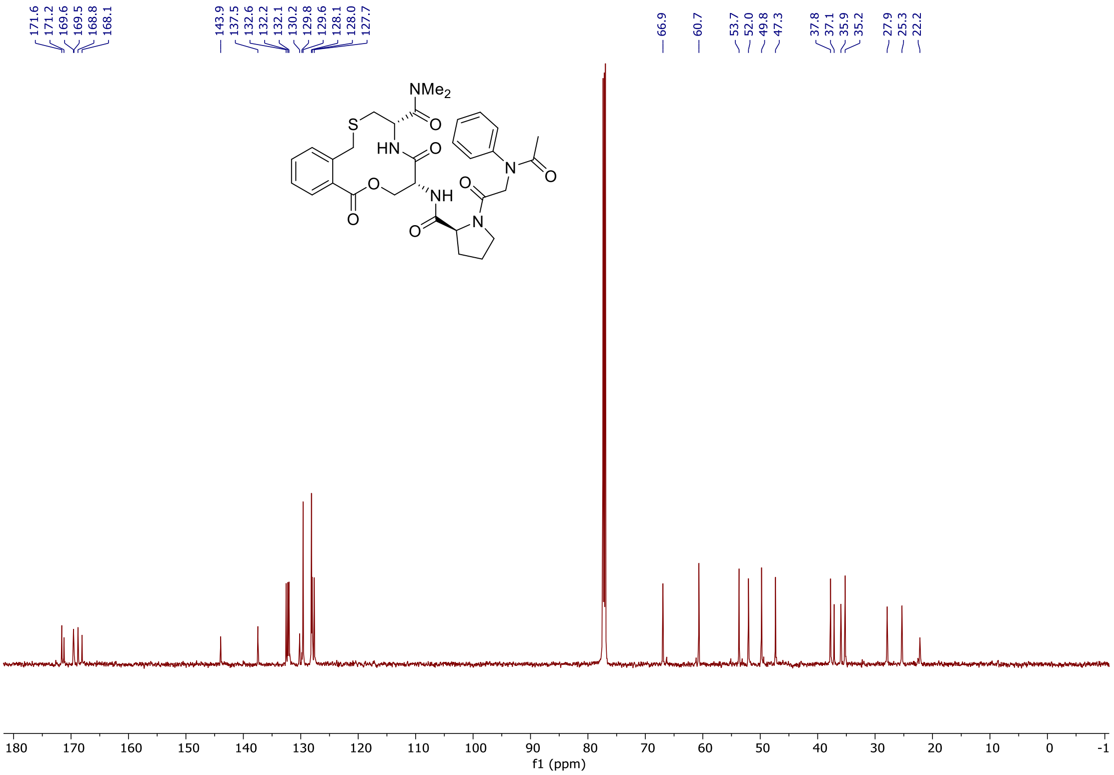

S12 ^{1}H NMR (600 MHz, $CDCl_3$)

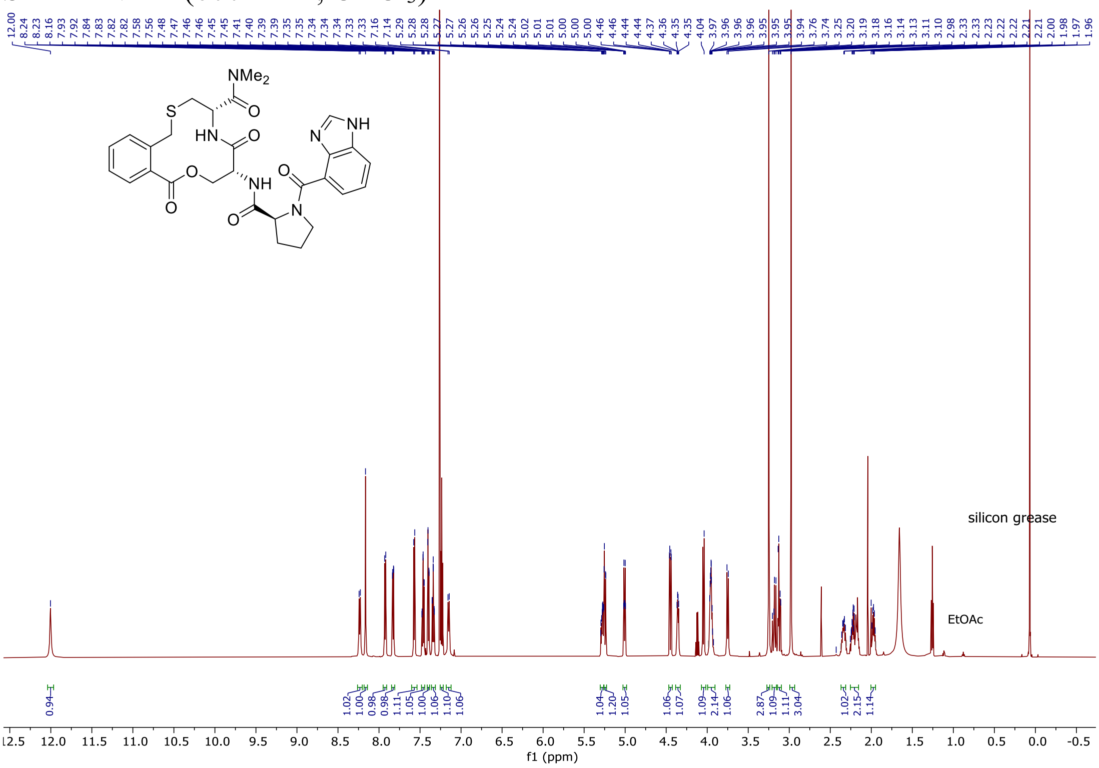

S12 ^{13}C NMR (151 MHz, $CDCl_3$)

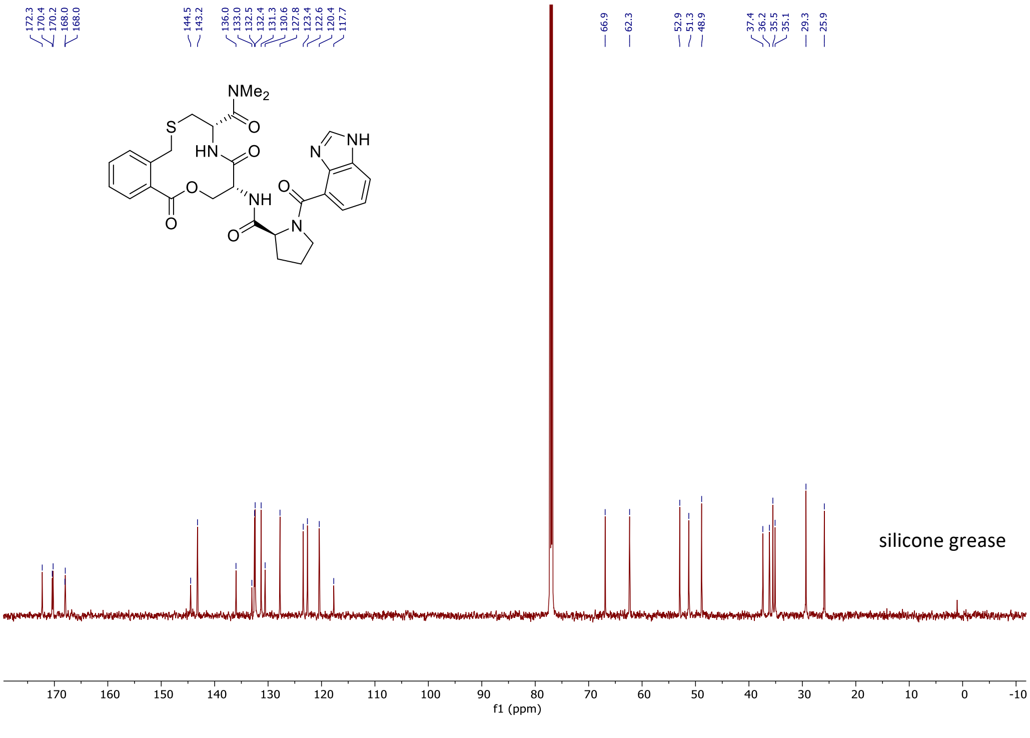

S13 ^{1}H NMR (600 MHz, $CDCl_3$)

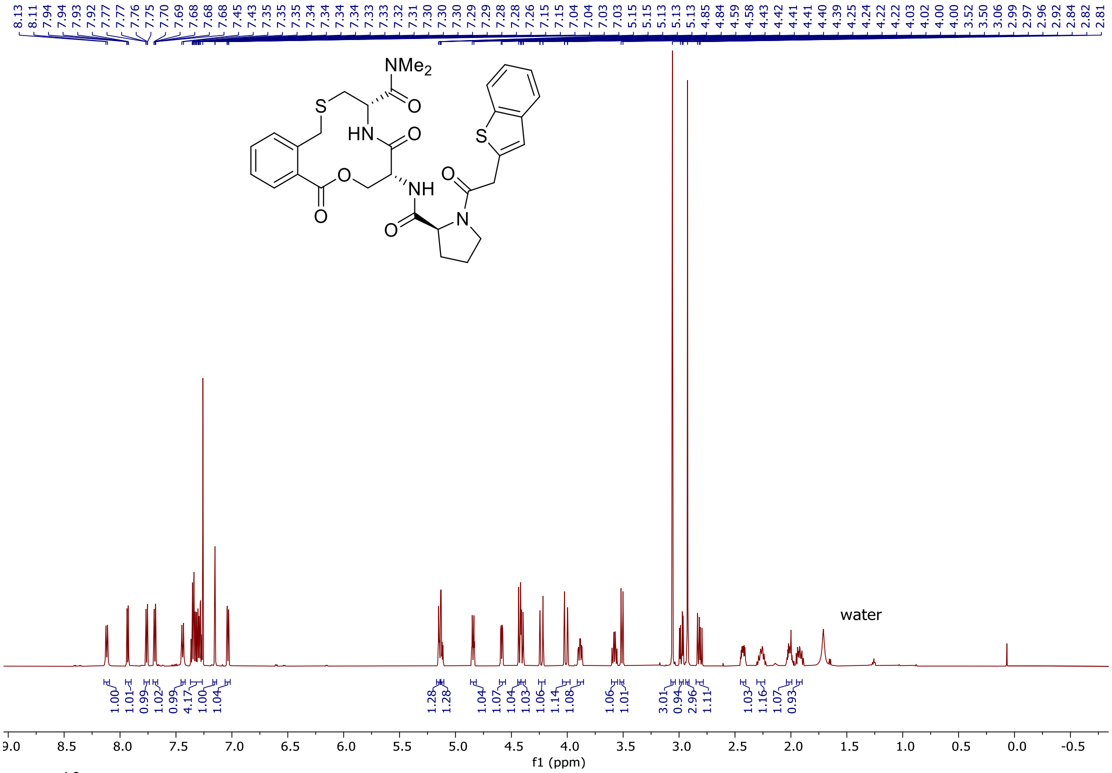

S13 ^{13}C NMR (151 MHz, $CDCl_3$)

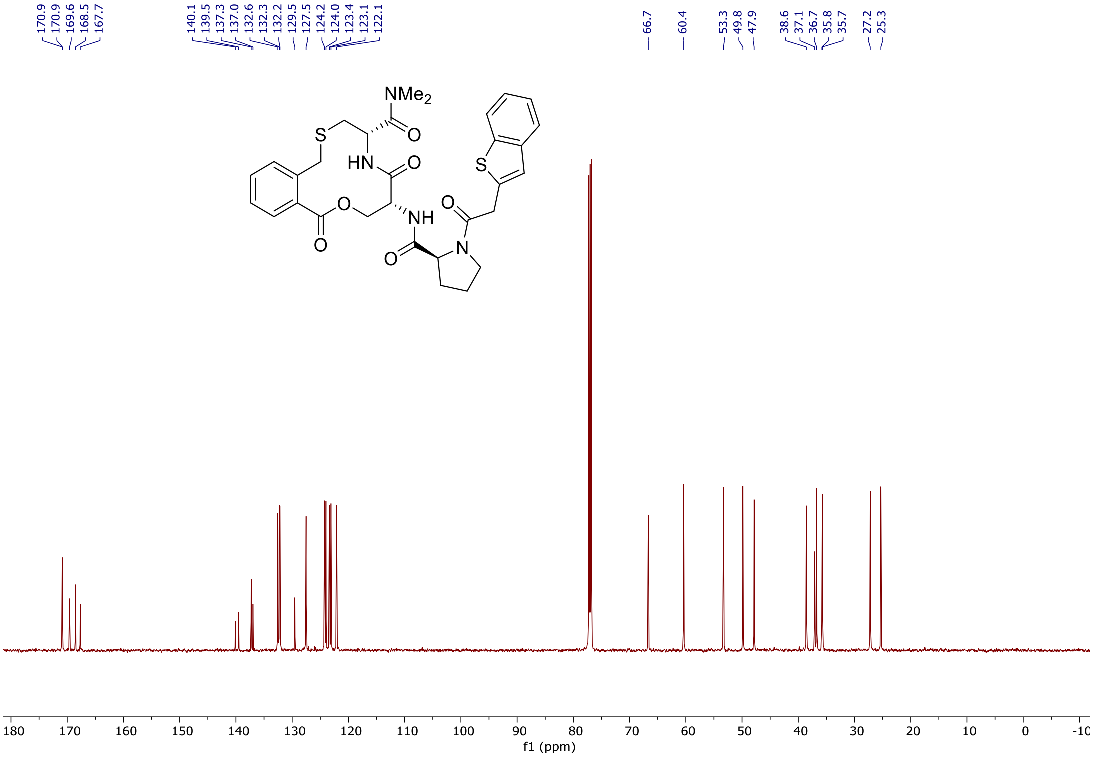

S14 ^{1}H NMR (600 MHz, $CDCl_3$)

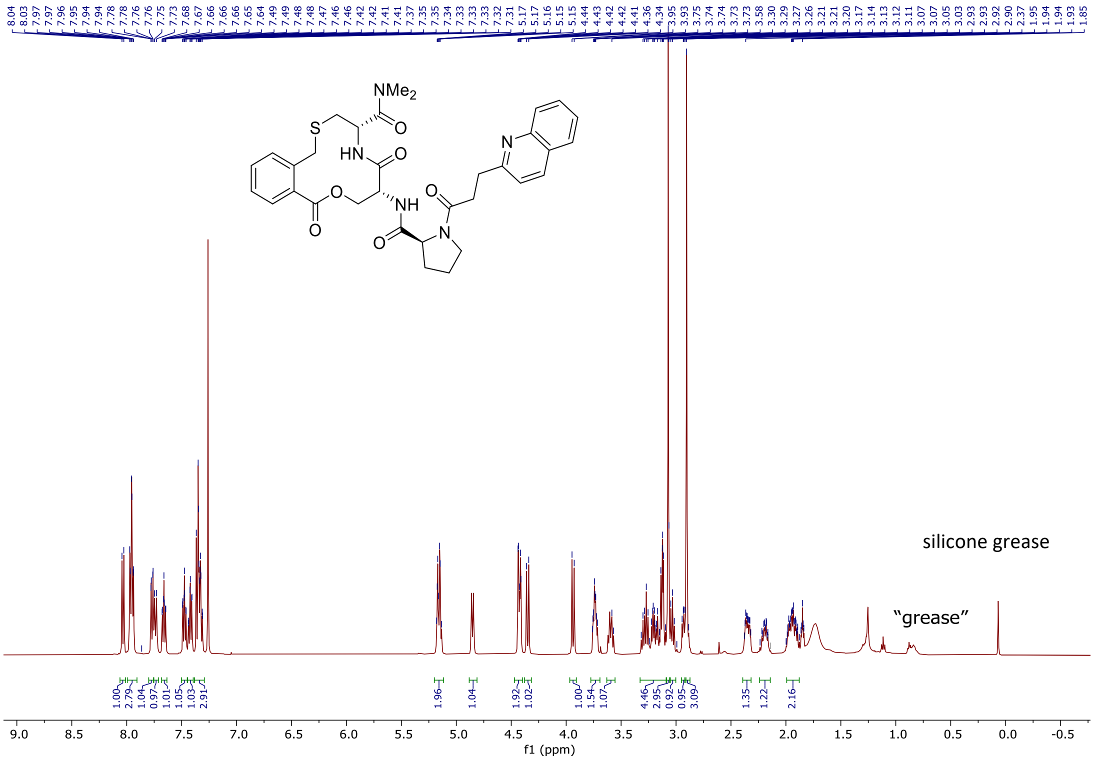

S14 ^{13}C NMR (151 MHz, $CDCl_3$)

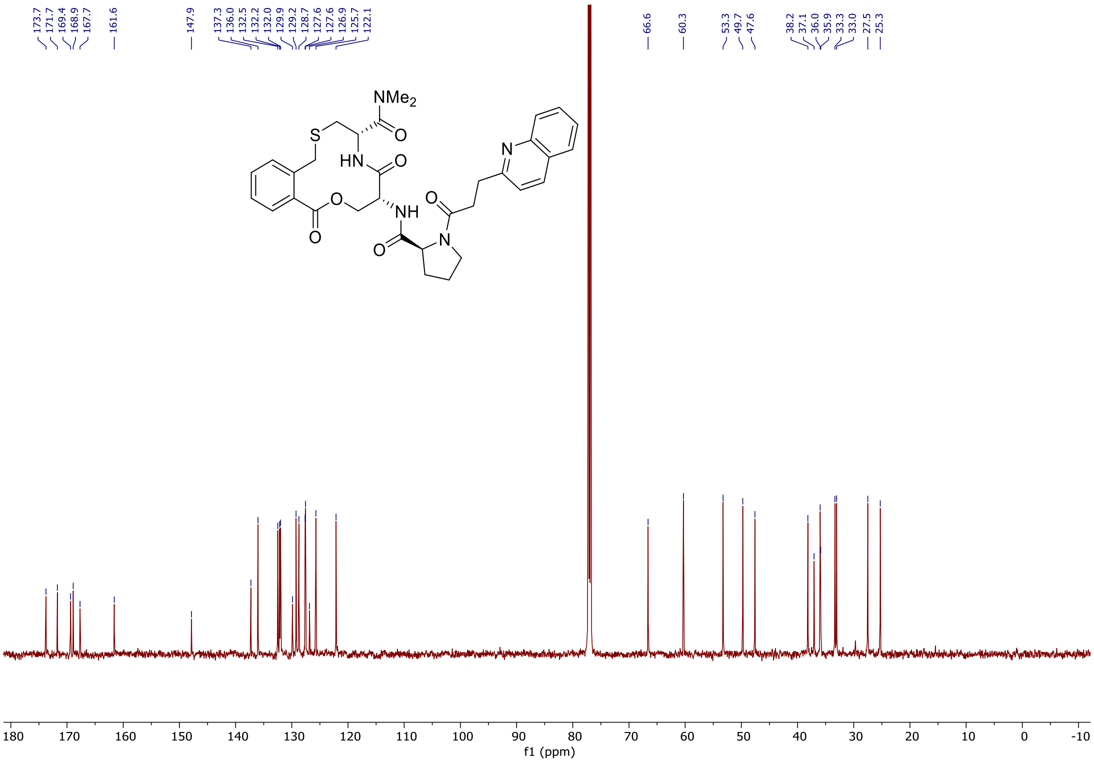

S15 ^{1}H NMR (600 MHz, $CDCl_3$)

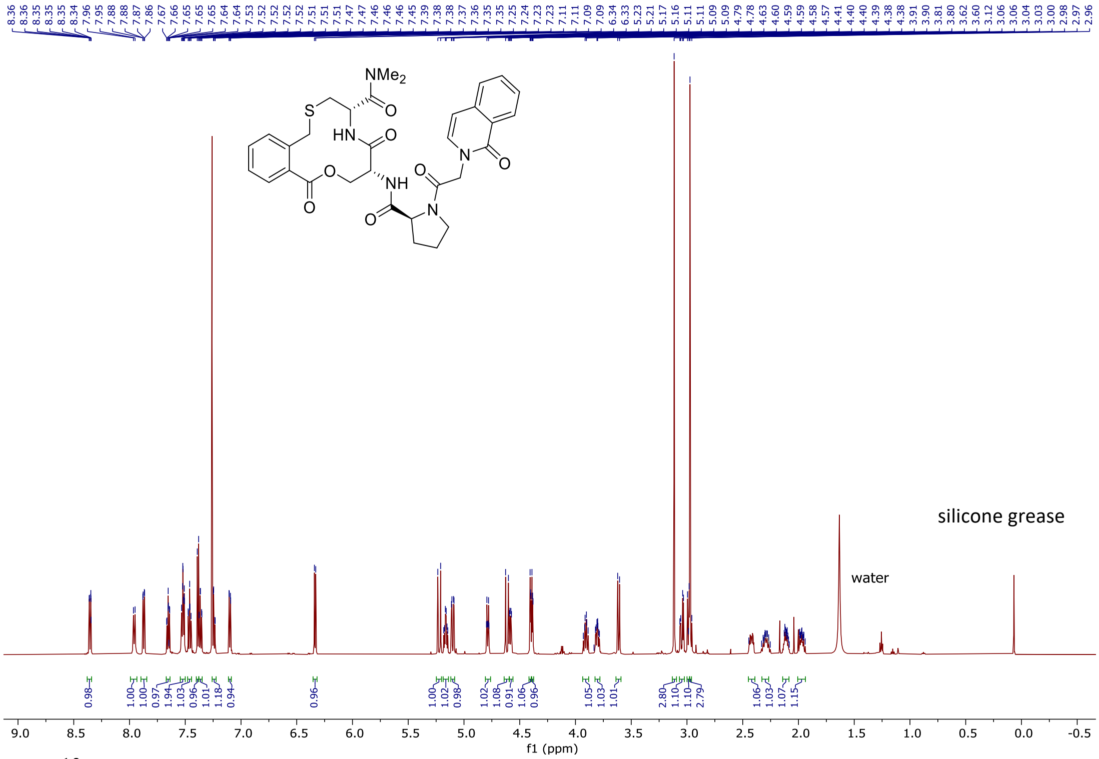

S15 ^{13}C NMR (151 MHz, $CDCl_3$)

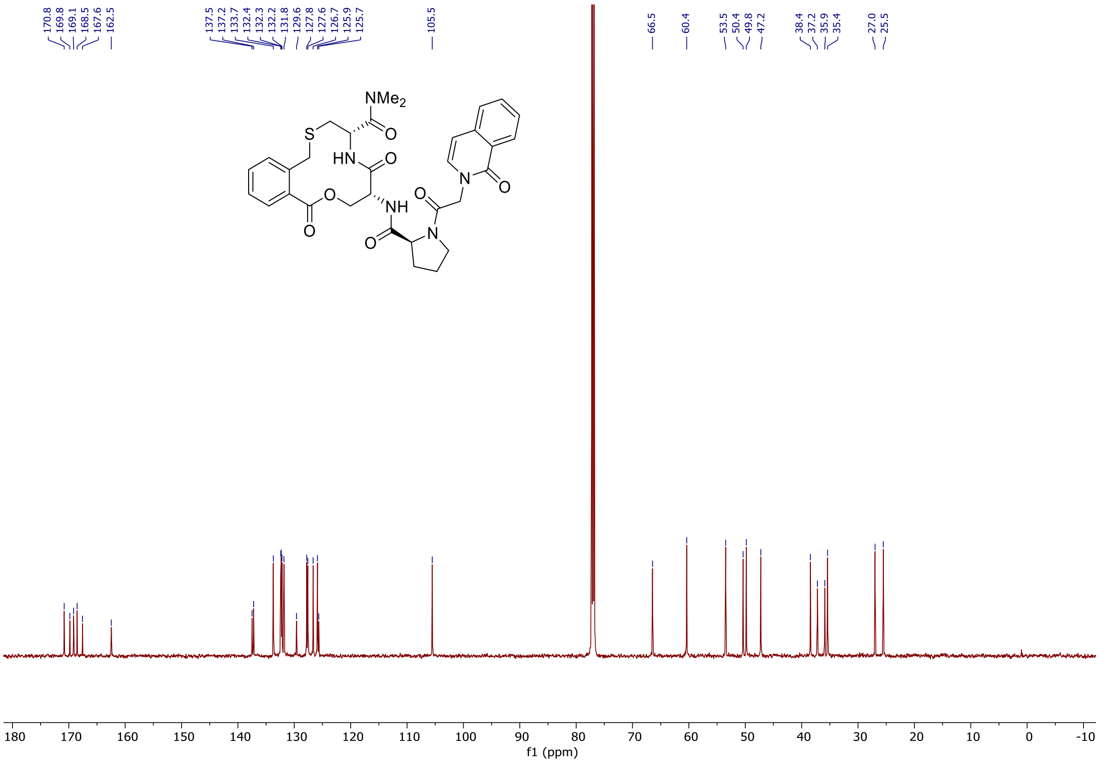

S16 ^{1}H NMR (600 MHz, $CDCl_3$)

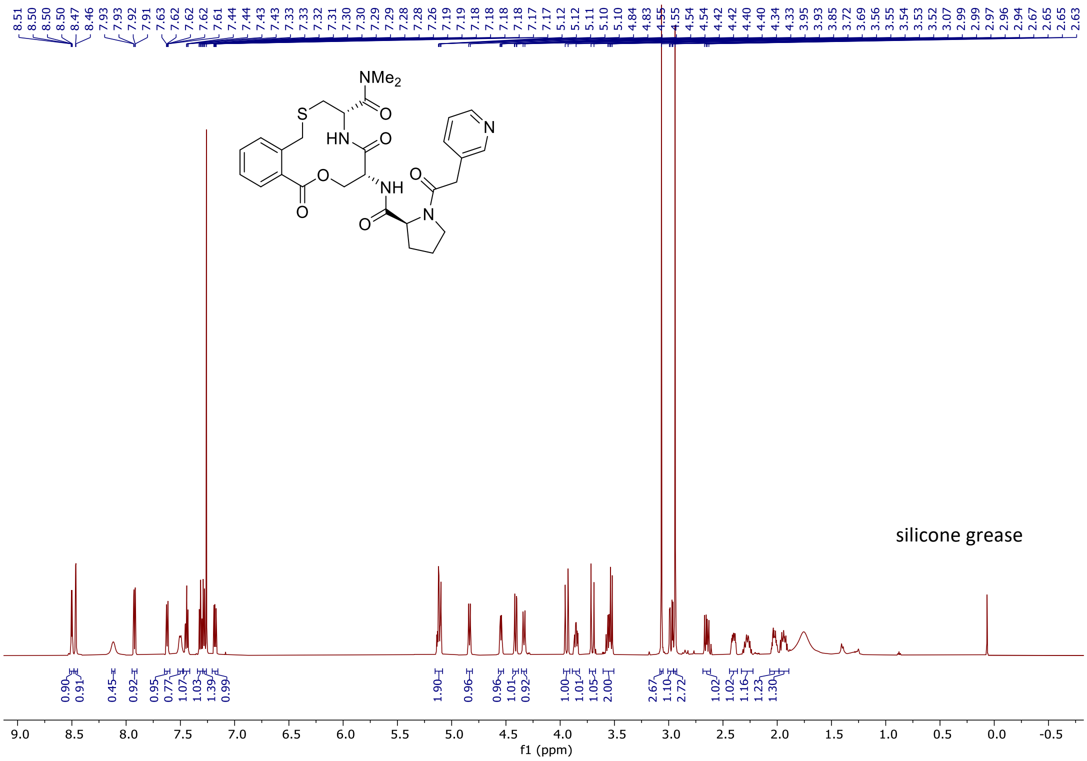

S16 ^{13}C NMR (151 MHz, $CDCl_3$)

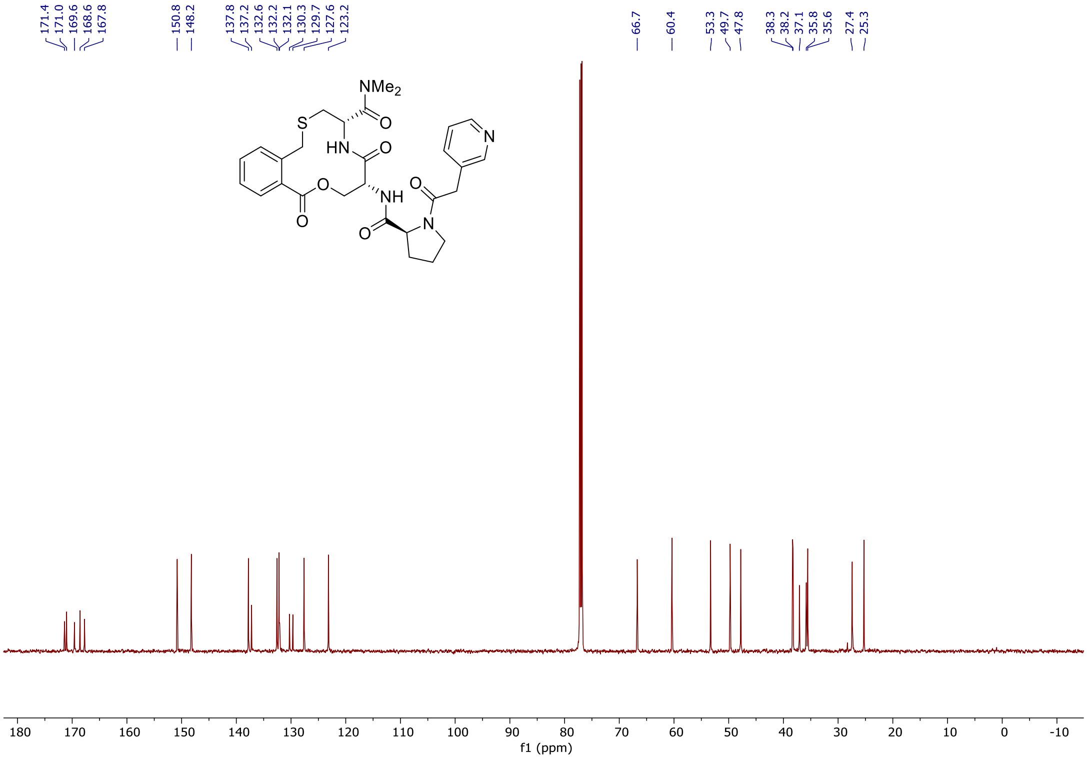

S17 ^{1}H NMR (500 MHz, $CDCl_3$)

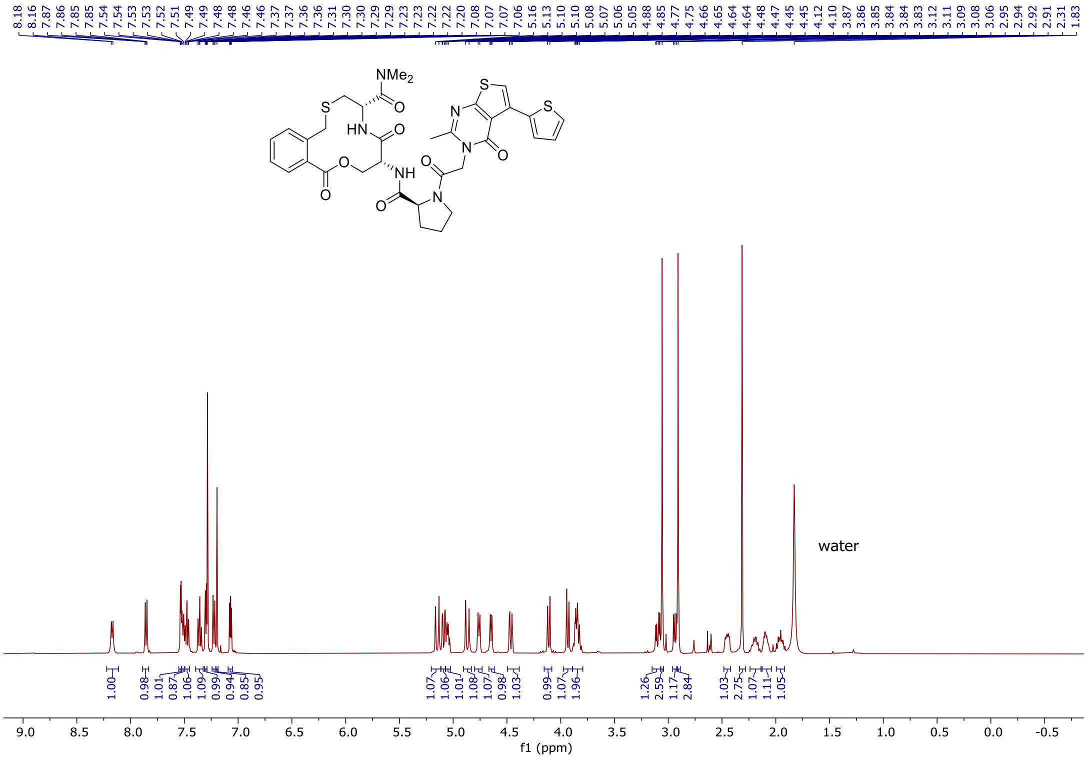

S17 ^{13}C NMR (126 MHz, $CDCl_3$)

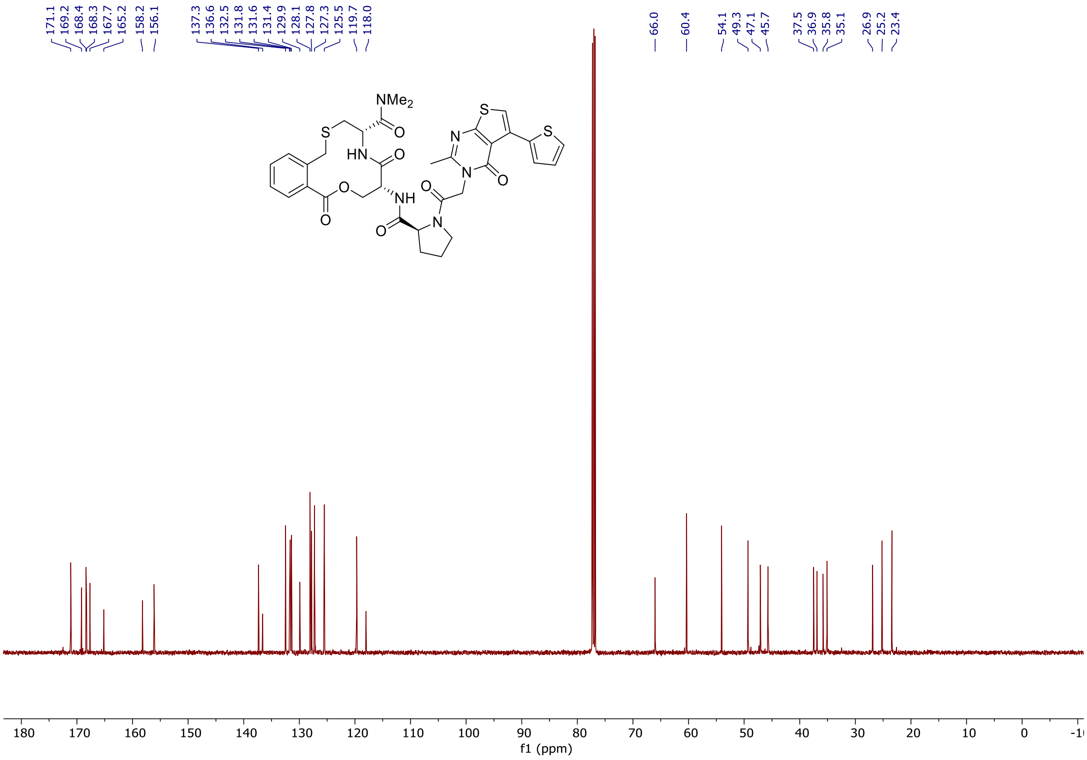

S18 ^{1}H NMR (500 MHz, $CDCl_3$)

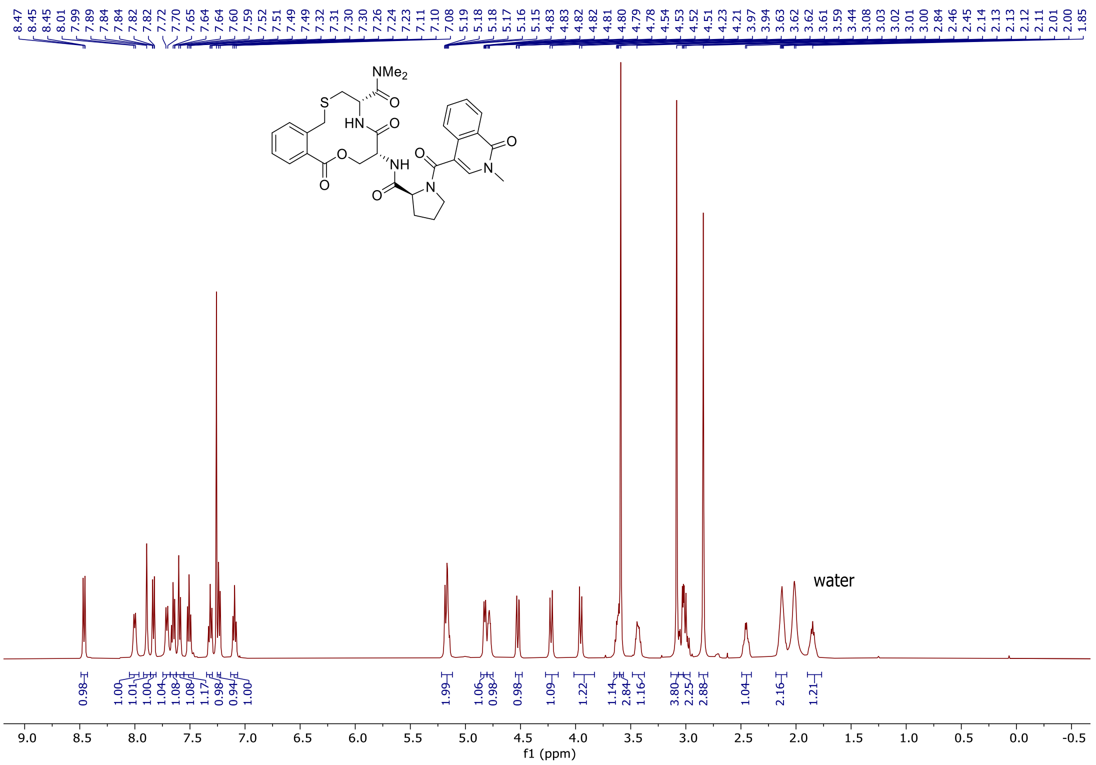

S18 ^{13}C NMR (126 MHz, $CDCl_3$)

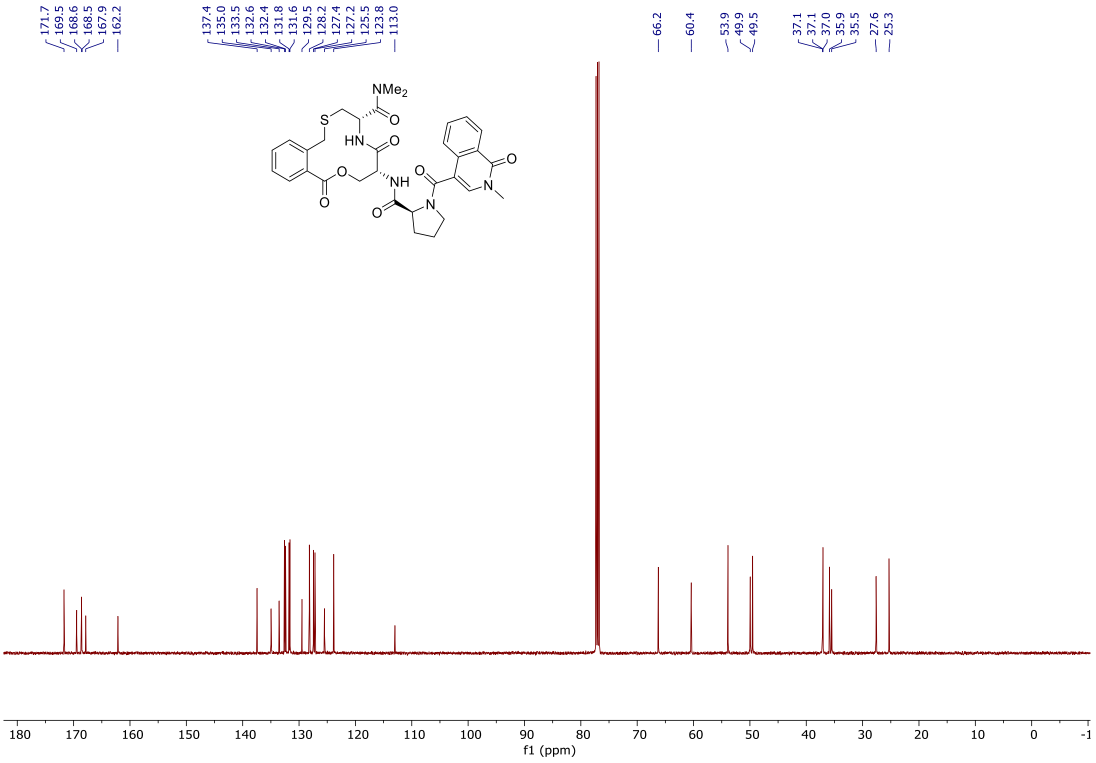

S19 ^{1}H NMR (500 MHz, $CDCl_3$)

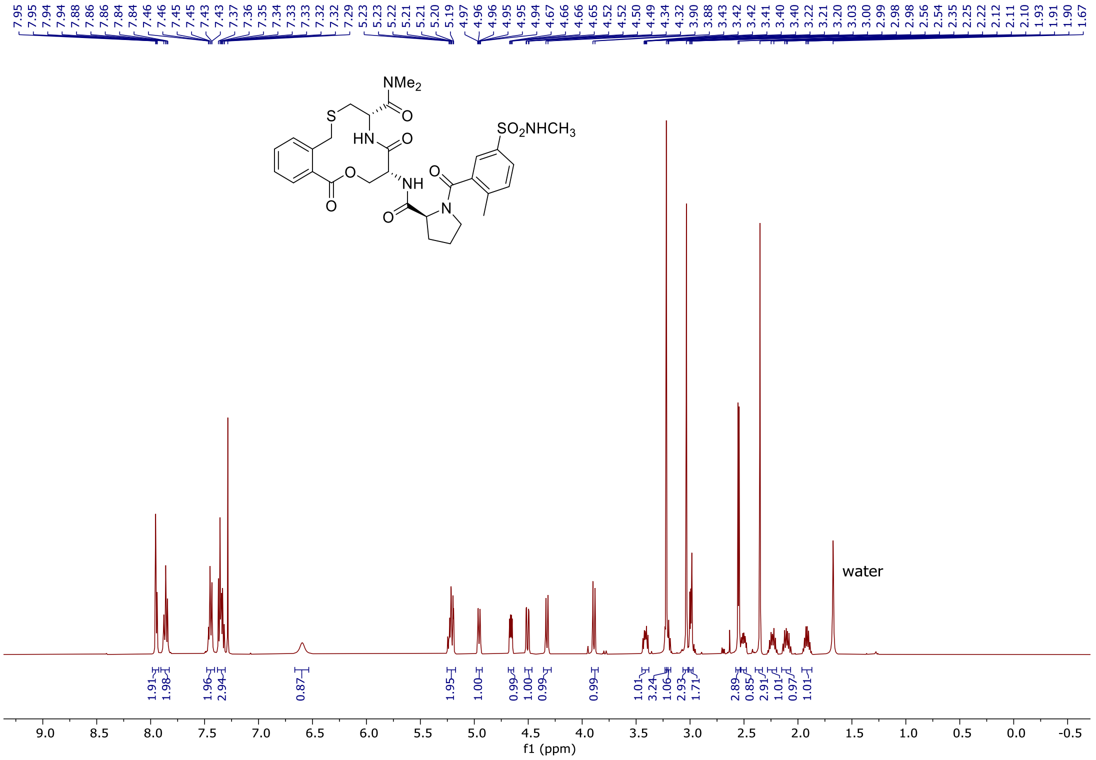

S19 ^{13}C NMR (126 MHz, $CDCl_3$)

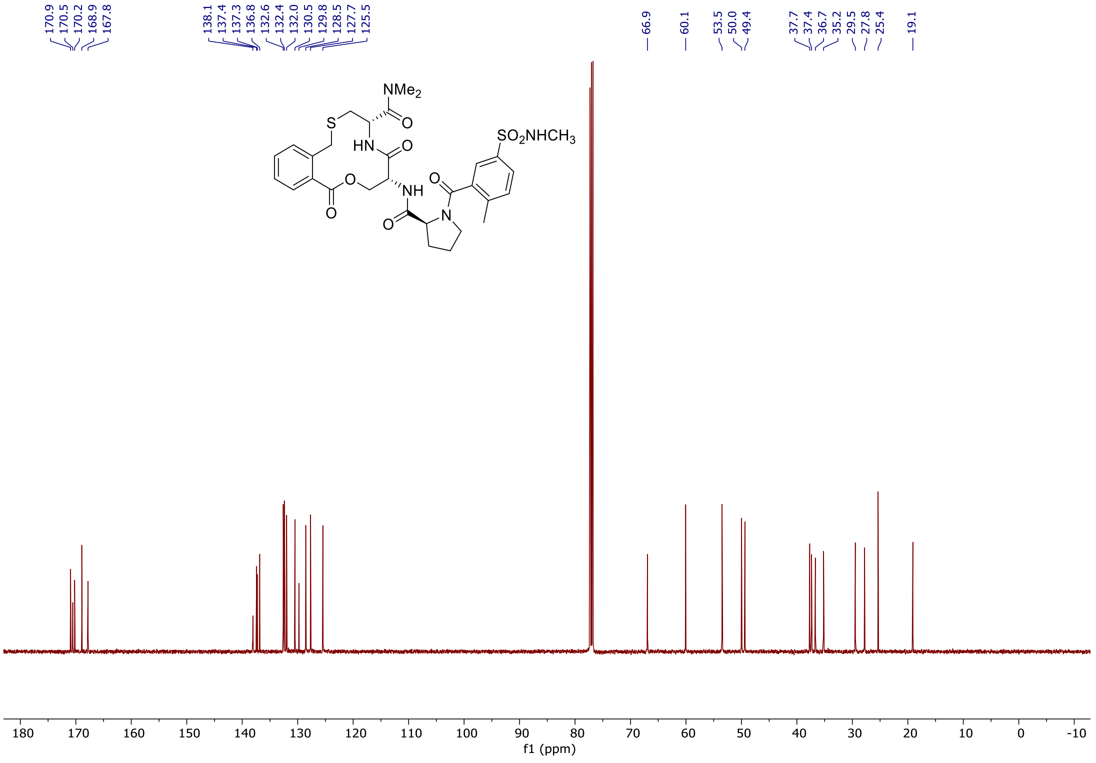

S20 ¹H NMR (500 MHz, $CDCl_3$)

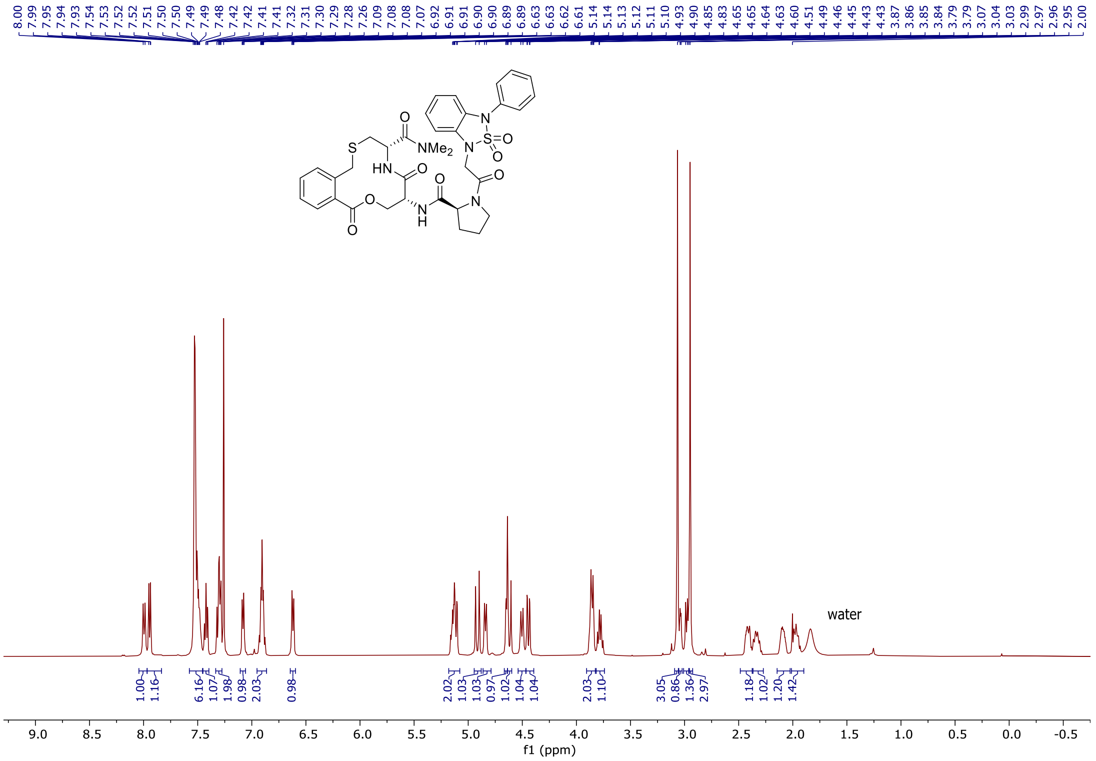

S20 ¹³C NMR (126 MHz, $CDCl_3$)

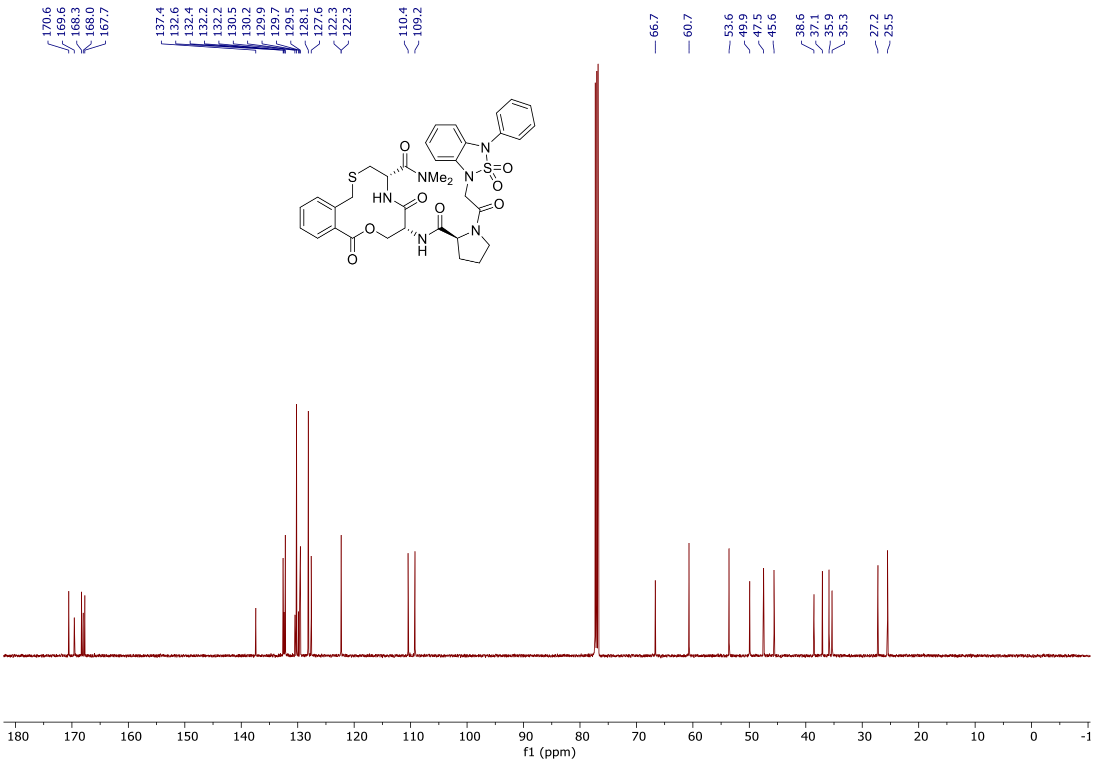

S21 ^{1}H NMR (500 MHz, $CDCl_3$)

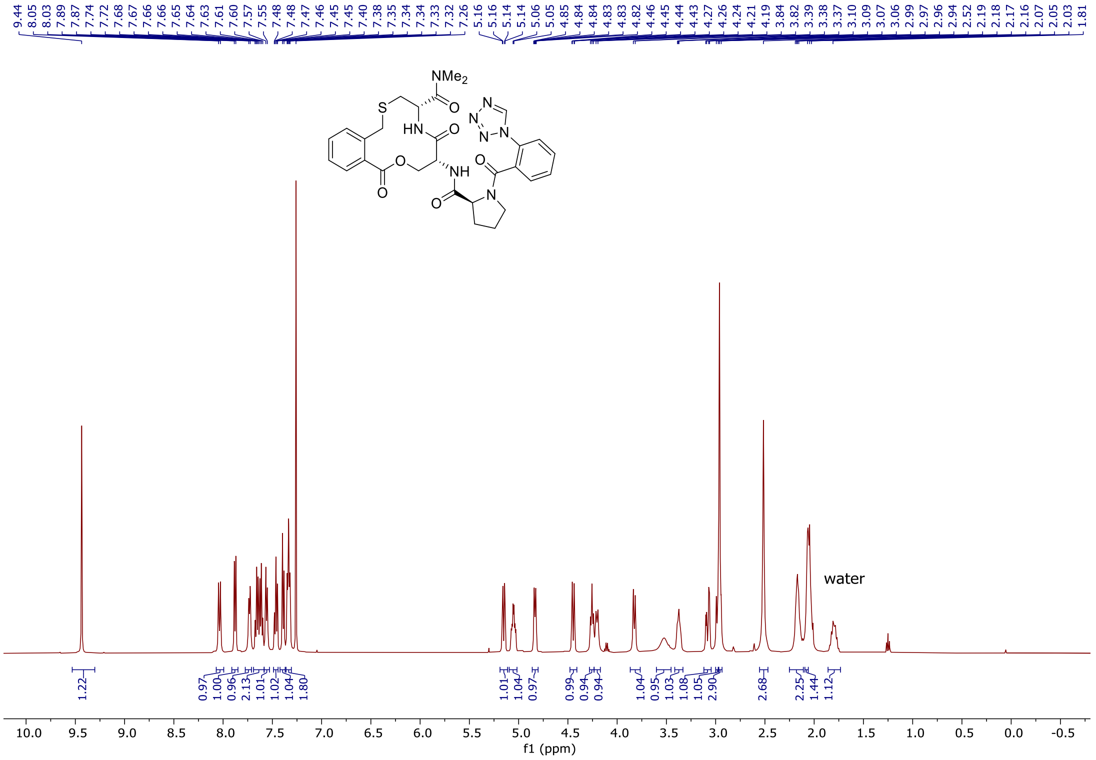

S21 ^{13}C NMR (126 MHz, $CDCl_3$)

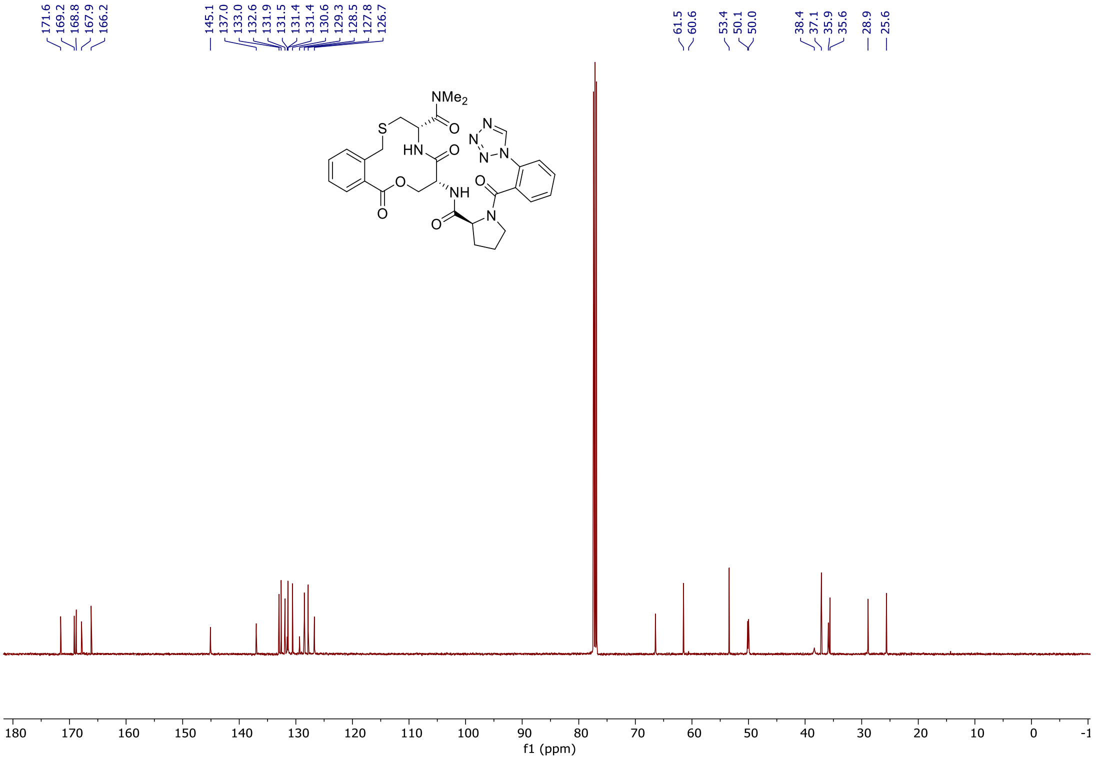

S22 ^{1}H NMR (500 MHz, $CDCl_3$)

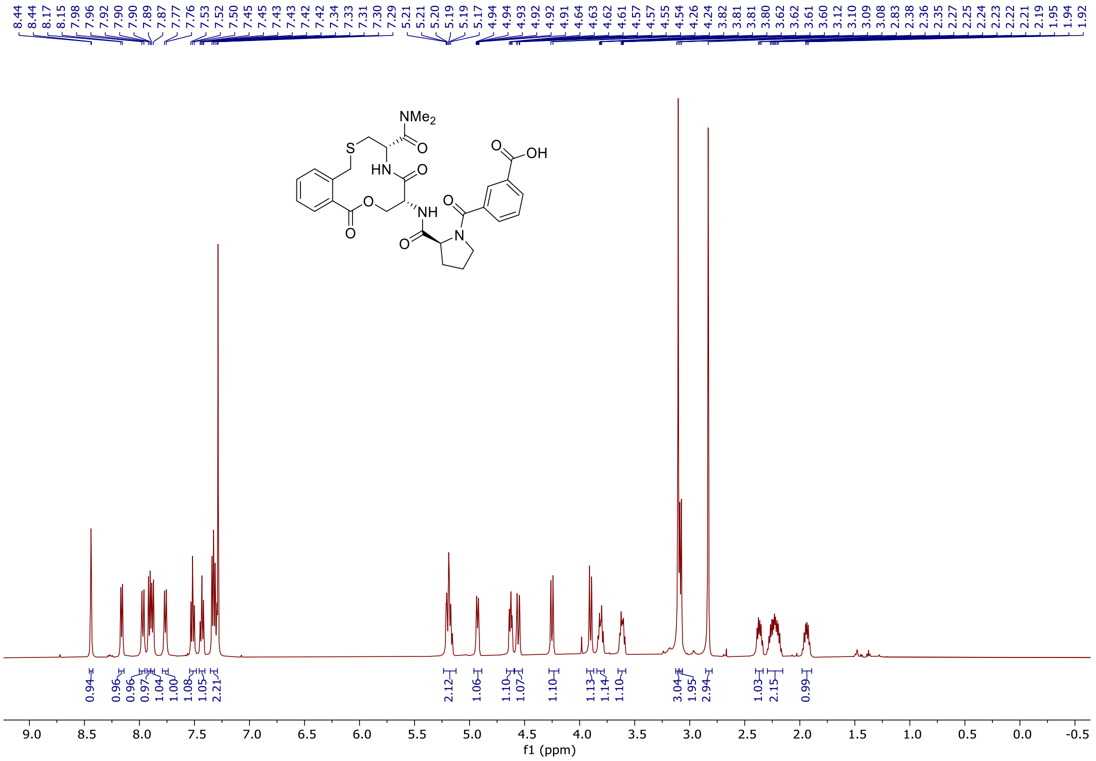

S22 ^{13}C NMR (126 MHz, $CDCl_3$)

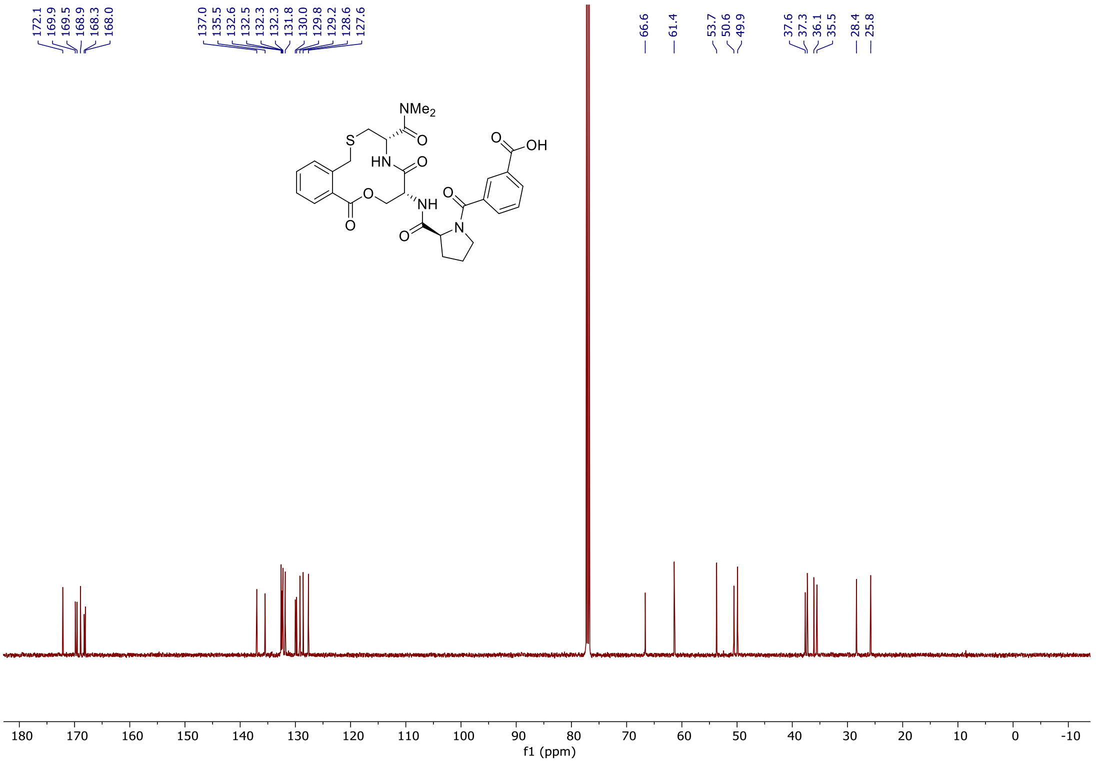

S23 ^{1}H NMR (500 MHz, $CDCl_3$)

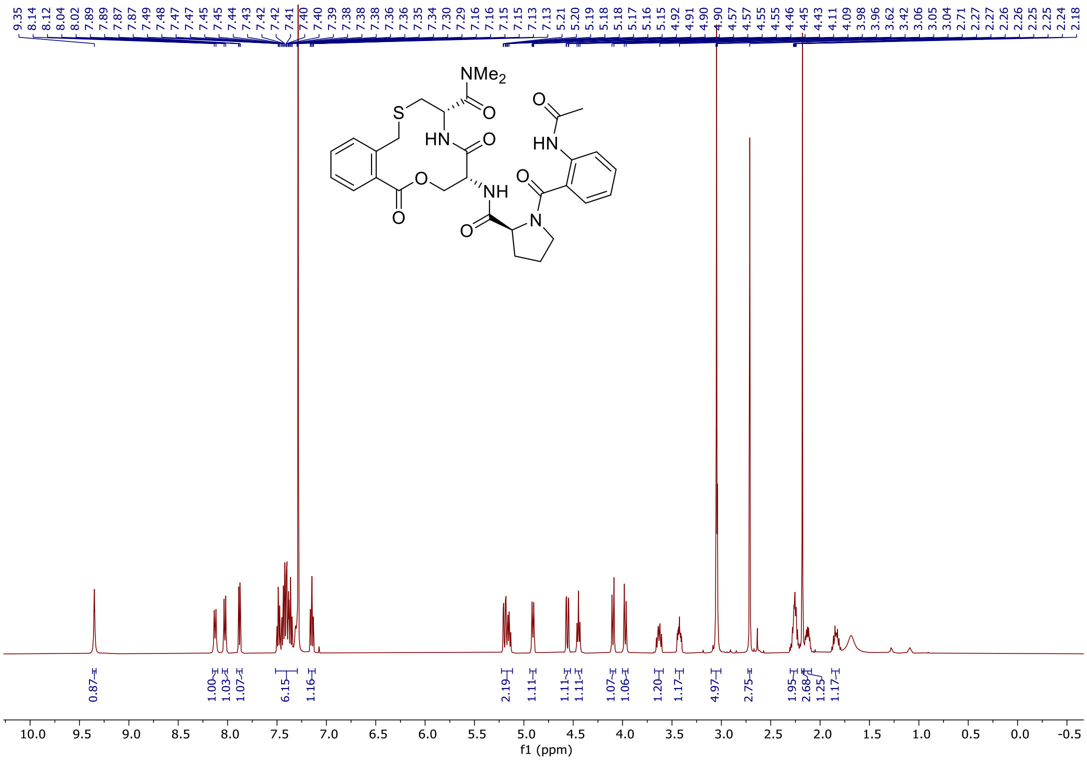

S23 ^{13}C NMR (126 MHz, $CDCl_3$)

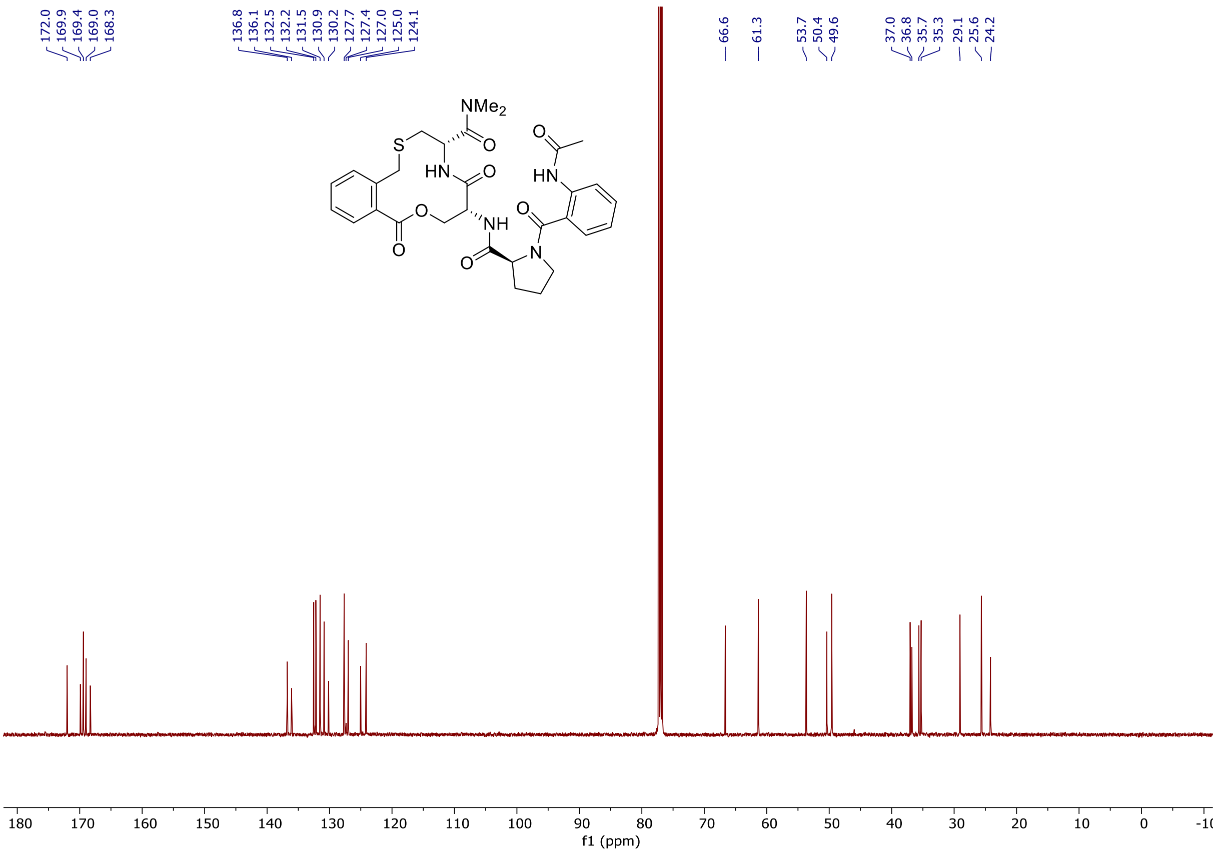

S24 ^{1}H NMR (500 MHz, $CDCl_3$)

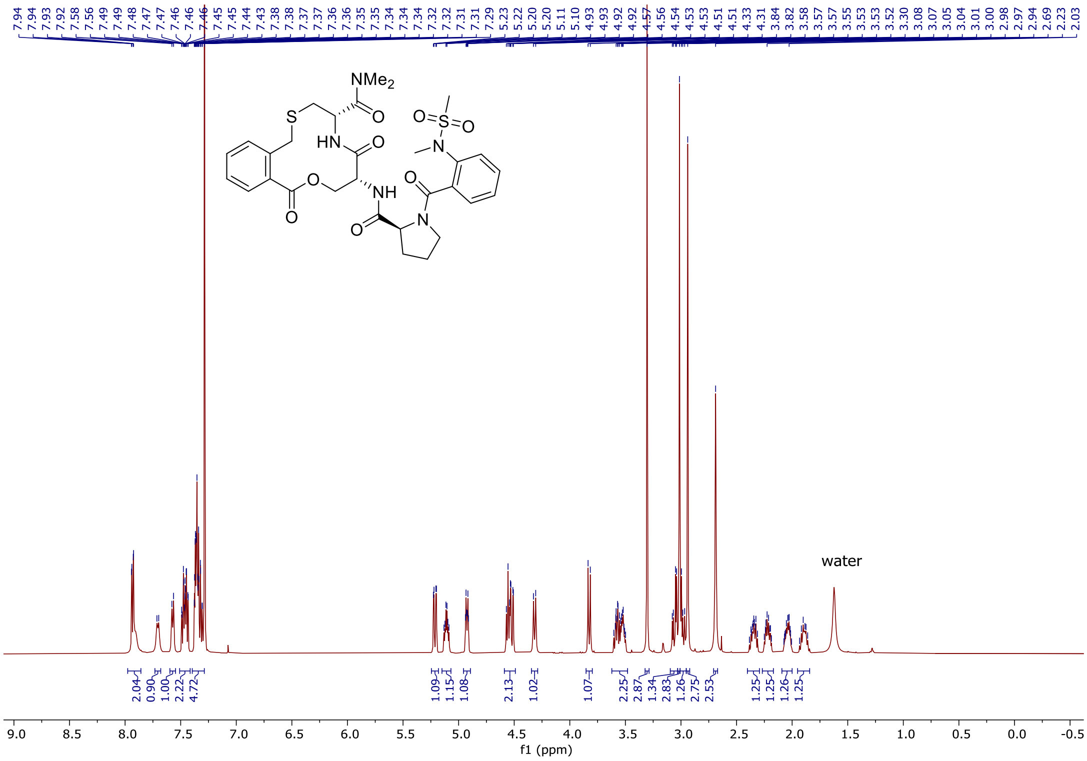

S24 ^{13}C NMR (126 MHz, $CDCl_3$)

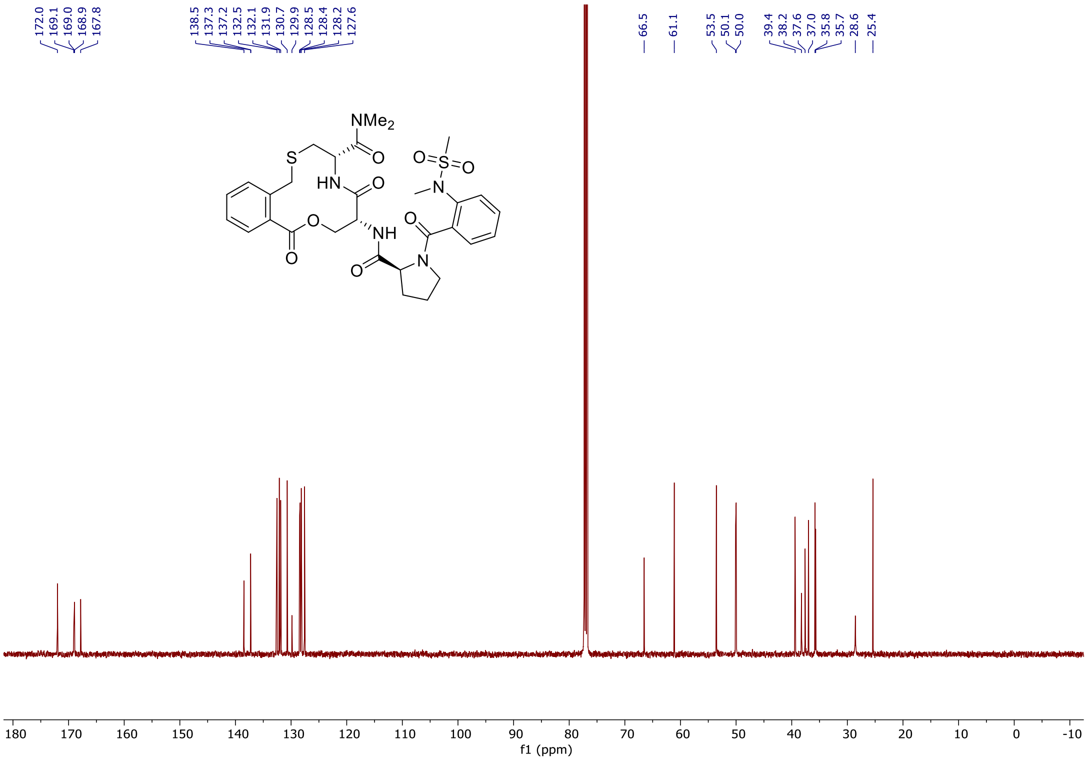

Purity reports for compounds S1-S24

Compound **S1**

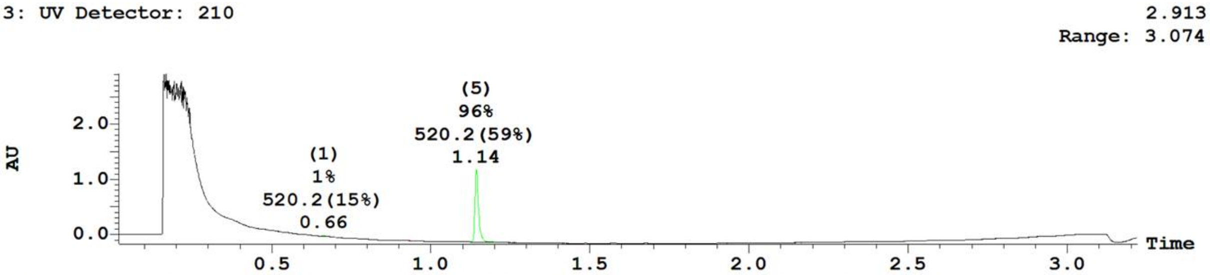

Peak Number	Compound	Time	AreaAbs	Area %Total	Width	Height	Mass Found
1	Found	0.66	2e+002	1.35	0	2e+004	520.20
3		0.90	9e+001	0.48	0	8e+003	
4		0.93	2e+001	0.13	0	3e+003	
5	Found	1.14	2e+004	96.32	0	1e+006	520.20
6	Found	1.18	3e+002	1.69	0	2e+004	520.20
8		2.35	7e+000	0.04	0	2e+003	

Compound **S2**

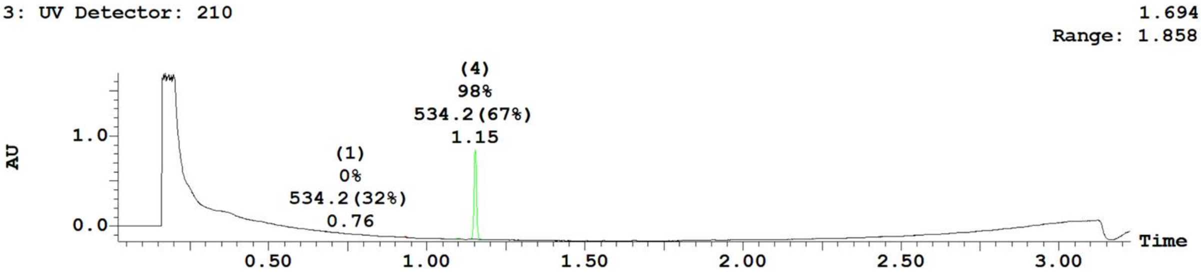

Compound **S3**

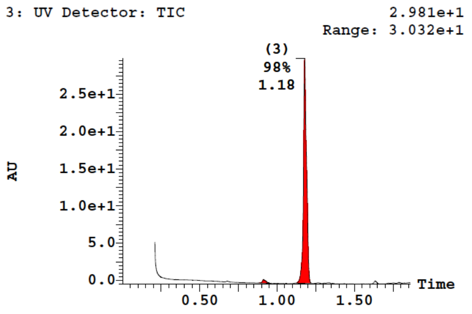

Peak ID	Time	Mass Found
3	1.18	

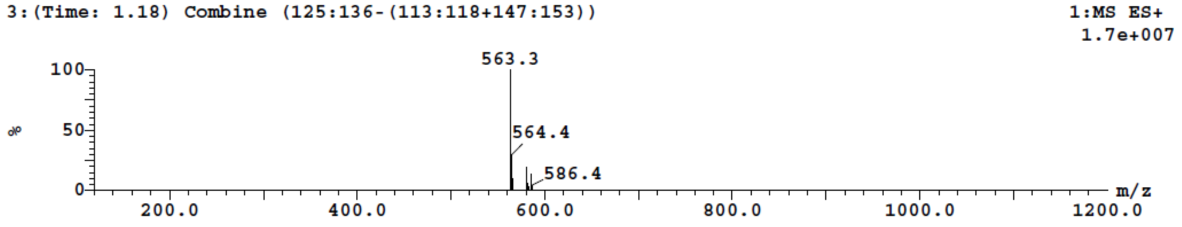

Compound **S4**

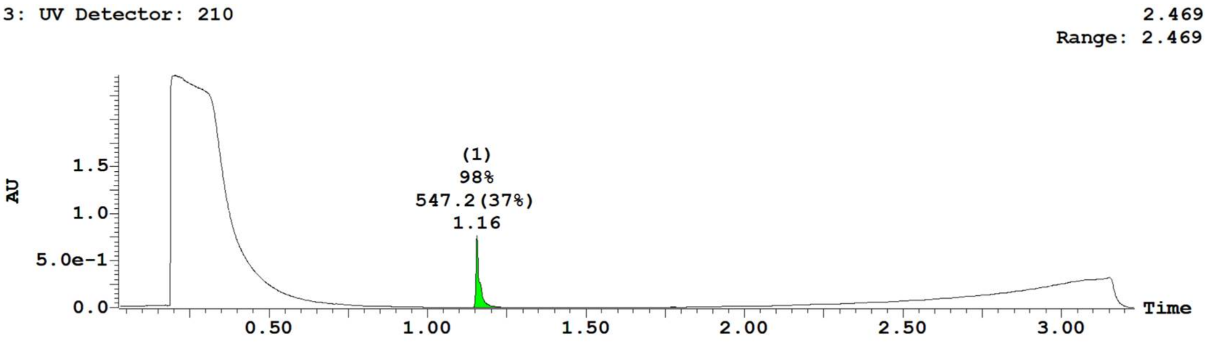

Compound **S5**

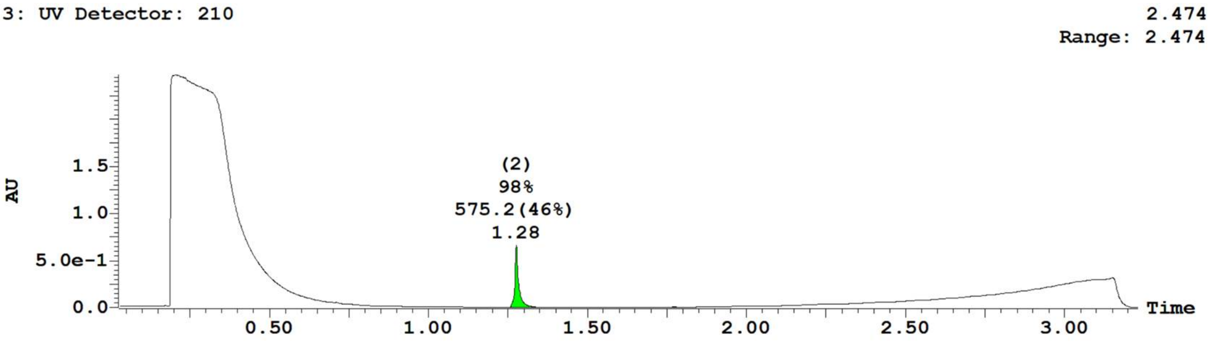

Compound **S6**

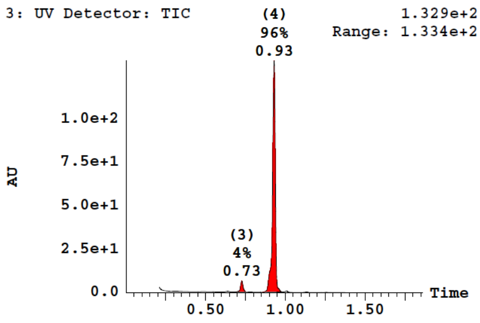

Peak ID	Time	Mass Found
4	0.93	

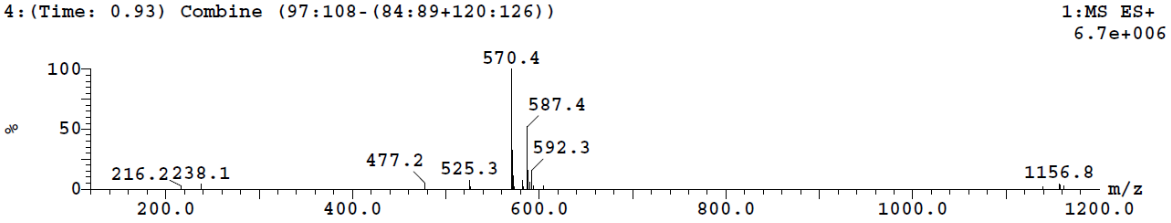

Compound **S7**

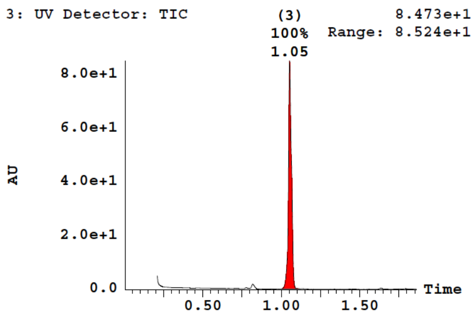

Peak ID	Time	Mass Found
3	1.06	

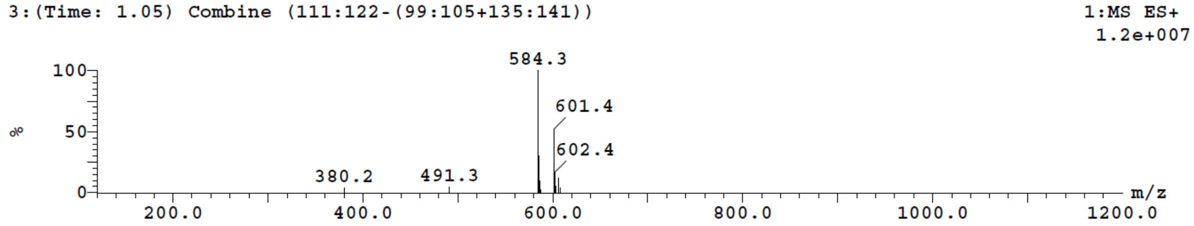

Compound **S8**

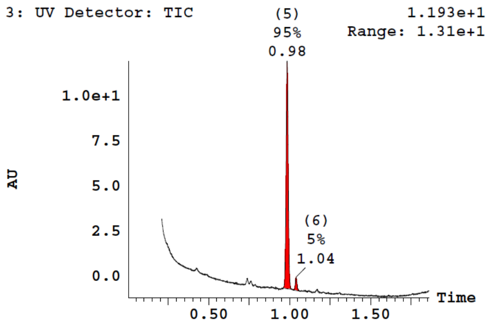

Peak ID	Time	Mass Found
5	0.99	

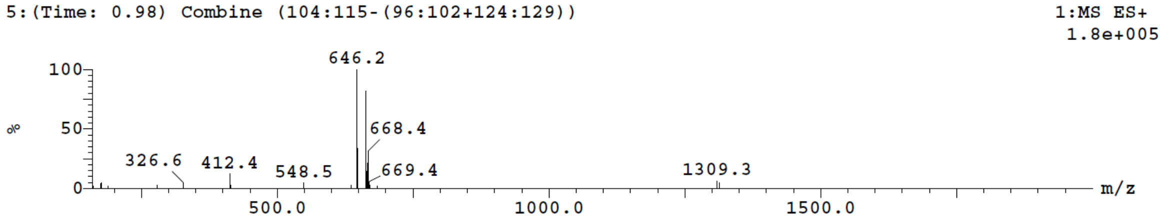

Compound **S9**

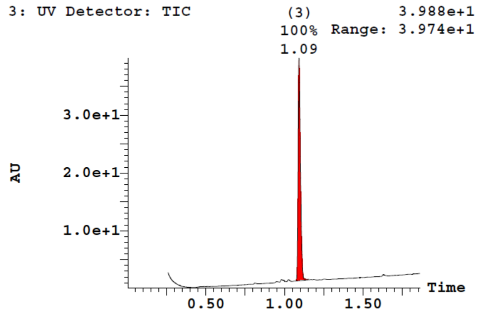

Peak ID	Time	Mass Found
3	1.09	

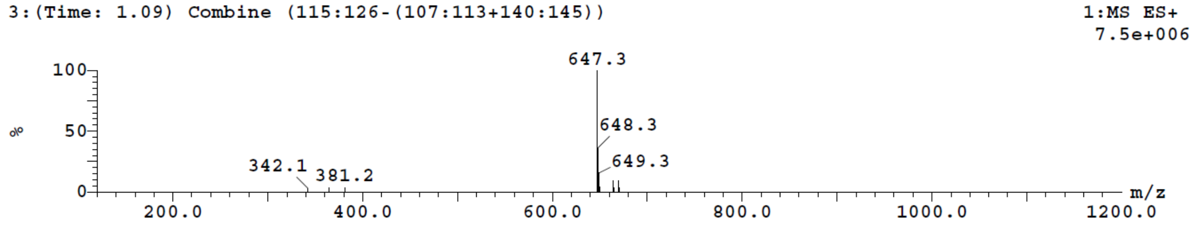

Compound **S10**

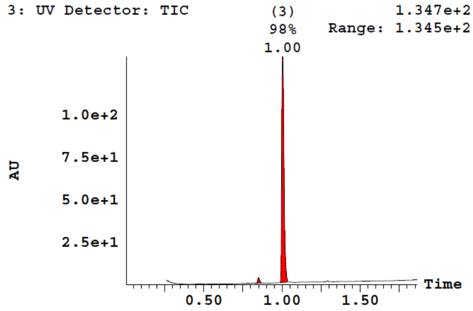

Peak ID	Time	Mass Found
3	1.00	

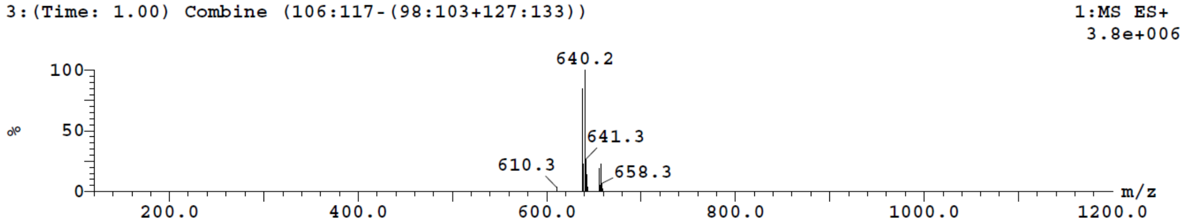

Compound **S11**

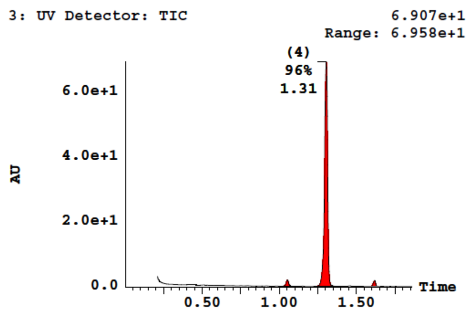

Peak ID	Time	Mass Found
4	1.30	

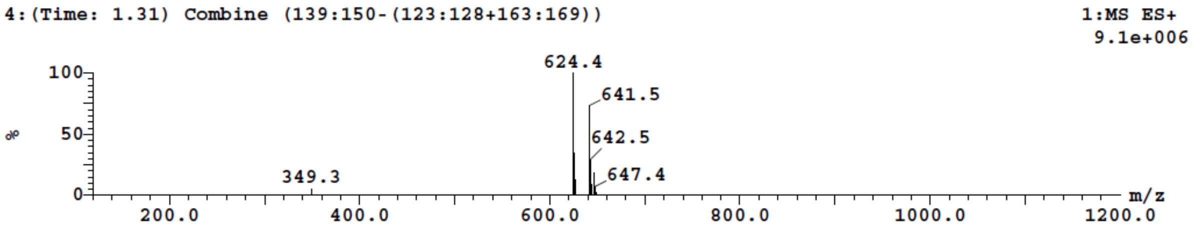

Compound **S12**

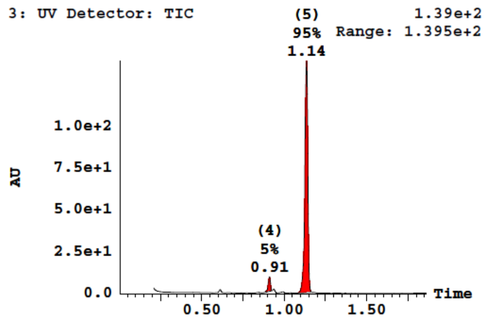

Peak ID	Time	Mass Found
5	1.15	

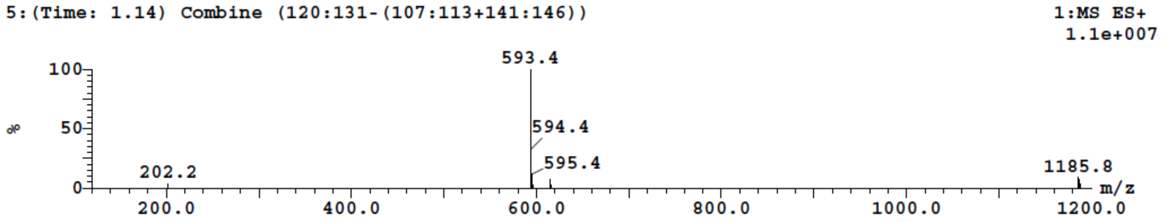

Compound **S13**

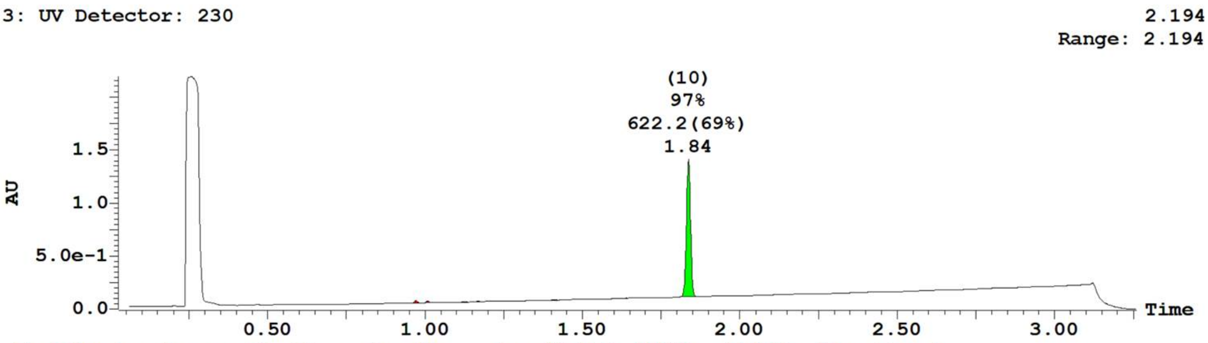

Peak Number	Compound	Time	AreaAbs	Area %Total	Width	Height	Mass Found
1		0.97	3e+002	1.25	0	2e+004	
2		1.01	2e+002	1.02	0	2e+004	
3		1.14	4e+001	0.20	0	3e+003	
4		1.17	3e+001	0.14	0	2e+003	
5		1.42	2e+001	0.12	0	2e+003	
6	Tentative	1.55	2e+001	0.10	0	1e+003	622.19
7	Tentative	1.56	2e+001	0.10	0	1e+003	622.19
8		1.64	3e+001	0.15	0	2e+003	
10	Found	1.84	2e+004	96.93	0	1e+006	622.19

Compound **S14**

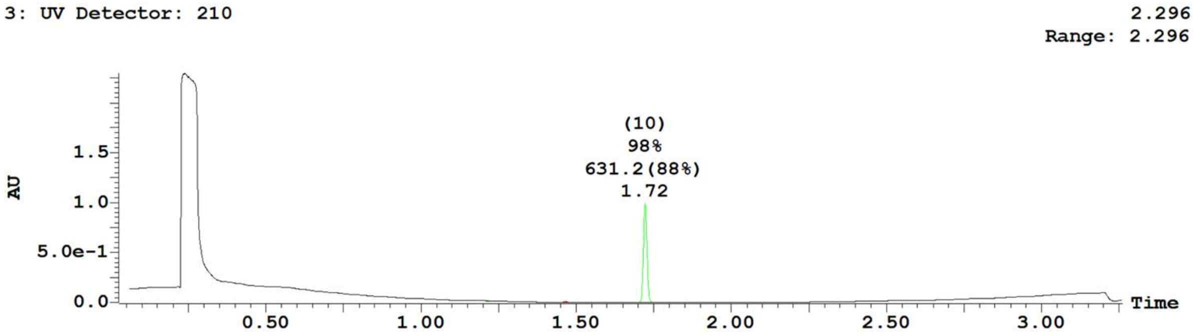

Peak Number	Compound	Time	AreaAbs	Area %Total	Width	Height	Mass Found
2	Tentative	1.18	3e+001	0.24	0	2e+003	631.25
3	Found	1.21	3e+001	0.24	0	3e+003	631.25
4	Found	1.24	8e+000	0.06	0	8e+001	631.25
5		1.47	1e+002	0.82	0	8e+003	
6		1.49	4e+001	0.27	0	2e+003	
7	Tentative	1.54	3e+001	0.25	0	3e+003	631.25
8	Found	1.60	2e+001	0.16	0	1e+003	631.25
9	Found	1.68	2e+001	0.15	0	2e+003	631.25
10	Found	1.72	1e+004	97.69	0	1e+006	631.25
12	Found	1.96	2e+001	0.12	0	1e+003	631.25

Compound **S15**

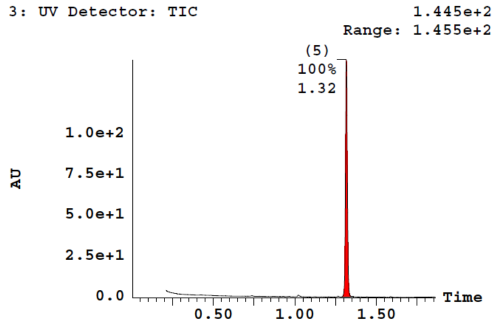

Peak ID	Time	Mass Found
5	1.32	

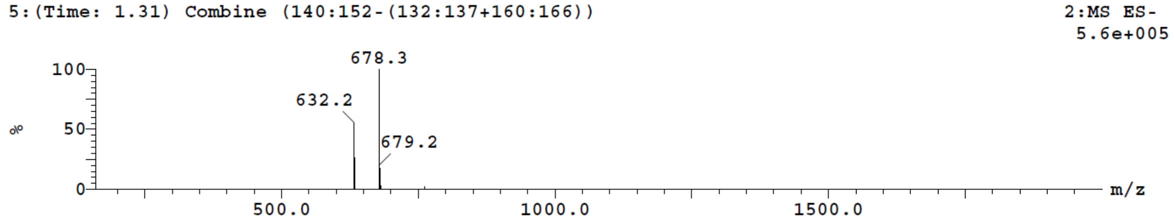

Compound **S16**

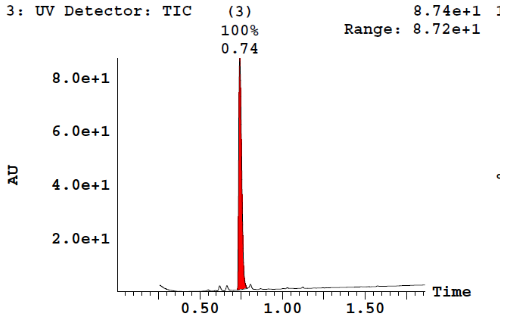

Peak ID	Time	Mass Found
3	0.74	

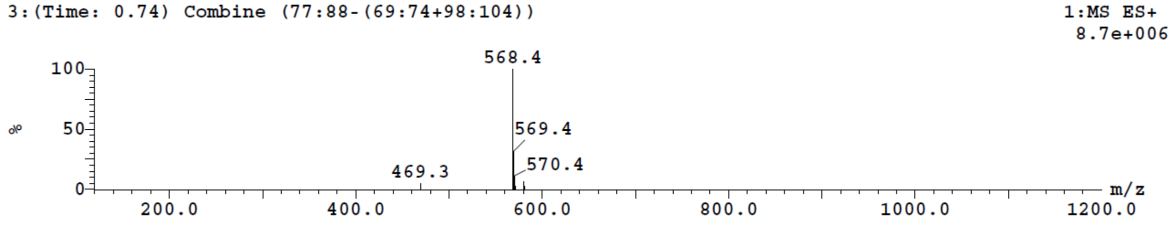

Compound **S17**

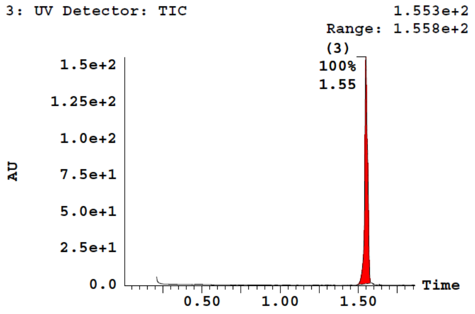

Peak ID	Time	Mass Found
3	1.56	

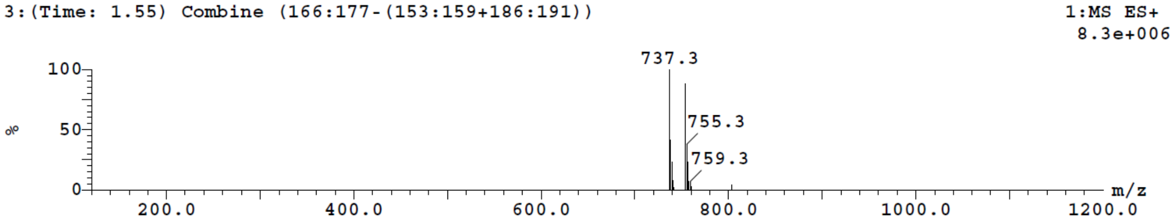

Compound **S18**

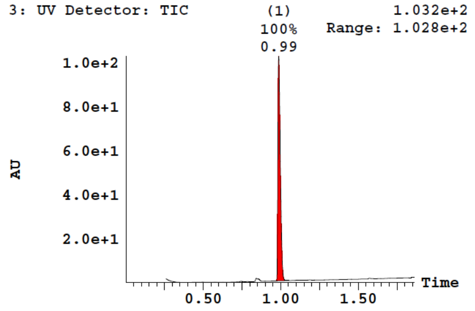

Peak ID	Time	Mass Found
1	0.99	

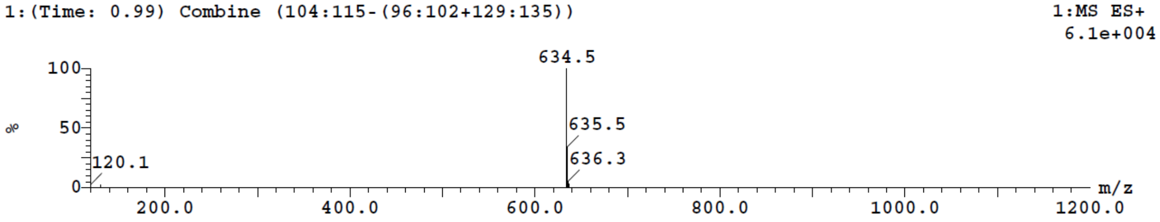

Compound **S19**

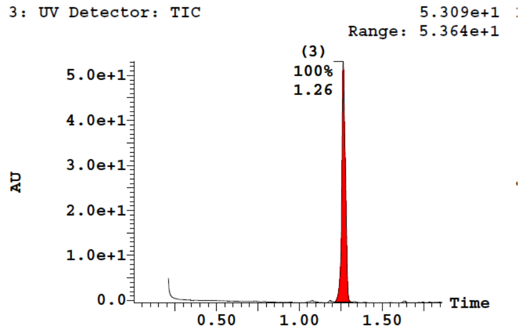

Peak ID	Time	Mass Found
3	1.27	

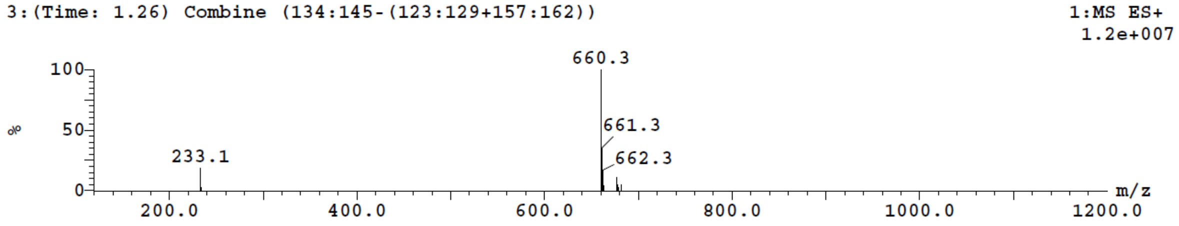

Compound **S20**

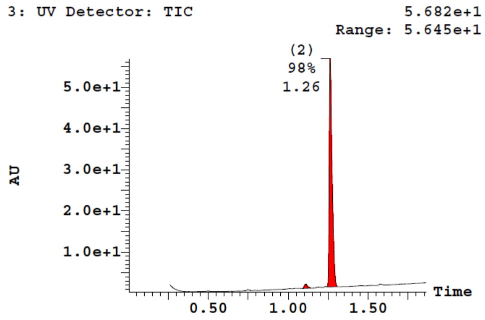

Peak ID	Time	Mass Found
2	1.26	

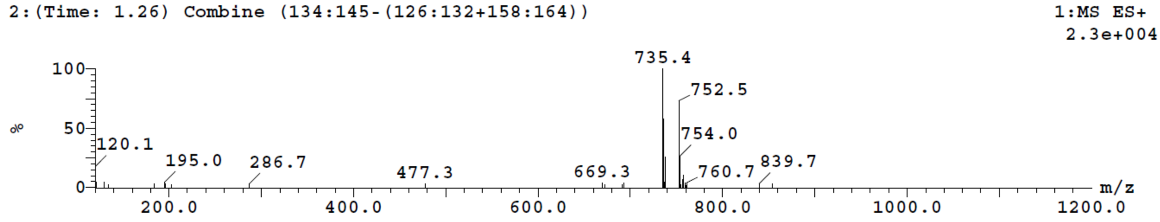

Compound **S21**

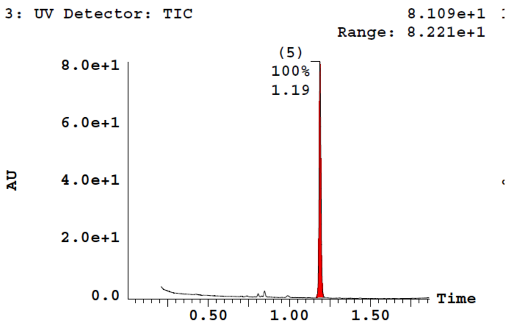

Peak ID	Time	Mass Found
5	1.18	

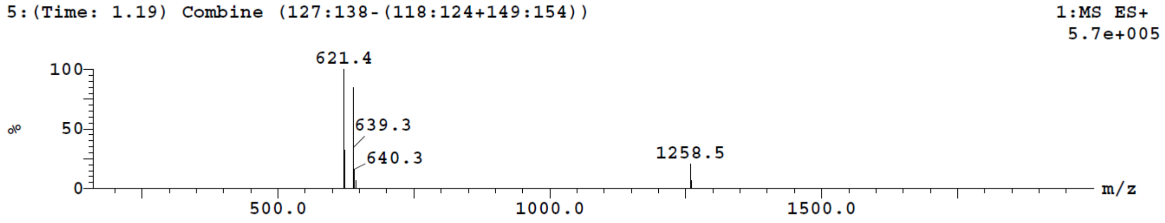

Compound **S22**

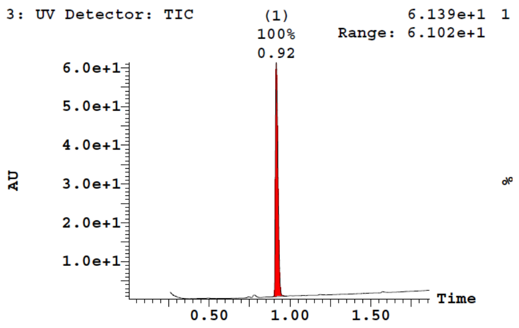

Peak ID	Time	Mass Found
1	0.92	

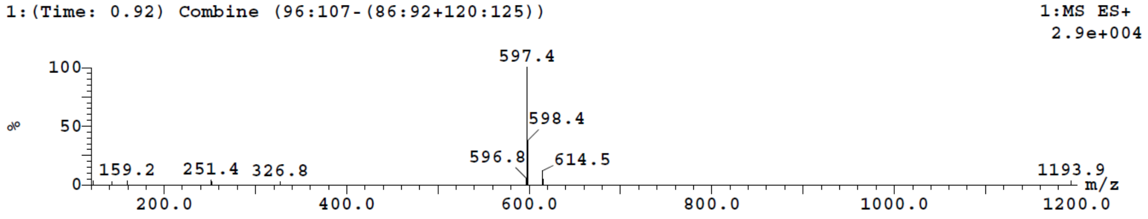

Compound **S23**

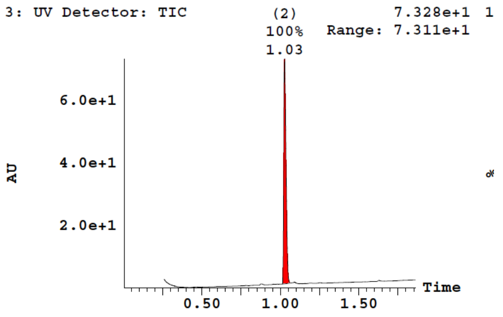

Peak ID	Time	Mass Found
2	1.03	

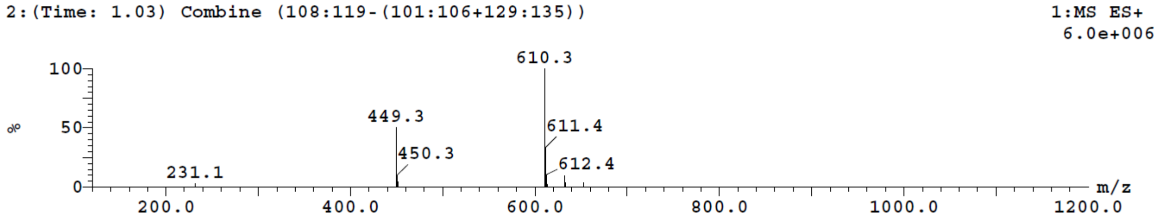

Compound **S24**

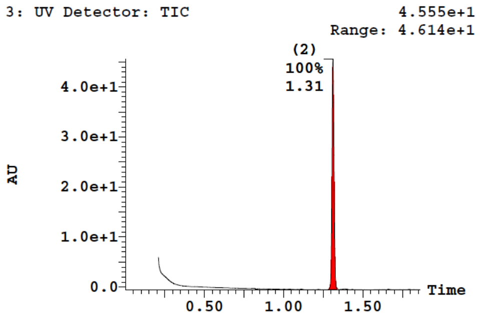

Peak ID	Time	Mass Found
2	1.31	

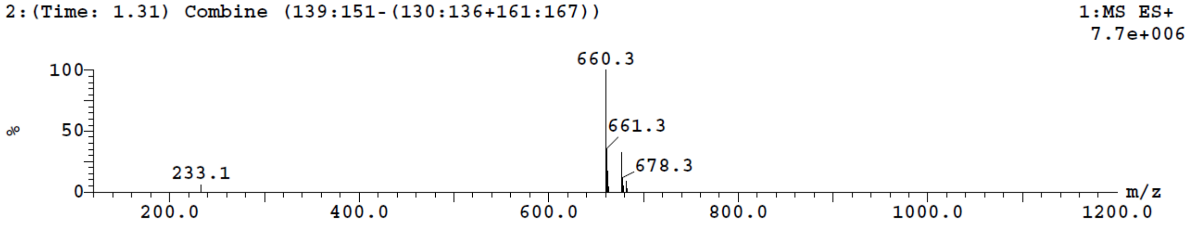

Supplementary References

(1) Marelius, J.; Kolmodin, K.; Feierberg, I.; Åqvist, J. Q: A Molecular Dynamics Program for Free Energy Calculations and Empirical Valence Bond Simulations in Biomolecular Systems. *J. Mol. Graph. Model.* **1998**, *16*, 213–225.

(2) Jorgensen, W. L.; Maxwell, D. S.; Tirado-Rives, J. Development and Testing of the OPLS All-Atom Force Field on Conformational Energetics and Properties of Organic Liquids. *J. Am. Chem. Soc.* **1996**, *118*, 11225–11236.

(3) Jorgensen, W. L.; Chandrasekhar, J.; Madura, J. D.; Impey, R. W.; Klein, M. L. Comparison of Simple Potential Functions for Simulating Liquid Water. *J. Chem. Phys.* **1983**, *79*, 926–935.

(4) King, G.; Warshel, A. A Surface Constrained All-Atom Solvent Model for Effective Simulations of Polar Solutions. *J. Chem. Phys.* **1989**, *91*, 3647–3661. https://doi.org/10.1063/1.456845.

(5) Ryckaert, J. P.; Ciccotti, G.; Berendsen, H. J. C. Numerical Integration of the Cartesian Equations of Motion of a System with Constraints: Molecular Dynamics of n-Alkanes. *J. Comput. Phys.* **1977**, *23*, 327–341.

(6) Lee, F. S.; Warshel, A. A Local Reaction Field Method for Fast Evaluation of Long-Range Electrostatic Interactions in Molecular Simulations. *J. Chem. Phys.* **1992**, *97*, 3100–3107.

(7) Beutler, T. C.; Mark, A. E.; van Schaik, R. C.; Gerber, P. R.; van Gunsteren, W. F. Avoiding Singularities and Numerical Instabilities in Free Energy Calculations Based on Molecular Simulations. *Chem. Phys. Lett.* **1994**, *222*, 529–539.

(8) Zwanzig, R. W. High Temperature Equation of State by a Perturbation Method. I. Nonpolar Gases. *J. Chem. Phys.* **1954**, *22*, 1420–1426.

(9) Matricon, P.; Ranganathan, A.; Warnick, E.; Gao, Z. G.; Rudling, A.; Lambertucci, C.; Marucci, G.; Ezzati, A.; Jaiteh, M.; Dal Ben, D.; Jacobson, K. A.; Carlsson, J. Fragment Optimization for GPCRs by Molecular Dynamics Free Energy Calculations: Probing Druggable Subpockets of the A2A Adenosine Receptor Binding Site. *Sci. Rep.* **2017**, *7* (6398).

(10). Begnini, F.; Poongavanam, V.; Over, B.; Castaldo, M.; Geschwindner, S.; Johansson, P.; Tyagi, M.; Tyrchan, C.; Wissler, L.; Sjo, P.; Schiesser, S.; Kihlberg, J., Mining Natural Products for Macrocycles to Drug Difficult Targets. *J. Med. Chem.* **2021,** *64* (2), 1054-1072.
